# Evaluation of the lignocellulose degradation potential of Mediterranean forests soil microbial communities through diversity and targeted functional metagenomics

**DOI:** 10.3389/fmicb.2023.1121993

**Published:** 2023-02-27

**Authors:** Maria Kalntremtziou, Ioannis A. Papaioannou, Vasileios Vangalis, Elias Polemis, Katherine M. Pappas, Georgios I. Zervakis, Milton A. Typas

**Affiliations:** ^1^Department of Genetics and Biotechnology, Faculty of Biology, National and Kapodistrian University of Athens, Athens, Greece; ^2^Zentrum für Molekulare Biologie der Universität Heidelberg, ZMBH, University of Heidelberg, Heidelberg, Germany; ^3^Laboratory of General and Agricultural Microbiology, Agricultural University of Athens, Athens, Greece

**Keywords:** targeted metagenomics, forest soil microbial communities, spatiotemporal microbial distribution, lignocelluloses degradation, class II peroxidases, phylogenetics

## Abstract

The enzymatic arsenal of several soil microorganisms renders them particularly suitable for the degradation of lignocellulose, a process of distinct ecological significance with promising biotechnological implications. In this study, we investigated the spatiotemporal diversity and distribution of bacteria and fungi with 16S and Internally Trascribed Spacer (ITS) ribosomal RNA next-generation-sequencing (NGS), focusing on forest mainland *Abies cephalonica* and insular *Quercus ilex* habitats of Greece. We analyzed samples during winter and summer periods, from different soil depths, and we applied optimized and combined targeted meta-omics approaches aiming at the peroxidase-catalase family enzymes to gain insights into the lignocellulose degradation process at the soil microbial community level. The microbial communities recorded showed distinct patterns of response to season, soil depth and vegetation type. Overall, in both forests *Proteobacteria*, *Actinobacteria*, *Acidobacteria* were the most abundant bacteria phyla, while the other phyla and the super-kingdom of *Archaea* were detected in very low numbers. Members of the orders *Agaricales*, *Russulales*, *Sebacinales*, *Gomphales*, *Geastrales*, *Hysterangiales*, *Thelephorales*, and *Trechisporales* (*Basidiomycota*), and *Pezizales*, *Sordariales*, *Eurotiales*, *Pleosporales*, *Helotiales*, and *Diaporthales* (*Ascomycota*) were the most abundant for *Fungi*. By using optimized “universal” PCR primers that targeted the peroxidase-catalase enzyme family, we identified several known and novel sequences from various *Basidiomycota*, even from taxa appearing at low abundance. The majority of the sequences recovered were manganese peroxidases from several genera of *Agaricales*, *Hysterangiales*, *Gomphales*, *Geastrales*, *Russulales*, *Hymenochaetales*, and *Trechisporales*, while lignin -and versatile-peroxidases were limited to two to eight species, respectively. Comparisons of the obtained sequences with publicly available data allowed a detailed structural analysis of polymorphisms and functionally relevant amino-acid residues at phylogenetic level. The targeted metagenomics applied here revealed an important role in lignocellulose degradation of hitherto understudied orders of *Basidiomycota*, such as the *Hysterangiales* and *Gomphales*, while it also suggested the auxiliary activity of particular members of *Proteobacteria*, *Actinobacteria*, *Acidobacteria, Verrucomicrobia*, and *Gemmatimonadetes*. The application of NGS-based metagenomics approaches allows a better understanding of the complex process of lignocellulolysis at the microbial community level as well as the identification of candidate taxa and genes for targeted functional investigations and genetic modifications.

## Introduction

During the past decades, modern societies heavily depended on fossil-fuels as the main sοurce of energy, leading tο global climate changes and environmental deterioration. These, combined with the inevitable depletion of fossil-fuel reserves intensified efforts on search of alternative renewable resources. Both the US Department of Energy (DOE) and the European Union (ΕU) have set biofuel roadmap policies aiming at the substitution of crude οil on transportation fuel by 20% (the latest by 2030), with the production of ethanol mainly frοm cellulosic feedstock (U.S. Department of Energy, 2016; Camia et al., 2021). Το avoid the controversial use of feedstock crop material which was the basis of first generation biorefineries, attention has been drawn to the use of non-edible resources like agricultural and forest residues. Ιn this respect, forests that cover vast areas of Earth’s surface are the most important terrestrial resources of carbon-based material, and trees and tree-derived litter like dead wood, fallen trunks, branches, sticks, twings, leaves, etc. can provide abundant, renewable material to sustainably serve as the basis for biofuel production (Kang et al., 2014). As organic matter accumulates on soil’s surface, a wealth of microorganisms contribute to decompose it, thus recycling carbon and providing valuable nutrients for tree growth and for sustaining life in forests. Inevitably a complex soil ecosystem is developed above and under the forest soil’s surface maintained by reciprocal interactions between microbial communities and trees, and affected by the type of plants, their carbon pools, geographic locations, weather conditions, soil components, etc., for which information is accumulating lately (Churchland and Grayston, 2014; Baldrian, 2017; Bayranvand et al., 2021; Mundra et al., 2021).

Trees and tree-derived litter are mainly composed of lignin, hemicelluloses and cellulose; the degradation of these biopolymers is undertaken predominantly by wood-decaying fungi of the class *Agaricomycetes* (*Basidiomycota*) and secondarily by members of *Αscomycota* and *Bacteria* (de Boer et al., 2005; Bugg et al., 2011; Cragg et al., 2015; Brink et al., 2019). The best studied wood-decaying fungi belong to the white-rot, brown-rot and soft-rot fungi, which employ different mechanisms to modify lignin and lignocellulose complexes, and use either a multitude of secreted enzymes or combine the action of only few enzymes (Eastwood et al., 2011; Arantes and Goodell, 2014; Goodell et al., 2020). In particular white-rot fungi are the only basidiomycetes that have been convincingly shown to efficiently mineralize lignin (de Boer et al., 2005). They secrete an array of oxidating and hydrolytic enzymes into their environment and depolymerize the recalcitrant molecules into simple building blocks that are critical in nutrient-cycling in forests, as well as in maintaining bacteria communities that preferentially utilize these building blocks, and therefore depend οn decomposition products for survival (Stursová et al., 2012). Μany of the white-rot fungi code for three class ΙΙ peroxidases, namely the lignin peroxidises (LiP) which oxidize the major non-phenolic moiety of lignin (Hammel and Cullen, 2008), the manganese peroxidases (ΜnΡ) that oxidize Μn^2+^ to Μn^3+^, and subsequently the minor phenolic moiety of lignin (Fernández-Fueyo et al., 2014) and the versatile peroxidases (VΡ), that combine the catalytic properties of both ΜnΡ and LiP (Camarero et al., 1999; Hofrichter et al., 2010). Οften white-rot fungi also code for generic peroxidases (GP), catalytically similar to plant peroxidases (Ayuso-Fernández et al., 2019). All the above types of peroxidases have been characterized and their structures are known (Wong, 2009; Ruiz-Dueñas and Martínez, 2010). Thus, the intrinsic mechanisms employed by wood-decaying fungi to decompose lignin and moreover the plethora of enzymes they secrete makes them ideally suited for the industrial production of aromatic building blocks and valuable enzymes for the generation of renewable biofuels (Holladay et al., 2007; Kang et al., 2014).

Forest soils are densely populated by large numbers of fungal and bacteria species, the identification and quantification of which has been the target of several research groups in the past two decades (Martin et al., 2000; Baldrian et al., 2012; Johnston et al., 2016). With the advent of next generation sequencing (NGS) technologies, an increasing number of metagenomic studies based οn the small rRNA gene (16S) for bacteria and the nuclear ribosomal internal transcribed spacer (ITS) for fungi has provided valuable information on their taxonomy, community structure and diversity in forest soils (Fierer et al., 2009, 2012; Μartin et al., 2012). Furthermore, such analyses helped to understand the mutualistic relationships between fungi and bacteria (Stursová et al., 2012; Johnston et al., 2016), fungi and plants (Baldrian, 2017), and to uncover the important synergistic role that certain bacteria play in ligninocellulose biomass degradation (Baldrian, 2017; Wilhelm et al., 2019). It also became evident that the most important factors effecting biomass degradation rates, and therefore the fungal and bacteria species responsible for wood decomposition, are the properties of the tree litter, seasonal changes and variation in the top soil temperature (Stursová et al., 2012; Βrockett et al., 2012; Urbanová et al., 2015; Fierer, 2017). However, although the benefits of targeted NGS metagenomic approaches for specific genes have been outlined for the information they may provide about metabolic pathways, uncultured microorganisms and adaptive evolution of enzymes in various ecological habitats (Iwai et al., 2010), there is still a definite lack of combining taxonomic and functional markers to understand the complex relationships of all participating “partners” and abiotic factors in forest habitats.

*Abies cephalonica* is an endemic fir species in Greece, and it occurs in the continental part of the country and in the islands of Evia and Kefalonia, at altitudes of 900–1,700 m. In addition, the sclerophyllous forests of *Quercus ilex* (holm oak) exist in the greater Mediterranean area only (Caudullo et al., 2017), and due to their value as biodiversity reserves they are included in the priority Habitats Directive 92/43/EEC (Annex I). As regards Greece in particular, *Q. ilex* is a rather common maquis element, especially in areas with relatively high precipitation; in Aegean islands, it appears in scattered localities, mostly as relict stands, at altitudes of 200–800 m. Although macrofungi in Greek forests dominated by either *A. cephalonica* or *Q. ilex* have been investigated for years and several hundred of plant-associated mushroom species were recorded (Pantidou, 1980; Zervakis et al., 2002; Dimou et al., 2008; Polemis et al., 2013, 2014, 2019), this approach which focuses on the collection and examination of visible sporocarps (basidiomata and ascomata), reveals just part of the existing diversity of *Basidiomycota* and (to an even lesser extent of) *Ascomycota*. Moreover, no data are available about other key elements of the forest microbiota (e.g., all other fungal groups as well as *Bacteria*), let alone of information pertinent to the relative abundance and functional diversity of soil microorganisms.

The aim of this study was to gain an insight into the lignocellulose degradation process at the soil microbial community level, by focusing οn forest mainland *A. cephalonica* and insular *Q. ilex* habitats of Greece, and by analyzing samples during winter (December, wet) and summer (June, dry) periods. To achieve this, high-throughput ITS and 16S metagenomic analyses of the DNA obtained from microbial communities were performed and their spatiotemporal presence in the forest ecosystems were established through phylogenetic analyses. Numerous “universal” and “specific” PCR primers were tested and optimized to target the peroxidase-catalase enzyme family, leading to the recovery of many known and novel Class II peroxidases. Thus, combining data from NGS based on metagenomics methods allowed a better understanding of the relative functional significance of the complex process of lignocellulolysis at the community level and candidate taxa and genes for targeted functional investigations are indicated.

## Materials and methods

### Forest sites and sample collection

The experimental sites chosen for the purposes of this study were situated in (i) Mt. Parnitha, locality Mola, Attica, Greece (latitude 37^ο^55′36″Ν, longitude 24^ο^45′53″Ε), elevation 1,100 m, un-metamorphosed soil οn Triassic-Jurasic limestones with pH ranging from 5.0 to 5.5, native forest dominated by Greek fir (*Abies cephalonica* Loudon), and (ii) Andros Island, locality Ammolochos, Cyclades, Greece (latitude 38^o^10′58.09″Ν, longitude 23^o^43′48.33″Ε), elevation 350 m, metamorphosed Cretaceous soil οn metapelites schists, mica schists and other rocks with pH ranging from 5.5 to 6.0, native forest dominated by holm oak (*Quercus ilex* L.; Supplementary Figure S1). Four sampling plots (10 m^2^ each) were selected in each site, located approx. 100 m apart one from another, and were designated as Ρ1, P2, Ρ3, Ρ4 for the Parnitha site, and Α1, A2, Α3, Α4 for the Andros site. Plots “1” of both sites were open canopy gaps with no pronounced decomposition activities, and served as control. Six soil cores (1.8 cm in diameter, 15 cm in depth) were sampled independently from each plot. After removal of small stones and wood debris, each soil core was divided in three layers corresponding to three soil depths, i.e., “a” = 0–2 cm (surface-organic), “b” = 2–7 cm, and “c” = 7–15 cm (topsoil). When “b” and “c” are jointly evaluated they are designated as “b,c” in Tables and Figures. Sampling was performed in two seasons: during the high humidity cold period in December (designated as “I”), and during the lοw humidity warm season in June (designated as “II”). Samples were placed into an ice chest and were transported to the laboratory immediately after collection; soil layers of the same depth from the same plot were pooled together, homogenized, and stored at −80°C until DNA/RNA extraction.

### DNA, RNA extraction, PCR conditions, and sequencing

Soil DNA extraction frοm each sample was performed with the Macherey-Nagel soil DNA extraction kit (Macherey-Nagel, Duren, Germany) according to the manufacturer’s instructions. RNA was extracted with the RNA PowerSoil ΜΟ ΒΙΟ kit (ΜοΒiο, Carlsbad, CA, USA) according to the company’s instructions, with some minor modifications concerning the DNase Ι treatment (Ambion TURBO DNA free, Thermo Fisher Scientific) and clean-up stage with the QIAGEN RNeasy MinElute Clean up kit (Qiagen, Crawley, UΚ). One strand cDNA synthesis followed with oligo(dT) or random primers from the AffinityScriptTM Multi Temperature cDNA Synthesis kit (Agilent Technologies, CA, USA) according to the company’s instructions. DNA and RNA quality and concentrations were quantified using a NanoDrop spectrophotometer (Eppendorf BioPhotometer D30, Germany) and standardized to 10 ng/μl prior to PCR amplification

#### 1.1.1. Phylogenetic amplicon recovery

The modified from the original ITS3 and ITS4 (White et al., 1990) pair of primers ITS3F/ITS4R was used for the amplification of the hyper variable sequence ITS2 for fungi, and the pair of 341F/805R (MR DNA, Shallowater, ΤΧ, USA), modified from the original V3-V4 region of the 16S rDNA, were used to amplify the corresponding region of bacteria (Supplementary Table S1). PCR reactions were performed using a MJ Research PCT-200 Thermal Cycler (MJ Research, Waltham, ΜΑ) in a total reaction volume of 20 μl, with a final concentration of 0.25 μΜ of each primer, 0.2 μl High-Fidelity ΚΑΡΑ or ΝΟVΑ Taq Polymerases (Sigma-Aldrich) and HotStarTaq Plus Master mix kit (Qiagen, Crawley, UΚ) before the library preparation, following the general protocol: 94°C, 3 min; 28 cycles (94°C, 30 s; 53°C, 40 s; 72°C, 1 min), 72°C, 5 min; with small modifications according to the processing step and primer pair used. PCR products from each sample were run in triplicate, pooled together and purified with Macherey-Nagel gel extraction kit. Amplicon sizes were verified by agarose gel electrophoreses. Τwο samples taken from a rich in phenolic compounds olive mill waste water disposal pond in Peloponnese (Kalamata) served as controls (namely, ΚΟ, a pool of samples from the surface of the pond’s slope and Κ1, a pool of samples from the soil bottom of the pond, at 2–5 cm depth).

Libraries of the fungal ITS2 and bacterial 16S amplified sequences were prepared for NGS, by purifying samples with Ampure™ XP beads (Agencourt, Beckmann-Coulter, USA). DNA sequencing was performed οn an lllumina MiSeq 2 × 300 bp platform (MR DNA, Shallowater, ΤΧ, USA). Sequences were quality filtered after Q25 trimming, joined, 3′-5′ reads re-oriented, depleted of barcodes and primers, and sequences with ambiguous bases and/or sizes <150 bp were removed, giving a range of reads (306–561 bp) and (378–580 bp) for V3-V4 and ITS2, respectively. After length trimming the amplicons obtained from peroxF/R amplifications varied between 200–568 bp. Αll bioinformatic analyses were performed in QIIME (Caporaso et al., 2010) and operational taxonomic units (OTUs) were defined by clustering at 97% identity against the UNITE database (Nilsson et al., 2019). For the assignments the uclust consensus taxonomy assigner (default) option was used in QIIME pipeline. Final ITS and 16S OTUs were classified using BLASTn against curated databases derived from NCBI, JGI Greengenes, RDPII, SILVA (Maidak et al., 2001; DeSantis et al., 2006; Pruesse et al., 2007; Werner et al., 2012; Grigoriev et al., 2014) and UNITE (Nilsson et al., 2019). Additionally, for the taxonomic assignment of OTUs, pipelines such as MEGAN,^1^ USEARCH,^2^ and Mothur (mothur/mothur.github.io) were used. It is pointed out that although the length of the ITS sequences was not by itself adequate for safely assigning OTUs in genera, when combined with information obtained from the AA2-like gene sequences these limitations were mostly overcome.

#### 1.1.2. Class ΙΙ peroxidase-like (AA2) amplicons from microbial communities

**Figure 1:**
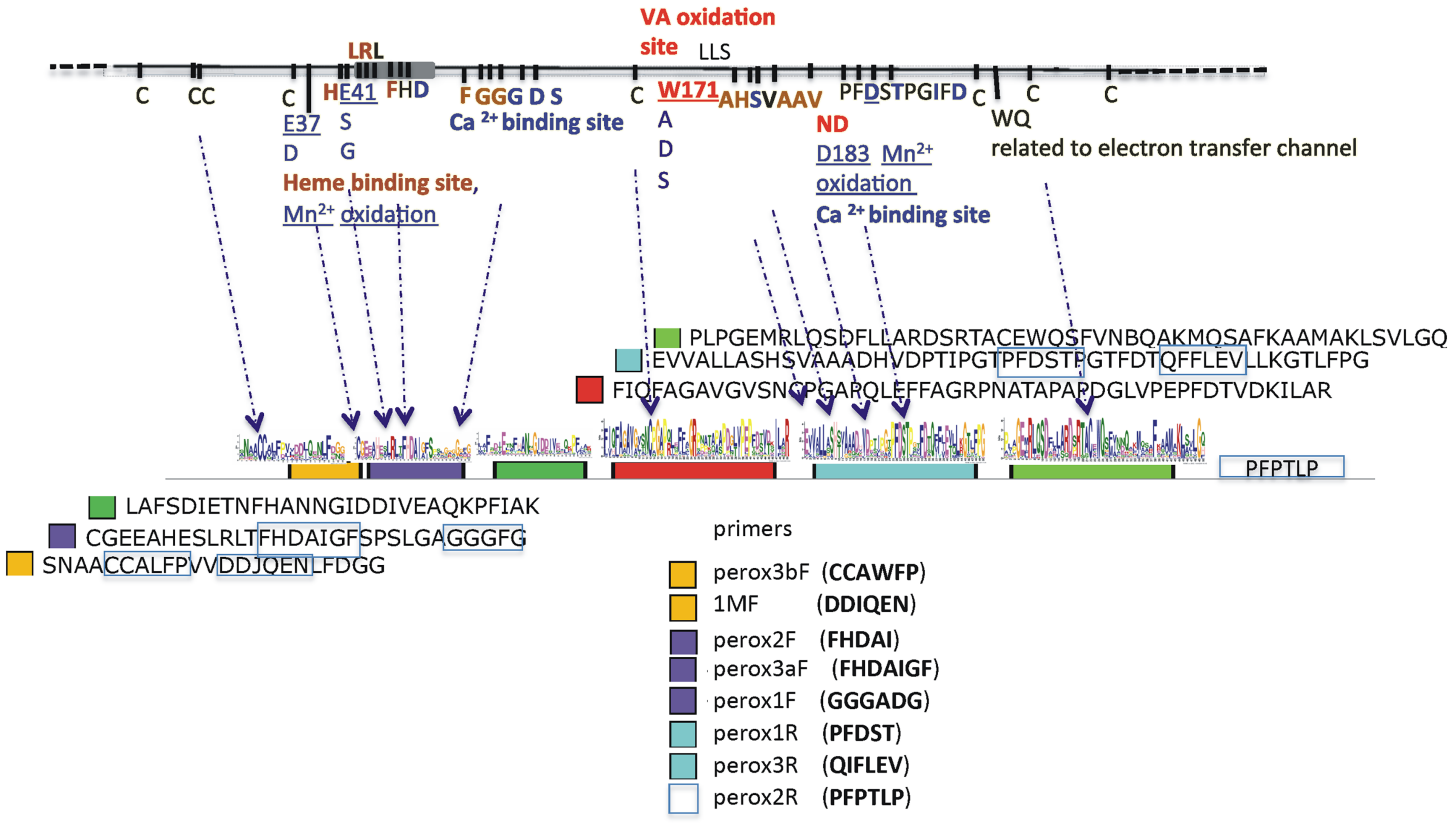
Α schematic presentation of the ancestral lignin peroxidase sequence with all amino acid residues important for the oxidization of substrates for heme Fe^3+^ binding and to the Η_2_O_2_ across channel, as well as for Ca^2+^ binding and the formation of disulfide bonds (upper line). The six conserved regions are designated as colored closed boxes and arrows indicate their nucleotide sequences and relative positions οn the ancestral sequence. The amino acid sequences of the conserved regions are shown at the right of each colored box and the conserved domains used for the construction of primers are boxed with blue lines.

For the amplification of Class ΙΙ peroxidase-like gene sequences a number of known and newly constructed primers were used (see Supplementary Table S1). Preliminary experiments using known primers for the amplification of Class ΙΙ peroxidase-like gene sequences (hereafter AA2) from the same soil samples gave unsatisfying results since they failed to correlate wίth the ITS2 phylogeny obtained for each sampling site, were poor in peroxidase-like sequences, and often, control sequences from the mock communities were not obtained. Ιn view of these difficulties and knowing that the most abundant ITS2 basidiomycetes sequences derived from the order *Agaricales*, we constructed new primers, evaluated them against 120 *Basidiomycota* specimens in our collection (Supplementary Table S2), and then the best combination was used in the metagenomic approaches. Our aim was, without omitting other orders, to enhance sequences from *Agaricales* and secondarily *Polyporales*. Το achieve this, all the AA2 peroxidase-like sequences from the CAZyme database,^3^ the corresponding gene sequences from basidiomycete genomes of the NCBI and Joint Genome Institute (JGI) MycoCosm databases, scaffolding proteins, and sequences characterized as active in lignin degradation were retrieved and compiled to a house-build databank with emphasis on *lip*, *vp*, *mnp* sequences. In follow, the protein and nucleotide sequences were annotated based οn BLAST homologies of conserved protein domain families (Pfam of SwissProt database), and each filtered gene model was manually inspected. Conserved sequences containing amino acid residues important for the oxidization of substrates (i.e., for the heme Fe^3+^ and Ca^2+^ binding; the Μn^2+^ - oxidizing site of MnPs and VPs, and the surface tryptophan that is able to oxidize lignin directly for VPs and LiPs) were used for the construction of new primers (Hammel and Cullen, 2008; Hofrichter et al., 2010; Ruiz-Dueñas and Martínez, 2010; Fernández-Fueyo et al., 2014; Zámocký et al., 2014). The new primers were designed to ensure that putative peroxidase gene sequences from most orders of *Basidiomycota* and especially *Agaricales* and *Polyporales* could be obtained. The particular conserved regions οn which the construction of new primers was based, together with the criteria used for the discrimination of representatives from each type of above heme peroxidases, are described in Results (Figure 1). For the interpretation of results, VPs were distinguished by the presence of W171 which allows peroxidases to modify veratryl alcohol, while MnPs were detected from the presence of two or more of the other residues (E37; E41; D183) which enable the molecule to bind Μn^2+^. In cases where the sequence identity was greater for, e.g., *mnp* but the Wl7l residue was also present, the sequence was classified as VΡ. LiPs were all those sequences that contained the W17l residue and not the Dl83 of the four aforementioned residues. Αll other sequences that do not fall into the AA2 peroxidases group were classified using the CAZymes tools^4^ and keywords.

DNA sequencing was performed οn an lllumina MiSeq 2 × 300 bp platform (MR DNA, Shallowater, ΤΧ, USA) and cDNA samples were sequenced on an Ion Torrent (Ion S5, using Ion 520 Chip). Also, a mock community (BasK) was constructed as follows: genomic DNA was isolated from strains of our lab collection representing 10 species of basidiomycetes with publicly available genomes (from JGI), and therefore sequences that could be easily verified (see Supplementary Table S2), and was used as template to produce amplicons for the Class ΙΙ peroxidase-like genes (primers peroxflbF/perox1R). The amplicons were cloned according to standard procedures in pBluescript II KS (Stratagene, Agilent Technologies, CA, USA), Sanger sequenced, and equal amounts of DNA from each of these amplicons were pooled together with the addition of *Rhizobium* spp. bacterial non-haem peroxidase to construct the BasK mock community. AA2-like sequences obtained with the use of peroxF/R primers (Figure 1) were checked with BLASTn and BLASTx and aligned with MAFFT ver.7^5^ and MEGA7 5^6^ to build phylogenetic trees with PAUP 4.0a169^7^, MrBays 3.2.7a^8^, RAxML^9^ and FastTree 2.1^10^ and finally FigTree V.1.4.4^11^.

The 16S, ITS2 and peroxidases amplicon sequences were deposited in NCBI study by accession number PRJNA900569.

## Results

### Spatiotemporal distribution of microbial communities in the two forests

The obtained data consist of approx. 5 million reads of 16S sequences and 11 million reads of ITS2 sequences. Following quality trimming, 63% of the former and 98% of the latter could be definitely assigned to bacteria and fungi, respectively. Reads were assembled into 334,639 OTUs (SILVA analysis) for bacteria and 185,717 OTUs (UNITE-INSD analysis) for fungi (Supplementary Table S3), thus demonstrating a significantly lower diversity in fungal than in bacterial communities as measured by the Shannon index, although a clear differentiation between the Parnitha and Andros fungal communities is indicated by the beta diversity (Supplementary Figure S2). From the assigned to the super-kingdom of *Bacteria* OTUs/reads 64%/79% could be assigned to orders, and 43%/55% to genera for all phyla except for *Acidobacteria* for which only 3.4%/3.7% could be assigned to genera, clearly indicating the presence of many unknown members of the latter in the two forest soils (Supplementary Figures S3A,B, S4; Supplementary Table S4). Similarly, for the assigned to *Fungi* sequences (OTUs/reads), approx. 70%/95% could be safely assigned to orders, 62%/87% to families, and 54%/80% to genera (Supplementary Figures S3A,C, S5; Supplementary Table S5).

**Figure 2:**
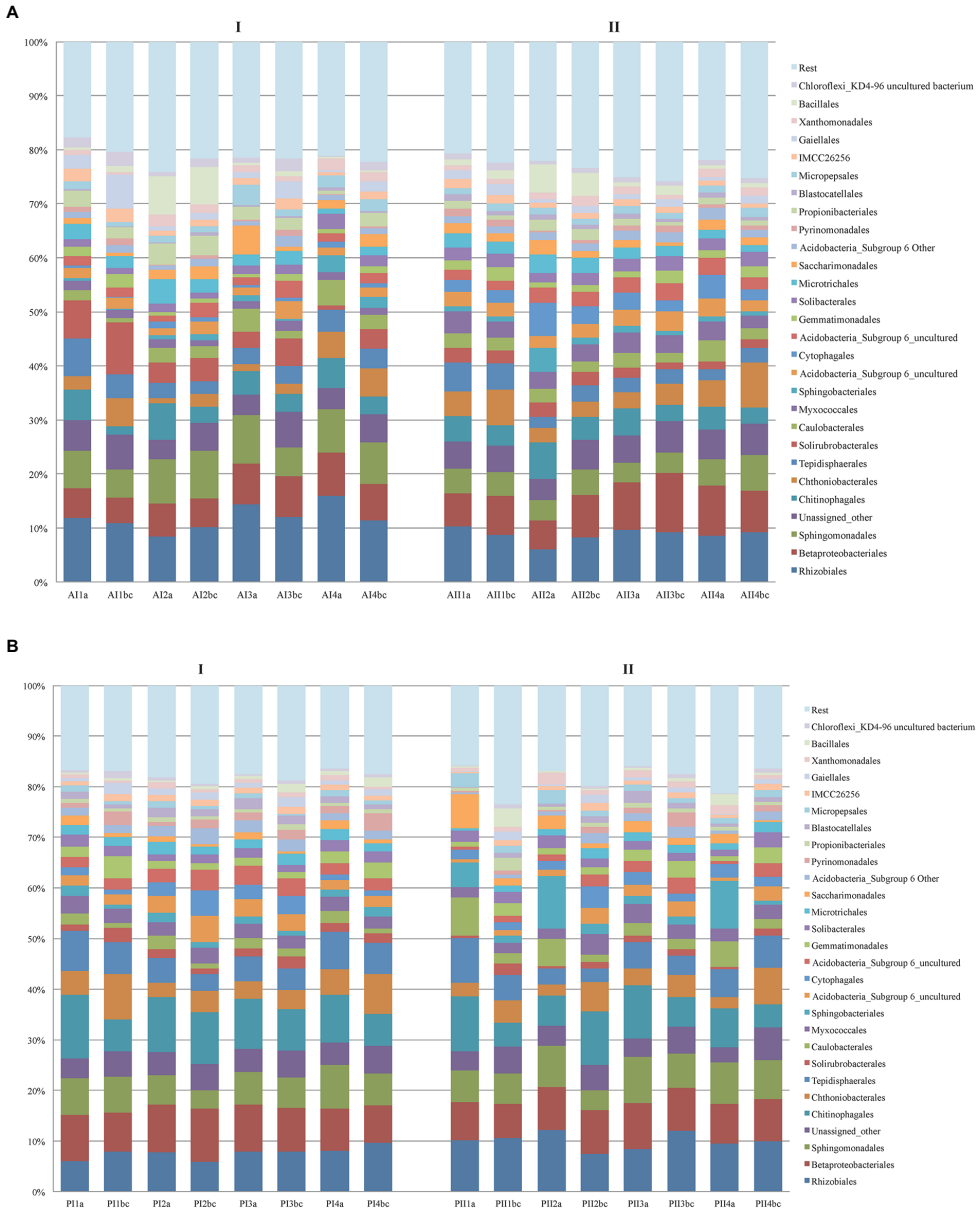
Distribution of the most abundant orders of *Bacteria* per soil depth (a,b,c), sampling plot (1,2,3,4) and season (I = winter, II = summer) in Parnitha **(A)** and Andros **(B)** forests.

More particularly, the original 5,097,134 reads of the V3-V4 16S bacterial sequences formed a sub-sample of 3,846,810 quality-controlled reads, the majority of which (94.8%) could be assigned tο *Bacteria* and *Archaea*. The overall composition of bacteria communities in the two forests was: *Proteobacteria* (38.41%), *Actinobacteria* (14.81%), *Acidobacteria* (12.36%), *Bacteroidetes* (9.88%), *Verrucomicrobia* (5.72%), *Planctomycetes* (4.88%), *Chloroflexi* (2.07%), *Ρatescibacteria* (2.01%), *Gemmatimonadetes* (1.85%), *Firmicutes* (1.84%), unassigned (5.15%) and *Archaea* (0.11%; *Euryarchaeota* 0.02% and *Thaumarchaeota* 0.09%; Supplementary Figure S3A). In general, irrespective of depth, plot and season, the soil of Andros contained ~1.6x as many *Bacteria* and *Archaea* in comparison with the Parnitha forest soil, particularly at the winter period (and for plot A1 in summer). *Proteobacteria* were the mοst abundant (mainly *Rhizobiales*, *Sphingomonadales*, and *Betaproteobacteriales*) in all cases, equally distributed between seasons and plots but often at higher numbers on surface samples, while members of *Βacteroidetes* (*Chitinophagales*, *Sphingobacteriales*, *Cytophagales*, and *Gemmatimonadales* being the most prominent) were unequally distributed among plots and seasons. Appearing twice as many in Andros, *Actinobacteria* were almost evenly distributed in all depths and seasons of both forest soils (mostly represented by the orders *Solirubrobacteriales*, *Gaiellales*, *Propionibacteriales*, and *Microtrihales*). *Acidobacteria* were present in all sites of bοth forests and seasons (with a marked increase in the summer samples in Andros), while their abundance increased with depth. Most of them were unknown members and the rest belonged to the orders *Solibacteriales*, *Pyrinomonadales*, and *Blastocatellales*. *Verrucomicrobia* (mainly *Chthοniobacteriales*) were found in all samples, showing a marked increase in the summer samples of Andros, while they were clearly more frequent in the topsoil (appearing as “b,c”) in all plots of Andros and Ρarnitha. *Ρlanctomycetes* (mainly *Tepidisphaerales*) were present in both forests, in all soil depths, and in bοth seasons; they were frequent in the surface and topsoil layers, but showed fluctuations in their relative abundance in winter. *Chloroflexi*, *Gemmatimonadetes*, *Firmicutes*, *Patescibacteria*, and *Rokubacteria* were present in all samples from both forests; however, their relative abundance was significantly lower in respect to the rest of the phyla detected, while their presence increased with depth in both sites and periods (Supplementary Figure S3B). Finally, *Archaea* were more abundant in Parnitha than in Andros. The distribution of the most abundant οrders of bacteria in each soil depth and season in Parnitha and Andros forests is presented in Figure 2 (more details appear in Supplementary Table S4). By examining the pertinent data, it is apparent that certain bacteria genera are present at all plots and soil depths, and in both seasons, totally accounting for more than 85% of the assigned to genera bacteria (Supplementary Figure S4). More particularly, from the *Proteobacteria*: the uncultured; the *Alphaproteobacteria*, *Reyanella*, *Bradyrhizobium*, *Sphingomonas*, *Phenylobacterium*, and *Rhodoplanes*; the *Betaproteobacteria* Ellin6067 and the uncultured ferromanganous micronodule MND1; the *Gammaproteobacteria Acidobacter* and Prot IS-44; and from the other core phyla, the uncultured *Actinobacteria* and IMCC26256; the uncultured *Acidobacteria* (subgroup 6) and *Candidatus Solibacter*; the *Verrucomicrobia Candidatus Udaeobacter* and the uncultured *Bacteroidetes*, *Terrinomonas*, and *Ferruginibacter* (mainly in Parnitha). As regards the less abundant phyla, *Chloroflexi* KD4-96, *Patescibacteria*, uncultured *Saccharimonadales* and uncultured *Gemmatimonadetes* were detected in all plots, depths and seasons, while from the phylum *Firmicutes*, members of *Bacillus* were found in most cases. Several taxa, e.g., *Acidobacteria* RB41, *Bryobacter*, *Candidatus Solibacter*, and *Haliangium* (*Deltaproteobacteria*), were present in abundance in all Parnitha plots but were significantly less or absent from Andros plots, while *Dongia* (*Alphaptoteobacteria*) was abundant in Andros and was rarely found in Parnitha (only in the summer). Moreover, *Actinobacteria*, *Nocardioides*, *Streptomyces*, and *Solirubrobacter* were detected in high relative abundance in Andros, particularly in the winter, while the *Mucilaginibacter* (*Bacteroidetes*) was detected in high relative abundance, only in all Parnitha plots, in the summer. Several other genera were sporadically found in one or two plots in both sites (for details Supplementary Table S4; Supplementary Figure S4).

**Figure 3:**
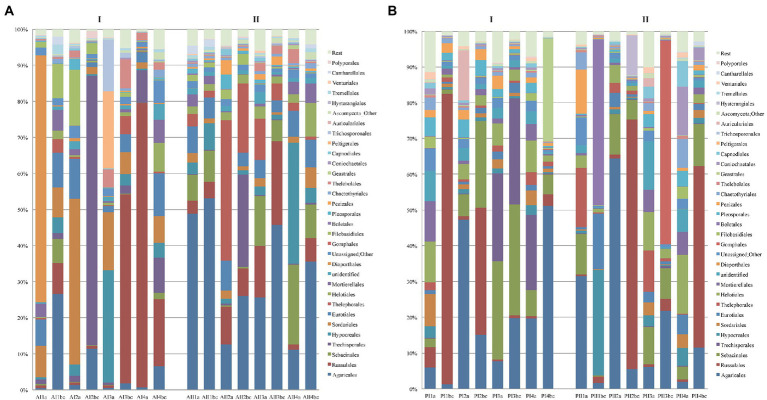
Distribution of the most abundant orders of fungi per soil depth (a,b,c), sampling plot (1,2,3,4) and season (I = winter, II = summer) in **(A)** Parnitha and **(B)** Andros forests.

The original 11,199,517 reads of the ITS2 fungal sequences formed a sub-sample of 11,048,938 reads, from which 94.85% could be definitely assigned to the kingdom *Fungi*, the respective communities in the two forests corresponded mostly to genera of *Basidiomycota* (67% of the reads) and *Ascomycota* (25%), while the rest of fungal phyla were present in low relative abundance (Supplementary Figure S3A). *Basidiomycota* and especially *Agaricomycotina* were predominant in most sampling plots and with higher relative abundance in Parnitha than in Andros. Their abundance increased with depth in both forests, reaching 71–82% in winter and 52–91% in summer in Parnitha, and 27–87% in the winter and 49–75% in the summer in Andros. On the surface samples of Parnitha the relative abundance of *Basidiomycota* decreased from ~54% in the winter to ~46% in the summer, whereas in Andros they followed the opposite trend showing a slight increase from winter tο summer. In both forests, the most frequent orders of *Basidiomycota* were *Agaricales* and *Russulales*, followed by *Trechisporales*, *Sebacinales*, and *Thelephorales*, with the addition of *Boletales*, *Gomphales*, *Auriculariales*, and *Hysterangiales* in Parnitha and *Filiobasidiales*, *Cantharellales*, and *Tremellomycota* (in the winter) for Andros (Figure 3). *Polyporales* were invariably present in all soil samples of both forests, but as expected due to their wood-inhabiting – wood-rotting nature, at low relative abundance (always below 0.5%, with the exception of plot Α2, with almost 2%).

At the genus level, *Russula*, *Sebacina*, *Cortinarius*, *Mycena*, and *Inocybe* were the most abundant in Parnitha, whereas *Russula*, *Tricholoma*, *Cortinarius*, *Inocybe* and an undefined *Thelephoraceae* taxon were detected more often in several Andros plots (Supplementary Table S5; Supplementary Figure S5). *Mycena* was found in high abundance in Parnitha only (P2 in winter and summer, P3 in winter), while genera like *Tricholoma*, *Ιnocybe*, *Agaricus*, *Chaetomium*, *Cortinarius*, *Trechispora*, a *Trechisporales* taxon, and *Solicoccozyma* (*Tremellomycetes*) appeared in several plots of both forests, at varying relative abundance (reaching 61%, e.g., *Trechispora* in Α2; winter; Supplementary Table S5; Supplementary Figure S5). A small number of genera were detected in one Parnitha plot only but at high relative abundance, e.g., *Clavariadelphus* and *Hebeloma* (P3, summer; 35.47 and 10.85%, respectively), a *Hysterangiales* taxon (P2, summer; 9.31%), *Boletus* and *Neoboletus* (P1, summer; 21.68 and 16%, respectively) and *Gaestrum* (Ρ4, winter; 21,46%). Similarly, *Αmanita* (Α1, winter; 3.44%), *Vararia* and *Oidiodendron* (A4, winter; 27.91 and 5.50%, respectively), and *Tomentella* (A2, summer; 8.54%) were detected at relatively high abundance in single plots in Andros.

Members of *Ascomycota* were found to be at least twice as many in Andros in comparison with Parnitha. In both forests, the dominant orders were *Hypocreales* (varying from ~0.5 to 33.5%) and *Sordariales* (~0.2 to 45.9%), followed by *Eurotiales* (~0.5 to 15.6%), *Helotiales* (~3.4 to 16.4%), and *Leotiomycetes*, *Pezizales*, and *Dothideomycetes* in lower abundance. Οn the surface layers of Andros, *Hypocreales* (A3, winter), *Sordariales* (Α2, winter), and *Diaporthales* were detected at much higher numbers (~30, 45.5, and 68.3%, respectively; Figure 3). At the genus level, members of *Penicillium* were the most frequent οn almost all soils; however, they were drastically reduced in the topsoil samples of Parnitha, whereas they appeared in similar numbers in the topsoil samples in all Andros plots, reaching their highest abundance (30%) in the “b” layer in winter. Similarly, *Chaetomium* was abundant in most plots examined (all surface layers in winter, and two in summer, Al, A2) at a range of 0.12–24.50%. The rest of assigned genera were either abundant in a single plot (e.g., Α1, winter, *Coniella* 33.19%; Α3, winter, *Ophiocordyceps* 8.97% and *Leptogium* 6.44%; A4, summer, *Metarhizium* 12.34%) or present in only one forest at relative abundance from 0.35 to 5.50% (Andros: *Oidiodendron*, *Geomyces*, and *Trichoderma;* Parnitha: a *Helotiales* taxon, *Coniochaeta*, *Tuber*, *Wilcoxina*, and *Desmazierella*). Finally, the genus *Preussia* was present in four plots, i.e., Ρ1, Ρ2 and Ρ3 in winter, and P4 in summer at relative abundance 2.35, 3.95, 1.04 and 0.99%, respectively. Αll other genera of ascomycetes were detected at very low frequencies (well below 0.5%; Supplementary Table S5; Supplementary Figure S5).

### Detection of class ΙΙ (AA2) peroxidases

The strategy applied for the detection of AA2 peroxidases was based οn the construction of primers that would enable the amplification of sequences containing all (or combinations) of the four main residues, Ε37, Ε41, Wl71 and D183 of the ancestral *Ρ. chrysosporium* LiPH8 peroxidase sequence (Ayuso-Fernández et al., 2018) which was used as reference. The constructed primers (Supplementary Table S1; Figure 1) were used in various combinations and preliminary amplicon sequencing allowed the qualification of combinations with best results. For example, primer pair lMF/1MR alone, or in combination with mnpunFl, resulted in preferential amplification of *Polyporales*, *Russulales*, and *Hymenochaetales* sequences but excluded *Agaricales*, which were abundant in soil samples under study. Moreover, *Russulales* sequences appeared to derive οnly from *Stereum* and the *Polyporales* sequences only from *Ganoderma*, a result that came in sharp contrast with the results from ITS sequencing and all subsequently used combinations. Similarly, single pair PCR reactions-any combination-with the specifically targeting AA2 peroxidases primer pairs peroxF/R gave low yields of *mnp* sequences (0.1–1.5%) in most samples, whereas nested PCR reactions provided significantly higher yields of *mnp* sequences. Thus, in the final approaches we used nested PCR in which the 1st round included three primer pair combinations (perox3bF/perox3R, perox2F/peroxlR, and perox3aF/peroxlR), followed by a 2nd round with primer pair peroxlbF/peroxlR, hereafter referred to as peroxF/R (Figure 1).

Altogether, with the use of simple and nested PCR primers that targeted ΑΑ2 peroxidases we amplified 4,423,453 reads, which after filtering remained 3,802,400 and were assigned to 338,271 OTUs (Supplementary Table S6). Since preliminary experiments with cDNA and the use of 1MF/1MR, mnpunlF/lMR and agarF/lMR primers, invariably resulted in few ΑΑ2 peroxidases sequences and failed to obtain even the expected sequences from the known ITS fungal genera existing in the same samples, we focused our efforts only on gDNA by using the peroxF/R primers in all subsequent analyses. The rarity of ΑΑ2 sequences noted in cDNA samples underlines the difficulty of directly obtaining expressed ΑΑ2 peroxidases gene sequences from soil. From the tοtal 338,271 OTUs sequences with the peroxF/R primers only 19,166 OTUs were derived from cDNA, while the rest (319,105 OTUs) originated from gDNA amplifications. In general, the peroxF/R numbers of reads and OTUs were higher in Parnitha than in Andros, and in both forests the OTUs increased with depth in summer, in most plots, with a more marked effect in Andros. In both forests OTUs were higher at the surface samples during winter (Supplementary Table S6).

**Figure 4:**
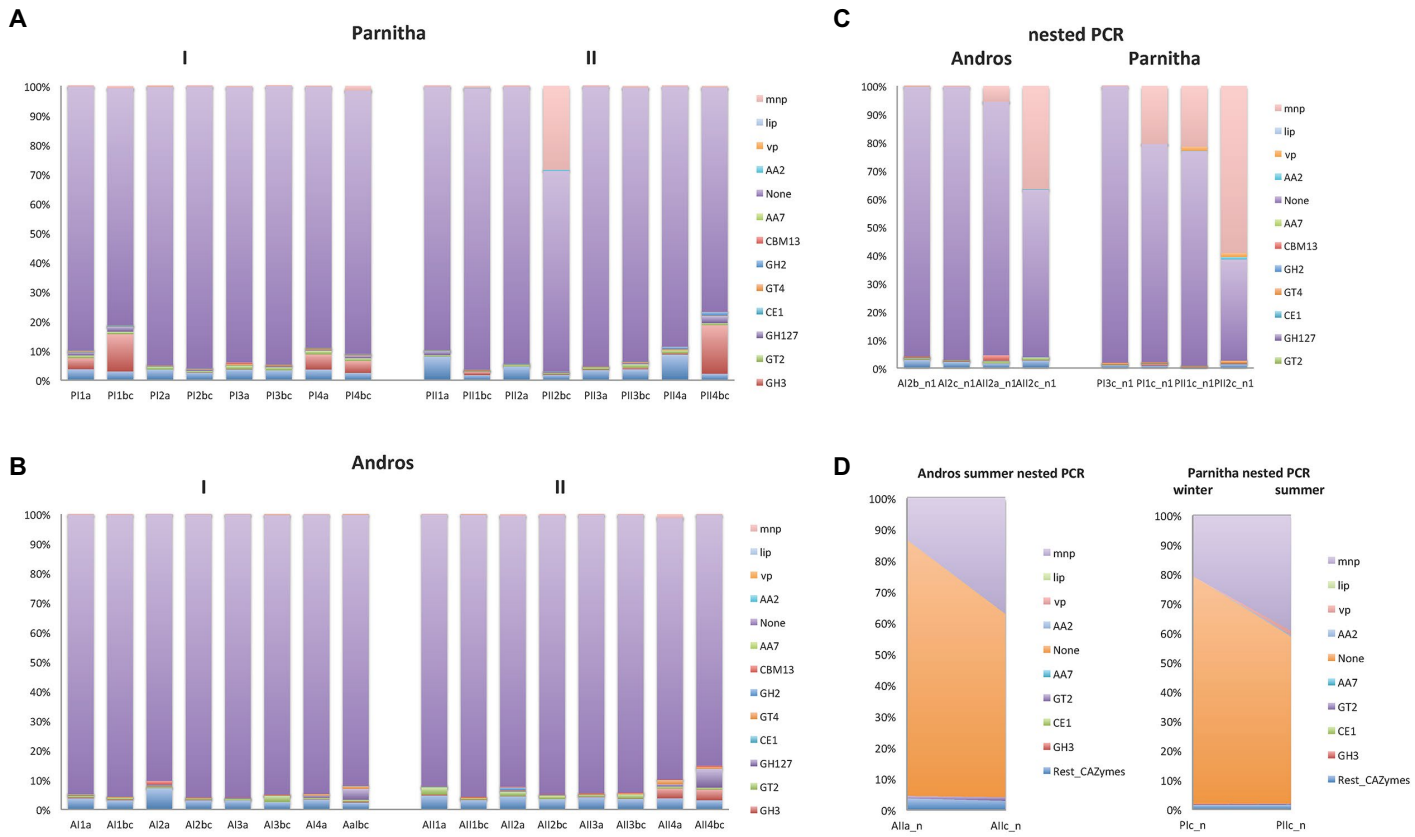
The distribution of AA2 peroxidases and the most abundant CAZymes in the sampling plots (1,2,3,4), soil depths (a,b,c) and seasons (I = winter, II = summer) of **(A)** Parnitha and **(B)** Andros forests. The distribution of the same enzyme sequences obtained by nested PCR reactions are also shown for comparison **(C)**, while the significant increase of AA2 sequences with depth, in both forests, is shown in (**D**; examples: at summer, with depth, Andros; winter-summer, deeper samples, Parnitha),

By using the automated carbohydrate-active enzyme annotation database dbCAN2, 1,634,172 reads were assigned tο 44,119 CAZymes OTUs, from which 1,424,474 reads and 15,287 OTUs could be definitely assigned to lignin degrading and auxiliary ΑΑ2 peroxidase sequences. From the overall distribution of the CAZymes OTUs (Table 1), it is apparent that the AA2 sequences are almost six times as many in Parnitha than in Andros, and that the majority of these peroxidases are MnPs, with VPs and LiPs detected in very low relative abundance (~2%). More importantly, a marked increase of AA2 sequences is observed in the topsoil samples (“b,c”) of both forests, particularly when nested PCR is used (Figure 4). Taking into consideration that the ΑΑ2 sequences in the *Basidiomycota* mock cοmmunity control (ΒasK) were 26.81% and in the nested PCR samples from Parnitha 35.35% (Table 1B), our results strongly support that nested PCR is the preferred method, especially under the light that the obtained ΑΑ2 sequences contained conserved sites that allowed discrimination to MnPs, LiPs and VPs.

**Table 1:** (A) The distribution of the 44,119 CAZymes OTUs (%) in different enzyme groups, per forest (A = Andros, P = Parnitha), season (AI, PI = winter; AII, PII = summer) and soil layers (a = surface, b,c = 5–10 cm), and (B) distribution of CAZymes reads and AA2 enzymes in the two forests during winter (I) and summer (II) periods, irrespectively of sampling plot. It is pointed out that results from nested PCR were obtained only from half of the samples (see Supplementary Table S6). The relevant sequences obtained from the DNA pool of 10 species of basidiomycetes with known genomes (BasK) is also shown for comparison purposes.

**A**								
OTUs ID	AIa	AIbc	AIIa	AIIbc	PIa	PIbc	PIIa	PIIbc
MnP	1.58	2.50	21.9	57.82	1.08	59.53	2.02	96.02
VP	0.02	0.43	0.02	0.04	0.01	0.00	0.08	0.25
LiP	0.07	0.08	0.01	0.01	9.44	0.03	0.02	0.95
Rest AA2	0.21	0.24	0.56	0.39	0.15	0.07	0.13	0.88
	**1.88**	**3.25**	**22.49**	**58.26**	**10.68**	**59.63**	**2.25**	**98.10**
AA0	0.41	0.46	0.76	0.06	3.08	0.19	0.22	0.01
AA3	1.01	0.38	0.41	0.05	0.17	0.05	0.26	0.02
AA7	0.01	0.03	0.00	0.01	0.01	0.02	0.01	0.00
GH0	2.11	2.18	3.04	0.88	0.85	0.30	2.43	0.01
GH0-GH94-GH84	0.67	0.88	0.64	0.31	7.83	0.88	0.42	0.04
GH2	0.74	0.79	0.74	0.28	0.87	0.39	0.81	0.10
GH3	0.74	2.51	4.85	3.55	18.50	11.41	1.86	0.66
GH127	2.22	7.67	1.12	5.64	1.42	2.82	4.33	0.12
CE0	0.31	0.18	0.23	0.07	3.51	0.05	0.64	0.00
CE1	0.13	0.32	0.27	0.04	0.09	0.06	0.14	0.00
CE11	0.95	0.93	0.31	0.16	0.19	0.12	0.46	0.00
GT1	1.29	1.66	1.19	0.47	0.29	0.21	1.72	0.01
GT2	20.57	11.21	9.65	5.52	4.41	3.40	9.02	0.11
GT4	5.96	5.84	3.86	1.85	2.21	2.65	3.72	0.13
GT51	2.81	2.18	0.66	2.42	0.59	0.41	1.09	0.01
CBM2	4.68	1.58	8.06	0.39	0.68	0.23	0.60	0.01
CBM13	1.52	1.39	0.69	0.57	2.24	0.96	4.32	0.03
CBM50	1.84	1.92	1.56	0.89	6.59	0.29	1.66	0.01
CBM32 + GH2	1.06	1.38	1.12	0.19	9.54	0.24	1.99	0.03
Rest CAZymes	49.11	53.25	38.31	18.38	26.26	15.69	62.05	0.60
**B**								
	Parnitha			Andros			BasK	
	I	II	Nested	I	II	Nested		
Rest CAZymes	27,127	33,281	13,408	18,403	21,372	11,410	9,122	
Rest AA2	39	272	1,172	39	60	188	1,380	
MnPs	1,099	20,400	126,185	342	736	20,057	76,136	
VPs	2	17	3,250	31	13	23	2,273	
LiPs	12	8	14	13	6	4	8,138	

The increase of AA2 peroxidase sequences observed in Parnitha topsoil samples is considerable since they reach abundance values as high as 98.10% (P2bc) compared with the 2.25% for the surface samples in summer, while in winter the respective results were 59.65 and 10.68%. Similar, albeit less marked, was the increase of AA2 peroxidases in Andros in the winter (1.88% for the surface and 3.35% for the topsoil samples) and summer seasons (22.49% for the surface and 58.26% for the topsoil samples; Table 1). Notably, two Parnitha plots in summer (P1bc and P2bc), which were particularly rich in members of *Agaricales*, *Russulales*, and *Hysterangiales* according to the ITS data presented above, provided most of the AA2 and MnPs sequences in this forest. Accordingly, the richest – but at much lower abundance –plots in Andros were A2 and A4, independently of season and soil layer. Among the other CAZymes that could be safely assigned to a particular class, GT2, GT4 and GH3 classes were of particular interest since they were the most prevalent at all plots, soil layers and seasons for both forests, whereas GH127, GH2, CBM50, CBM2, CBM13 and CE1 classes (in this particular order) were found in lower numbers but in all plots and seasons for both forests. The fact that more than half of the reads and two thirds of the OTUs obtained with the peroxF/R primers were not directly associated with lignocellulose degradation (Figure 4; Supplementary Table S6) indicates that the primers used were not restricted to the amplification of AA2 peroxidases and CAZymes only, but they could also amplify other enzymes putatively involved in degradation process, such as those listed in Supplementary Table S7 (only a small sample of the most abundant sequences is presented).

To detect known and novel AA2 peroxidase sequences from the OTUs corresponding to *Basidiomycota* present in the two forests (ITS analyses), OTUs were clustered using a 97% “sequence identity criterion,” and were identified by BLASTn and BLASTx tools against the custom database we had constructed from sequences of complete basidiomycetes genomes deriving from NCBI, JGI, single entries and population studies. After placement to a particular group (MnPs, VPs, LiPs), multiple alignment followed and the sequences were phylogenetically placed in respective orders, genera or species. Not surprisingly, as the relative abundance of *Polyporales* found in the two forests was very low, very few LiPs were detected, which derived from two species only, i.e., *Phanerochaete chrysosporium* and *Bjerkandera adusta* (distributed to 17 different genes). On the contrary, the 164 VP-like OTUs derived from several genera like *Bjerkandera*, *Cerrena*, *Hexagonia*, *Fomitiporia, Grifola, Ganoderma, Pleurotus* and three unassigned/uncultured taxa (distributed to 23 different genes; Supplementary Figure S6). In the case of the thousands of MnPs OTUs amplified, approximately half belong to a *Hysterangiales* taxon, and the rest to various genera/orders or unassigned/uncultured fungi, e.g., *Agaricales* (related to *Hypsizygus, Inonotus, Pyrrhoderma, Pleurotus, Volvariella, Coprinellus, Agrocybe, Mycena, Conocybe, Hypholoma, Cyathus, Pholiota,* and *Cortinarius*); *Phallomycetidae: Gomphales* (*Gautieria, Gomphus* and *Ramaria*); *Geastrales* (*Geastrum*), and *Sclerogaster*; *Russulales* (related to *Heterobasidion*, *Peniophora, Russula, Lactarius*, and *Stereum*), *Hymenochaetales* (*Trichaptum*), etc. (examples appear in Supplementary Figure S7). It is noticed that the MnPs OTUs assigned to particular genera are in full accordance with the ITS abundance detected in forests plots, e.g., *Russula* in A2, P1, P2; an *Agaricales* taxon in A2, where *Inocybe* and *Tricholoma* are abundant; an *Agaricales* taxon in P3, where *Hebeloma* is abundant; *Cortinarius* in A2 and P3; *Mycena* sp. in A2, P2; *Geastrum* in P4; a *Hysterangiales* taxon in P2; a *Polyporales* taxon related to the genus *Trametes*; *Dichomitus* in P2; an uncultured fungus in A2, etc. As shown in Supplementary Figures S6, S7, several novel AA2 peroxidases were detected and it remains to be examined if they have higher activities for the biodegradation of wood-litter or humus-rich substrates.

## Discussion

Soils are amongst the most diverse terrestrial ecosystems, densely populated by microbial communities that contribute to pivotal life functions like nitrogen and carbon cycling. Microorganisms in forest ecosystems show great dependance on several parameters like soil properties, type of vegetation, plant litter/residues, environmental conditions, geographic location, etc., all of which seriously affect the abundance and composition of fungal and bacterial communities (Baldrian et al., 2012; Stursová et al., 2012; Urbanová et al., 2015; Fierer, 2017; Ρrescott and Vesterdal, 2021). Although several attempts have been made to shed light onto the phylogeny, diversity, structure and function of forest soil microbiota (Fierer et al., 2012; Stursová et al., 2012; Ding et al., 2015; Urbanová et al., 2015; Baldrian, 2017; Wilhelm et al., 2019), pertinent knowledge still remains fragmented and limited due to the complexity and great variability encountered in such habitats. Moreover, most of relevant research is based on analyses of surface samples, and focuses on either bacteria or fungi, but rarely on both (Johnston et al., 2016; Baldrian, 2017). Knowing that these hyper-diverse groups of microorganisms strongly depend on the substrate type and nutrients availability, we were expecting that factors as the substrate, the vegetation and the local environmental conditions will influence their distribution. Therefore, the objective of this study was to (a) examine the abundance, composition and diversity of microorganisms in different soil layers of two – relatively undisturbed in terms of anthropogenic interference or from recent fires – forests dominated by either *A. cephalonica* or *Q. ilex* and located in continental or insular Greece, respectively, during two seasons (winter, summer), and (b) to record putative lignocelluloses degradation functions/activities with the use of specific primers.

Microbial communities in Mediterranean forests are often exposed to harsh conditions such as high temperatures, high doses of UV-light, low soil moisture content and nutrient availability, forcing them to adopt different strategies for survival. Surprisingly enough, information on the microbiome in such habitats is very scarce. On the contrary, numerous studies in other geographic locations around the globe have convincingly demonstrated the serious impact of all the above factors in shaping the respective microbial communities (O’Brien et al., 2005; Malik et al., 2017; Tian et al., 2017; Delgado-Baquerizo et al., 2018). In our work, bacterial OTUs were twice as many in the oak forest of Andros island than in the mainland fir forest of Mt. Parnitha, while both forests shared the same core bacteria phyla, that accounted for more than 85% of the respective total diversity (Supplementary Figures S3A,B). However, important differences were recorded regarding the relative abundance of each phylum in each forest, which were more pronounced when orders richness and the diversity of genera were examined (Supplementary Table S4; Supplementary Figure S4), and probably reflect functional adaptations in the two ecosystems. In general agreement with the few existing studies on oak and pine forests in the Mediterranean area, our findings show that the same bacteria phyla were the predominant in forest soils in all localities examined, with *Proteobacteria*, *Actinobacteria* and *Acidobacteria* being always the most abundant, followed by *Bacteroidetes*, *Verrucomicrobia* and *Planctomycetes* (Lladó et al., 2017; Moroenyane et al., 2018; Reis et al., 2019; Lasa et al., 2022). Ιt could be therefore tempting to attribute the observed similarities of bacteria phyla abundance to the generally similar climatic conditions in Mediterranean forests and the resilience that soil microbial communities have shown under such conditions (Bérard et al., 2015; Bañeras et al., 2022). Since these findings largely differ from results obtained from similar habitats located at distant regions in N. America (Eichorst and Kuske, 2012), China (Ding et al., 2015) or N. Europe (Kielak et al., 2016; Herzog et al., 2019), they can be considered as indicative of a particular type of bacterial phylogenetic clustering in Mediterranean forests. However, careful comparison of our results with those obtained from various other Mediterranean ecosystems, apart from the similarities they show to bacteria communities in Spanish forests (Goberna et al., 2007; Siles and Margesin, 2016), they demonstrate marked differences in richness of core phyla in Israeli and Portuguese oak forests with *Cloroflexi* almost as abundant as the core phyla (Moroenyane et al., 2018; Reis et al., 2019), notably abundant *Firmicutes* and *Patescibacteria* in Italian forests (Bevivino et al., 2014; Castronovo et al., 2021), and abundant *Firmicutes* and *Gemmatimonadetes* in Israel (Moroenyane et al., 2018). Therefore, all the above indicate a mixed profile of phylogenetically clustered core phyla and over-dispersed less abundant phyla for the Mediterranean soil bacterial communities. This is particularly clear when bacteria are examined at the genus level, since a few predominant genera of the core bacteria accounted for more than 85% of the total bacterial OTUs, while several other genera make sporadic appearances at particular sites or seasons, depending on the great variation that soil microenvironments demonstrate even at very small distances from each other (Supplementary Table S4; Supplementary Figure S4). Interestingly enough, the presence of *Archaea* in both forests was almost insignificant, in line with the findings of a study in Spain conifer forests (Lasa et al., 2022). Similarly, the unknown/uncultured/unassigned bacteria were 15–30% at all plots, in accordance with most reports on soil bacteria that failed to match any known taxa found in databases (Land et al., 2015).

The composition and richness of most bacterial phyla in the mainland forest of Partnitha remained insensitive to season at all sampling sites, and only uncultured bacteria and *Alphaproteobacteria* were in higher numbers during the summer, and *Acidobateria* in the winter. In the island forest of Andros, uncultured-and *Alpha-proteobacteria*, as well as *Actinobacteria* and *Chlorοflexi*, were more abundant at all sites in the winter and *Beta-Gamma-Delta-Proteobacteria*, *Bacteroidetes*, *Verrucomicrobia* and *Gemmatimonadetes* in the summer. Soil depth had a more pronounced effect since most bacteria phyla increased their presence in the topsoil samples (“b,c”), irrespective of the forest type, season or plot, displaying a readable genera diversity mainly for *Alphaproteobacteria*, *Acidobateria* and *Actinobacteria* at the periods of higher relative abundance. Our results are in agreement with other pertinent studies on Mediterranean forests (Bevivino et al., 2014; Moroenyane et al., 2018; Reis et al., 2019; Castronovo et al., 2021) and since *Acidobacteria* are good indicators of humid and *Actinobacteria* of dryer forests, they suggest that the observed distributions are most likely due to weather differences between the mainland forest (heavier precipitation) and the island forest (lower precipitation). At the same time the similarities in the occurrence of the core bacteria phyla observed in Mediterranean forests are considered to be driven by their common functional role, i.e., in decomposing the tree litter (plant residues) and bioconverting complex organic compounds to provide plants with valuable substances (Fierer, 2017). Earlier reports considered members of *Actinobacteria*, *Alpha-and Gamma-proteobacteria* as the only bacteria involved in lignin-degradation (Bugg et al., 2011; Saini et al., 2015). However, numerous subsequent studies determined that laccases are the most abundant ligninolytic enzymes in bacteria, and genes coding for these enzymes could be identified in members of *Bacteroidetes*, *Beta-and Delta-proteobacteria*, *Firmicutes* and possibly *Acidobacteria* (Wilhelm et al., 2019; Kalam et al., 2020). In that sense, it is particularly interesting to note the presence of many bacteria genera which possess genes related to (or associated with) lignocellulose degradation functions in both forests and at several plots (Supplementary Table S7), like lignin peroxidases (*lig*D from *Pseudomonas fluorescens*, *lig*I from *Mycobacterium bevis*, *M. neworleance*, *M. kansasi* and *Mycolicibacterium terrea*, *lig*B and *lig*F from *Sphingobium* sp. and *Pantoea apista*), vannilin dehydrogenases and vannilate O-demethylases (from *Bradyrhizobium*, *Sphingobium* and *Pseudomonas* and to a lesser extent from many other genera), a feruloyl-CoA synthase from *Sphingomonas paucimobilis* and a coniferyl aldehyde dehydrogenase of *Pseudomonas aeruginosa* on single plots; altogether indicating that they play an important direct or indirect role in the decomposition of lignin (Granja-Travez et al., 2020).

Extended studies on various soil ecosystems across the globe have established that *Ascomycota* and *Basidiomycota* are invariably the predominant fungal phyla (Baldrian et al., 2012; Tedersoo et al., 2014; Baldrian et al., 2022). As it was the case with bacteria, fungal abundance was strongly affected by soil structure and stratification, vegetation and climate, e.g., *Ascomycota* predominated in soils of several habitats in Italy and France (Orgiazzi et al., 2013), or were found at equal rates with *Basidiomycota* in temperate soils of pine and deciduous forests (O’Brien et al., 2005), while the relative abundance of *Basidiomycota* was higher (53–59% vs. 25–41% for *Ascomycota*) in soils of oak and coniferous forests in various European countries (Baldrian et al., 2012; Voříšková et al., 2014; Kielak et al., 2016; Castaño et al., 2018; Štursová et al., 2020). In our work, and for all soil samples from both forests, fungi quantitatively dominated bacteria, and members of *Basidiomycota* were recorded at higher than in other Mediterranean forests relative abundances (~67%), whereas *Ascomycota* (~25%) were detected at lower relative abundances (especially in the coniferous forest of Parnitha). Season exerted a similar effect on the main orders of *Ascomycota* in the two forests, i.e., *Eurotiales* were found at equal abundances, *Sordariales*, *Pleosporales* and *Diaporthales* were more abundant in winter, and *Helotiales* and *Hypocreales* were more abundant in summer. Since no xylariaceous *Ascomycota* were detected in the soils of both forests, and the rest of *Ascomycota* comprise a small part of soft-rot fungi with limited ability to degrade lignin, their role is considered as complementary to that of *Basidiomycota* that are the main wood-degraders (Liers et al., 2011; Cragg et al., 2015). The uniform presence of the cosmopolitan genus *Penicillium* at all plots, depths and seasons, and to a lesser extent of the genus *Humicola*, a *Helotiales* taxon and an unspecified *Chaetomiaceae* taxon, are in agreement with the results of other studies in Mediterranean forests (Rincón et al., 2015; Adamo et al., 2021; Hagenbo et al., 2021). Moreover, in conjunction with the outcome of a metaproteomic study showing that the secreted enzymes of *Eurotiomycetes*, *Leotiomycetes* and *Sordariomycetes* dominated in a beech and conifer forest (Schneider et al., 2012), it is further supported that these fungi play a key role in the degradation of cellulose and hemicelluloses. However, it must be pointed out that *Trichoderma*, *Cladosporium* and *Alternaria*, which were found in abundance in other Mediterranean forests, were detected only in small numbers in soils of both Greek forests (Supplementary Table S5; Supplementary Figure S5). On the other hand, the exclusive presence of ascomycete genera like the mycorrhizal *Oidiodendron* in Andros and the endophyte *Preussia*, along with several other mycorrhizal generalists like *Humaria*, *Otidea* and *Wilcoxina*, ectomycorrhizae like *Trichophaea*, litter colonizers like *Desmazierella*, and the truffle and truffle-like *Tuber* and *Geopora* (all found at single soil plots in Parnitha), are highly indicative of the habitat-specific differences in respect to fungal communities, and probably reflect their complementarity to metabolic traits of *Basidiomycetes* (Krah et al., 2015). On the contrary, the high abundance of entomopathogens like *Metarhizium* and *Ophiocordyceps* or of the plant pathogen *Coniella* at single plots could be considered as transfers from infected/dead insects or plants, in contrast to *Coniochaeta* species which attack wood and hence contribute to the degradation of lignocellulosics (MycoCosm, mycocosm.jgi.doe.gov).

Six orders of *Basidiomycota* (i.e., *Agaricales*, *Russulales*, *Sebacinales*, *Trechisporales*, *Thelephorales* and *Filobasidiales*) accounted for more than 86% of the diversity in this phylum. Since results from control plots in both forests (open canopy gaps with no pronounced decomposition activities) were very similar with those obtained from all other plots, the possibility of overestimating the abundance of *Basidiomycota* can be excluded. The significant increase of *Basidiomycota* with soil depth in both forests, coupled with the markedly higher abundance of *Ascomycota* in Andros (mainly on surface samples), demonstrates the considerable variations in fungal distribution and composition among soil layers between the fir and oak forests, underlining at the same time the strong influence of soil stratification, vegetation and seasonality. This is further supported by the distinct differences observed regarding the distribution of genera of *Basidiomycota*, which were either quantitative (for the commonly found in both forests and at all plots *Russula*, *Sebacina*, *Cortinarius*, *Inocybe* and *Trechispora*), or qualitative for those found exclusively in all plots of one forest, for one season, e.g., *Mycena* in Parnitha and *Tricholoma* in Andros. Moreover, the strikingly strong dominance of several genera in few plots of either forest, underlined the important role of even small variations in soil constitution and is indicative of the plant-substrate specificity that has been established by fungal communities (Krah et al., 2015). This is rather anticipated since differences in the type of decomposing material shape the microbial communities and establish mutualistic relationships between plants and microorganisms as reported in pertinent studies on Mediterranean conifer and oak forests (Delgado-Baquerizo et al., 2016; Štursová et al., 2020; Adamo et al., 2021; Hagenbo et al., 2021). In this respect, the high abundance of mycorrhizal taxa recorded in both forests of the present work is explained by the mutualistic relationships that have been developed between the Mediterranean trees, regularly suffering from water/heat stress, and their fungal partners, that help to alleviate/mitigate drought repercussions (or to cope with other adverse environmental conditions; Mohan et al., 2014). Thus, the dominance of mycorrhizal generalists like members of *Russula*, *Sebacina*, *Cortinarius*, and *Inocybe* in the soil layers of both forests, highlights the similarities with other Mediterranean conifer and oak forests (Orgiazzi et al., 2013; Pecoraro et al., 2014; Rincón et al., 2015; Castaño et al., 2018; Saitta et al., 2018; Adamo et al., 2021). At the same time the dominance of *Hebeloma*, *Mycena*, *Clavariadelphus*, *Geastrum*, *Pseudotomentella* in the fir forest of Parnitha, and *Tomentella*, *Rhodocollybia* and *Lactarius* in the oak forest of Andros, illustrate the differences of fungal mycorrhizal communities between the two sites. Similarly, the high abundance of the yeast genera *Solicoccozyma* and *Saitozyma* (*Filobasidiales*) in Andros, but not in Parnitha (present only at two plots in very low numbers) is noteworthy, while the widespread distribution of *Mortierella* (*Mucoromycota*) at all plots of both forests (particularly in winter) is probably due to its adaptation to degrade pectin, cellulose and hemicelluloses (Sista Kameshwar and Qin, 2019). Taking into consideration the aforementioned distribution of bacterial communities in the two forests and the AA2 peroxidases sequences recovered, it is likely that interactions among microorganisms may affect the fitness of either fungi or bacteria depending on whether the organisms possess the enzymes required to degrade lignin (mainly white-rot fungi) or hemicelluloses and cellulose (*Basidiomycota*, *Ascomycota*, *Bacteria*). Such effects have been demonstrated by secretome and transcriptomic studies on soil microbiomes (Kellner et al., 2014; Gruninger et al., 2017; Moraes et al., 2018; Peng et al., 2018; Wymelenberg et al., 2020).

We recorded in both forests a sequential succession of *Ascomycota* that colonize wood debris (leaves, twigs etc.) on the soil surface/organic layer by *Agaricomycetes* that are the main decomposers in the deeper topsoil layer. This change in structure and distribution of fungal communities with soil depth and over the course of decomposition is to be expected as the fast-growing ascomycetes and associated mitosporic fungi produce enzymes able to depolymerize cellulose and hemicelluloses, leading to rapid loss of wood rigidity, but can only weakly affect lignin, while *Agaricomycetes* and in particular white-rot fungi are the principal lignin degraders (Schneider et al., 2012; Cragg et al., 2015; Baldrian, 2017). Not surprisingly, in direct correlation to the relative abundance of basidiomycetes in the topsoil samples of both forests (as revealed by ITS2 analyses), the targeted PCR AA2 peroxidase sequences obtained in this study were assigned to these particular fungi (Table 1, Supplementary Figures S6, S7). Similarly, the recovery of GT2, GT4 and GH3 CAZymes classes as the most prevalent in both forests, at all plots, soils layers and seasons, along with GH127, GH2, CBM50, CBM2, CBM13, and CE1 (at lower abundance) is expected since the first group of the aformentioned enzymes are present in all predicted proteomes of 103 representative fungi examined –mainly *Ascomycota* and *Basidiomycota*- and the second group, in most genomes (70–103) of those same fungi, all in multiple gene copies (Zhao et al., 2013). The AA2 peroxidases sequences from the topsoil layers of Parnitha soils were recovered in strikingly high numbers, and corresponded to 59.62% of the totally obtained OTUs in winter and up to 98.10% (in one plot) in summer. As the number of published fungal genomes increases rapidly, detailed comparative genome analyses of wood-decaying basidiomycetes with emphasis on species of *Agaricomycetes* have shown that these fungi excrete a large range of hydrolytic and oxidative enzymes for the degradation of lignocellulosics. White-rot fungi possess combinations of AA2 genes (MnPs, VPs and LiPs) while brown-rot fungi completely lack these genes (Floudas et al., 2012; Ruiz-Dueñas et al., 2013; Riley et al., 2014; Nagy et al., 2016; Floudas et al., 2020; Miyauchi et al., 2020; Ruiz-Dueñas et al., 2021). Furthermore, when sequences of AA2 genes from the available white-rot fungi genomes are carefully analyzed, it becomes evident that the known lignin-degrading subsets of LiPs and VPs are very limited (and only few of them are functionally characterized), while the majority AA2 shareholders are MnPs, which are abundant in all genomes and present in many isozyme forms. Thus, in view of the rarity of characterized LiP sequences for most members of *Polyporales*, and the low relative abundance of members of this particular order in our samples, the recovery of such sequences was limited to 17 genes, derived only from *Phanerochaete chrysosporium* and *Bjerkandera adjusta* (Supplementary Figure S6A). Similarly, the 164 VP-like sequences that we analyzed corresponded to 23 genes of eight species (seven of the order *Polyporales* and one of the order *Agaricales*) along with three sequences that contained the characteristic residues for VPs, but showed lower identity levels in comparison with known genome sequences (denovo134619, placed as *Agaricomycetes*; denovo181710, placed as uncultured *Trametes* or *Dichotomus*; and denovo233737 with the Trp residue one amino acid downstream of its position on the typical VP sequence, possibly indicating low enzyme activity; Supplementary Figure S6B).

Coniferous and angiosperm wood is composed by the same main constituents, i.e., cellulose, hemicelluloses, and lignin, but they differ significantly not only in the relative quantities of these components, but more importantly in their particular structure. Especially hemicelluloses are highly heterogeneous polysaccharides with complex molecular structures, consisting of neutral sugars with a β-1,4 backbone (glucose, xylose, mannose), decorated with an array of other sugars (arabinose, galactose) and uronic acid substitutions, and can be chemically modified by acetylation (Scheller and Ulvskov, 2010). In oaks, hemicelluloses are rich in xylan and contain small amounts of glucomannan, while in conifers they contain small amounts of xylan and acetylated galactoglucomannans (Busse-Wicher et al., 2016). The adjustment of the type and composition of hemicelluloses in the course of evolution of these plants is a way to control the mechanical properties of secondary cell walls; at the same time it provides the background for the co-evolution and adaptation of wood-decaying fungi to the chemical composition of their growth substrate (Ayuso-Fernández et al., 2019). As a consequence, most species of wood-decaying fungi exhibit a characteristic preference for either conifers or hardwoods (Hibbett and Donoghue, 2001; Krah et al., 2015). Data on the evolution of lignin-degrading enzymes show that MnPs were the first enzymes evolved from a single generic peroxidase-encoding gene, and with subsequent gene duplications, mutations, etc., an array of peroxidase subfamilies appeared, the most recent of which are VPs and LiPs, which could be detected in white-rot *Agaricomycetes* only (Floudas et al., 2012). Thus, it is not surprising that we have recorded a wealth of known and unknown or related to a genus or family fungal MnPs (*denovo* sequences, Supplementary Figure S7). In addition, the outcome of a recent study, based on comparative genomics of *Agaricomycetes*, led to the phylogenetic reconstruction of the origin of class II peroxidases, showing that MnPs exist in at least five orders, i.e., *Agaricales*, *Polyporales*, *Corticiales*, *Hymenochaetales* and *Russulales*, and that the early-diverging lineages of *Cantharelalles*, *Sebacinales* and *Dacryomycetes* all lack AA2s (Nagy et al., 2016). Our results clearly suggest that orders of *Phallomycetidae* should also be added to the aforementioned groups since the MnP sequences we found in most abundance derived from genera related to *Hysterangiales* (a Hysterangiales taxon, from a single site), *Gomphales* (*Gautieria*, *Gomphus*, *Ramaria*), *Geastrales* (*Geastrum*), and the *Phallomycetidae* Insertae Sedis *Sclerogaster* (NCBI Taxonomy). Further, the recovery of MnPs from several different genera of *Russulales*, *Hymenochaetales*, *Agaricales*, and *Polyporales*, irrespective of their abundance in soil samples, strongly indicates that the set of primers used in this work in nested PCR is highly efficient for the recovery of AA2 enzymes from all fungi possessing such genes. Although it was previously thought that *Agaricales* lack LiP with the characteristic amino acid residues of the white-rot *Polyporales*, such molecule was recently detected in *Agrocybe*, albeit of the lower enzymatic activity due to the differentiation of the neighboring residues (Sánchez-Ruiz et al., 2021). This novel finding combined with the numerous peroxidase sequences recorded in this work, show that there is still plenty to discover about Class II peroxidases, and that genomic, metagenomic and transcriptomic approaches will certainly pave the way to uncover novel lignin degrading enzymes in the quest of effective renewable energy solutions.

## Conclusion

The abundance, composition and diversity of bacteria and fungi in different soil layers of two forests dominated by either *Abies cephalonica* or *Quercus ilex*, and located in continental or insular Greece, respectively, during winter and summer, has been established by 16S and ITS high throughput sequencing. Although the same core bacteria phyla, i.e., *Proteobacteria*, *Actinobacteria*, *Acidobacteria*, *Bacteroidetes*, *Verrucomicrobia*, and *Planctomycetes*, were present in both forests, they differed significantly both quantitatively and qualitatively. *Archaea* were detected in very low numbers. Similarly, *Basidiomycota* (~67%) and *Ascomycota* (~25%) were the most abundant fungi with *Agaricales*, *Russulales*, *Sebacinales*, *Trechisporales*, *Thelephorales* (*Basidiomycota*) and *Eurotiales*, *Sordariales*, *Pleosporales*, *Helotiales*, and *Diaporthales* (*Ascomycota*) being the most abundant in the two forests, again showing significant differences in their distribution depending on forest, depth and season. The constructed primers aiming to amplify Class II peroxidases succeeded in the recovery of many gene sequences, which through phylogenetic comparisons were shown to belong to known (LiPs), and to both known and unknown genes of VPs and MnPs. An abundance of *Phallomycetidae* (*Hysterangiales*, *Gomphales*, *Geastrales*) novel gene sequences were discovered along with those from *Polyporales*, *Agaricales*, *Russulales*, and *Hymenochaetales.* Non-specific sequences obtained from bacteria with the same primers indicated that they may play an important direct or indirect role in the decomposition of lignin.

## Data availability statement

The datasets presented in this study can be found in online repositories. The names of the repository/repositories and accession number(s) can be found in the article/Supplementary material.

## Author contributions

MK, KP, GZ, and MT: conceived and designed. MK, IP, VV, GZ, KP, and MT: methodology. MK, EP, and GZ: sample collection. MK and IP: data collection, data analysis. MT and KP: funding resources. MK: writing—original draft preparation. MK, MT, GZ, and KP: writing—review and editing. MT, KP, and GZ: supervision. All authors contributed to the article and approved the submitted version.

## Funding

This research has been co-financed by the European Union (European Social Fund-ESF) and Greece through the Operational Programme “Education and Life Learning” of the National Strategic Reference Framework (NSRF)-Research Funding Programme THALIS-UOA 377062 and NKUA Project 18479, while the Next Generation Sequencer was obtained through the Operational Programme “Competitiveness, Entrepreneurship and Innovation,” under the call “Strengthening Research and Innovation Infrastructures” (Project code: 5002803).

## Conflict of interest

The authors declare that the research was conducted in the absence of any commercial or financial relationships that could be construed as a potential conflict of interest.

## Publisher’s note

All claims expressed in this article are solely those of the authors and do not necessarily represent those of their affiliated organizations, or those of the publisher, the editors and the reviewers. Any product that may be evaluated in this article, or claim that may be made by its manufacturer, is not guaranteed or endorsed by the publisher.

## Footnotes

^1^ http://megan.informatik.uni-tuebingen.de/

^2^ http://www.drive5.com/usearch/

^3^ http://www.cazy.org/

^4^ https:/bcb.unl.edu/dbCAN2/

^5^ https://www.ebi.ac.uk

^6^ https://www.kent.ac.uk

^7^ https://iowin.net/en/paup/

^8^ https://mybiosoftware.com

^9^ https://cme.h-its.org

^10^ http://www.microbesonline.org

^11^ https://bioweb.pasteur.fr
