# Supplementary material for: Evaluation of the lignocellulose degradation potential of Mediterranean forests soil microbial communities through diversity and targeted functional metagenomics
Source: Front Microbiol. 2023 Feb 27;14:1121993. doi: 10.3389/fmicb.2023.1121993 (PMC10008878; doi:10.3389/fmicb.2023.1121993)

Supplementary Material

Article Title

Evaluation of the lignocellulose degradation potential of Mediterranean forests soil microbial communities through diversity and targeted functional metagenomics

Maria Kalntremtziou, Ioannis A. Papaioannou, Vasileios Vangalis, Elias Polemis, Katherine M. Pappas, Georgios I. Zervakis, Milton A. Typas*

Corresponding Author
matypas@biol.uoa.gr

Supplementary Figure S1. Distribution of (**a**) *Abies cephalonica* and (**b**) *Quercus ilex* in Greece (black dots), and positions of the two sampling sites indicated by red dots and arrows; representative photos of the respective sites appear under (**c**) *A. cephalonica* and (**d**) *Q. ilex*.

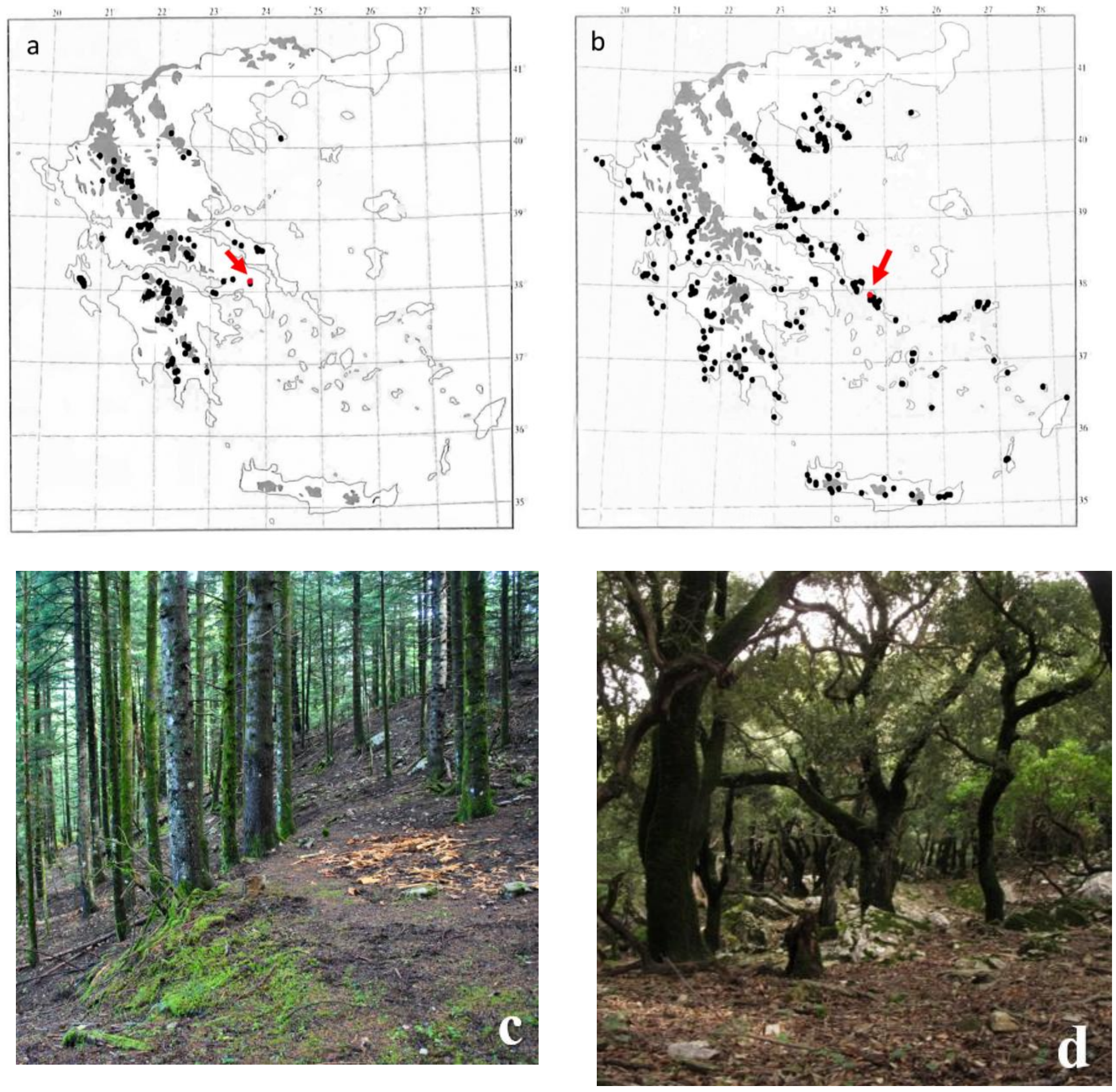

Supplementary Figure S2. (A) ITS2 beta diversity box-plots between samples of Andros, Parnitha and the control (Kalamata, oil-waste). The diversity between samples from Andros and the rest of the samples is higher than between the samples of Parnitha and the control Kalamata. **(B)** Bacterial 16S beta diversity indicates that the high biodiversity of Andros and differentiation to Parnitha and Kalamata, is mainly due to fungi, while the control (Kalamata) holds the highest beta diversity towards bacteria.

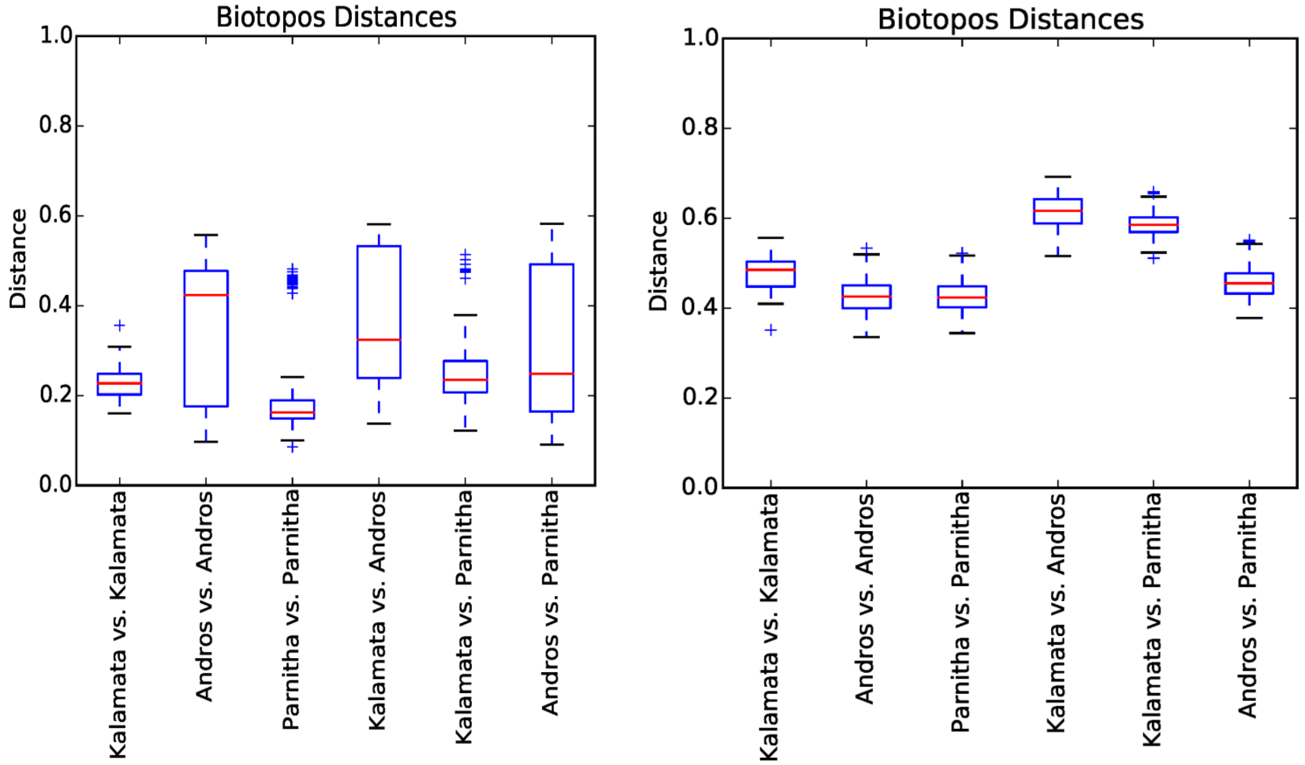

(A) **(B)**

Supplementary Figure S2 (C-D). ITS2 alpha diversity box-plots; **(C1)** Biotopos alpha diversity (Shannon entropy of counts in bits) and the observed OTUs (the number of distinct OTUs) index, within samples of Andros, Parnitha and the control (Kalamata), (**C2**) within winter and summer samples of Andros (AI, AII), Parnitha (PI, PII) and the control (K_) by Shannon and the observed OTUs index, (**C3**) within depths by Shannon and the observed OTUs index showing a decrease of alpha diversity with depth in both forests. Alpha diversity is higher in surface samples of Parnitha and in deeper samples of Andros. Although the p-values of statistical tests do not give much support, the box-plots are in agreement with the general observations. **(D)** Bacterial alpha diversity is in agreement with the fungal; **D1** for Biotopos, **D2** for plots and **D3** for depth with the observed OTUs index.

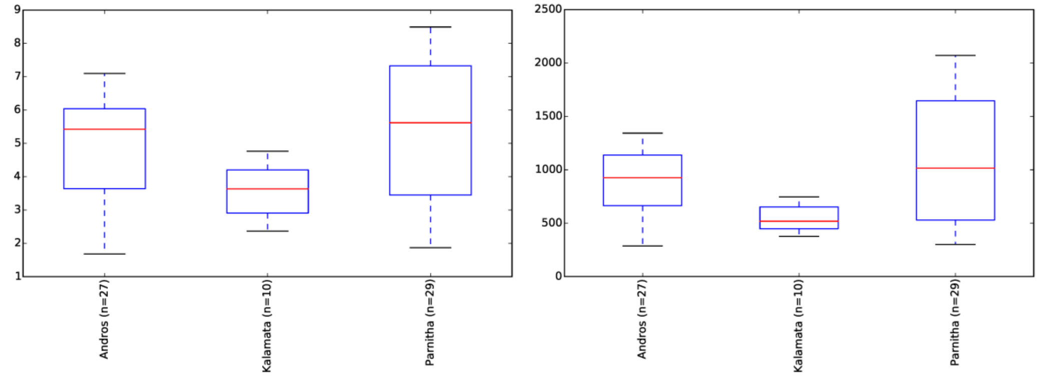

C1

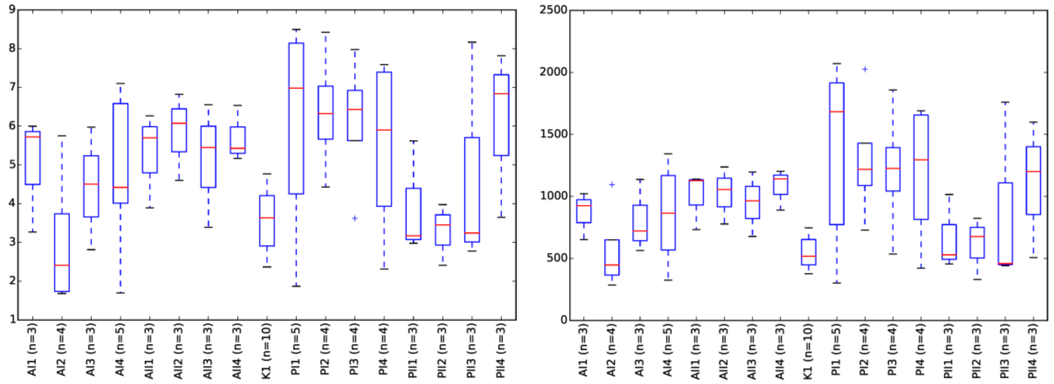

C2

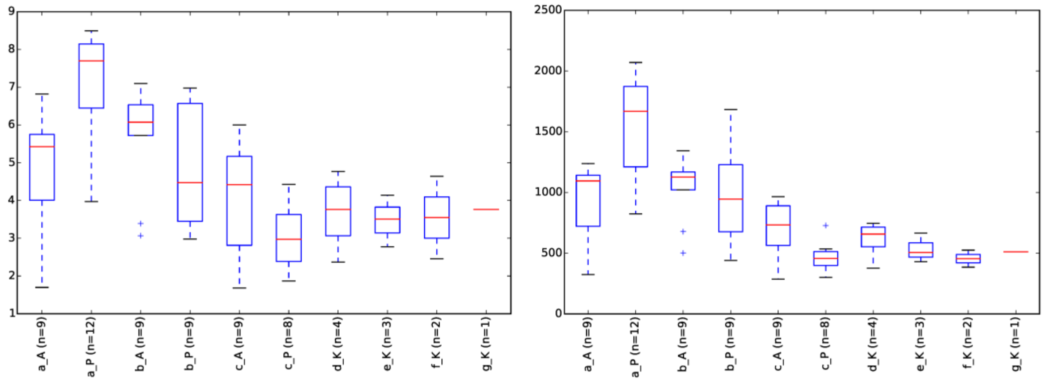

C3

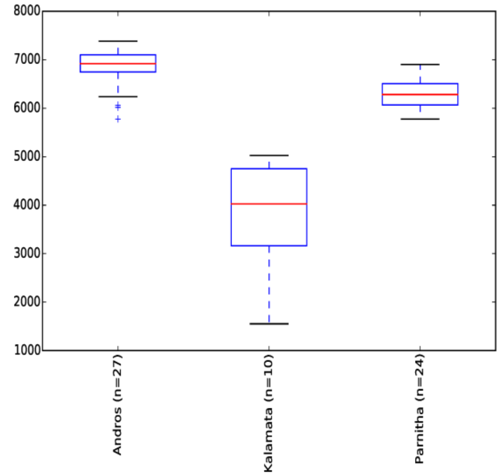
8000
7000
6000
5000
4000
3000
2000
1000
Andros (n=27)
Kalamata (n=10)
Parnitha (n=24)

D1

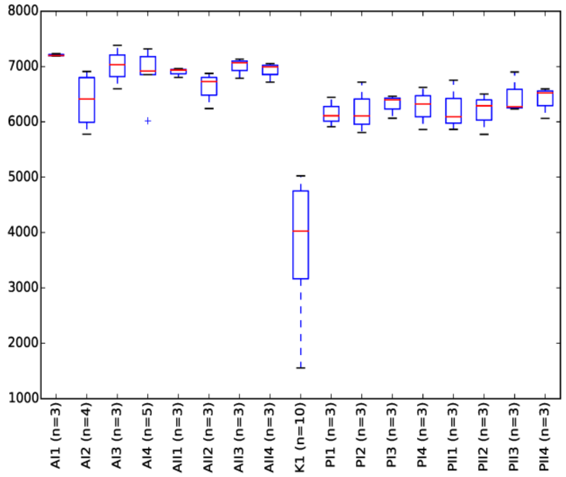
8000
7000
6000
5000
4000
3000
2000
1000
AI1 (n=3)
AI2 (n=4)
AI3 (n=3)
AI4 (n=5)
AII1 (n=3)
AII2 (n=3)
AII3 (n=3)
AII4 (n=3)
K1 (n=10)
PI1 (n=3)
PI2 (n=3)
PI3 (n=3)
PI4 (n=3)
PII1 (n=3)
PII2 (n=3)
PII3 (n=3)
PII4 (n=3)

D2

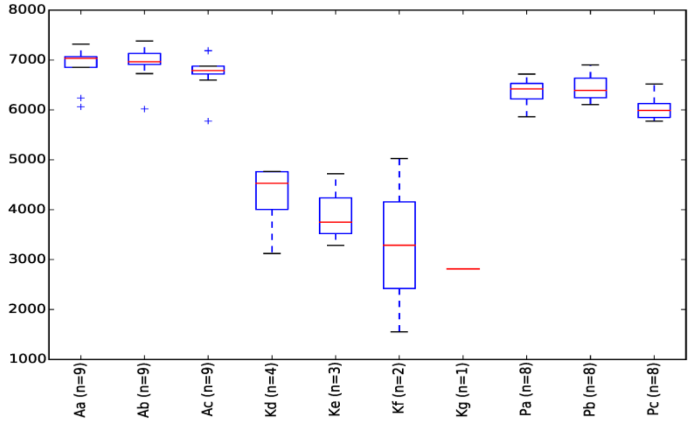
8000
7000
6000
5000
4000
3000
2000
1000
Aa (n=9)
Ab (n=9)
Ac (n=9)
Kd (n=4)
Ke (n=3)
Kf (n=2)
Kg (n=1)
Pa (n=8)
Pb (n=8)
Pc (n=8)

D3

•

Supplementary Figure S3a. The overall distribution of bacteria-archaea (**A**) and fungi phyla (**B**) in the two sampling seasons (I=winter, II=summer) for the Andros and Parnitha forests.

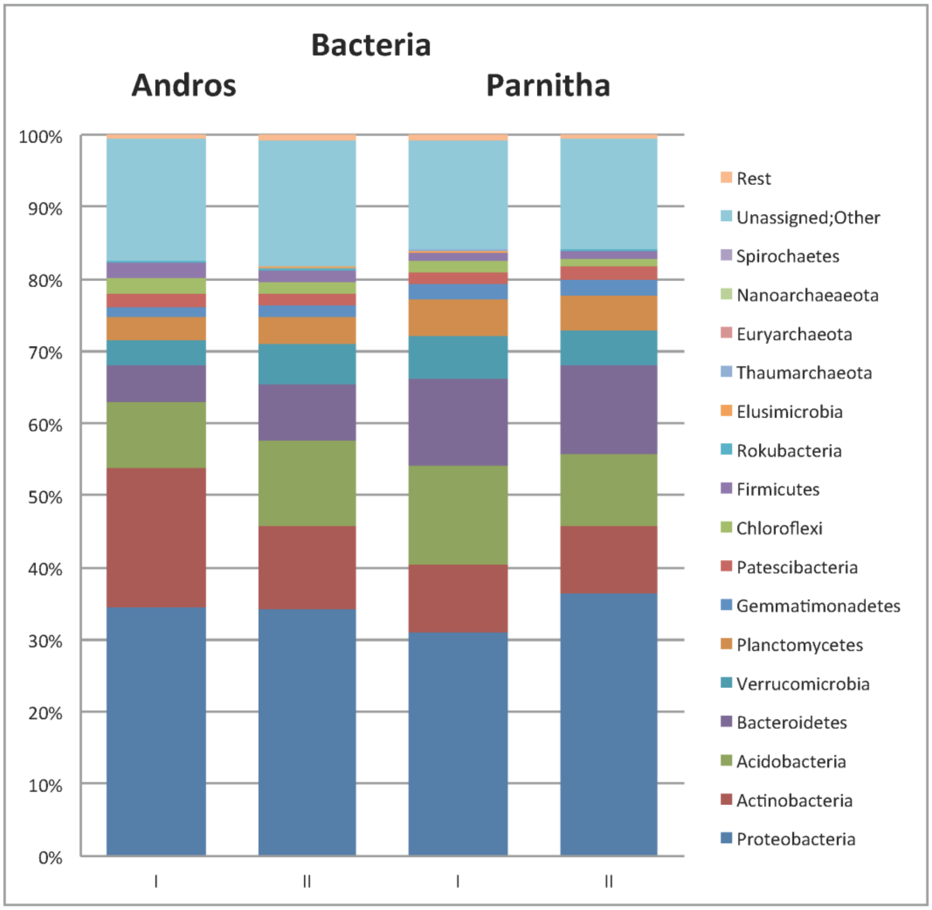

(A)

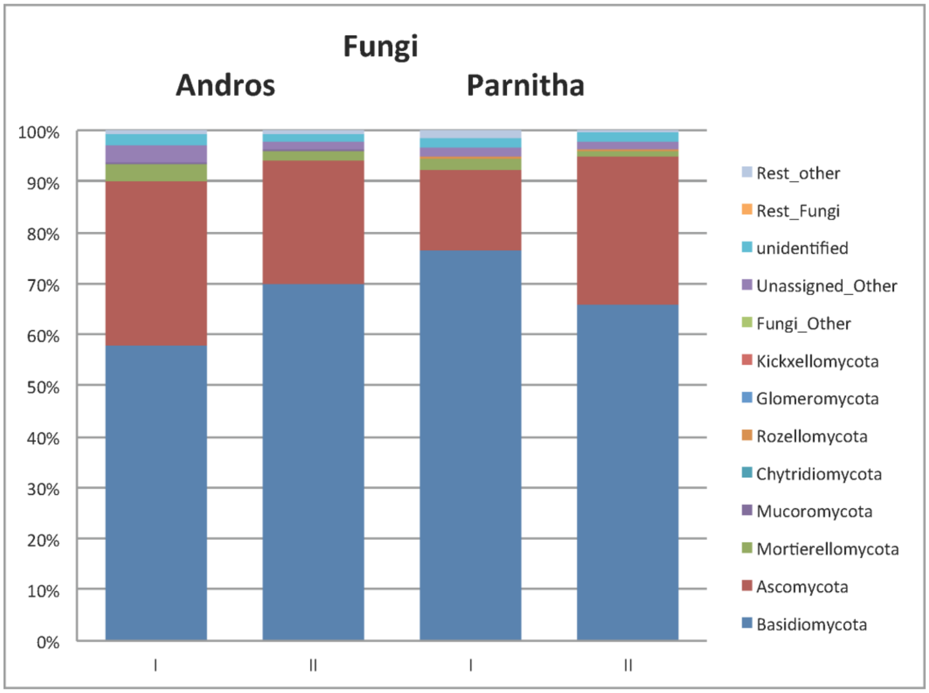

(B)

Supplementary Figure S3b. The distribution of bacteria-archaea phyla in the sampling plots (1,2,3,4), soil depths (a, bc) and seasons (I=winter, II=summer) for (**A**) the Parnitha and (**B**) Andros forests.

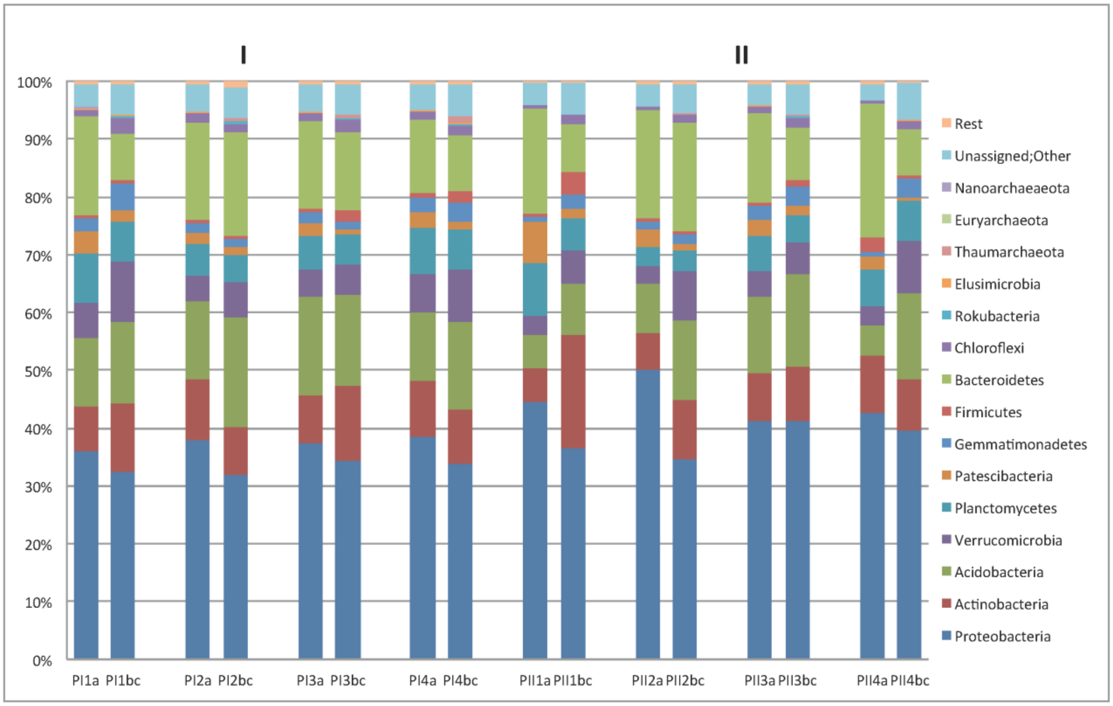

(A)

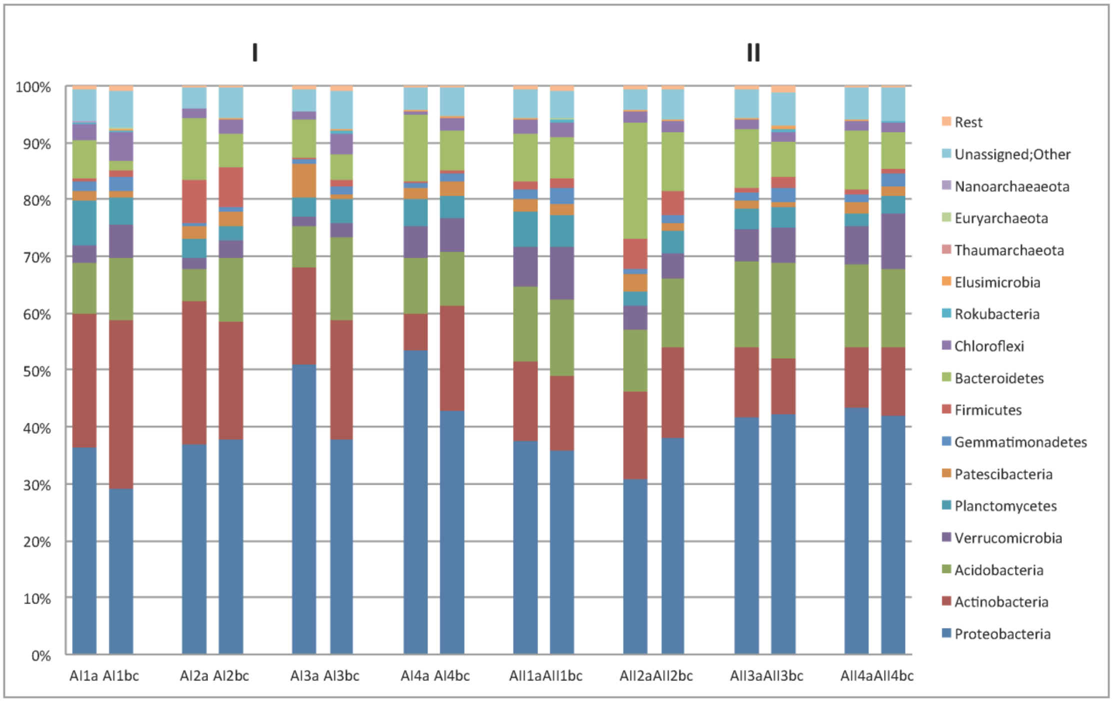

(B)

Supplementary Figure S3c. The distribution of fungi Phyla in the sampling plots (1,2,3,4), soil depths (a, bc) and seasons (I=winter, II=summer) for (**A**) the Parnitha and (**B**) Andros forests.

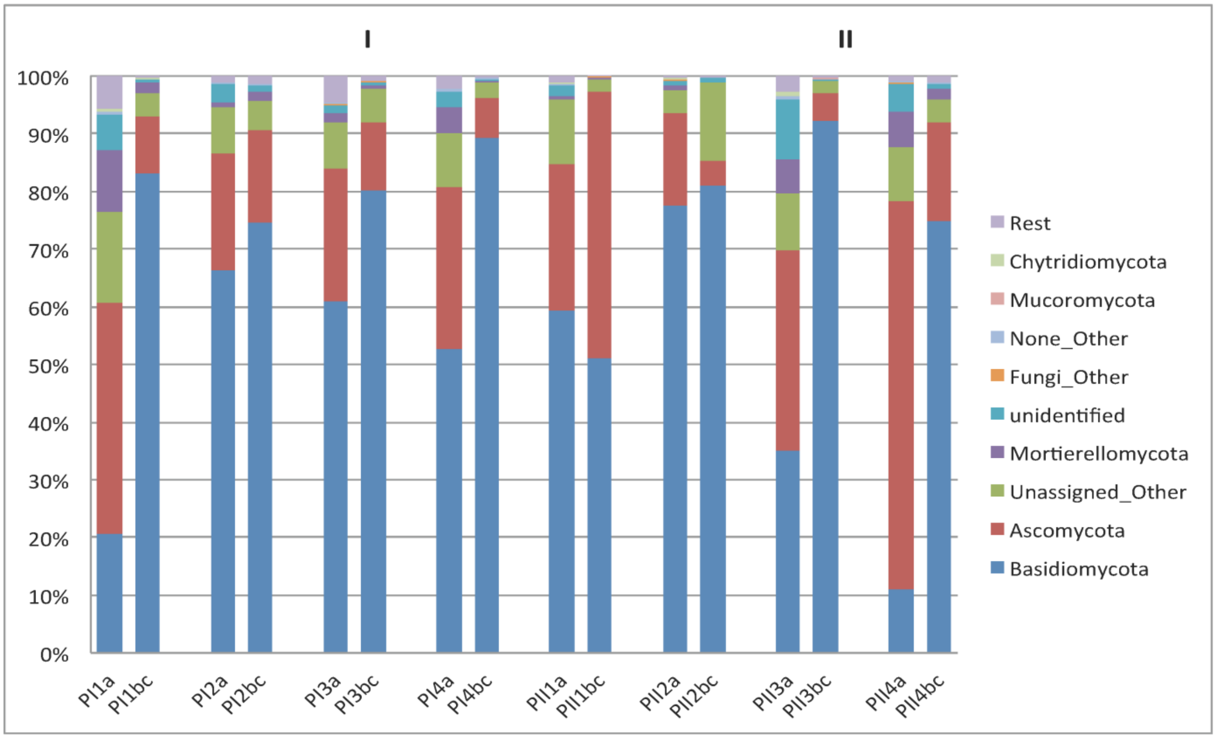

(A)

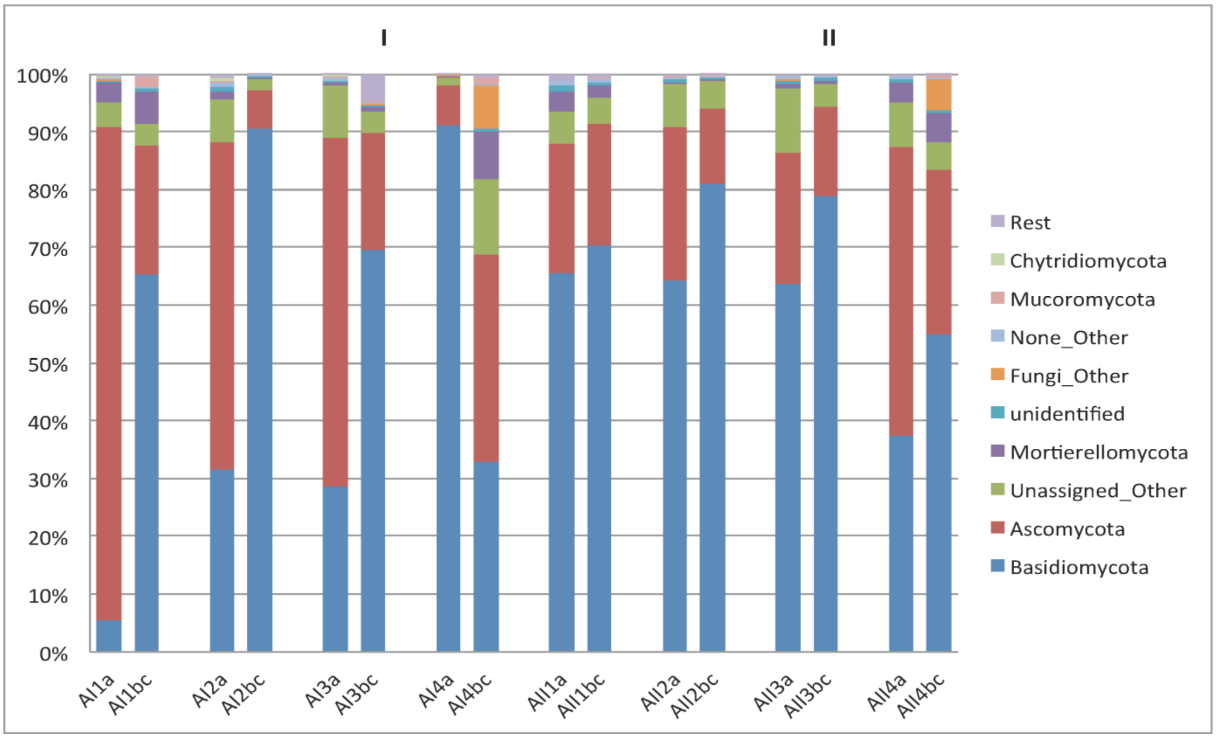

(B)

Supplementary Figure S4. The most abundant genera (>0.5%) of bacteria in the 4 sampling sites (1,2,3,4) and the two season periods (I=winter, II=summer) of the Andros (A) and Parnitha (P) forests.

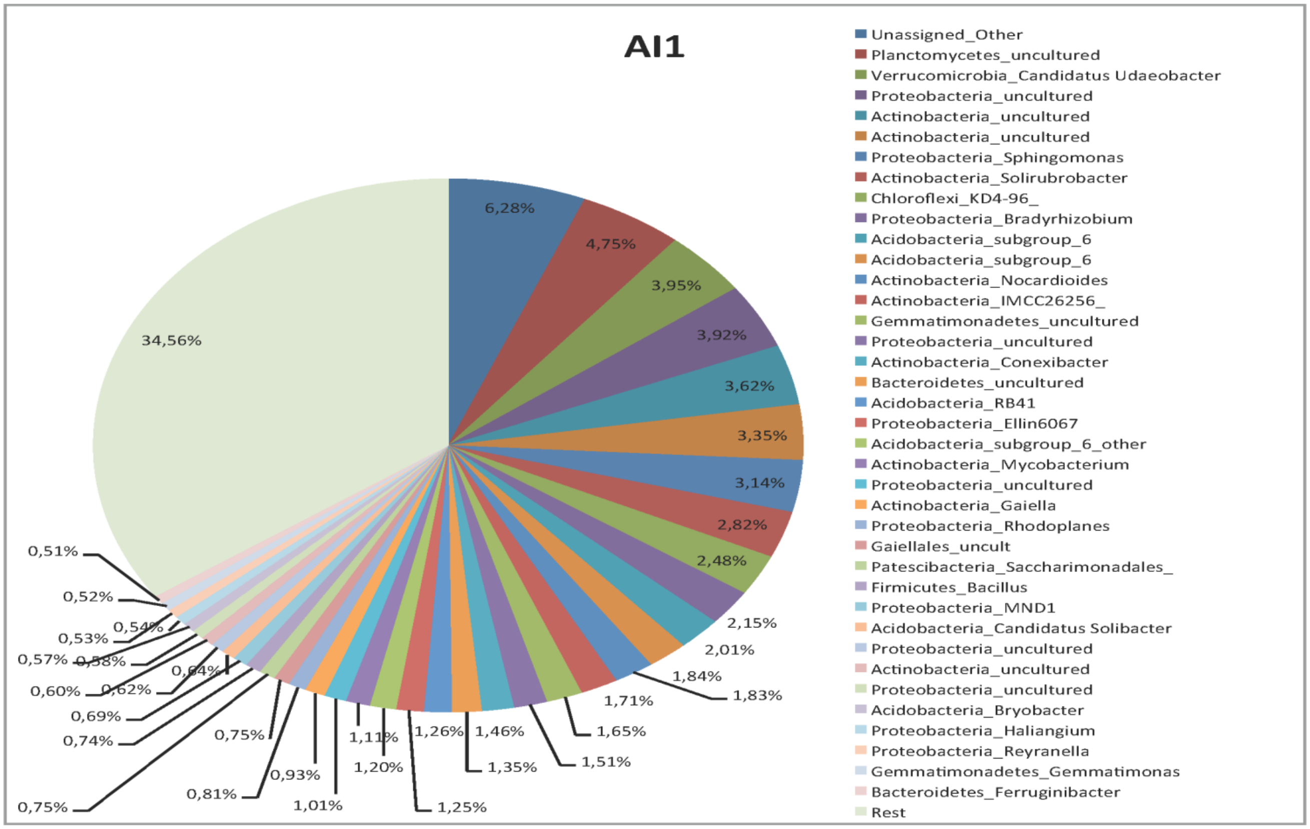

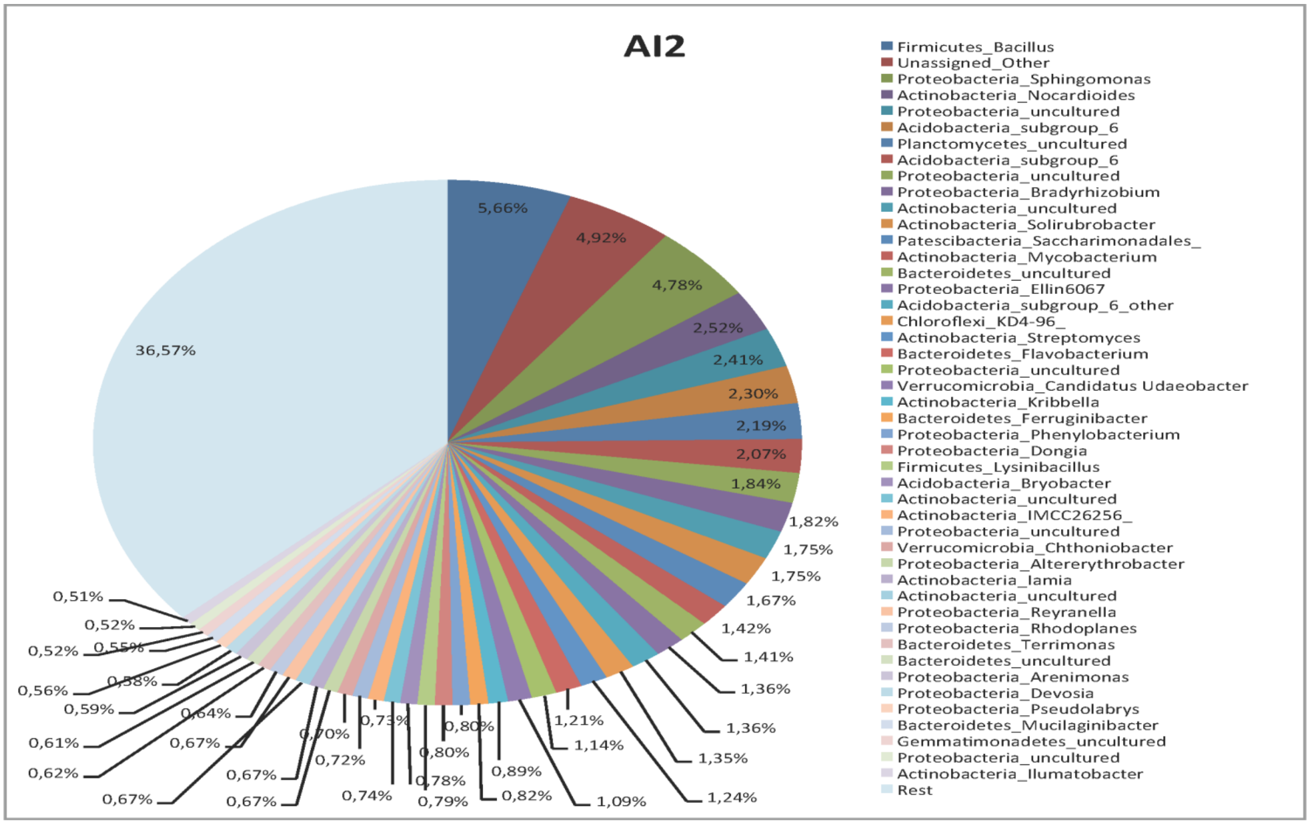

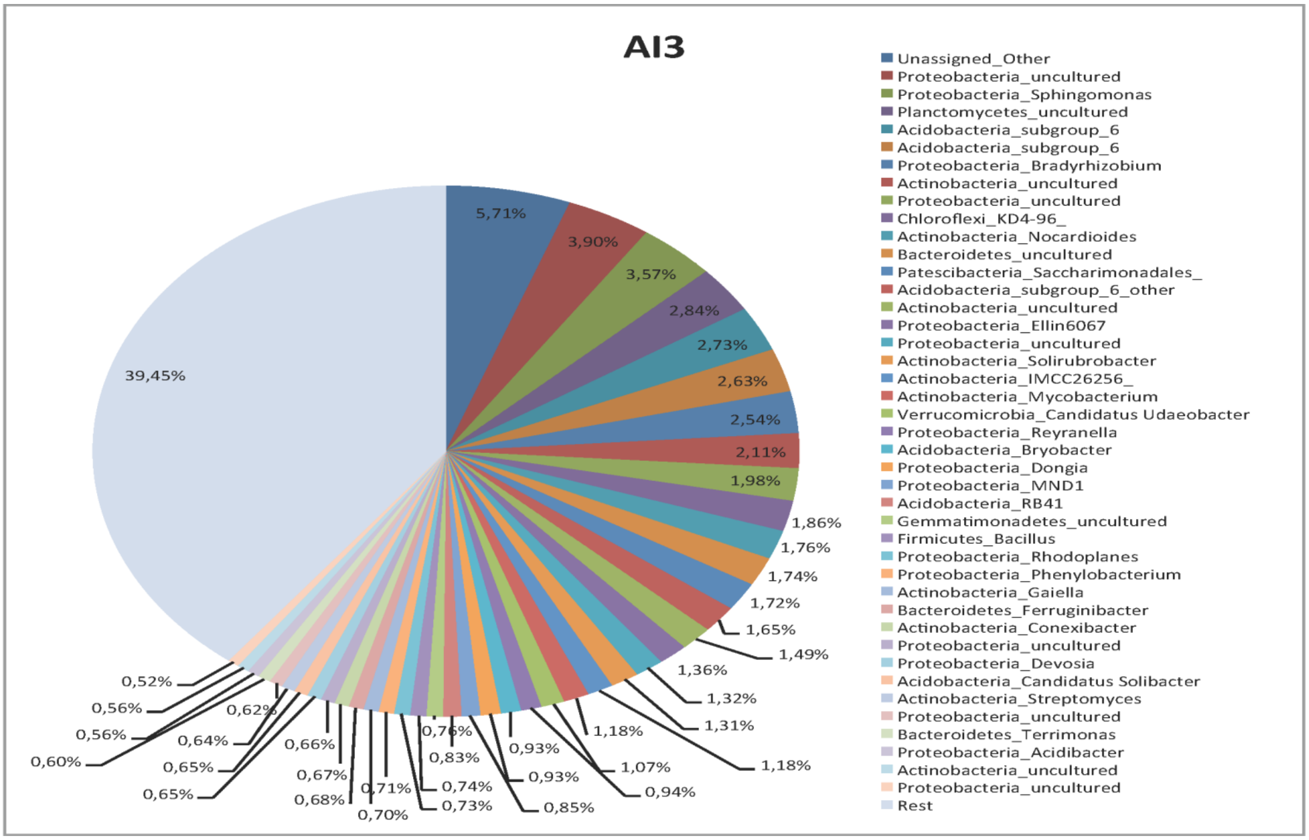
AI3
Unassigned_Other
Proteobacteria_uncultured
Proteobacteria_Sphingomonas
Planctomycetes_uncultured
Acidobacteria_subgroup_6
Acidobacteria_subgroup_6
Proteobacteria_Bradyrhizobium
Actinobacteria_uncultured
Proteobacteria_uncultured
Chloroflexi_KD4-96_
Actinobacteria_Nocardioides
Bacteroidetes_uncultured
Patescibacteria_Saccharimonadales_
Acidobacteria_subgroup_6_other
Actinobacteria_uncultured
Proteobacteria_Ellin6067
Proteobacteria_uncultured
Actinobacteria_Solirubrobacter
Actinobacteria_IMCC26256_
Actinobacteria_Mycobacterium
Verrucomicrobia_Candidatus Udaeobacter
Proteobacteria_Reyranella
Acidobacteria_Bryobacter
Proteobacteria_Dongia
Proteobacteria_MND1
Acidobacteria_RB41
Gemmatimonadetes_uncultured
Firmicutes_Bacillus
Proteobacteria_Rhodoplanes
Proteobacteria_Phenylobacterium
Actinobacteria_Gaiella
Bacteroidetes_Ferruginibacter
Actinobacteria_Conexibacter
Proteobacteria_uncultured
Proteobacteria_Devosia
Acidobacteria_Candidatus Solibacter
Actinobacteria_Streptomyces
Proteobacteria_uncultured
Bacteroidetes_Terrimonas
Proteobacteria_Acidibacter
Actinobacteria_uncultured
Proteobacteria_uncultured
Rest
5,71%
3,90%
3,57%
2,84%
2,73%
2,63%
2,54%
2,11%
1,98%
1,86%
1,76%
1,74%
1,72%
1,65%
1,49%
1,36%
1,32%
1,31%
1,18%
1,18%
1,07%
0,94%
0,93%
0,93%
0,85%
0,83%
0,76%
0,74%
0,73%
0,71%
0,70%
0,68%
0,67%
0,66%
0,65%
0,65%
0,64%
0,62%
0,60%
0,56%
0,56%
0,52%
39,45%

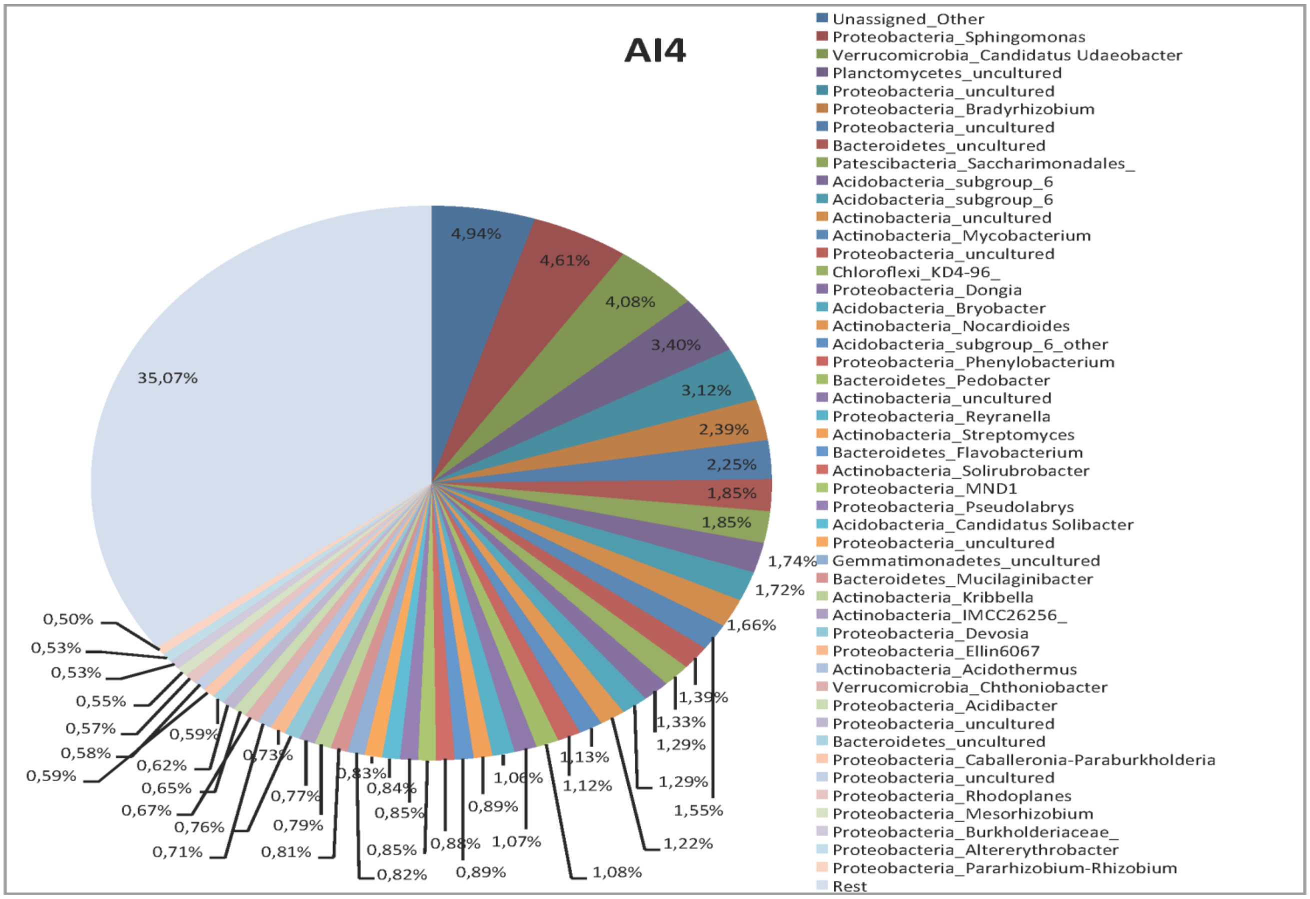
AI4
Unassigned_Other
Proteobacteria_Sphingomonas
Verrucomicrobia_Candidatus Udaeobacter
Planctomycetes_uncultured
Proteobacteria_uncultured
Proteobacteria_Bradyrhizobium
Proteobacteria_uncultured
Bacteroidetes_uncultured
Patescibacteria_Saccharimonadales_
Acidobacteria_subgroup_6
Acidobacteria_subgroup_6
Actinobacteria_uncultured
Actinobacteria_Mycobacterium
Proteobacteria_uncultured
Chloroflexi_KD4-96_
Proteobacteria_Dongia
Acidobacteria_Bryobacter
Actinobacteria_Nocardioides
Acidobacteria_subgroup_6_other
Proteobacteria_Phenylobacterium
Bacteroidetes_Pedobacter
Actinobacteria_uncultured
Proteobacteria_Reyranella
Actinobacteria_Streptomyces
Bacteroidetes_Flavobacterium
Actinobacteria_Solirubrobacter
Proteobacteria_MND1
Proteobacteria_Pseudolabrys
Acidobacteria_Candidatus Solibacter
Proteobacteria_uncultured
Gemmatimonadetes_uncultured
Bacteroidetes_Mucilaginibacter
Actinobacteria_Kribbella
Actinobacteria_IMCC26256_
Proteobacteria_Devosia
Proteobacteria_Ellin6067
Actinobacteria_Acidothermus
Verrucomicrobia_Chthoniobacter
Proteobacteria_Acidibacter
Proteobacteria_uncultured
Bacteroidetes_uncultured
Proteobacteria_Caballeronia-Paraburkholderia
Proteobacteria_uncultured
Proteobacteria_Rhodoplanes
Proteobacteria_Mesorhizobium
Proteobacteria_Burkholderiaceae_
Proteobacteria_Altererythrobacter
Proteobacteria_Pararhizobium-Rhizobium
Rest
4,94%
4,61%
4,08%
3,40%
3,12%
2,39%
2,25%
1,85%
1,85%
1,74%
1,72%
1,66%
1,55%
1,39%
1,33%
1,29%
1,29%
1,22%
1,13%
1,12%
1,08%
1,07%
1,06%
0,89%
0,89%
0,88%
0,85%
0,85%
0,84%
0,83%
0,82%
0,81%
0,79%
0,77%
0,76%
0,73%
0,71%
0,67%
0,65%
0,62%
0,59%
0,59%
0,58%
0,57%
0,55%
0,53%
0,53%
0,50%
35,07%

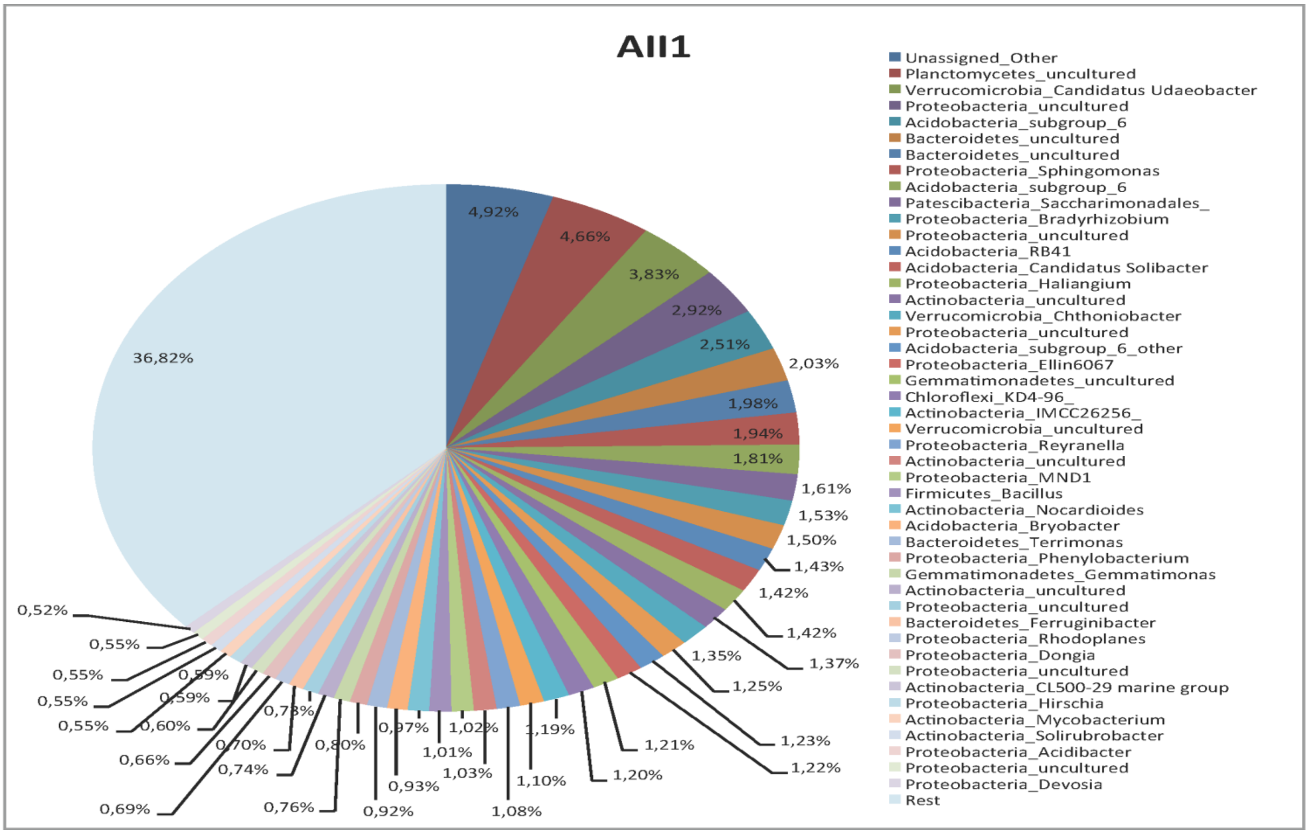
AII1
Unassigned_Other
Planctomycetes_uncultured
Verrucomicrobia_Candidatus Udaeobacter
Proteobacteria_uncultured
Acidobacteria_subgroup_6
Bacteroidetes_uncultured
Bacteroidetes_uncultured
Proteobacteria_Sphingomonas
Acidobacteria_subgroup_6
Patescibacteria_Saccharimonadales_
Proteobacteria_Bradyrhizobium
Proteobacteria_uncultured
Acidobacteria_RB41
Acidobacteria_Candidatus Solibacter
Proteobacteria_Haliangium
Actinobacteria_uncultured
Verrucomicrobia_Chthoniobacter
Proteobacteria_uncultured
Acidobacteria_subgroup_6_other
Proteobacteria_Ellin6067
Gemmatimonadetes_uncultured
Chloroflexi_KD4-96_
Actinobacteria_IMCC26256_
Verrucomicrobia_uncultured
Proteobacteria_Reyranella
Actinobacteria_uncultured
Proteobacteria_MND1
Firmicutes_Bacillus
Actinobacteria_Nocardioides
Acidobacteria_Bryobacter
Bacteroidetes_Terrimonas
Proteobacteria_Phenylobacterium
Gemmatimonadetes_Gemmatimonas
Actinobacteria_uncultured
Proteobacteria_uncultured
Bacteroidetes_Ferruginibacter
Proteobacteria_Rhodoplanes
Proteobacteria_Dongia
Proteobacteria_uncultured
Actinobacteria_CL500-29 marine group
Proteobacteria_Hirschia
Actinobacteria_Mycobacterium
Actinobacteria_Solirubrobacter
Proteobacteria_Acidibacter
Proteobacteria_uncultured
Proteobacteria_Devosia
Rest
4,92%
4,66%
3,83%
2,92%
2,51%
2,03%
1,98%
1,94%
1,81%
1,61%
1,53%
1,50%
1,43%
1,42%
1,42%
1,37%
1,35%
1,25%
1,23%
1,22%
1,21%
1,20%
1,19%
1,10%
1,08%
1,03%
1,02%
1,01%
0,97%
0,93%
0,92%
0,80%
0,76%
0,74%
0,73%
0,70%
0,69%
0,66%
0,60%
0,59%
0,59%
0,55%
0,55%
0,55%
0,55%
0,52%
36,82%

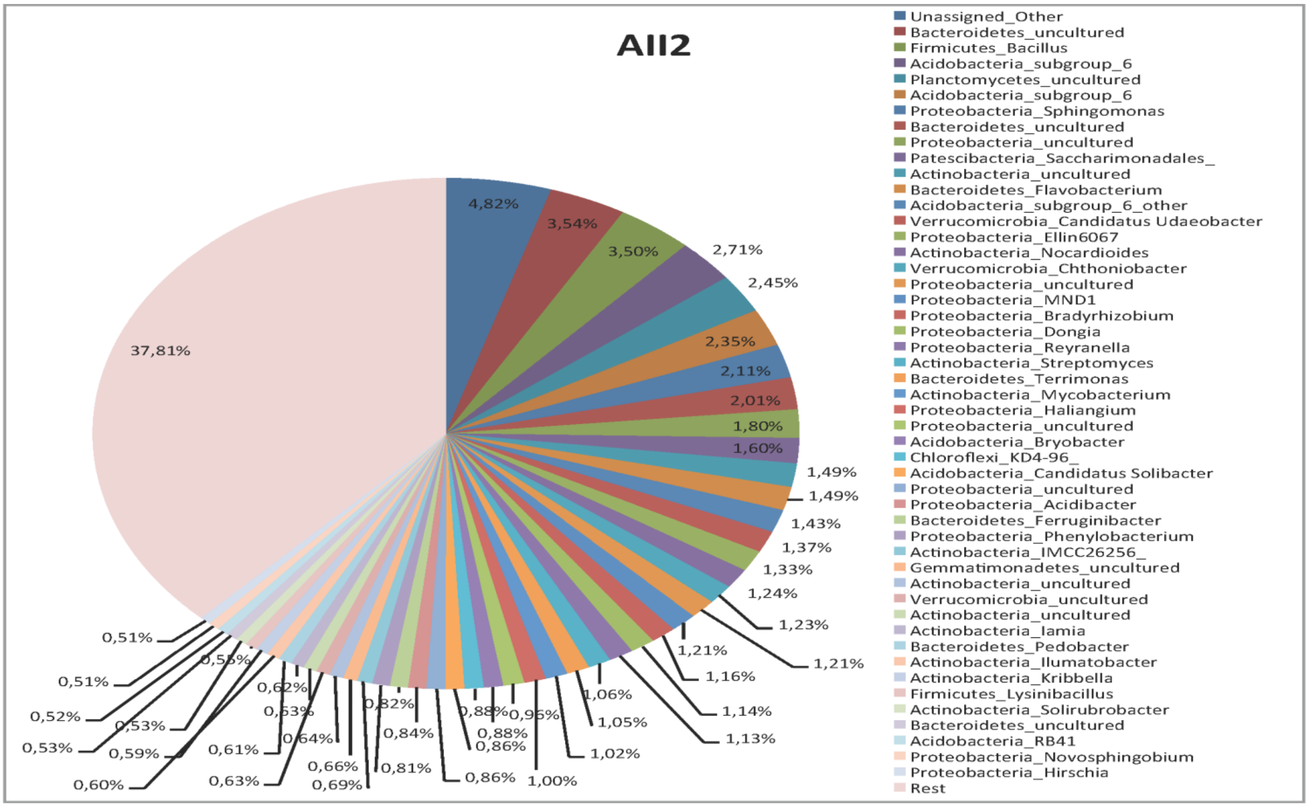
AII2
Unassigned_Other
Bacteroidetes_uncultured
Firmicutes_Bacillus
Acidobacteria_subgroup_6
Planctomycetes_uncultured
Acidobacteria_subgroup_6
Proteobacteria_Sphingomonas
Bacteroidetes_uncultured
Proteobacteria_uncultured
Patescibacteria_Saccharimonadales_
Actinobacteria_uncultured
Bacteroidetes_Flavobacterium
Acidobacteria_subgroup_6_other
Verrucomicrobia_Candidatus Udaeobacter
Proteobacteria_Ellin6067
Actinobacteria_Nocardioides
Verrucomicrobia_Chthoniobacter
Proteobacteria_uncultured
Proteobacteria_MND1
Proteobacteria_Bradyrhizobium
Proteobacteria_Dongia
Proteobacteria_Reyranella
Actinobacteria_Streptomyces
Bacteroidetes_Terrimonas
Actinobacteria_Mycobacterium
Proteobacteria_Haliangium
Proteobacteria_uncultured
Acidobacteria_Bryobacter
Chloroflexi_KD4-96_
Acidobacteria_Candidatus Solibacter
Proteobacteria_uncultured
Proteobacteria_Acidibacter
Bacteroidetes_Ferruginibacter
Proteobacteria_Phenylobacterium
Actinobacteria_IMCC26256_
Gemmatimonadetes_uncultured
Actinobacteria_uncultured
Verrucomicrobia_uncultured
Actinobacteria_uncultured
Actinobacteria_Iamia
Bacteroidetes_Pedobacter
Actinobacteria_Ilumatobacter
Actinobacteria_Kribbella
Firmicutes_Lysinibacillus
Actinobacteria_Solirubrobacter
Bacteroidetes_uncultured
Acidobacteria_RB41
Proteobacteria_Novosphingobium
Proteobacteria_Hirschia
Rest
4,82%
3,54%
3,50%
2,71%
2,45%
2,35%
2,11%
2,01%
1,80%
1,60%
1,49%
1,49%
1,43%
1,37%
1,33%
1,24%
1,23%
1,21%
1,21%
1,16%
1,14%
1,13%
1,06%
1,05%
1,02%
1,00%
0,96%
0,88%
0,88%
0,86%
0,86%
0,84%
0,82%
0,81%
0,69%
0,66%
0,64%
0,63%
0,63%
0,62%
0,61%
0,60%
0,59%
0,55%
0,53%
0,53%
0,52%
0,51%
0,51%
37,81%

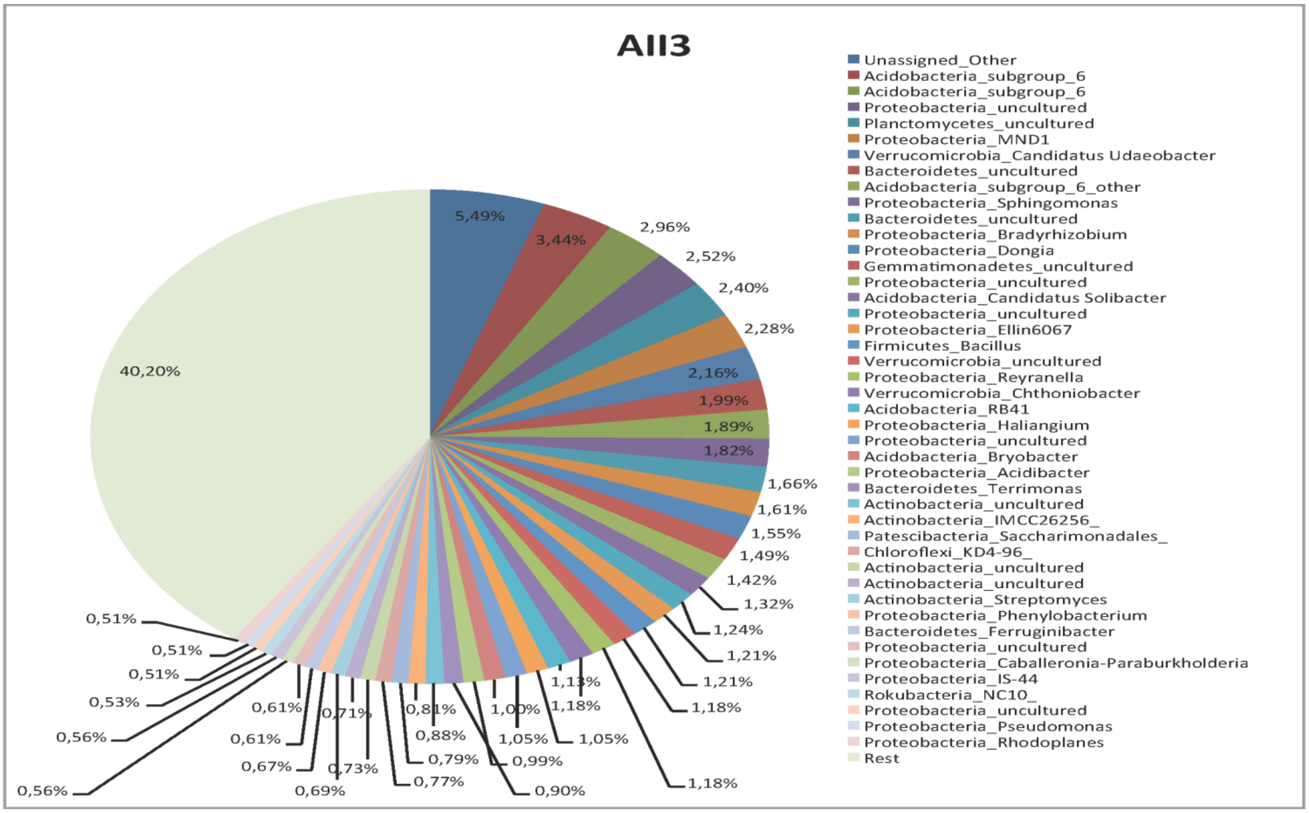
AII3
Unassigned_Other
Acidobacteria_subgroup_6
Acidobacteria_subgroup_6
Proteobacteria_uncultured
Planctomycetes_uncultured
Proteobacteria_MND1
Verrucomicrobia_Candidatus Udaeobacter
Bacteroidetes_uncultured
Acidobacteria_subgroup_6_other
Proteobacteria_Sphingomonas
Bacteroidetes_uncultured
Proteobacteria_Bradyrhizobium
Proteobacteria_Dongia
Gemmatimonadetes_uncultured
Proteobacteria_uncultured
Acidobacteria_Candidatus Solibacter
Proteobacteria_uncultured
Proteobacteria_Ellin6067
Firmicutes_Bacillus
Verrucomicrobia_uncultured
Proteobacteria_Reyranella
Verrucomicrobia_Chthoniobacter
Acidobacteria_RB41
Proteobacteria_Haliangium
Proteobacteria_uncultured
Acidobacteria_Bryobacter
Proteobacteria_Acidibacter
Bacteroidetes_Terrimonas
Actinobacteria_uncultured
Actinobacteria_IMCC26256_
Patescibacteria_Saccharimonadales_
Chloroflexi_KD4-96_
Actinobacteria_uncultured
Actinobacteria_uncultured
Actinobacteria_Streptomyces
Proteobacteria_Phenylobacterium
Bacteroidetes_Ferruginibacter
Proteobacteria_uncultured
Proteobacteria_Caballeronia-Paraburkholderia
Proteobacteria_IS-44
Rokubacteria_NC10_
Proteobacteria_uncultured
Proteobacteria_Pseudomonas
Proteobacteria_Rhodoplanes
Rest
5,49%
3,44%
2,96%
2,52%
2,40%
2,28%
2,16%
1,99%
1,89%
1,82%
1,66%
1,61%
1,55%
1,49%
1,42%
1,32%
1,24%
1,21%
1,21%
1,18%
1,18%
1,13%
1,18%
1,05%
1,05%
1,00%
0,99%
0,90%
0,81%
0,88%
0,79%
0,77%
0,73%
0,71%
0,69%
0,67%
0,61%
0,61%
0,56%
0,56%
0,53%
0,51%
0,51%
0,51%
40,20%

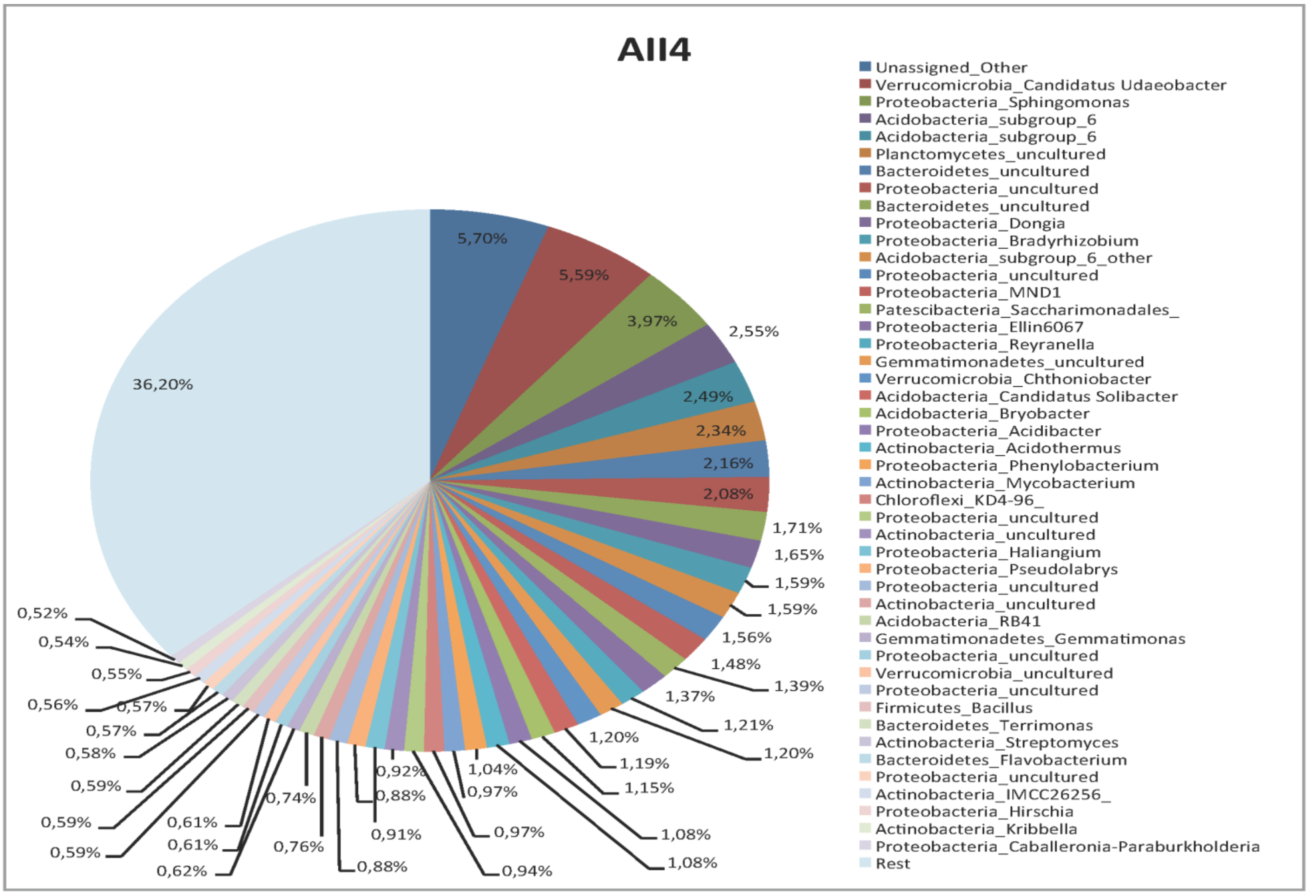
AII4
Unassigned_Other
Verrucomicrobia_Candidatus Udaeobacter
Proteobacteria_Sphingomonas
Acidobacteria_subgroup_6
Acidobacteria_subgroup_6
Planctomycetes_uncultured
Bacteroidetes_uncultured
Proteobacteria_uncultured
Bacteroidetes_uncultured
Proteobacteria_Dongia
Proteobacteria_Bradyrhizobium
Acidobacteria_subgroup_6_other
Proteobacteria_uncultured
Proteobacteria_MND1
Patescibacteria_Saccharimonadales_
Proteobacteria_Ellin6067
Proteobacteria_Reyranella
Gemmatimonadetes_uncultured
Verrucomicrobia_Chthoniobacter
Acidobacteria_Candidatus Solibacter
Acidobacteria_Bryobacter
Proteobacteria_Acidibacter
Actinobacteria_Acidothermus
Proteobacteria_Phenylobacterium
Actinobacteria_Mycobacterium
Chloroflexi_KD4-96_
Proteobacteria_uncultured
Actinobacteria_uncultured
Proteobacteria_Haliangium
Proteobacteria_Pseudolabrys
Proteobacteria_uncultured
Actinobacteria_uncultured
Acidobacteria_RB41
Gemmatimonadetes_Gemmatimonas
Proteobacteria_uncultured
Verrucomicrobia_uncultured
Proteobacteria_uncultured
Firmicutes_Bacillus
Bacteroidetes_Terrimonas
Actinobacteria_Streptomyces
Bacteroidetes_Flavobacterium
Proteobacteria_uncultured
Actinobacteria_IMCC26256_
Proteobacteria_Hirschia
Actinobacteria_Kribbella
Proteobacteria_Caballeronia-Paraburkholderia
Rest
5,70%
5,59%
3,97%
2,55%
2,49%
2,34%
2,16%
2,08%
1,71%
1,65%
1,59%
1,59%
1,56%
1,48%
1,39%
1,37%
1,21%
1,20%
1,20%
1,19%
1,15%
1,08%
1,08%
1,04%
0,97%
0,97%
0,94%
0,92%
0,88%
0,91%
0,88%
0,74%
0,76%
0,61%
0,61%
0,62%
0,59%
0,59%
0,59%
0,58%
0,57%
0,57%
0,56%
0,55%
0,54%
0,52%
36,20%

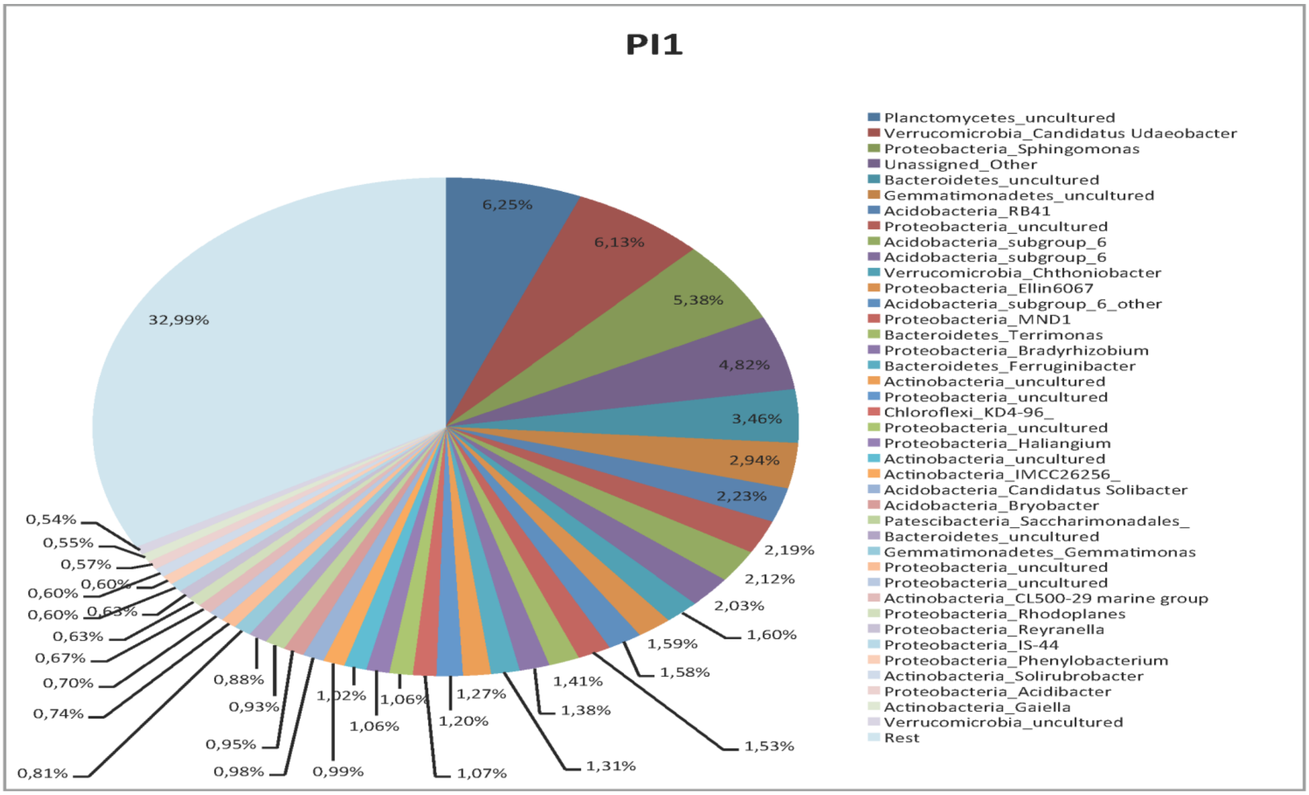
PI1
6,25%
6,13%
5,38%
4,82%
3,46%
2,94%
2,23%
2,19%
2,12%
2,03%
1,60%
1,59%
1,58%
1,53%
1,41%
1,38%
1,31%
1,27%
1,20%
1,07%
1,06%
1,06%
0,99%
0,98%
0,95%
0,93%
0,88%
0,81%
0,74%
0,70%
0,67%
0,63%
0,63%
0,60%
0,60%
0,60%
0,57%
0,55%
0,54%
32,99%
Planctomycetes_uncultured
Verrucomicrobia_Candidatus Udaeobacter
Proteobacteria_Sphingomonas
Unassigned_Other
Bacteroidetes_uncultured
Gemmatimonadetes_uncultured
Acidobacteria_RB41
Proteobacteria_uncultured
Acidobacteria_subgroup_6
Acidobacteria_subgroup_6
Verrucomicrobia_Chthoniobacter
Proteobacteria_Ellin6067
Acidobacteria_subgroup_6_other
Proteobacteria_MND1
Bacteroidetes_Terrimonas
Proteobacteria_Bradyrhizobium
Bacteroidetes_Ferruginibacter
Actinobacteria_uncultured
Proteobacteria_uncultured
Chloroflexi_KD4-96_
Proteobacteria_uncultured
Proteobacteria_Haliangium
Actinobacteria_uncultured
Actinobacteria_IMCC26256_
Acidobacteria_Candidatus Solibacter
Acidobacteria_Bryobacter
Patescibacteria_Saccharimonadales_
Bacteroidetes_uncultured
Gemmatimonadetes_Gemmatimonas
Proteobacteria_uncultured
Proteobacteria_uncultured
Actinobacteria_CL500-29 marine group
Proteobacteria_Rhodoplanes
Proteobacteria_Reyranella
Proteobacteria_IS-44
Proteobacteria_Phenylobacterium
Actinobacteria_Solirubrobacter
Proteobacteria_Acidibacter
Actinobacteria_Gaiella
Verrucomicrobia_uncultured
Rest

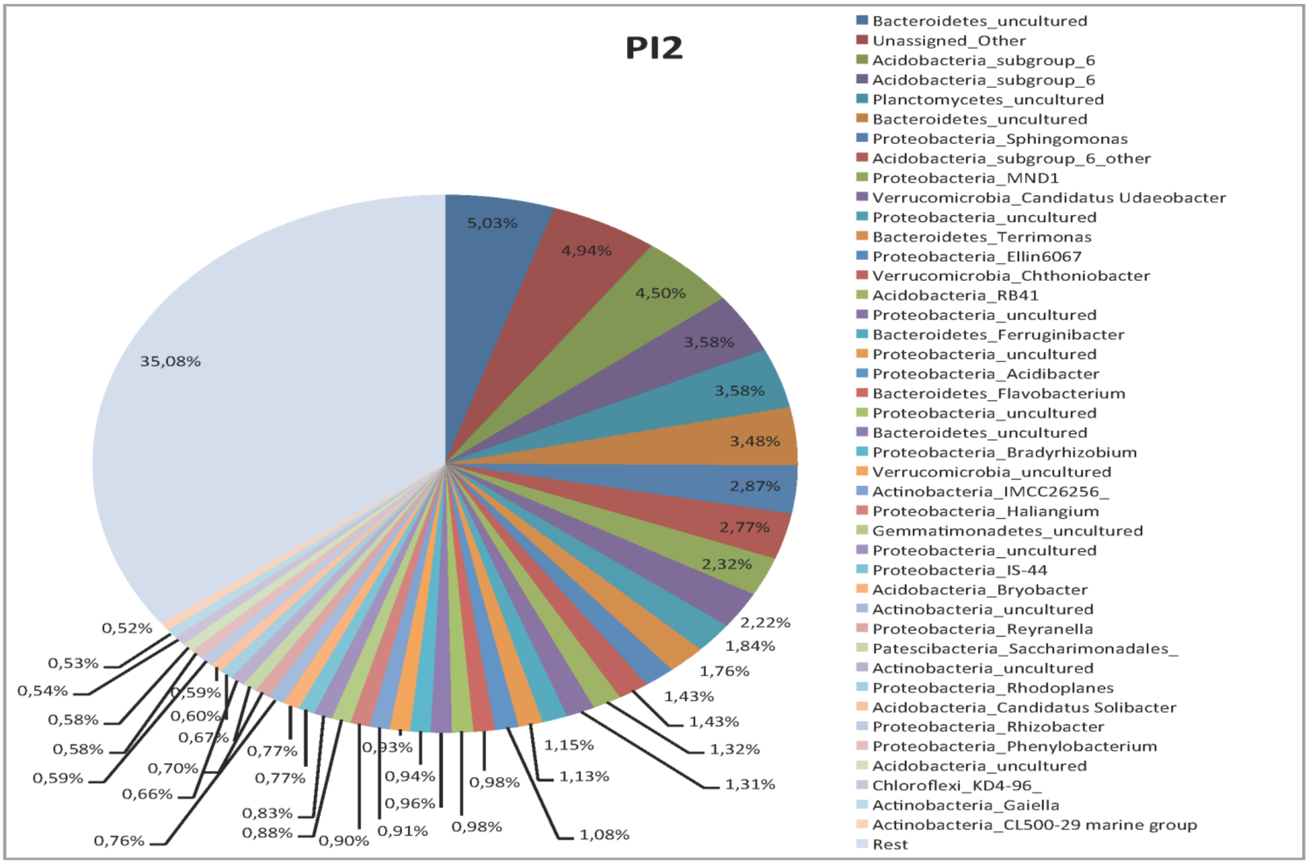
PI2
5,03%
4,94%
4,50%
3,58%
3,58%
3,48%
2,87%
2,77%
2,32%
2,22%
1,84%
1,76%
1,43%
1,43%
1,32%
1,31%
1,15%
1,13%
1,08%
0,98%
0,98%
0,96%
0,94%
0,93%
0,91%
0,90%
0,88%
0,83%
0,77%
0,77%
0,76%
0,70%
0,67%
0,66%
0,60%
0,59%
0,59%
0,58%
0,58%
0,54%
0,53%
0,52%
35,08%
Bacteroidetes_uncultured
Unassigned_Other
Acidobacteria_subgroup_6
Acidobacteria_subgroup_6
Planctomycetes_uncultured
Bacteroidetes_uncultured
Proteobacteria_Sphingomonas
Acidobacteria_subgroup_6_other
Proteobacteria_MND1
Verrucomicrobia_Candidatus Udaeobacter
Proteobacteria_uncultured
Bacteroidetes_Terrimonas
Proteobacteria_Ellin6067
Verrucomicrobia_Chthoniobacter
Acidobacteria_RB41
Proteobacteria_uncultured
Bacteroidetes_Ferruginibacter
Proteobacteria_uncultured
Proteobacteria_Acidibacter
Bacteroidetes_Flavobacterium
Proteobacteria_uncultured
Bacteroidetes_uncultured
Proteobacteria_Bradyrhizobium
Verrucomicrobia_uncultured
Actinobacteria_IMCC26256_
Proteobacteria_Haliangium
Gemmatimonadetes_uncultured
Proteobacteria_uncultured
Proteobacteria_IS-44
Acidobacteria_Bryobacter
Actinobacteria_uncultured
Proteobacteria_Reyranella
Patescibacteria_Saccharimonadales_
Actinobacteria_uncultured
Proteobacteria_Rhodoplanes
Acidobacteria_Candidatus Solibacter
Proteobacteria_Rhizobacter
Proteobacteria_Phenylobacterium
Acidobacteria_uncultured
Chloroflexi_KD4-96_
Actinobacteria_Gaiella
Actinobacteria_CL500-29 marine group
Rest

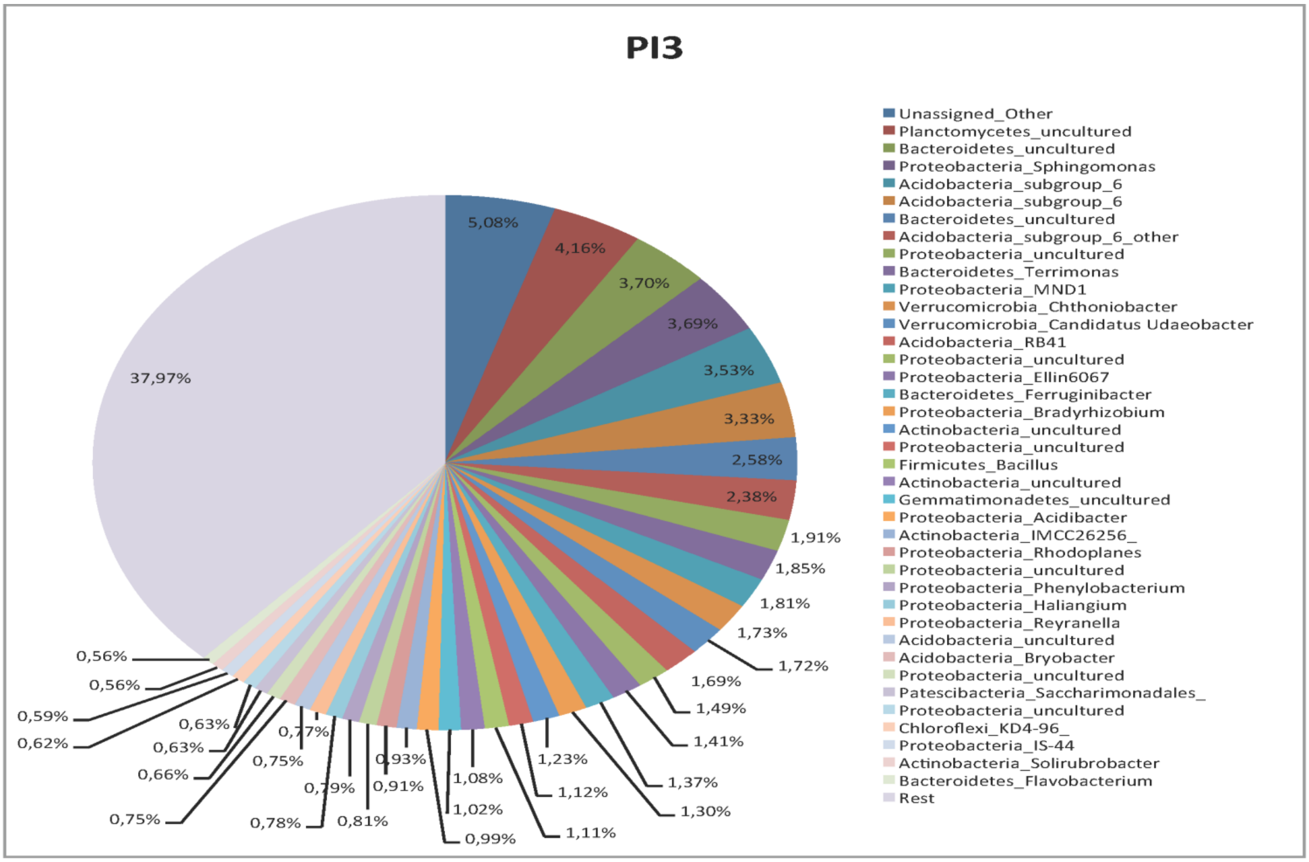
PI3
Unassigned_Other
Planctomycetes_uncultured
Bacteroidetes_uncultured
Proteobacteria_Sphingomonas
Acidobacteria_subgroup_6
Acidobacteria_subgroup_6
Bacteroidetes_uncultured
Acidobacteria_subgroup_6_other
Proteobacteria_uncultured
Bacteroidetes_Terrimonas
Proteobacteria_MND1
Verrucomicrobia_Chthoniobacter
Verrucomicrobia_Candidatus Udaeobacter
Acidobacteria_RB41
Proteobacteria_uncultured
Proteobacteria_Ellin6067
Bacteroidetes_Ferruginibacter
Proteobacteria_Bradyrhizobium
Actinobacteria_uncultured
Proteobacteria_uncultured
Firmicutes_Bacillus
Actinobacteria_uncultured
Gemmatimonadetes_uncultured
Proteobacteria_Acidibacter
Actinobacteria_IMCC26256_
Proteobacteria_Rhodoplanes
Proteobacteria_uncultured
Proteobacteria_Phenylobacterium
Proteobacteria_Haliangium
Proteobacteria_Reyranella
Acidobacteria_uncultured
Acidobacteria_Bryobacter
Proteobacteria_uncultured
Patescibacteria_Saccharimonadales_
Proteobacteria_uncultured
Chloroflexi_KD4-96_
Proteobacteria_IS-44
Actinobacteria_Solirubrobacter
Bacteroidetes_Flavobacterium
Rest
5,08%
4,16%
3,70%
3,69%
3,53%
3,33%
2,58%
2,38%
1,91%
1,85%
1,81%
1,73%
1,72%
1,69%
1,49%
1,41%
1,37%
1,30%
1,23%
1,12%
1,11%
1,08%
1,02%
0,99%
0,93%
0,91%
0,81%
0,79%
0,78%
0,77%
0,75%
0,75%
0,66%
0,63%
0,63%
0,62%
0,59%
0,56%
0,56%
37,97%

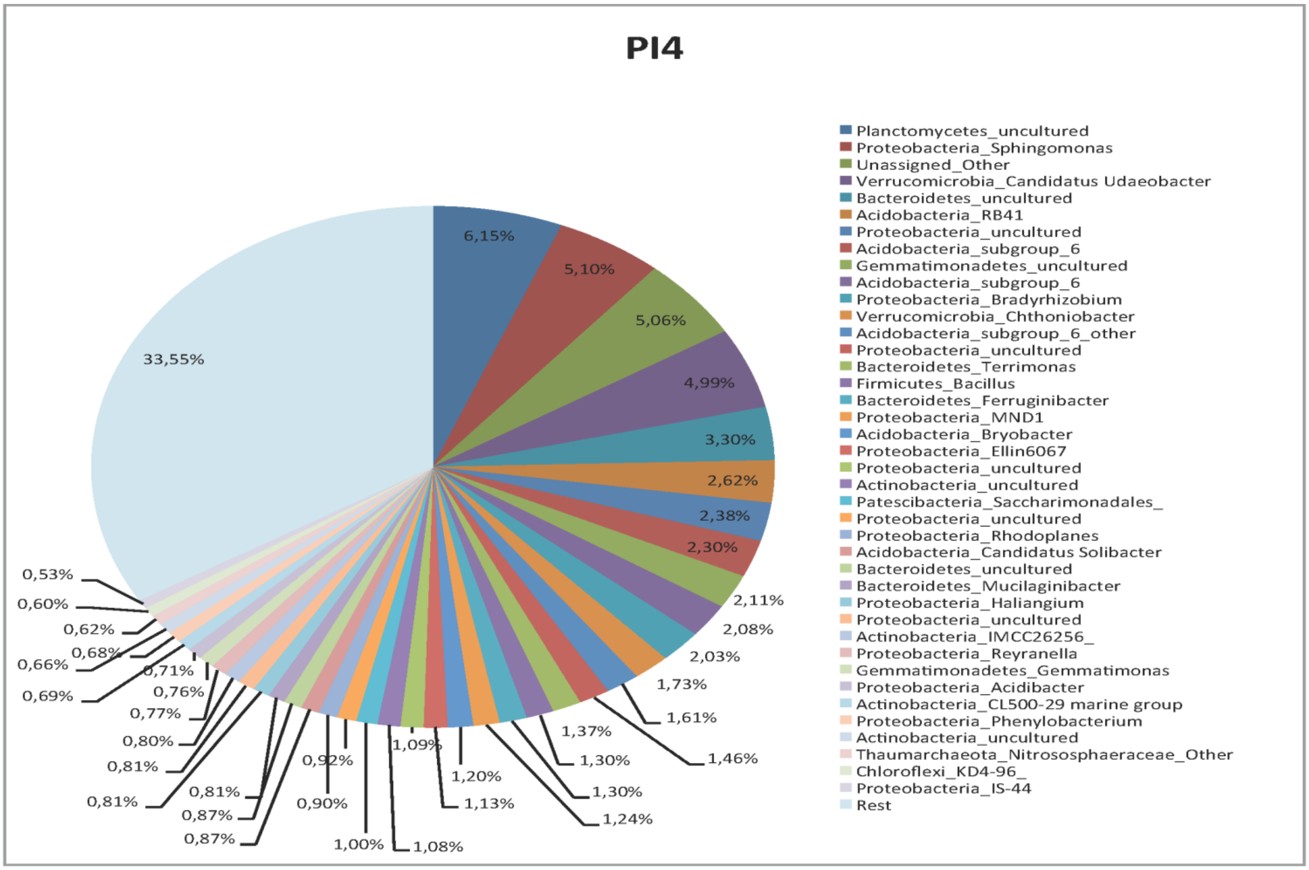
PI4
Planctomycetes_uncultured
Proteobacteria_Sphingomonas
Unassigned_Other
Verrucomicrobia_Candidatus Udaeobacter
Bacteroidetes_uncultured
Acidobacteria_RB41
Proteobacteria_uncultured
Acidobacteria_subgroup_6
Gemmatimonadetes_uncultured
Acidobacteria_subgroup_6
Proteobacteria_Bradyrhizobium
Verrucomicrobia_Chthoniobacter
Acidobacteria_subgroup_6_other
Proteobacteria_uncultured
Bacteroidetes_Terrimonas
Firmicutes_Bacillus
Bacteroidetes_Ferruginibacter
Proteobacteria_MND1
Acidobacteria_Bryobacter
Proteobacteria_Ellin6067
Proteobacteria_uncultured
Actinobacteria_uncultured
Patescibacteria_Saccharimonadales_
Proteobacteria_uncultured
Proteobacteria_Rhodoplanes
Acidobacteria_Candidatus Solibacter
Bacteroidetes_uncultured
Bacteroidetes_Mucilaginibacter
Proteobacteria_Haliangium
Proteobacteria_uncultured
Actinobacteria_IMCC26256_
Proteobacteria_Reyranella
Gemmatimonadetes_Gemmatimonas
Proteobacteria_Acidibacter
Actinobacteria_CL500-29 marine group
Proteobacteria_Phenylobacterium
Actinobacteria_uncultured
Thaumarchaeota_Nitrososphaeraceae_Other
Chloroflexi_KD4-96_
Proteobacteria_IS-44
Rest
6,15%
5,10%
5,06%
4,99%
3,30%
2,62%
2,38%
2,30%
2,11%
2,08%
2,03%
1,73%
1,61%
1,46%
1,37%
1,30%
1,30%
1,24%
1,20%
1,13%
1,09%
1,08%
1,00%
0,92%
0,90%
0,87%
0,87%
0,81%
0,81%
0,81%
0,80%
0,77%
0,76%
0,71%
0,69%
0,68%
0,66%
0,62%
0,60%
0,53%
33,55%

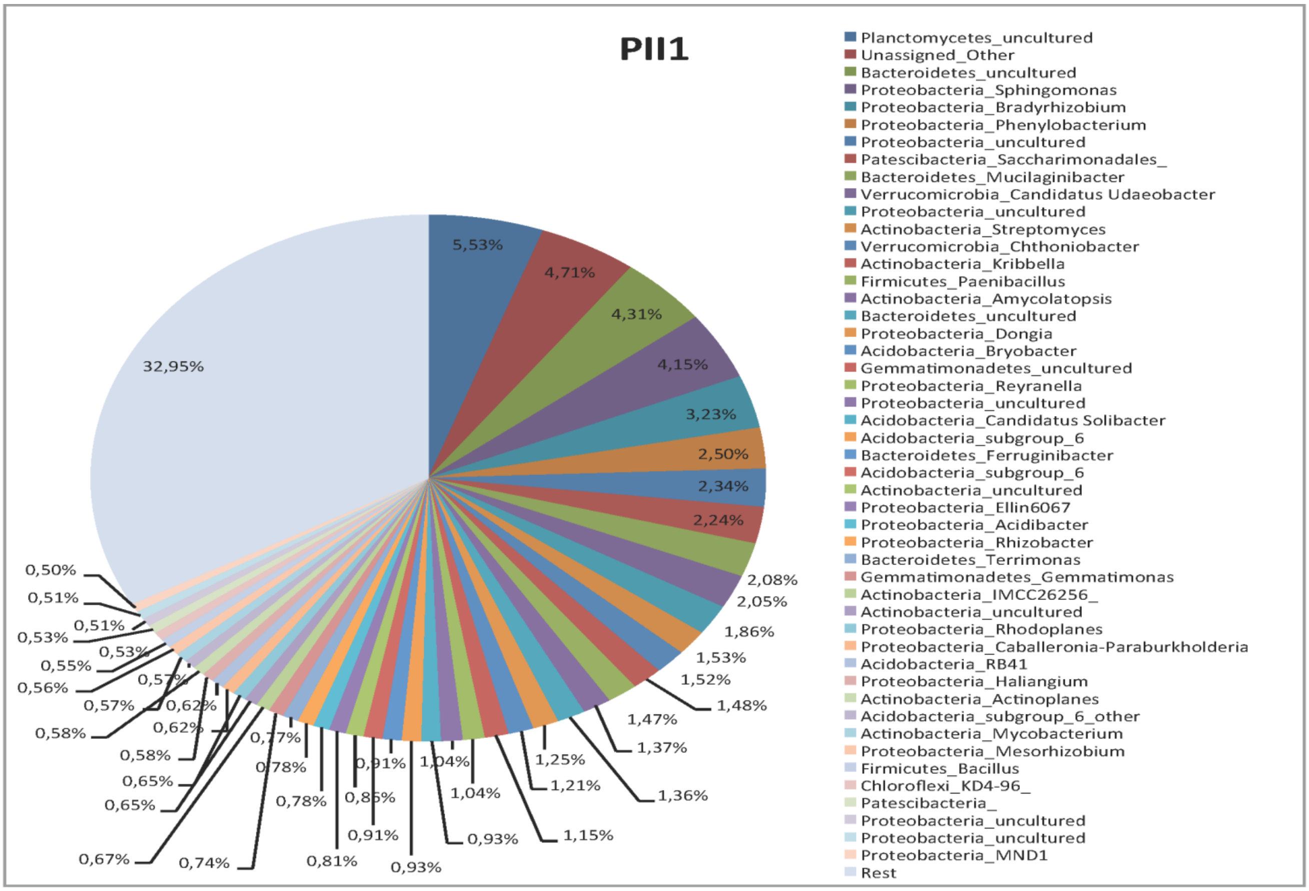
PII1
Planctomycetes_uncultured
Unassigned_Other
Bacteroidetes_uncultured
Proteobacteria_Sphingomonas
Proteobacteria_Bradyrhizobium
Proteobacteria_Phenylobacterium
Proteobacteria_uncultured
Patescibacteria_Saccharimonadales_
Bacteroidetes_Mucilaginibacter
Verrucomicrobia_Candidatus Udaeobacter
Proteobacteria_uncultured
Actinobacteria_Streptomyces
Verrucomicrobia_Chthoniobacter
Actinobacteria_Kribbella
Firmicutes_Paenibacillus
Actinobacteria_Amycolatopsis
Bacteroidetes_uncultured
Proteobacteria_Dongia
Acidobacteria_Bryobacter
Gemmatimonadetes_uncultured
Proteobacteria_Reyranella
Proteobacteria_uncultured
Acidobacteria_Candidatus Solibacter
Acidobacteria_subgroup_6
Bacteroidetes_Ferruginibacter
Acidobacteria_subgroup_6
Actinobacteria_uncultured
Proteobacteria_Ellin6067
Proteobacteria_Acidibacter
Proteobacteria_Rhizobacter
Bacteroidetes_Terrimonas
Gemmatimonadetes_Gemmatimonas
Actinobacteria_IMCC26256_
Actinobacteria_uncultured
Proteobacteria_Rhodoplanes
Proteobacteria_Caballeronia-Paraburkholderia
Acidobacteria_RB41
Proteobacteria_Haliangium
Actinobacteria_Actinoplanes
Acidobacteria_subgroup_6_other
Actinobacteria_Mycobacterium
Proteobacteria_Mesorhizobium
Firmicutes_Bacillus
Chloroflexi_KD4-96_
Patescibacteria_
Proteobacteria_uncultured
Proteobacteria_uncultured
Proteobacteria_MND1
Rest
5,53%
4,71%
4,31%
4,15%
3,23%
2,50%
2,34%
2,24%
2,08%
2,05%
1,86%
1,53%
1,52%
1,48%
1,47%
1,37%
1,36%
1,25%
1,21%
1,15%
1,04%
1,04%
0,93%
0,93%
0,91%
0,91%
0,85%
0,81%
0,78%
0,78%
0,77%
0,74%
0,67%
0,65%
0,65%
0,62%
0,62%
0,58%
0,58%
0,57%
0,57%
0,56%
0,55%
0,53%
0,53%
0,51%
0,51%
0,50%
32,95%

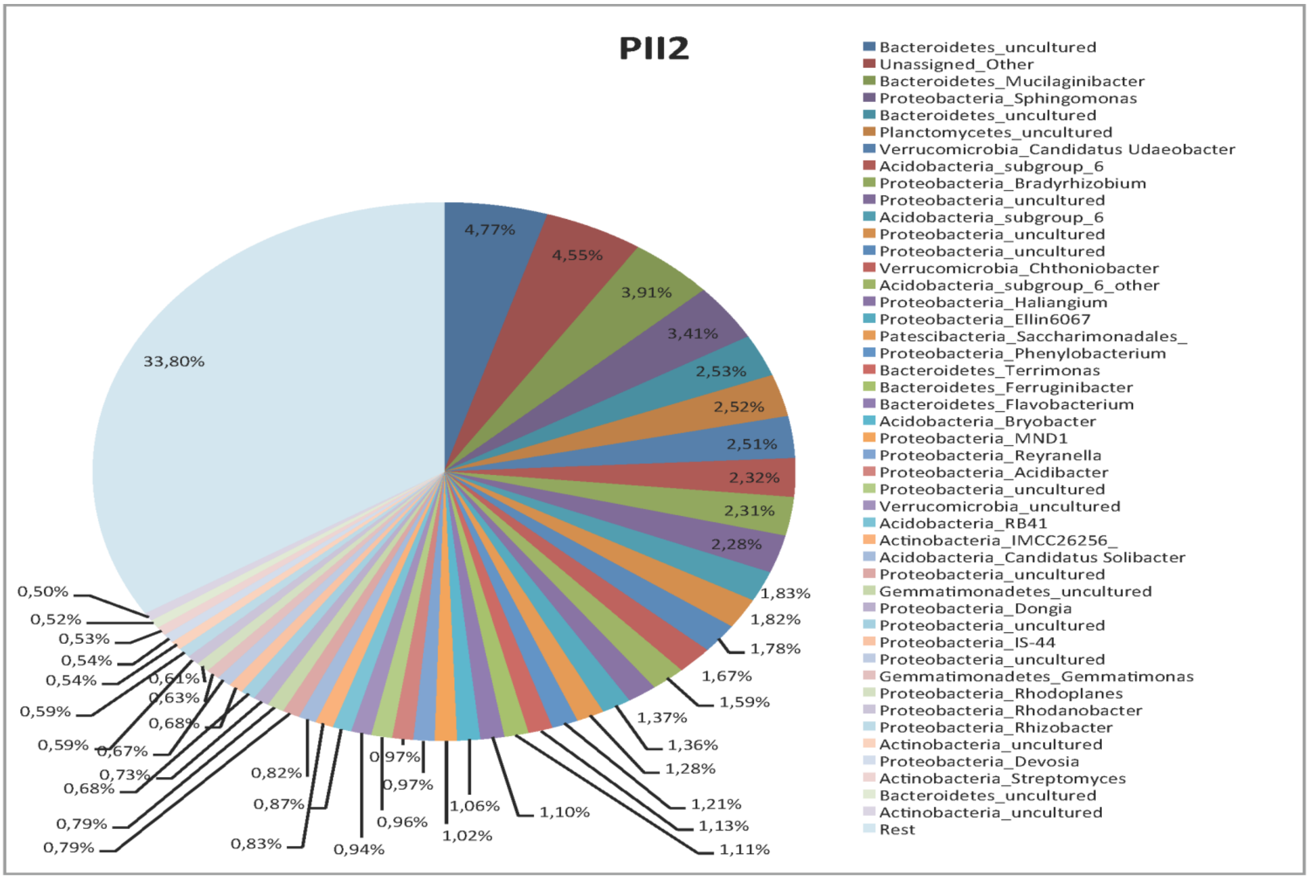
PII2
Bacteroidetes_uncultured
Unassigned_Other
Bacteroidetes_Mucilaginibacter
Proteobacteria_Sphingomonas
Bacteroidetes_uncultured
Planctomycetes_uncultured
Verrucomicrobia_Candidatus Udaeobacter
Acidobacteria_subgroup_6
Proteobacteria_Bradyrhizobium
Proteobacteria_uncultured
Acidobacteria_subgroup_6
Proteobacteria_uncultured
Proteobacteria_uncultured
Verrucomicrobia_Chthoniobacter
Acidobacteria_subgroup_6_other
Proteobacteria_Haliangium
Proteobacteria_Ellin6067
Patescibacteria_Saccharimonadales_
Proteobacteria_Phenylobacterium
Bacteroidetes_Terrimonas
Bacteroidetes_Ferruginibacter
Bacteroidetes_Flavobacterium
Acidobacteria_Bryobacter
Proteobacteria_MND1
Proteobacteria_Reyranella
Proteobacteria_Acidibacter
Proteobacteria_uncultured
Verrucomicrobia_uncultured
Acidobacteria_RB41
Actinobacteria_IMCC26256_
Acidobacteria_Candidatus Solibacter
Proteobacteria_uncultured
Gemmatimonadetes_uncultured
Proteobacteria_Dongia
Proteobacteria_uncultured
Proteobacteria_IS-44
Proteobacteria_uncultured
Gemmatimonadetes_Gemmatimonas
Proteobacteria_Rhodoplanes
Proteobacteria_Rhodanobacter
Proteobacteria_Rhizobacter
Actinobacteria_uncultured
Proteobacteria_Devosia
Actinobacteria_Streptomyces
Bacteroidetes_uncultured
Actinobacteria_uncultured
Rest
4,77%
4,55%
3,91%
3,41%
2,53%
2,52%
2,51%
2,32%
2,31%
2,28%
1,83%
1,82%
1,78%
1,67%
1,59%
1,37%
1,36%
1,28%
1,21%
1,13%
1,11%
1,10%
1,06%
1,02%
0,97%
0,97%
0,96%
0,94%
0,87%
0,83%
0,82%
0,79%
0,79%
0,73%
0,68%
0,68%
0,67%
0,63%
0,61%
0,59%
0,59%
0,54%
0,54%
0,53%
0,52%
0,50%
33,80%

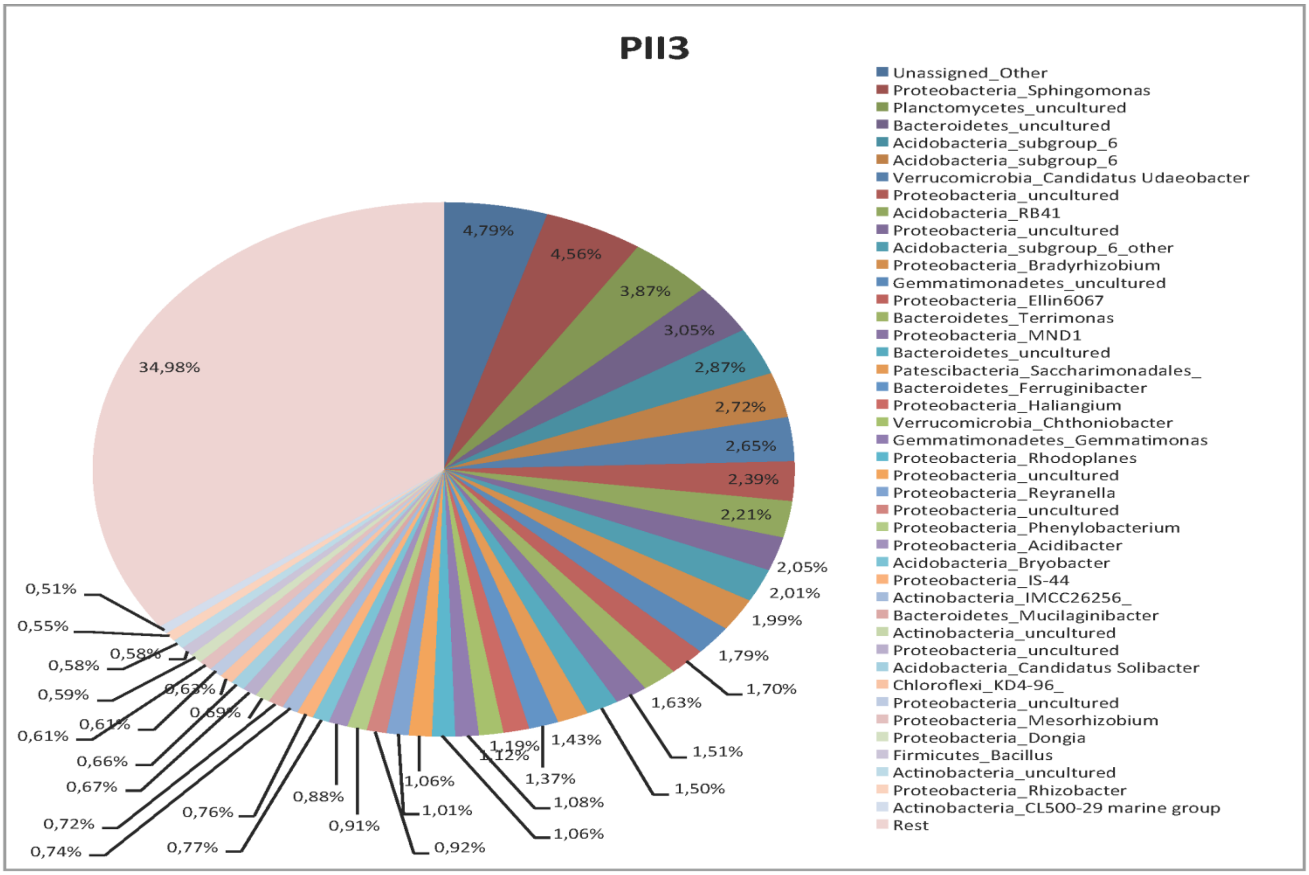
PII3
4,79%
4,56%
3,87%
3,05%
2,87%
2,72%
2,65%
2,39%
2,21%
2,05%
2,01%
1,99%
1,79%
1,70%
1,63%
1,51%
1,50%
1,43%
1,37%
1,19%
1,12%
1,08%
1,06%
1,06%
1,01%
0,92%
0,91%
0,88%
0,77%
0,76%
0,74%
0,72%
0,69%
0,67%
0,66%
0,63%
0,61%
0,61%
0,59%
0,58%
0,58%
0,55%
0,51%
34,98%
Unassigned_Other
Proteobacteria_Sphingomonas
Planctomycetes_uncultured
Bacteroidetes_uncultured
Acidobacteria_subgroup_6
Acidobacteria_subgroup_6
Verrucomicrobia_Candidatus Udaeobacter
Proteobacteria_uncultured
Acidobacteria_RB41
Proteobacteria_uncultured
Acidobacteria_subgroup_6_other
Proteobacteria_Bradyrhizobium
Gemmatimonadetes_uncultured
Proteobacteria_Ellin6067
Bacteroidetes_Terrimonas
Proteobacteria_MND1
Bacteroidetes_uncultured
Patescibacteria_Saccharimonadales_
Bacteroidetes_Ferruginibacter
Proteobacteria_Haliangium
Verrucomicrobia_Chthoniobacter
Gemmatimonadetes_Gemmatimonas
Proteobacteria_Rhodoplanes
Proteobacteria_uncultured
Proteobacteria_Reyranella
Proteobacteria_uncultured
Proteobacteria_Phenylobacterium
Proteobacteria_Acidibacter
Acidobacteria_Bryobacter
Proteobacteria_IS-44
Actinobacteria_IMCC26256_
Bacteroidetes_Mucilaginibacter
Actinobacteria_uncultured
Proteobacteria_uncultured
Acidobacteria_Candidatus Solibacter
Chloroflexi_KD4-96_
Proteobacteria_uncultured
Proteobacteria_Mesorhizobium
Proteobacteria_Dongia
Firmicutes_Bacillus
Actinobacteria_uncultured
Proteobacteria_Rhizobacter
Actinobacteria_CL500-29 marine group
Rest

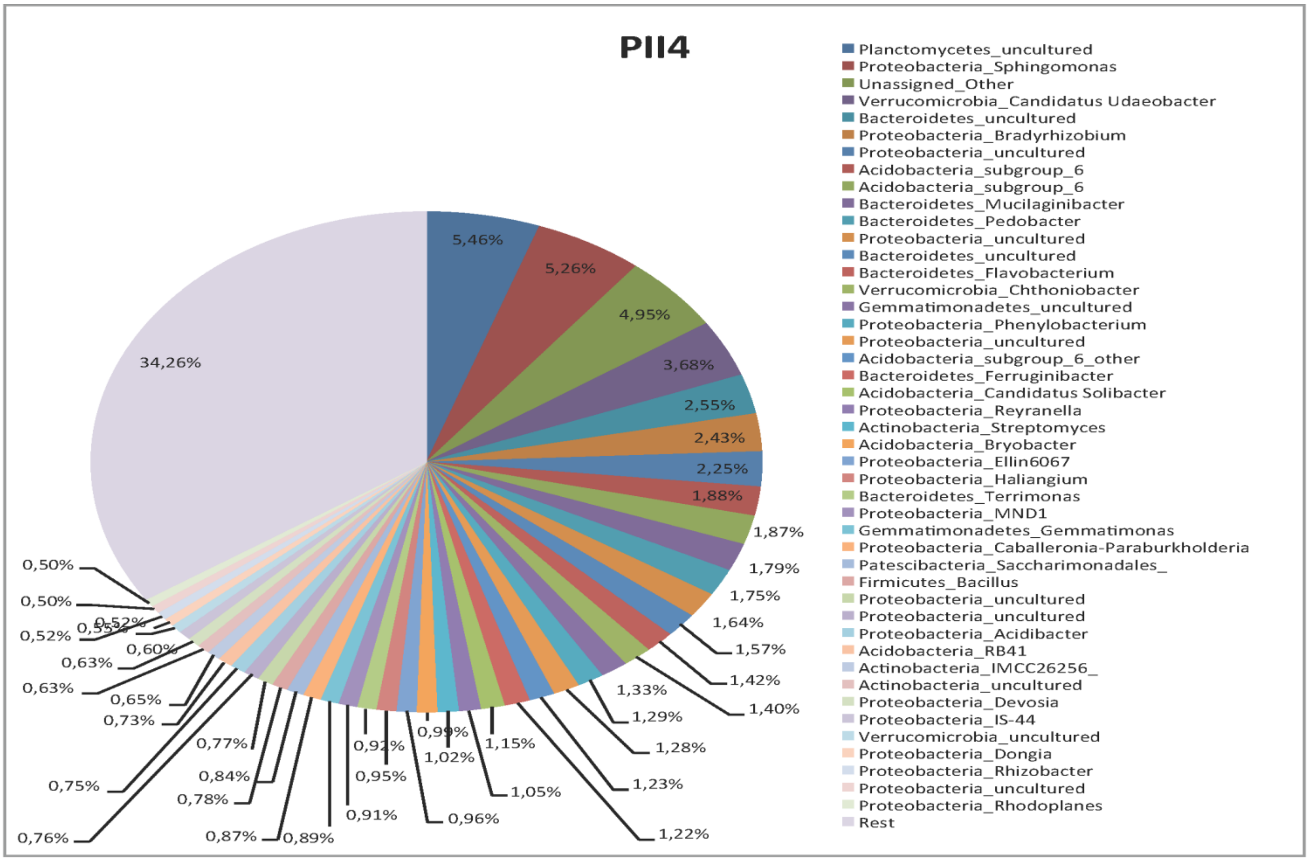
PII4
5,46%
5,26%
4,95%
3,68%
2,55%
2,43%
2,25%
1,88%
1,87%
1,79%
1,75%
1,64%
1,57%
1,42%
1,40%
1,33%
1,29%
1,28%
1,23%
1,22%
1,15%
1,05%
1,02%
0,99%
0,96%
0,95%
0,92%
0,91%
0,89%
0,87%
0,84%
0,78%
0,77%
0,76%
0,75%
0,73%
0,65%
0,63%
0,63%
0,60%
0,52%
0,52%
0,50%
0,50%
34,26%
Planctomycetes_uncultured
Proteobacteria_Sphingomonas
Unassigned_Other
Verrucomicrobia_Candidatus Udaeobacter
Bacteroidetes_uncultured
Proteobacteria_Bradyrhizobium
Proteobacteria_uncultured
Acidobacteria_subgroup_6
Acidobacteria_subgroup_6
Bacteroidetes_Mucilaginibacter
Bacteroidetes_Pedobacter
Proteobacteria_uncultured
Bacteroidetes_uncultured
Bacteroidetes_Flavobacterium
Verrucomicrobia_Chthoniobacter
Gemmatimonadetes_uncultured
Proteobacteria_Phenylobacterium
Proteobacteria_uncultured
Acidobacteria_subgroup_6_other
Bacteroidetes_Ferruginibacter
Acidobacteria_Candidatus Solibacter
Proteobacteria_Reyranella
Actinobacteria_Streptomyces
Acidobacteria_Bryobacter
Proteobacteria_Ellin6067
Proteobacteria_Haliangium
Bacteroidetes_Terrimonas
Proteobacteria_MND1
Gemmatimonadetes_Gemmatimonas
Proteobacteria_Caballeronia-Paraburkholderia
Patescibacteria_Saccharimonadales_
Firmicutes_Bacillus
Proteobacteria_uncultured
Proteobacteria_uncultured
Proteobacteria_Acidibacter
Acidobacteria_RB41
Actinobacteria_IMCC26256_
Actinobacteria_uncultured
Proteobacteria_Devosia
Proteobacteria_IS-44
Verrucomicrobia_uncultured
Proteobacteria_Dongia
Proteobacteria_Rhizobacter
Proteobacteria_uncultured
Proteobacteria_Rhodoplanes
Rest

Supplementary Figure S5. The most abundant genera (>0.5%) of fungi in the 4 sampling sites (1,2,3,4) and the two season periods (I=winter, II=summer) of the Andros (A) and Parnitha (P) forests.

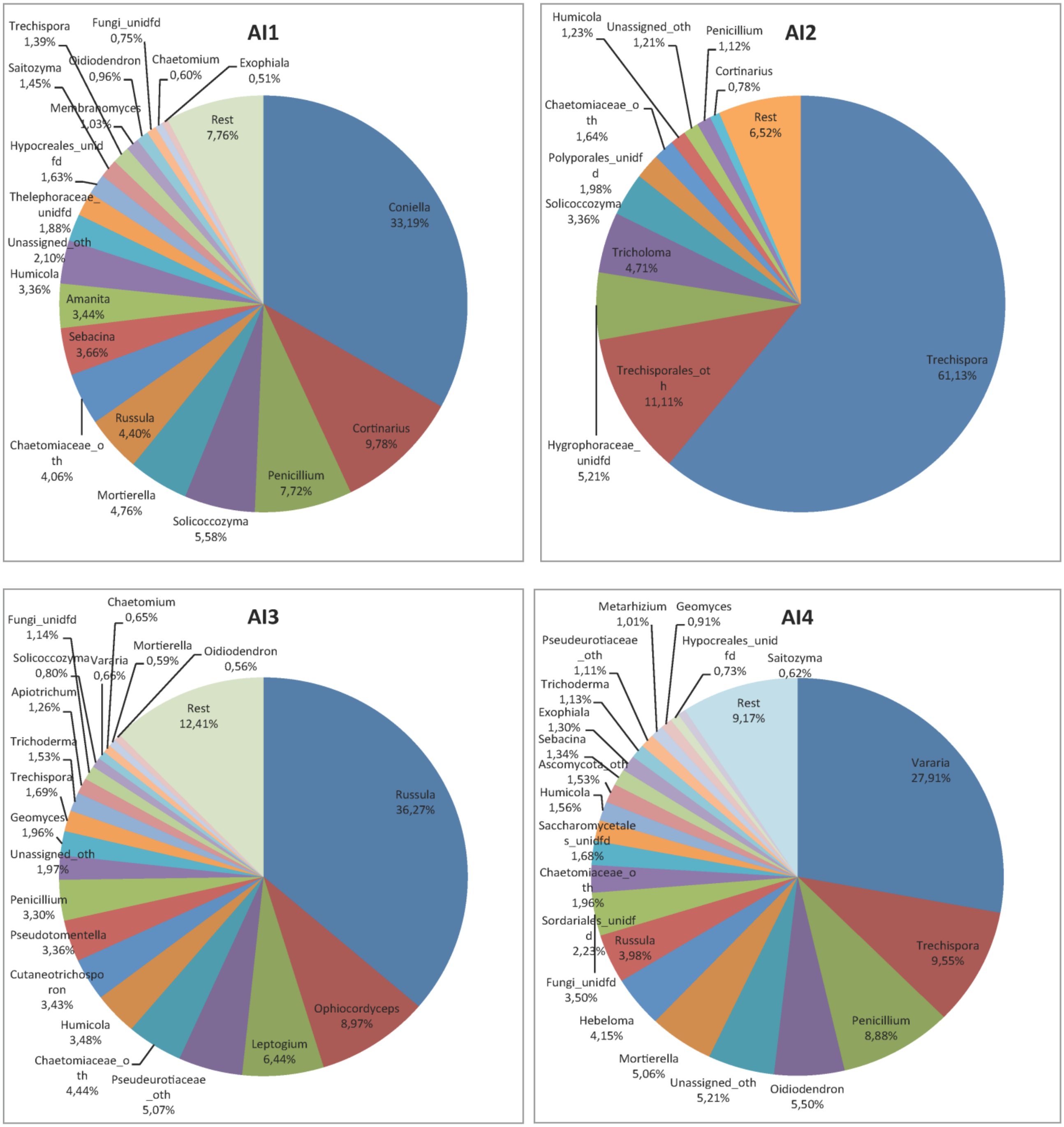

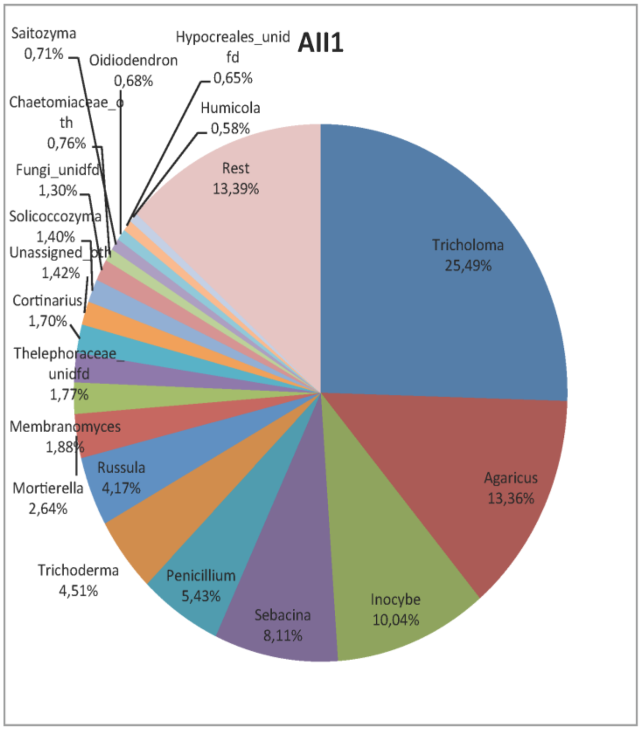
AII1
Saitozyma 0,71%
Oidiodendron 0,68%
Hypocreales_unidfd 0,65%
Humicola 0,58%
Chaetomiaceae_oth 0,76%
Fungi_unidfd 1,30%
Solicoccozyma 1,40%
Unassigned_oth 1,42%
Cortinarius 1,70%
Thelephoraceae_unidfd 1,77%
Membranomyces 1,88%
Mortierella 2,64%
Russula 4,17%
Trichoderma 4,51%
Penicillium 5,43%
Sebacina 8,11%
Inocybe 10,04%
Agaricus 13,36%
Tricholoma 25,49%
Rest 13,39%

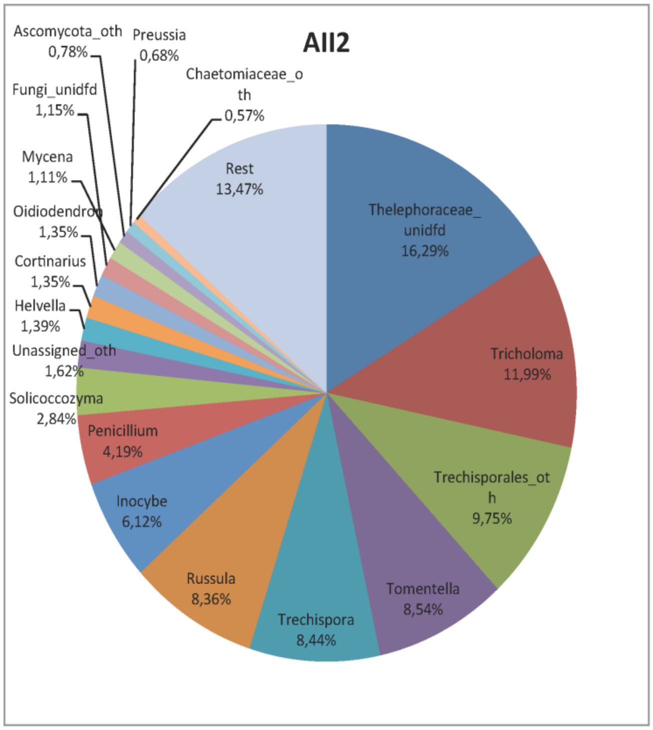
AII2
Ascomycota_oth 0,78%
Preussia 0,68%
Chaetomiaceae_oth 0,57%
Fungi_unidfd 1,15%
Mycena 1,11%
Oidiodendron 1,35%
Cortinarius 1,35%
Helvella 1,39%
Unassigned_oth 1,62%
Solicoccozyma 2,84%
Penicillium 4,19%
Inocybe 6,12%
Russula 8,36%
Trechispora 8,44%
Tomentella 8,54%
Trechisporales_oth 9,75%
Tricholoma 11,99%
Thelephoraceae_unidfd 16,29%
Rest 13,47%

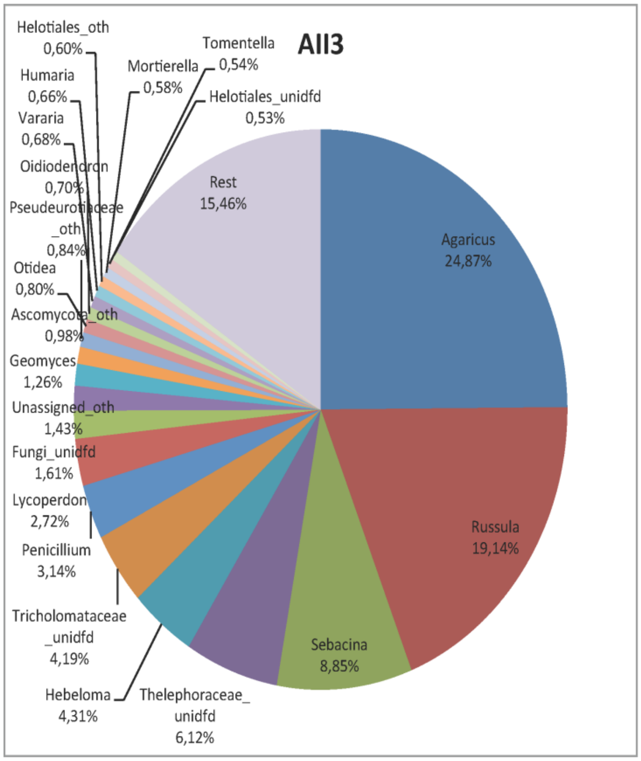
AII3
Helotiales_oth 0,60%
Tomentella 0,54%
Mortierella 0,58%
Helotiales_unidfd 0,53%
Humaria 0,66%
Vararia 0,68%
Oidiodendron 0,70%
Pseudeurotiaceae_oth 0,84%
Otidea 0,80%
Ascomycota_oth 0,98%
Geomyces 1,26%
Unassigned_oth 1,43%
Fungi_unidfd 1,61%
Lycoperdon 2,72%
Penicillium 3,14%
Tricholomataceae_unidfd 4,19%
Hebeloma 4,31%
Thelephoraceae_unidfd 6,12%
Sebacina 8,85%
Russula 19,14%
Agaricus 24,87%
Rest 15,46%

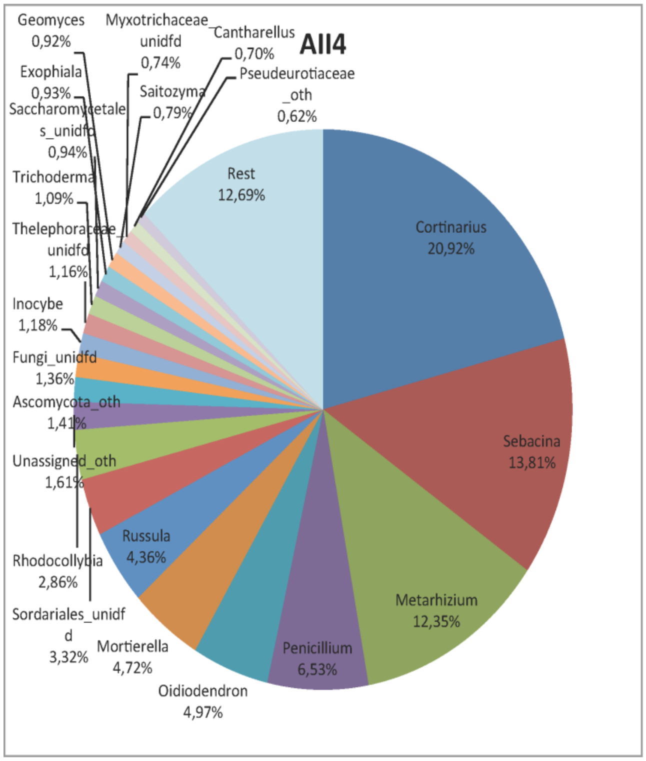
AII4
Geomyces 0,92%
Myxotrichaceae unidfd 0,74%
Cantharellus 0,70%
Pseudeurotiaceae_oth 0,62%
Exophiala 0,93%
Saitozyma 0,79%
Saccharomycetales_unidfd 0,94%
Trichoderma 1,09%
Thelephoraceae_unidfd 1,16%
Inocybe 1,18%
Fungi_unidfd 1,36%
Ascomycota_oth 1,41%
Unassigned_oth 1,61%
Rhodocollybia 2,86%
Sordariales_unidfd 3,32%
Russula 4,36%
Mortierella 4,72%
Oidiodendron 4,97%
Penicillium 6,53%
Metarhizium 12,35%
Sebacina 13,81%
Cortinarius 20,92%
Rest 12,69%

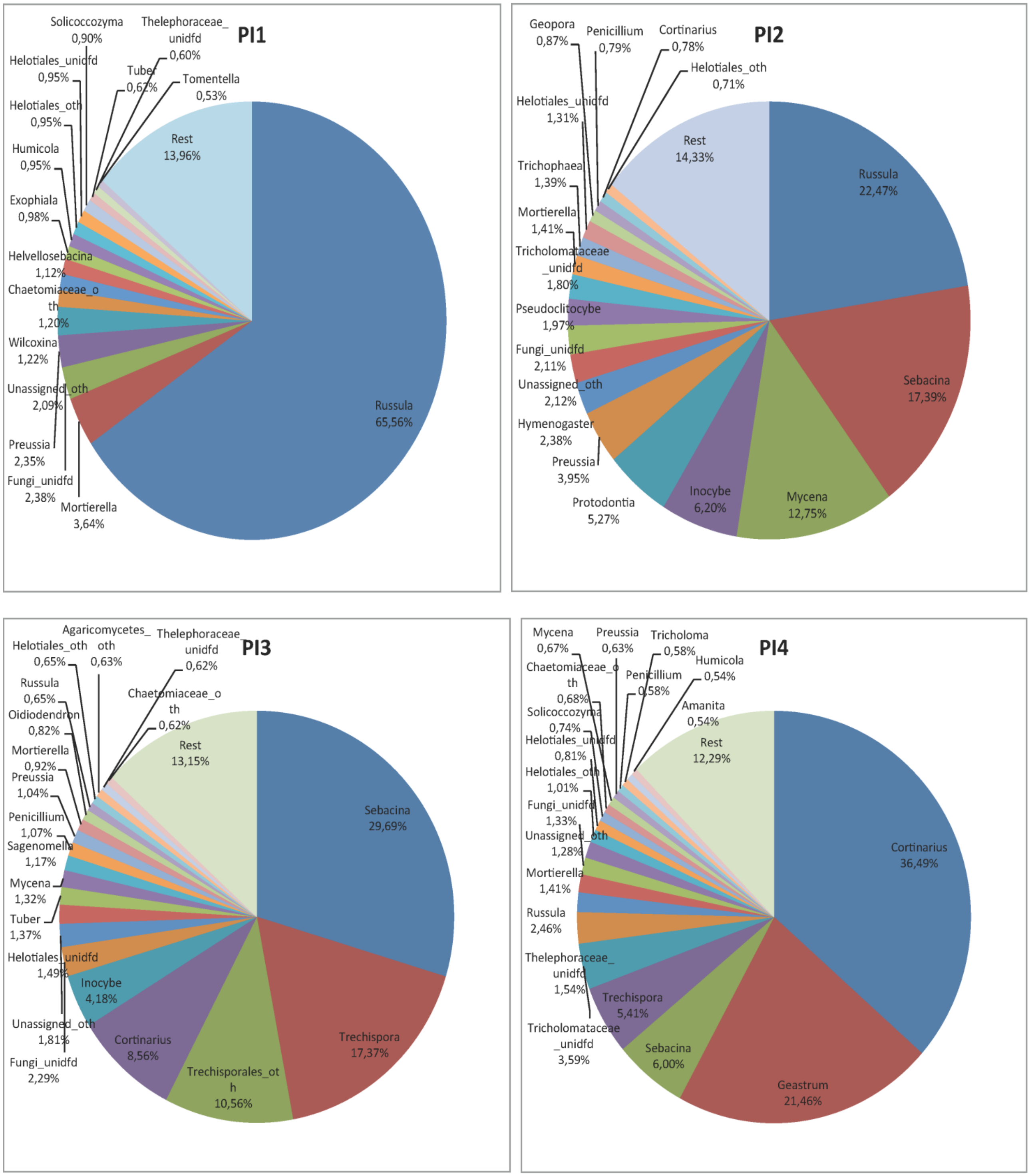
PI1
Solicoccozyma 0,90%
Thelephoraceae_unidfd 0,60%
Helotiales_unidfd 0,95%
Tuber 0,62%
Tomentella 0,53%
Helotiales_oth 0,95%
Humicola 0,95%
Exophiala 0,98%
Helvellosebacina 1,12%
Chaetomiaceae_oth 1,20%
Wilcoxina 1,22%
Unassigned_oth 2,09%
Preussia 2,35%
Fungi_unidfd 2,38%
Mortierella 3,64%
Rest 13,96%
Russula 65,56%
PI2
Geopora 0,87%
Penicillium 0,79%
Cortinarius 0,78%
Helotiales_oth 0,71%
Helotiales_unidfd 1,31%
Trichophaea 1,39%
Mortierella 1,41%
Tricholomataceae_unidfd 1,80%
Pseudoclitocybe 1,97%
Fungi_unidfd 2,11%
Unassigned_oth 2,12%
Hymenogaster 2,38%
Preussia 3,95%
Protodontia 5,27%
Inocybe 6,20%
Mycena 12,75%
Sebacina 17,39%
Russula 22,47%
Rest 14,33%
PI3
Agaricomycetes_oth 0,63%
Thelephoraceae_unidfd 0,62%
Helotiales_oth 0,65%
Russula 0,65%
Oidiodendron 0,82%
Chaetomiaceae_oth 0,62%
Mortierella 0,92%
Preussia 1,04%
Penicillium 1,07%
Sagenomella 1,17%
Mycena 1,32%
Tuber 1,37%
Helotiales_unidfd 1,49%
Inocybe 4,18%
Unassigned_oth 1,81%
Fungi_unidfd 2,29%
Cortinarius 8,56%
Trechisporales_oth 10,56%
Trechispora 17,37%
Sebacina 29,69%
Rest 13,15%
PI4
Mycena 0,67%
Preussia 0,63%
Tricholoma 0,58%
Humicola 0,54%
Chaetomiaceae_oth 0,68%
Penicillium 0,58%
Amanita 0,54%
Solicoccozyma 0,74%
Helotiales_unidfd 0,81%
Helotiales_oth 1,01%
Fungi_unidfd 1,33%
Unassigned_oth 1,28%
Mortierella 1,41%
Russula 2,46%
Thelephoraceae_unidfd 1,54%
Trechispora 5,41%
Tricholomataceae_unidfd 3,59%
Sebacina 6,00%
Geastrum 21,46%
Cortinarius 36,49%
Rest 12,29%

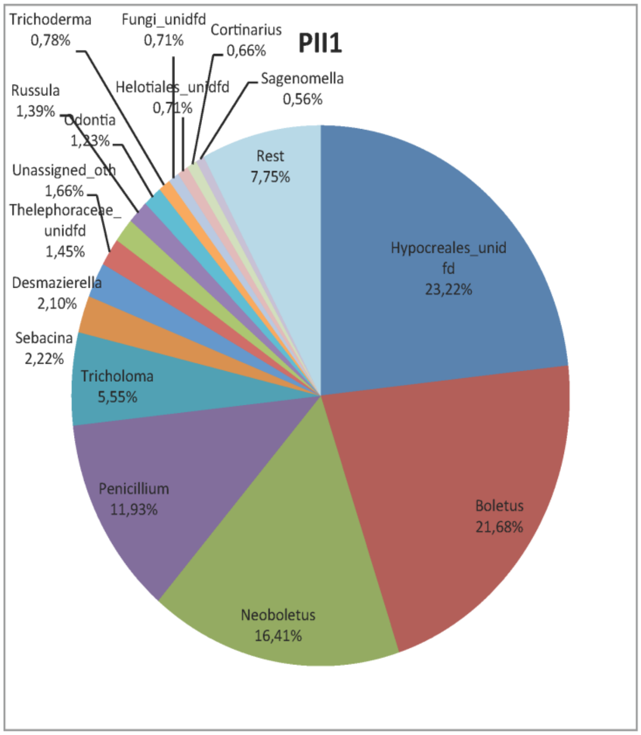
PII1
Trichoderma 0,78%
Fungi_unidfd 0,71%
Cortinarius 0,66%
Russula 1,39%
Helotiales_unidfd 0,71%
Sagenomella 0,56%
Odontia 1,23%
Unassigned_oth 1,66%
Thelephoraceae_unidfd 1,45%
Desmazierella 2,10%
Sebacina 2,22%
Tricholoma 5,55%
Penicillium 11,93%
Neoboletus 16,41%
Boletus 21,68%
Hypocreales_unidfd 23,22%
Rest 7,75%

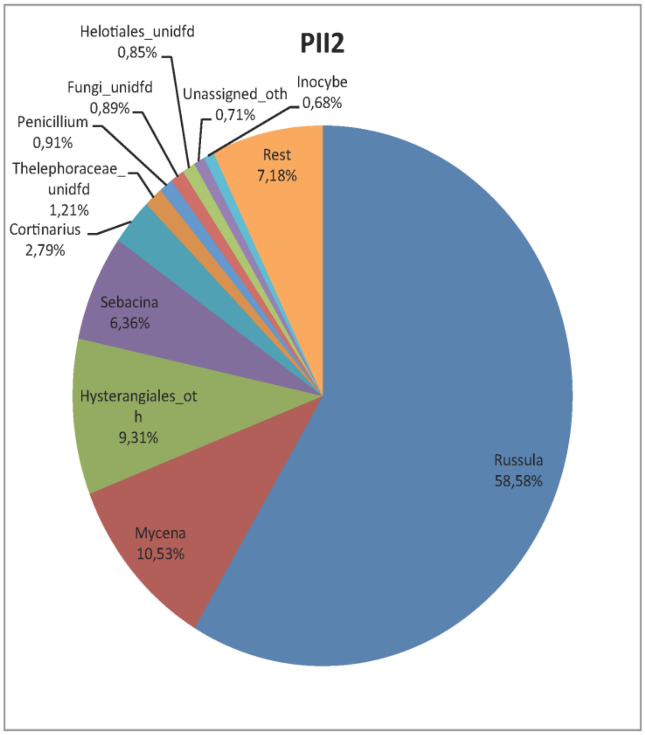
PII2
Helotiales_unidfd 0,85%
Fungi_unidfd 0,89%
Unassigned_oth 0,71%
Inocybe 0,68%
Penicillium 0,91%
Thelephoraceae_unidfd 1,21%
Cortinarius 2,79%
Rest 7,18%
Sebacina 6,36%
Hysterangiales_oth 9,31%
Mycena 10,53%
Russula 58,58%

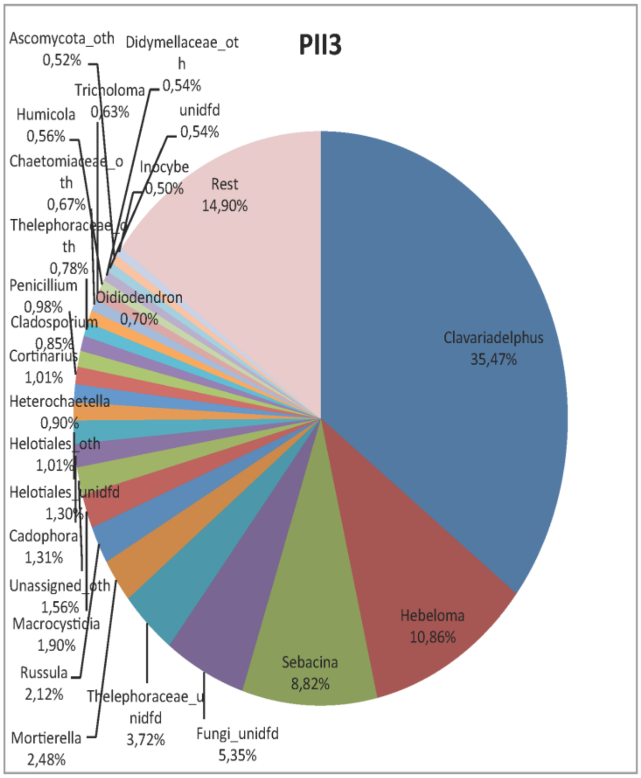
PII3
Ascomycota_oth 0,52%
Didymellaceae_oth 0,54%
Tricholoma 0,63%
Humicola 0,56%
unidfd 0,54%
Chaetomiaceae_oth 0,67%
Inocybe 0,50%
Rest 14,90%
Thelephoraceae_oth 0,78%
Penicillium 0,98%
Oidiodendron 0,70%
Cladosporium 0,85%
Cortinarius 1,01%
Heterochaetella 0,90%
Helotiales_oth 1,01%
Helotiales_unidfd 1,30%
Cadophora 1,31%
Unassigned_oth 1,56%
Macrocystidia 1,90%
Russula 2,12%
Mortierella 2,48%
Thelephoraceae_unidfd 3,72%
Fungi_unidfd 5,35%
Sebacina 8,82%
Hebeloma 10,86%
Clavariadelphus 35,47%

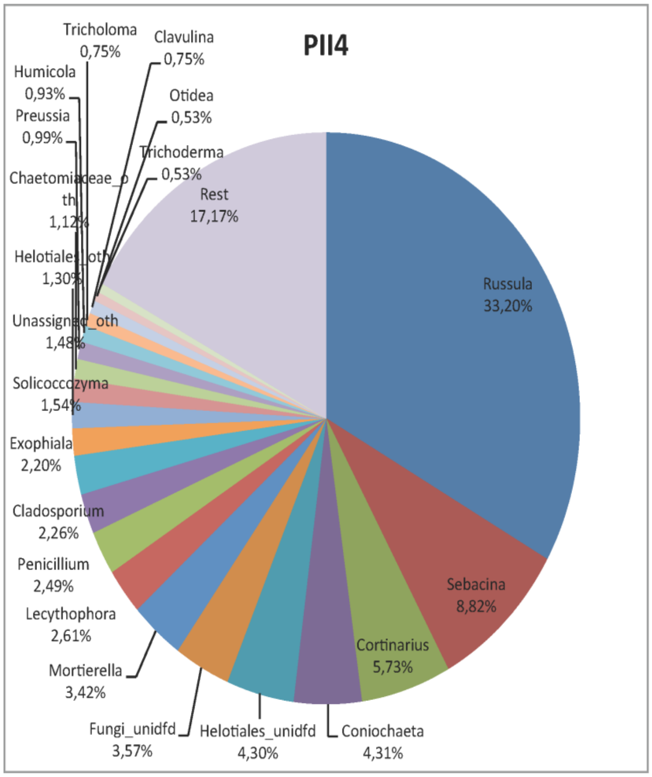
PII4
Tricholoma 0,75%
Clavulina 0,75%
Humicola 0,93%
Preussia 0,99%
Otidea 0,53%
Trichoderma 0,53%
Chaetomiaceae_oth 1,12%
Rest 17,17%
Helotiales_oth 1,30%
Unassigned_oth 1,48%
Solicoccozyma 1,54%
Exophiala 2,20%
Cladosporium 2,26%
Penicillium 2,49%
Lecythophora 2,61%
Mortierella 3,42%
Fungi_unidfd 3,57%
Helotiales_unidfd 4,30%
Coniochaeta 4,31%
Cortinarius 5,73%
Sebacina 8,82%
Russula 33,20%

Figure S6. Phylogenetic placemement of the predicted protein sequences of LiP (**A**) and VP (**B**) peroxidases amplified from soil samples of both forests (blue print) in comparison with known sequences from published genomes of *Basidiomycetes*. [horizontal bar = mean length between branches; vertical axis (coloured boxes) = bootstrap support]. The trees were built on RAxML and GTR+GAMMA model with 1000 bootstrap replications.

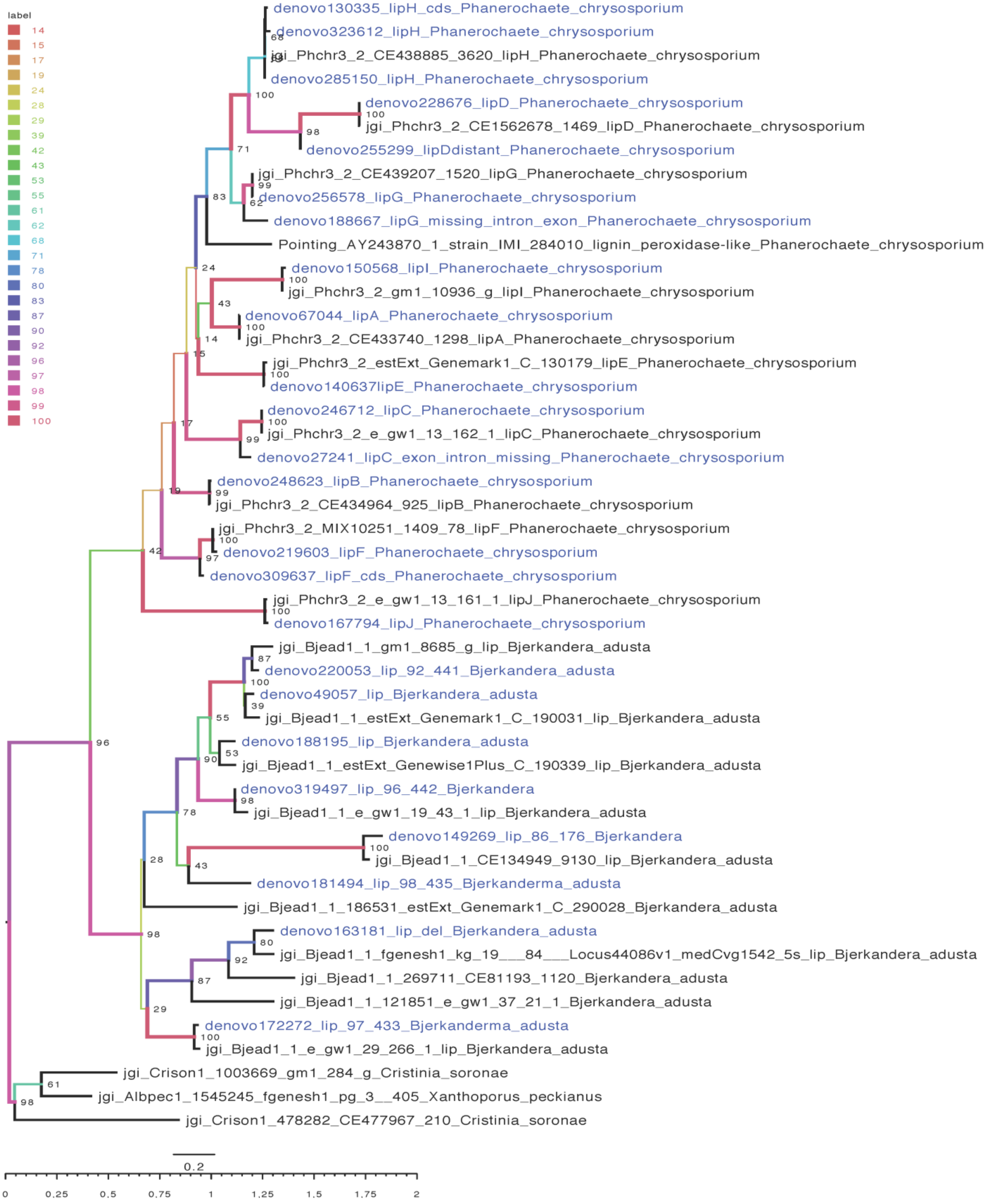

(**A**)

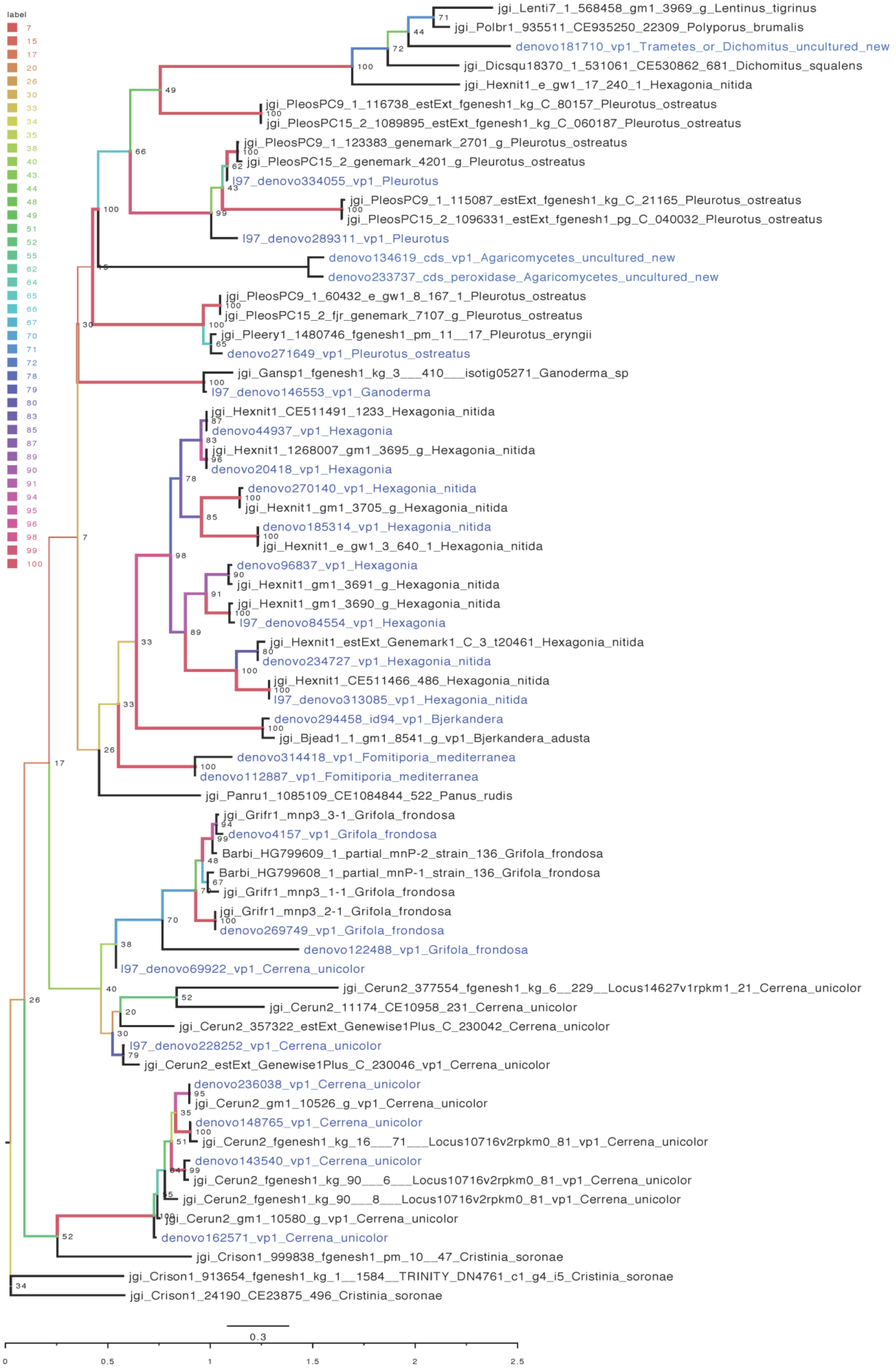
label
jgi_Lenti7_1_568458_gm1_3969_g_Lentinus_tigrinus
jgi_Polbr1_935511_CE935250_22309_Polyporus_brumalis
denovo181710_vp1_Trametes_or_Dichomitus_uncultured_new
jgi_Dicsqu18370_1_531061_CE530862_681_Dichomitus_squalens
jgi_Hexnit1_e_gw1_17_240_1_Hexagonia_nitida
jgi_PleosPC9_1_116738_estExt_fgenesh1_kg_C_80157_Pleurotus_ostreatus
jgi_PleosPC15_2_1089895_estExt_fgenesh1_kg_C_060187_Pleurotus_ostreatus
jgi_PleosPC9_1_123383_genemark_2701_g_Pleurotus_ostreatus
jgi_PleosPC15_2_genemark_4201_g_Pleurotus_ostreatus
I97_denovo334055_vp1_Pleurotus
jgi_PleosPC9_1_115087_estExt_fgenesh1_kg_C_21165_Pleurotus_ostreatus
jgi_PleosPC15_2_1096331_estExt_fgenesh1_pg_C_040032_Pleurotus_ostreatus
I97_denovo289311_vp1_Pleurotus
denovo134619_cds_vp1_Agaricomycetes_uncultured_new
denovo233737_cds_peroxidase_Agaricomycetes_uncultured_new
jgi_PleosPC9_1_60432_e_gw1_8_167_1_Pleurotus_ostreatus
jgi_PleosPC15_2_fjr_genemark_7107_g_Pleurotus_ostreatus
jgi_Pleery1_1480746_fgenesh1_pm_11__17_Pleurotus_eryngii
denovo271649_vp1_Pleurotus_ostreatus
jgi_Gansp1_fgenesh1_kg_3___410___isotig05271_Ganoderma_sp
I97_denovo146553_vp1_Ganoderma
jgi_Hexnit1_CE511491_1233_Hexagonia_nitida
denovo44937_vp1_Hexagonia
jgi_Hexnit1_1268007_gm1_3695_g_Hexagonia_nitida
denovo20418_vp1_Hexagonia
denovo270140_vp1_Hexagonia_nitida
jgi_Hexnit1_gm1_3705_g_Hexagonia_nitida
denovo185314_vp1_Hexagonia_nitida
jgi_Hexnit1_e_gw1_3_640_1_Hexagonia_nitida
denovo96837_vp1_Hexagonia
jgi_Hexnit1_gm1_3691_g_Hexagonia_nitida
jgi_Hexnit1_gm1_3690_g_Hexagonia_nitida
I97_denovo84554_vp1_Hexagonia
jgi_Hexnit1_estExt_Genemark1_C_3_t20461_Hexagonia_nitida
denovo234727_vp1_Hexagonia_nitida
jgi_Hexnit1_CE511466_486_Hexagonia_nitida
I97_denovo313085_vp1_Hexagonia_nitida
denovo294458_id94_vp1_Bjerkandera
jgi_Bjead1_1_gm1_8541_g_vp1_Bjerkandera_adusta
denovo314418_vp1_Fomitiporia_mediterranea
denovo112887_vp1_Fomitiporia_mediterranea
jgi_Panru1_1085109_CE1084844_522_Panus_rudis
jgi_Grifr1_mnp3_3-1_Grifola_frondosa
denovo4157_vp1_Grifola_frondosa
Barbi_HG799609_1_partial_mnP-2_strain_136_Grifola_frondosa
Barbi_HG799608_1_partial_mnP-1_strain_136_Grifola_frondosa
jgi_Grifr1_mnp3_1-1_Grifola_frondosa
jgi_Grifr1_mnp3_2-1_Grifola_frondosa
denovo269749_vp1_Grifola_frondosa
denovo122488_vp1_Grifola_frondosa
I97_denovo69922_vp1_Cerrena_unicolor
jgi_Cerun2_377554_fgenesh1_kg_6__229__Locus14627v1rpkm1_21_Cerrena_unicolor
jgi_Cerun2_11174_CE10958_231_Cerrena_unicolor
jgi_Cerun2_357322_estExt_Genewise1Plus_C_230042_Cerrena_unicolor
I97_denovo228252_vp1_Cerrena_unicolor
jgi_Cerun2_estExt_Genewise1Plus_C_230046_vp1_Cerrena_unicolor
denovo236038_vp1_Cerrena_unicolor
jgi_Cerun2_gm1_10526_g_vp1_Cerrena_unicolor
denovo148765_vp1_Cerrena_unicolor
jgi_Cerun2_fgenesh1_kg_16___71___Locus10716v2rpkm0_81_vp1_Cerrena_unicolor
denovo143540_vp1_Cerrena_unicolor
jgi_Cerun2_fgenesh1_kg_90___6___Locus10716v2rpkm0_81_vp1_Cerrena_unicolor
jgi_Cerun2_fgenesh1_kg_90___8___Locus10716v2rpkm0_81_vp1_Cerrena_unicolor
jgi_Cerun2_gm1_10580_g_vp1_Cerrena_unicolor
denovo162571_vp1_Cerrena_unicolor
jgi_Crison1_999838_fgenesh1_pm_10__47_Cristinia_soronae
jgi_Crison1_913654_fgenesh1_kg_1__1584__TRINITY_DN4761_c1_g4_i5_Cristinia_soronae
jgi_Crison1_24190_CE23875_496_Cristinia_soronae
0.3
0
0.5
1
1.5
2
2.5

(B)

Figure S7. Phylogenetic placemement of the predicted protein sequences of MnP peroxidases amplified from soil samples of both forests (blue print) in comparison with known sequences from published genomes of Basidiomycetes. [horizontal bar = mean length between branches; vertical axis (coloured boxes) = bootstrap suppor]). The tree was built on RAxML and GTR+GAMMA model with 1000 bootstrap replications.

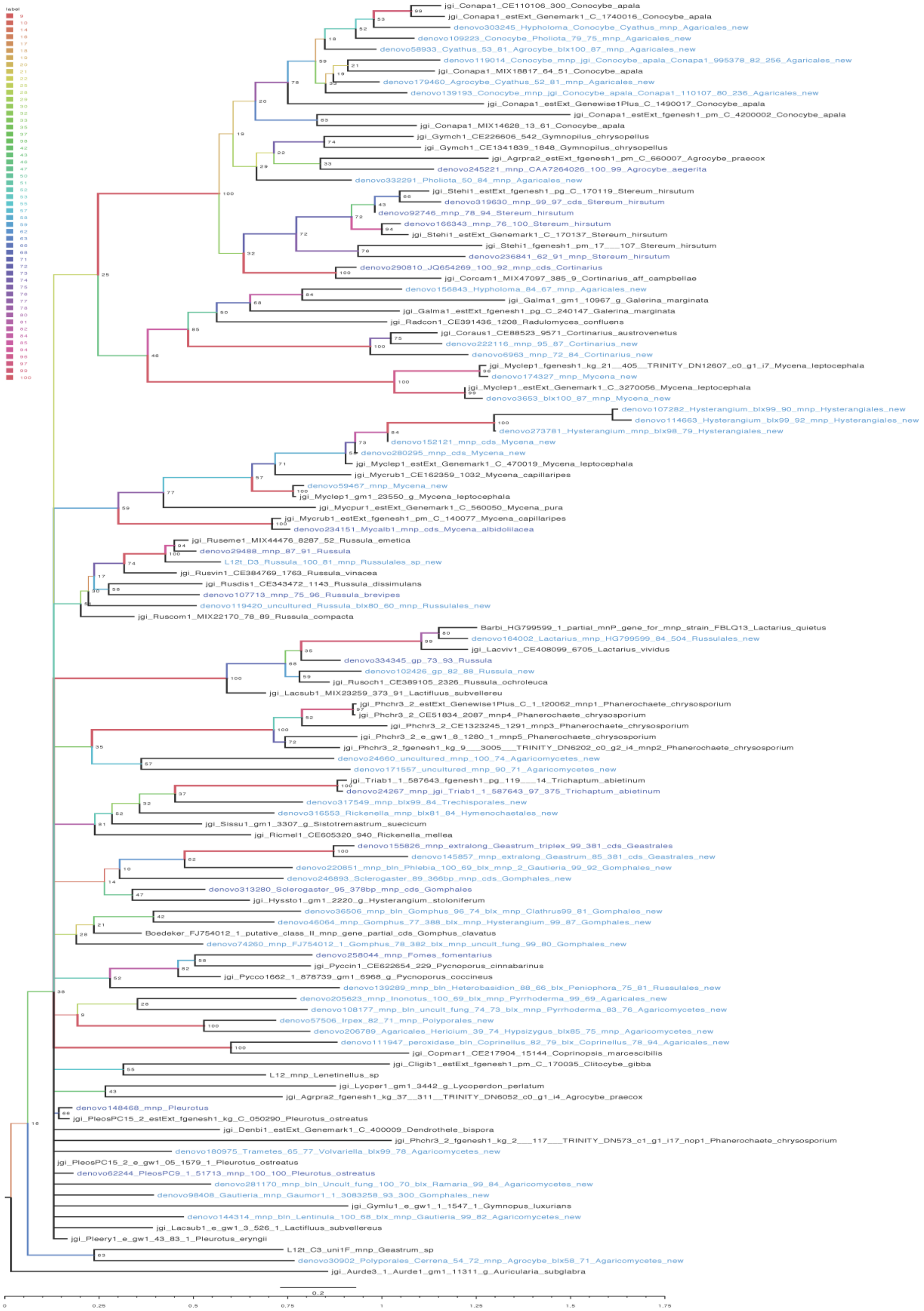

Supplement: Supplementary file 1 [file Data_Sheet_1.PDF]
