# Supplementary material for: Evaluation of the lignocellulose degradation potential of Mediterranean forests soil microbial communities through diversity and targeted functional metagenomics
Source: Front Microbiol. 2023 Feb 27;14:1121993. doi: 10.3389/fmicb.2023.1121993 (PMC10008878; doi:10.3389/fmicb.2023.1121993)
Supplement: Supplementary file 2 [file Data_Sheet_2.PDF]

Supplementary Material

Article Title

Evaluation of the lignocellulose degradation potential of Mediterranean forests soil microbial communities through diversity and targeted functional metagenomics

Maria Kalntremtziou, Ioannis A. Papaioannou, Vasileios Vangalis, Elias Polemis, Katherine M. Pappas, Georgios I. Zervakis, Milton A. Typas*

Corresponding Author
matypas@biol.uoa.gr

Table S1. Primers used in this work; the strategy followed for their construction (and the anticipated amplicons) is explained in the Results section and presented in Figure 3.

**Primer name**	**Sequence 5' - 3'**	**Origin**
341F	CCTACGGGNGGCWGCAG	MR DNA, Shallowater, TX, USA
805R	GACTACNVGGGTATCTAATCC	MR DNA, Shallowater, TX, USA
ITS3F	GCATCGATGAAGAACGCAGC	MR DNA, Shallowater, TX, USA
ITS4R	TCCTCCGCTTATTGATATGC	White et al. 1990
ITS7A	GTGAATCATCGAATCTTTG	White et al. 1990
1MF	GACGAYATCCAGMAGAACCT	This work
1MR	TGARCCTCGABGAAGAHCTG	This work
uni1MF	AGNGGYGCWGAYGGYTC	This work
agarF	TGGNGGTGCTGATGGTTC	This work
mnpun1F	GGAGGTGCGGAYGGYTC	This work
Perox2F	CBTTCCAYGAYGCNATYG	This work
Perox3aF	TTCCAYGAYGCNATTGGNTTYTC	This work
Perox1aF	GGYGGNGGNGCNGAYGGNTC	This work
Perox1bF	GGHGGAGGHGCNGAYGG	This work
Perox1R	GGNGTNGARTCGAADGG	This work
Perox3R	GNGTCTCRAYGAAGAHCTG	This work
Perox3bF	TGCTGYGCNCTDTTCCCNGT	This work
Perox2R	GTCGGGAGGGTCGGGAAGGG	This work

Table S2. *Basidiomycota* specimens used to test for amplification of peroxidase-like sequences in order to construct the best pair of primers ("universal") for the metagenomic search. The species of basidiomycetes with publicly available genomes used for the construction of the BasK mock community are indicated with bold letters.
LGAM= The Culture Collection of the Laboratory of General and Agricultural Microbiology, Agricultural University of Athens, Greece; ATCC= American Type Culture Collection, Maryland, USA; CBS= CBS-KNAW, Westerdijk Fungal Biodiversity Institute, The Netherlands;HMAS, Mycological Herbarium, Institute of Microbiology,Academia Sinica, Beijing, China; ATHUM= Mycetotheca, Faculty of Biology, National and Kapodistrian University of Athens, Greece. Material with codes starting from "ACAM", "EP" and "DD" represent fungarium specimens deposited at ACAM (Agricultural University of Athens, Laboratory of General and Agricultural Microbiology; http://sweetgum.nybg.org/science/ih/herbarium_details.php?irn=225631).

	**Order**	**Family**	**Species**	**Code and geographic origin (Greece, if no country is stated) when available**
1	Agaricales	Agaricaceae	*Agaricus augustus*	EP.12-A1166, Mt. Parnitha
2	Agaricales	Agaricaceae	*Leucoagaricus* sp.	ACAM 2012-219, Andros
3	Agaricales	Amanitaceae	*Amanita spissa / fruceti*	EP1, Mt. Parnitha
4	Agaricales	Strophariaceae	*Cyclocybe cylindracea*	LGAM 172
5	Agaricales	Fistulinaceae	*Fistulina hepatica*	LGAM 530
6	Agaricales	Inocybaceae	*Inocybe* sp.	ACAM 2015-073, Mt. Parnitha
7	Agaricales	Lyophyllaceae	*Hypsizygus ulmarius*	DD2690, Fthiotida
8	Agaricales	Mycenaceae	*Mycena* sp.	ACAM 2012-192, Mt. Parnitha
9	Agaricales	Mycenaceae	*Mycena* sp.	ACAM 2012-198, Mt. Parnitha
10	Agaricales	Omphalotaceae	*Lentinula edodes*	LGAM 889
11	Agaricales	Omphalotaceae	*Omphalotus illudens*	LGAM 347, Kiato
12	Agaricales	Physalacriaceae	*Armillaria mellea*	EP.12-K189, Ikaria
13	Agaricales	Physalacriaceae	*Armillaria ostoye*	LGAM 338 , Mt Dirfi
14	Agaricales	Physalacriaceae	*Armillaria tabescens*	LGAM 288, Gardiki
15	Agaricales	Physalacriaceae	*Flammulina velutipes*	LGAM 803
16	Agaricales	Pleurotaceae	*Hohenbuehelia petaloides*	ACAM 2012-0089, Mt. Parnitha
17	Agaricales	Pleurotaceae	***Pleurotus ostreatus***	CBS37551
18	Agaricales	Pleurotaceae	*Pleurotus cystidiosus*	LGAM206
19	Agaricales	Pleurotaceae	*Pleurotus australis*	LGAM D2245.11
20	Agaricales	Pleurotaceae	*Pleurotus djamor*	LGAM 812
21	Agaricales	Pleurotaceae	*Pleurotus dryinus*	CBS44977
22	Agaricales	Pleurotaceae	*Pleurotus eryngii*	CBS10082
23	Agaricales	Pleurotaceae	*Pleurotus tuoliensis*	HMAS 86357
24	Agaricales	Pleurotaceae	*Pleurotus pulmonarius*	ATCC36050
25	Agaricales	Pleurotaceae	*Pleurotus pulmonarius*	LGAM851003

26	Agaricales	Pleurotaceae	*Pleurotus tuberregium*	LGAM 823
27	Agaricales	Pleurotaceae	*Pleurotus citrinopileatus*	LGAM 158
28	Agaricales	Pluteaceae	*Volvariella bombycina*	DD2906, Mt Oxya
29	Agaricales	Psathyrellaceae	*Coprinopsis romagnesiana*	EP14-A1268, Andros
30	Agaricales	Schizophyllaceae	*Schizophyllum communae*	DD791, Fthiotida
31	Agaricales	Strophariaceae	*Cyclocybe cylindracea*	LGAM 493
32	Agaricales	Strophariaceae	*Galerina marginata*	ACAM 2014-009, Mt. Parnitha
33	Agaricales	Strophariaceae	*Galerina marginata*	ACAM 2012-191, Mt. Parnitha
34	Agaricales	Strophariaceae	*Hypholoma fasciculare*	EP13-A1194, Andros
35	Agaricales	Strophariaceae	*Hypholoma sublateritium*	ATHUM
36	Agaricales	Strophariaceae	*Pholiota squarrosa*	ATHUM 3144
37	Agaricales	Tricholomataceae	*Clitocybe squamulosa*	EP.12-A1151, Andros
38	Agaricales	Tricholomataceae	*Hemimycena cephalotricha*	EP.12-K156, Ikaria
39	Agaricales	Tricholomataceae	*Tricholomopsis* sp. (aff. *rutilans*)	ACAM 2012-083, Mt. Parnitha
40	Agaricales	Tricholomataceae	*Laccaria* cf.*laccata*	ACAM 2012-247, Lasithi
41	Agaricales	Tricholomataceae	*Macrocystidia cucumis*	ACAM 2611/28 2021-217
42	Auriculariales	Auriculariaceae	*Auricularia mesenterica*	LGAM 472
43	Auriculariales	Auriculariaceae	***Auricularia auricula-judae***	LGAM 468
44	Boletales	Boletaceae	*Xerocomellus chrysenteron*	ACAM 2015-072, Mt. Parnitha
45	Boletales	Boletaceae	*Xerocomellus* sp.	EP8, Mt. Parnitha
46	Boletales	Boletaceae	*Boletus* sp.	EP2, Mt. Parnitha
47	Boletales	Coniophoraceae	*Coniophora puteana*	EP.14-A1260, Andros
48	Boletales	Pisolithaceae	*Pisolithus arrhizus*	ACAM 2014-76,Messinia
49	Boletales	Sclerodermataceae	*Scleroderma verrucosum*	ACAM 2014-77, Messinia
50	Boletales	Suillaceae	*Suillus bellinii*	ACAM2012-188,Attica
51	Canthalellales	Botryobasidiaceae	*Botryobasidium* sp.	EP3, Mt. Parnitha
52	Dacrymycetales	Dacrymycetaceae	*Calocera furcata*	ACAM 2012-0205,Mt. Parnitha
53	Geastrales	Geastraceae	***Geastrum fimbriatum (=sessile)***	ACAM 2012-213, Mt. Parnitha
54	Gomphales	Gomphaceae	*Ramaria sp.*	ACAM 2014-023, Ileia
55	Gloeophyllales	Gloeophyllaceae	*Gloeophyllum odoratum*	LGAM 482, Mt. Parnitha

56	Hymenochaetales	Hymenochaetaceae	*Phellinus rimosus*	LGAM 520
57	Hymenochaetales	Hymenochaetaceae	*Phellinus torulosus*	LGAM 414, Gardiki
58	Hymenochaetales	Hymenochaetaceae	***Fomitiporia mediterranea***	LGAM 297, Argoliko
59	Hymenochaetales	Hymenochaetaceae	***Phellinus punctatus***	LGAM 662
60	Hymenochaetales	Hymenochaetaceae	*Inonotus hispidus*	LGAM 348, Crete
61	Hymenochaetales	Hymenochaetaceae	*Inonotus hispidus*	LGAM 311, Attica
62	Hymenochaetales	Hymenochaetaceae	*Inonotus levis*	LGAM 450, Athens
63	Hymenochaetales	Hymenochaetaceae	*Inonotus nodulosus*	LGAM 556, Czech Republic
64	Hymenochaetales	Hymenochaetaceae	*Inonotus tamaricis*	LGAM 524
65	Hymenochaetales	Hymenochaetaceae	*Phylloporia ribis*	ACAM 2014-062, Mt. Parnitha
66	Hymenochaetales	Insertae sedis	*Trichaptum* sp.	ACAM 2012-0201, Mt. Parnitha
67	Hymenochaetales	Schizoporaceae	*Schizopora radula*	EP.13-A1206, Andros
68	Polyporales	Amylocorticiaceae	*Ceraceomyces tessulatus*	ACAM 2012-0208, Mt. Parnitha
69	Polyporales	Cerrenaceae	*Cerrena unicolor*	ATHUM
70	Polyporales	Dacryobolaceae	*Postia caesia*	LGAM 523
71	Polyporales	Fomitopsidaceae	*Fomitopsis pinicola*	ACAM 2012-0133, Mt. Taygetos
72	Polyporales	Fomitopsidaceae	*Daedelea quercina*	LGAM 433, Drama
73	Polyporales	Ganodermataceae	*Ganoderma adspersum*	LGAM 487
74	Polyporales	Ganodermataceae	*Ganoderma applanatum*	LGAM564 & ACAM2015_0057, Mt. Oxya
75	Polyporales	Ganodermataceae	***Ganoderma carnosum***	LGAM 305 & DD1243, Mt Oxya
76	Polyporales	Ganodermataceae	*Ganoderma lingzhi*	LGAM 803
77	Polyporales	Ganodermataceae	***Ganoderma lucidum***	LGAM490
78	Polyporales	Ganodermataceae	*Ganoderma pfeifferii*	LGAM 336 & DD2118, Mt. Oxya
79	Polyporales	Ganodermataceae	*Ganoderma resinaceum*	LGAM486 & ACAM2013-0013,Attica
80	Polyporales	Grifolaceae	*Grifola frondosa*	LGAM 494
81	Polyporales	Incrustoporiaceae	*Tyromyces lacteus*	LGAM616, Czech Republic
82	Polyporales	Irpicaceae	***Irpex lacteus***	LGAM 238,, Czech Republic
83	Polyporales	Irpicaceae	*Gloeoporus pannocinctus*	ACAM 2012-300, Lasithi
84	Polyporales	Ischnodermataceae	*Ischnoderma benzoinum*	LGAM 521
85	Polyporales	Meripilaceae	*Meripilus giganteus*	LGAM 335, Mt Oxya
86	Polyporales	Meruliaceae	***Phlebia tremelloidea***	LGAM 477
87	Polyporales	Meruliaceae	*Phlebia unica*	EP.12-K181, Ikaria

88	Polyporales	Meruliaceae	*Meripilus giganteus*	LGAM 335, Mt Oxya
89	Polyporales	Phanerochaetaceae	***Phanerochaete chrysosporium***	LGAM 322
90	Polyporales	Phanerochetacea	*Phanerochaete velutina*	ACAM 2012-129, Mt. Taygetos
91	Polyporales	Phanerochetacea	*Hapalopilus croceus*	LGAM 922
92	Polyporales	Phanerochaetaceae	*Fistulina hepatica*	EP0411Fh
93	Polyporales	Podoscyphaceae	*Abortiporus biennis*	LGAM 436, Lesvos
94	Polyporales	Polyporaceae	*Coriolopsis gallica*	LGAM 519
95	Polyporales	Polyporaceae	*Trametes versicolor*	EP.12-A1140, Andros
96	Polyporales	Polyporaceae	*Dichomitus squalens*	LGAM 342, Olympia
97	Polyporales	Polyporaceae	*Fomes fomentarius*	LGAM 296, Aliatros
98	Polyporales	Polyporaceae	*Hexagonia nitida*	LGAM 467
99	Polyporales	Polyporaceae	*Lentinus tigrinus*	DD4486, Lesvos
100	Polyporales	Polyporaceae	*Perenniporia fraxinea*	LGAM 346, Loutra Ypatis
101	Polyporales	Polyporaceae	*Podofomes trogii*	ACAM 2015-0071, Mt. Parnitha
102	Polyporales	Polyporaceae	*Polyporus ciliatus*	LGAM 992
103	Polyporales	Polyporaceae	*Polyporus lentus*	LGAM 890
104	Polyporales	Polyporaceae	*Pycnoporus cinnabarinus*	DD283, Ioannina
105	Polyporales	Polyporaceae	*Skeletocutis percandida*	EP.12-K194,Ikaria
106	Polyporales	Polyporaceae	*Trametes hirsuta*	LGAM 434, Drama
107	Polyporales	Polyporaceae	*Daedalea quercina*	LGAM 528, Czech Republic
108	Polyporales	Polyporaceae	*Trametes versicolor*	LGAM 614, Czech Republic
109	Polyporales	Polyporaceae	*Trametes suaveolens*	LGAM 611, Czech Republic
110	Polyporales	insertae sedis	*Spongipellis delectans*	LGAM 981, Czech Republic
111	Russulales	Auriscalpiaceae	*Lentinellus ursinus*	ATHUM
112	Russulales	Russulaceae	*Russula* sp.	EP6, Parnitha
113	Russulales	Stereaceae	*Stereum hirsutum*	LGAM 608, Czech Republic
114	Russulales	Stereaceae	*Stereum hirsutum*	ACAM 2012-254, Lasithi
115	Russulales	Stereaceae	*Stereum gausapatum*	ACAM 2012-277, Lasithi
116	Sebacinales	Sebacinaceae	*Sebacina (Exidiopsis) grisea*	ACAM 2014-0010, Mt. Parnitha
117	Thelephorales	Thelephoraceae	*Thelephora*	EP.13-A1225, Andros
118	Thelephorales	Thelephoraceae	*Thelephora caryophyllea*	DD1359, Fthiotida
119	Thelephorales	Thelephoraceae	*Tomentella*	ACAM 2014-0008, Mt. Parnitha
120	Trechisporales	Trechisporaceae	*Xenasmatella vaga*	EP.12-K200, Ikaria

Table S3. Reads, OTUs and genera of bacteria and fungi found in the forests of Parnitha (P) and Andros (A). Numbers 1,2,3,4 designate the four sampling plots. PI, AI and PII, AII designate the winter and summer period samples, respectively. Letters a,b,c next to PI,PII, AI, AII refer to the soil depths from which the samples were collected.

Bacteria

		**1**			**2**			**3**			**4**	
	PI1a	PI1b	PI1c	PI2a	PI2b	PI2c	P13a	PI3b	PI3c	PI4a	PI4b	PI4c
OTUs	13278	17723	18588	25186	22636	21032	23493	21313	21814	21042	17513	17603
Genera	494	509	486	578	542	508	557	543	529	537	495	480
Reads	34316	42850	48646	58103	58955	60995	57891	49834	58286	47219	38920	44639

	AI1a	AI1b	AI1c	AI2a	AI2b	AI2c	AI3a	AI3b	AI3c	AI4a	AI4b	AI4c
OTUs	31311	29708	37836	24666	35034	24127	25883	37761	20404	29308	31339	25323
Genera	530	512	568	580	549	575	551	571	495	572	554	514
Reads	66432	61192	92026	70214	83374	68022	56809	77819	45069	63310	76881	54794

		**1**			**2**			**3**			**4**	
	PII1a	PII1b	PII1c	PII2a	PII2b	PII2c	PII3a	PII3b	PII3c	PII4a	PII4b	PII4c
OTUs	20256	22229	14193	20272	14463	14760	16183	16920	18713	21416	21138	19862
Genera	507	524	463	532	475	463	502	554	503	549	493	491
Reads	58127	49926	36163	48913	32075	37751	37742	33788	43735	60975	45509	44378

	AII1a	AII1b	AII1c	AII2a	AII2b	AII2c	AII3a	AII3b	AII3c	AII4a	AII4b	AII4c
OTUs	75743	23883	24841	21020	18236	23803	26921	21876	20681	24771	25570	23465
Genera	724	555	562	580	549	539	610	568	542	554	534	531
Reads	236270	52072	54204	54510	38291	50184	57752	44108	45488	51063	52866	54200

Fungi

		**1**			**2**			**3**			**4**	
	PI1a	PI1b	PI1c	PI2a	PI2b	PI2c	PI3a	PI3b	PI3c	PI4a	PI4b	PI4c
OTUs	21115	11047	4774	12868	9334	8351	9718	10200	4778	15656	8586	5642
Genera	494	379	269	433	368	345	400	366	292	472	369	326
Reads	195768	338783	420770	236622	139840	257738	177225	155798	194265	180074	178385	346060

	AI1a	AI1b	AI1c	AI2a	AI2b	AI2c	AI3a	AI3b	AI3c	AI4a	AI4b	AI4c
OTUs	6474	4281	7861	1764	2988	2791	4563	4798	8032	6290	15932	4741
Genera	204	172	211	146	168	180	192	223	201	278	317	160
Reads	112361	32085	87790	10917	46429	179652	89315	69368	144791	171909	188149	81521

		**1**			**2**			**3**			**4**	
	PII1a	PII1b	PII1c	PII2a	PII2b	PII2c	PII3a	PII3b	PII3c	PII4a	PII4b	PII4c
OTUs	10122	6415	8636	5676	8754	4619	12371	2353	3773	15051	7465	5584
Genera	377	330	372	312	383	271	444	151	267	473	359	308
Reads	210964	302367	659441	128430	296282	363452	124270	47683	156683	193856	102861	260575

	AII1a	AII1b	AII1c	AII2a	AII2b	AII2c	AII3a	AII3b	AII3c	AII4a	AII4b	AII4c
OTUs	11253	11497	7053	10812	8653	8971	11232	5448	8614	9515	8135	8369
Genera	441	423	346	438	380	390	428	303	371	401	369	378
Reads	218390	228913	210584	187439	165704	281623	203607	156717	176820	174760	123614	202957

Table S6. Distribution of OTUs obtained with the peroxF/R primers (simple and nested reactions) from the four plots (1,2,3,4), two seasons (PI,AI = winter, PII,AII= summer), depths (a, bc) of Parnitha (P) and Andros (A) forests.

	**PI1a**	**PI1bc**	**PI2a**	**PI2bc**	**PI3a**	**PI3bc**	**PI4a**	**PI4bc**
reads	28476	31101	22219	38663	27168	26308	34633	39158
OTUs	11431	9367	10037	10097	12440	10282	16038	12163

	**PII1a**	**PII1bc**	**PII2a**	**PII2bc**	**PII3a**	**PII3bc**	**PII4a**	**PII4bc**
reads	35391	51564	31328	68541	26032	35368	31239	45746
OTUs	7420	9757	7831	11544	12637	11602	11113	11718

	**AI1a**	**AI1bc**	**AI2a**	**AI2bc**	**AI3a**	**AI3bc**	**AI4a**	**AI4bc**
reads	17950	25309	26650	56677	51736	23746	27587	23526
OTUs	8502	8823	8898	10826	12002	12379	9494	7632

	**AII1a**	**AII1bc**	**AII2a**	**AII2bc**	**AII3a**	**AII3bc**	**AII4a**	**AII4bc**
reads	33048	30010	31342	27883	16325	24041	30210	23879
OTUs	9967	21143	9087	12330	8091	9367	8587	8569

**nested**	**PI1c**	**PI3c**	**PII1c**	**PII2c**	**AI2a**	**AI2c**	**AII2a**	**AII2c**
reads	162971	96932	126178	110633	16268	66279	85125	41297
OTUs	12530	8903	8814	9842	7463	18594	9567	5743
