# Supplementary material for: Evaluation of the lignocellulose degradation potential of Mediterranean forests soil microbial communities through diversity and targeted functional metagenomics
Source: Front Microbiol. 2023 Feb 27;14:1121993. doi: 10.3389/fmicb.2023.1121993 (PMC10008878; doi:10.3389/fmicb.2023.1121993)
Supplement: Supplementary file 3 [file Data_Sheet_3.PDF]

Table S4. The 200 most abundant genera of bacteria in Andros forest; **(A)** horizon a , **(B)** horizon b, **(C)** horizon c, in Parnitha forest; **(D)** horizon a, **(E)** horizon b, **(F)** horizon c.

(A)

AI1a	AI2a	AI3a	AI4a	AII1a	AII2a	AII3a	AII4a	#OTU ID	
18,90%	11,50%	15,19%	16,29%	17,87%	12,75%	18,25%	18,09%	Unassigned;Other;Other;Other;Other;Other	
4,69%	1,97%	1,95%	2,78%	3,57%	1,47%	1,71%	1,34%	D_0__Bacteria;D_1__Planctomycetes;D_2__Phycisphaerae;D_3__Tepidisphaerales;D_4__WD2101 soil group	uncultured bacterium
2,77%	3,15%	3,48%	2,75%	1,42%	1,04%	1,22%	1,81%	D_0__Bacteria;D_1__Proteobacteria;D_2__Alphaproteobacteria;D_3__Sphingomonadales;D_4__Sphingomonadaceae	Sphingomonas
2,98%	1,64%	2,93%	2,59%	2,42%	1,29%	1,98%	1,47%	D_0__Bacteria;D_1__Proteobacteria;D_2__Alphaproteobacteria;D_3__Rhizobiales;D_4__Xanthobacteraceae	uncultured
2,16%	1,85%	1,91%	1,86%	1,80%	2,17%	1,76%	1,52%	D_0__Bacteria;D_1__Bacteroidetes;D_2__Bacteroidia;D_3__Chitinophagales;D_4__Chitinophagaceae	uncultured
1,65%	1,18%	1,27%	1,36%	2,17%	2,01%	2,62%	2,92%	D_0__Bacteria;D_1__Acidobacteria;D_2__Subgroup 6;D_3__uncultured bacterium;D_4__	
1,53%	1,07%	1,23%	1,30%	1,62%	2,53%	2,41%	2,77%	D_0__Bacteria;D_1__Acidobacteria;D_2__Subgroup 6;D_3__uncultured Acidobacteria bacterium;D_4__	
0,89%	1,41%	3,41%	1,22%	1,37%	2,06%	0,97%	1,35%	D_0__Bacteria;D_1__Patescibacteria;D_2__Saccharimonadia;D_3__Saccharimonadales;D_4__uncultured bacterium	
1,16%	0,95%	3,01%	2,10%	0,95%	1,09%	1,17%	0,96%	D_0__Bacteria;D_1__Proteobacteria;D_2__Alphaproteobacteria;D_3__Micropepsales;D_4__Micropepsaceae	uncultured
1,50%	1,16%	2,26%	2,08%	1,19%	0,72%	1,37%	1,06%	D_0__Bacteria;D_1__Proteobacteria;D_2__Alphaproteobacteria;D_3__Rhizobiales;D_4__Xanthobacteraceae	Bradyrhizobium
0,23%	0,67%	0,28%	0,45%	1,61%	4,35%	1,98%	2,75%	D_0__Bacteria;D_1__Bacteroidetes;D_2__Bacteroidia;D_3__Cytophagales;D_4__Microscillaceae	uncultured
2,13%	2,81%	1,70%	1,04%	0,98%	1,35%	0,62%	0,61%	D_0__Bacteria;D_1__Actinobacteria;D_2__Actinobacteria;D_3__Propionibacteriales;D_4__Nocardioidaceae	Nocardioides
1,45%	0,24%	0,40%	1,62%	2,52%	0,77%	1,11%	2,38%	D_0__Bacteria;D_1__Verrucomicrobia;D_2__Verrucomicrobiae;D_3__Chthoniobacterales;D_4__Chthoniobacteraceae	Candidatus Udaeobacter
2,21%	1,27%	1,06%	1,05%	1,20%	1,40%	0,93%	0,73%	D_0__Bacteria;D_1__Actinobacteria;D_2__Thermoleophilia;D_3__Solirubrobacterales;D_4__67-14	uncultured bacterium
1,42%	1,35%	1,34%	1,33%	1,21%	0,82%	0,77%	0,79%	D_0__Bacteria;D_1__Proteobacteria;D_2__Alphaproteobacteria;D_3__Sphingomonadales;D_4__Sphingomonadaceae	uncultured
0,84%	0,70%	0,67%	0,79%	1,05%	1,31%	1,46%	1,71%	D_0__Bacteria;D_1__Acidobacteria;D_2__Subgroup 6;Other;Other;Other	
0,31%	3,91%	0,28%	0,17%	0,68%	2,91%	0,54%	0,29%	D_0__Bacteria;D_1__Firmicutes;D_2__Bacilli;D_3__Bacillales;D_4__Bacillaceae	Bacillus
1,17%	0,91%	0,80%	0,61%	0,97%	0,80%	1,03%	1,31%	D_0__Bacteria;D_1__Proteobacteria;D_2__Gammaproteobacteria;D_3__Betaproteobacteriales;D_4__Nitrosomonadaceae	Ellin6067
1,01%	1,77%	0,87%	0,71%	0,64%	1,12%	0,69%	0,53%	D_0__Bacteria;D_1__Bacteroidetes;D_2__Bacteroidia;D_3__Chitinophagales;D_4__Chitinophagaceae	Ferruginibacter
0,64%	0,88%	0,87%	1,22%	0,78%	0,77%	0,85%	0,79%	D_0__Bacteria;D_1__Acidobacteria;D_2__Acidobacteriia;D_3__Solibacterales;D_4__Solibacteraceae (Subgroup 3)	Bryobacter
0,44%	0,57%	0,49%	0,82%	1,16%	1,32%	1,10%	1,22%	D_0__Bacteria;D_1__Verrucomicrobia;D_2__Verrucomicrobiae;D_3__Chthoniobacterales;D_4__Chthoniobacteraceae	Chthoniobacter
2,40%	1,52%	0,91%	0,67%	0,51%	0,42%	0,29%	0,26%	D_0__Bacteria;D_1__Actinobacteria;D_2__Thermoleophilia;D_3__Solirubrobacterales;D_4__Solirubrobacteraceae	Solirubrobacter
0,57%	0,38%	0,92%	0,94%	0,93%	0,82%	1,03%	1,11%	D_0__Bacteria;D_1__Proteobacteria;D_2__Alphaproteobacteria;D_3__Reyranellales;D_4__Reyranellaceae	Reyranella
1,02%	1,30%	0,93%	0,87%	0,46%	0,84%	0,41%	0,62%	D_0__Bacteria;D_1__Actinobacteria;D_2__Actinobacteria;D_3__Corynebacteriales;D_4__Mycobacteriaceae	Mycobacterium
0,10%	1,79%	0,51%	1,04%	0,18%	1,71%	0,40%	0,43%	D_0__Bacteria;D_1__Bacteroidetes;D_2__Bacteroidia;D_3__Flavobacteriales;D_4__Flavobacteriaceae	Flavobacterium
0,43%	0,68%	1,09%	0,93%	0,63%	0,67%	0,62%	0,92%	D_0__Bacteria;D_1__Proteobacteria;D_2__Alphaproteobacteria;D_3__Caulobacterales;D_4__Caulobacteraceae	Phenylobacterium
1,46%	0,67%	0,79%	0,54%	0,91%	0,54%	0,65%	0,73%	D_0__Bacteria;D_1__Chloroflexi;D_2__KD4-96;D_3__uncultured bacterium;D_4__	
0,37%	0,32%	1,02%	0,99%	0,45%	0,53%	1,08%	1,07%	D_0__Bacteria;D_1__Proteobacteria;D_2__Alphaproteobacteria;D_3__Dongiales;D_4__Dongiaceae	Dongia
0,38%	1,40%	0,62%	0,67%	0,27%	1,03%	0,83%	0,56%	D_0__Bacteria;D_1__Actinobacteria;D_2__Actinobacteria;D_3__Streptomycetales;D_4__Streptomycetaceae	Streptomyces
1,29%	0,56%	0,69%	0,61%	1,01%	0,53%	0,63%	0,48%	D_0__Bacteria;D_1__Actinobacteria;D_2__Acidimicrobiia;D_3__IMCC26256;D_4__uncultured bacterium	
0,70%	0,74%	0,39%	0,47%	0,74%	1,15%	0,91%	0,73%	D_0__Bacteria;D_1__Bacteroidetes;D_2__Bacteroidia;D_3__Chitinophagales;D_4__Chitinophagaceae	Terrimonas
0,63%	0,27%	0,36%	0,32%	1,47%	0,84%	0,93%	1,12%	D_0__Bacteria;D_1__Proteobacteria;D_2__Deltaproteobacteria;D_3__Myxococcales;D_4__Haliangiaceae	Haliangium
0,58%	0,22%	0,42%	0,73%	1,15%	0,49%	0,93%	0,84%	D_0__Bacteria;D_1__Acidobacteria;D_2__Acidobacteriia;D_3__Solibacterales;D_4__Solibacteraceae (Subgroup 3)	Candidatus Solibacter
1,53%	0,47%	0,68%	0,49%	0,86%	0,31%	0,52%	0,36%	D_0__Bacteria;D_1__Actinobacteria;D_2__Thermoleophilia;D_3__Gaiellales;D_4__uncultured	uncultured bacterium
0,42%	0,26%	0,55%	0,73%	0,45%	0,58%	0,80%	1,12%	D_0__Bacteria;D_1__Proteobacteria;D_2__Gammaproteobacteria;D_3__Gammaproteobacteria Incertae Sedis;D_4__Unknown Family	Acidibacter
0,55%	0,48%	0,96%	0,90%	0,43%	0,34%	0,41%	0,46%	D_0__Bacteria;D_1__Proteobacteria;D_2__Alphaproteobacteria;D_3__Rhizobiales;D_4__Devosiaceae	Devosia
0,86%	0,37%	0,31%	0,41%	0,91%	0,40%	0,65%	0,68%	D_0__Bacteria;D_1__Gemmatimonadetes;D_2__Gemmatimonadetes;D_3__Gemmatimonadales;D_4__Gemmatimonadaceae	uncultured
0,77%	0,54%	0,45%	0,60%	0,59%	0,23%	0,54%	0,58%	D_0__Bacteria;D_1__Proteobacteria;D_2__Gammaproteobacteria;D_3__Betaproteobacteriales;D_4__SC-I-84	uncultured bacterium
0,40%	0,66%	0,36%	0,49%	0,53%	0,55%	0,50%	0,86%	D_0__Bacteria;D_1__Proteobacteria;D_2__Alphaproteobacteria;D_3__Caulobacterales;D_4__Hyphomonadaceae	Hirschia
0,22%	0,38%	0,93%	0,75%	0,39%	0,32%	0,41%	0,41%	D_0__Bacteria;D_1__Proteobacteria;D_2__Alphaproteobacteria;D_3__Caulobacterales;D_4__Caulobacteraceae	uncultured
0,38%	0,30%	0,28%	0,38%	0,59%	0,52%	0,91%	0,79%	D_0__Bacteria;D_1__Proteobacteria;D_2__Gammaproteobacteria;D_3__Betaproteobacteriales;D_4__TRA3-20	uncultured bacterium
0,08%	0,31%	0,57%	1,11%	0,10%	0,55%	0,31%	0,35%	D_0__Bacteria;D_1__Bacteroidetes;D_2__Bacteroidia;D_3__Sphingobacteriales;D_4__Sphingobacteriaceae	Mucilaginibacter
0,03%	0,20%	0,08%	1,39%	0,05%	1,11%	0,09%	0,11%	D_0__Bacteria;D_1__Bacteroidetes;D_2__Bacteroidia;D_3__Sphingobacteriales;D_4__Sphingobacteriaceae	Pedobacter
0,32%	0,15%	0,18%	0,22%	0,68%	0,49%	1,02%	1,11%	D_0__Bacteria;D_1__Proteobacteria;D_2__Gammaproteobacteria;D_3__Betaproteobacteriales;D_4__Nitrosomonadaceae	MND1
0,26%	0,39%	0,67%	0,85%	0,28%	0,29%	0,33%	0,43%	D_0__Bacteria;D_1__Proteobacteria;D_2__Gammaproteobacteria;D_3__Betaproteobacteriales;D_4__Burkholderiaceae;Other	
0,15%	0,77%	0,74%	0,48%	0,25%	0,64%	0,37%	0,40%	D_0__Bacteria;D_1__Proteobacteria;D_2__Alphaproteobacteria;D_3__Sphingomonadales;D_4__Sphingomonadaceae	Novosphingobium
0,80%	0,10%	0,21%	0,13%	1,18%	0,29%	0,81%	0,55%	D_0__Bacteria;D_1__Acidobacteria;D_2__Blastocatellia (Subgroup 4);D_3__Pyrinomonadales;D_4__Pyrinomonadaceae	RB41
0,63%	0,36%	0,51%	0,51%	0,57%	0,28%	0,41%	0,38%	D_0__Bacteria;D_1__Proteobacteria;D_2__Alphaproteobacteria;D_3__Rhizobiales;D_4__Xanthobacteraceae	Rhodoplanes
0,65%	0,34%	0,28%	0,37%	0,69%	0,43%	0,42%	0,55%	D_0__Bacteria;D_1__Gemmatimonadetes;D_2__Gemmatimonadetes;D_3__Gemmatimonadales;D_4__Gemmatimonadaceae	Gemmatimonas
0,51%	0,86%	0,47%	0,48%	0,29%	0,35%	0,20%	0,44%	D_0__Bacteria;D_1__Proteobacteria;D_2__Alphaproteobacteria;D_3__Sphingomonadales;D_4__Sphingomonadaceae	Altererythrobacter
0,07%	0,08%	0,83%	0,86%	0,15%	0,16%	0,60%	0,42%	D_0__Bacteria;D_1__Proteobacteria;D_2__Gammaproteobacteria;D_3__Betaproteobacteriales;D_4__Burkholderiaceae	Burkholderia-Caballeronia-Paraburkholderia
0,31%	0,41%	0,32%	0,17%	0,64%	0,68%	0,71%	0,59%	D_0__Bacteria;D_1__Proteobacteria;D_2__Deltaproteobacteria;D_3__Myxococcales;D_4__Sandaracinaceae	uncultured
0,38%	0,36%	0,53%	0,68%	0,33%	0,14%	0,33%	0,25%	D_0__Bacteria;D_1__Proteobacteria;D_2__Alphaproteobacteria;D_3__Acetobacterales;D_4__Acetobacteraceae	uncultured
0,39%	0,74%	0,36%	0,22%	0,42%	0,59%	0,34%	0,38%	D_0__Bacteria;D_1__Actinobacteria;D_2__Acidimicrobiia;D_3__Microtrichales;D_4__Iamiaceae	Iamia
0,17%	0,07%	0,11%	0,12%	0,94%	0,46%	1,09%	0,55%	D_0__Bacteria;D_1__Verrucomicrobia;D_2__Verrucomicrobiae;D_3__Pedosphaerales;D_4__Pedosphaeraceae	uncultured bacterium
0,22%	0,59%	0,42%	0,39%	0,38%	0,35%	0,36%	0,53%	D_0__Bacteria;D_1__Proteobacteria;D_2__Gammaproteobacteria;D_3__Betaproteobacteriales;D_4__Burkholderiaceae	Rhizobacter
0,11%	0,38%	0,71%	0,72%	0,17%	0,25%	0,25%	0,32%	D_0__Bacteria;D_1__Proteobacteria;D_2__Alphaproteobacteria;D_3__Rhizobiales;D_4__Rhizobiaceae	Allorhizobium-Neorhizobium-Pararhizobium-Rhizobium
0,04%	0,31%	0,11%	0,69%	0,34%	0,42%	0,52%	0,50%	D_0__Bacteria;D_1__Proteobacteria;D_2__Gammaproteobacteria;D_3__Pseudomonadales;D_4__Pseudomonadaceae	Pseudomonas
0,61%	0,38%	0,44%	0,26%	0,54%	0,44%	0,37%	0,20%	D_0__Bacteria;D_1__Actinobacteria;D_2__Acidimicrobiia;D_3__Microtrichales;D_4__Ilumatobacteraceae	CL500-29 marine group
0,47%	0,42%	0,43%	0,64%	0,27%	0,16%	0,19%	0,25%	D_0__Bacteria;D_1__Proteobacteria;D_2__Alphaproteobacteria;D_3__Rhizobiales;D_4__Xanthobacteraceae	Pseudolabrys
0,96%	0,43%	0,46%	0,34%	0,28%	0,14%	0,23%	0,15%	D_0__Bacteria;D_1__Actinobacteria;D_2__Thermoleophilia;D_3__Solirubrobacterales;D_4__Solirubrobacteraceae	Conexibacter
0,34%	1,08%	0,26%	0,21%	0,25%	0,44%	0,22%	0,31%	D_0__Bacteria;D_1__Proteobacteria;D_2__Gammaproteobacteria;D_3__Xanthomonadales;D_4__Xanthomonadaceae	Arenimonas
0,21%	0,40%	0,38%	0,43%	0,19%	0,28%	0,39%	0,40%	D_0__Bacteria;D_1__Proteobacteria;D_2__Alphaproteobacteria;D_3__Rhizobiales;D_4__Rhizobiaceae	Mesorhizobium
0,44%	0,43%	0,25%	0,21%	0,57%	0,26%	0,45%	0,25%	D_0__Bacteria;D_1__Actinobacteria;D_2__Acidimicrobiia;D_3__Microtrichales;D_4__uncultured	uncultured bacterium
0,26%	0,24%	0,32%	0,36%	0,39%	0,26%	0,33%	0,54%	D_0__Bacteria;D_1__Proteobacteria;D_2__Alphaproteobacteria;D_3__Caulobacterales;D_4__Hyphomonadaceae	SWB02
0,36%	0,75%	0,32%	0,25%	0,22%	0,42%	0,15%	0,20%	D_0__Bacteria;D_1__Actinobacteria;D_2__Acidimicrobiia;D_3__Microtrichales;D_4__Ilumatobacteraceae	uncultured
0,11%	0,30%	0,42%	0,53%	0,16%	0,32%	0,23%	0,31%	D_0__Bacteria;D_1__Proteobacteria;D_2__Alphaproteobacteria;D_3__Caulobacterales;D_4__Caulobacteraceae	Caulobacter
0,17%	0,88%	0,11%	0,13%	0,14%	0,81%	0,11%	0,15%	D_0__Bacteria;D_1__Actinobacteria;D_2__Acidimicrobiia;D_3__Microtrichales;D_4__Ilumatobacteraceae	Ilumatobacter
0,17%	0,20%	0,41%	0,47%	0,18%	0,19%	0,26%	0,27%	D_0__Bacteria;D_1__Proteobacteria;D_2__Gammaproteobacteria;D_3__Betaproteobacteriales;D_4__Burkholderiaceae	uncultured
0,11%	0,47%	0,38%	0,36%	0,12%	0,32%	0,18%	0,29%	D_0__Bacteria;D_1__Proteobacteria;D_2__Gammaproteobacteria;D_3__Betaproteobacteriales;D_4__Burkholderiaceae	Variovorax
0,12%	0,85%	0,20%	0,23%	0,15%	0,51%	0,10%	0,18%	D_0__Bacteria;D_1__Bacteroidetes;D_2__Bacteroidia;D_3__Chitinophagales;D_4__Chitinophagaceae	Parafilimonas
0,50%	0,34%	0,33%	0,23%	0,31%	0,18%	0,24%	0,16%	D_0__Bacteria;D_1__Actinobacteria;D_2__Acidimicrobiia;D_3__IMCC26256;Other;Other	
0,57%	0,27%	0,26%	0,20%	0,32%	0,18%	0,28%	0,22%	D_0__Bacteria;D_1__Actinobacteria;D_2__Thermoleophilia;D_3__Gaiellales;D_4__Gaiellaceae	Gaiella

0,28%	0,18%	0,29%	0,32%	0,32%	0,10%	0,26%	0,30%	D_0__Bacteria;D_1__Proteobacteria;D_2__Alphaproteobacteria;D_3__Elsterales;D_4__uncultured	uncultured bacterium
0,40%	0,36%	0,21%	0,22%	0,32%	0,25%	0,19%	0,20%	D_0__Bacteria;D_1__Actinobacteria;D_2__Actinobacteria;D_3__Pseudonocardiales;D_4__Pseudonocardiaceae	Pseudonocardia
0,14%	0,14%	0,24%	0,29%	0,26%	0,19%	0,38%	0,43%	D_0__Bacteria;D_1__Proteobacteria;D_2__Gammaproteobacteria;D_3__Xanthomonadales;D_4__Rhodanobacteraceae	Dokdonella
0,03%	1,43%	0,01%	0,01%	0,02%	0,73%	0,02%	0,01%	D_0__Bacteria;D_1__Firmicutes;D_2__Bacilli;D_3__Bacillales;D_4__Planococcaceae	Lysinibacillus
0,12%	0,20%	0,53%	0,42%	0,13%	0,14%	0,13%	0,15%	D_0__Bacteria;D_1__Proteobacteria;D_2__Alphaproteobacteria;D_3__Sphingomonadales;D_4__Sphingomonadaceae	Sphingobium
0,13%	0,11%	0,16%	0,25%	0,17%	0,17%	0,47%	0,52%	D_0__Bacteria;D_1__Proteobacteria;D_2__Gammaproteobacteria;D_3__Betaproteobacteriales;D_4__Nitrosomonadaceae	IS-44
0,11%	0,11%	0,06%	0,23%	0,30%	0,35%	0,62%	0,21%	D_0__Bacteria;D_1__Acidobacteria;D_2__Thermoanaerobaculia;D_3__Thermoanaerobaculales;D_4__Thermoanaerobacula ceae	Subgroup 10
0,20%	0,10%	0,27%	0,35%	0,27%	0,14%	0,25%	0,24%	D_0__Bacteria;D_1__Proteobacteria;D_2__Alphaproteobacteria;D_3__Rhizobiales;D_4__Hyphomicrobiaceae	Hyphomicrobium
0,15%	0,27%	0,16%	0,33%	0,10%	0,24%	0,20%	0,30%	D_0__Bacteria;D_1__Actinobacteria;D_2__Actinobacteria;D_3__Propionibacteriales;D_4__Nocardioidaceae	Kribbella
0,13%	0,11%	0,11%	0,16%	0,28%	0,27%	0,41%	0,38%	D_0__Bacteria;D_1__Proteobacteria;D_2__Gammaproteobacteria;D_3__Steroidobacterales;D_4__Steroidobacteraceae	uncultured
0,11%	0,07%	0,27%	0,46%	0,15%	0,09%	0,13%	0,19%	D_0__Bacteria;D_1__Proteobacteria;D_2__Gammaproteobacteria;D_3__Xanthomonadales;D_4__Rhodanobacteraceae	Rhodanobacter
0,25%	0,12%	0,32%	0,25%	0,16%	0,08%	0,16%	0,14%	D_0__Bacteria;D_1__Proteobacteria;D_2__Alphaproteobacteria;D_3__Rhizobiales;D_4__Xanthobacteraceae;Other	
0,06%	0,15%	0,15%	0,19%	0,13%	0,25%	0,22%	0,32%	D_0__Bacteria;D_1__Proteobacteria;D_2__Gammaproteobacteria;D_3__Steroidobacterales;D_4__Steroidobacteraceae	Steroidobacter
0,28%	0,15%	0,19%	0,18%	0,24%	0,12%	0,16%	0,16%	D_0__Bacteria;D_1__Proteobacteria;D_2__Alphaproteobacteria;D_3__Rhizobiales;D_4__Hyphomicrobiaceae	Pedomicrobium
0,03%	0,08%	0,01%	0,04%	0,10%	0,60%	0,25%	0,49%	D_0__Bacteria;D_1__Bacteroidetes;D_2__Bacteroidia;D_3__Cytophagales;D_4__Microscillaceae	Ohtaekwangia
0,08%	0,12%	0,75%	0,12%	0,11%	0,15%	0,10%	0,10%	D_0__Bacteria;D_1__Patescibacteria;D_2__Saccharimonadia;D_3__Saccharimonadales;Other;Other	
0,09%	0,07%	0,05%	0,06%	0,31%	0,26%	0,42%	0,30%	D_0__Bacteria;D_1__Proteobacteria;D_2__Deltaproteobacteria;D_3__Myxococcales;D_4__BIrii41	uncultured bacterium
0,02%	0,04%	0,12%	0,57%	0,01%	0,05%	0,12%	0,09%	D_0__Bacteria;D_1__Proteobacteria;D_2__Gammaproteobacteria;D_3__Xanthomonadales;D_4__Rhodanobacteraceae	Luteibacter
0,25%	0,09%	0,11%	0,12%	0,32%	0,09%	0,22%	0,27%	D_0__Bacteria;D_1__Acidobacteria;D_2__Holophagae;D_3__Subgroup 7;D_4__uncultured bacterium	
0,15%	0,18%	0,11%	0,14%	0,20%	0,21%	0,22%	0,21%	D_0__Bacteria;D_1__Proteobacteria;D_2__Gammaproteobacteria;D_3__Betaproteobacteriales;D_4__TRA3-20	metagenome
0,12%	0,35%	0,32%	0,17%	0,07%	0,12%	0,10%	0,14%	D_0__Bacteria;D_1__Proteobacteria;D_2__Gammaproteobacteria;D_3__Betaproteobacteriales;D_4__Burkholderiaceae	Ramlibacter
0,21%	0,15%	0,26%	0,23%	0,13%	0,07%	0,14%	0,13%	D_0__Bacteria;D_1__Actinobacteria;D_2__Actinobacteria;D_3__Frankiales;D_4__Frankiaceae	Jatrophihabitans
0,21%	0,16%	0,21%	0,18%	0,23%	0,09%	0,14%	0,14%	D_0__Bacteria;D_1__Proteobacteria;D_2__Alphaproteobacteria;D_3__Rhizobiales;D_4__Rhizobiales Incertae Sedis	uncultured
0,16%	0,29%	0,24%	0,22%	0,09%	0,10%	0,08%	0,11%	D_0__Bacteria;D_1__Proteobacteria;D_2__Alphaproteobacteria;D_3__Sphingomonadales;D_4__Sphingomonadaceae;Other	
0,19%	0,54%	0,22%	0,13%	0,09%	0,10%	0,07%	0,05%	D_0__Bacteria;D_1__Actinobacteria;D_2__Actinobacteria;D_3__Frankiales;D_4__Nakamurellaceae	Nakamurella
0,09%	0,23%	0,19%	0,09%	0,14%	0,25%	0,21%	0,22%	D_0__Bacteria;D_1__Proteobacteria;D_2__Deltaproteobacteria;D_3__Myxococcales;D_4__BIrii41	metagenome
0,24%	0,17%	0,19%	0,17%	0,16%	0,08%	0,12%	0,17%	D_0__Bacteria;D_1__Proteobacteria;D_2__Alphaproteobacteria;D_3__Sphingomonadales;D_4__Sphingomonadaceae	Ellin6055
0,12%	0,19%	0,20%	0,17%	0,11%	0,10%	0,20%	0,20%	D_0__Bacteria;D_1__Proteobacteria;D_2__Gammaproteobacteria;D_3__Betaproteobacteriales;D_4__SC-I-84;Other	
0,05%	0,03%	0,01%	0,02%	0,23%	0,83%	0,17%	0,10%	D_0__Bacteria;D_1__Bacteroidetes;D_2__Bacteroidia;D_3__Sphingobacteriales;D_4__env,OPS 17	uncultured bacterium
0,18%	0,07%	0,08%	0,11%	0,25%	0,13%	0,29%	0,20%	D_0__Bacteria;D_1__Proteobacteria;D_2__Deltaproteobacteria;D_3__Desulfarculales;D_4__Desulfarculaceae	uncultured
0,18%	0,07%	0,06%	0,16%	0,16%	0,12%	0,23%	0,28%	D_0__Bacteria;D_1__Proteobacteria;D_2__Gammaproteobacteria;D_3__CCD24;D_4__uncultured bacterium	
0,12%	0,20%	0,24%	0,14%	0,12%	0,16%	0,15%	0,11%	D_0__Bacteria;D_1__Proteobacteria;D_2__Deltaproteobacteria;D_3__Bdellovibrionales;D_4__Bdellovibrionaceae	Bdellovibrio
0,12%	0,21%	0,14%	0,14%	0,14%	0,19%	0,16%	0,15%	D_0__Bacteria;D_1__Actinobacteria;D_2__Actinobacteria;D_3__Micromonosporales;D_4__Micromonosporaceae	Actinoplanes
0,26%	0,05%	0,09%	0,16%	0,20%	0,05%	0,21%	0,16%	D_0__Bacteria;D_1__Proteobacteria;D_2__Alphaproteobacteria;D_3__Rhizobiales;D_4__uncultured	uncultured bacterium
0,04%	0,05%	0,05%	0,09%	0,30%	0,22%	0,24%	0,26%	D_0__Bacteria;D_1__Acidobacteria;D_2__Blastocatellia (Subgroup 4);D_3__Blastocatellales;D_4__Blastocatellaceae	JGI 0001001-H03
0,17%	0,06%	0,11%	0,11%	0,24%	0,13%	0,20%	0,17%	D_0__Bacteria;D_1__Proteobacteria;D_2__Alphaproteobacteria;D_3__Rhizobiales;D_4__A0839	uncultured bacterium
0,19%	0,09%	0,16%	0,15%	0,19%	0,09%	0,15%	0,12%	D_0__Bacteria;D_1__Proteobacteria;D_2__Alphaproteobacteria;D_3__Rhizobiales;D_4__Rhizobiales Incertae Sedis	Bauldia
0,16%	0,06%	0,10%	0,10%	0,20%	0,16%	0,17%	0,23%	D_0__Bacteria;D_1__Proteobacteria;D_2__Alphaproteobacteria;D_3__Rhizobiales;D_4__KF-JG30-B3	uncultured bacterium
0,18%	0,26%	0,11%	0,09%	0,17%	0,15%	0,11%	0,10%	D_0__Bacteria;D_1__Actinobacteria;D_2__Acidimicrobiia;D_3__Microtrichales;D_4__uncultured;Other	
0,10%	0,22%	0,23%	0,12%	0,06%	0,21%	0,07%	0,10%	D_0__Bacteria;D_1__Proteobacteria;D_2__Gammaproteobacteria;D_3__R7C24;D_4__metagenome	
0,11%	0,15%	0,05%	0,08%	0,11%	0,27%	0,14%	0,23%	D_0__Bacteria;D_1__Bacteroidetes;D_2__Bacteroidia;D_3__Chitinophagales;D_4__Saprospiraceae	uncultured
0,20%	0,61%	0,19%	0,03%	0,05%	0,05%	0,04%	0,01%	D_0__Bacteria;D_1__Actinobacteria;D_2__Actinobacteria;D_3__Frankiales;D_4__Geodermatophilaceae	Blastococcus
0,05%	0,26%	0,14%	0,11%	0,06%	0,17%	0,15%	0,16%	D_0__Bacteria;D_1__Actinobacteria;D_2__Actinobacteria;D_3__Micrococcales;D_4__Microbacteriaceae	Lysinimonas
0,13%	0,03%	0,10%	0,09%	0,31%	0,10%	0,20%	0,14%	D_0__Bacteria;D_1__Proteobacteria;D_2__Alphaproteobacteria;D_3__Rhizobiales;D_4__Methyloligellaceae	uncultured
0,08%	0,04%	0,04%	0,04%	0,21%	0,26%	0,26%	0,23%	D_0__Bacteria;D_1__Proteobacteria;D_2__Deltaproteobacteria;D_3__Myxococcales;D_4__Phaselicystidaceae	Phaselicystis
0,02%	0,02%	0,29%	0,32%	0,04%	0,03%	0,12%	0,04%	D_0__Bacteria;D_1__Acidobacteria;D_2__Acidobacteriia;D_3__Acidobacteriales;D_4__Acidobacteriaceae (Subgroup 1)	Granulicella
0,11%	0,10%	0,11%	0,14%	0,15%	0,12%	0,16%	0,14%	D_0__Bacteria;D_1__Proteobacteria;D_2__Alphaproteobacteria;D_3__Rhizobiales;D_4__KF-JG30-B3	metagenome
0,12%	0,10%	0,14%	0,14%	0,17%	0,08%	0,13%	0,13%	D_0__Bacteria;D_1__Proteobacteria;D_2__Gammaproteobacteria;D_3__Diplorickettsiales;D_4__Diplorickettsiaceae	uncultured
0,21%	0,31%	0,18%	0,10%	0,07%	0,10%	0,06%	0,03%	D_0__Bacteria;D_1__Actinobacteria;D_2__Actinobacteria;D_3__Propionibacteriales;D_4__Nocardioidaceae	Marmoricola
0,12%	0,07%	0,11%	0,12%	0,13%	0,08%	0,21%	0,21%	D_0__Bacteria;D_1__Proteobacteria;D_2__Alphaproteobacteria;D_3__uncultured;D_4__uncultured bacterium	
0,14%	0,06%	0,10%	0,08%	0,12%	0,07%	0,33%	0,19%	D_0__Bacteria;D_1__Acidobacteria;D_2__Subgroup 17;D_3__uncultured bacterium;D_4__	
0,10%	0,15%	0,12%	0,12%	0,14%	0,10%	0,11%	0,18%	D_0__Bacteria;D_1__Proteobacteria;D_2__Alphaproteobacteria;D_3__Reyranellales;D_4__Reyranellaceae	uncultured
0,08%	0,42%	0,30%	0,07%	0,03%	0,11%	0,02%	0,03%	D_0__Bacteria;D_1__Actinobacteria;D_2__Actinobacteria;D_3__Micrococcales;D_4__Cellulomonadaceae	Cellulomonas
0,05%	0,05%	0,04%	0,06%	0,18%	0,20%	0,25%	0,19%	D_0__Bacteria;D_1__Verrucomicrobia;D_2__Verrucomicrobiae;D_3__Pedosphaerales;D_4__Pedosphaeraceae	metagenome
0,14%	0,08%	0,13%	0,13%	0,13%	0,08%	0,13%	0,14%	D_0__Bacteria;D_1__Proteobacteria;D_2__Alphaproteobacteria;D_3__Elsterales;D_4__uncultured;Other	
0,26%	0,22%	0,19%	0,09%	0,06%	0,06%	0,05%	0,04%	D_0__Bacteria;D_1__Actinobacteria;D_2__Actinobacteria;D_3__Frankiales;D_4__Sporichthyaceae	uncultured
0,16%	0,05%	0,12%	0,07%	0,22%	0,12%	0,14%	0,10%	D_0__Bacteria;D_1__Proteobacteria;D_2__Deltaproteobacteria;D_3__Myxococcales;D_4__Polyangiaceae	Pajaroellobacter
0,06%	0,07%	0,18%	0,17%	0,10%	0,10%	0,13%	0,08%	D_0__Bacteria;D_1__Proteobacteria;D_2__Gammaproteobacteria;D_3__Betaproteobacteriales;D_4__Burkholderiaceae	Massilia
0,10%	0,09%	0,08%	0,08%	0,16%	0,15%	0,16%	0,15%	D_0__Bacteria;D_1__Acidobacteria;D_2__Subgroup 6;D_3__uncultured Acidobacteriales bacterium;D_4__	
0,09%	0,09%	0,08%	0,09%	0,14%	0,12%	0,16%	0,18%	D_0__Bacteria;D_1__Proteobacteria;D_2__Gammaproteobacteria;D_3__Betaproteobacteriales;D_4__TRA3-20;Other	
0,12%	0,51%	0,13%	0,04%	0,04%	0,07%	0,03%	0,03%	D_0__Bacteria;D_1__Proteobacteria;D_2__Alphaproteobacteria;D_3__Rhizobiales;D_4__Beijerinckiaceae	Microvirga
0,02%	0,07%	0,10%	0,26%	0,05%	0,06%	0,09%	0,11%	D_0__Bacteria;D_1__Acidobacteria;D_2__Acidobacteriia;D_3__Acidobacteriales;D_4__Acidobacteriaceae (Subgroup 1)	Edaphobacter
0,04%	0,07%	0,06%	0,07%	0,16%	0,23%	0,14%	0,18%	D_0__Bacteria;D_1__Acidobacteria;D_2__Blastocatellia (Subgroup 4);D_3__Blastocatellales;D_4__Blastocatellaceae	uncultured
0,14%	0,08%	0,11%	0,08%	0,18%	0,07%	0,14%	0,12%	D_0__Bacteria;D_1__Actinobacteria;D_2__Acidimicrobiia;D_3__uncultured;D_4__uncultured bacterium	
0,07%	0,18%	0,10%	0,11%	0,07%	0,16%	0,08%	0,13%	D_0__Bacteria;D_1__Proteobacteria;D_2__Gammaproteobacteria;D_3__Xanthomonadales;D_4__Xanthomonadaceae	Lysobacter
0,07%	0,19%	0,35%	0,12%	0,05%	0,03%	0,05%	0,04%	D_0__Bacteria;D_1__Proteobacteria;D_2__Alphaproteobacteria;D_3__Sphingomonadales;D_4__Sphingomonadaceae	Porphyrobacter
0,05%	0,08%	0,24%	0,14%	0,05%	0,09%	0,10%	0,12%	D_0__Bacteria;D_1__Proteobacteria;D_2__Alphaproteobacteria;D_3__Rhodospirillales;D_4__uncultured	uncultured bacterium
0,27%	0,11%	0,20%	0,07%	0,07%	0,15%	0,06%	0,01%	D_0__Bacteria;D_1__Patescibacteria;D_2__Parcubacteria;D_3__Candidatus Kaiserbacteria;D_4__uncultured bacterium	
0,11%	0,25%	0,20%	0,08%	0,06%	0,08%	0,06%	0,06%	D_0__Bacteria;D_1__Actinobacteria;D_2__Acidimicrobiia;D_3__Microtrichales;D_4__Microtrichaceae	uncultured
0,05%	0,24%	0,16%	0,10%	0,03%	0,11%	0,08%	0,11%	D_0__Bacteria;D_1__Actinobacteria;D_2__Actinobacteria;D_3__Micrococcales;D_4__Microbacteriaceae;Other	
0,28%	0,05%	0,10%	0,09%	0,19%	0,03%	0,09%	0,06%	D_0__Bacteria;D_1__Actinobacteria;D_2__Thermoleophilia;D_3__Gaiellales;D_4__uncultured;Other	
0,03%	0,15%	0,07%	0,09%	0,05%	0,22%	0,09%	0,16%	D_0__Bacteria;D_1__Acidobacteria;D_2__Acidobacteriia;D_3__Solibacterales;D_4__Solibacteraceae (Subgroup 3)	Paludibaculum
0,12%	0,07%	0,16%	0,15%	0,08%	0,05%	0,08%	0,09%	D_0__Bacteria;D_1__Proteobacteria;D_2__Alphaproteobacteria;D_3__Rhizobiales;Other;Other	
0,05%	0,13%	0,04%	0,10%	0,05%	0,19%	0,09%	0,18%	D_0__Bacteria;D_1__Bacteroidetes;D_2__Bacteroidia;D_3__Cytophagales;D_4__Microscillaceae	Chryseolinea
0,05%	0,06%	0,12%	0,08%	0,14%	0,08%	0,15%	0,13%	D_0__Bacteria;D_1__Proteobacteria;D_2__Alphaproteobacteria;D_3__Rhodospirillales;D_4__Rhodospirillaceae	uncultured
0,06%	0,06%	0,10%	0,10%	0,16%	0,13%	0,09%	0,13%	D_0__Bacteria;D_1__Proteobacteria;D_2__Gammaproteobacteria;D_3__Diplorickettsiales;D_4__Diplorickettsiaceae	Aquicella
0,04%	0,10%	0,10%	0,21%	0,04%	0,08%	0,05%	0,08%	D_0__Bacteria;D_1__Proteobacteria;D_2__Alphaproteobacteria;D_3__Caulobacterales;D_4__Caulobacteraceae	Brevundimonas
0,09%	0,02%	0,08%	0,04%	0,17%	0,11%	0,20%	0,15%	D_0__Bacteria;D_1__Acidobacteria;D_2__Subgroup 17;Other;Other;Other	
0,26%	0,06%	0,03%	0,04%	0,18%	0,16%	0,05%	0,05%	D_0__Bacteria;D_1__Actinobacteria;D_2__Thermoleophilia;D_3__Solirubrobacterales;D_4__Solirubrobacteraceae	uncultured
0,01%	0,07%	0,02%	0,03%	0,17%	0,19%	0,20%	0,14%	D_0__Bacteria;D_1__Verrucomicrobia;D_2__Verrucomicrobiae;D_3__Opitutales;D_4__Opitutaceae	Lacunisphaera
0,06%	0,10%	0,26%	0,09%	0,05%	0,08%	0,07%	0,08%	D_0__Bacteria;D_1__Proteobacteria;D_2__Alphaproteobacteria;D_3__Rickettsiales;D_4__SM2D12	metagenome
0,02%	0,35%	0,25%	0,03%	0,01%	0,13%	0,02%	0,02%	D_0__Bacteria;D_1__Actinobacteria;D_2__Actinobacteria;D_3__Micrococcales;D_4__Cellulomonadaceae;Other	
0,08%	0,10%	0,12%	0,08%	0,13%	0,09%	0,13%	0,06%	D_0__Bacteria;D_1__Proteobacteria;D_2__Deltaproteobacteria;D_3__Oligoflexales;D_4__0319-6G20	uncultured bacterium
0,13%	0,16%	0,15%	0,10%	0,06%	0,07%	0,06%	0,03%	D_0__Bacteria;D_1__Planctomycetes;D_2__Phycisphaerae;D_3__Tepidisphaerales;D_4__Tepidisphaeraceae	uncultured bacterium
0,03%	0,03%	0,02%	0,06%	0,27%	0,14%	0,12%	0,12%	D_0__Bacteria;D_1__Acidobacteria;D_2__Blastocatellia (Subgroup 4);D_3__Blastocatellales;D_4__Blastocatellaceae	Stenotrophobacter

0,05%	0,03%	0,06%	0,17%	0,04%	0,06%	0,07%	0,21%	D_0__Bacteria;D_1__Verrucomicrobia;D_2__Verrucomicrobiae;D_3__Chthoniobacterales;D_4__Xiphinematobacteraceae	Candidatus Xiphinematobacter
0,09%	0,07%	0,04%	0,07%	0,16%	0,12%	0,16%	0,08%	D_0__Bacteria;D_1__Planctomycetes;D_2__OM190;D_3__uncultured bacterium;D_4__	
0,03%	0,20%	0,03%	0,12%	0,04%	0,16%	0,07%	0,07%	D_0__Bacteria;D_1__Verrucomicrobia;D_2__Verrucomicrobiae;D_3__Verrucomicrobiales;D_4__Rubritaleaceae	Luteolibacter
0,05%	0,05%	0,22%	0,06%	0,06%	0,16%	0,09%	0,09%	D_0__Bacteria;D_1__Proteobacteria;D_2__Gammaproteobacteria;D_3__R7C24;D_4__uncultured bacterium	
0,07%	0,21%	0,03%	0,05%	0,06%	0,25%	0,07%	0,05%	D_0__Bacteria;D_1__Chloroflexi;D_2__Chloroflexia;D_3__Thermomicrobiales;D_4__JG30-KF-CM45	uncultured bacterium
0,08%	0,06%	0,05%	0,17%	0,09%	0,03%	0,07%	0,09%	D_0__Bacteria;D_1__Actinobacteria;D_2__Actinobacteria;D_3__Frankiales;D_4__Acidothermaceae	Acidothermus
0,02%	0,06%	0,07%	0,12%	0,02%	0,16%	0,11%	0,14%	D_0__Bacteria;D_1__Actinobacteria;D_2__Actinobacteria;D_3__Pseudonocardiales;D_4__Pseudonocardiaceae	Actinophytocola
0,04%	0,05%	0,04%	0,03%	0,17%	0,12%	0,20%	0,12%	D_0__Bacteria;D_1__Verrucomicrobia;D_2__Verrucomicrobiae;D_3__Pedosphaerales;D_4__Pedosphaeraceae;Other	
0,10%	0,15%	0,07%	0,12%	0,04%	0,09%	0,04%	0,04%	D_0__Bacteria;D_1__Bacteroidetes;D_2__Bacteroidia;D_3__Chitinophagales;D_4__Chitinophagaceae	Flavitalea
0,05%	0,12%	0,12%	0,09%	0,04%	0,12%	0,06%	0,07%	D_0__Bacteria;D_1__Proteobacteria;D_2__Alphaproteobacteria;D_3__Micavibrionales;D_4__uncultured	uncultured bacterium
0,01%	0,39%	0,09%	0,07%	0,01%	0,07%	0,03%	0,02%	D_0__Bacteria;D_1__Proteobacteria;D_2__Gammaproteobacteria;D_3__Pseudomonadales;D_4__Moraxellaceae	uncultured
0,03%	0,13%	0,07%	0,15%	0,02%	0,09%	0,04%	0,09%	D_0__Bacteria;D_1__Proteobacteria;D_2__Alphaproteobacteria;D_3__Rhizobiales;D_4__Rhizobiaceae	Phyllobacterium
0,16%	0,06%	0,10%	0,09%	0,11%	0,04%	0,06%	0,05%	D_0__Bacteria;D_1__Planctomycetes;D_2__Phycisphaerae;D_3__Tepidisphaerales;D_4__WD2101 soil group;Other	
0,08%	0,09%	0,16%	0,07%	0,06%	0,08%	0,06%	0,07%	D_0__Bacteria;D_1__Proteobacteria;D_2__Alphaproteobacteria;D_3__Rickettsiales;D_4__SM2D12	uncultured bacterium
0,07%	0,06%	0,09%	0,11%	0,06%	0,04%	0,11%	0,09%	D_0__Bacteria;D_1__Proteobacteria;D_2__Deltaproteobacteria;D_3__Myxococcales;D_4__bacteriap25	uncultured bacterium
0,08%	0,05%	0,12%	0,11%	0,05%	0,08%	0,06%	0,08%	D_0__Bacteria;D_1__Proteobacteria;D_2__Gammaproteobacteria;D_3__R7C24;Other;Other	
0,06%	0,10%	0,04%	0,05%	0,09%	0,14%	0,10%	0,09%	D_0__Bacteria;D_1__Verrucomicrobia;D_2__Verrucomicrobiae;D_3__Verrucomicrobiales;D_4__Verrucomicrobiaceae	uncultured
0,07%	0,06%	0,13%	0,11%	0,05%	0,05%	0,09%	0,06%	D_0__Bacteria;D_1__Proteobacteria;D_2__Alphaproteobacteria;D_3__Rhizobiales;D_4__Labraceae	Labrys
0,08%	0,04%	0,03%	0,09%	0,13%	0,08%	0,15%	0,05%	D_0__Bacteria;D_1__Planctomycetes;D_2__Phycisphaerae;D_3__Phycisphaerales;D_4__Phycisphaeraceae	SM1A02
0,11%	0,07%	0,04%	0,08%	0,14%	0,04%	0,12%	0,03%	D_0__Bacteria;D_1__Planctomycetes;D_2__Planctomycetacia;D_3__Gemmatales;D_4__Gemmataceae	uncultured
0,06%	0,03%	0,07%	0,09%	0,09%	0,04%	0,13%	0,12%	D_0__Bacteria;D_1__Proteobacteria;D_2__Alphaproteobacteria;D_3__uncultured;Other;Other	
0,02%	0,11%	0,02%	0,07%	0,05%	0,21%	0,08%	0,07%	D_0__Bacteria;D_1__Proteobacteria;D_2__Gammaproteobacteria;D_3__Cellvibrionales;D_4__Halieaceae	OM60(NOR5) clade
0,04%	0,06%	0,02%	0,05%	0,08%	0,17%	0,11%	0,11%	D_0__Bacteria;D_1__Bacteroidetes;D_2__Bacteroidia;D_3__Cytophagales;D_4__Hymenobacteraceae	Adhaeribacter
0,12%	0,12%	0,10%	0,08%	0,05%	0,04%	0,05%	0,06%	D_0__Bacteria;D_1__Proteobacteria;D_2__Alphaproteobacteria;D_3__Tistrellales;D_4__Geminicoccaceae	Candidatus Alysiosphaera
0,15%	0,09%	0,08%	0,05%	0,09%	0,09%	0,05%	0,03%	D_0__Bacteria;D_1__Actinobacteria;D_2__Thermoleophilia;D_3__Solirubrobacterales;D_4__67-14;Other	
0,09%	0,08%	0,09%	0,08%	0,05%	0,10%	0,06%	0,05%	D_0__Bacteria;D_1__Proteobacteria;D_2__Deltaproteobacteria;D_3__SAR324 clade(Marine group B);D_4__uncultured bacterium	
0,07%	0,04%	0,12%	0,13%	0,04%	0,03%	0,07%	0,04%	D_0__Bacteria;D_1__Proteobacteria;D_2__Alphaproteobacteria;D_3__Rhizobiales;D_4__Xanthobacteraceae	Afipia
0,10%	0,15%	0,07%	0,06%	0,05%	0,09%	0,05%	0,05%	D_0__Bacteria;D_1__Chloroflexi;D_2__KD4-96;Other;Other;Other	
0,07%	0,09%	0,13%	0,09%	0,06%	0,04%	0,04%	0,06%	D_0__Bacteria;D_1__Proteobacteria;D_2__Alphaproteobacteria;D_3__Rhizobiales;D_4__Xanthobacteraceae	Pseudorhodoplanes
0,06%	0,12%	0,05%	0,03%	0,05%	0,09%	0,12%	0,09%	D_0__Bacteria;D_1__Proteobacteria;D_2__Gammaproteobacteria;D_3__Xanthomonadales;D_4__Rhodanobacteraceae	uncultured
0,05%	0,02%	0,05%	0,06%	0,12%	0,04%	0,14%	0,10%	D_0__Bacteria;D_1__Acidobacteria;D_2__Subgroup 5;D_3__uncultured Acidobacteria bacterium;D_4__	
0,04%	0,04%	0,04%	0,06%	0,06%	0,12%	0,13%	0,09%	D_0__Bacteria;D_1__Actinobacteria;D_2__Actinobacteria;D_3__Pseudonocardiales;D_4__Pseudonocardiaceae	Amycolatopsis
0,11%	0,01%	0,05%	0,05%	0,13%	0,03%	0,10%	0,11%	D_0__Bacteria;D_1__Acidobacteria;D_2__Blastocatellia (Subgroup 4);D_3__Elev-16S-573;D_4__uncultured bacterium	
0,06%	0,10%	0,06%	0,06%	0,09%	0,06%	0,10%	0,07%	D_0__Bacteria;D_1__Actinobacteria;D_2__Acidimicrobiia;D_3__uncultured;Other;Other	
0,08%	0,05%	0,08%	0,04%	0,09%	0,10%	0,08%	0,09%	D_0__Bacteria;D_1__Proteobacteria;D_2__Deltaproteobacteria;D_3__Bdellovibrionales;D_4__Bdellovibrionaceae	OM27 clade
0,01%	0,12%	0,00%	0,00%	0,01%	0,42%	0,01%	0,01%	D_0__Bacteria;D_1__Firmicutes;D_2__Bacilli;D_3__Bacillales;D_4__Planococcaceae	Psychrobacillus
0,00%	0,00%	0,02%	0,04%	0,01%	0,47%	0,01%	0,01%	D_0__Bacteria;D_1__Bacteroidetes;D_2__Bacteroidia;D_3__Flavobacteriales;D_4__Weeksellaceae	Chryseobacterium
0,09%	0,04%	0,07%	0,11%	0,02%	0,04%	0,03%	0,08%	D_0__Bacteria;D_1__Proteobacteria;D_2__Gammaproteobacteria;D_3__PLTA13;D_4__uncultured bacterium	
0,09%	0,05%	0,08%	0,10%	0,05%	0,02%	0,05%	0,04%	D_0__Bacteria;D_1__Proteobacteria;D_2__Alphaproteobacteria;D_3__Rhizobiales;D_4__Beijerinckiaceae	uncultured
0,01%	0,01%	0,24%	0,11%	0,02%	0,04%	0,03%	0,01%	D_0__Bacteria;D_1__Proteobacteria;D_2__Alphaproteobacteria;D_3__Caulobacterales;D_4__Caulobacteraceae	Asticcacaulis
0,09%	0,04%	0,07%	0,05%	0,11%	0,06%	0,06%	0,06%	D_0__Bacteria;D_1__Proteobacteria;D_2__Alphaproteobacteria;D_3__Rhizobiales;D_4__Rhizobiales Incertae Sedis	Nordella
0,14%	0,04%	0,08%	0,03%	0,12%	0,02%	0,07%	0,04%	D_0__Bacteria;D_1__Actinobacteria;D_2__MB-A2-108;D_3__uncultured bacterium;D_4__	

(B)

AI1b	AI2b	AI3b	AI4b	AII1b	AII2b	AII3b	AII4b	#OTU ID	
19,02%	16,38%	21,10%	14,36%	17,56%	16,93%	20,18%	19,42%	Unassigned;Other;Other;Other;Other;Other	
2,47%	4,23%	2,32%	5,25%	1,37%	1,29%	1,36%	3,38%	D_0__Bacteria;D_1__Proteobacteria;D_2__Alphaproteobacteria;D_3__Sphingomonadales;D_4__Sphingomonadaceae;	Sphingomonas
2,08%	0,39%	1,18%	5,15%	2,98%	0,83%	1,58%	4,94%	D_0__Bacteria;D_1__Verrucomicrobia;D_2__Verrucomicrobiae;D_3__Chthoniobacterales;D_4__Chthoniobacteraceae;	Candidatus Udaeobacter
2,77%	2,67%	2,67%	2,79%	4,28%	1,74%	1,36%	1,65%	D_0__Bacteria;D_1__Planctomycetes;D_2__Phycisphaerae;D_3__Tepidisphaerales;D_4__WD2101 soil group;	uncultured bacterium
1,90%	1,83%	2,71%	1,93%	2,17%	2,29%	3,31%	2,58%	D_0__Bacteria;D_1__Acidobacteria;D_2__Subgroup 6;D_3__uncultured bacterium;D_4__;	
3,19%	2,39%	2,79%	2,57%	2,03%	1,44%	1,87%	1,36%	D_0__Bacteria;D_1__Proteobacteria;D_2__Alphaproteobacteria;D_3__Rhizobiales;D_4__Xanthobacteraceae;	uncultured
1,66%	1,92%	2,70%	1,97%	1,55%	2,31%	2,92%	2,49%	D_0__Bacteria;D_1__Acidobacteria;D_2__Subgroup 6;D_3__uncultured Acidobacteria bacterium;D_4__;	
3,85%	2,58%	2,27%	1,25%	1,08%	1,17%	0,56%	0,70%	D_0__Bacteria;D_1__Actinobacteria;D_2__Thermoleophilia;D_3__Solirubrobacterales;D_4__67-14;	uncultured bacterium
0,71%	1,00%	1,41%	1,66%	1,67%	1,82%	1,29%	1,48%	D_0__Bacteria;D_1__Bacteroidetes;D_2__Bacteroidia;D_3__Chitinophagales;D_4__Chitinophagaceae;	uncultured
1,51%	1,16%	1,65%	1,95%	0,92%	0,90%	1,10%	1,18%	D_0__Bacteria;D_1__Proteobacteria;D_2__Alphaproteobacteria;D_3__Rhizobiales;D_4__Xanthobacteraceae;	Bradyrhizobium
1,05%	1,01%	1,58%	1,30%	0,98%	1,29%	1,81%	1,47%	D_0__Bacteria;D_1__Acidobacteria;D_2__Subgroup 6;Other;Other;Other	
1,92%	3,27%	1,64%	0,89%	0,58%	1,08%	0,28%	0,41%	D_0__Bacteria;D_1__Actinobacteria;D_2__Actinobacteria;D_3__Propionibacteriales;D_4__Nocardioidaceae;	Nocardioides
0,10%	0,60%	0,29%	0,58%	2,40%	2,99%	1,41%	1,92%	D_0__Bacteria;D_1__Bacteroidetes;D_2__Bacteroidia;D_3__Cytophagales;D_4__Microscillaceae;	uncultured
3,45%	1,12%	1,78%	0,95%	0,91%	0,49%	0,53%	0,66%	D_0__Bacteria;D_1__Actinobacteria;D_2__Thermoleophilia;D_3__Gaiellales;D_4__uncultured;	uncultured bacterium
1,29%	1,62%	1,33%	0,69%	1,31%	1,18%	0,99%	1,57%	D_0__Bacteria;D_1__Proteobacteria;D_2__Gammaproteobacteria;D_3__Betaproteobacteriales;D_4__Nitrosomonadaceae;	Ellin6067
1,97%	1,56%	1,58%	1,37%	0,79%	0,50%	0,56%	0,82%	D_0__Bacteria;D_1__Chloroflexi;D_2__KD4-96;D_3__uncultured bacterium;D_4__;	
1,38%	2,26%	0,72%	1,11%	1,20%	0,88%	0,98%	0,78%	D_0__Bacteria;D_1__Proteobacteria;D_2__Alphaproteobacteria;D_3__Sphingomonadales;D_4__Sphingomonadaceae;	uncultured
0,86%	2,28%	0,38%	0,21%	0,71%	4,16%	1,01%	0,32%	D_0__Bacteria;D_1__Firmicutes;D_2__Bacilli;D_3__Bacillales;D_4__Bacillaceae;	Bacillus
3,18%	2,47%	1,41%	0,73%	0,44%	0,49%	0,16%	0,24%	D_0__Bacteria;D_1__Actinobacteria;D_2__Thermoleophilia;D_3__Solirubrobacterales;D_4__Solirubrobacteraceae;	Solirubrobacter
0,50%	0,79%	0,61%	2,07%	1,06%	0,93%	0,61%	0,87%	D_0__Bacteria;D_1__Patescibacteria;D_2__Saccharimonadia;D_3__Saccharimonadales;D_4__uncultured bacterium;	
0,30%	0,53%	0,57%	1,39%	0,72%	1,08%	1,32%	1,63%	D_0__Bacteria;D_1__Proteobacteria;D_2__Alphaproteobacteria;D_3__Dongiales;D_4__Dongiaceae;	Dongia
0,76%	0,47%	1,04%	1,28%	1,16%	0,75%	1,00%	0,88%	D_0__Bacteria;D_1__Proteobacteria;D_2__Alphaproteobacteria;D_3__Micropepsales;D_4__Micropepsaceae;	uncultured
0,57%	0,34%	0,62%	0,31%	1,25%	1,04%	2,05%	1,56%	D_0__Bacteria;D_1__Proteobacteria;D_2__Gammaproteobacteria;D_3__Betaproteobacteriales;D_4__Nitrosomonadaceae;	MND1
0,98%	1,05%	1,07%	1,28%	0,33%	0,75%	0,32%	0,73%	D_0__Bacteria;D_1__Actinobacteria;D_2__Actinobacteria;D_3__Corynebacteriales;D_4__Mycobacteriaceae;	Mycobacterium
1,56%	1,12%	1,11%	0,60%	0,98%	0,56%	0,60%	0,48%	D_0__Bacteria;D_1__Actinobacteria;D_2__Acidimicrobiia;D_3__IMCC26256;D_4__uncultured bacterium;	
0,38%	0,55%	0,67%	1,02%	0,77%	0,93%	0,86%	1,08%	D_0__Bacteria;D_1__Proteobacteria;D_2__Alphaproteobacteria;D_3__Reyranellales;D_4__Reyranellaceae;	Reyranella
1,21%	0,56%	0,57%	0,39%	1,37%	0,61%	1,20%	0,89%	D_0__Bacteria;D_1__Gemmatimonadetes;D_2__Gemmatimonadetes;D_3__Gemmatimonadales;D_4__Gemmatimonadace ae;	uncultured
0,54%	0,62%	0,71%	1,07%	0,73%	0,82%	0,81%	0,78%	D_0__Bacteria;D_1__Acidobacteria;D_2__Acidobacteriia;D_3__Solibacterales;D_4__Solibacteraceae (Subgroup 3);	Bryobacter
0,68%	0,25%	0,51%	0,71%	1,22%	0,83%	1,05%	0,84%	D_0__Bacteria;D_1__Acidobacteria;D_2__Acidobacteriia;D_3__Solibacterales;D_4__Solibacteraceae (Subgroup 3);	Candidatus Solibacter
1,16%	0,52%	0,79%	0,46%	1,24%	0,52%	0,94%	0,64%	D_0__Bacteria;D_1__Acidobacteria;D_2__Blastocatellia (Subgroup 4);D_3__Pyrinomonadales;D_4__Pyrinomonadaceae;	RB41
0,28%	0,32%	0,44%	0,61%	1,01%	0,88%	0,92%	1,21%	D_0__Bacteria;D_1__Verrucomicrobia;D_2__Verrucomicrobiae;D_3__Chthoniobacterales;D_4__Chthoniobacteraceae;	Chthoniobacter
0,52%	0,34%	0,43%	0,32%	1,38%	0,89%	0,94%	0,97%	D_0__Bacteria;D_1__Proteobacteria;D_2__Deltaproteobacteria;D_3__Myxococcales;D_4__Haliangiaceae;	Haliangium
0,36%	0,37%	0,56%	0,35%	0,78%	0,83%	1,33%	0,91%	D_0__Bacteria;D_1__Proteobacteria;D_2__Gammaproteobacteria;D_3__Betaproteobacteriales;D_4__TRA3-20;	uncultured bacterium
0,44%	0,84%	0,63%	0,81%	0,29%	0,95%	0,54%	0,46%	D_0__Bacteria;D_1__Actinobacteria;D_2__Actinobacteria;D_3__Streptomycetales;D_4__Streptomycetaceae;	Streptomyces
0,35%	0,73%	0,60%	0,45%	0,91%	1,04%	0,71%	0,43%	D_0__Bacteria;D_1__Bacteroidetes;D_2__Bacteroidia;D_3__Chitinophagales;D_4__Chitinophagaceae;	Terrimonas
0,24%	0,33%	0,34%	1,15%	0,60%	0,50%	0,50%	0,85%	D_0__Bacteria;D_1__Proteobacteria;D_2__Alphaproteobacteria;D_3__Caulobacterales;D_4__Caulobacteraceae;	Phenylobacterium
0,21%	0,46%	0,46%	0,51%	0,47%	0,82%	1,00%	0,90%	D_0__Bacteria;D_1__Proteobacteria;D_2__Gammaproteobacteria;D_3__Gammaproteobacteria Incertae	Acidibacter

								Sedis;D_4__Unknown Family;	
0,52%	0,55%	0,73%	0,71%	0,43%	0,32%	0,55%	0,64%	D_0__Bacteria;D_1__Proteobacteria;D_2__Gammaproteobacteria;D_3__Betaproteobacteriales;D_4__SC-I-84;	uncultured bacterium
0,80%	0,64%	0,62%	0,51%	0,52%	0,45%	0,38%	0,29%	D_0__Bacteria;D_1__Proteobacteria;D_2__Alphaproteobacteria;D_3__Rhizobiales;D_4__Xanthobacteraceae;	Rhodoplanes
0,26%	0,55%	0,58%	0,35%	0,73%	0,66%	0,50%	0,41%	D_0__Bacteria;D_1__Bacteroidetes;D_2__Bacteroidia;D_3__Chitinophagales;D_4__Chitinophagaceae;	Ferruginibacter
0,02%	0,24%	0,15%	0,85%	0,20%	1,20%	0,16%	0,66%	D_0__Bacteria;D_1__Bacteroidetes;D_2__Bacteroidia;D_3__Flavobacteriales;D_4__Flavobacteriaceae;	Flavobacterium
1,53%	0,51%	0,74%	0,38%	0,23%	0,16%	0,12%	0,18%	D_0__Bacteria;D_1__Actinobacteria;D_2__Thermoleophilia;D_3__Solirubrobacterales;D_4__Solirubrobacteraceae;	Conexibacter
0,58%	0,50%	0,46%	0,24%	0,83%	0,41%	0,56%	0,38%	D_0__Bacteria;D_1__Actinobacteria;D_2__Acidimicrobiia;D_3__Microtrichales;D_4__uncultured;	uncultured bacterium
0,12%	0,10%	0,19%	0,14%	1,18%	0,52%	1,02%	0,51%	D_0__Bacteria;D_1__Verrucomicrobia;D_2__Verrucomicrobiae;D_3__Pedosphaerales;D_4__Pedosphaeraceae;	uncultured bacterium
0,92%	0,49%	0,77%	0,29%	0,29%	0,25%	0,33%	0,23%	D_0__Bacteria;D_1__Actinobacteria;D_2__Thermoleophilia;D_3__Gaiellales;D_4__Gaiellaceae;	Gaiella
0,27%	0,46%	0,39%	0,54%	0,49%	0,50%	0,30%	0,36%	D_0__Bacteria;D_1__Proteobacteria;D_2__Alphaproteobacteria;D_3__Rhizobiales;D_4__Devosiaceae;	Devosia
0,51%	0,17%	0,31%	0,34%	0,75%	0,38%	0,36%	0,61%	D_0__Bacteria;D_1__Gemmatimonadetes;D_2__Gemmatimonadetes;D_3__Gemmatimonadales;D_4__Gemmatimonadaceae;	Gemmatimonas
0,21%	0,40%	0,38%	0,71%	0,14%	0,49%	0,17%	0,46%	D_0__Bacteria;D_1__Actinobacteria;D_2__Actinobacteria;D_3__Propionibacteriales;D_4__Nocardioidaceae;	Kribbella
0,38%	1,13%	0,41%	0,20%	0,31%	0,51%	0,18%	0,30%	D_0__Bacteria;D_1__Actinobacteria;D_2__Acidimicrobiia;D_3__Microtrichales;D_4__Iamiaceae;	Iamia
0,35%	0,44%	0,29%	0,72%	0,24%	0,18%	0,19%	0,36%	D_0__Bacteria;D_1__Proteobacteria;D_2__Alphaproteobacteria;D_3__Rhizobiales;D_4__Xanthobacteraceae;	Pseudolabrys
0,37%	0,84%	0,34%	0,51%	0,24%	0,26%	0,09%	0,26%	D_0__Bacteria;D_1__Proteobacteria;D_2__Alphaproteobacteria;D_3__Sphingomonadales;D_4__Sphingomonadaceae;	Altererythrobacter
0,55%	0,29%	0,30%	0,49%	0,29%	0,18%	0,25%	0,47%	D_0__Bacteria;D_1__Proteobacteria;D_2__Alphaproteobacteria;D_3__Elsterales;D_4__uncultured;	uncultured bacterium
0,42%	0,95%	0,53%	0,27%	0,18%	0,28%	0,15%	0,19%	D_0__Bacteria;D_1__Actinobacteria;D_2__Acidimicrobiia;D_3__Microtrichales;D_4__Ilumatobacteraceae;	uncultured
0,15%	0,33%	0,28%	0,21%	0,51%	0,57%	0,46%	0,36%	D_0__Bacteria;D_1__Proteobacteria;D_2__Alphaproteobacteria;D_3__Caulobacterales;D_4__Hyphomonadaceae;	Hirschia
0,17%	0,37%	0,28%	0,50%	0,17%	0,36%	0,32%	0,39%	D_0__Bacteria;D_1__Proteobacteria;D_2__Alphaproteobacteria;D_3__Rhizobiales;D_4__Rhizobiaceae;	Mesorhizobium
0,30%	0,22%	0,26%	0,50%	0,27%	0,15%	0,27%	0,26%	D_0__Bacteria;D_1__Proteobacteria;D_2__Alphaproteobacteria;D_3__Acetobacterales;D_4__Acetobacteraceae;	uncultured
0,09%	0,24%	0,22%	0,23%	0,20%	0,44%	0,52%	0,41%	D_0__Bacteria;D_1__Proteobacteria;D_2__Gammaproteobacteria;D_3__Steroidobacterales;D_4__Steroidobacteraceae;	uncultured
0,41%	0,34%	0,39%	0,18%	0,40%	0,26%	0,25%	0,17%	D_0__Bacteria;D_1__Actinobacteria;D_2__Acidimicrobiia;D_3__Microtrichales;D_4__Ilumatobacteraceae;	CL500-29 marine group
0,12%	0,30%	0,25%	0,34%	0,24%	0,27%	0,31%	0,40%	D_0__Bacteria;D_1__Proteobacteria;D_2__Gammaproteobacteria;D_3__Betaproteobacteriales;D_4__Burkholderiaceae;	Rhizobacter
0,12%	0,28%	0,27%	0,37%	0,18%	0,26%	0,32%	0,34%	D_0__Bacteria;D_1__Proteobacteria;D_2__Gammaproteobacteria;D_3__Betaproteobacteriales;D_4__Burkholderiaceae;Other	
0,28%	0,44%	0,32%	0,17%	0,36%	0,26%	0,12%	0,30%	D_0__Bacteria;D_1__Proteobacteria;D_2__Gammaproteobacteria;D_3__Xanthomonadales;D_4__Xanthomonadaceae;	Arenimonas
0,13%	0,54%	0,23%	0,19%	0,36%	0,31%	0,25%	0,23%	D_0__Bacteria;D_1__Proteobacteria;D_2__Alphaproteobacteria;D_3__Caulobacterales;D_4__Hyphomonadaceae;	SWB02
0,41%	0,45%	0,35%	0,18%	0,26%	0,21%	0,24%	0,14%	D_0__Bacteria;D_1__Proteobacteria;D_2__Alphaproteobacteria;D_3__Rhizobiales;D_4__Hyphomicrobiaceae;	Pedomicrobium
0,05%	0,04%	0,19%	0,39%	0,10%	0,15%	0,62%	0,46%	D_0__Bacteria;D_1__Proteobacteria;D_2__Gammaproteobacteria;D_3__Betaproteobacteriales;D_4__Burkholderiaceae;	Burkholderia-Caballeronia-Paraburkholderia
0,16%	0,19%	0,25%	0,42%	0,29%	0,23%	0,22%	0,22%	D_0__Bacteria;D_1__Proteobacteria;D_2__Alphaproteobacteria;D_3__Caulobacterales;D_4__Caulobacteraceae;	uncultured
0,11%	0,12%	0,23%	0,16%	0,31%	0,37%	0,56%	0,34%	D_0__Bacteria;D_1__Proteobacteria;D_2__Gammaproteobacteria;D_3__Betaproteobacteriales;D_4__Nitrosomonadaceae;	IS-44
0,43%	0,39%	0,40%	0,21%	0,20%	0,17%	0,11%	0,15%	D_0__Bacteria;D_1__Actinobacteria;D_2__Acidimicrobiia;D_3__IMCC26256;Other;Other	
0,08%	0,16%	0,18%	0,08%	0,39%	0,51%	0,41%	0,29%	D_0__Bacteria;D_1__Proteobacteria;D_2__Deltaproteobacteria;D_3__Myxococcales;D_4__Sandaracinaceae;	uncultured
0,34%	0,12%	0,21%	0,23%	0,36%	0,15%	0,20%	0,22%	D_0__Bacteria;D_1__Acidobacteria;D_2__Holophagae;D_3__Subgroup 7;D_4__uncultured bacterium;	
0,06%	0,31%	0,16%	0,24%	0,15%	0,41%	0,22%	0,21%	D_0__Bacteria;D_1__Proteobacteria;D_2__Alphaproteobacteria;D_3__Sphingomonadales;D_4__Sphingomonadaceae;	Novosphingobium
0,02%	0,02%	0,06%	0,53%	0,13%	0,19%	0,19%	0,28%	D_0__Bacteria;D_1__Bacteroidetes;D_2__Bacteroidia;D_3__Sphingobacteriales;D_4__Sphingobacteriaceae;	Mucilaginibacter
0,02%	0,06%	0,07%	0,23%	0,23%	0,40%	0,51%	0,19%	D_0__Bacteria;D_1__Proteobacteria;D_2__Gammaproteobacteria;D_3__Pseudomonadales;D_4__Pseudomonadaceae;	Pseudomonas
0,04%	0,04%	0,14%	0,36%	0,18%	0,19%	0,30%	0,33%	D_0__Bacteria;D_1__Proteobacteria;D_2__Gammaproteobacteria;D_3__Xanthomonadales;D_4__Rhodanobacteraceae;	Dokdonella
0,68%	0,13%	0,31%	0,16%	0,20%	0,07%	0,10%	0,12%	D_0__Bacteria;D_1__Actinobacteria;D_2__Thermoleophilia;D_3__Gaiellales;D_4__uncultured;Other	
0,18%	0,17%	0,20%	0,23%	0,17%	0,16%	0,31%	0,29%	D_0__Bacteria;D_1__Proteobacteria;D_2__Alphaproteobacteria;D_3__uncultured;D_4__uncultured bacterium;	
0,10%	0,14%	0,19%	0,35%	0,08%	0,27%	0,24%	0,22%	D_0__Bacteria;D_1__Proteobacteria;D_2__Alphaproteobacteria;D_3__Rhizobiales;D_4__Rhizobiaceae;	Allorhizobium-Neorhizobium-Pararhizobium-Rhizobium
0,30%	0,33%	0,15%	0,22%	0,13%	0,30%	0,11%	0,15%	D_0__Bacteria;D_1__Actinobacteria;D_2__Actinobacteria;D_3__Pseudonocardiales;D_4__Pseudonocardiaceae;	Pseudonocardia
0,15%	0,16%	0,24%	0,32%	0,09%	0,11%	0,24%	0,28%	D_0__Bacteria;D_1__Proteobacteria;D_2__Deltaproteobacteria;D_3__Myxococcales;D_4__bacteriap25;	uncultured bacterium
0,01%	0,03%	0,03%	0,66%	0,05%	0,35%	0,07%	0,04%	D_0__Bacteria;D_1__Bacteroidetes;D_2__Bacteroidia;D_3__Sphingobacteriales;D_4__Sphingobacteriaceae;	Pedobacter
0,05%	0,19%	0,16%	0,16%	0,11%	0,38%	0,36%	0,28%	D_0__Bacteria;D_1__Proteobacteria;D_2__Gammaproteobacteria;D_3__Steroidobacterales;D_4__Steroidobacteraceae;	Steroidobacter
0,19%	0,10%	0,18%	0,14%	0,27%	0,25%	0,26%	0,28%	D_0__Bacteria;D_1__Proteobacteria;D_2__Alphaproteobacteria;D_3__Rhizobiales;D_4__KF-JG30-B3;	uncultured bacterium
0,10%	0,18%	0,19%	0,25%	0,17%	0,22%	0,23%	0,24%	D_0__Bacteria;D_1__Proteobacteria;D_2__Gammaproteobacteria;D_3__Betaproteobacteriales;D_4__Burkholderiaceae;	uncultured
0,10%	0,10%	0,10%	0,23%	0,20%	0,25%	0,39%	0,21%	D_0__Bacteria;D_1__Acidobacteria;D_2__Thermoanaerobaculia;D_3__Thermoanaerobaculales;D_4__Thermoanaerobaculaceae;	Subgroup 10
0,34%	0,10%	0,20%	0,35%	0,12%	0,08%	0,08%	0,18%	D_0__Bacteria;D_1__Actinobacteria;D_2__Actinobacteria;D_3__Frankiales;D_4__Frankiaceae;	Jatrophihabitans
0,24%	0,39%	0,17%	0,17%	0,18%	0,12%	0,18%	0,17%	D_0__Bacteria;D_1__Proteobacteria;D_2__Alphaproteobacteria;D_3__Sphingomonadales;D_4__Sphingomonadaceae;	Ellin6055
0,14%	0,10%	0,18%	0,14%	0,27%	0,23%	0,35%	0,23%	D_0__Bacteria;D_1__Proteobacteria;D_2__Deltaproteobacteria;D_3__Desulfarculales;D_4__Desulfarculaceae;	uncultured
0,12%	0,60%	0,19%	0,11%	0,09%	0,37%	0,07%	0,13%	D_0__Bacteria;D_1__Actinobacteria;D_2__Acidimicrobiia;D_3__Microtrichales;D_4__Ilumatobacteraceae;	Ilumatobacter
0,23%	0,28%	0,21%	0,18%	0,17%	0,15%	0,18%	0,18%	D_0__Bacteria;D_1__Proteobacteria;D_2__Alphaproteobacteria;D_3__Rhizobiales;D_4__uncultured;	uncultured bacterium
0,21%	0,17%	0,34%	0,16%	0,10%	0,10%	0,34%	0,18%	D_0__Bacteria;D_1__Acidobacteria;D_2__Subgroup 17;D_3__uncultured bacterium;D_4__;	
0,11%	0,10%	0,14%	0,15%	0,19%	0,23%	0,36%	0,31%	D_0__Bacteria;D_1__Proteobacteria;D_2__Gammaproteobacteria;D_3__CCD24;D_4__uncultured bacterium;	
0,16%	0,21%	0,20%	0,21%	0,19%	0,20%	0,23%	0,12%	D_0__Bacteria;D_1__Proteobacteria;D_2__Alphaproteobacteria;D_3__Rhizobiales;D_4__Hyphomicrobiaceae;	Hyphomicrobium
0,17%	0,11%	0,26%	0,21%	0,13%	0,10%	0,27%	0,21%	D_0__Bacteria;D_1__Proteobacteria;D_2__Gammaproteobacteria;D_3__Betaproteobacteriales;D_4__SC-I-84;Other	
0,20%	0,15%	0,16%	0,24%	0,13%	0,08%	0,18%	0,26%	D_0__Bacteria;D_1__Proteobacteria;D_2__Alphaproteobacteria;D_3__Elsterales;D_4__uncultured;Other	
0,30%	0,24%	0,19%	0,11%	0,24%	0,10%	0,17%	0,17%	D_0__Bacteria;D_1__Actinobacteria;D_2__Acidimicrobiia;D_3__uncultured;D_4__uncultured bacterium;	
0,18%	0,30%	0,20%	0,14%	0,19%	0,14%	0,22%	0,11%	D_0__Bacteria;D_1__Proteobacteria;D_2__Alphaproteobacteria;D_3__Rhizobiales;D_4__Rhizobiales Incertae Sedis;	uncultured
0,01%	0,02%	0,08%	0,45%	0,02%	0,04%	0,08%	0,40%	D_0__Bacteria;D_1__Proteobacteria;D_2__Gammaproteobacteria;D_3__Xanthomonadales;D_4__Rhodanobacteraceae;	Luteibacter
0,21%	0,06%	0,27%	0,06%	0,21%	0,05%	0,46%	0,15%	D_0__Bacteria;D_1__Rokubacteria;D_2__NC10;D_3__Rokubacteriales;D_4__uncultured bacterium;	
0,21%	0,05%	0,09%	0,42%	0,06%	0,02%	0,08%	0,21%	D_0__Bacteria;D_1__Actinobacteria;D_2__Actinobacteria;D_3__Frankiales;D_4__Acidothermaceae;	Acidothermus
0,21%	0,17%	0,20%	0,21%	0,12%	0,09%	0,15%	0,13%	D_0__Bacteria;D_1__Proteobacteria;D_2__Alphaproteobacteria;D_3__Rhizobiales;D_4__Xanthobacteraceae;Other	
0,06%	0,08%	0,07%	0,18%	0,28%	0,20%	0,26%	0,19%	D_0__Bacteria;D_1__Acidobacteria;D_2__Blastocatellia (Subgroup 4);D_3__Blastocatellales;D_4__Blastocatellaceae;	JGI 0001001-H03
0,04%	0,14%	0,11%	0,26%	0,14%	0,20%	0,15%	0,13%	D_0__Bacteria;D_1__Proteobacteria;D_2__Alphaproteobacteria;D_3__Caulobacterales;D_4__Caulobacteraceae;	Caulobacter
0,04%	0,20%	0,14%	0,17%	0,16%	0,20%	0,17%	0,19%	D_0__Bacteria;D_1__Acidobacteria;D_2__Blastocatellia (Subgroup 4);D_3__Blastocatellales;D_4__Blastocatellaceae;	uncultured
0,19%	0,08%	0,10%	0,13%	0,22%	0,16%	0,23%	0,15%	D_0__Bacteria;D_1__Proteobacteria;D_2__Alphaproteobacteria;D_3__Rhizobiales;D_4__Methyloligellaceae;	uncultured
0,06%	0,19%	0,21%	0,21%	0,02%	0,30%	0,10%	0,10%	D_0__Bacteria;D_1__Actinobacteria;D_2__Actinobacteria;D_3__Pseudonocardiales;D_4__Pseudonocardiaceae;	Actinophytocola
0,05%	0,03%	0,09%	0,27%	0,11%	0,15%	0,12%	0,27%	D_0__Bacteria;D_1__Proteobacteria;D_2__Gammaproteobacteria;D_3__Xanthomonadales;D_4__Rhodanobacteraceae;	Rhodanobacter
0,01%	0,06%	0,04%	0,07%	0,17%	0,43%	0,16%	0,31%	D_0__Bacteria;D_1__Bacteroidetes;D_2__Bacteroidia;D_3__Cytophagales;D_4__Microscillaceae;	Ohtaekwangia
0,04%	0,12%	0,10%	0,25%	0,07%	0,21%	0,10%	0,16%	D_0__Bacteria;D_1__Proteobacteria;D_2__Gammaproteobacteria;D_3__Betaproteobacteriales;D_4__Burkholderiaceae;	Variovorax
0,05%	0,11%	0,09%	0,30%	0,03%	0,07%	0,06%	0,28%	D_0__Bacteria;D_1__Verrucomicrobia;D_2__Verrucomicrobiae;D_3__Chthoniobacterales;D_4__Xiphinematobacteraceae;	Candidatus Xiphinematobacter
0,11%	0,09%	0,14%	0,09%	0,19%	0,21%	0,20%	0,17%	D_0__Bacteria;D_1__Proteobacteria;D_2__Gammaproteobacteria;D_3__Betaproteobacteriales;D_4__TRA3-20;	metagenome
0,11%	0,17%	0,14%	0,08%	0,24%	0,17%	0,20%	0,10%	D_0__Bacteria;D_1__Proteobacteria;D_2__Alphaproteobacteria;D_3__Rhizobiales;D_4__A0839;	uncultured bacterium
0,06%	0,11%	0,11%	0,25%	0,15%	0,12%	0,11%	0,13%	D_0__Bacteria;D_1__Proteobacteria;D_2__Alphaproteobacteria;D_3__Sphingomonadales;D_4__Sphingomonadaceae;	Sphingobium
0,10%	0,24%	0,13%	0,18%	0,10%	0,09%	0,10%	0,15%	D_0__Bacteria;D_1__Proteobacteria;D_2__Alphaproteobacteria;D_3__Sphingomonadales;D_4__Sphingomonadaceae;Other	
0,27%	0,33%	0,22%	0,12%	0,05%	0,07%	0,04%	0,03%	D_0__Bacteria;D_1__Actinobacteria;D_2__Actinobacteria;D_3__Frankiales;D_4__Sporichthyaceae;	uncultured
0,03%	0,23%	0,17%	0,07%	0,07%	0,27%	0,09%	0,20%	D_0__Bacteria;D_1__Bacteroidetes;D_2__Bacteroidia;D_3__Chitinophagales;D_4__Saprospiraceae;	uncultured
0,09%	0,03%	0,06%	0,05%	0,20%	0,25%	0,22%	0,26%	D_0__Bacteria;D_1__Proteobacteria;D_2__Deltaproteobacteria;D_3__Myxococcales;D_4__Phaselicystidaceae;	Phaselicystis

0,13%	0,14%	0,16%	0,10%	0,15%	0,14%	0,16%	0,11%	D_0__Bacteria;D_1__Acidobacteria;D_2__Subgroup 6;D_3__uncultured Acidobacteriales bacterium;D_4__;	
0,10%	0,14%	0,15%	0,16%	0,15%	0,13%	0,14%	0,07%	D_0__Bacteria;D_1__Proteobacteria;D_2__Deltaproteobacteria;D_3__Bdellovibrionales;D_4__Bdellovibrionaceae;	Bdellovibrio
0,19%	0,14%	0,14%	0,11%	0,15%	0,11%	0,13%	0,12%	D_0__Bacteria;D_1__Proteobacteria;D_2__Alphaproteobacteria;D_3__Rhizobiales;D_4__Rhizobiales Incertae Sedis;	Bauldia
0,39%	0,16%	0,22%	0,05%	0,13%	0,07%	0,07%	0,03%	D_0__Bacteria;D_1__Actinobacteria;D_2__MB-A2-108;D_3__uncultured bacterium;D_4__;	
0,08%	0,08%	0,15%	0,14%	0,12%	0,09%	0,21%	0,16%	D_0__Bacteria;D_1__Proteobacteria;D_2__Alphaproteobacteria;D_3__uncultured;Other;Other	
0,03%	0,05%	0,07%	0,04%	0,21%	0,25%	0,23%	0,21%	D_0__Bacteria;D_1__Proteobacteria;D_2__Deltaproteobacteria;D_3__Myxococcales;D_4__BIrii41;	uncultured bacterium
0,16%	0,27%	0,14%	0,07%	0,12%	0,13%	0,07%	0,09%	D_0__Bacteria;D_1__Actinobacteria;D_2__Acidimicrobiia;D_3__Microtrichales;D_4__uncultured;Other	
0,07%	0,04%	0,11%	0,30%	0,04%	0,04%	0,08%	0,12%	D_0__Bacteria;D_1__Actinobacteria;D_2__Actinobacteria;D_3__Micromonosporales;D_4__Micromonosporaceae;	uncultured
0,07%	0,07%	0,11%	0,13%	0,18%	0,11%	0,15%	0,13%	D_0__Bacteria;D_1__Proteobacteria;D_2__Alphaproteobacteria;D_3__Rhizobiales;D_4__KF-JG30-B3;	metagenome
0,08%	0,08%	0,11%	0,07%	0,14%	0,15%	0,21%	0,13%	D_0__Bacteria;D_1__Proteobacteria;D_2__Gammaproteobacteria;D_3__Betaproteobacteriales;D_4__TRA3-20;Other	
0,05%	0,13%	0,08%	0,15%	0,07%	0,16%	0,07%	0,18%	D_0__Bacteria;D_1__Actinobacteria;D_2__Actinobacteria;D_3__Micrococcales;D_4__Microbacteriaceae;	Lysinimonas
0,03%	0,12%	0,11%	0,13%	0,11%	0,21%	0,07%	0,12%	D_0__Bacteria;D_1__Bacteroidetes;D_2__Bacteroidia;D_3__Chitinophagales;D_4__Chitinophagaceae;	Parafilimonas
0,06%	0,10%	0,10%	0,12%	0,14%	0,13%	0,14%	0,10%	D_0__Bacteria;D_1__Proteobacteria;D_2__Gammaproteobacteria;D_3__Diplorickettsiales;D_4__Diplorickettsiaceae;	uncultured
0,12%	0,24%	0,12%	0,08%	0,10%	0,07%	0,12%	0,08%	D_0__Bacteria;D_1__Actinobacteria;D_2__Acidimicrobiia;D_3__uncultured;Other;Other	
0,12%	0,11%	0,09%	0,11%	0,11%	0,15%	0,08%	0,09%	D_0__Bacteria;D_1__Actinobacteria;D_2__Actinobacteria;D_3__Micromonosporales;D_4__Micromonosporaceae;	Actinoplanes
0,07%	0,09%	0,10%	0,03%	0,16%	0,17%	0,20%	0,11%	D_0__Bacteria;D_1__Planctomycetes;D_2__OM190;D_3__uncultured bacterium;D_4__;	
0,04%	0,08%	0,07%	0,22%	0,09%	0,10%	0,06%	0,07%	D_0__Bacteria;D_1__Patescibacteria;D_2__Saccharimonadia;D_3__Saccharimonadales;Other;Other	
0,07%	0,17%	0,06%	0,10%	0,08%	0,13%	0,11%	0,13%	D_0__Bacteria;D_1__Proteobacteria;D_2__Alphaproteobacteria;D_3__Reyranellales;D_4__Reyranellaceae;	uncultured
0,07%	0,15%	0,09%	0,14%	0,04%	0,06%	0,07%	0,18%	D_0__Bacteria;D_1__Proteobacteria;D_2__Gammaproteobacteria;D_3__PLTA13;D_4__uncultured bacterium;	
0,19%	0,09%	0,14%	0,05%	0,12%	0,06%	0,12%	0,10%	D_0__Bacteria;D_1__Acidobacteria;D_2__Blastocatellia (Subgroup 4);D_3__Elev-16S-573;D_4__uncultured bacterium;	
0,04%	0,28%	0,01%	0,01%	0,03%	0,52%	0,04%	0,01%	D_0__Bacteria;D_1__Firmicutes;D_2__Bacilli;D_3__Bacillales;D_4__Planococcaceae;	Lysinibacillus
0,22%	0,41%	0,16%	0,02%	0,03%	0,06%	0,02%	0,01%	D_0__Bacteria;D_1__Actinobacteria;D_2__Actinobacteria;D_3__Frankiales;D_4__Geodermatophilaceae;	Blastococcus
0,02%	0,03%	0,04%	0,04%	0,23%	0,16%	0,19%	0,17%	D_0__Bacteria;D_1__Verrucomicrobia;D_2__Verrucomicrobiae;D_3__Pedosphaerales;D_4__Pedosphaeraceae;	metagenome
0,04%	0,08%	0,07%	0,15%	0,09%	0,09%	0,13%	0,12%	D_0__Bacteria;D_1__Proteobacteria;D_2__Alphaproteobacteria;D_3__Rhodospirillales;D_4__uncultured;	uncultured bacterium
0,18%	0,11%	0,07%	0,07%	0,11%	0,12%	0,10%	0,06%	D_0__Bacteria;D_1__Proteobacteria;D_2__Alphaproteobacteria;D_3__Rhizobiales;D_4__Rhizobiales Incertae Sedis;	Nordella
0,10%	0,05%	0,16%	0,05%	0,14%	0,09%	0,16%	0,08%	D_0__Bacteria;D_1__Acidobacteria;D_2__Subgroup 17;Other;Other;Other	
0,03%	0,03%	0,04%	0,03%	0,27%	0,14%	0,18%	0,12%	D_0__Bacteria;D_1__Verrucomicrobia;D_2__Verrucomicrobiae;D_3__Pedosphaerales;D_4__Pedosphaeraceae;Other	
0,10%	0,09%	0,11%	0,13%	0,08%	0,08%	0,06%	0,09%	D_0__Bacteria;D_1__Proteobacteria;D_2__Alphaproteobacteria;D_3__Rhizobiales;Other;Other	
0,03%	0,13%	0,12%	0,11%	0,04%	0,14%	0,08%	0,10%	D_0__Bacteria;D_1__Proteobacteria;D_2__Gammaproteobacteria;D_3__Xanthomonadales;D_4__Xanthomonadaceae;	Lysobacter
0,03%	0,12%	0,10%	0,12%	0,03%	0,11%	0,16%	0,09%	D_0__Bacteria;D_1__Proteobacteria;D_2__Alphaproteobacteria;D_3__Rhizobiales;D_4__Rhizobiaceae;	Phyllobacterium
0,14%	0,19%	0,12%	0,11%	0,04%	0,05%	0,04%	0,06%	D_0__Bacteria;D_1__Chloroflexi;D_2__KD4-96;Other;Other;Other	
0,09%	0,06%	0,13%	0,02%	0,13%	0,06%	0,31%	0,04%	D_0__Bacteria;D_1__Acidobacteria;D_2__Subgroup 22;D_3__uncultured bacterium;D_4__;	
0,03%	0,04%	0,03%	0,12%	0,19%	0,12%	0,09%	0,11%	D_0__Bacteria;D_1__Acidobacteria;D_2__Blastocatellia (Subgroup 4);D_3__Blastocatellales;D_4__Blastocatellaceae;	Stenotrophobacter
0,02%	0,04%	0,07%	0,03%	0,17%	0,16%	0,17%	0,15%	D_0__Bacteria;D_1__Proteobacteria;D_2__Deltaproteobacteria;D_3__Myxococcales;D_4__BIrii41;	metagenome
0,01%	0,01%	0,03%	0,24%	0,03%	0,04%	0,10%	0,12%	D_0__Bacteria;D_1__Acidobacteria;D_2__Acidobacteriia;D_3__Acidobacteriales;D_4__Acidobacteriaceae (Subgroup 1);	Edaphobacter
0,03%	0,07%	0,06%	0,12%	0,12%	0,13%	0,10%	0,08%	D_0__Bacteria;D_1__Proteobacteria;D_2__Gammaproteobacteria;D_3__Diplorickettsiales;D_4__Diplorickettsiaceae;	Aquicella
0,15%	0,17%	0,13%	0,12%	0,02%	0,03%	0,03%	0,05%	D_0__Bacteria;D_1__Proteobacteria;D_2__Alphaproteobacteria;D_3__Tistrellales;D_4__Geminicoccaceae;	Candidatus Alysiosphaera
0,11%	0,07%	0,07%	0,05%	0,18%	0,11%	0,11%	0,07%	D_0__Bacteria;D_1__Proteobacteria;D_2__Deltaproteobacteria;D_3__Myxococcales;D_4__Polyangiaceae;	Pajaroellobacter
0,01%	0,01%	0,02%	0,04%	0,21%	0,31%	0,09%	0,09%	D_0__Bacteria;D_1__Bacteroidetes;D_2__Bacteroidia;D_3__Sphingobacteriales;D_4__env,OPS 17;	uncultured bacterium
0,02%	0,10%	0,06%	0,11%	0,08%	0,15%	0,06%	0,11%	D_0__Bacteria;D_1__Bacteroidetes;D_2__Bacteroidia;D_3__Cytophagales;D_4__Microscillaceae;	Chryseolinea
0,03%	0,23%	0,07%	0,03%	0,03%	0,21%	0,07%	0,10%	D_0__Bacteria;D_1__Proteobacteria;D_2__Gammaproteobacteria;D_3__Xanthomonadales;D_4__Rhodanobacteraceae;	uncultured
0,11%	0,30%	0,10%	0,04%	0,04%	0,12%	0,03%	0,03%	D_0__Bacteria;D_1__Proteobacteria;D_2__Alphaproteobacteria;D_3__Rhizobiales;D_4__Beijerinckiaceae;	Microvirga
0,26%	0,11%	0,11%	0,05%	0,08%	0,05%	0,04%	0,03%	D_0__Bacteria;D_1__Actinobacteria;D_2__Thermoleophilia;D_3__Solirubrobacterales;D_4__67-14;Other	
0,05%	0,19%	0,07%	0,09%	0,04%	0,12%	0,04%	0,10%	D_0__Bacteria;D_1__Actinobacteria;D_2__Actinobacteria;D_3__Micrococcales;D_4__Microbacteriaceae;Other	
0,40%	0,06%	0,08%	0,02%	0,09%	0,05%	0,02%	0,02%	D_0__Bacteria;D_1__Actinobacteria;D_2__Thermoleophilia;D_3__Solirubrobacterales;D_4__Solirubrobacteraceae;	uncultured
0,09%	0,01%	0,09%	0,15%	0,03%	0,02%	0,07%	0,14%	D_0__Bacteria;D_1__Proteobacteria;D_2__Gammaproteobacteria;D_3__Betaproteobacteriales;D_4__A21b;	uncultured beta proteobacterium
0,04%	0,15%	0,08%	0,09%	0,10%	0,10%	0,04%	0,07%	D_0__Bacteria;D_1__Proteobacteria;D_2__Gammaproteobacteria;D_3__Betaproteobacteriales;D_4__Burkholderiaceae;	Ramlibacter
0,04%	0,02%	0,05%	0,06%	0,15%	0,10%	0,16%	0,13%	D_0__Bacteria;D_1__Proteobacteria;D_2__Alphaproteobacteria;D_3__Rhodospirillales;D_4__Rhodospirillaceae;	uncultured
0,11%	0,22%	0,10%	0,04%	0,11%	0,04%	0,04%	0,04%	D_0__Bacteria;D_1__Patescibacteria;D_2__Parcubacteria;D_3__Candidatus Kaiserbacteria;D_4__uncultured bacterium;	
0,03%	0,04%	0,08%	0,05%	0,18%	0,13%	0,12%	0,08%	D_0__Bacteria;D_1__Planctomycetes;D_2__Phycisphaerae;D_3__Phycisphaerales;D_4__Phycisphaeraceae;	SM1A02
0,06%	0,12%	0,10%	0,12%	0,05%	0,10%	0,04%	0,04%	D_0__Bacteria;D_1__Bacteroidetes;D_2__Bacteroidia;D_3__Chitinophagales;D_4__Chitinophagaceae;	Flavitalea
0,06%	0,07%	0,09%	0,09%	0,07%	0,08%	0,07%	0,07%	D_0__Bacteria;D_1__Proteobacteria;D_2__Gammaproteobacteria;D_3__Betaproteobacteriales;D_4__Burkholderiaceae;	Massilia
0,02%	0,09%	0,04%	0,07%	0,06%	0,19%	0,07%	0,08%	D_0__Bacteria;D_1__Acidobacteria;D_2__Acidobacteriia;D_3__Solibacterales;D_4__Solibacteraceae (Subgroup 3);	Paludibaculum
0,14%	0,08%	0,11%	0,04%	0,11%	0,08%	0,08%	0,03%	D_0__Bacteria;D_1__Planctomycetes;D_2__Planctomycetacia;D_3__Gemmatales;D_4__Gemmataceae;	uncultured
0,06%	0,02%	0,07%	0,11%	0,05%	0,04%	0,15%	0,09%	D_0__Bacteria;D_1__Proteobacteria;D_2__Gammaproteobacteria;D_3__Betaproteobacteriales;D_4__Burkholderiaceae;	Comamonas
0,15%	0,24%	0,10%	0,04%	0,03%	0,06%	0,01%	0,02%	D_0__Bacteria;D_1__Actinobacteria;D_2__Actinobacteria;D_3__Propionibacteriales;D_4__Nocardioidaceae;	Marmoricola
0,01%	0,01%	0,01%	0,01%	0,21%	0,17%	0,15%	0,11%	D_0__Bacteria;D_1__Verrucomicrobia;D_2__Verrucomicrobiae;D_3__Opitutales;D_4__Opitutaceae;	Lacunisphaera
0,06%	0,08%	0,09%	0,07%	0,10%	0,07%	0,10%	0,06%	D_0__Bacteria;D_1__Proteobacteria;D_2__Deltaproteobacteria;D_3__Oligoflexales;D_4__0319-6G20;	uncultured bacterium
0,00%	0,01%	0,01%	0,14%	0,07%	0,17%	0,09%	0,06%	D_0__Bacteria;D_1__Bacteroidetes;D_2__Bacteroidia;D_3__Chitinophagales;D_4__Chitinophagaceae;	Chitinophaga
0,03%	0,02%	0,09%	0,04%	0,12%	0,08%	0,16%	0,11%	D_0__Bacteria;D_1__Acidobacteria;D_2__Subgroup 5;D_3__uncultured Acidobacteria bacterium;D_4__;	
0,09%	0,06%	0,15%	0,04%	0,06%	0,04%	0,15%	0,05%	D_0__Bacteria;D_1__Acidobacteria;D_2__Subgroup 17;D_3__uncultured Acidobacteria bacterium;D_4__;	
0,21%	0,01%	0,11%	0,01%	0,11%	0,02%	0,15%	0,02%	D_0__Bacteria;D_1__Nitrospirae;D_2__Nitrospira;D_3__Nitrospirales;D_4__Nitrospiraceae;	Nitrospira
0,03%	0,03%	0,04%	0,08%	0,08%	0,07%	0,11%	0,13%	D_0__Bacteria;D_1__Proteobacteria;D_2__Alphaproteobacteria;D_3__uncultured;D_4__metagenome;	
0,08%	0,10%	0,10%	0,07%	0,04%	0,11%	0,05%	0,03%	D_0__Bacteria;D_1__Chloroflexi;D_2__Chloroflexia;D_3__Thermomicrobiales;D_4__JG30-KF-CM45;	uncultured bacterium
0,07%	0,05%	0,10%	0,06%	0,14%	0,07%	0,05%	0,04%	D_0__Bacteria;D_1__Planctomycetes;D_2__Phycisphaerae;D_3__Tepidisphaerales;D_4__WD2101 soil group;Other	
0,11%	0,06%	0,08%	0,05%	0,11%	0,07%	0,07%	0,05%	D_0__Bacteria;D_1__Chloroflexi;D_2__TK10;D_3__uncultured bacterium;D_4__;	
0,07%	0,02%	0,07%	0,06%	0,10%	0,02%	0,15%	0,07%	D_0__Bacteria;D_1__Acidobacteria;D_2__Acidobacteriia;D_3__Subgroup 2;D_4__uncultured bacterium;	
0,06%	0,03%	0,06%	0,10%	0,06%	0,06%	0,08%	0,08%	D_0__Bacteria;D_1__Actinobacteria;D_2__Actinobacteria;D_3__Micromonosporales;D_4__Micromonosporaceae;	Luedemannella
0,04%	0,12%	0,11%	0,07%	0,04%	0,06%	0,07%	0,04%	D_0__Bacteria;D_1__Proteobacteria;D_2__Deltaproteobacteria;D_3__SAR324 clade(Marine group B);D_4__uncultured bacterium;	
0,05%	0,10%	0,08%	0,09%	0,05%	0,05%	0,07%	0,04%	D_0__Bacteria;D_1__Proteobacteria;D_2__Alphaproteobacteria;D_3__Rickettsiales;D_4__SM2D12;	metagenome
0,08%	0,06%	0,09%	0,03%	0,10%	0,07%	0,09%	0,06%	D_0__Bacteria;D_1__Proteobacteria;D_2__Deltaproteobacteria;D_3__Bdellovibrionales;D_4__Bdellovibrionaceae;	OM27 clade
0,08%	0,04%	0,06%	0,12%	0,03%	0,04%	0,04%	0,07%	D_0__Bacteria;D_1__Actinobacteria;D_2__Actinobacteria;D_3__Micromonosporales;D_4__Micromonosporaceae;Other	
0,18%	0,03%	0,04%	0,03%	0,13%	0,01%	0,06%	0,07%	D_0__Bacteria;D_1__Acidobacteria;D_2__Acidobacteriia;D_3__Acidobacteriales;D_4__uncultured;	uncultured bacterium
0,08%	0,04%	0,07%	0,12%	0,03%	0,04%	0,04%	0,06%	D_0__Bacteria;D_1__Proteobacteria;D_2__Alphaproteobacteria;D_3__Rhizobiales;D_4__Xanthobacteraceae;	Afipia
0,03%	0,10%	0,08%	0,05%	0,08%	0,07%	0,07%	0,05%	D_0__Bacteria;D_1__Proteobacteria;D_2__Alphaproteobacteria;D_3__Rickettsiales;D_4__SM2D12;	uncultured bacterium
0,04%	0,02%	0,06%	0,04%	0,09%	0,07%	0,14%	0,09%	D_0__Bacteria;D_1__Elusimicrobia;D_2__Lineage IIa;D_3__uncultured bacterium;D_4__;	
0,04%	0,03%	0,05%	0,13%	0,03%	0,05%	0,04%	0,08%	D_0__Bacteria;D_1__Actinobacteria;D_2__Actinobacteria;D_3__Micromonosporales;D_4__Micromonosporaceae;	Asanoa
0,04%	0,03%	0,05%	0,06%	0,10%	0,07%	0,08%	0,09%	D_0__Bacteria;D_1__Proteobacteria;D_2__Alphaproteobacteria;D_3__Rhizobiales;D_4__KF-JG30-B3;Other	
0,04%	0,04%	0,06%	0,02%	0,16%	0,11%	0,07%	0,06%	D_0__Bacteria;D_1__Bacteroidetes;D_2__Bacteroidia;D_3__Sphingobacteriales;D_4__AKYH767;	uncultured bacterium
0,12%	0,09%	0,08%	0,05%	0,05%	0,04%	0,04%	0,06%	D_0__Bacteria;D_1__Actinobacteria;D_2__Acidimicrobiia;D_3__IMCC26256;D_4__uncultured Acidimicrobidae bacterium;	
0,02%	0,05%	0,03%	0,16%	0,02%	0,08%	0,02%	0,03%	D_0__Bacteria;D_1__Proteobacteria;D_2__Gammaproteobacteria;D_3__Xanthomonadales;D_4__Xanthomonadaceae;	Luteimonas
0,15%	0,07%	0,09%	0,05%	0,06%	0,03%	0,03%	0,03%	D_0__Bacteria;D_1__Chloroflexi;D_2__KD4-96;D_3__uncultured Chloroflexi bacterium;D_4__;	
0,02%	0,05%	0,04%	0,13%	0,03%	0,09%	0,03%	0,05%	D_0__Bacteria;D_1__Proteobacteria;D_2__Alphaproteobacteria;D_3__Caulobacterales;D_4__Caulobacteraceae;	Brevundimonas
0,05%	0,04%	0,05%	0,06%	0,07%	0,09%	0,08%	0,06%	D_0__Bacteria;D_1__Actinobacteria;D_2__Actinobacteria;D_3__Pseudonocardiales;D_4__Pseudonocardiaceae;	Amycolatopsis
0,08%	0,02%	0,09%	0,01%	0,14%	0,04%	0,12%	0,04%	D_0__Bacteria;D_1__Latescibacteria;D_2__uncultured bacterium;D_3__;D_4__;	

(C)

AI1c	AI2c	AI3c	AI4c	AII1c	AII2c	AII3c	AII4c	#OTU ID	
19,8%	13,9%	16,7%	20,1%	16,4%	17,6%	18,3%	16,7%	Unassigned;Other;Other;Other;Other;Other	
4,64%	1,7%	0,77%	2,06%	5,33%	1,71%	2,48%	5,51%	D_0__Bacteria;D_1__Verrucomicrobia;D_2__Verrucomicrobiae;D_3__Chthoniobacterales;D_4__Chthoniobacteraceae;	Candidatus Udaeobacter
2,07%	4,1%	2,91%	1,71%	2,30%	2,76%	1,72%	4,11%	D_0__Bacteria;D_1__Proteobacteria;D_2__Alphaproteobacteria;D_3__Sphingomonadales;D_4__Sphingomonadaceae;	Sphingomonas
1,87%	2,6%	3,46%	1,28%	2,09%	2,07%	2,89%	1,28%	D_0__Bacteria;D_1__Acidobacteria;D_2__Subgroup 6;D_3__uncultured bacterium;D_4__;	
2,76%	2,0%	3,40%	2,25%	1,97%	1,63%	1,93%	2,04%	D_0__Bacteria;D_1__Proteobacteria;D_2__Alphaproteobacteria;D_3__Rhizobiales;D_4__Xanthobacteraceae;	uncultured
1,60%	2,9%	2,96%	1,33%	1,59%	2,37%	2,37%	1,40%	D_0__Bacteria;D_1__Acidobacteria;D_2__Subgroup 6;D_3__uncultured Acidobacteria bacterium;D_4__;	
0,55%	6,1%	1,33%	0,11%	1,39%	1,50%	1,35%	0,77%	D_0__Bacteria;D_1__Firmicutes;D_2__Bacilli;D_3__Bacillales;D_4__Bacillaceae;	Bacillus
3,00%	1,1%	1,27%	1,37%	2,52%	2,49%	2,26%	2,27%	D_0__Bacteria;D_1__Planctomycetes;D_2__Phycisphaerae;D_3__Tepidisphaerales;D_4__WD2101 soil group;	uncultured bacterium
4,61%	0,7%	1,83%	2,40%	1,64%	1,09%	0,97%	1,25%	D_0__Bacteria;D_1__Actinobacteria;D_2__Thermoleophilia;D_3__Gaiellales;D_4__uncultured;	uncultured bacterium
3,66%	1,2%	2,05%	2,48%	1,39%	1,33%	0,72%	0,99%	D_0__Bacteria;D_1__Actinobacteria;D_2__Thermoleophilia;D_3__Solirubrobacterales;D_4__67-14;	uncultured bacterium
0,85%	0,4%	1,78%	3,10%	1,60%	1,79%	3,12%	1,33%	D_0__Bacteria;D_1__Proteobacteria;D_2__Gammaproteobacteria;D_3__Betaproteobacteriales;D_4__Nitrosomonadaceae;	MND1
2,03%	0,6%	1,39%	2,41%	1,96%	0,95%	2,30%	1,76%	D_0__Bacteria;D_1__Gemmatimonadetes;D_2__Gemmatimonadetes;D_3__Gemmatimonadales;D_4__Gemmatimonadaceae;	uncultured
1,62%	1,7%	1,88%	1,08%	1,29%	1,14%	1,09%	1,34%	D_0__Bacteria;D_1__Proteobacteria;D_2__Alphaproteobacteria;D_3__Rhizobiales;D_4__Xanthobacteraceae;	Bradyrhizobium
2,25%	1,1%	2,19%	1,33%	1,36%	1,08%	0,60%	0,76%	D_0__Bacteria;D_1__Chloroflexi;D_2__KD4-96;D_3__uncultured bacterium;D_4__;	
1,06%	1,7%	1,89%	0,70%	1,03%	1,17%	1,41%	0,82%	D_0__Bacteria;D_1__Acidobacteria;D_2__Subgroup 6;Other;Other;Other	
0,60%	1,1%	0,72%	2,37%	1,15%	1,09%	0,75%	1,90%	D_0__Bacteria;D_1__Proteobacteria;D_2__Alphaproteobacteria;D_3__Micropepsales;D_4__Micropepsaceae;	uncultured
0,56%	1,8%	0,37%	1,22%	1,32%	0,99%	0,32%	1,21%	D_0__Bacteria;D_1__Patescibacteria;D_2__Saccharimonadia;D_3__Saccharimonadales;D_4__uncultured bacterium;	
0,79%	1,2%	1,31%	0,64%	1,11%	1,56%	1,05%	0,69%	D_0__Bacteria;D_1__Proteobacteria;D_2__Gammaproteobacteria;D_3__Betaproteobacteriales;D_4__Nitrosomonadaceae;	Ellin6067
0,53%	1,2%	0,90%	1,17%	1,06%	1,24%	0,89%	1,30%	D_0__Bacteria;D_1__Bacteroidetes;D_2__Bacteroidia;D_3__Chitinophagales;D_4__Chitinophagaceae;	uncultured
0,21%	1,0%	0,83%	0,88%	0,84%	1,41%	1,47%	1,45%	D_0__Bacteria;D_1__Proteobacteria;D_2__Alphaproteobacteria;D_3__Dongiales;D_4__Dongiaceae;	Dongia
0,70%	1,1%	0,78%	1,67%	0,46%	0,94%	0,38%	0,99%	D_0__Bacteria;D_1__Actinobacteria;D_2__Actinobacteria;D_3__Corynebacteriales;D_4__Mycobacteriaceae;	Mycobacterium
0,36%	0,7%	0,87%	1,15%	0,89%	0,70%	0,82%	1,36%	D_0__Bacteria;D_1__Acidobacteria;D_2__Acidobacteriia;D_3__Solibacterales;D_4__Solibacteraceae (Subgroup 3);	Bryobacter
0,41%	0,3%	0,84%	0,92%	1,17%	0,91%	1,24%	1,31%	D_0__Bacteria;D_1__Acidobacteria;D_2__Acidobacteriia;D_3__Solibacterales;D_4__Solibacteraceae (Subgroup 3);	Candidatus Solibacter
1,46%	0,5%	1,13%	0,89%	0,93%	0,72%	0,76%	0,49%	D_0__Bacteria;D_1__Actinobacteria;D_2__Acidimicrobiia;D_3__IMCC26256;D_4__uncultured bacterium;	
1,98%	1,1%	0,89%	0,83%	0,39%	0,52%	0,18%	0,22%	D_0__Bacteria;D_1__Actinobacteria;D_2__Thermoleophilia;D_3__Solirubrobacterales;D_4__Solirubrobacteraceae;	Solirubrobacter
0,78%	1,3%	0,84%	1,08%	0,27%	0,78%	0,23%	0,25%	D_0__Bacteria;D_1__Actinobacteria;D_2__Actinobacteria;D_3__Propionibacteriales;D_4__Nocardioidaceae;	Nocardioides
0,33%	0,4%	0,84%	0,86%	0,79%	1,04%	1,48%	0,62%	D_0__Bacteria;D_1__Proteobacteria;D_2__Gammaproteobacteria;D_3__Betaproteobacteriales;D_4__TRA3-20;	uncultured bacterium
0,69%	0,2%	0,13%	2,22%	0,50%	0,21%	0,11%	2,54%	D_0__Bacteria;D_1__Actinobacteria;D_2__Actinobacteria;D_3__Frankiales;D_4__Acidothermaceae;	Acidothermus
0,66%	1,1%	0,98%	0,44%	0,66%	0,60%	0,59%	0,54%	D_0__Bacteria;D_1__Proteobacteria;D_2__Alphaproteobacteria;D_3__Sphingomonadales;D_4__Sphingomonadaceae;	uncultured
1,09%	0,6%	1,13%	0,16%	0,91%	0,57%	1,05%	0,64%	D_0__Bacteria;D_1__Acidobacteria;D_2__Blastocatellia (Subgroup 4);D_3__Pyrinomonadales;D_4__Pyrinomonadaceae;	RB41
0,07%	0,5%	0,33%	0,56%	1,06%	1,62%	1,31%	0,70%	D_0__Bacteria;D_1__Bacteroidetes;D_2__Bacteroidia;D_3__Cytophagales;D_4__Microscillaceae;	uncultured
0,54%	0,3%	0,59%	0,50%	1,11%	1,23%	1,20%	0,61%	D_0__Bacteria;D_1__Proteobacteria;D_2__Deltaproteobacteria;D_3__Myxococcales;D_4__Haliangiaceae;	Haliangium
0,32%	0,7%	0,69%	0,58%	0,69%	1,04%	0,87%	0,78%	D_0__Bacteria;D_1__Proteobacteria;D_2__Alphaproteobacteria;D_3__Reyranellales;D_4__Reyranellaceae;	Reyranella
0,40%	1,1%	0,40%	0,92%	0,28%	0,82%	0,34%	0,52%	D_0__Bacteria;D_1__Actinobacteria;D_2__Actinobacteria;D_3__Streptomycetales;D_4__Streptomycetaceae;	Streptomyces
0,37%	0,9%	0,35%	1,41%	0,28%	0,41%	0,25%	1,04%	D_0__Bacteria;D_1__Proteobacteria;D_2__Alphaproteobacteria;D_3__Acetobacterales;D_4__Acetobacteraceae;	uncultured
0,23%	0,9%	0,21%	0,14%	1,00%	0,89%	0,82%	0,52%	D_0__Bacteria;D_1__Verrucomicrobia;D_2__Verrucomicrobiae;D_3__Chthoniobacterales;D_4__Chthoniobacteraceae;	Chthoniobacter
0,21%	0,8%	0,38%	0,45%	0,77%	0,80%	0,44%	0,76%	D_0__Bacteria;D_1__Proteobacteria;D_2__Alphaproteobacteria;D_3__Caulobacterales;D_4__Caulobacteraceae;	Phenylobacterium
0,22%	1,0%	0,20%	1,01%	0,21%	0,68%	0,18%	0,50%	D_0__Bacteria;D_1__Actinobacteria;D_2__Actinobacteria;D_3__Propionibacteriales;D_4__Nocardioidaceae;	Kribbella
0,29%	0,5%	0,28%	0,97%	0,34%	0,33%	0,17%	1,53%	D_0__Bacteria;D_1__Proteobacteria;D_2__Alphaproteobacteria;D_3__Rhizobiales;D_4__Xanthobacteraceae;	Pseudolabrys
0,57%	0,3%	0,44%	0,24%	0,72%	0,95%	0,76%	0,32%	D_0__Bacteria;D_1__Actinobacteria;D_2__Acidimicrobiia;D_3__Microtrichales;D_4__uncultured;	uncultured bacterium
1,15%	0,3%	0,95%	0,51%	0,42%	0,37%	0,35%	0,23%	D_0__Bacteria;D_1__Actinobacteria;D_2__Thermoleophilia;D_3__Gaiellales;D_4__Gaiellaceae;	Gaiella
0,69%	0,3%	0,56%	0,61%	0,49%	0,25%	0,35%	0,87%	D_0__Bacteria;D_1__Proteobacteria;D_2__Alphaproteobacteria;D_3__Elsterales;D_4__uncultured;	uncultured bacterium
0,49%	0,5%	0,64%	0,32%	0,46%	0,43%	0,61%	0,40%	D_0__Bacteria;D_1__Proteobacteria;D_2__Gammaproteobacteria;D_3__Betaproteobacteriales;D_4__SC-I-84;	uncultured bacterium
0,18%	0,4%	0,38%	0,47%	0,49%	0,78%	0,53%	0,68%	D_0__Bacteria;D_1__Proteobacteria;D_2__Gammaproteobacteria;D_3__Gammaproteobacteria Incertae Sedis;D_4__Unknown Family;	Acidibacter
0,20%	0,1%	0,30%	0,37%	0,84%	0,70%	0,93%	0,54%	D_0__Bacteria;D_1__Verrucomicrobia;D_2__Verrucomicrobiae;D_3__Pedosphaerales;D_4__Pedosphaeraceae;	uncultured bacterium
0,50%	0,6%	0,60%	0,30%	0,49%	0,45%	0,40%	0,30%	D_0__Bacteria;D_1__Proteobacteria;D_2__Alphaproteobacteria;D_3__Rhizobiales;D_4__Xanthobacteraceae;	Rhodoplanes
1,31%	0,3%	0,54%	0,59%	0,33%	0,22%	0,14%	0,28%	D_0__Bacteria;D_1__Actinobacteria;D_2__Thermoleophilia;D_3__Solirubrobacterales;D_4__Solirubrobacteraceae;	Conexibacter
0,33%	0,3%	0,24%	0,58%	0,70%	0,53%	0,46%	0,55%	D_0__Bacteria;D_1__Gemmatimonadetes;D_2__Gemmatimonadetes;D_3__Gemmatimonadales;D_4__Gemmatimonadaceae;	Gemmatimonas
0,03%	1,2%	0,07%	0,08%	0,18%	0,70%	0,15%	0,28%	D_0__Bacteria;D_1__Bacteroidetes;D_2__Bacteroidia;D_3__Flavobacteriales;D_4__Flavobacteriaceae;	Flavobacterium
0,63%	0,1%	1,18%	0,03%	0,43%	0,11%	0,89%	0,14%	D_0__Bacteria;D_1__Rokubacteria;D_2__NC10;D_3__Rokubacteriales;D_4__uncultured bacterium;	
0,15%	0,4%	0,30%	0,42%	0,27%	0,43%	0,33%	0,38%	D_0__Bacteria;D_1__Proteobacteria;D_2__Alphaproteobacteria;D_3__Rhizobiales;D_4__Rhizobiaceae;	Mesorhizobium
0,18%	0,5%	0,25%	0,37%	0,32%	0,34%	0,19%	0,24%	D_0__Bacteria;D_1__Proteobacteria;D_2__Alphaproteobacteria;D_3__Rhizobiales;D_4__Devosiaceae;	Devosia
0,19%	0,3%	0,28%	0,23%	0,47%	0,44%	0,37%	0,25%	D_0__Bacteria;D_1__Bacteroidetes;D_2__Bacteroidia;D_3__Chitinophagales;D_4__Chitinophagaceae;	Terrimonas
0,10%	0,3%	0,06%	0,39%	0,23%	0,86%	0,13%	0,43%	D_0__Bacteria;D_1__Proteobacteria;D_2__Gammaproteobacteria;D_3__Xanthomonadales;D_4__Rhodanobacteraceae;	Rhodanobacter
0,26%	0,2%	0,30%	0,46%	0,25%	0,24%	0,26%	0,54%	D_0__Bacteria;D_1__Proteobacteria;D_2__Alphaproteobacteria;D_3__Elsterales;D_4__uncultured;Other	
0,32%	0,3%	0,52%	0,33%	0,15%	0,31%	0,18%	0,13%	D_0__Bacteria;D_1__Actinobacteria;D_2__Acidimicrobiia;D_3__Microtrichales;D_4__Ilumatobacteraceae;	uncultured
0,17%	0,2%	0,54%	0,24%	0,14%	0,17%	0,55%	0,35%	D_0__Bacteria;D_1__Proteobacteria;D_2__Deltaproteobacteria;D_3__Myxococcales;D_4__bacteriap25;	uncultured bacterium
0,03%	0,7%	0,05%	0,18%	0,20%	0,23%	0,09%	0,35%	D_0__Bacteria;D_1__Bacteroidetes;D_2__Bacteroidia;D_3__Sphingobacteriales;D_4__Sphingobacteriaceae;	Mucilaginibacter
0,15%	0,5%	0,20%	0,19%	0,38%	0,21%	0,19%	0,29%	D_0__Bacteria;D_1__Proteobacteria;D_2__Alphaproteobacteria;D_3__Caulobacterales;D_4__Caulobacteraceae;	uncultured
0,08%	0,3%	0,19%	0,28%	0,23%	0,29%	0,28%	0,56%	D_0__Bacteria;D_1__Proteobacteria;D_2__Gammaproteobacteria;D_3__Betaproteobacteriales;D_4__Burkholderiaceae;	Burkholderia-Caballeronia-Paraburkholderia
0,89%	0,1%	0,41%	0,31%	0,28%	0,13%	0,14%	0,19%	D_0__Bacteria;D_1__Actinobacteria;D_2__Thermoleophilia;D_3__Gaiellales;D_4__uncultured;Other	
0,17%	0,2%	0,34%	0,43%	0,22%	0,21%	0,34%	0,44%	D_0__Bacteria;D_1__Proteobacteria;D_2__Alphaproteobacteria;D_3__uncultured;D_4__uncultured bacterium;	
0,19%	0,3%	0,29%	0,32%	0,28%	0,48%	0,21%	0,10%	D_0__Bacteria;D_1__Actinobacteria;D_2__Acidimicrobiia;D_3__Microtrichales;D_4__Iamiaceae;	Iamia
0,58%	0,2%	0,17%	0,12%	0,46%	0,21%	0,29%	0,16%	D_0__Bacteria;D_1__Acidobacteria;D_2__Holophagae;D_3__Subgroup 7;D_4__uncultured bacterium;	
0,11%	0,1%	0,28%	0,48%	0,21%	0,26%	0,53%	0,36%	D_0__Bacteria;D_1__Proteobacteria;D_2__Gammaproteobacteria;D_3__CCD24;D_4__uncultured bacterium;	
0,38%	0,1%	0,22%	0,58%	0,23%	0,26%	0,09%	0,24%	D_0__Bacteria;D_1__Actinobacteria;D_2__Actinobacteria;D_3__Frankiales;D_4__Frankiaceae;	Jatrophihabitans
0,33%	0,2%	0,53%	0,18%	0,16%	0,21%	0,26%	0,11%	D_0__Bacteria;D_1__Proteobacteria;D_2__Alphaproteobacteria;D_3__Rhizobiales;D_4__Hyphomicrobiaceae;	Pedomicrobium
0,37%	0,1%	0,21%	0,12%	0,40%	0,31%	0,30%	0,23%	D_0__Bacteria;D_1__Actinobacteria;D_2__Acidimicrobiia;D_3__uncultured;D_4__uncultured bacterium;	
0,12%	0,4%	0,15%	0,19%	0,26%	0,32%	0,27%	0,15%	D_0__Bacteria;D_1__Bacteroidetes;D_2__Bacteroidia;D_3__Chitinophagales;D_4__Chitinophagaceae;	Ferruginibacter
0,09%	0,2%	0,29%	0,25%	0,15%	0,31%	0,42%	0,23%	D_0__Bacteria;D_1__Proteobacteria;D_2__Gammaproteobacteria;D_3__Steroidobacterales;D_4__Steroidobacteraceae;	uncultured
0,17%	0,4%	0,16%	0,13%	0,37%	0,34%	0,14%	0,13%	D_0__Bacteria;D_1__Proteobacteria;D_2__Gammaproteobacteria;D_3__Xanthomonadales;D_4__Xanthomonadaceae;	Arenimonas
0,11%	0,3%	0,26%	0,13%	0,21%	0,40%	0,30%	0,20%	D_0__Bacteria;D_1__Proteobacteria;D_2__Gammaproteobacteria;D_3__Betaproteobacteriales;D_4__Burkholderiaceae;	Rhizobacter
0,12%	0,4%	0,02%	0,37%	0,06%	0,07%	0,02%	0,70%	D_0__Bacteria;D_1__Acidobacteria;D_2__Acidobacteriia;D_3__Acidobacteriales;D_4__Acidobacteriaceae (Subgroup 1);	Acidipila
0,23%	0,3%	0,50%	0,19%	0,15%	0,12%	0,28%	0,11%	D_0__Bacteria;D_1__Acidobacteria;D_2__Subgroup 17;D_3__uncultured bacterium;D_4__;	
0,10%	0,4%	0,05%	0,15%	0,21%	0,14%	0,08%	0,57%	D_0__Bacteria;D_1__Acidobacteria;D_2__Acidobacteriia;D_3__Acidobacteriales;D_4__Acidobacteriaceae (Subgroup 1);	Edaphobacter
0,17%	0,1%	0,26%	0,17%	0,29%	0,23%	0,40%	0,24%	D_0__Bacteria;D_1__Proteobacteria;D_2__Alphaproteobacteria;D_3__Rhizobiales;D_4__KF-JG30-B3;	uncultured bacterium
0,34%	0,2%	0,29%	0,30%	0,20%	0,23%	0,11%	0,12%	D_0__Bacteria;D_1__Actinobacteria;D_2__Acidimicrobiia;D_3__IMCC26256;Other;Other	
0,11%	0,3%	0,25%	0,18%	0,20%	0,26%	0,23%	0,17%	D_0__Bacteria;D_1__Proteobacteria;D_2__Gammaproteobacteria;D_3__Betaproteobacteriales;D_4__Burkholderiaceae;Other	

0,21%	0,4%	0,14%	0,20%	0,15%	0,23%	0,13%	0,13%	D_0__Bacteria;D_1__Actinobacteria;D_2__Actinobacteria;D_3__Pseudonocardiales;D_4__Pseudonocardiaceae;	Pseudonocardia
0,06%	0,3%	0,14%	0,15%	0,14%	0,30%	0,16%	0,23%	D_0__Bacteria;D_1__Proteobacteria;D_2__Alphaproteobacteria;D_3__Rhizobiales;D_4__Rhizobiaceae;	Allorhizobium-Neorhizobium-Pararhizobium-Rhizobium
0,69%	0,1%	0,25%	0,04%	0,24%	0,15%	0,16%	0,04%	D_0__Bacteria;D_1__Actinobacteria;D_2__MB-A2-108;D_3__uncultured bacterium;D_4__;	
0,16%	0,1%	0,24%	0,07%	0,23%	0,23%	0,46%	0,18%	D_0__Bacteria;D_1__Proteobacteria;D_2__Deltaproteobacteria;D_3__Desulfarculales;D_4__Desulfarculaceae;	uncultured
0,22%	0,1%	0,30%	0,13%	0,23%	0,21%	0,31%	0,08%	D_0__Bacteria;D_1__Actinobacteria;D_2__Acidimicrobiia;D_3__Microtrichales;D_4__Ilumatobacteraceae;	CL500-29 marine group
0,22%	0,2%	0,30%	0,14%	0,18%	0,14%	0,30%	0,14%	D_0__Bacteria;D_1__Proteobacteria;D_2__Gammaproteobacteria;D_3__Betaproteobacteriales;D_4__SC-I-84;Other	
0,41%	0,0%	0,08%	0,11%	0,45%	0,05%	0,14%	0,43%	D_0__Bacteria;D_1__Acidobacteria;D_2__Acidobacteriia;D_3__Acidobacteriales;D_4__uncultured;	uncultured bacterium
0,11%	0,3%	0,21%	0,18%	0,13%	0,14%	0,05%	0,14%	D_0__Bacteria;D_1__Proteobacteria;D_2__Alphaproteobacteria;D_3__Sphingomonadales;D_4__Sphingomonadaceae;	Altererythrobacter
0,03%	0,5%	0,03%	0,15%	0,13%	0,09%	0,02%	0,17%	D_0__Bacteria;D_1__Patescibacteria;D_2__Saccharimonadia;D_3__Saccharimonadales;Other;Other	
0,06%	0,2%	0,08%	0,28%	0,17%	0,24%	0,17%	0,28%	D_0__Bacteria;D_1__Proteobacteria;D_2__Gammaproteobacteria;D_3__Xanthomonadales;D_4__Rhodanobacteraceae;	Dokdonella
0,09%	0,2%	0,20%	0,11%	0,16%	0,27%	0,22%	0,11%	D_0__Bacteria;D_1__Proteobacteria;D_2__Gammaproteobacteria;D_3__Betaproteobacteriales;D_4__Burkholderiaceae;	uncultured
0,03%	0,4%	0,06%	0,08%	0,15%	0,22%	0,15%	0,15%	D_0__Bacteria;D_1__Proteobacteria;D_2__Alphaproteobacteria;D_3__Sphingomonadales;D_4__Sphingomonadaceae;	Novosphingobium
0,23%	0,0%	0,11%	0,14%	0,37%	0,14%	0,15%	0,37%	D_0__Bacteria;D_1__Acidobacteria;D_2__Acidobacteriia;D_3__Subgroup 2;D_4__uncultured bacterium;	
0,07%	0,1%	0,19%	0,09%	0,25%	0,22%	0,38%	0,12%	D_0__Bacteria;D_1__Proteobacteria;D_2__Gammaproteobacteria;D_3__Betaproteobacteriales;D_4__Nitrosomonadaceae;	IS-44
0,08%	0,1%	0,06%	0,72%	0,10%	0,07%	0,11%	0,26%	D_0__Bacteria;D_1__Actinobacteria;D_2__Actinobacteria;D_3__Micromonosporales;D_4__Micromonosporaceae;	uncultured
0,17%	0,3%	0,22%	0,05%	0,15%	0,17%	0,14%	0,08%	D_0__Bacteria;D_1__Acidobacteria;D_2__Subgroup 6;D_3__uncultured Acidobacteriales bacterium;D_4__;	
0,02%	0,1%	0,14%	0,02%	0,34%	0,22%	0,30%	0,18%	D_0__Bacteria;D_1__Proteobacteria;D_2__Gammaproteobacteria;D_3__Pseudomonadales;D_4__Pseudomonadaceae;	Pseudomonas
0,15%	0,2%	0,26%	0,14%	0,11%	0,16%	0,23%	0,11%	D_0__Bacteria;D_1__Proteobacteria;D_2__Alphaproteobacteria;D_3__Rhizobiales;D_4__uncultured;	uncultured bacterium
0,11%	0,0%	0,02%	0,48%	0,08%	0,04%	0,03%	0,67%	D_0__Bacteria;D_1__Acidobacteria;D_2__Acidobacteriia;D_3__Acidobacteriales;D_4__Acidobacteriaceae (Subgroup 1);	uncultured
0,08%	0,1%	0,23%	0,12%	0,21%	0,12%	0,29%	0,16%	D_0__Bacteria;D_1__Proteobacteria;D_2__Alphaproteobacteria;D_3__uncultured;Other;Other	
0,35%	0,0%	0,43%	0,03%	0,18%	0,03%	0,38%	0,02%	D_0__Bacteria;D_1__Nitrospirae;D_2__Nitrospira;D_3__Nitrospirales;D_4__Nitrospiraceae;	Nitrospira
0,04%	0,3%	0,13%	0,04%	0,16%	0,29%	0,12%	0,13%	D_0__Bacteria;D_1__Acidobacteria;D_2__Blastocatellia (Subgroup 4);D_3__Blastocatellales;D_4__Blastocatellaceae;	uncultured
0,05%	0,1%	0,13%	0,21%	0,22%	0,22%	0,19%	0,18%	D_0__Bacteria;D_1__Proteobacteria;D_2__Alphaproteobacteria;D_3__Caulobacterales;D_4__Hyphomonadaceae;	Hirschia
0,07%	0,1%	0,14%	0,10%	0,21%	0,24%	0,25%	0,14%	D_0__Bacteria;D_1__Proteobacteria;D_2__Deltaproteobacteria;D_3__Myxococcales;D_4__Sandaracinaceae;	uncultured
0,11%	0,1%	0,21%	0,09%	0,19%	0,12%	0,33%	0,16%	D_0__Bacteria;D_1__Proteobacteria;D_2__Alphaproteobacteria;D_3__Rhizobiales;D_4__Methyloligellaceae;	uncultured
0,12%	0,3%	0,04%	0,12%	0,08%	0,10%	0,07%	0,27%	D_0__Bacteria;D_1__Verrucomicrobia;D_2__Verrucomicrobiae;D_3__Chthoniobacterales;D_4__Xiphinematobacteraceae;	Candidatus Xiphinematobacter
0,16%	0,1%	0,16%	0,18%	0,12%	0,10%	0,12%	0,21%	D_0__Bacteria;D_1__Proteobacteria;D_2__Alphaproteobacteria;D_3__Rhizobiales;D_4__Xanthobacteraceae;Other	
0,03%	0,5%	0,03%	0,01%	0,05%	0,10%	0,02%	0,02%	D_0__Bacteria;D_1__Firmicutes;D_2__Bacilli;D_3__Bacillales;D_4__Planococcaceae;	Lysinibacillus
0,17%	0,2%	0,26%	0,10%	0,07%	0,09%	0,12%	0,12%	D_0__Bacteria;D_1__Proteobacteria;D_2__Alphaproteobacteria;D_3__Sphingomonadales;D_4__Sphingomonadaceae;	Ellin6055
0,05%	0,1%	0,17%	0,16%	0,10%	0,19%	0,19%	0,15%	D_0__Bacteria;D_1__Proteobacteria;D_2__Gammaproteobacteria;D_3__Steroidobacterales;D_4__Steroidobacteraceae;	Steroidobacter
0,18%	0,0%	0,09%	0,07%	0,35%	0,08%	0,17%	0,29%	D_0__Bacteria;D_1__Acidobacteria;D_2__Acidobacteriia;D_3__Acidobacteriales;D_4__uncultured;	uncultured Acidobacteria bacterium
0,06%	0,2%	0,14%	0,10%	0,10%	0,14%	0,18%	0,16%	D_0__Bacteria;D_1__Proteobacteria;D_2__Gammaproteobacteria;D_3__Diplorickettsiales;D_4__Diplorickettsiaceae;	uncultured
0,17%	0,0%	0,29%	0,03%	0,13%	0,11%	0,42%	0,04%	D_0__Bacteria;D_1__Latescibacteria;D_2__uncultured bacterium;D_3__;D_4__;	
0,13%	0,1%	0,09%	0,07%	0,16%	0,35%	0,20%	0,11%	D_0__Bacteria;D_1__Proteobacteria;D_2__Deltaproteobacteria;D_3__Myxococcales;D_4__Phaselicystidaceae;	Phaselicystis
0,08%	0,2%	0,21%	0,08%	0,12%	0,14%	0,22%	0,06%	D_0__Bacteria;D_1__Proteobacteria;D_2__Alphaproteobacteria;D_3__Rhizobiales;D_4__Rhizobiales Incertae Sedis;	uncultured
0,09%	0,1%	0,18%	0,04%	0,15%	0,14%	0,27%	0,08%	D_0__Bacteria;D_1__Acidobacteria;D_2__Thermoanaerobaculia;D_3__Thermoanaerobaculales;D_4__Thermoanaerobaculaceae;	Subgroup 10
0,04%	0,2%	0,07%	0,21%	0,15%	0,12%	0,07%	0,18%	D_0__Bacteria;D_1__Proteobacteria;D_2__Alphaproteobacteria;D_3__Rhodospirillales;D_4__uncultured;	uncultured bacterium
0,10%	0,1%	0,17%	0,04%	0,13%	0,21%	0,21%	0,07%	D_0__Bacteria;D_1__Actinobacteria;D_2__Acidimicrobiia;D_3__uncultured;Other;Other	
0,10%	0,0%	0,34%	0,03%	0,10%	0,07%	0,41%	0,03%	D_0__Bacteria;D_1__Acidobacteria;D_2__Subgroup 22;D_3__uncultured bacterium;D_4__;	
0,06%	0,2%	0,05%	0,01%	0,18%	0,23%	0,14%	0,12%	D_0__Bacteria;D_1__Acidobacteria;D_2__Blastocatellia (Subgroup 4);D_3__Blastocatellales;D_4__Blastocatellaceae;	JGI 0001001-H03
0,05%	0,3%	0,02%	0,02%	0,18%	0,04%	0,02%	0,13%	D_0__Bacteria;D_1__Verrucomicrobia;D_2__Verrucomicrobiae;D_3__Methylacidiphilales;D_4__Methylacidiphilaceae;	uncultured
0,07%	0,1%	0,13%	0,20%	0,12%	0,13%	0,18%	0,13%	D_0__Bacteria;D_1__Proteobacteria;D_2__Alphaproteobacteria;D_3__Caulobacterales;D_4__Hyphomonadaceae;	SWB02
0,16%	0,1%	0,17%	0,13%	0,15%	0,11%	0,11%	0,09%	D_0__Bacteria;D_1__Proteobacteria;D_2__Alphaproteobacteria;D_3__Rhizobiales;D_4__Rhizobiales Incertae Sedis;	Bauldia
0,09%	0,0%	0,19%	0,09%	0,11%	0,20%	0,27%	0,07%	D_0__Bacteria;D_1__Planctomycetes;D_2__OM190;D_3__uncultured bacterium;D_4__;	
0,08%	0,1%	0,21%	0,08%	0,13%	0,08%	0,17%	0,10%	D_0__Bacteria;D_1__Proteobacteria;D_2__Deltaproteobacteria;D_3__Bdellovibrionales;D_4__Bdellovibrionaceae;	Bdellovibrio
0,24%	0,0%	0,20%	0,08%	0,11%	0,08%	0,08%	0,20%	D_0__Bacteria;D_1__Proteobacteria;D_2__Gammaproteobacteria;D_3__Betaproteobacteriales;D_4__A21b;	uncultured beta proteobacterium
0,08%	0,1%	0,03%	0,13%	0,14%	0,08%	0,05%	0,30%	D_0__Bacteria;D_1__Acidobacteria;D_2__Acidobacteriia;D_3__Acidobacteriales;D_4__Acidobacteriaceae (Subgroup 1);	Granulicella
0,06%	0,1%	0,14%	0,11%	0,13%	0,14%	0,22%	0,08%	D_0__Bacteria;D_1__Proteobacteria;D_2__Gammaproteobacteria;D_3__Betaproteobacteriales;D_4__TRA3-20;Other	
0,07%	0,1%	0,05%	0,13%	0,15%	0,18%	0,05%	0,12%	D_0__Bacteria;D_1__Actinobacteria;D_2__Actinobacteria;D_3__Micromonosporales;D_4__Micromonosporaceae;	Actinoplanes
0,07%	0,1%	0,11%	0,13%	0,06%	0,24%	0,09%	0,07%	D_0__Bacteria;D_1__Actinobacteria;D_2__Acidimicrobiia;D_3__Microtrichales;D_4__Ilumatobacteraceae;	Ilumatobacter
0,03%	0,2%	0,08%	0,07%	0,12%	0,10%	0,08%	0,07%	D_0__Bacteria;D_1__Proteobacteria;D_2__Alphaproteobacteria;D_3__Caulobacterales;D_4__Caulobacteraceae;	Caulobacter
0,06%	0,1%	0,12%	0,05%	0,16%	0,15%	0,18%	0,10%	D_0__Bacteria;D_1__Proteobacteria;D_2__Gammaproteobacteria;D_3__Betaproteobacteriales;D_4__TRA3-20;	metagenome
0,05%	0,2%	0,08%	0,14%	0,10%	0,08%	0,10%	0,12%	D_0__Bacteria;D_1__Proteobacteria;D_2__Alphaproteobacteria;D_3__Sphingomonadales;D_4__Sphingomonadaceae;	Sphingobium
0,04%	0,2%	0,09%	0,12%	0,09%	0,13%	0,11%	0,11%	D_0__Bacteria;D_1__Proteobacteria;D_2__Gammaproteobacteria;D_3__Diplorickettsiales;D_4__Diplorickettsiaceae;	Aquicella
0,02%	0,0%	0,02%	0,27%	0,05%	0,03%	0,00%	0,54%	D_0__Bacteria;D_1__Acidobacteria;D_2__Acidobacteriia;D_3__Acidobacteriales;D_4__Acidobacteriaceae (Subgroup 1);	Occallatibacter
0,07%	0,1%	0,16%	0,11%	0,11%	0,12%	0,18%	0,07%	D_0__Bacteria;D_1__Proteobacteria;D_2__Alphaproteobacteria;D_3__Rhizobiales;D_4__Hyphomicrobiaceae;	Hyphomicrobium
0,01%	0,2%	0,10%	0,13%	0,04%	0,12%	0,04%	0,11%	D_0__Bacteria;D_1__Proteobacteria;D_2__Gammaproteobacteria;D_3__Xanthomonadales;D_4__Rhodanobacteraceae;	Luteibacter
0,04%	0,0%	0,09%	0,10%	0,18%	0,17%	0,19%	0,12%	D_0__Bacteria;D_1__Verrucomicrobia;D_2__Verrucomicrobiae;D_3__Pedosphaerales;D_4__Pedosphaeraceae;Other	
0,13%	0,1%	0,31%	0,06%	0,08%	0,06%	0,11%	0,03%	D_0__Bacteria;D_1__Acidobacteria;D_2__Subgroup 17;D_3__uncultured Acidobacteria bacterium;D_4__;	
0,12%	0,3%	0,05%	0,01%	0,05%	0,08%	0,04%	0,01%	D_0__Bacteria;D_1__Actinobacteria;D_2__Actinobacteria;D_3__Frankiales;D_4__Geodermatophilaceae;	Blastococcus
0,04%	0,1%	0,05%	0,06%	0,11%	0,33%	0,17%	0,07%	D_0__Bacteria;D_1__Proteobacteria;D_2__Deltaproteobacteria;D_3__Myxococcales;D_4__BIrii41;	uncultured bacterium
0,04%	0,1%	0,17%	0,06%	0,11%	0,12%	0,21%	0,07%	D_0__Bacteria;D_1__Proteobacteria;D_2__Alphaproteobacteria;D_3__Rhizobiales;D_4__A0839;	uncultured bacterium
0,07%	0,2%	0,14%	0,13%	0,03%	0,13%	0,05%	0,06%	D_0__Bacteria;D_1__Actinobacteria;D_2__Actinobacteria;D_3__Pseudonocardiales;D_4__Pseudonocardiaceae;	Actinophytocola
0,08%	0,3%	0,07%	0,04%	0,04%	0,09%	0,03%	0,02%	D_0__Bacteria;D_1__Proteobacteria;D_2__Alphaproteobacteria;D_3__Rhizobiales;D_4__Beijerinckiaceae;	Microvirga
0,18%	0,1%	0,13%	0,04%	0,09%	0,09%	0,12%	0,08%	D_0__Bacteria;D_1__Acidobacteria;D_2__Blastocatellia (Subgroup 4);D_3__Elev-16S-573;D_4__uncultured bacterium;	
0,05%	0,1%	0,13%	0,28%	0,04%	0,07%	0,07%	0,07%	D_0__Bacteria;D_1__Proteobacteria;D_2__Gammaproteobacteria;D_3__PLTA13;D_4__uncultured bacterium;	
0,15%	0,0%	0,17%	0,10%	0,07%	0,08%	0,16%	0,05%	D_0__Bacteria;D_1__Planctomycetes;D_2__Planctomycetacia;D_3__Gemmatales;D_4__Gemmataceae;	uncultured
0,03%	0,1%	0,08%	0,08%	0,11%	0,13%	0,05%	0,11%	D_0__Bacteria;D_1__Actinobacteria;D_2__Actinobacteria;D_3__Micrococcales;D_4__Microbacteriaceae;	Lysinimonas
0,06%	0,0%	0,14%	0,05%	0,04%	0,06%	0,42%	0,07%	D_0__Bacteria;D_1__Proteobacteria;D_2__Gammaproteobacteria;D_3__Betaproteobacteriales;D_4__Nitrosomonadaceae;	GOUTA6
0,04%	0,1%	0,05%	0,15%	0,12%	0,14%	0,10%	0,12%	D_0__Bacteria;D_1__Proteobacteria;D_2__Alphaproteobacteria;D_3__Rhizobiales;D_4__KF-JG30-B3;	metagenome
0,28%	0,1%	0,08%	0,08%	0,09%	0,08%	0,04%	0,03%	D_0__Bacteria;D_1__Actinobacteria;D_2__Thermoleophilia;D_3__Solirubrobacterales;D_4__67-14;Other	
0,10%	0,1%	0,14%	0,06%	0,09%	0,12%	0,10%	0,03%	D_0__Bacteria;D_1__Acidobacteria;D_2__Subgroup 17;Other;Other;Other	
0,01%	0,1%	0,20%	0,03%	0,04%	0,13%	0,12%	0,10%	D_0__Bacteria;D_1__Bacteroidetes;D_2__Bacteroidia;D_3__Chitinophagales;D_4__Saprospiraceae;	uncultured
0,14%	0,1%	0,08%	0,03%	0,12%	0,08%	0,12%	0,04%	D_0__Bacteria;D_1__Proteobacteria;D_2__Alphaproteobacteria;D_3__Rhizobiales;D_4__Rhizobiales Incertae Sedis;	Nordella
0,10%	0,1%	0,09%	0,07%	0,08%	0,18%	0,08%	0,06%	D_0__Bacteria;D_1__Actinobacteria;D_2__Acidimicrobiia;D_3__Microtrichales;D_4__uncultured;Other	
0,15%	0,1%	0,18%	0,13%	0,05%	0,05%	0,02%	0,04%	D_0__Bacteria;D_1__Actinobacteria;D_2__Actinobacteria;D_3__Frankiales;D_4__Sporichthyaceae;	uncultured
0,02%	0,0%	0,08%	0,26%	0,02%	0,07%	0,05%	0,20%	D_0__Bacteria;D_1__Proteobacteria;D_2__Alphaproteobacteria;D_3__Rhizobiales;D_4__Rhodomicrobiaceae;	Rhodomicrobium
0,01%	0,0%	0,02%	0,23%	0,12%	0,08%	0,07%	0,19%	D_0__Bacteria;D_1__Verrucomicrobia;D_2__Verrucomicrobiae;D_3__Pedosphaerales;D_4__Pedosphaeraceae;	uncultured Verrucomicrobia bacterium
0,06%	0,1%	0,10%	0,10%	0,08%	0,09%	0,04%	0,11%	D_0__Bacteria;D_1__Proteobacteria;D_2__Alphaproteobacteria;D_3__Sphingomonadales;D_4__Sphingomonadaceae;Other	
0,05%	0,0%	0,14%	0,05%	0,07%	0,10%	0,25%	0,05%	D_0__Bacteria;D_1__Elusimicrobia;D_2__Lineage IIa;D_3__uncultured bacterium;D_4__;	
0,26%	0,0%	0,06%	0,01%	0,21%	0,07%	0,10%	0,02%	D_0__Bacteria;D_1__Proteobacteria;D_2__Deltaproteobacteria;D_3__Myxococcales;D_4__Archangiaceae;	Anaeromyxobacter

0,18%	0,0%	0,05%	0,03%	0,19%	0,08%	0,08%	0,09%	D_0__Bacteria;D_1__Actinobacteria;D_2__Acidimicrobiia;D_3__Acidimicrobiales;D_4__Acidimicrobiaceae;	uncultured
0,03%	0,1%	0,12%	0,09%	0,08%	0,04%	0,08%	0,05%	D_0__Bacteria;D_1__Proteobacteria;D_2__Alphaproteobacteria;D_3__Rickettsiales;D_4__SM2D12;	uncultured bacterium
0,01%	0,0%	0,02%	0,06%	0,21%	0,14%	0,12%	0,11%	D_0__Bacteria;D_1__Verrucomicrobia;D_2__Verrucomicrobiae;D_3__Pedosphaerales;D_4__Pedosphaeraceae;	metagenome
0,14%	0,1%	0,12%	0,08%	0,06%	0,07%	0,04%	0,04%	D_0__Bacteria;D_1__Chloroflexi;D_2__KD4-96;Other;Other;Other	
0,07%	0,0%	0,03%	0,26%	0,07%	0,05%	0,04%	0,12%	D_0__Bacteria;D_1__Actinobacteria;D_2__Actinobacteria;D_3__Micromonosporales;D_4__Micromonosporaceae;Other	
0,01%	0,2%	0,03%	0,05%	0,11%	0,04%	0,05%	0,03%	D_0__Bacteria;D_1__Bacteroidetes;D_2__Bacteroidia;D_3__Sphingobacteriales;D_4__Sphingobacteriaceae;	Pedobacter
0,04%	0,1%	0,06%	0,06%	0,12%	0,09%	0,08%	0,11%	D_0__Bacteria;D_1__Proteobacteria;D_2__Alphaproteobacteria;D_3__uncultured;D_4__metagenome;	
0,03%	0,1%	0,04%	0,08%	0,13%	0,13%	0,11%	0,10%	D_0__Bacteria;D_1__Proteobacteria;D_2__Alphaproteobacteria;D_3__Rhodospirillales;D_4__Rhodospirillaceae;	uncultured
0,03%	0,0%	0,20%	0,10%	0,05%	0,04%	0,21%	0,04%	D_0__Bacteria;D_1__Proteobacteria;D_2__Gammaproteobacteria;D_3__Betaproteobacteriales;D_4__Burkholderiaceae;	Comamonas
0,14%	0,0%	0,02%	0,03%	0,16%	0,05%	0,10%	0,18%	D_0__Bacteria;D_1__Acidobacteria;D_2__Acidobacteriia;D_3__Acidobacteriales;D_4__uncultured;Other	
0,06%	0,1%	0,09%	0,05%	0,14%	0,09%	0,11%	0,05%	D_0__Bacteria;D_1__Proteobacteria;D_2__Deltaproteobacteria;D_3__Myxococcales;D_4__Polyangiaceae;	Pajaroellobacter
0,03%	0,0%	0,12%	0,06%	0,11%	0,07%	0,18%	0,06%	D_0__Bacteria;D_1__Acidobacteria;D_2__Subgroup 5;D_3__uncultured Acidobacteria bacterium;D_4__;	
0,03%	0,1%	0,04%	0,08%	0,06%	0,13%	0,03%	0,07%	D_0__Bacteria;D_1__Bacteroidetes;D_2__Bacteroidia;D_3__Chitinophagales;D_4__Chitinophagaceae;	Parafilimonas
0,51%	0,0%	0,00%	0,00%	0,13%	0,00%	0,04%	0,00%	D_0__Bacteria;D_1__Chloroflexi;D_2__AD3;D_3__uncultured bacterium;D_4__;	
0,04%	0,0%	0,27%	0,03%	0,04%	0,04%	0,22%	0,02%	D_0__Bacteria;D_1__Proteobacteria;D_2__Deltaproteobacteria;D_3__NB1-j;D_4__uncultured bacterium;	
0,13%	0,1%	0,11%	0,05%	0,09%	0,09%	0,06%	0,06%	D_0__Bacteria;D_1__Chloroflexi;D_2__TK10;D_3__uncultured bacterium;D_4__;	
0,06%	0,1%	0,07%	0,10%	0,07%	0,07%	0,05%	0,10%	D_0__Bacteria;D_1__Proteobacteria;D_2__Alphaproteobacteria;D_3__Rhizobiales;Other;Other	
0,03%	0,1%	0,06%	0,21%	0,05%	0,05%	0,04%	0,10%	D_0__Bacteria;D_1__Actinobacteria;D_2__Actinobacteria;D_3__Micromonosporales;D_4__Micromonosporaceae;	Asanoa
0,02%	0,1%	0,07%	0,02%	0,03%	0,26%	0,06%	0,04%	D_0__Bacteria;D_1__Proteobacteria;D_2__Gammaproteobacteria;D_3__Xanthomonadales;D_4__Rhodanobacteraceae;	uncultured
0,01%	0,0%	0,04%	0,03%	0,12%	0,18%	0,13%	0,06%	D_0__Bacteria;D_1__Proteobacteria;D_2__Deltaproteobacteria;D_3__Myxococcales;D_4__BIrii41;	metagenome
0,03%	0,2%	0,06%	0,02%	0,08%	0,06%	0,05%	0,02%	D_0__Bacteria;D_1__Proteobacteria;D_2__Gammaproteobacteria;D_3__Xanthomonadales;D_4__Xanthomonadaceae;	Lysobacter
0,02%	0,1%	0,08%	0,04%	0,07%	0,09%	0,05%	0,03%	D_0__Bacteria;D_1__Proteobacteria;D_2__Gammaproteobacteria;D_3__Betaproteobacteriales;D_4__Burkholderiaceae;	Ramlibacter
0,03%	0,0%	0,07%	0,12%	0,06%	0,10%	0,09%	0,11%	D_0__Bacteria;D_1__Actinobacteria;D_2__Actinobacteria;D_3__Micromonosporales;D_4__Micromonosporaceae;	Luedemannella
0,07%	0,1%	0,16%	0,06%	0,02%	0,04%	0,04%	0,06%	D_0__Bacteria;D_1__Proteobacteria;D_2__Alphaproteobacteria;D_3__Tistrellales;D_4__Geminicoccaceae;	Candidatus Alysiosphaera
0,11%	0,1%	0,15%	0,04%	0,06%	0,07%	0,07%	0,03%	D_0__Bacteria;D_1__Actinobacteria;D_2__Acidimicrobiia;D_3__IMCC26256;D_4__uncultured Acidimicrobidae bacterium;	
0,05%	0,1%	0,14%	0,06%	0,03%	0,05%	0,12%	0,07%	D_0__Bacteria;D_1__Proteobacteria;D_2__Deltaproteobacteria;D_3__Myxococcales;D_4__bacteriap25;Other	
0,01%	0,2%	0,01%	0,07%	0,02%	0,03%	0,01%	0,02%	D_0__Bacteria;D_1__Proteobacteria;D_2__Gammaproteobacteria;D_3__Xanthomonadales;D_4__Xanthomonadaceae;	Luteimonas
0,34%	0,0%	0,06%	0,02%	0,08%	0,02%	0,03%	0,02%	D_0__Bacteria;D_1__Actinobacteria;D_2__Thermoleophilia;D_3__Solirubrobacterales;D_4__Solirubrobacteraceae;	uncultured
0,06%	0,0%	0,12%	0,08%	0,06%	0,09%	0,09%	0,04%	D_0__Bacteria;D_1__Proteobacteria;D_2__Deltaproteobacteria;D_3__Bdellovibrionales;D_4__Bdellovibrionaceae;	OM27 clade
0,17%	0,0%	0,12%	0,05%	0,07%	0,04%	0,04%	0,03%	D_0__Bacteria;D_1__Chloroflexi;D_2__KD4-96;D_3__uncultured Chloroflexi bacterium;D_4__;	
0,03%	0,0%	0,06%	0,11%	0,09%	0,08%	0,08%	0,10%	D_0__Bacteria;D_1__Proteobacteria;D_2__Alphaproteobacteria;D_3__Rhizobiales;D_4__KF-JG30-B3;Other	
0,11%	0,0%	0,09%	0,06%	0,10%	0,04%	0,06%	0,10%	D_0__Bacteria;D_1__Proteobacteria;D_2__Gammaproteobacteria;D_3__Betaproteobacteriales;D_4__A21b;Other	
0,03%	0,1%	0,05%	0,07%	0,06%	0,09%	0,05%	0,04%	D_0__Bacteria;D_1__Proteobacteria;D_2__Gammaproteobacteria;D_3__Betaproteobacteriales;D_4__Burkholderiaceae;	Variovorax
0,09%	0,0%	0,07%	0,16%	0,02%	0,02%	0,04%	0,17%	D_0__Bacteria;D_1__Patescibacteria;D_2__Parcubacteria;D_3__Candidatus Kaiserbacteria;D_4__uncultured bacterium;	
0,02%	0,1%	0,05%	0,07%	0,05%	0,08%	0,03%	0,06%	D_0__Bacteria;D_1__Actinobacteria;D_2__Actinobacteria;D_3__Micrococcales;D_4__Microbacteriaceae;Other	
0,04%	0,0%	0,05%	0,06%	0,10%	0,12%	0,11%	0,08%	D_0__Bacteria;D_1__Planctomycetes;D_2__Phycisphaerae;D_3__Phycisphaerales;D_4__Phycisphaeraceae;	SM1A02
0,01%	0,1%	0,02%	0,00%	0,11%	0,10%	0,05%	0,05%	D_0__Bacteria;D_1__Acidobacteria;D_2__Blastocatellia (Subgroup 4);D_3__Blastocatellales;D_4__Blastocatellaceae;	Stenotrophobacter
0,02%	0,1%	0,05%	0,08%	0,05%	0,04%	0,04%	0,06%	D_0__Bacteria;D_1__Proteobacteria;D_2__Alphaproteobacteria;D_3__Rickettsiales;D_4__SM2D12;	metagenome
0,01%	0,0%	0,02%	0,03%	0,09%	0,19%	0,11%	0,08%	D_0__Bacteria;D_1__Bacteroidetes;D_2__Bacteroidia;D_3__Cytophagales;D_4__Microscillaceae;	Ohtaekwangia
0,04%	0,1%	0,10%	0,03%	0,07%	0,06%	0,05%	0,06%	D_0__Bacteria;D_1__Proteobacteria;D_2__Gammaproteobacteria;D_3__Betaproteobacteriales;D_4__Burkholderiaceae;	Massilia
0,05%	0,1%	0,07%	0,06%	0,08%	0,07%	0,07%	0,07%	D_0__Bacteria;D_1__Proteobacteria;D_2__Deltaproteobacteria;D_3__Oligoflexales;D_4__0319-6G20;	uncultured bacterium

(D)

PI1a	PI2a	PI3a	PI4a	PII1a	PII2a	PII3a	PII4a	#OTU ID	
14,34%	15,92%	14,51%	15,38%	13,19%	14,34%	13,08%	11,82%	Unassigned;Other;Other;Other;Other;Other	
5,54%	3,40%	3,60%	5,32%	5,74%	2,19%	3,75%	3,77%	D_0__Bacteria;D_1__Planctomycetes;D_2__Phycisphaerae;D_3__Tepidisphaerales;D_4__WD2101 soil group	uncultured bacterium
3,85%	4,04%	3,28%	3,31%	6,39%	2,71%	3,34%	2,68%	D_0__Bacteria;D_1__Bacteroidetes;D_2__Bacteroidia;D_3__Chitinophagales;D_4__Chitinophagaceae	uncultured
3,80%	2,83%	3,01%	4,44%	3,71%	3,56%	4,18%	3,51%	D_0__Bacteria;D_1__Proteobacteria;D_2__Alphaproteobacteria;D_3__Sphingomonadales;D_4__Sphingomonadaceae	Sphingomonas
0,28%	0,37%	0,24%	0,27%	3,52%	7,21%	0,23%	3,52%	D_0__Bacteria;D_1__Bacteroidetes;D_2__Bacteroidia;D_3__Sphingobacteriales;D_4__Sphingobacteriaceae	Mucilaginibacter
2,76%	1,63%	1,68%	1,90%	1,04%	0,82%	2,32%	2,03%	D_0__Bacteria;D_1__Bacteroidetes;D_2__Bacteroidia;D_3__Chitinophagales;D_4__Chitinophagaceae	Ferruginibacter
1,84%	2,40%	3,41%	1,99%	0,58%	1,14%	2,05%	0,68%	D_0__Bacteria;D_1__Acidobacteria;D_2__Subgroup 6;D_3__uncultured bacterium;D_4__	
1,74%	2,86%	3,06%	1,72%	0,55%	1,11%	2,00%	0,58%	D_0__Bacteria;D_1__Acidobacteria;D_2__Subgroup 6;D_3__uncultured Acidobacteria bacterium;D_4__	
1,45%	0,89%	1,00%	1,35%	3,52%	1,87%	1,77%	1,41%	D_0__Bacteria;D_1__Patescibacteria;D_2__Saccharimonadia;D_3__Saccharimonadales;D_4__uncultured bacterium	
1,38%	1,18%	1,45%	1,52%	0,99%	2,44%	2,15%	1,87%	D_0__Bacteria;D_1__Proteobacteria;D_2__Alphaproteobacteria;D_3__Sphingomonadales;D_4__Sphingomonadaceae	uncultured
1,26%	1,63%	1,67%	1,79%	1,66%	2,09%	1,48%	0,99%	D_0__Bacteria;D_1__Proteobacteria;D_2__Alphaproteobacteria;D_3__Rhizobiales;D_4__Xanthobacteraceae	uncultured
0,93%	0,88%	0,92%	1,12%	2,12%	2,74%	1,17%	2,51%	D_0__Bacteria;D_1__Proteobacteria;D_2__Alphaproteobacteria;D_3__Rhizobiales;D_4__Xanthobacteraceae	Bradyrhizobium
2,04%	1,30%	1,64%	1,96%	1,69%	0,95%	1,30%	1,23%	D_0__Bacteria;D_1__Verrucomicrobia;D_2__Verrucomicrobiae;D_3__Chthoniobacterales;D_4__Chthoniobacteraceae	Chthoniobacter
0,72%	0,75%	0,55%	0,77%	4,15%	1,54%	0,92%	1,58%	D_0__Bacteria;D_1__Proteobacteria;D_2__Alphaproteobacteria;D_3__Caulobacterales;D_4__Caulobacteraceae	Phenylobacterium
0,96%	1,65%	1,79%	0,69%	1,42%	1,16%	1,53%	1,52%	D_0__Bacteria;D_1__Bacteroidetes;D_2__Bacteroidia;D_3__Cytophagales;D_4__Microscillaceae	uncultured
1,52%	1,61%	1,66%	1,19%	0,83%	0,73%	1,64%	1,06%	D_0__Bacteria;D_1__Bacteroidetes;D_2__Bacteroidia;D_3__Chitinophagales;D_4__Chitinophagaceae	Terrimonas
0,92%	1,02%	0,80%	0,97%	2,20%	2,26%	0,96%	0,92%	D_0__Bacteria;D_1__Proteobacteria;D_2__Alphaproteobacteria;D_3__Micropepsales;D_4__Micropepsaceae	uncultured
1,88%	1,03%	1,08%	2,23%	0,54%	0,70%	1,32%	0,51%	D_0__Bacteria;D_1__Verrucomicrobia;D_2__Verrucomicrobiae;D_3__Chthoniobacterales;D_4__Chthoniobacteraceae	Candidatus Udaeobacter
1,54%	1,24%	1,39%	1,16%	0,68%	0,87%	1,75%	0,43%	D_0__Bacteria;D_1__Proteobacteria;D_2__Gammaproteobacteria;D_3__Betaproteobacteriales;D_4__Nitrosomonadaceae	Ellin6067
1,26%	1,68%	2,11%	1,19%	0,28%	0,70%	1,35%	0,48%	D_0__Bacteria;D_1__Acidobacteria;D_2__Subgroup 6;Other;Other;Other	
1,08%	0,73%	0,97%	1,05%	1,20%	1,28%	0,81%	0,71%	D_0__Bacteria;D_1__Acidobacteria;D_2__Acidobacteriia;D_3__Solibacterales;D_4__Solibacteraceae (Subgroup 3)	Bryobacter
1,18%	0,90%	0,87%	0,88%	0,45%	0,83%	1,20%	0,53%	D_0__Bacteria;D_1__Proteobacteria;D_2__Deltaproteobacteria;D_3__Myxococcales;D_4__Haliangiaceae	Haliangium
0,76%	1,18%	1,05%	0,69%	0,80%	0,85%	0,79%	0,65%	D_0__Bacteria;D_1__Proteobacteria;D_2__Gammaproteobacteria;D_3__Gammaproteobacteria Incertae Sedis;D_4__Unknown Family	Acidibacter
0,99%	0,75%	0,93%	1,23%	0,38%	0,67%	0,94%	0,41%	D_0__Bacteria;D_1__Gemmatimonadetes;D_2__Gemmatimonadetes;D_3__Gemmatimonadales;D_4__Gemmatimonadaceae	uncultured
0,87%	0,57%	1,23%	1,15%	0,37%	0,32%	1,00%	0,10%	D_0__Bacteria;D_1__Acidobacteria;D_2__Blastocatellia (Subgroup 4);D_3__Pyrinomonadales;D_4__Pyrinomonadaceae	RB41
0,44%	0,87%	0,27%	0,37%	0,43%	0,41%	0,32%	2,42%	D_0__Bacteria;D_1__Bacteroidetes;D_2__Bacteroidia;D_3__Flavobacteriales;D_4__Flavobacteriaceae	Flavobacterium
0,82%	0,63%	0,52%	0,79%	0,53%	0,64%	1,19%	0,40%	D_0__Bacteria;D_1__Gemmatimonadetes;D_2__Gemmatimonadetes;D_3__Gemmatimonadales;D_4__Gemmatimonadaceae	Gemmatimonas
0,80%	0,85%	0,60%	0,99%	0,61%	0,43%	0,69%	0,32%	D_0__Bacteria;D_1__Proteobacteria;D_2__Gammaproteobacteria;D_3__Betaproteobacteriales;D_4__SC-I-84	uncultured bacterium
0,64%	0,66%	0,45%	0,56%	0,97%	0,54%	0,81%	0,57%	D_0__Bacteria;D_1__Proteobacteria;D_2__Gammaproteobacteria;D_3__Betaproteobacteriales;D_4__Burkholderiaceae	Rhizobacter
0,12%	0,19%	0,13%	0,10%	0,27%	0,45%	0,08%	3,65%	D_0__Bacteria;D_1__Bacteroidetes;D_2__Bacteroidia;D_3__Sphingobacteriales;D_4__Sphingobacteriaceae	Pedobacter
0,60%	0,77%	1,04%	0,57%	0,35%	0,58%	0,72%	0,28%	D_0__Bacteria;D_1__Proteobacteria;D_2__Gammaproteobacteria;D_3__Betaproteobacteriales;D_4__TRA3-20	uncultured bacterium
0,39%	0,49%	0,38%	0,43%	0,65%	1,24%	0,42%	0,76%	D_0__Bacteria;D_1__Proteobacteria;D_2__Alphaproteobacteria;D_3__Caulobacterales;D_4__Caulobacteraceae	uncultured
0,66%	0,94%	1,14%	0,57%	0,15%	0,39%	0,48%	0,19%	D_0__Bacteria;D_1__Proteobacteria;D_2__Gammaproteobacteria;D_3__Betaproteobacteriales;D_4__Nitrosomonadaceae	MND1
0,36%	0,52%	0,53%	0,55%	0,77%	0,78%	0,47%	0,55%	D_0__Bacteria;D_1__Proteobacteria;D_2__Alphaproteobacteria;D_3__Reyranellales;D_4__Reyranellaceae	Reyranella
0,56%	0,69%	0,69%	0,54%	0,40%	0,59%	0,52%	0,36%	D_0__Bacteria;D_1__Proteobacteria;D_2__Gammaproteobacteria;D_3__Betaproteobacteriales;D_4__Nitrosomonadaceae	IS-44
0,55%	0,74%	0,80%	0,85%	0,24%	0,26%	0,55%	0,23%	D_0__Bacteria;D_1__Actinobacteria;D_2__Thermoleophilia;D_3__Solirubrobacterales;D_4__67-14	uncultured

									bacterium
0,44%	0,79%	0,68%	0,75%	0,24%	0,44%	0,52%	0,33%	D_0__Bacteria;D_1__Actinobacteria;D_2__Acidimicrobiia;D_3__IMCC26256;D_4__uncultured bacterium	
0,33%	0,43%	0,41%	0,29%	1,21%	0,84%	0,30%	0,36%	D_0__Bacteria;D_1__Proteobacteria;D_2__Alphaproteobacteria;D_3__Dongiales;D_4__Dongiaceae	Dongia
0,67%	0,31%	0,49%	0,66%	0,69%	0,59%	0,45%	0,29%	D_0__Bacteria;D_1__Acidobacteria;D_2__Acidobacteriia;D_3__Solibacterales;D_4__Solibacteraceae (Subgroup 3)	Candidatus Solibacter
0,29%	0,43%	0,35%	0,36%	0,46%	0,81%	0,43%	0,93%	D_0__Bacteria;D_1__Proteobacteria;D_2__Alphaproteobacteria;D_3__Rhizobiales;D_4__Devosiaceae	Devosia
0,26%	0,30%	0,73%	0,36%	0,36%	0,84%	0,51%	0,64%	D_0__Bacteria;D_1__Proteobacteria;D_2__Gammaproteobacteria;D_3__Betaproteobacteriales;D_4__Burkholderiaceae	uncultured
0,57%	0,63%	0,45%	0,78%	0,18%	0,36%	0,51%	0,29%	D_0__Bacteria;D_1__Actinobacteria;D_2__Acidimicrobiia;D_3__Microtrichales;D_4__Ilumatobacteraceae	CL500-29 marine group
0,39%	0,51%	0,60%	0,54%	0,38%	0,49%	0,63%	0,21%	D_0__Bacteria;D_1__Proteobacteria;D_2__Alphaproteobacteria;D_3__Rhizobiales;D_4__Xanthobacteraceae	Rhodoplanes
0,48%	0,30%	0,28%	0,35%	0,29%	0,40%	0,62%	0,95%	D_0__Bacteria;D_1__Proteobacteria;D_2__Deltaproteobacteria;D_3__Myxococcales;D_4__Sandaracinaceae	uncultured
0,39%	0,37%	0,31%	0,37%	0,61%	0,56%	0,39%	0,53%	D_0__Bacteria;D_1__Proteobacteria;D_2__Gammaproteobacteria;D_3__Betaproteobacteriales;D_4__Burkholderiaceae;Other	
0,16%	0,18%	0,14%	0,10%	0,44%	0,39%	0,11%	1,78%	D_0__Bacteria;D_1__Actinobacteria;D_2__Actinobacteria;D_3__Streptomycetales;D_4__Streptomycetaceae	Streptomyces
0,32%	0,42%	0,35%	0,31%	0,45%	0,53%	0,34%	0,57%	D_0__Bacteria;D_1__Proteobacteria;D_2__Alphaproteobacteria;D_3__Caulobacterales;D_4__Hyphomonadaceae	Hirschia
0,20%	0,19%	0,42%	0,57%	0,13%	0,17%	0,37%	1,21%	D_0__Bacteria;D_1__Firmicutes;D_2__Bacilli;D_3__Bacillales;D_4__Bacillaceae	Bacillus
0,12%	0,13%	0,11%	0,12%	0,53%	0,48%	0,10%	1,63%	D_0__Bacteria;D_1__Proteobacteria;D_2__Gammaproteobacteria;D_3__Betaproteobacteriales;D_4__Burkholderiaceae	Burkholderia-Caballeronia-Paraburkholderia
0,31%	0,25%	0,19%	0,37%	0,18%	1,06%	0,33%	0,44%	D_0__Bacteria;D_1__Proteobacteria;D_2__Alphaproteobacteria;D_3__Acetobacterales;D_4__Acetobacteraceae	uncultured
0,43%	0,30%	0,41%	0,39%	0,23%	0,34%	0,52%	0,31%	D_0__Bacteria;D_1__Proteobacteria;D_2__Alphaproteobacteria;D_3__Rhizobiales;D_4__Rhizobiales Incertae Sedis	uncultured
0,17%	0,21%	0,14%	0,15%	0,71%	0,57%	0,19%	0,75%	D_0__Bacteria;D_1__Proteobacteria;D_2__Alphaproteobacteria;D_3__Caulobacterales;D_4__Caulobacteraceae	Caulobacter
0,38%	0,38%	0,23%	0,38%	0,16%	0,30%	0,72%	0,33%	D_0__Bacteria;D_1__Proteobacteria;D_2__Gammaproteobacteria;D_3__Xanthomonadales;D_4__Xanthomonadaceae	Arenimonas
0,86%	0,21%	0,50%	0,43%	0,06%	0,08%	0,59%	0,12%	D_0__Bacteria;D_1__Bacteroidetes;D_2__Bacteroidia;D_3__Chitinophagales;D_4__Chitinophagaceae	Flavisolibacter
0,29%	0,57%	0,51%	0,45%	0,09%	0,16%	0,48%	0,11%	D_0__Bacteria;D_1__Actinobacteria;D_2__Thermoleophilia;D_3__Gaiellales;D_4__uncultured	uncultured bacterium
0,32%	0,35%	0,12%	0,22%	0,28%	0,38%	0,26%	0,58%	D_0__Bacteria;D_1__Proteobacteria;D_2__Gammaproteobacteria;D_3__Betaproteobacteriales;D_4__Burkholderiaceae	Massilia
0,39%	0,47%	0,42%	0,50%	0,13%	0,11%	0,36%	0,09%	D_0__Bacteria;D_1__Actinobacteria;D_2__Thermoleophilia;D_3__Solirubrobacterales;D_4__Solirubrobacteraceae	Solirubrobacter
0,36%	0,49%	0,27%	0,33%	0,18%	0,23%	0,34%	0,24%	D_0__Bacteria;D_1__Actinobacteria;D_2__Actinobacteria;D_3__Propionibacteriales;D_4__Nocardioidaceae	Nocardioides
0,13%	0,11%	0,12%	0,13%	0,71%	0,30%	0,22%	0,71%	D_0__Bacteria;D_1__Proteobacteria;D_2__Alphaproteobacteria;D_3__Rhizobiales;D_4__Rhizobiaceae	Allorhizobium-Neorhizobium-Pararhizobium-Rhizobium
0,57%	0,40%	0,32%	0,33%	0,09%	0,21%	0,32%	0,15%	D_0__Bacteria;D_1__Bacteroidetes;D_2__Bacteroidia;D_3__Sphingobacteriales;D_4__env,OPS 17	uncultured bacterium
0,38%	0,27%	0,48%	0,35%	0,07%	0,18%	0,46%	0,18%	D_0__Bacteria;D_1__Acidobacteria;D_2__Blastocatellia (Subgroup 4);D_3__Blastocatellales;D_4__Blastocatellaceae	JGI 0001001-H03
0,45%	0,32%	0,26%	0,37%	0,16%	0,16%	0,34%	0,18%	D_0__Bacteria;D_1__Proteobacteria;D_2__Gammaproteobacteria;D_3__Betaproteobacteriales;D_4__Burkholderiaceae	Ramlibacter
0,33%	0,29%	0,32%	0,34%	0,15%	0,20%	0,52%	0,09%	D_0__Bacteria;D_1__Acidobacteria;D_2__Holophagae;D_3__Subgroup 7;D_4__uncultured bacterium	
0,14%	0,11%	0,20%	0,15%	0,80%	0,32%	0,24%	0,27%	D_0__Bacteria;D_1__Proteobacteria;D_2__Alphaproteobacteria;D_3__Rhizobiales;D_4__Rhizobiaceae	Mesorhizobium
0,34%	0,54%	0,34%	0,36%	0,12%	0,11%	0,32%	0,07%	D_0__Bacteria;D_1__Chloroflexi;D_2__KD4-96;D_3__uncultured bacterium;D_4__	
0,39%	0,37%	0,34%	0,22%	0,08%	0,18%	0,35%	0,27%	D_0__Bacteria;D_1__Bacteroidetes;D_2__Bacteroidia;D_3__Chitinophagales;D_4__Saprospiraceae	uncultured
0,33%	0,44%	0,38%	0,30%	0,07%	0,24%	0,26%	0,18%	D_0__Bacteria;D_1__Verrucomicrobia;D_2__Verrucomicrobiae;D_3__Pedosphaerales;D_4__Pedosphaeraceae	uncultured bacterium
0,21%	0,20%	0,21%	0,28%	0,29%	0,21%	0,26%	0,50%	D_0__Bacteria;D_1__Actinobacteria;D_2__Actinobacteria;D_3__Corynebacteriales;D_4__Mycobacteriaceae	Mycobacterium
0,29%	0,40%	0,25%	0,30%	0,12%	0,19%	0,23%	0,34%	D_0__Bacteria;D_1__Bacteroidetes;D_2__Bacteroidia;D_3__Chitinophagales;D_4__Chitinophagaceae	Flavitalea
0,11%	0,17%	0,10%	0,18%	0,33%	0,20%	0,20%	0,73%	D_0__Bacteria;D_1__Proteobacteria;D_2__Gammaproteobacteria;D_3__Pseudomonadales;D_4__Pseudomonadaceae	Pseudomonas
0,24%	0,12%	0,08%	0,16%	0,67%	0,09%	0,20%	0,45%	D_0__Bacteria;D_1__Planctomycetes;D_2__Phycisphaerae;D_3__Tepidisphaerales;D_4__Tepidisphaeraceae	uncultured bacterium
0,33%	0,32%	0,47%	0,25%	0,03%	0,16%	0,35%	0,10%	D_0__Bacteria;D_1__Acidobacteria;D_2__Blastocatellia (Subgroup 4);D_3__Blastocatellales;D_4__Blastocatellaceae	uncultured
0,27%	0,16%	0,49%	0,22%	0,03%	0,12%	0,53%	0,08%	D_0__Bacteria;D_1__Acidobacteria;D_2__Blastocatellia (Subgroup 4);D_3__Blastocatellales;D_4__Blastocatellaceae	Stenotrophobacter
0,24%	0,28%	0,35%	0,23%	0,29%	0,20%	0,17%	0,14%	D_0__Bacteria;D_1__Proteobacteria;D_2__Gammaproteobacteria;D_3__Steroidobacterales;D_4__Steroidobacteraceae	uncultured
0,31%	0,41%	0,25%	0,27%	0,08%	0,17%	0,23%	0,16%	D_0__Bacteria;D_1__Actinobacteria;D_2__Acidimicrobiia;D_3__Microtrichales;D_4__Ilumatobacteraceae	uncultured
0,26%	0,16%	0,19%	0,30%	0,12%	0,12%	0,28%	0,42%	D_0__Bacteria;D_1__Proteobacteria;D_2__Alphaproteobacteria;D_3__Sphingomonadales;D_4__Sphingomonadaceae	Novosphingobium
0,13%	0,32%	0,22%	0,20%	0,19%	0,28%	0,27%	0,23%	D_0__Bacteria;D_1__Proteobacteria;D_2__Alphaproteobacteria;D_3__Rhizobiales;D_4__Hyphomicrobiaceae	Hyphomicrobium
0,29%	0,25%	0,28%	0,18%	0,19%	0,16%	0,22%	0,21%	D_0__Bacteria;D_1__Proteobacteria;D_2__Deltaproteobacteria;D_3__Bdellovibrionales;D_4__Bdellovibrionaceae	Bdellovibrio
0,29%	0,21%	0,24%	0,19%	0,16%	0,15%	0,31%	0,21%	D_0__Bacteria;D_1__Proteobacteria;D_2__Deltaproteobacteria;D_3__Myxococcales;D_4__BIrii41	uncultured bacterium
0,15%	0,29%	0,24%	0,15%	0,16%	0,35%	0,20%	0,19%	D_0__Bacteria;D_1__Proteobacteria;D_2__Alphaproteobacteria;D_3__Caulobacterales;D_4__Hyphomonadaceae	SWB02
0,24%	0,33%	0,30%	0,25%	0,07%	0,16%	0,25%	0,12%	D_0__Bacteria;D_1__Actinobacteria;D_2__Acidimicrobiia;D_3__Microtrichales;D_4__uncultured	uncultured bacterium
0,09%	0,19%	0,18%	0,11%	0,62%	0,21%	0,12%	0,16%	D_0__Bacteria;D_1__Proteobacteria;D_2__Gammaproteobacteria;D_3__Steroidobacterales;D_4__Steroidobacteraceae	Steroidobacter
0,20%	0,09%	0,10%	0,13%	0,50%	0,33%	0,14%	0,16%	D_0__Bacteria;D_1__Patescibacteria;D_2__Saccharimonadia;D_3__Saccharimonadales;Other;Other	
0,19%	0,14%	0,13%	0,20%	0,12%	0,30%	0,27%	0,27%	D_0__Bacteria;D_1__Actinobacteria;D_2__Actinobacteria;D_3__Pseudonocardiales;D_4__Pseudonocardiaceae	Pseudonocardia
0,20%	0,50%	0,26%	0,28%	0,05%	0,10%	0,17%	0,06%	D_0__Bacteria;D_1__Actinobacteria;D_2__Thermoleophilia;D_3__Gaiellales;D_4__Gaiellaceae	Gaiella
0,13%	0,10%	0,12%	0,15%	0,53%	0,14%	0,20%	0,24%	D_0__Bacteria;D_1__Proteobacteria;D_2__Gammaproteobacteria;D_3__Betaproteobacteriales;D_4__Burkholderiaceae	Variovorax
0,08%	0,23%	0,17%	0,14%	0,24%	0,15%	0,17%	0,34%	D_0__Bacteria;D_1__Actinobacteria;D_2__Actinobacteria;D_3__Propionibacteriales;D_4__Nocardioidaceae	Kribbella
0,14%	0,15%	0,13%	0,12%	0,27%	0,08%	0,24%	0,39%	D_0__Bacteria;D_1__Actinobacteria;D_2__Actinobacteria;D_3__Micromonosporales;D_4__Micromonosporaceae	Actinoplanes
0,02%	0,06%	0,04%	0,01%	0,09%	0,47%	0,02%	0,79%	D_0__Bacteria;D_1__Proteobacteria;D_2__Gammaproteobacteria;D_3__Xanthomonadales;D_4__Rhodanobacteraceae	Luteibacter
0,03%	0,04%	0,05%	0,04%	0,29%	0,59%	0,03%	0,42%	D_0__Bacteria;D_1__Acidobacteria;D_2__Acidobacteriia;D_3__Acidobacteriales;D_4__Acidobacteriaceae (Subgroup 1)	Granulicella
0,20%	0,20%	0,25%	0,17%	0,14%	0,20%	0,24%	0,09%	D_0__Bacteria;D_1__Proteobacteria;D_2__Gammaproteobacteria;D_3__Betaproteobacteriales;D_4__TRA3-20	metagenome
0,12%	0,13%	0,11%	0,12%	0,54%	0,09%	0,21%	0,13%	D_0__Bacteria;D_1__Proteobacteria;D_2__Deltaproteobacteria;D_3__Myxococcales;D_4__BIrii41	metagenome
0,08%	0,13%	0,09%	0,10%	0,03%	0,94%	0,03%	0,03%	D_0__Bacteria;D_1__Proteobacteria;D_2__Gammaproteobacteria;D_3__Xanthomonadales;D_4__Rhodanobacteraceae	Rhodanobacter
0,18%	0,11%	0,15%	0,13%	0,15%	0,16%	0,14%	0,39%	D_0__Bacteria;D_1__Bacteroidetes;D_2__Bacteroidia;D_3__Cytophagales;D_4__Microscillaceae	Chryseolinea
0,20%	0,27%	0,15%	0,20%	0,04%	0,14%	0,16%	0,24%	D_0__Bacteria;D_1__Actinobacteria;D_2__Acidimicrobiia;D_3__Microtrichales;D_4__Ilumatobacteraceae	Ilumatobacter
0,11%	0,18%	0,22%	0,17%	0,15%	0,28%	0,18%	0,10%	D_0__Bacteria;D_1__Proteobacteria;D_2__Alphaproteobacteria;D_3__Rhizobiales;D_4__Xanthobacteraceae	Pseudolabrys
0,06%	0,06%	0,03%	0,06%	0,97%	0,08%	0,06%	0,05%	D_0__Bacteria;D_1__Patescibacteria;D_2__Saccharimonadia;D_3__Saccharimonadales;D_4__uncultured Candidatus Saccharibacteria bacterium	
0,18%	0,27%	0,18%	0,25%	0,05%	0,15%	0,12%	0,13%	D_0__Bacteria;D_1__Actinobacteria;D_2__Acidimicrobiia;D_3__IMCC26256;Other;Other	
0,38%	0,20%	0,20%	0,17%	0,08%	0,08%	0,13%	0,07%	D_0__Bacteria;D_1__Patescibacteria;D_2__Parcubacteria;D_3__Candidatus Kaiserbacteria;D_4__uncultured bacterium	
0,13%	0,28%	0,21%	0,22%	0,04%	0,12%	0,16%	0,13%	D_0__Bacteria;D_1__Actinobacteria;D_2__Acidimicrobiia;D_3__Microtrichales;D_4__Iamiaceae	Iamia
0,17%	0,12%	0,07%	0,20%	0,09%	0,07%	0,10%	0,46%	D_0__Bacteria;D_1__Verrucomicrobia;D_2__Verrucomicrobiae;D_3__Verrucomicrobiales;D_4__Rubritaleaceae	Luteolibacter
0,18%	0,13%	0,17%	0,15%	0,12%	0,11%	0,29%	0,13%	D_0__Bacteria;D_1__Proteobacteria;D_2__Deltaproteobacteria;D_3__Myxococcales;D_4__Polyangiaceae	Pajaroellobacter
0,12%	0,15%	0,16%	0,19%	0,07%	0,20%	0,25%	0,14%	D_0__Bacteria;D_1__Proteobacteria;D_2__Alphaproteobacteria;D_3__Sphingomonadales;D_4__Sphingomonadaceae	Ellin6055
0,14%	0,17%	0,22%	0,07%	0,16%	0,13%	0,19%	0,16%	D_0__Bacteria;D_1__Bacteroidetes;D_2__Bacteroidia;D_3__Cytophagales;D_4__Microscillaceae	Ohtaekwangia
0,22%	0,07%	0,20%	0,13%	0,10%	0,18%	0,19%	0,18%	D_0__Bacteria;D_1__Acidobacteria;D_2__Acidobacteriia;D_3__Solibacterales;D_4__Solibacteraceae (Subgroup 3)	Paludibaculum
0,11%	0,09%	0,08%	0,16%	0,13%	0,13%	0,16%	0,39%	D_0__Bacteria;D_1__Proteobacteria;D_2__Alphaproteobacteria;D_3__Sphingomonadales;D_4__Sphingomonadaceae	Sphingobium

0,18%	0,10%	0,14%	0,22%	0,25%	0,09%	0,13%	0,12%	D_0__Bacteria;D_1__Planctomycetes;D_2__Phycisphaerae;D_3__Tepidisphaerales;D_4__WD2101 soil group;Other	
0,23%	0,21%	0,24%	0,19%	0,03%	0,10%	0,15%	0,08%	D_0__Bacteria;D_1__Acidobacteria;D_2__Thermoanaerobaculia;D_3__Thermoanaerobaculales;D_4__Thermoanaero baculaceae	Subgroup 10
0,14%	0,12%	0,11%	0,15%	0,16%	0,15%	0,17%	0,17%	D_0__Bacteria;D_1__Proteobacteria;D_2__Alphaproteobacteria;D_3__Sphingomonadales;D_4__Sphingomonadaceae;Other	
0,18%	0,17%	0,12%	0,13%	0,07%	0,15%	0,20%	0,14%	D_0__Bacteria;D_1__Proteobacteria;D_2__Deltaproteobacteria;D_3__Myxococcales;D_4__Phaselicystidaceae	Phaselicystis
0,12%	0,11%	0,12%	0,14%	0,20%	0,26%	0,10%	0,07%	D_0__Bacteria;D_1__Proteobacteria;D_2__Alphaproteobacteria;D_3__Rhodospirillales;D_4__Rhodospirillaceae	uncultured
0,09%	0,12%	0,12%	0,13%	0,18%	0,24%	0,09%	0,16%	D_0__Bacteria;D_1__Proteobacteria;D_2__Alphaproteobacteria;D_3__Rhizobiales;D_4__Xanthobacteraceae;Other	
0,18%	0,13%	0,18%	0,17%	0,03%	0,11%	0,22%	0,08%	D_0__Bacteria;D_1__Bacteroidetes;D_2__Bacteroidia;D_3__Sphingobacteriales;D_4__AKYH767	uncultured bacterium
0,10%	0,18%	0,16%	0,17%	0,11%	0,15%	0,18%	0,05%	D_0__Bacteria;D_1__Proteobacteria;D_2__Alphaproteobacteria;D_3__Rhizobiales;D_4__uncultured	uncultured bacterium
0,36%	0,14%	0,07%	0,03%	0,40%	0,06%	0,02%	0,04%	D_0__Bacteria;D_1__Bacteroidetes;D_2__Bacteroidia;D_3__Chitinophagales;D_4__Chitinophagaceae	Niastella
0,07%	0,52%	0,13%	0,05%	0,03%	0,04%	0,23%	0,03%	D_0__Bacteria;D_1__Acidobacteria;D_2__Blastocatellia (Subgroup 4);D_3__Blastocatellales;D_4__Blastocatellaceae;Other	
0,10%	0,26%	0,23%	0,06%	0,02%	0,11%	0,19%	0,14%	D_0__Bacteria;D_1__Bacteroidetes;D_2__Bacteroidia;D_3__Cytophagales;D_4__Hymenobacteraceae	Adhaeribacter
0,10%	0,27%	0,30%	0,10%	0,03%	0,13%	0,07%	0,08%	D_0__Bacteria;D_1__Proteobacteria;D_2__Gammaproteobacteria;D_3__CCD24;D_4__uncultured bacterium	
0,12%	0,16%	0,25%	0,17%	0,07%	0,07%	0,18%	0,06%	D_0__Bacteria;D_1__Proteobacteria;D_2__Alphaproteobacteria;D_3__Rhizobiales;D_4__Hyphomicrobiaceae	Pedomicrobium
0,14%	0,14%	0,20%	0,15%	0,09%	0,11%	0,20%	0,04%	D_0__Bacteria;D_1__Proteobacteria;D_2__Gammaproteobacteria;D_3__Betaproteobacteriales;D_4__TRA3-20;Other	
0,12%	0,16%	0,26%	0,14%	0,06%	0,10%	0,16%	0,04%	D_0__Bacteria;D_1__Proteobacteria;D_2__Alphaproteobacteria;D_3__Rhizobiales;D_4__KF-JG30-B3	uncultured bacterium
0,11%	0,17%	0,12%	0,15%	0,10%	0,17%	0,13%	0,05%	D_0__Bacteria;D_1__Proteobacteria;D_2__Alphaproteobacteria;D_3__Rhizobiales;D_4__Rhizobiales Incertae Sedis	Bauldia
0,13%	0,11%	0,14%	0,15%	0,07%	0,10%	0,21%	0,09%	D_0__Bacteria;D_1__Proteobacteria;D_2__Deltaproteobacteria;D_3__Oligoflexales;D_4__0319-6G20	uncultured bacterium
0,14%	0,12%	0,18%	0,13%	0,09%	0,13%	0,14%	0,06%	D_0__Bacteria;D_1__Proteobacteria;D_2__Deltaproteobacteria;D_3__Desulfarculales;D_4__Desulfarculaceae	uncultured
0,15%	0,15%	0,11%	0,11%	0,16%	0,10%	0,10%	0,09%	D_0__Bacteria;D_1__Bacteroidetes;D_2__Bacteroidia;D_3__Chitinophagales;D_4__Chitinophagaceae;Other	
0,15%	0,17%	0,15%	0,18%	0,06%	0,09%	0,09%	0,05%	D_0__Bacteria;D_1__Proteobacteria;D_2__Gammaproteobacteria;D_3__Betaproteobacteriales;D_4__SC-I-84;Other	
0,14%	0,13%	0,14%	0,12%	0,06%	0,11%	0,10%	0,10%	D_0__Bacteria;D_1__Verrucomicrobia;D_2__Verrucomicrobiae;D_3__Opitutales;D_4__Opitutaceae	Opitutus
0,16%	0,12%	0,16%	0,14%	0,05%	0,04%	0,12%	0,12%	D_0__Bacteria;D_1__Verrucomicrobia;D_2__Verrucomicrobiae;D_3__Verrucomicrobiales;D_4__Verrucomicrobiaceae	uncultured
0,06%	0,08%	0,03%	0,06%	0,14%	0,16%	0,07%	0,29%	D_0__Bacteria;D_1__Proteobacteria;D_2__Alphaproteobacteria;D_3__Caulobacterales;D_4__Caulobacteraceae	Brevundimonas
0,20%	0,13%	0,15%	0,14%	0,02%	0,05%	0,13%	0,06%	D_0__Bacteria;D_1__Bacteroidetes;D_2__Bacteroidia;D_3__Chitinophagales;D_4__Chitinophagaceae	Parafilimonas
0,05%	0,15%	0,08%	0,10%	0,19%	0,14%	0,08%	0,09%	D_0__Bacteria;D_1__Proteobacteria;D_2__Gammaproteobacteria;D_3__Xanthomonadales;D_4__Rhodanobacterace ae	Dokdonella
0,08%	0,16%	0,10%	0,11%	0,12%	0,09%	0,08%	0,14%	D_0__Bacteria;D_1__Proteobacteria;D_2__Gammaproteobacteria;D_3__R7C24;D_4__metagenome	
0,06%	0,09%	0,07%	0,04%	0,45%	0,06%	0,05%	0,04%	D_0__Bacteria;D_1__Actinobacteria;D_2__Actinobacteria;D_3__Micromonosporales;D_4__Micromonosporaceae	Luedemannella
0,12%	0,07%	0,14%	0,11%	0,11%	0,09%	0,13%	0,08%	D_0__Bacteria;D_1__Proteobacteria;D_2__Gammaproteobacteria;D_3__Diplorickettsiales;D_4__Diplorickettsiaceae	uncultured
0,10%	0,13%	0,14%	0,16%	0,04%	0,10%	0,09%	0,07%	D_0__Bacteria;D_1__Proteobacteria;D_2__Gammaproteobacteria;D_3__Xanthomonadales;D_4__Xanthomonadacea e	Lysobacter
0,12%	0,09%	0,14%	0,09%	0,02%	0,03%	0,24%	0,09%	D_0__Bacteria;D_1__Acidobacteria;D_2__Blastocatellia (Subgroup 4);D_3__Blastocatellales;D_4__Blastocatellaceae	Blastocatella
0,05%	0,05%	0,04%	0,03%	0,50%	0,01%	0,07%	0,07%	D_0__Bacteria;D_1__Actinobacteria;D_2__Actinobacteria;D_3__Micromonosporales;D_4__Micromonosporaceae	Dactylosporangium
0,08%	0,08%	0,04%	0,08%	0,21%	0,11%	0,10%	0,14%	D_0__Bacteria;D_1__Proteobacteria;D_2__Gammaproteobacteria;D_3__Betaproteobacteriales;D_4__Burkholderiace ae	Rhodoferax
0,12%	0,08%	0,10%	0,08%	0,08%	0,06%	0,21%	0,09%	D_0__Bacteria;D_1__Proteobacteria;D_2__Gammaproteobacteria;D_3__Betaproteobacteriales;D_4__Burkholderiace ae	Caenimonas
0,10%	0,11%	0,13%	0,17%	0,04%	0,06%	0,17%	0,04%	D_0__Bacteria;D_1__Actinobacteria;D_2__Acidimicrobiia;D_3__uncultured;Other;Other	
0,10%	0,11%	0,08%	0,07%	0,12%	0,10%	0,08%	0,15%	D_0__Bacteria;D_1__Verrucomicrobia;D_2__Verrucomicrobiae;D_3__Opitutales;D_4__Opitutaceae	Lacunisphaera
0,10%	0,07%	0,05%	0,12%	0,04%	0,07%	0,17%	0,18%	D_0__Bacteria;D_1__Actinobacteria;D_2__Actinobacteria;D_3__Frankiales;D_4__Nakamurellaceae	Nakamurella
0,07%	0,09%	0,05%	0,05%	0,16%	0,11%	0,10%	0,18%	D_0__Bacteria;D_1__Bacteroidetes;D_2__Bacteroidia;D_3__Sphingobacteriales;D_4__Sphingobacteriaceae	uncultured
0,12%	0,12%	0,14%	0,11%	0,04%	0,11%	0,09%	0,09%	D_0__Bacteria;D_1__Verrucomicrobia;D_2__Verrucomicrobiae;D_3__Pedosphaerales;D_4__Pedosphaeraceae;Other	
0,08%	0,05%	0,02%	0,05%	0,32%	0,09%	0,05%	0,15%	D_0__Bacteria;D_1__Planctomycetes;D_2__Phycisphaerae;D_3__Tepidisphaerales;D_4__Tepidisphaeraceae	Tepidisphaera
0,09%	0,12%	0,13%	0,08%	0,03%	0,07%	0,17%	0,10%	D_0__Bacteria;D_1__Proteobacteria;D_2__Gammaproteobacteria;D_3__Xanthomonadales;D_4__Rhodanobacterace ae	uncultured
0,14%	0,10%	0,12%	0,12%	0,10%	0,04%	0,11%	0,05%	D_0__Bacteria;D_1__Proteobacteria;D_2__Deltaproteobacteria;D_3__Myxococcales;D_4__Polyangiaceae	uncultured
0,13%	0,07%	0,07%	0,06%	0,18%	0,04%	0,15%	0,07%	D_0__Bacteria;D_1__Bacteroidetes;D_2__Bacteroidia;D_3__Chitinophagales;D_4__Chitinophagaceae	Sediminibacterium
0,12%	0,08%	0,08%	0,17%	0,06%	0,05%	0,15%	0,08%	D_0__Bacteria;D_1__Actinobacteria;D_2__Actinobacteria;D_3__Frankiales;D_4__Geodermatophilaceae	Blastococcus
0,05%	0,15%	0,14%	0,06%	0,04%	0,17%	0,11%	0,06%	D_0__Bacteria;D_1__Proteobacteria;D_2__Alphaproteobacteria;D_3__Rhizobiales;D_4__A0839	uncultured bacterium
0,22%	0,13%	0,13%	0,12%	0,04%	0,03%	0,06%	0,03%	D_0__Bacteria;D_1__Patescibacteria;D_2__Parcubacteria;D_3__Candidatus Adlerbacteria;D_4__uncultured bacterium	
0,05%	0,05%	0,03%	0,03%	0,24%	0,12%	0,03%	0,20%	D_0__Bacteria;D_1__Acidobacteria;D_2__Acidobacteriia;D_3__Acidobacteriales;D_4__Acidobacteriaceae (Subgroup 1)	Edaphobacter
0,05%	0,11%	0,08%	0,07%	0,13%	0,15%	0,11%	0,05%	D_0__Bacteria;D_1__Proteobacteria;D_2__Alphaproteobacteria;D_3__Rhizobiales;D_4__KF-JG30-B3	metagenome
0,01%	0,01%	0,01%	0,01%	0,68%	0,03%	0,00%	0,02%	D_0__Bacteria;D_1__Patescibacteria;D_2__Saccharimonadia;D_3__Saccharimonadales;D_4__uncultured soil bacterium	
0,10%	0,09%	0,15%	0,07%	0,03%	0,06%	0,17%	0,09%	D_0__Bacteria;D_1__Proteobacteria;D_2__Alphaproteobacteria;D_3__Sphingomonadales;D_4__Sphingomonadaceae	Altererythrobacter
0,13%	0,08%	0,10%	0,14%	0,06%	0,09%	0,08%	0,07%	D_0__Bacteria;D_1__Proteobacteria;D_2__Alphaproteobacteria;D_3__Elsterales;D_4__uncultured	uncultured bacterium
0,08%	0,04%	0,20%	0,14%	0,02%	0,04%	0,22%	0,01%	D_0__Bacteria;D_1__Actinobacteria;D_2__Rubrobacteria;D_3__Rubrobacterales;D_4__Rubrobacteriaceae	Rubrobacter
0,11%	0,13%	0,15%	0,14%	0,05%	0,03%	0,14%	0,01%	D_0__Bacteria;D_1__Acidobacteria;D_2__Subgroup 17;D_3__uncultured bacterium;D_4__	
0,12%	0,13%	0,08%	0,13%	0,07%	0,08%	0,07%	0,06%	D_0__Bacteria;D_1__Actinobacteria;D_2__Thermoleophilia;D_3__Solirubrobacterales;D_4__Solirubrobacteraceae	Conexibacter
0,05%	0,06%	0,05%	0,03%	0,11%	0,04%	0,03%	0,35%	D_0__Bacteria;D_1__Firmicutes;D_2__Bacilli;D_3__Bacillales;D_4__Paenibacillaceae	Paenibacillus
0,10%	0,10%	0,11%	0,11%	0,06%	0,07%	0,08%	0,10%	D_0__Bacteria;D_1__Planctomycetes;D_2__Phycisphaerae;D_3__Phycisphaerales;D_4__Phycisphaeraceae	SM1A02
0,08%	0,08%	0,08%	0,10%	0,02%	0,05%	0,06%	0,25%	D_0__Bacteria;D_1__Proteobacteria;D_2__Gammaproteobacteria;D_3__Cellvibrionales;D_4__Halieaceae	OM60(NOR5) clade
0,11%	0,06%	0,16%	0,15%	0,02%	0,02%	0,16%	0,01%	D_0__Bacteria;D_1__Proteobacteria;D_2__Deltaproteobacteria;D_3__Myxococcales;D_4__bacteriap25;Other	
0,03%	0,04%	0,05%	0,05%	0,36%	0,05%	0,09%	0,01%	D_0__Bacteria;D_1__Acidobacteria;D_2__Holophagae;D_3__Subgroup 7;Other;Other	
0,09%	0,07%	0,07%	0,09%	0,08%	0,11%	0,08%	0,09%	D_0__Bacteria;D_1__Bacteroidetes;D_2__Bacteroidia;D_3__Bacteroidales;D_4__Prevotellaceae	Prevotella 7
0,08%	0,08%	0,10%	0,06%	0,13%	0,09%	0,07%	0,06%	D_0__Bacteria;D_1__Bacteroidetes;D_2__Bacteroidia;D_3__Chitinophagales;D_4__Chitinophagaceae	Chitinophaga
0,13%	0,11%	0,09%	0,09%	0,02%	0,05%	0,10%	0,07%	D_0__Bacteria;D_1__Bacteroidetes;D_2__Bacteroidia;D_3__Sphingobacteriales;D_4__AKYH767;Other	
0,06%	0,05%	0,08%	0,11%	0,03%	0,09%	0,16%	0,08%	D_0__Bacteria;D_1__Proteobacteria;D_2__Alphaproteobacteria;D_3__Rhizobiales;D_4__Beijerinckiaceae	Microvirga
0,10%	0,09%	0,10%	0,08%	0,06%	0,07%	0,07%	0,07%	D_0__Bacteria;D_1__Verrucomicrobia;D_2__Verrucomicrobiae;D_3__Pedosphaerales;D_4__Pedosphaeraceae	metagenome
0,05%	0,08%	0,09%	0,09%	0,10%	0,13%	0,06%	0,05%	D_0__Bacteria;D_1__Proteobacteria;D_2__Alphaproteobacteria;D_3__Rickettsiales;D_4__SM2D12	metagenome
0,06%	0,08%	0,19%	0,06%	0,02%	0,04%	0,18%	0,01%	D_0__Bacteria;D_1__Proteobacteria;D_2__Deltaproteobacteria;D_3__Myxococcales;D_4__bacteriap25	uncultured bacterium
0,13%	0,12%	0,08%	0,09%	0,06%	0,05%	0,09%	0,03%	D_0__Bacteria;D_1__Proteobacteria;D_2__Deltaproteobacteria;D_3__Bdellovibrionales;D_4__Bdellovibrionaceae	OM27 clade
0,12%	0,04%	0,08%	0,11%	0,13%	0,03%	0,08%	0,03%	D_0__Bacteria;D_1__Planctomycetes;D_2__Phycisphaerae;D_3__Tepidisphaerales;D_4__WD2101 soil group	uncultured planctomycete
0,15%	0,11%	0,08%	0,12%	0,01%	0,04%	0,10%	0,04%	D_0__Bacteria;D_1__Bacteroidetes;D_2__Bacteroidia;D_3__Chitinophagales;D_4__uncultured	uncultured bacterium
0,03%	0,07%	0,05%	0,04%	0,18%	0,13%	0,05%	0,07%	D_0__Bacteria;D_1__Proteobacteria;D_2__Alphaproteobacteria;D_3__Rhizobiales;Other;Other	
0,22%	0,07%	0,11%	0,11%	0,01%	0,03%	0,05%	0,01%	D_0__Bacteria;D_1__Patescibacteria;D_2__Parcubacteria;D_3__uncultured bacterium;D_4__	
0,17%	0,07%	0,11%	0,10%	0,01%	0,06%	0,06%	0,03%	D_0__Bacteria;D_1__Elusimicrobia;D_2__Lineage IIa;D_3__uncultured bacterium;D_4__	
0,07%	0,11%	0,14%	0,09%	0,04%	0,05%	0,10%	0,02%	D_0__Bacteria;D_1__Acidobacteria;D_2__Blastocatellia (Subgroup 4);D_3__Elev-16S-573;D_4__uncultured bacterium	
0,13%	0,07%	0,08%	0,09%	0,04%	0,02%	0,13%	0,03%	D_0__Bacteria;D_1__Planctomycetes;D_2__Planctomycetacia;D_3__Gemmatales;D_4__Gemmataceae	uncultured
0,08%	0,18%	0,11%	0,06%	0,02%	0,07%	0,05%	0,03%	D_0__Bacteria;D_1__Acidobacteria;D_2__Subgroup 5;D_3__uncultured Acidobacteria bacterium;D_4__	
0,02%	0,04%	0,11%	0,04%	0,15%	0,04%	0,15%	0,04%	D_0__Bacteria;D_1__Verrucomicrobia;D_2__Verrucomicrobiae;D_3__Chthoniobacterales;D_4__Xiphinematobacterac eae	Candidatus Xiphinematobacter
0,02%	0,06%	0,03%	0,01%	0,17%	0,13%	0,02%	0,14%	D_0__Bacteria;D_1__Proteobacteria;D_2__Alphaproteobacteria;D_3__Azospirillales;D_4__Inquilinaceae	Inquilinus
0,10%	0,08%	0,06%	0,13%	0,02%	0,07%	0,10%	0,03%	D_0__Bacteria;D_1__Actinobacteria;D_2__Acidimicrobiia;D_3__uncultured;D_4__uncultured bacterium	
0,14%	0,07%	0,05%	0,07%	0,03%	0,01%	0,10%	0,10%	D_0__Bacteria;D_1__Bacteroidetes;D_2__Bacteroidia;D_3__Chitinophagales;D_4__Chitinophagaceae	Segetibacter
0,08%	0,07%	0,04%	0,07%	0,08%	0,07%	0,08%	0,10%	D_0__Bacteria;D_1__Proteobacteria;D_2__Gammaproteobacteria;D_3__Betaproteobacteriales;D_4__Burkholderiace	Acidovorax

								ae	
0,05%	0,06%	0,06%	0,08%	0,08%	0,14%	0,06%	0,03%	D_0__Bacteria;D_1__Proteobacteria;D_2__Alphaproteobacteria;D_3__Rhodospirillales;D_4__uncultured	uncultured bacterium
0,04%	0,05%	0,04%	0,05%	0,09%	0,17%	0,05%	0,10%	D_0__Bacteria;D_1__Proteobacteria;D_2__Alphaproteobacteria;D_3__Rhizobiales;D_4__Xanthobacteraceae	Afipia
0,07%	0,06%	0,04%	0,08%	0,04%	0,15%	0,07%	0,06%	D_0__Bacteria;D_1__Actinobacteria;D_2__Actinobacteria;D_3__Frankiales;D_4__Frankiaceae	Jatrophihabitans
0,14%	0,06%	0,09%	0,13%	0,01%	0,01%	0,07%	0,05%	D_0__Bacteria;D_1__Gemmatimonadetes;D_2__Longimicrobia;D_3__Longimicrobiales;D_4__Longimicrobiaceae	uncultured bacterium
0,05%	0,08%	0,08%	0,08%	0,09%	0,08%	0,06%	0,04%	D_0__Bacteria;D_1__Proteobacteria;D_2__Alphaproteobacteria;D_3__uncultured;D_4__uncultured bacterium	
0,02%	0,05%	0,05%	0,05%	0,11%	0,08%	0,05%	0,13%	D_0__Bacteria;D_1__Actinobacteria;D_2__Actinobacteria;D_3__Micrococcales;D_4__Microbacteriaceae	Lysinimonas
0,07%	0,11%	0,13%	0,10%	0,02%	0,04%	0,05%	0,01%	D_0__Bacteria;D_1__Chloroflexi;D_2__Dehalococcoidia;D_3__S085;D_4__uncultured bacterium	
0,08%	0,05%	0,11%	0,04%	0,01%	0,01%	0,19%	0,05%	D_0__Bacteria;D_1__Acidobacteria;D_2__Blastocatellia (Subgroup 4);D_3__Blastocatellales;D_4__Blastocatellaceae	Aridibacter
0,03%	0,03%	0,03%	0,02%	0,07%	0,15%	0,01%	0,20%	D_0__Bacteria;D_1__Bacteroidetes;D_2__Bacteroidia;D_3__Chitinophagales;D_4__Chitinophagaceae	Taibaiella
0,07%	0,11%	0,09%	0,07%	0,02%	0,05%	0,10%	0,03%	D_0__Bacteria;D_1__Acidobacteria;D_2__Subgroup 6;D_3__uncultured Acidobacteriales bacterium;D_4__	
0,07%	0,05%	0,05%	0,09%	0,06%	0,05%	0,08%	0,08%	D_0__Bacteria;D_1__Armatimonadetes;D_2__Fimbriimonadia;D_3__Fimbriimonadales;D_4__Fimbriimonadaceae	uncultured bacterium
0,08%	0,10%	0,06%	0,08%	0,02%	0,08%	0,06%	0,04%	D_0__Bacteria;D_1__Planctomycetes;D_2__OM190;D_3__uncultured bacterium;D_4__	
0,05%	0,10%	0,07%	0,04%	0,06%	0,08%	0,05%	0,06%	D_0__Bacteria;D_1__Proteobacteria;D_2__Alphaproteobacteria;D_3__Reyranellales;D_4__Reyranellaceae	uncultured
0,03%	0,08%	0,09%	0,05%	0,09%	0,07%	0,04%	0,05%	D_0__Bacteria;D_1__Proteobacteria;D_2__Alphaproteobacteria;D_3__Micavibrionales;D_4__uncultured	uncultured bacterium
0,04%	0,04%	0,06%	0,05%	0,12%	0,03%	0,08%	0,07%	D_0__Bacteria;D_1__Actinobacteria;D_2__Actinobacteria;D_3__Micromonosporales;D_4__Micromonosporaceae	Asanoa

(E)

PI1b	PI2b	PI3b	PI4b	PII1b	PII2b	PII3b	PII4b	#OTU ID	
14,05%	14,66%	16,79%	15,40%	16,80%	15,18%	18,09%	17,32%	Unassigned;Other;Other;Other;Other;Other	
4,79%	2,67%	3,31%	4,94%	4,45%	2,42%	3,94%	6,46%	D_0__Bacteria;D_1__Planctomycetes;D_2__Phycisphaerae;D_3__Tepidisphaerales;D_4__WD2101 soil group	uncultured bacterium
3,95%	2,19%	3,00%	4,20%	3,47%	2,30%	3,84%	4,97%	D_0__Bacteria;D_1__Proteobacteria;D_2__Alphaproteobacteria;D_3__Sphingomonadales;D_4__Sphingomonadaceae	Sphingomonas
3,68%	4,40%	2,82%	3,04%	2,96%	4,82%	2,84%	1,58%	D_0__Bacteria;D_1__Bacteroidetes;D_2__Bacteroidia;D_3__Chitinophagales;D_4__Chitinophagaceae	uncultured
3,93%	2,68%	1,71%	3,99%	1,73%	2,45%	1,00%	2,44%	D_0__Bacteria;D_1__Verrucomicrobia;D_2__Verrucomicrobiae;D_3__Chthoniobacterales;D_4__Chthoniobacteraceae	Candidatus Udaeobacter
2,01%	3,86%	2,62%	1,99%	1,22%	2,24%	2,63%	2,92%	D_0__Bacteria;D_1__Acidobacteria;D_2__Subgroup 6;D_3__uncultured Acidobacteria bacterium;D_4__	
2,11%	2,99%	3,08%	2,20%	1,20%	1,88%	2,76%	2,86%	D_0__Bacteria;D_1__Acidobacteria;D_2__Subgroup 6;D_3__uncultured bacterium;D_4__	
1,64%	1,66%	1,67%	2,01%	1,98%	1,97%	2,33%	2,90%	D_0__Bacteria;D_1__Proteobacteria;D_2__Alphaproteobacteria;D_3__Rhizobiales;D_4__Xanthobacteraceae	uncultured
2,53%	1,72%	1,85%	3,29%	0,44%	1,06%	1,68%	0,99%	D_0__Bacteria;D_1__Acidobacteria;D_2__Blastocatellia (Subgroup 4);D_3__Pyrinomonadales;D_4__Pyrinomonadaceae	RB41
1,48%	2,08%	1,89%	1,47%	0,76%	1,45%	1,58%	1,69%	D_0__Bacteria;D_1__Acidobacteria;D_2__Subgroup 6;Other;Other;Other	
1,05%	2,71%	1,86%	0,89%	1,78%	2,63%	0,96%	0,45%	D_0__Bacteria;D_1__Bacteroidetes;D_2__Bacteroidia;D_3__Cytophagales;D_4__Microscillaceae	uncultured
2,22%	0,85%	0,99%	2,03%	0,93%	0,72%	1,36%	2,04%	D_0__Bacteria;D_1__Gemmatimonadetes;D_2__Gemmatimonadetes;D_3__Gemmatimonadales;D_4__Gemmatimonad aceae	uncultured
1,01%	0,88%	1,36%	1,17%	1,74%	1,27%	1,07%	1,84%	D_0__Bacteria;D_1__Proteobacteria;D_2__Alphaproteobacteria;D_3__Rhizobiales;D_4__Xanthobacteraceae	Bradyrhizobium
1,44%	1,47%	1,47%	1,35%	0,83%	1,79%	1,42%	0,54%	D_0__Bacteria;D_1__Bacteroidetes;D_2__Bacteroidia;D_3__Chitinophagales;D_4__Chitinophagaceae	Terrimonas
1,75%	2,14%	1,67%	1,15%	0,41%	1,03%	1,33%	0,80%	D_0__Bacteria;D_1__Proteobacteria;D_2__Gammaproteobacteria;D_3__Betaproteobacteriales;D_4__Nitrosomonadace ae	MND1
1,70%	1,33%	1,13%	1,14%	1,00%	1,37%	1,41%	1,21%	D_0__Bacteria;D_1__Proteobacteria;D_2__Gammaproteobacteria;D_3__Betaproteobacteriales;D_4__Nitrosomonadace ae	Ellin6067
1,51%	1,31%	1,25%	1,33%	1,69%	1,51%	0,51%	0,88%	D_0__Bacteria;D_1__Verrucomicrobia;D_2__Verrucomicrobiae;D_3__Chthoniobacterales;D_4__Chthoniobacteraceae	Chthoniobacter
1,31%	1,00%	0,80%	0,93%	1,03%	1,84%	1,75%	1,05%	D_0__Bacteria;D_1__Proteobacteria;D_2__Deltaproteobacteria;D_3__Myxococcales;D_4__Haliangiaceae	Haliangium
0,94%	0,88%	1,10%	1,16%	1,01%	0,90%	1,93%	1,35%	D_0__Bacteria;D_1__Proteobacteria;D_2__Alphaproteobacteria;D_3__Sphingomonadales;D_4__Sphingomonadaceae	uncultured
0,64%	0,75%	0,43%	0,80%	1,66%	1,15%	1,09%	1,44%	D_0__Bacteria;D_1__Proteobacteria;D_2__Alphaproteobacteria;D_3__Micropepsales;D_4__Micropepsaceae	uncultured
0,99%	0,90%	0,87%	1,11%	1,15%	1,42%	0,93%	0,36%	D_0__Bacteria;D_1__Bacteroidetes;D_2__Bacteroidia;D_3__Chitinophagales;D_4__Chitinophagaceae	Ferruginibacter
0,80%	0,68%	0,34%	1,08%	1,59%	1,09%	1,54%	0,31%	D_0__Bacteria;D_1__Patescibacteria;D_2__Saccharimonadia;D_3__Saccharimonadales;D_4__uncultured bacterium	
0,86%	0,53%	0,35%	0,78%	1,14%	0,65%	1,40%	1,46%	D_0__Bacteria;D_1__Gemmatimonadetes;D_2__Gemmatimonadetes;D_3__Gemmatimonadales;D_4__Gemmatimonad aceae	Gemmatimonas
0,91%	0,81%	0,51%	0,81%	0,94%	0,75%	0,62%	1,76%	D_0__Bacteria;D_1__Acidobacteria;D_2__Acidobacteriia;D_3__Solibacterales;D_4__Solibacteraceae (Subgroup 3)	Candidatus Solibacter
0,65%	0,86%	0,64%	1,02%	1,20%	0,82%	0,68%	1,13%	D_0__Bacteria;D_1__Acidobacteria;D_2__Acidobacteriia;D_3__Solibacterales;D_4__Solibacteraceae (Subgroup 3)	Bryobacter
0,58%	0,90%	0,79%	0,64%	0,91%	1,20%	0,80%	0,86%	D_0__Bacteria;D_1__Proteobacteria;D_2__Gammaproteobacteria;D_3__Gammaproteobacteria Incertae Sedis;D_4__Unknown Family	Acidibacter
0,68%	0,96%	0,93%	0,83%	0,62%	1,06%	0,65%	0,74%	D_0__Bacteria;D_1__Proteobacteria;D_2__Gammaproteobacteria;D_3__Betaproteobacteriales;D_4__TRA3-20	uncultured bacterium
1,35%	0,75%	1,27%	1,11%	0,43%	0,43%	0,64%	0,39%	D_0__Bacteria;D_1__Actinobacteria;D_2__Thermoleophilia;D_3__Solirubrobacterales;D_4__67-14	uncultured bacterium
0,82%	1,06%	0,64%	0,93%	0,52%	0,67%	0,67%	0,98%	D_0__Bacteria;D_1__Proteobacteria;D_2__Gammaproteobacteria;D_3__Betaproteobacteriales;D_4__SC-I-84	uncultured bacterium
0,40%	0,54%	0,66%	0,45%	0,69%	0,86%	0,88%	1,23%	D_0__Bacteria;D_1__Proteobacteria;D_2__Alphaproteobacteria;D_3__Reyranellales;D_4__Reyranellaceae	Reyranella
0,83%	0,79%	0,76%	0,73%	0,46%	0,78%	0,83%	0,49%	D_0__Bacteria;D_1__Actinobacteria;D_2__Acidimicrobiia;D_3__IMCC26256;D_4__uncultured bacterium	
0,55%	0,54%	0,93%	0,74%	0,54%	0,61%	0,94%	0,63%	D_0__Bacteria;D_1__Proteobacteria;D_2__Alphaproteobacteria;D_3__Rhizobiales;D_4__Xanthobacteraceae	Rhodoplanes
0,77%	0,79%	0,50%	0,53%	0,59%	0,81%	0,78%	0,70%	D_0__Bacteria;D_1__Proteobacteria;D_2__Gammaproteobacteria;D_3__Betaproteobacteriales;D_4__Nitrosomonadace ae	IS-44
0,43%	0,33%	0,69%	0,44%	0,99%	0,72%	0,59%	0,97%	D_0__Bacteria;D_1__Proteobacteria;D_2__Alphaproteobacteria;D_3__Caulobacterales;D_4__Caulobacteraceae	Phenylobacterium
0,22%	0,38%	1,81%	1,33%	0,69%	0,30%	0,15%	0,24%	D_0__Bacteria;D_1__Firmicutes;D_2__Bacilli;D_3__Bacillales;D_4__Bacillaceae	Bacillus
0,29%	0,44%	0,40%	0,38%	1,41%	0,75%	0,50%	0,66%	D_0__Bacteria;D_1__Proteobacteria;D_2__Alphaproteobacteria;D_3__Dongiales;D_4__Dongiaceae	Dongia
0,81%	0,58%	1,09%	0,60%	0,23%	0,36%	0,53%	0,25%	D_0__Bacteria;D_1__Actinobacteria;D_2__Thermoleophilia;D_3__Gaiellales;D_4__uncultured	uncultured bacterium
0,59%	0,98%	0,34%	0,49%	0,45%	0,84%	0,26%	0,47%	D_0__Bacteria;D_1__Verrucomicrobia;D_2__Verrucomicrobiae;D_3__Pedosphaerales;D_4__Pedosphaeraceae	uncultured bacterium
0,59%	0,40%	0,29%	0,58%	0,53%	0,47%	0,55%	0,44%	D_0__Bacteria;D_1__Actinobacteria;D_2__Acidimicrobiia;D_3__Microtrichales;D_4__Ilumatobacteraceae	CL500-29 marine group
0,60%	0,40%	0,46%	0,49%	0,20%	0,25%	0,54%	0,30%	D_0__Bacteria;D_1__Chloroflexi;D_2__KD4-96;D_3__uncultured bacterium;D_4__	
0,29%	0,37%	0,28%	0,32%	0,68%	0,43%	0,32%	0,43%	D_0__Bacteria;D_1__Proteobacteria;D_2__Gammaproteobacteria;D_3__Betaproteobacteriales;D_4__Burkholderiaceae	Rhizobacter
0,81%	0,29%	0,62%	0,40%	0,19%	0,20%	0,36%	0,16%	D_0__Bacteria;D_1__Actinobacteria;D_2__Thermoleophilia;D_3__Solirubrobacterales;D_4__Solirubrobacteraceae	Solirubrobacter
0,33%	0,20%	0,34%	0,30%	0,24%	0,44%	0,48%	0,48%	D_0__Bacteria;D_1__Proteobacteria;D_2__Alphaproteobacteria;D_3__Rhizobiales;D_4__Rhizobiales Incertae Sedis	uncultured
0,32%	0,41%	0,45%	0,25%	0,12%	0,47%	0,29%	0,36%	D_0__Bacteria;D_1__Proteobacteria;D_2__Gammaproteobacteria;D_3__Steroidobacterales;D_4__Steroidobacteraceae	uncultured
0,41%	0,41%	0,47%	0,23%	0,36%	0,39%	0,20%	0,20%	D_0__Bacteria;D_1__Acidobacteria;D_2__Blastocatellia (Subgroup 4);D_3__Blastocatellales;D_4__Blastocatellaceae	uncultured
0,19%	0,15%	0,21%	0,24%	0,53%	0,37%	0,41%	0,50%	D_0__Bacteria;D_1__Proteobacteria;D_2__Alphaproteobacteria;D_3__Rhizobiales;D_4__Devosiaceae	Devosia
0,29%	0,32%	0,37%	0,34%	0,15%	0,32%	0,38%	0,38%	D_0__Bacteria;D_1__Actinobacteria;D_2__Acidimicrobiia;D_3__Microtrichales;D_4__uncultured	uncultured bacterium
0,22%	0,39%	0,15%	0,14%	0,41%	1,04%	0,07%	0,10%	D_0__Bacteria;D_1__Bacteroidetes;D_2__Bacteroidia;D_3__Flavobacteriales;D_4__Flavobacteriaceae	Flavobacterium
0,40%	0,24%	0,23%	0,28%	0,27%	0,18%	0,33%	0,57%	D_0__Bacteria;D_1__Acidobacteria;D_2__Holophagae;D_3__Subgroup 7;D_4__uncultured bacterium	
0,14%	0,22%	0,18%	0,19%	0,46%	0,54%	0,34%	0,37%	D_0__Bacteria;D_1__Proteobacteria;D_2__Alphaproteobacteria;D_3__Caulobacterales;D_4__Hyphomonadaceae	Hirschia
0,44%	0,24%	0,41%	0,28%	0,24%	0,30%	0,31%	0,22%	D_0__Bacteria;D_1__Actinobacteria;D_2__Actinobacteria;D_3__Propionibacteriales;D_4__Nocardioidaceae	Nocardioides
0,48%	0,47%	0,42%	0,27%	0,10%	0,30%	0,24%	0,15%	D_0__Bacteria;D_1__Actinobacteria;D_2__Thermoleophilia;D_3__Gaiellales;D_4__Gaiellaceae	Gaiella
0,34%	0,38%	0,29%	0,38%	0,10%	0,48%	0,21%	0,09%	D_0__Bacteria;D_1__Bacteroidetes;D_2__Bacteroidia;D_3__Chitinophagales;D_4__Chitinophagaceae	Flavitalea
0,19%	0,22%	0,17%	0,29%	0,49%	0,30%	0,25%	0,37%	D_0__Bacteria;D_1__Proteobacteria;D_2__Alphaproteobacteria;D_3__Acetobacterales;D_4__Acetobacteraceae	uncultured
0,27%	0,27%	0,20%	0,24%	0,13%	0,29%	0,33%	0,51%	D_0__Bacteria;D_1__Proteobacteria;D_2__Alphaproteobacteria;D_3__Rhizobiales;D_4__uncultured	uncultured bacterium
0,14%	0,16%	0,24%	0,19%	0,77%	0,34%	0,11%	0,22%	D_0__Bacteria;D_1__Actinobacteria;D_2__Actinobacteria;D_3__Streptomycetales;D_4__Streptomycetaceae	Streptomyces

0,23%	0,20%	0,41%	0,26%	0,33%	0,27%	0,18%	0,27%	D_0__Bacteria;D_1__Actinobacteria;D_2__Actinobacteria;D_3__Corynebacteriales;D_4__Mycobacteriaceae	Mycobacterium
0,11%	0,26%	0,72%	0,20%	0,23%	0,13%	0,29%	0,21%	D_0__Bacteria;D_1__Proteobacteria;D_2__Gammaproteobacteria;D_3__Betaproteobacteriales;D_4__Burkholderiaceae	uncultured
0,22%	0,23%	0,34%	0,32%	0,14%	0,21%	0,36%	0,34%	D_0__Bacteria;D_1__Proteobacteria;D_2__Alphaproteobacteria;D_3__Rhizobiales;D_4__KF-JG30-B3	uncultured bacterium
0,16%	0,16%	0,23%	0,21%	0,49%	0,23%	0,32%	0,33%	D_0__Bacteria;D_1__Proteobacteria;D_2__Alphaproteobacteria;D_3__Caulobacterales;D_4__Caulobacteraceae	uncultured
0,29%	0,22%	0,16%	0,27%	0,43%	0,31%	0,21%	0,23%	D_0__Bacteria;D_1__Proteobacteria;D_2__Deltaproteobacteria;D_3__Myxococcales;D_4__Sandaracinaceae	uncultured
0,23%	0,24%	0,21%	0,22%	0,54%	0,19%	0,28%	0,17%	D_0__Bacteria;D_1__Proteobacteria;D_2__Gammaproteobacteria;D_3__Xanthomonadales;D_4__Xanthomonadaceae	Arenimonas
0,23%	0,29%	0,28%	0,22%	0,24%	0,37%	0,21%	0,22%	D_0__Bacteria;D_1__Acidobacteria;D_2__Blastocatellia (Subgroup 4);D_3__Blastocatellales;D_4__Blastocatellaceae	JGI 0001001-H03
0,19%	0,18%	0,20%	0,23%	0,41%	0,24%	0,26%	0,31%	D_0__Bacteria;D_1__Proteobacteria;D_2__Gammaproteobacteria;D_3__Betaproteobacteriales;D_4__Burkholderiaceae;Other	
0,20%	0,39%	0,13%	0,13%	0,27%	0,48%	0,24%	0,16%	D_0__Bacteria;D_1__Proteobacteria;D_2__Deltaproteobacteria;D_3__Myxococcales;D_4__BIrii41	uncultured bacterium
0,14%	0,27%	0,37%	0,34%	0,31%	0,28%	0,14%	0,14%	D_0__Bacteria;D_1__Actinobacteria;D_2__Actinobacteria;D_3__Propionibacteriales;D_4__Nocardioidaceae	Kribbella
0,12%	0,06%	0,15%	0,16%	0,93%	0,11%	0,14%	0,29%	D_0__Bacteria;D_1__Bacteroidetes;D_2__Bacteroidia;D_3__Sphingobacteriales;D_4__Sphingobacteriaceae	Mucilaginibacter
0,31%	0,36%	0,20%	0,18%	0,17%	0,47%	0,17%	0,02%	D_0__Bacteria;D_1__Bacteroidetes;D_2__Bacteroidia;D_3__Sphingobacteriales;D_4__env,OPS 17	uncultured bacterium
0,21%	0,27%	0,42%	0,25%	0,08%	0,12%	0,34%	0,14%	D_0__Bacteria;D_1__Proteobacteria;D_2__Alphaproteobacteria;D_3__Rhizobiales;D_4__Hyphomicrobiaceae	Pedomicrobium
0,11%	0,21%	0,10%	0,09%	0,23%	0,32%	0,43%	0,31%	D_0__Bacteria;D_1__Proteobacteria;D_2__Alphaproteobacteria;D_3__Caulobacterales;D_4__Hyphomonadaceae	SWB02
0,23%	0,22%	0,30%	0,17%	0,35%	0,18%	0,17%	0,14%	D_0__Bacteria;D_1__Acidobacteria;D_2__Blastocatellia (Subgroup 4);D_3__Blastocatellales;D_4__Blastocatellaceae	Stenotrophobacter
0,18%	0,10%	0,45%	0,14%	0,24%	0,24%	0,16%	0,21%	D_0__Bacteria;D_1__Proteobacteria;D_2__Alphaproteobacteria;D_3__Rhizobiales;D_4__Rhizobiaceae	Mesorhizobium
0,12%	0,65%	0,18%	0,13%	0,09%	0,33%	0,12%	0,07%	D_0__Bacteria;D_1__Bacteroidetes;D_2__Bacteroidia;D_3__Chitinophagales;D_4__Saprospiraceae	uncultured
0,22%	0,27%	0,11%	0,14%	0,28%	0,32%	0,18%	0,16%	D_0__Bacteria;D_1__Proteobacteria;D_2__Deltaproteobacteria;D_3__Myxococcales;D_4__Phaselicystidaceae	Phaselicystis
0,17%	0,43%	0,32%	0,13%	0,11%	0,27%	0,09%	0,13%	D_0__Bacteria;D_1__Proteobacteria;D_2__Gammaproteobacteria;D_3__CCD24;D_4__uncultured bacterium	
0,21%	0,21%	0,30%	0,18%	0,21%	0,16%	0,22%	0,14%	D_0__Bacteria;D_1__Actinobacteria;D_2__Acidimicrobiia;D_3__Microtrichales;D_4__Ilumatobacteraceae	uncultured
0,06%	0,05%	0,15%	0,11%	0,90%	0,09%	0,16%	0,08%	D_0__Bacteria;D_1__Actinobacteria;D_2__Actinobacteria;D_3__Micromonosporales;D_4__Micromonosporaceae	Actinoplanes
0,14%	0,04%	0,18%	0,09%	0,80%	0,07%	0,09%	0,18%	D_0__Bacteria;D_1__Proteobacteria;D_2__Alphaproteobacteria;D_3__Sphingomonadales;D_4__Sphingomonadaceae	Novosphingobium
0,26%	0,24%	0,18%	0,17%	0,15%	0,24%	0,18%	0,16%	D_0__Bacteria;D_1__Actinobacteria;D_2__Acidimicrobiia;D_3__IMCC26256;Other;Other	
0,21%	0,17%	0,30%	0,16%	0,10%	0,24%	0,30%	0,10%	D_0__Bacteria;D_1__Actinobacteria;D_2__Acidimicrobiia;D_3__Microtrichales;D_4__Iamiaceae	Iamia
0,22%	0,23%	0,15%	0,16%	0,07%	0,22%	0,24%	0,25%	D_0__Bacteria;D_1__Proteobacteria;D_2__Deltaproteobacteria;D_3__Desulfarculales;D_4__Desulfarculaceae	uncultured
0,43%	0,05%	0,27%	0,27%	0,27%	0,04%	0,16%	0,06%	D_0__Bacteria;D_1__Bacteroidetes;D_2__Bacteroidia;D_3__Chitinophagales;D_4__Chitinophagaceae	Flavisolibacter
0,11%	0,17%	0,19%	0,13%	0,24%	0,24%	0,20%	0,24%	D_0__Bacteria;D_1__Proteobacteria;D_2__Alphaproteobacteria;D_3__Rhizobiales;D_4__Hyphomicrobiaceae	Hyphomicrobium
0,17%	0,17%	0,11%	0,20%	0,19%	0,21%	0,18%	0,26%	D_0__Bacteria;D_1__Proteobacteria;D_2__Gammaproteobacteria;D_3__Betaproteobacteriales;D_4__TRA3-20	metagenome
0,12%	0,24%	0,47%	0,23%	0,03%	0,16%	0,11%	0,11%	D_0__Bacteria;D_1__Proteobacteria;D_2__Deltaproteobacteria;D_3__Myxococcales;D_4__bacteriap25	uncultured bacterium
0,09%	0,18%	0,24%	0,11%	0,16%	0,34%	0,11%	0,21%	D_0__Bacteria;D_1__Proteobacteria;D_2__Gammaproteobacteria;D_3__Steroidobacterales;D_4__Steroidobacteraceae	Steroidobacter
0,13%	0,06%	0,65%	0,26%	0,05%	0,03%	0,18%	0,01%	D_0__Bacteria;D_1__Actinobacteria;D_2__Rubrobacteria;D_3__Rubrobacterales;D_4__Rubrobacteriaceae	Rubrobacter
0,16%	0,11%	0,10%	0,11%	0,37%	0,22%	0,08%	0,20%	D_0__Bacteria;D_1__Proteobacteria;D_2__Gammaproteobacteria;D_3__Pseudomonadales;D_4__Pseudomonadaceae	Pseudomonas
0,16%	0,14%	0,14%	0,16%	0,05%	0,08%	0,46%	0,15%	D_0__Bacteria;D_1__Acidobacteria;D_2__Subgroup 17;D_3__uncultured bacterium;D_4__	
0,14%	0,26%	0,16%	0,17%	0,18%	0,21%	0,11%	0,06%	D_0__Bacteria;D_1__Proteobacteria;D_2__Deltaproteobacteria;D_3__Bdellovibrionales;D_4__Bdellovibrionaceae	Bdellovibrio
0,16%	0,09%	0,13%	0,16%	0,24%	0,12%	0,17%	0,19%	D_0__Bacteria;D_1__Actinobacteria;D_2__Actinobacteria;D_3__Pseudonocardiales;D_4__Pseudonocardiaceae	Pseudonocardia
0,14%	0,25%	0,15%	0,17%	0,07%	0,24%	0,15%	0,08%	D_0__Bacteria;D_1__Bacteroidetes;D_2__Bacteroidia;D_3__Sphingobacteriales;D_4__AKYH767	uncultured bacterium
0,11%	0,11%	0,11%	0,14%	0,20%	0,10%	0,22%	0,23%	D_0__Bacteria;D_1__Proteobacteria;D_2__Alphaproteobacteria;D_3__Rhizobiales;D_4__Xanthobacteraceae	Pseudolabrys
0,15%	0,26%	0,10%	0,18%	0,11%	0,14%	0,12%	0,16%	D_0__Bacteria;D_1__Proteobacteria;D_2__Gammaproteobacteria;D_3__Betaproteobacteriales;D_4__SC-I-84;Other	
0,09%	0,11%	0,16%	0,11%	0,10%	0,18%	0,27%	0,20%	D_0__Bacteria;D_1__Proteobacteria;D_2__Alphaproteobacteria;D_3__Sphingomonadales;D_4__Sphingomonadaceae	Ellin6055
0,21%	0,10%	0,13%	0,20%	0,15%	0,09%	0,12%	0,23%	D_0__Bacteria;D_1__Proteobacteria;D_2__Alphaproteobacteria;D_3__Elsterales;D_4__uncultured	uncultured bacterium
0,12%	0,14%	0,08%	0,16%	0,13%	0,15%	0,22%	0,21%	D_0__Bacteria;D_1__Proteobacteria;D_2__Alphaproteobacteria;D_3__Rhizobiales;D_4__Rhizobiales Incertae Sedis	Bauldia
0,18%	0,15%	0,11%	0,10%	0,18%	0,14%	0,12%	0,18%	D_0__Bacteria;D_1__Proteobacteria;D_2__Gammaproteobacteria;D_3__Betaproteobacteriales;D_4__Burkholderiaceae	Ramlibacter
0,18%	0,15%	0,16%	0,15%	0,10%	0,09%	0,21%	0,12%	D_0__Bacteria;D_1__Acidobacteria;D_2__Thermoanaerobaculia;D_3__Thermoanaerobaculales;D_4__Thermoanaerobaculaceae	Subgroup 10
0,10%	0,28%	0,09%	0,11%	0,13%	0,19%	0,09%	0,15%	D_0__Bacteria;D_1__Acidobacteria;D_2__Subgroup 5;D_3__uncultured Acidobacteria bacterium;D_4__	
0,09%	0,13%	0,10%	0,11%	0,23%	0,16%	0,13%	0,20%	D_0__Bacteria;D_1__Proteobacteria;D_2__Gammaproteobacteria;D_3__Betaproteobacteriales;D_4__Burkholderiaceae	Massilia
0,07%	0,18%	0,28%	0,12%	0,14%	0,11%	0,11%	0,14%	D_0__Bacteria;D_1__Acidobacteria;D_2__Acidobacteriia;D_3__Solibacterales;D_4__Solibacteraceae (Subgroup 3)	Paludibaculum
0,04%	0,30%	0,13%	0,04%	0,08%	0,46%	0,03%	0,05%	D_0__Bacteria;D_1__Bacteroidetes;D_2__Bacteroidia;D_3__Cytophagales;D_4__Hymenobacteraceae	Adhaeribacter
0,10%	0,14%	0,20%	0,15%	0,11%	0,13%	0,16%	0,14%	D_0__Bacteria;D_1__Proteobacteria;D_2__Gammaproteobacteria;D_3__Betaproteobacteriales;D_4__TRA3-20;Other	
0,08%	0,08%	0,08%	0,09%	0,21%	0,12%	0,04%	0,41%	D_0__Bacteria;D_1__Proteobacteria;D_2__Gammaproteobacteria;D_3__Betaproteobacteriales;D_4__Burkholderiaceae	Burkholderia-Caballeronia-Paraburkholderia
0,18%	0,17%	0,12%	0,10%	0,11%	0,19%	0,12%	0,10%	D_0__Bacteria;D_1__Actinobacteria;D_2__Acidimicrobiia;D_3__Microtrichales;D_4__Ilumatobacteraceae	Ilumatobacter
0,05%	0,09%	0,06%	0,07%	0,22%	0,22%	0,16%	0,20%	D_0__Bacteria;D_1__Proteobacteria;D_2__Alphaproteobacteria;D_3__Rhizobiales;D_4__KF-JG30-B3	metagenome
0,15%	0,15%	0,17%	0,16%	0,07%	0,12%	0,15%	0,11%	D_0__Bacteria;D_1__Proteobacteria;D_2__Alphaproteobacteria;D_3__Elsterales;D_4__uncultured;Other	
0,15%	0,20%	0,12%	0,09%	0,12%	0,20%	0,08%	0,11%	D_0__Bacteria;D_1__Verrucomicrobia;D_2__Verrucomicrobiae;D_3__Pedosphaerales;D_4__Pedosphaeraceae;Other	
0,12%	0,11%	0,16%	0,16%	0,07%	0,08%	0,15%	0,20%	D_0__Bacteria;D_1__Acidobacteria;D_2__Blastocatellia (Subgroup 4);D_3__Elev-16S-573;D_4__uncultured bacterium	
0,05%	0,19%	0,06%	0,06%	0,10%	0,09%	0,07%	0,41%	D_0__Bacteria;D_1__Acidobacteria;D_2__Blastocatellia (Subgroup 4);D_3__Blastocatellales;D_4__Blastocatellaceae;Other	
0,12%	0,11%	0,27%	0,22%	0,04%	0,05%	0,08%	0,15%	D_0__Bacteria;D_1__Proteobacteria;D_2__Deltaproteobacteria;D_3__Myxococcales;D_4__bacteriap25;Other	
0,13%	0,20%	0,14%	0,08%	0,08%	0,16%	0,18%	0,06%	D_0__Bacteria;D_1__Planctomycetes;D_2__OM190;D_3__uncultured bacterium;D_4__	
0,08%	0,15%	0,09%	0,05%	0,10%	0,17%	0,18%	0,21%	D_0__Bacteria;D_1__Proteobacteria;D_2__Alphaproteobacteria;D_3__Rhizobiales;D_4__A0839	uncultured bacterium
0,11%	0,16%	0,08%	0,05%	0,19%	0,13%	0,17%	0,13%	D_0__Bacteria;D_1__Proteobacteria;D_2__Deltaproteobacteria;D_3__Myxococcales;D_4__BIrii41	metagenome
0,09%	0,14%	0,07%	0,14%	0,15%	0,17%	0,17%	0,09%	D_0__Bacteria;D_1__Proteobacteria;D_2__Deltaproteobacteria;D_3__Myxococcales;D_4__Polyangiaceae	Pajaroellobacter
0,07%	0,06%	0,09%	0,11%	0,28%	0,11%	0,11%	0,19%	D_0__Bacteria;D_1__Proteobacteria;D_2__Alphaproteobacteria;D_3__Rhizobiales;D_4__Rhizobiaceae	Allorhizobium-Neorhizobium-Pararhizobium-Rhizobium
0,08%	0,09%	0,09%	0,10%	0,25%	0,16%	0,09%	0,13%	D_0__Bacteria;D_1__Proteobacteria;D_2__Alphaproteobacteria;D_3__Rhodospirillales;D_4__Rhodospirillaceae	uncultured
0,10%	0,28%	0,32%	0,09%	0,02%	0,09%	0,07%	0,04%	D_0__Bacteria;D_1__Actinobacteria;D_2__MB-A2-108;D_3__uncultured bacterium;D_4__	
0,12%	0,05%	0,10%	0,18%	0,20%	0,05%	0,13%	0,18%	D_0__Bacteria;D_1__Planctomycetes;D_2__Phycisphaerae;D_3__Tepidisphaerales;D_4__WD2101 soil group;Other	
0,25%	0,16%	0,11%	0,13%	0,06%	0,11%	0,16%	0,02%	D_0__Bacteria;D_1__Patescibacteria;D_2__Parcubacteria;D_3__Candidatus Kaiserbacteria;D_4__uncultured bacterium	
0,08%	0,15%	0,18%	0,06%	0,14%	0,28%	0,06%	0,04%	D_0__Bacteria;D_1__Bacteroidetes;D_2__Bacteroidia;D_3__Cytophagales;D_4__Microscillaceae	Ohtaekwangia
0,09%	0,10%	0,08%	0,09%	0,20%	0,22%	0,09%	0,08%	D_0__Bacteria;D_1__Verrucomicrobia;D_2__Verrucomicrobiae;D_3__Opitutales;D_4__Opitutaceae	Opitutus
0,26%	0,08%	0,09%	0,15%	0,09%	0,08%	0,14%	0,08%	D_0__Bacteria;D_1__Actinobacteria;D_2__Thermoleophilia;D_3__Solirubrobacterales;D_4__Solirubrobacteraceae	Conexibacter
0,07%	0,06%	0,12%	0,09%	0,15%	0,13%	0,14%	0,16%	D_0__Bacteria;D_1__Proteobacteria;D_2__Alphaproteobacteria;D_3__Rhizobiales;D_4__Xanthobacteraceae;Other	
0,10%	0,09%	0,12%	0,13%	0,20%	0,11%	0,07%	0,10%	D_0__Bacteria;D_1__Proteobacteria;D_2__Gammaproteobacteria;D_3__Xanthomonadales;D_4__Xanthomonadaceae	Lysobacter
0,17%	0,17%	0,12%	0,08%	0,10%	0,10%	0,09%	0,08%	D_0__Bacteria;D_1__Planctomycetes;D_2__Phycisphaerae;D_3__Phycisphaerales;D_4__Phycisphaeraceae	SM1A02
0,07%	0,06%	0,12%	0,05%	0,29%	0,10%	0,10%	0,12%	D_0__Bacteria;D_1__Proteobacteria;D_2__Alphaproteobacteria;D_3__Caulobacterales;D_4__Caulobacteraceae	Caulobacter
0,09%	0,13%	0,15%	0,06%	0,19%	0,14%	0,08%	0,07%	D_0__Bacteria;D_1__Actinobacteria;D_2__Actinobacteria;D_3__Micromonosporales;D_4__Micromonosporaceae	Luedemannella
0,09%	0,16%	0,09%	0,09%	0,11%	0,16%	0,05%	0,09%	D_0__Bacteria;D_1__Verrucomicrobia;D_2__Verrucomicrobiae;D_3__Pedosphaerales;D_4__Pedosphaeraceae	metagenome
0,13%	0,18%	0,05%	0,09%	0,13%	0,15%	0,05%	0,06%	D_0__Bacteria;D_1__Bacteroidetes;D_2__Bacteroidia;D_3__Chitinophagales;D_4__Chitinophagaceae	Parafilimonas
0,17%	0,12%	0,15%	0,09%	0,05%	0,06%	0,13%	0,08%	D_0__Bacteria;D_1__Chloroflexi;D_2__TK10;D_3__uncultured bacterium;D_4__	
0,10%	0,06%	0,11%	0,14%	0,10%	0,07%	0,17%	0,09%	D_0__Bacteria;D_1__Actinobacteria;D_2__Acidimicrobiia;D_3__uncultured;Other;Other	
0,09%	0,06%	0,07%	0,07%	0,13%	0,14%	0,15%	0,12%	D_0__Bacteria;D_1__Proteobacteria;D_2__Gammaproteobacteria;D_3__Diplorickettsiales;D_4__Diplorickettsiaceae	uncultured
0,12%	0,05%	0,06%	0,15%	0,05%	0,09%	0,20%	0,10%	D_0__Bacteria;D_1__Actinobacteria;D_2__Acidimicrobiia;D_3__uncultured;D_4__uncultured bacterium	
0,07%	0,17%	0,14%	0,08%	0,11%	0,16%	0,05%	0,04%	D_0__Bacteria;D_1__Bacteroidetes;D_2__Bacteroidia;D_3__Cytophagales;D_4__Microscillaceae	Chryseolinea
0,06%	0,12%	0,05%	0,08%	0,10%	0,16%	0,11%	0,12%	D_0__Bacteria;D_1__Proteobacteria;D_2__Gammaproteobacteria;D_3__Xanthomonadales;D_4__Rhodanobacteraceae	Dokdonella
0,05%	0,08%	0,04%	0,08%	0,19%	0,17%	0,14%	0,04%	D_0__Bacteria;D_1__Patescibacteria;D_2__Saccharimonadia;D_3__Saccharimonadales;Other;Other	
0,20%	0,06%	0,07%	0,12%	0,10%	0,04%	0,09%	0,10%	D_0__Bacteria;D_1__Elusimicrobia;D_2__Lineage IIa;D_3__uncultured bacterium;D_4__	

0,17%	0,11%	0,10%	0,11%	0,05%	0,06%	0,13%	0,06%	D_0__Bacteria;D_1__Planctomycetes;D_2__Planctomycetacia;D_3__Gemmatales;D_4__Gemmataceae	uncultured
0,29%	0,14%	0,06%	0,12%	0,04%	0,06%	0,06%	0,02%	D_0__Bacteria;D_1__Patescibacteria;D_2__Parcubacteria;D_3__uncultured bacterium;D_4__	
0,13%	0,11%	0,07%	0,10%	0,11%	0,08%	0,12%	0,05%	D_0__Bacteria;D_1__Proteobacteria;D_2__Deltaproteobacteria;D_3__Oligoflexales;D_4__0319-6G20	uncultured bacterium
0,10%	0,10%	0,15%	0,08%	0,06%	0,10%	0,11%	0,05%	D_0__Bacteria;D_1__Chloroflexi;D_2__Dehalococcoidia;D_3__S085;D_4__uncultured bacterium	
0,05%	0,05%	0,07%	0,07%	0,15%	0,07%	0,14%	0,14%	D_0__Bacteria;D_1__Proteobacteria;D_2__Alphaproteobacteria;D_3__Sphingomonadales;D_4__Sphingomonadaceae;Other	
0,09%	0,10%	0,08%	0,08%	0,07%	0,09%	0,11%	0,10%	D_0__Bacteria;D_1__Acidobacteria;D_2__Subgroup 6;D_3__uncultured Acidobacteriales bacterium;D_4__	
0,09%	0,09%	0,08%	0,09%	0,08%	0,14%	0,12%	0,03%	D_0__Bacteria;D_1__Bacteroidetes;D_2__Bacteroidia;D_3__Chitinophagales;D_4__Chitinophagaceae;Other	
0,13%	0,07%	0,10%	0,12%	0,04%	0,08%	0,12%	0,08%	D_0__Bacteria;D_1__Proteobacteria;D_2__Alphaproteobacteria;D_3__Rhizobiales;D_4__Rhizobiales Incertae Sedis	Nordella
0,12%	0,23%	0,12%	0,18%	0,01%	0,02%	0,02%	0,02%	D_0__Bacteria;D_1__Nitrospirae;D_2__Nitrospira;D_3__Nitrospirales;D_4__Nitrospiraceae	Nitrospira
0,07%	0,14%	0,16%	0,11%	0,01%	0,04%	0,12%	0,06%	D_0__Bacteria;D_1__Proteobacteria;D_2__Gammaproteobacteria;D_3__PLTA13;D_4__uncultured bacterium	
0,07%	0,08%	0,06%	0,07%	0,07%	0,08%	0,11%	0,18%	D_0__Bacteria;D_1__Proteobacteria;D_2__Alphaproteobacteria;D_3__uncultured;D_4__uncultured bacterium	
0,12%	0,12%	0,08%	0,07%	0,07%	0,10%	0,11%	0,05%	D_0__Bacteria;D_1__Proteobacteria;D_2__Deltaproteobacteria;D_3__Bdellovibrionales;D_4__Bdellovibrionaceae	OM27 clade
0,20%	0,02%	0,01%	0,17%	0,08%	0,01%	0,07%	0,15%	D_0__Bacteria;D_1__Acidobacteria;D_2__Acidobacteriia;D_3__Acidobacteriales;D_4__uncultured	uncultured bacterium
0,17%	0,16%	0,07%	0,07%	0,07%	0,05%	0,08%	0,00%	D_0__Bacteria;D_1__Patescibacteria;D_2__Parcubacteria;D_3__Candidatus Adlerbacteria;D_4__uncultured bacterium	
0,03%	0,08%	0,16%	0,10%	0,10%	0,09%	0,02%	0,09%	D_0__Bacteria;D_1__Proteobacteria;D_2__Gammaproteobacteria;D_3__Cellvibrionales;D_4__Halieaceae	OM60(NOR5) clade
0,12%	0,14%	0,04%	0,09%	0,04%	0,13%	0,06%	0,06%	D_0__Bacteria;D_1__Bacteroidetes;D_2__Bacteroidia;D_3__Chitinophagales;D_4__uncultured	uncultured bacterium
0,06%	0,06%	0,07%	0,06%	0,30%	0,05%	0,02%	0,04%	D_0__Bacteria;D_1__Bacteroidetes;D_2__Bacteroidia;D_3__Chitinophagales;D_4__Chitinophagaceae	Chitinophaga
0,17%	0,15%	0,06%	0,05%	0,04%	0,07%	0,11%	0,00%	D_0__Bacteria;D_1__Patescibacteria;D_2__Parcubacteria;D_3__Candidatus Nomurabacteria;Other;Other	
0,07%	0,06%	0,06%	0,04%	0,16%	0,08%	0,08%	0,09%	D_0__Bacteria;D_1__Proteobacteria;D_2__Gammaproteobacteria;D_3__Betaproteobacteriales;D_4__Burkholderiaceae	Variovorax
0,07%	0,12%	0,12%	0,08%	0,08%	0,08%	0,05%	0,06%	D_0__Bacteria;D_1__Verrucomicrobia;D_2__Verrucomicrobiae;D_3__Verrucomicrobiales;D_4__Verrucomicrobiaceae	uncultured
0,08%	0,07%	0,10%	0,13%	0,02%	0,06%	0,07%	0,12%	D_0__Bacteria;D_1__Acidobacteria;D_2__Holophagae;D_3__Subgroup 7;Other;Other	
0,04%	0,08%	0,04%	0,06%	0,16%	0,17%	0,04%	0,05%	D_0__Bacteria;D_1__Verrucomicrobia;D_2__Verrucomicrobiae;D_3__Opitutales;D_4__Opitutaceae	Lacunisphaera
0,07%	0,16%	0,15%	0,09%	0,01%	0,02%	0,10%	0,03%	D_0__Bacteria;D_1__Rokubacteria;D_2__NC10;D_3__Rokubacteriales;D_4__uncultured bacterium	
0,08%	0,05%	0,07%	0,04%	0,15%	0,11%	0,05%	0,08%	D_0__Bacteria;D_1__Proteobacteria;D_2__Alphaproteobacteria;D_3__Sphingomonadales;D_4__Sphingomonadaceae	Sphingobium
0,08%	0,12%	0,02%	0,08%	0,07%	0,11%	0,04%	0,09%	D_0__Bacteria;D_1__Proteobacteria;D_2__Gammaproteobacteria;D_3__Xanthomonadales;D_4__Rhodanobacteraceae	Rhodanobacter
0,10%	0,14%	0,06%	0,07%	0,04%	0,12%	0,04%	0,04%	D_0__Bacteria;D_1__Bacteroidetes;D_2__Bacteroidia;D_3__Sphingobacteriales;D_4__AKYH767;Other	
0,07%	0,04%	0,06%	0,11%	0,13%	0,05%	0,09%	0,05%	D_0__Bacteria;D_1__Actinobacteria;D_2__Actinobacteria;D_3__Frankiales;D_4__Frankiaceae	Jatrophihabitans
0,09%	0,06%	0,08%	0,06%	0,17%	0,07%	0,01%	0,05%	D_0__Bacteria;D_1__Verrucomicrobia;D_2__Verrucomicrobiae;D_3__Verrucomicrobiales;D_4__Rubritaleaceae	Luteolibacter
0,14%	0,07%	0,07%	0,10%	0,03%	0,05%	0,07%	0,06%	D_0__Bacteria;D_1__Actinobacteria;D_2__Thermoleophilia;D_3__Gaiellales;D_4__uncultured;Other	
0,04%	0,14%	0,11%	0,06%	0,04%	0,05%	0,07%	0,06%	D_0__Bacteria;D_1__Proteobacteria;D_2__Alphaproteobacteria;D_3__Rhizobiales;D_4__Methyloligellaceae	uncultured
0,05%	0,10%	0,05%	0,05%	0,07%	0,07%	0,07%	0,11%	D_0__Bacteria;D_1__Proteobacteria;D_2__Alphaproteobacteria;D_3__Rhizobiales;D_4__KF-JG30-B3;Other	
0,06%	0,06%	0,05%	0,08%	0,04%	0,07%	0,14%	0,07%	D_0__Bacteria;D_1__Acidobacteria;D_2__Subgroup 17;Other;Other;Other	
0,07%	0,08%	0,19%	0,08%	0,05%	0,03%	0,06%	0,02%	D_0__Bacteria;D_1__Verrucomicrobia;D_2__Verrucomicrobiae;D_3__Chthoniobacterales;D_4__Xiphinematobacteraceae	Candidatus Xiphinematobacter
0,08%	0,11%	0,04%	0,07%	0,06%	0,10%	0,05%	0,06%	D_0__Bacteria;D_1__Bacteroidetes;D_2__Bacteroidia;D_3__Sphingobacteriales;D_4__AKYH767	metagenome
0,04%	0,03%	0,05%	0,04%	0,05%	0,09%	0,12%	0,13%	D_0__Bacteria;D_1__Proteobacteria;D_2__Alphaproteobacteria;D_3__Reyranellales;D_4__Reyranellaceae	uncultured
0,06%	0,03%	0,07%	0,04%	0,20%	0,09%	0,02%	0,03%	D_0__Bacteria;D_1__Bacteroidetes;D_2__Bacteroidia;D_3__Chitinophagales;D_4__Chitinophagaceae	Niastella
0,04%	0,06%	0,03%	0,07%	0,13%	0,09%	0,07%	0,07%	D_0__Bacteria;D_1__Proteobacteria;D_2__Gammaproteobacteria;D_3__Diplorickettsiales;D_4__Diplorickettsiaceae	Aquicella
0,10%	0,09%	0,07%	0,08%	0,02%	0,05%	0,08%	0,05%	D_0__Bacteria;D_1__Proteobacteria;D_2__Deltaproteobacteria;D_3__Myxococcales;D_4__Polyangiaceae	uncultured
0,08%	0,03%	0,06%	0,09%	0,10%	0,04%	0,06%	0,08%	D_0__Bacteria;D_1__Planctomycetes;D_2__Phycisphaerae;D_3__Tepidisphaerales;D_4__WD2101 soil group	uncultured planctomycete
0,05%	0,04%	0,05%	0,05%	0,14%	0,06%	0,08%	0,08%	D_0__Bacteria;D_1__Proteobacteria;D_2__Alphaproteobacteria;D_3__Rhodospirillales;D_4__uncultured	uncultured bacterium
0,07%	0,04%	0,06%	0,06%	0,20%	0,04%	0,03%	0,04%	D_0__Bacteria;D_1__Bacteroidetes;D_2__Bacteroidia;D_3__Sphingobacteriales;D_4__Sphingobacteriaceae	Pedobacter
0,04%	0,05%	0,05%	0,05%	0,05%	0,05%	0,06%	0,17%	D_0__Bacteria;D_1__Proteobacteria;D_2__Alphaproteobacteria;D_3__uncultured;Other;Other	
0,06%	0,06%	0,07%	0,05%	0,02%	0,06%	0,17%	0,04%	D_0__Bacteria;D_1__Acidobacteria;D_2__Subgroup 17;D_3__uncultured Acidobacteria bacterium;D_4__	
0,05%	0,05%	0,04%	0,04%	0,13%	0,10%	0,06%	0,05%	D_0__Bacteria;D_1__Proteobacteria;D_2__Alphaproteobacteria;D_3__Rickettsiales;D_4__SM2D12	metagenome
0,07%	0,08%	0,08%	0,05%	0,08%	0,10%	0,00%	0,07%	D_0__Bacteria;D_1__Bacteroidetes;D_2__Bacteroidia;D_3__Bacteroidales;D_4__Prevotellaceae	Prevotella 7
0,19%	0,02%	0,05%	0,10%	0,07%	0,03%	0,06%	0,00%	D_0__Bacteria;D_1__Actinobacteria;D_2__Thermoleophilia;D_3__Solirubrobacterales;D_4__Solirubrobacteraceae	uncultured
0,06%	0,08%	0,05%	0,05%	0,05%	0,13%	0,08%	0,01%	D_0__Bacteria;D_1__Bacteroidetes;D_2__Bacteroidia;D_3__Sphingobacteriales;D_4__env,OPS 17;Other	
0,05%	0,04%	0,07%	0,07%	0,10%	0,05%	0,06%	0,07%	D_0__Bacteria;D_1__Proteobacteria;D_2__Alphaproteobacteria;D_3__Sphingomonadales;D_4__Sphingomonadaceae	Altererythrobacter
0,09%	0,04%	0,10%	0,06%	0,07%	0,06%	0,05%	0,03%	D_0__Bacteria;D_1__Actinobacteria;D_2__Actinobacteria;D_3__Frankiales;D_4__Geodermatophilaceae	Blastococcus
0,06%	0,05%	0,08%	0,05%	0,05%	0,08%	0,07%	0,07%	D_0__Bacteria;D_1__Actinobacteria;D_2__Acidimicrobiia;D_3__Microtrichales;D_4__uncultured;Other	
0,27%	0,02%	0,02%	0,08%	0,04%	0,01%	0,03%	0,03%	D_0__Bacteria;D_1__Proteobacteria;D_2__Deltaproteobacteria;D_3__Myxococcales;D_4__Archangiaceae	Anaeromyxobacter
0,10%	0,03%	0,09%	0,07%	0,06%	0,05%	0,06%	0,02%	D_0__Bacteria;D_1__Chloroflexi;D_2__Chloroflexia;D_3__Thermomicrobiales;D_4__JG30-KF-CM45	uncultured bacterium
0,03%	0,03%	0,05%	0,06%	0,10%	0,05%	0,07%	0,10%	D_0__Bacteria;D_1__Proteobacteria;D_2__Alphaproteobacteria;D_3__Rhizobiales;Other;Other	
0,06%	0,06%	0,21%	0,05%	0,01%	0,01%	0,07%	0,01%	D_0__Bacteria;D_1__Actinobacteria;D_2__Actinobacteria;D_3__Propionibacteriales;D_4__Propionibacteriaceae	Microlunatus
0,07%	0,09%	0,05%	0,08%	0,05%	0,06%	0,03%	0,05%	D_0__Bacteria;D_1__Proteobacteria;D_2__Gammaproteobacteria;D_3__Betaproteobacteriales;D_4__Nitrosomonadaceae	mle1-7
0,05%	0,08%	0,06%	0,05%	0,06%	0,05%	0,09%	0,02%	D_0__Bacteria;D_1__Planctomycetes;D_2__Planctomycetacia;D_3__Pirellulales;D_4__Pirellulaceae	uncultured
0,05%	0,04%	0,10%	0,07%	0,06%	0,05%	0,04%	0,05%	D_0__Bacteria;D_1__Proteobacteria;D_2__Alphaproteobacteria;D_3__Rhizobiales;D_4__Beijerinckiaceae	Microvirga
0,06%	0,06%	0,02%	0,11%	0,03%	0,02%	0,06%	0,09%	D_0__Bacteria;D_1__Acidobacteria;D_2__Acidobacteriia;D_3__Subgroup 2;D_4__uncultured bacterium	
0,08%	0,03%	0,10%	0,07%	0,05%	0,03%	0,06%	0,02%	D_0__Bacteria;D_1__Actinobacteria;D_2__Actinobacteria;D_3__Frankiales;D_4__Sporichthyaceae	uncultured
0,07%	0,07%	0,05%	0,04%	0,01%	0,06%	0,05%	0,09%	D_0__Bacteria;D_1__Actinobacteria;D_2__MB-A2-108;Other;Other;Other	
0,07%	0,14%	0,03%	0,06%	0,01%	0,05%	0,07%	0,02%	D_0__Bacteria;D_1__Latescibacteria;D_2__uncultured bacterium;D_3__;D_4__	

(F)

PI1c	PI2c	PI3c	PI4c	PII1c	PII2c	PII3c	PII4c	#OTU ID	
14,65%	15,04%	14,64%	14,53%	16,95%	14,80%	15,08%	18,08%	Unassigned;Other;Other;Other;Other;Other	
8,43%	1,85%	1,59%	6,48%	3,41%	3,59%	3,85%	6,89%	D_0__Bacteria;D_1__Verrucomicrobia;D_2__Verrucomicrobiae;D_3__Chthoniobacterales;D_4__Chthoniobacteraceae	Candidatus Udaeobacter
5,23%	2,05%	2,96%	3,87%	2,82%	2,37%	3,16%	4,62%	D_0__Bacteria;D_1__Proteobacteria;D_2__Alphaproteobacteria;D_3__Sphingomonadales;D_4__Sphingomonadaceae	Sphingomonas
4,99%	2,50%	3,03%	4,59%	2,19%	1,49%	1,75%	2,86%	D_0__Bacteria;D_1__Planctomycetes;D_2__Phycisphaerae;D_3__Tepidisphaerales;D_4__WD2101 soil group	uncultured bacterium
1,93%	4,45%	3,31%	2,19%	0,57%	5,29%	1,88%	2,50%	D_0__Bacteria;D_1__Bacteroidetes;D_2__Bacteroidia;D_3__Chitinophagales;D_4__Chitinophagaceae	uncultured
1,77%	5,40%	3,31%	2,08%	0,69%	3,31%	2,72%	1,99%	D_0__Bacteria;D_1__Acidobacteria;D_2__Subgroup 6;D_3__uncultured Acidobacteria bacterium;D_4__	
1,93%	4,26%	3,13%	2,17%	0,79%	2,24%	2,97%	1,92%	D_0__Bacteria;D_1__Acidobacteria;D_2__Subgroup 6;D_3__uncultured bacterium;D_4__	
4,03%	0,99%	0,99%	2,58%	2,32%	0,88%	2,40%	1,42%	D_0__Bacteria;D_1__Gemmatimonadetes;D_2__Gemmatimonadetes;D_3__Gemmatimonadales;D_4__Gemmatimonadaceae	uncultured
0,42%	4,44%	2,86%	0,79%	0,22%	3,28%	1,40%	2,17%	D_0__Bacteria;D_1__Bacteroidetes;D_2__Bacteroidia;D_3__Cytophagales;D_4__Microscillaceae	uncultured
1,59%	3,04%	2,03%	1,73%	1,11%	1,57%	2,14%	1,70%	D_0__Bacteria;D_1__Proteobacteria;D_2__Gammaproteobacteria;D_3__Betaproteobacteriales;D_4__Nitrosomonadaceae	MND1
2,29%	1,27%	1,37%	2,10%	2,07%	1,51%	2,02%	1,88%	D_0__Bacteria;D_1__Proteobacteria;D_2__Alphaproteobacteria;D_3__Rhizobiales;D_4__Xanthobacteraceae	uncultured
1,37%	0,53%	0,90%	2,63%	4,23%	1,18%	2,36%	1,29%	D_0__Bacteria;D_1__Proteobacteria;D_2__Alphaproteobacteria;D_3__Rhizobiales;D_4__Xanthobacteraceae	Bradyrhizobium
1,36%	3,22%	2,10%	1,51%	0,46%	2,15%	2,13%	1,24%	D_0__Bacteria;D_1__Acidobacteria;D_2__Subgroup 6;Other;Other;Other	
2,17%	1,07%	1,25%	2,49%	0,95%	1,02%	2,68%	0,96%	D_0__Bacteria;D_1__Acidobacteria;D_2__Blastocatellia (Subgroup 4);D_3__Pyrinomonadales;D_4__Pyrinomonadaceae	RB41
1,76%	0,84%	1,55%	0,94%	2,28%	1,10%	0,73%	0,70%	D_0__Bacteria;D_1__Actinobacteria;D_2__Thermoleophilia;D_3__Gaiellales;D_4__uncultured	uncultured bacterium

0,86%	1,07%	1,54%	1,17%	0,31%	2,11%	1,06%	1,54%	D_0__Bacteria;D_1__Verrucomicrobia;D_2__Verrucomicrobiae;D_3__Chthoniobacterales;D_4__Chthoniobacteraceae	Chthoniobacter
1,54%	0,58%	1,32%	1,04%	2,14%	0,85%	0,77%	1,29%	D_0__Bacteria;D_1__Actinobacteria;D_2__Thermoleophilia;D_3__Solirubrobacterales;D_4__67-14	uncultured bacterium
1,08%	1,14%	1,20%	0,70%	0,47%	1,47%	1,44%	1,12%	D_0__Bacteria;D_1__Proteobacteria;D_2__Gammaproteobacteria;D_3__Betaproteobacteriales;D_4__Nitrosomonadaceae	Ellin6067
0,61%	1,67%	1,00%	0,78%	0,28%	0,94%	1,13%	1,10%	D_0__Bacteria;D_1__Proteobacteria;D_2__Gammaproteobacteria;D_3__Betaproteobacteriales;D_4__TRA3-20	uncultured bacterium
1,23%	0,77%	0,96%	0,63%	1,27%	1,01%	0,56%	0,99%	D_0__Bacteria;D_1__Actinobacteria;D_2__Acidimicrobiia;D_3__IMCC26256;D_4__uncultured bacterium	
1,30%	1,20%	0,70%	1,14%	0,28%	1,22%	0,54%	1,01%	D_0__Bacteria;D_1__Proteobacteria;D_2__Gammaproteobacteria;D_3__Betaproteobacteriales;D_4__SC-I-84	uncultured bacterium
0,75%	0,66%	0,74%	0,92%	1,25%	0,85%	1,14%	0,99%	D_0__Bacteria;D_1__Proteobacteria;D_2__Alphaproteobacteria;D_3__Reyranellales;D_4__Reyranellaceae	Reyranella
1,56%	0,47%	0,81%	0,75%	1,28%	0,68%	0,76%	0,92%	D_0__Bacteria;D_1__Chloroflexi;D_2__KD4-96;D_3__uncultured bacterium;D_4__	
0,79%	0,85%	0,74%	0,63%	0,27%	1,57%	0,71%	1,41%	D_0__Bacteria;D_1__Proteobacteria;D_2__Deltaproteobacteria;D_3__Myxococcales;D_4__Haliangiaceae	Haliangium
0,71%	1,29%	1,40%	0,95%	0,14%	0,52%	1,01%	0,62%	D_0__Bacteria;D_1__Bacteroidetes;D_2__Bacteroidia;D_3__Chitinophagales;D_4__Chitinophagaceae	Terrimonas
0,60%	1,08%	0,51%	0,63%	0,37%	1,69%	0,63%	1,01%	D_0__Bacteria;D_1__Verrucomicrobia;D_2__Verrucomicrobiae;D_3__Pedosphaerales;D_4__Pedosphaeraceae	uncultured bacterium
0,18%	0,30%	0,35%	0,45%	3,85%	0,23%	0,53%	0,18%	D_0__Bacteria;D_1__Actinobacteria;D_2__Actinobacteria;D_3__Propionibacteriales;D_4__Nocardioidaceae	Kribbella
0,94%	0,45%	0,28%	0,84%	0,84%	0,87%	0,64%	1,16%	D_0__Bacteria;D_1__Acidobacteria;D_2__Acidobacteriia;D_3__Solibacterales;D_4__Solibacteraceae (Subgroup 3)	Candidatus Solibacter
0,15%	0,12%	0,21%	0,42%	3,56%	0,71%	0,50%	0,35%	D_0__Bacteria;D_1__Actinobacteria;D_2__Actinobacteria;D_3__Streptomycetales;D_4__Streptomycetaceae	Streptomyces
0,62%	0,43%	0,71%	0,97%	0,75%	0,46%	1,01%	0,48%	D_0__Bacteria;D_1__Proteobacteria;D_2__Alphaproteobacteria;D_3__Rhizobiales;D_4__Xanthobacteraceae	Rhodoplanes
0,02%	0,02%	0,05%	0,08%	5,02%	0,02%	0,04%	0,03%	D_0__Bacteria;D_1__Firmicutes;D_2__Bacilli;D_3__Bacillales;D_4__Paenibacillaceae	Paenibacillus
0,85%	0,46%	0,39%	1,07%	0,66%	0,49%	0,55%	0,81%	D_0__Bacteria;D_1__Acidobacteria;D_2__Acidobacteriia;D_3__Solibacterales;D_4__Solibacteraceae (Subgroup 3)	Bryobacter
0,75%	0,54%	0,60%	0,36%	1,08%	0,81%	0,30%	0,59%	D_0__Bacteria;D_1__Actinobacteria;D_2__Thermoleophilia;D_3__Gaiellales;D_4__Gaiellaceae	Gaiella
0,02%	1,20%	0,97%	0,15%	0,17%	1,55%	0,16%	0,77%	D_0__Bacteria;D_1__Bacteroidetes;D_2__Bacteroidia;D_3__Flavobacteriales;D_4__Flavobacteriaceae	Flavobacterium
0,36%	0,29%	0,62%	1,40%	0,64%	0,15%	0,84%	0,28%	D_0__Bacteria;D_1__Firmicutes;D_2__Bacilli;D_3__Bacillales;D_4__Bacillaceae	Bacillus
0,69%	0,29%	0,95%	0,76%	0,21%	0,37%	0,83%	0,43%	D_0__Bacteria;D_1__Proteobacteria;D_2__Alphaproteobacteria;D_3__Sphingomonadales;D_4__Sphingomonadaceae	uncultured
0,05%	0,04%	0,02%	0,04%	4,11%	0,03%	0,12%	0,04%	D_0__Bacteria;D_1__Actinobacteria;D_2__Actinobacteria;D_3__Pseudonocardiales;D_4__Pseudonocardiaceae	Amycolatopsis
0,41%	0,32%	0,32%	0,52%	0,31%	0,89%	0,65%	0,90%	D_0__Bacteria;D_1__Proteobacteria;D_2__Alphaproteobacteria;D_3__Micropepsales;D_4__Micropepsaceae	uncultured
0,43%	0,35%	0,73%	0,48%	0,23%	0,57%	0,77%	0,51%	D_0__Bacteria;D_1__Proteobacteria;D_2__Alphaproteobacteria;D_3__Caulobacterales;D_4__Caulobacteraceae	Phenylobacterium
0,17%	0,53%	0,98%	0,31%	0,10%	0,86%	0,45%	0,63%	D_0__Bacteria;D_1__Bacteroidetes;D_2__Bacteroidia;D_3__Chitinophagales;D_4__Chitinophagaceae	Ferruginibacter
0,25%	0,69%	0,63%	0,59%	0,18%	0,49%	0,68%	0,44%	D_0__Bacteria;D_1__Proteobacteria;D_2__Gammaproteobacteria;D_3__Gammaproteobacteria Incertae Sedis;D_4__Unknown Family	Acidibacter
0,03%	0,05%	0,21%	1,61%	0,10%	0,52%	1,26%	0,09%	D_0__Bacteria;D_1__Bacteroidetes;D_2__Bacteroidia;D_3__Sphingobacteriales;D_4__Sphingobacteriaceae	Mucilaginibacter
0,65%	0,16%	0,25%	0,60%	0,41%	0,47%	0,54%	0,78%	D_0__Bacteria;D_1__Gemmatimonadetes;D_2__Gemmatimonadetes;D_3__Gemmatimonadales;D_4__Gemmatimonadaceae	Gemmatimonas
0,56%	0,34%	0,63%	0,38%	0,21%	0,32%	0,41%	0,50%	D_0__Bacteria;D_1__Actinobacteria;D_2__Acidimicrobiia;D_3__Microtrichales;D_4__uncultured	uncultured bacterium
0,40%	0,17%	0,51%	0,35%	0,92%	0,29%	0,38%	0,31%	D_0__Bacteria;D_1__Actinobacteria;D_2__Thermoleophilia;D_3__Solirubrobacterales;D_4__Solirubrobacteraceae	Solirubrobacter
0,28%	0,49%	0,38%	0,36%	0,12%	0,42%	0,70%	0,55%	D_0__Bacteria;D_1__Proteobacteria;D_2__Gammaproteobacteria;D_3__Betaproteobacteriales;D_4__Nitrosomonadaceae	IS-44
0,40%	0,26%	0,34%	0,40%	0,26%	0,30%	0,70%	0,39%	D_0__Bacteria;D_1__Proteobacteria;D_2__Alphaproteobacteria;D_3__Dongiales;D_4__Dongiaceae	Dongia
0,11%	0,65%	0,81%	0,26%	0,05%	0,57%	0,35%	0,16%	D_0__Bacteria;D_1__Acidobacteria;D_2__Blastocatellia (Subgroup 4);D_3__Blastocatellales;D_4__Blastocatellaceae	uncultured
0,50%	0,23%	0,36%	0,30%	0,27%	0,24%	0,61%	0,38%	D_0__Bacteria;D_1__Patescibacteria;D_2__Saccharimonadia;D_3__Saccharimonadales;D_4__uncultured bacterium	
0,28%	0,08%	0,25%	0,41%	0,23%	0,22%	0,99%	0,20%	D_0__Bacteria;D_1__Proteobacteria;D_2__Alphaproteobacteria;D_3__Rhizobiales;D_4__Rhizobiaceae	Mesorhizobium
0,27%	0,11%	0,26%	0,19%	0,94%	0,21%	0,26%	0,40%	D_0__Bacteria;D_1__Actinobacteria;D_2__Actinobacteria;D_3__Corynebacteriales;D_4__Mycobacteriaceae	Mycobacterium
0,31%	0,54%	0,46%	0,39%	0,14%	0,23%	0,43%	0,14%	D_0__Bacteria;D_1__Proteobacteria;D_2__Deltaproteobacteria;D_3__Myxococcales;D_4__bacteriap25	uncultured bacterium
0,18%	0,50%	0,43%	0,42%	0,09%	0,25%	0,38%	0,33%	D_0__Bacteria;D_1__Proteobacteria;D_2__Gammaproteobacteria;D_3__Steroidobacterales;D_4__Steroidobacteraceae	uncultured
0,51%	0,26%	0,28%	0,37%	0,17%	0,36%	0,24%	0,39%	D_0__Bacteria;D_1__Actinobacteria;D_2__Acidimicrobiia;D_3__Microtrichales;D_4__Ilumatobacteraceae	CL500-29 marine group
0,21%	0,55%	0,65%	0,23%	0,35%	0,27%	0,18%	0,09%	D_0__Bacteria;D_1__Actinobacteria;D_2__MB-A2-108;D_3__uncultured bacterium;D_4__	
0,27%	0,45%	0,41%	0,21%	0,15%	0,48%	0,26%	0,27%	D_0__Bacteria;D_1__Proteobacteria;D_2__Gammaproteobacteria;D_3__Betaproteobacteriales;D_4__Burkholderiaceae	Rhizobacter
0,04%	1,36%	0,37%	0,05%	0,01%	0,38%	0,08%	0,19%	D_0__Bacteria;D_1__Bacteroidetes;D_2__Bacteroidia;D_3__Chitinophagales;D_4__Saprospiraceae	uncultured
0,30%	0,33%	0,48%	0,50%	0,21%	0,18%	0,36%	0,09%	D_0__Bacteria;D_1__Proteobacteria;D_2__Alphaproteobacteria;D_3__Rhizobiales;D_4__Hyphomicrobiaceae	Pedomicrobium
0,25%	0,32%	0,32%	0,38%	0,12%	0,23%	0,51%	0,26%	D_0__Bacteria;D_1__Proteobacteria;D_2__Alphaproteobacteria;D_3__Rhizobiales;D_4__KF-JG30-B3	uncultured bacterium
0,19%	0,19%	0,29%	0,23%	0,26%	0,35%	0,24%	0,53%	D_0__Bacteria;D_1__Actinobacteria;D_2__Actinobacteria;D_3__Propionibacteriales;D_4__Nocardioidaceae	Nocardioides
0,06%	0,53%	0,78%	0,15%	0,23%	0,15%	0,31%	0,08%	D_0__Bacteria;D_1__Proteobacteria;D_2__Gammaproteobacteria;D_3__Betaproteobacteriales;D_4__Burkholderiaceae	uncultured
0,41%	0,56%	0,40%	0,25%	0,27%	0,12%	0,25%	0,04%	D_0__Bacteria;D_1__Rokubacteria;D_2__NC10;D_3__Rokubacteriales;D_4__uncultured bacterium	
0,02%	0,53%	0,12%	1,42%	0,02%	0,03%	0,01%	0,00%	D_0__Archaea;D_1__Thaumarchaeota;D_2__Nitrososphaeria;D_3__Nitrososphaerales;D_4__Nitrososphaeraceae;Other	
0,18%	0,37%	0,19%	0,23%	0,31%	0,39%	0,18%	0,16%	D_0__Bacteria;D_1__Proteobacteria;D_2__Alphaproteobacteria;D_3__Elsterales;D_4__uncultured;Other	
0,08%	0,07%	0,10%	0,09%	1,17%	0,11%	0,12%	0,12%	D_0__Bacteria;D_1__Proteobacteria;D_2__Gammaproteobacteria;D_3__Betaproteobacteriales;D_4__Burkholderiaceae	Burkholderia-Caballeronia-Paraburkholderia
0,10%	0,27%	0,48%	0,14%	0,06%	0,31%	0,18%	0,34%	D_0__Bacteria;D_1__Actinobacteria;D_2__Acidimicrobiia;D_3__Microtrichales;D_4__Iamiaceae	Iamia
0,16%	0,21%	0,46%	0,19%	0,11%	0,27%	0,22%	0,23%	D_0__Bacteria;D_1__Actinobacteria;D_2__Acidimicrobiia;D_3__Microtrichales;D_4__Ilumatobacteraceae	uncultured
0,13%	0,27%	0,15%	0,14%	0,10%	0,56%	0,18%	0,31%	D_0__Bacteria;D_1__Verrucomicrobia;D_2__Verrucomicrobiae;D_3__Pedosphaerales;D_4__Pedosphaeraceae;Other	
0,38%	0,15%	0,27%	0,14%	0,40%	0,14%	0,13%	0,19%	D_0__Bacteria;D_1__Actinobacteria;D_2__Actinobacteria;D_3__Micromonosporales;D_4__Micromonosporaceae	Luedemannella
0,07%	0,14%	0,22%	0,16%	0,05%	0,76%	0,09%	0,29%	D_0__Bacteria;D_1__Bacteroidetes;D_2__Bacteroidia;D_3__Sphingobacteriales;D_4__env,OPS 17	uncultured bacterium
0,34%	0,21%	0,10%	0,22%	0,40%	0,16%	0,16%	0,18%	D_0__Bacteria;D_1__Proteobacteria;D_2__Alphaproteobacteria;D_3__Elsterales;D_4__uncultured	uncultured bacterium
0,12%	0,29%	0,44%	0,12%	0,10%	0,35%	0,16%	0,18%	D_0__Bacteria;D_1__Proteobacteria;D_2__Gammaproteobacteria;D_3__Xanthomonadales;D_4__Xanthomonadaceae	Arenimonas
0,69%	0,02%	0,01%	0,17%	0,49%	0,12%	0,02%	0,21%	D_0__Bacteria;D_1__Acidobacteria;D_2__Acidobacteriia;D_3__Acidobacteriales;D_4__uncultured	uncultured bacterium
0,13%	0,37%	0,24%	0,11%	0,06%	0,25%	0,29%	0,27%	D_0__Bacteria;D_1__Acidobacteria;D_2__Blastocatellia (Subgroup 4);D_3__Blastocatellales;D_4__Blastocatellaceae	JGI 0001001-H03
0,07%	0,49%	0,30%	0,16%	0,04%	0,25%	0,25%	0,13%	D_0__Bacteria;D_1__Proteobacteria;D_2__Gammaproteobacteria;D_3__CCD24;D_4__uncultured bacterium	
0,05%	0,29%	0,17%	0,06%	0,05%	0,53%	0,16%	0,34%	D_0__Bacteria;D_1__Proteobacteria;D_2__Deltaproteobacteria;D_3__Myxococcales;D_4__BIrii41	uncultured bacterium
0,08%	0,16%	0,20%	0,06%	0,12%	0,57%	0,09%	0,37%	D_0__Bacteria;D_1__Actinobacteria;D_2__Acidimicrobiia;D_3__Microtrichales;D_4__Ilumatobacteraceae	Ilumatobacter
0,25%	0,36%	0,13%	0,27%	0,06%	0,27%	0,13%	0,17%	D_0__Bacteria;D_1__Proteobacteria;D_2__Gammaproteobacteria;D_3__Betaproteobacteriales;D_4__SC-I-84;Other	
0,17%	0,37%	0,21%	0,27%	0,05%	0,31%	0,11%	0,14%	D_0__Bacteria;D_1__Bacteroidetes;D_2__Bacteroidia;D_3__Chitinophagales;D_4__Chitinophagaceae	Flavitalea
0,19%	0,18%	0,22%	0,19%	0,14%	0,28%	0,13%	0,29%	D_0__Bacteria;D_1__Actinobacteria;D_2__Acidimicrobiia;D_3__IMCC26256;Other;Other	
0,29%	0,09%	0,21%	0,18%	0,06%	0,07%	0,54%	0,17%	D_0__Bacteria;D_1__Proteobacteria;D_2__Alphaproteobacteria;D_3__Rhizobiales;D_4__Rhizobiales Incertae Sedis	uncultured
0,23%	0,12%	0,18%	0,21%	0,18%	0,11%	0,21%	0,35%	D_0__Bacteria;D_1__Acidobacteria;D_2__Holophagae;D_3__Subgroup 7;D_4__uncultured bacterium	
0,18%	0,22%	0,11%	0,12%	0,10%	0,35%	0,11%	0,30%	D_0__Bacteria;D_1__Proteobacteria;D_2__Deltaproteobacteria;D_3__Myxococcales;D_4__Phaselicystidaceae	Phaselicystis
0,07%	0,05%	0,11%	0,14%	0,68%	0,12%	0,17%	0,11%	D_0__Bacteria;D_1__Proteobacteria;D_2__Gammaproteobacteria;D_3__Betaproteobacteriales;D_4__Burkholderiaceae	Massilia
0,10%	0,15%	0,11%	0,10%	0,19%	0,31%	0,35%	0,13%	D_0__Bacteria;D_1__Proteobacteria;D_2__Gammaproteobacteria;D_3__Pseudomonadales;D_4__Pseudomonadaceae	Pseudomonas
0,16%	0,09%	0,09%	0,35%	0,11%	0,19%	0,21%	0,23%	D_0__Bacteria;D_1__Proteobacteria;D_2__Alphaproteobacteria;D_3__Acetobacterales;D_4__Acetobacteraceae	uncultured
0,12%	0,18%	0,27%	0,12%	0,18%	0,21%	0,18%	0,13%	D_0__Bacteria;D_1__Proteobacteria;D_2__Gammaproteobacteria;D_3__Betaproteobacteriales;D_4__Burkholderiaceae;Other	
0,46%	0,06%	0,06%	0,18%	0,22%	0,14%	0,14%	0,12%	D_0__Bacteria;D_1__Proteobacteria;D_2__Alphaproteobacteria;D_3__Rhizobiales;D_4__Rhizobiales Incertae Sedis	Nordella

0,26%	0,18%	0,24%	0,15%	0,11%	0,08%	0,26%	0,10%	D_0__Bacteria;D_1__Acidobacteria;D_2__Subgroup 17;D_3__uncultured bacterium;D_4__	
0,13%	0,07%	0,37%	0,25%	0,32%	0,03%	0,19%	0,01%	D_0__Bacteria;D_1__Actinobacteria;D_2__Rubrobacteria;D_3__Rubrobacterales;D_4__Rubrobacteriaceae	Rubrobacter
0,22%	0,13%	0,12%	0,21%	0,07%	0,14%	0,22%	0,21%	D_0__Bacteria;D_1__Proteobacteria;D_2__Alphaproteobacteria;D_3__Rhizobiales;D_4__uncultured	uncultured bacterium
0,02%	0,43%	0,19%	0,01%	0,01%	0,33%	0,04%	0,26%	D_0__Bacteria;D_1__Bacteroidetes;D_2__Bacteroidia;D_3__Cytophagales;D_4__Hymenobacteraceae	Adhaeribacter
0,26%	0,08%	0,10%	0,08%	0,36%	0,17%	0,05%	0,13%	D_0__Bacteria;D_1__Actinobacteria;D_2__Thermoleophilia;D_3__Gaiellales;D_4__uncultured;Other	
0,28%	0,04%	0,07%	0,09%	0,37%	0,11%	0,07%	0,15%	D_0__Bacteria;D_1__Actinobacteria;D_2__Thermoleophilia;D_3__Solirubrobacterales;D_4__Solirubrobacteraceae	Conexibacter
0,08%	0,04%	0,11%	0,14%	0,09%	0,14%	0,53%	0,06%	D_0__Bacteria;D_1__Proteobacteria;D_2__Alphaproteobacteria;D_3__Rhizobiales;D_4__Rhizobiaceae	Allorhizobium-Neorhizobium-Pararhizobium-Rhizobium
0,17%	0,14%	0,12%	0,16%	0,10%	0,13%	0,20%	0,10%	D_0__Bacteria;D_1__Acidobacteria;D_2__Blastocatellia (Subgroup 4);D_3__Elev-16S-573;D_4__uncultured bacterium	
0,10%	0,16%	0,02%	0,06%	0,04%	0,45%	0,05%	0,26%	D_0__Bacteria;D_1__Proteobacteria;D_2__Gammaproteobacteria;D_3__Xanthomonadales;D_4__Rhodanobacteracea e	Rhodanobacter
0,10%	0,19%	0,11%	0,14%	0,04%	0,14%	0,18%	0,23%	D_0__Bacteria;D_1__Proteobacteria;D_2__Gammaproteobacteria;D_3__Betaproteobacteriales;D_4__TRA3-20	metagenome
0,07%	0,25%	0,04%	0,08%	0,05%	0,35%	0,03%	0,24%	D_0__Bacteria;D_1__Acidobacteria;D_2__Subgroup 5;D_3__uncultured Acidobacteria bacterium;D_4__	
0,04%	0,25%	0,17%	0,06%	0,01%	0,32%	0,06%	0,20%	D_0__Bacteria;D_1__Bacteroidetes;D_2__Bacteroidia;D_3__Sphingobacteriales;D_4__AKYH767	uncultured bacterium
0,14%	0,05%	0,02%	0,14%	0,13%	0,08%	0,05%	0,48%	D_0__Bacteria;D_1__Acidobacteria;D_2__Acidobacteriia;D_3__Subgroup 2;D_4__uncultured bacterium	
0,10%	0,06%	0,09%	0,09%	0,42%	0,06%	0,13%	0,12%	D_0__Bacteria;D_1__Actinobacteria;D_2__Actinobacteria;D_3__Pseudonocardiales;D_4__Pseudonocardiaceae	Pseudonocardia
0,11%	0,26%	0,17%	0,09%	0,04%	0,22%	0,12%	0,05%	D_0__Bacteria;D_1__Planctomycetes;D_2__OM190;D_3__uncultured bacterium;D_4__	
0,08%	0,16%	0,25%	0,07%	0,02%	0,15%	0,21%	0,14%	D_0__Bacteria;D_1__Acidobacteria;D_2__Blastocatellia (Subgroup 4);D_3__Blastocatellales;D_4__Blastocatellaceae	Stenotrophobacter
0,00%	0,07%	0,69%	0,02%	0,01%	0,08%	0,06%	0,11%	D_0__Bacteria;D_1__Proteobacteria;D_2__Gammaproteobacteria;D_3__Cellvibrionales;D_4__Halieaceae	OM60(NOR5) clade
0,05%	0,15%	0,20%	0,07%	0,03%	0,20%	0,14%	0,21%	D_0__Bacteria;D_1__Proteobacteria;D_2__Deltaproteobacteria;D_3__Myxococcales;D_4__Sandaracinaceae	uncultured
0,10%	0,06%	0,17%	0,14%	0,08%	0,07%	0,30%	0,11%	D_0__Bacteria;D_1__Proteobacteria;D_2__Alphaproteobacteria;D_3__Rhizobiales;D_4__Devosiaceae	Devosia
0,15%	0,16%	0,15%	0,15%	0,13%	0,14%	0,11%	0,04%	D_0__Bacteria;D_1__Chloroflexi;D_2__TK10;D_3__uncultured bacterium;D_4__	
0,05%	0,14%	0,24%	0,14%	0,04%	0,10%	0,19%	0,11%	D_0__Bacteria;D_1__Proteobacteria;D_2__Gammaproteobacteria;D_3__Steroidobacterales;D_4__Steroidobacteracea e	Steroidobacter
0,09%	0,22%	0,22%	0,12%	0,06%	0,14%	0,10%	0,06%	D_0__Bacteria;D_1__Chloroflexi;D_2__Dehalococcoidia;D_3__S085;D_4__uncultured bacterium	
0,07%	0,09%	0,18%	0,12%	0,05%	0,09%	0,25%	0,14%	D_0__Bacteria;D_1__Proteobacteria;D_2__Alphaproteobacteria;D_3__Caulobacterales;D_4__Caulobacteraceae	uncultured
0,08%	0,22%	0,15%	0,15%	0,06%	0,21%	0,05%	0,06%	D_0__Bacteria;D_1__Acidobacteria;D_2__Thermoanaerobaculia;D_3__Thermoanaerobaculales;D_4__Thermoanaerob aculaceae	Subgroup 10
0,11%	0,13%	0,11%	0,23%	0,05%	0,07%	0,22%	0,06%	D_0__Bacteria;D_1__Proteobacteria;D_2__Deltaproteobacteria;D_3__Myxococcales;D_4__bacteriap25;Other	
0,39%	0,01%	0,01%	0,17%	0,18%	0,03%	0,03%	0,16%	D_0__Bacteria;D_1__Acidobacteria;D_2__Acidobacteriia;D_3__Acidobacteriales;D_4__uncultured	uncultured Acidobacteria bacterium
0,03%	0,18%	0,30%	0,05%	0,01%	0,11%	0,16%	0,14%	D_0__Bacteria;D_1__Acidobacteria;D_2__Acidobacteriia;D_3__Solibacterales;D_4__Solibacteraceae (Subgroup 3)	Paludibaculum
0,06%	0,07%	0,48%	0,05%	0,05%	0,08%	0,09%	0,07%	D_0__Bacteria;D_1__Proteobacteria;D_2__Alphaproteobacteria;D_3__Sphingomonadales;D_4__Sphingomonadaceae	Novosphingobium
0,16%	0,15%	0,11%	0,09%	0,04%	0,16%	0,13%	0,10%	D_0__Bacteria;D_1__Proteobacteria;D_2__Deltaproteobacteria;D_3__Desulfarculales;D_4__Desulfarculaceae	uncultured
0,07%	0,16%	0,12%	0,08%	0,05%	0,12%	0,18%	0,15%	D_0__Bacteria;D_1__Proteobacteria;D_2__Gammaproteobacteria;D_3__Betaproteobacteriales;D_4__TRA3-20;Other	
0,08%	0,43%	0,09%	0,18%	0,04%	0,01%	0,07%	0,03%	D_0__Bacteria;D_1__Nitrospirae;D_2__Nitrospira;D_3__Nitrospirales;D_4__Nitrospiraceae	Nitrospira
0,13%	0,03%	0,02%	0,09%	0,19%	0,24%	0,08%	0,13%	D_0__Bacteria;D_1__Actinobacteria;D_2__Actinobacteria;D_3__Frankiales;D_4__Acidothermaceae	Acidothermus
0,05%	0,22%	0,25%	0,12%	0,01%	0,04%	0,16%	0,06%	D_0__Bacteria;D_1__Proteobacteria;D_2__Gammaproteobacteria;D_3__PLTA13;D_4__uncultured bacterium	
0,02%	0,19%	0,33%	0,03%	0,02%	0,12%	0,09%	0,12%	D_0__Bacteria;D_1__Bacteroidetes;D_2__Bacteroidia;D_3__Cytophagales;D_4__Microscillaceae	Ohtaekwangia
0,05%	0,02%	0,03%	0,35%	0,05%	0,17%	0,09%	0,14%	D_0__Bacteria;D_1__Acidobacteria;D_2__Acidobacteriia;D_3__Acidobacteriales;D_4__Acidobacteriaceae (Subgroup 1)	Granulicella
0,07%	0,09%	0,18%	0,12%	0,03%	0,06%	0,29%	0,06%	D_0__Bacteria;D_1__Verrucomicrobia;D_2__Verrucomicrobiae;D_3__Chthoniobacterales;D_4__Xiphinematobacterace ae	Candidatus Xiphinematobacter
0,17%	0,29%	0,05%	0,11%	0,04%	0,08%	0,08%	0,06%	D_0__Bacteria;D_1__Latescibacteria;D_2__uncultured bacterium;D_3__;D_4__	
0,12%	0,07%	0,11%	0,11%	0,17%	0,13%	0,06%	0,10%	D_0__Bacteria;D_1__Planctomycetes;D_2__Planctomycetacia;D_3__Gemmatales;D_4__Gemmataceae	uncultured
0,06%	0,09%	0,09%	0,08%	0,05%	0,21%	0,10%	0,18%	D_0__Bacteria;D_1__Verrucomicrobia;D_2__Verrucomicrobiae;D_3__Pedosphaerales;D_4__Pedosphaeraceae	metagenome
0,16%	0,04%	0,04%	0,14%	0,19%	0,11%	0,07%	0,11%	D_0__Bacteria;D_1__Actinobacteria;D_2__Actinobacteria;D_3__Frankiales;D_4__Frankiaceae	Jatrophihabitans
0,09%	0,05%	0,07%	0,14%	0,19%	0,08%	0,12%	0,12%	D_0__Bacteria;D_1__Proteobacteria;D_2__Alphaproteobacteria;D_3__Rhizobiales;D_4__Xanthobacteraceae;Other	
0,01%	0,02%	0,01%	0,06%	0,68%	0,02%	0,02%	0,03%	D_0__Bacteria;D_1__Actinobacteria;D_2__Actinobacteria;D_3__Streptosporangiales;D_4__Streptosporangiaceae	Streptosporangium
0,09%	0,05%	0,08%	0,05%	0,23%	0,18%	0,10%	0,05%	D_0__Bacteria;D_1__Actinobacteria;D_2__Actinobacteria;D_3__Micromonosporales;D_4__Micromonosporaceae;Other	
0,06%	0,03%	0,08%	0,10%	0,33%	0,05%	0,16%	0,04%	D_0__Bacteria;D_1__Actinobacteria;D_2__Actinobacteria;D_3__Micromonosporales;D_4__Micromonosporaceae	Dactylosporangium
0,50%	0,01%	0,00%	0,06%	0,18%	0,04%	0,01%	0,03%	D_0__Bacteria;D_1__Proteobacteria;D_2__Deltaproteobacteria;D_3__Myxococcales;D_4__Archangiaceae	Anaeromyxobacter
0,11%	0,09%	0,09%	0,10%	0,09%	0,09%	0,14%	0,11%	D_0__Bacteria;D_1__Proteobacteria;D_2__Alphaproteobacteria;D_3__uncultured;D_4__uncultured bacterium	
0,02%	0,01%	0,08%	0,35%	0,12%	0,06%	0,10%	0,08%	D_0__Bacteria;D_1__Acidobacteria;D_2__Acidobacteriia;D_3__Acidobacteriales;D_4__Acidobacteriaceae (Subgroup 1)	Edaphobacter
0,01%	0,18%	0,13%	0,01%	0,00%	0,16%	0,02%	0,29%	D_0__Bacteria;D_1__Bacteroidetes;D_2__Bacteroidia;D_3__Chitinophagales;D_4__Chitinophagaceae	Dinghuibacter
0,34%	0,01%	0,00%	0,05%	0,28%	0,06%	0,01%	0,06%	D_0__Bacteria;D_1__Acidobacteria;D_2__Acidobacteriia;D_3__Acidobacteriales;D_4__uncultured;Other	
0,14%	0,03%	0,11%	0,06%	0,19%	0,07%	0,15%	0,05%	D_0__Bacteria;D_1__Actinobacteria;D_2__Actinobacteria;D_3__Micromonosporales;D_4__Micromonosporaceae	Actinoplanes
0,18%	0,05%	0,06%	0,13%	0,09%	0,06%	0,12%	0,09%	D_0__Bacteria;D_1__Proteobacteria;D_2__Alphaproteobacteria;D_3__Rhizobiales;D_4__Rhizobiales Incertae Sedis	Bauldia
0,01%	0,00%	0,04%	0,01%	0,02%	0,01%	0,66%	0,01%	D_0__Bacteria;D_1__Proteobacteria;D_2__Gammaproteobacteria;D_3__Xanthomonadales;D_4__Rhodanobacteracea e	Luteibacter
0,00%	0,01%	0,00%	0,00%	0,72%	0,01%	0,01%	0,00%	D_0__Bacteria;D_1__Firmicutes;D_2__Bacilli;D_3__Bacillales;D_4__Paenibacillaceae	Cohnella
0,20%	0,04%	0,09%	0,08%	0,08%	0,07%	0,07%	0,12%	D_0__Bacteria;D_1__Actinobacteria;D_2__Acidimicrobiia;D_3__uncultured;D_4__uncultured bacterium	
0,08%	0,07%	0,07%	0,03%	0,06%	0,16%	0,11%	0,16%	D_0__Bacteria;D_1__Proteobacteria;D_2__Deltaproteobacteria;D_3__Myxococcales;D_4__BIrii41	metagenome
0,11%	0,13%	0,11%	0,07%	0,05%	0,13%	0,05%	0,09%	D_0__Bacteria;D_1__Planctomycetes;D_2__Phycisphaerae;D_3__Phycisphaerales;D_4__Phycisphaeraceae	SM1A02
0,10%	0,06%	0,22%	0,06%	0,06%	0,04%	0,09%	0,11%	D_0__Bacteria;D_1__Actinobacteria;D_2__Acidimicrobiia;D_3__uncultured;Other;Other	
0,03%	0,07%	0,36%	0,03%	0,01%	0,10%	0,04%	0,09%	D_0__Bacteria;D_1__Verrucomicrobia;D_2__Verrucomicrobiae;D_3__Verrucomicrobiales;D_4__Rubritaleaceae	Luteolibacter
0,17%	0,03%	0,05%	0,06%	0,24%	0,06%	0,08%	0,01%	D_0__Bacteria;D_1__Actinobacteria;D_2__Actinobacteria;D_3__Micromonosporales;D_4__Micromonosporaceae	Micromonospora
0,09%	0,09%	0,14%	0,08%	0,05%	0,03%	0,13%	0,08%	D_0__Bacteria;D_1__Proteobacteria;D_2__Alphaproteobacteria;D_3__Sphingomonadales;D_4__Sphingomonadaceae	Ellin6055
0,05%	0,10%	0,12%	0,09%	0,05%	0,11%	0,09%	0,07%	D_0__Bacteria;D_1__Proteobacteria;D_2__Deltaproteobacteria;D_3__Bdellovibrionales;D_4__Bdellovibrionaceae	Bdellovibrio
0,04%	0,11%	0,11%	0,03%	0,04%	0,14%	0,09%	0,11%	D_0__Bacteria;D_1__Proteobacteria;D_2__Gammaproteobacteria;D_3__Betaproteobacteriales;D_4__Burkholderiacea e	Ramlibacter
0,11%	0,05%	0,20%	0,11%	0,03%	0,08%	0,06%	0,05%	D_0__Bacteria;D_1__Bacteroidetes;D_2__Bacteroidia;D_3__Chitinophagales;D_4__Chitinophagaceae	Flavisolibacter
0,08%	0,13%	0,11%	0,08%	0,02%	0,09%	0,10%	0,07%	D_0__Bacteria;D_1__Acidobacteria;D_2__Subgroup 6;D_3__uncultured Acidobacteriales bacterium;D_4__	
0,16%	0,04%	0,10%	0,16%	0,09%	0,02%	0,04%	0,06%	D_0__Bacteria;D_1__Planctomycetes;D_2__Phycisphaerae;D_3__Tepidisphaerales;D_4__WD2101 soil group;Other	
0,05%	0,10%	0,09%	0,07%	0,09%	0,06%	0,11%	0,10%	D_0__Bacteria;D_1__Proteobacteria;D_2__Alphaproteobacteria;D_3__Rhizobiales;D_4__Xanthobacteraceae	Pseudolabrys
0,11%	0,03%	0,12%	0,06%	0,13%	0,08%	0,06%	0,06%	D_0__Bacteria;D_1__Chloroflexi;D_2__Chloroflexia;D_3__Thermomicrobiales;D_4__JG30-KF-CM45	uncultured bacterium
0,05%	0,03%	0,07%	0,06%	0,11%	0,11%	0,06%	0,18%	D_0__Bacteria;D_1__Actinobacteria;D_2__Actinobacteria;D_3__Frankiales;D_4__Sporichthyaceae	uncultured
0,07%	0,04%	0,07%	0,07%	0,15%	0,08%	0,12%	0,04%	D_0__Bacteria;D_1__Actinobacteria;D_2__Actinobacteria;D_3__Micromonosporales;D_4__Micromonosporaceae	uncultured
0,07%	0,07%	0,10%	0,06%	0,07%	0,06%	0,14%	0,08%	D_0__Bacteria;D_1__Proteobacteria;D_2__Gammaproteobacteria;D_3__Xanthomonadales;D_4__Rhodanobacteracea e	Dokdonella
0,09%	0,08%	0,07%	0,04%	0,07%	0,15%	0,08%	0,07%	D_0__Bacteria;D_1__Proteobacteria;D_2__Deltaproteobacteria;D_3__Myxococcales;D_4__Polyangiaceae	Pajaroellobacter
0,04%	0,15%	0,09%	0,03%	0,01%	0,17%	0,03%	0,11%	D_0__Bacteria;D_1__Bacteroidetes;D_2__Bacteroidia;D_3__Chitinophagales;D_4__Chitinophagaceae	Parafilimonas
0,09%	0,01%	0,01%	0,18%	0,22%	0,02%	0,03%	0,04%	D_0__Bacteria;D_1__Proteobacteria;D_2__Gammaproteobacteria;D_3__Betaproteobacteriales;D_4__Nitrosomonadac eae	GOUTA6
0,05%	0,06%	0,05%	0,08%	0,03%	0,16%	0,06%	0,10%	D_0__Bacteria;D_1__Verrucomicrobia;D_2__Verrucomicrobiae;D_3__Pedosphaerales;D_4__Pedosphaeraceae	uncultured Verrucomicrobia bacterium
0,07%	0,10%	0,08%	0,09%	0,07%	0,06%	0,09%	0,06%	D_0__Bacteria;D_1__Proteobacteria;D_2__Alphaproteobacteria;D_3__Rhizobiales;D_4__Methyloligellaceae	uncultured
0,08%	0,07%	0,06%	0,09%	0,04%	0,10%	0,08%	0,07%	D_0__Bacteria;D_1__Proteobacteria;D_2__Gammaproteobacteria;D_3__Diplorickettsiales;D_4__Diplorickettsiaceae	uncultured
0,08%	0,08%	0,06%	0,07%	0,08%	0,07%	0,08%	0,07%	D_0__Bacteria;D_1__Bacteroidetes;D_2__Bacteroidia;D_3__Bacteroidales;D_4__Prevotellaceae	Prevotella 7

0,15%	0,05%	0,03%	0,02%	0,20%	0,05%	0,02%	0,06%	D_0__Bacteria;D_1__Proteobacteria;D_2__Alphaproteobacteria;D_3__Elsterales;D_4__uncultured	uncultured alpha proteobacterium
0,02%	0,11%	0,15%	0,04%	0,02%	0,08%	0,11%	0,04%	D_0__Bacteria;D_1__Bacteroidetes;D_2__Bacteroidia;D_3__Cytophagales;D_4__Microscillaceae	Chryseolinea
0,05%	0,07%	0,09%	0,07%	0,06%	0,05%	0,09%	0,09%	D_0__Bacteria;D_1__Proteobacteria;D_2__Alphaproteobacteria;D_3__Rhizobiales;D_4__Hyphomicrobiaceae	Hyphomicrobium
0,09%	0,04%	0,12%	0,06%	0,13%	0,04%	0,04%	0,05%	D_0__Bacteria;D_1__Actinobacteria;D_2__Thermoleophilia;D_3__Solirubrobacterales;D_4__67-14;Other	
0,03%	0,02%	0,08%	0,18%	0,03%	0,03%	0,17%	0,01%	D_0__Bacteria;D_1__Proteobacteria;D_2__Alphaproteobacteria;D_3__Rhizobiales;D_4__Rhizobiaceae	Phyllobacterium
0,03%	0,08%	0,10%	0,05%	0,04%	0,03%	0,15%	0,08%	D_0__Bacteria;D_1__Proteobacteria;D_2__Alphaproteobacteria;D_3__Caulobacterales;D_4__Hyphomonadaceae	Hirschia
0,04%	0,05%	0,10%	0,04%	0,03%	0,13%	0,09%	0,07%	D_0__Bacteria;D_1__Verrucomicrobia;D_2__Verrucomicrobiae;D_3__Opitutales;D_4__Opitutaceae	Opitutus
0,04%	0,10%	0,14%	0,05%	0,02%	0,05%	0,04%	0,09%	D_0__Bacteria;D_1__Proteobacteria;D_2__Gammaproteobacteria;D_3__Xanthomonadales;D_4__Xanthomonadaceae	Lysobacter
0,06%	0,01%	0,01%	0,03%	0,29%	0,01%	0,03%	0,11%	D_0__Bacteria;D_1__Actinobacteria;D_2__Actinobacteria;D_3__Streptosporangiales;D_4__Thermomonosporaceae	Actinoallomurus
0,04%	0,04%	0,06%	0,08%	0,04%	0,07%	0,11%	0,11%	D_0__Bacteria;D_1__Proteobacteria;D_2__Alphaproteobacteria;D_3__Rhodospirillales;D_4__Rhodospirillaceae	uncultured
0,22%	0,01%	0,02%	0,11%	0,08%	0,02%	0,05%	0,03%	D_0__Bacteria;D_1__Chloroflexi;D_2__Chloroflexia;D_3__Chloroflexales;D_4__Roseiflexaceae	uncultured
0,03%	0,13%	0,14%	0,04%	0,01%	0,07%	0,04%	0,08%	D_0__Bacteria;D_1__Verrucomicrobia;D_2__Verrucomicrobiae;D_3__Verrucomicrobiales;D_4__Verrucomicrobiaceae	uncultured
0,09%	0,03%	0,03%	0,09%	0,06%	0,06%	0,09%	0,07%	D_0__Bacteria;D_1__Proteobacteria;D_2__Alphaproteobacteria;D_3__uncultured;Other;Other	
0,09%	0,07%	0,07%	0,07%	0,03%	0,04%	0,06%	0,05%	D_0__Bacteria;D_1__Acidobacteria;D_2__Subgroup 17;D_3__uncultured Acidobacteria bacterium;D_4__	
0,04%	0,05%	0,05%	0,06%	0,01%	0,05%	0,14%	0,09%	D_0__Bacteria;D_1__Proteobacteria;D_2__Alphaproteobacteria;D_3__Caulobacterales;D_4__Hyphomonadaceae	SWB02
0,07%	0,05%	0,01%	0,04%	0,07%	0,12%	0,03%	0,09%	D_0__Bacteria;D_1__Verrucomicrobia;D_2__Verrucomicrobiae;D_3__Pedosphaerales;D_4__Pedosphaeraceae	ADurb,Bin063-1
0,07%	0,07%	0,05%	0,04%	0,04%	0,09%	0,07%	0,06%	D_0__Bacteria;D_1__Acidobacteria;D_2__Subgroup 17;Other;Other;Other	
0,06%	0,05%	0,03%	0,03%	0,04%	0,12%	0,05%	0,12%	D_0__Bacteria;D_1__Proteobacteria;D_2__Alphaproteobacteria;D_3__Rhizobiales;D_4__KF-JG30-B3;Other	
0,03%	0,03%	0,07%	0,06%	0,01%	0,10%	0,04%	0,14%	D_0__Bacteria;D_1__Bacteroidetes;D_2__Bacteroidia;D_3__Sphingobacteriales;D_4__env,OPS 17;Other	
0,03%	0,08%	0,09%	0,06%	0,03%	0,04%	0,06%	0,08%	D_0__Bacteria;D_1__Proteobacteria;D_2__Gammaproteobacteria;D_3__Betaproteobacteriales;D_4__Burkholderiaceae	Variovorax
0,06%	0,07%	0,07%	0,04%	0,02%	0,05%	0,08%	0,07%	D_0__Bacteria;D_1__Proteobacteria;D_2__Alphaproteobacteria;D_3__Rhizobiales;D_4__A0839	uncultured bacterium
0,02%	0,03%	0,12%	0,05%	0,03%	0,04%	0,12%	0,06%	D_0__Bacteria;D_1__Proteobacteria;D_2__Alphaproteobacteria;D_3__Caulobacterales;D_4__Caulobacteraceae	Caulobacter
0,05%	0,02%	0,04%	0,09%	0,09%	0,05%	0,08%	0,05%	D_0__Bacteria;D_1__Proteobacteria;D_2__Alphaproteobacteria;D_3__Rhizobiales;D_4__Xanthobacteraceae	Afipia
0,04%	0,02%	0,09%	0,04%	0,08%	0,02%	0,12%	0,04%	D_0__Bacteria;D_1__Actinobacteria;D_2__Actinobacteria;D_3__Micromonosporales;D_4__Micromonosporaceae	Asanoa
0,00%	0,08%	0,10%	0,26%	0,01%	0,01%	0,00%	0,00%	D_0__Archaea;D_1__Thaumarchaeota;D_2__Nitrososphaeria;D_3__Nitrososphaerales;D_4__Nitrososphaeraceae	uncultured archaeon
0,07%	0,08%	0,04%	0,05%	0,04%	0,07%	0,02%	0,07%	D_0__Bacteria;D_1__Actinobacteria;D_2__MB-A2-108;Other;Other;Other	
0,05%	0,04%	0,03%	0,04%	0,02%	0,09%	0,07%	0,11%	D_0__Bacteria;D_1__Proteobacteria;D_2__Alphaproteobacteria;D_3__Rhizobiales;D_4__KF-JG30-B3	metagenome
0,04%	0,12%	0,05%	0,05%	0,02%	0,05%	0,06%	0,06%	D_0__Bacteria;D_1__Acidobacteria;D_2__Subgroup 5;D_3__uncultured bacterium;D_4__	
0,00%	0,16%	0,15%	0,01%	0,01%	0,06%	0,01%	0,03%	D_0__Bacteria;D_1__Bacteroidetes;D_2__Bacteroidia;D_3__Chitinophagales;D_4__Chitinophagaceae	Parasegetibacter
0,06%	0,07%	0,14%	0,05%	0,05%	0,01%	0,03%	0,00%	D_0__Bacteria;D_1__Chloroflexi;D_2__Gitt-GS-136;D_3__uncultured bacterium;D_4__	
0,07%	0,06%	0,03%	0,08%	0,04%	0,08%	0,03%	0,03%	D_0__Bacteria;D_1__Proteobacteria;D_2__Gammaproteobacteria;D_3__Betaproteobacteriales;D_4__Nitrosomonadaceae	mle1-7
0,04%	0,06%	0,07%	0,05%	0,02%	0,04%	0,04%	0,09%	D_0__Bacteria;D_1__Actinobacteria;D_2__Acidimicrobiia;D_3__Microtrichales;D_4__uncultured;Other	
0,06%	0,03%	0,04%	0,07%	0,03%	0,07%	0,03%	0,09%	D_0__Bacteria;D_1__Chloroflexi;D_2__Anaerolineae;D_3__Anaerolineales;D_4__Anaerolineaceae	uncultured
0,04%	0,07%	0,06%	0,04%	0,03%	0,08%	0,03%	0,07%	D_0__Bacteria;D_1__Actinobacteria;D_2__Acidimicrobiia;D_3__IMCC26256;D_4__uncultured Acidimicrobidae bacterium	
0,06%	0,03%	0,05%	0,07%	0,03%	0,03%	0,08%	0,05%	D_0__Bacteria;D_1__Proteobacteria;D_2__Alphaproteobacteria;D_3__Sphingomonadales;D_4__Sphingomonadaceae;Other	
