# Supplementary material for: Evaluation of the lignocellulose degradation potential of Mediterranean forests soil microbial communities through diversity and targeted functional metagenomics
Source: Front Microbiol. 2023 Feb 27;14:1121993. doi: 10.3389/fmicb.2023.1121993 (PMC10008878; doi:10.3389/fmicb.2023.1121993)
Supplement: Supplementary file 4 [file Data_Sheet_4.PDF]

Table S5. The 200 most abundant genera of fungi in Andros forest; (A) horizon a , (B) horizon b, (C) horizon c, in Parnitha forest; (D) horizon a, (E) horizon b, (F) horizon c.

(A)

AI1a	AI2a	AI3a	AI4a	AII1a	AII2a	AII3a	AII4a	taxonomy	
0,39%	0,27%	0,46%	63,42%	0,07%	0,16%	1,66%	0,08%	k__Fungi;p__Basidiomycota;c__Agaricomycetes;o__Russulales;f__Lachnocladiaceae	Vararia
68,28%	0,75%	0,24%	0,21%	0,20%	0,02%	0,02%	0,01%	k__Fungi;p__Ascomycota;c__Sordariomycetes;o__Diaporthales;f__Schizoparmaceae	Coniella
0,00%	0,00%	0,00%	0,04%	38,87%	0,14%	10,03%	0,21%	k__Fungi;p__Basidiomycota;c__Agaricomycetes;o__Agaricales;f__Agaricaceae	Agaricus
6,80%	8,73%	1,34%	3,23%	6,46%	7,38%	4,25%	6,32%	k__Fungi;p__Ascomycota;c__Eurotiomycetes;o__Eurotiales;f__Aspergillaceae	Penicillium
0,36%	0,09%	0,09%	0,37%	7,20%	0,36%	13,76%	22,09%	k__Fungi;p__Basidiomycota;c__Agaricomycetes;o__Sebacinales;f__Sebacinaceae	Sebacina
4,20%	24,49%	8,00%	1,07%	1,17%	0,86%	0,76%	0,64%	k__Fungi;p__Ascomycota;c__Sordariomycetes;o__Sordariales;f__Chaetomiaceae;Other	Chaetomiaceae_Other
0,23%	0,41%	0,16%	0,16%	2,91%	23,18%	10,10%	1,97%	k__Fungi;p__Basidiomycota;c__Agaricomycetes;o__Thelephorales;f__Thelephoraceae	Thelephoraceae_unidentified
1,23%	1,70%	0,68%	17,01%	0,14%	0,26%	0,12%	0,17%	k__Fungi;p__Basidiomycota;c__Agaricomycetes;o__Trechisporales;f__Hydnodontaceae	Trechispora
0,08%	0,42%	0,09%	0,15%	0,19%	0,18%	0,37%	32,11%	k__Fungi;p__Ascomycota;c__Sordariomycetes;o__Hypocreales;f__Clavicipitaceae	Metarhizium
3,48%	18,90%	6,39%	0,83%	0,90%	0,54%	0,46%	0,42%	k__Fungi;p__Ascomycota;c__Sordariomycetes;o__Sordariales;f__Chaetomiaceae	Humicola
0,06%	0,06%	29,72%	0,04%	0,00%	0,00%	0,00%	0,00%	k__Fungi;p__Ascomycota;c__Sordariomycetes;o__Hypocreales;f__Ophiocordycipitaceae	Ophiocordyceps
0,40%	0,32%	0,25%	0,54%	3,48%	10,20%	12,68%	1,46%	k__Fungi;p__Basidiomycota;c__Agaricomycetes;o__Russulales;f__Russulaceae	Russula
1,46%	15,21%	0,11%	0,12%	1,62%	2,66%	0,21%	0,07%	k__Fungi;p__Basidiomycota;c__Tremellomycetes;o__Filobasidiales;f__Piskurozymaceae	Solicoccozyma
0,04%	0,06%	21,36%	0,02%	0,00%	0,00%	0,00%	0,00%	k__Fungi;p__Ascomycota;c__Lecanoromycetes;o__Peltigerales;f__Collemataceae	Leptogium
0,02%	0,02%	0,02%	0,03%	0,41%	15,41%	0,92%	0,19%	k__Fungi;p__Basidiomycota;c__Agaricomycetes;o__Thelephorales;f__Thelephoraceae	Tomentella
0,01%	0,03%	0,01%	0,07%	8,37%	6,75%	0,17%	0,29%	k__Fungi;p__Basidiomycota;c__Agaricomycetes;o__Agaricales;f__Inocybaceae	Inocybe
3,64%	1,46%	0,33%	0,66%	3,27%	0,26%	0,73%	3,50%	k__Fungi;p__Mortierellomycota;c__Mortierellomycetes;o__Mortierellales;f__Mortierellaceae	Mortierella
0,56%	2,30%	1,29%	0,62%	1,70%	1,41%	3,14%	0,91%	k__Fungi;p__unidentified;c__unidentified;o__unidentified;f__unidentified	Fungi_unidentified
0,07%	0,49%	11,04%	0,06%	0,01%	0,19%	0,10%	0,07%	k__Fungi;p__Basidiomycota;c__Tremellomycetes;o__Trichosporonales;f__Trichosporonaceae	Cutaneotrichosporon
0,16%	1,15%	3,51%	1,24%	0,07%	0,12%	0,85%	1,51%	k__Fungi;p__Ascomycota;c__Leotiomycetes;o__Thelebolales;f__Pseudeurotiaceae;Other	Pseudeurotiaceae_Other
0,00%	0,00%	0,00%	0,06%	0,02%	0,01%	0,33%	8,13%	k__Fungi;p__Basidiomycota;c__Agaricomycetes;o__Agaricales;f__Omphalotaceae	Rhodocollybia
0,01%	0,34%	0,00%	0,03%	0,05%	0,03%	7,10%	0,04%	k__Fungi;p__Basidiomycota;c__Agaricomycetes;o__Agaricales;f__Lycoperdaceae	Lycoperdon
0,07%	0,35%	1,31%	0,76%	0,11%	0,16%	1,26%	2,20%	k__Fungi;p__Ascomycota;c__Leotiomycetes;o__Thelebolales;f__Pseudeurotiaceae	Geomyces
0,08%	0,08%	0,08%	0,19%	0,08%	0,12%	5,89%	0,25%	k__Fungi;p__Basidiomycota;c__Agaricomycetes;o__Agaricales;f__Hymenogastraceae	Hebeloma
0,20%	1,71%	0,11%	0,37%	1,73%	0,29%	0,25%	0,53%	k__Fungi;p__Ascomycota;c__Sordariomycetes;o__Hypocreales;f__Hypocreaceae	Trichoderma
0,02%	0,09%	0,01%	0,06%	0,18%	3,31%	0,43%	0,86%	k__Fungi;p__Basidiomycota;c__Agaricomycetes;o__Agaricales;f__Tricholomataceae	Mycena
0,17%	0,22%	0,29%	0,64%	0,24%	0,30%	0,72%	1,34%	k__Fungi;p__Ascomycota;c__Leotiomycetes;o__Helotiales;f__Myxotrichaceae	Oidiodendron
0,12%	1,87%	0,05%	0,11%	0,48%	1,10%	0,32%	0,14%	k__Fungi;p__Ascomycota;c__Dothideomycetes;o__Pleosporales;f__Sporormiaceae	Preussia
0,02%	0,19%	3,08%	0,05%	0,06%	0,05%	0,41%	0,17%	k__Fungi;p__Basidiomycota;c__Tremellomycetes;o__Trichosporonales;f__Trichosporonaceae	Apiotrichum
0,00%	0,00%	0,00%	0,01%	0,33%	3,53%	0,12%	0,03%	k__Fungi;p__Ascomycota;c__Pezizomycetes;o__Pezizales;f__Helvellaceae	Helvella
0,03%	0,37%	0,22%	0,17%	0,27%	1,40%	0,71%	0,16%	k__Fungi;p__Ascomycota;c__Dothideomycetes;o__Capnodiales;f__Cladosporiaceae	Cladosporium
0,62%	0,92%	1,04%	0,09%	0,12%	0,06%	0,08%	0,10%	k__Fungi;p__Ascomycota;c__Sordariomycetes;o__Sordariales;f__Chaetomiaceae	Chaetomium
0,13%	1,58%	0,05%	0,18%	0,26%	0,16%	0,21%	0,16%	k__Fungi;p__Ascomycota;c__Eurotiomycetes;o__Eurotiales;f__Trichocomaceae	Talaromyces
0,03%	0,12%	0,09%	0,27%	0,52%	0,51%	0,66%	0,43%	k__Fungi;p__Ascomycota;c__Leotiomycetes;o__Helotiales;f__unidentified	unidentified
0,37%	0,27%	0,18%	0,18%	0,56%	0,16%	0,30%	0,45%	k__Fungi;p__Basidiomycota;c__Tremellomycetes;o__Tremellales;f__Trimorphomycetaceae	Saitozyma
0,11%	0,18%	0,08%	0,24%	0,48%	0,52%	0,38%	0,31%	k__Fungi;p__Basidiomycota;c__Agaricomycetes;o__Agaricales;f__Cortinariaceae	Cortinarius
0,18%	0,12%	0,03%	0,30%	0,49%	0,20%	0,35%	0,50%	k__Fungi;p__Ascomycota;c__Eurotiomycetes;o__Chaetothyriales;f__Herpotrichiellaceae	Exophiala
0,09%	0,22%	0,09%	0,33%	0,25%	0,24%	0,39%	0,36%	k__Fungi;p__Ascomycota;Other;Other;Other;Other	
0,31%	0,58%	0,27%	0,17%	0,17%	0,22%	0,20%	0,21%	k__Fungi;p__Ascomycota;c__Eurotiomycetes;o__Eurotiales;f__Aspergillaceae	Aspergillus
0,03%	0,07%	0,11%	0,11%	0,40%	0,25%	1,00%	0,17%	k__Fungi;p__Ascomycota;c__Leotiomycetes;o__Helotiales;Other;Other	
0,01%	0,01%	0,01%	0,01%	2,18%	0,01%	0,01%	0,00%	k__Fungi;p__Basidiomycota;c__Agaricomycetes;o__Cantharellales;f__Clavulinaceae	Membranomyces
0,28%	1,10%	0,55%	0,06%	0,08%	0,04%	0,04%	0,04%	k__Fungi;p__Ascomycota;c__Sordariomycetes;o__Sordariales;f__Chaetomiaceae	unidentified
0,00%	0,01%	0,00%	0,01%	0,48%	1,31%	0,21%	0,02%	k__Fungi;p__Ascomycota;c__Dothideomycetes;o__Dothideales;f__Aureobasidiaceae	Aureobasidium
0,01%	0,00%	0,01%	0,03%	0,93%	0,13%	0,52%	0,10%	k__Fungi;p__Ascomycota;c__Sordariomycetes;o__Myrmecridiales;f__unidentified	unidentified
0,03%	0,04%	0,02%	0,12%	0,30%	0,35%	0,40%	0,31%	k__Fungi;p__Ascomycota;c__Sordariomycetes;o__Coniochaetales;f__Coniochaetaceae	Coniochaeta
0,09%	0,27%	0,07%	0,13%	0,27%	0,28%	0,25%	0,15%	k__Fungi;p__Basidiomycota;c__Agaricomycetes;o__Agaricales;f__Tricholomataceae	Tricholoma
0,05%	0,17%	0,05%	0,15%	0,23%	0,22%	0,25%	0,29%	k__Fungi;p__Ascomycota;c__Sordariomycetes;o__Hypocreales;f__unidentified	unidentified
0,00%	0,00%	0,00%	0,00%	0,05%	1,42%	0,03%	0,01%	k__Fungi;p__Ascomycota;c__Dothideomycetes;o__Pleosporales;f__Pleomassariaceae	Prosthemium
0,00%	0,00%	0,00%	0,10%	0,28%	0,37%	0,29%	0,30%	k__Fungi;p__Ascomycota;c__Eurotiomycetes;o__Eurotiales;f__Thermoascaceae	Byssochlamys
0,01%	0,70%	0,01%	0,04%	0,10%	0,17%	0,14%	0,06%	k__Fungi;p__Ascomycota;c__Dothideomycetes;o__Pleosporales;f__unidentified	unidentified
0,01%	0,00%	0,00%	0,02%	0,14%	0,45%	0,42%	0,15%	k__Fungi;p__Basidiomycota;c__Agaricomycetes;o__Agaricales;f__Tricholomataceae	unidentified
0,00%	0,02%	0,00%	0,03%	0,01%	0,01%	0,01%	1,07%	k__Fungi;p__Basidiomycota;c__Agaricomycetes;o__Geastrales;f__Sclerogastraceae	Sclerogaster
0,05%	0,05%	0,02%	0,09%	0,14%	0,22%	0,28%	0,08%	k__Fungi;p__Ascomycota;c__Eurotiomycetes;o__Chaetothyriales;f__Herpotrichiellaceae;Other	
0,00%	0,19%	0,02%	0,01%	0,04%	0,05%	0,63%	0,03%	k__Fungi;p__Ascomycota;c__Sordariomycetes;o__Hypocreales;f__Cordycipitaceae	Beauveria
0,01%	0,00%	0,95%	0,00%	0,00%	0,01%	0,00%	0,00%	k__Fungi;p__Ascomycota;c__Sordariomycetes;o__Hypocreales;f__Clavicipitaceae	unidentified
0,00%	0,00%	0,00%	0,00%	0,01%	0,01%	0,91%	0,01%	k__Fungi;p__Ascomycota;c__Pezizomycetes;o__Pezizales;f__Pyronemataceae	Humaria
0,02%	0,03%	0,02%	0,07%	0,12%	0,15%	0,12%	0,24%	k__Fungi;p__Ascomycota;c__Eurotiomycetes;o__Eurotiales;f__Trichocomaceae	Sagenomella
0,01%	0,01%	0,01%	0,02%	0,29%	0,07%	0,30%	0,06%	k__Fungi;p__Ascomycota;c__Sordariomycetes;o__Hypocreales;f__Nectriaceae	Neonectria
0,01%	0,05%	0,00%	0,01%	0,07%	0,24%	0,33%	0,05%	k__Fungi;p__Ascomycota;c__Sordariomycetes;o__Sordariales;f__Lasiosphaeriaceae	unidentified
0,04%	0,21%	0,01%	0,04%	0,10%	0,13%	0,12%	0,07%	k__Fungi;p__Ascomycota;c__Sordariomycetes;o__unidentified;f__unidentified	unidentified
0,02%	0,01%	0,01%	0,04%	0,16%	0,11%	0,32%	0,06%	k__Fungi;p__Ascomycota;c__Leotiomycetes;o__Helotiales;f__Helotiales_fam_Incertae_sedis	Cadophora
0,03%	0,18%	0,01%	0,14%	0,03%	0,10%	0,07%	0,04%	k__Fungi;p__Ascomycota;c__Sordariomycetes;o__Microascales;f__Microascaceae	Cephalotrichum
0,01%	0,02%	0,02%	0,05%	0,27%	0,07%	0,09%	0,14%	k__Fungi;p__Ascomycota;c__Sordariomycetes;o__Hypocreales;f__Hypocreales_fam_Incertae_sedis	Acremonium
0,00%	0,00%	0,00%	0,00%	0,05%	0,62%	0,02%	0,01%	k__Fungi;p__Ascomycota;c__Leotiomycetes;o__Helotiales;f__Sclerotiniaceae;Other	
0,01%	0,08%	0,11%	0,03%	0,09%	0,04%	0,26%	0,03%	k__Fungi;p__Ascomycota;c__Dothideomycetes;o__Venturiales;f__Venturiaceae	unidentified
0,02%	0,15%	0,21%	0,05%	0,06%	0,04%	0,03%	0,08%	k__Fungi;p__Mucoromycota;c__Umbelopsidomycetes;o__Umbelopsidales;f__Umbelopsidaceae	Umbelopsis
0,02%	0,09%	0,02%	0,06%	0,11%	0,16%	0,04%	0,06%	k__Fungi;Other;Other;Other;Other;Other	
0,01%	0,05%	0,02%	0,05%	0,12%	0,12%	0,18%	0,02%	k__Fungi;p__Ascomycota;c__Leotiomycetes;o__Helotiales;f__Helotiaceae	Meliniomyces
0,00%	0,03%	0,10%	0,17%	0,02%	0,04%	0,03%	0,03%	k__Fungi;p__Basidiomycota;c__Agaricomycetes;o__Tremellodendropsidales;f__unidentified	unidentified
0,05%	0,09%	0,04%	0,03%	0,04%	0,01%	0,23%	0,01%	k__Fungi;p__Basidiomycota;c__Agaricomycetes;o__Thelephorales;f__Thelephoraceae	Pseudotomentella
0,00%	0,00%	0,00%	0,06%	0,08%	0,20%	0,07%	0,06%	k__Fungi;p__Basidiomycota;c__Agaricomycetes;o__Trechisporales;Other;Other	
0,01%	0,02%	0,12%	0,01%	0,25%	0,03%	0,10%	0,01%	k__Fungi;p__Ascomycota;c__Leotiomycetes;o__Helotiales;f__Hyaloscyphaceae	Lachnum
0,02%	0,02%	0,04%	0,03%	0,07%	0,05%	0,20%	0,08%	k__Fungi;p__Ascomycota;c__Sordariomycetes;o__Sordariales;Other;Other	
0,00%	0,10%	0,00%	0,05%	0,10%	0,13%	0,05%	0,05%	k__Fungi;p__Ascomycota;c__Dothideomycetes;o__Pleosporales;Other;Other	
0,01%	0,11%	0,00%	0,01%	0,06%	0,27%	0,04%	0,03%	k__Fungi;p__Ascomycota;c__Sordariomycetes;o__Sordariales;f__Lasiosphaeriaceae	Apodus
0,06%	0,06%	0,03%	0,08%	0,02%	0,03%	0,02%	0,12%	k__Fungi;p__Ascomycota;c__Saccharomycetes;o__Saccharomycetales;f__unidentified	unidentified
0,06%	0,18%	0,00%	0,02%	0,10%	0,05%	0,03%	0,02%	k__Fungi;p__Ascomycota;c__Leotiomycetes;o__Thelebolales;f__Thelebolaceae	Thelebolus
0,00%	0,02%	0,00%	0,00%	0,00%	0,44%	0,01%	0,00%	k__Fungi;p__Basidiomycota;c__Agaricomycetes;o__Agaricales;f__Bolbitiaceae	Agrocybe
0,01%	0,05%	0,01%	0,01%	0,06%	0,15%	0,04%	0,15%	k__Fungi;p__Basidiomycota;c__Agaricomycetes;o__Agaricales;f__Tricholomataceae	Mycenella
0,00%	0,00%	0,00%	0,04%	0,10%	0,09%	0,12%	0,08%	k__Fungi;p__Ascomycota;c__Sordariomycetes;o__Microascales;f__Microascaceae	Pseudallescheria
0,00%	0,00%	0,00%	0,04%	0,09%	0,12%	0,07%	0,10%	k__Fungi;p__Basidiomycota;c__Agaricomycetes;o__Geastrales;f__Geastraceae	Geastrum
0,01%	0,03%	0,03%	0,02%	0,02%	0,09%	0,18%	0,06%	k__Fungi;p__Ascomycota;c__Pezizomycetes;o__Pezizales;f__Tuberaceae	Tuber
0,10%	0,18%	0,05%	0,05%	0,00%	0,02%	0,00%	0,00%	k__Fungi;p__Basidiomycota;c__Agaricomycetes;o__Agaricales;f__Hygrophoraceae	unidentified
0,07%	0,05%	0,04%	0,05%	0,04%	0,06%	0,04%	0,03%	k__Fungi;p__Ascomycota;c__Sordariomycetes;o__Sordariales;f__unidentified	unidentified
0,00%	0,02%	0,00%	0,01%	0,24%	0,11%	0,06%	0,00%	k__Fungi;p__Ascomycota;c__Dothideomycetes;o__Pleosporales;f__Melanommataceae;Other	
0,01%	0,09%	0,01%	0,02%	0,06%	0,10%	0,09%	0,04%	k__Fungi;p__Ascomycota;c__unidentified;o__unidentified;f__unidentified	unidentified
0,03%	0,03%	0,01%	0,05%	0,13%	0,05%	0,05%	0,04%	k__Fungi;p__Ascomycota;c__Dothideomycetes;o__Capnodiales;f__unidentified	unidentified
0,00%	0,16%	0,04%	0,03%	0,02%	0,02%	0,04%	0,07%	k__Fungi;p__Ascomycota;c__Leotiomycetes;o__Thelebolales;f__Pseudeurotiaceae	Pseudogymnoascus
0,00%	0,00%	0,00%	0,00%	0,05%	0,31%	0,04%	0,00%	k__Fungi;p__Ascomycota;c__Dothideomycetes;o__Pleosporales;f__Pleomassariaceae;Other	
0,08%	0,07%	0,01%	0,01%	0,12%	0,05%	0,02%	0,02%	k__Fungi;p__Basidiomycota;c__Tremellomycetes;o__unidentified;f__unidentified	unidentified
0,00%	0,00%	0,01%	0,11%	0,02%	0,02%	0,02%	0,10%	k__Fungi;p__Ascomycota;c__Eurotiomycetes;o__Onygenales;f__Ajellomycetaceae	Histoplasma

0,00%	0,00%	0,00%	0,03%	0,08%	0,07%	0,11%	0,08%	k__Fungi;p__Basidiomycota;c__Agaricomycetes;o__Agaricales;f__Psathyrellaceae	unidentified
0,05%	0,05%	0,04%	0,05%	0,04%	0,04%	0,03%	0,03%	k__Fungi;p__Basidiomycota;c__Agaricomycetes;o__Gomphales;f__Gomphaceae	Clavariadelphus
0,00%	0,06%	0,10%	0,01%	0,03%	0,02%	0,12%	0,01%	k__Fungi;p__Ascomycota;c__Dothideomycetes;o__Venturiales;Other;Other	
0,01%	0,11%	0,00%	0,01%	0,08%	0,05%	0,06%	0,02%	k__Fungi;p__Ascomycota;c__Eurotiomycetes;o__Chaetothyriales;f__unidentified	unidentified
0,04%	0,12%	0,00%	0,08%	0,03%	0,01%	0,00%	0,00%	k__Fungi;p__Chytridiomycota;c__unidentified;o__unidentified;f__unidentified	unidentified
0,05%	0,04%	0,02%	0,02%	0,07%	0,05%	0,07%	0,03%	k__Fungi;p__Ascomycota;c__Sordariomycetes;Other;Other;Other	
0,05%	0,11%	0,04%	0,01%	0,04%	0,01%	0,03%	0,03%	k__Fungi;p__Ascomycota;c__Sordariomycetes;o__Ophiostomatales;f__Ophiostomataceae	Sporothrix
0,00%	0,00%	0,00%	0,01%	0,02%	0,02%	0,26%	0,03%	k__Fungi;p__Ascomycota;c__Pezizomycetes;o__Pezizales;f__Pyronemataceae	Otidea
0,01%	0,00%	0,01%	0,04%	0,05%	0,07%	0,08%	0,04%	k__Fungi;p__Ascomycota;c__Eurotiomycetes;o__Chaetothyriales;f__Herpotrichiellaceae	Cladophialophora
0,00%	0,00%	0,00%	0,00%	0,00%	0,00%	0,00%	0,33%	k__Fungi;p__Basidiomycota;c__Agaricomycetes;o__Boletales;f__Gyroporaceae	Gyroporus
0,01%	0,04%	0,00%	0,01%	0,05%	0,14%	0,05%	0,02%	k__Fungi;p__Ascomycota;c__Dothideomycetes;o__Pleosporales;f__Didymellaceae;Other	
0,03%	0,14%	0,05%	0,01%	0,02%	0,02%	0,03%	0,01%	k__Fungi;p__Ascomycota;c__Sordariomycetes;o__Chaetosphaeriales;f__Chaetosphaeriaceae	Chloridium
0,00%	0,00%	0,00%	0,00%	0,04%	0,24%	0,00%	0,00%	k__Fungi;p__Ascomycota;c__Sordariomycetes;o__Xylariales;f__Xylariaceae	Rosellinia
0,00%	0,00%	0,00%	0,00%	0,26%	0,00%	0,01%	0,00%	k__Fungi;p__Basidiomycota;c__Agaricomycetes;o__Boletales;f__Sclerodermataceae	Scleroderma
0,00%	0,00%	0,00%	0,02%	0,07%	0,06%	0,07%	0,05%	k__Fungi;p__Basidiomycota;c__Agaricomycetes;o__Boletales;f__Boletaceae	Boletus
0,01%	0,05%	0,01%	0,02%	0,03%	0,07%	0,06%	0,01%	k__Fungi;p__Ascomycota;c__Dothideomycetes;o__Capnodiales;Other;Other	
0,00%	0,00%	0,00%	0,00%	0,01%	0,25%	0,01%	0,01%	k__Fungi;p__Ascomycota;c__Eurotiomycetes;o__Onygenales;f__Onygenaceae	unidentified
0,00%	0,05%	0,00%	0,02%	0,05%	0,05%	0,03%	0,06%	k__Fungi;p__Ascomycota;c__Sordariomycetes;o__Hypocreales;f__Nectriaceae	Fusarium
0,15%	0,09%	0,02%	0,00%	0,00%	0,00%	0,00%	0,00%	k__Fungi;p__Basidiomycota;c__Tremellomycetes;o__Tremellales;f__unidentified	unidentified
0,00%	0,04%	0,01%	0,00%	0,08%	0,01%	0,13%	0,00%	k__Fungi;p__Ascomycota;c__Dothideomycetes;o__Venturiales;f__Venturiaceae	Tothia
0,00%	0,00%	0,00%	0,00%	0,12%	0,08%	0,04%	0,01%	k__Fungi;p__Ascomycota;c__Dothideomycetes;o__Pleosporales;f__Melanommataceae	unidentified
0,01%	0,16%	0,00%	0,00%	0,01%	0,06%	0,00%	0,00%	k__Fungi;p__Ascomycota;c__Dothideomycetes;o__Dothideomycetes_ord_Incertae_sedis;f__Eremomycetaceae	Eremomyces
0,00%	0,00%	0,00%	0,00%	0,02%	0,04%	0,15%	0,02%	k__Fungi;p__Ascomycota;c__Sordariomycetes;o__Sordariales;f__Lasiosphaeriaceae	Podospora
0,01%	0,03%	0,01%	0,03%	0,02%	0,04%	0,04%	0,03%	k__Fungi;p__Ascomycota;c__Sordariomycetes;o__Hypocreales;Other;Other	
0,00%	0,00%	0,00%	0,01%	0,05%	0,04%	0,07%	0,05%	k__Fungi;p__Ascomycota;c__Saccharomycetes;o__Saccharomycetales;f__Dipodascaceae	Geotrichum
0,09%	0,00%	0,01%	0,00%	0,12%	0,01%	0,01%	0,00%	k__Fungi;p__Ascomycota;c__Dothideomycetes;o__Capnodiales;f__Capnodiaceae	Readerielliopsis
0,02%	0,21%	0,00%	0,00%	0,00%	0,00%	0,00%	0,00%	k__Fungi;p__Chytridiomycota;c__Rhizophydiomycetes;o__Rhizophydiales;f__Rhizophydiales_fam_Incertae_sedis	Operculomyces
0,06%	0,05%	0,01%	0,00%	0,05%	0,01%	0,04%	0,01%	k__Fungi;p__Ascomycota;c__Pezizomycotina_cls_Incertae_sedis;o__Pezizomycotina_ord_Incertae_sedis;f__Pezizomycotina_fam_Incertae_sedis	Ciliophora
0,00%	0,00%	0,00%	0,01%	0,10%	0,08%	0,02%	0,01%	k__Fungi;p__Ascomycota;c__Dothideomycetes;o__Venturiales;f__Venturiaceae;Other	
0,02%	0,00%	0,01%	0,02%	0,03%	0,04%	0,05%	0,04%	k__Fungi;p__Ascomycota;c__Dothideomycetes;o__Mytilinidales;f__Gloniaceae	Cenococcum
0,00%	0,02%	0,01%	0,04%	0,03%	0,03%	0,03%	0,03%	k__Fungi;p__Ascomycota;c__Sordariomycetes;o__Hypocreales;f__Nectriaceae;Other	
0,00%	0,05%	0,00%	0,01%	0,04%	0,04%	0,03%	0,03%	k__Fungi;p__Ascomycota;c__Sordariomycetes;o__Sordariales;f__Sordariales_fam_Incertae_sedis	Ramophialophora
0,01%	0,00%	0,00%	0,00%	0,03%	0,13%	0,02%	0,01%	k__Fungi;p__Basidiomycota;c__Agaricomycetes;o__Thelephorales;f__Thelephoraceae;Other	
0,00%	0,21%	0,00%	0,00%	0,00%	0,00%	0,00%	0,00%	k__Fungi;p__Basidiomycota;c__Tremellomycetes;o__Filobasidiales;f__Filobasidiaceae	Naganishia
0,00%	0,00%	0,00%	0,01%	0,03%	0,05%	0,08%	0,02%	k__Fungi;p__Basidiomycota;c__Agaricomycetes;o__Agaricales;f__Hymenogastraceae	Hymenogaster
0,00%	0,00%	0,00%	0,03%	0,03%	0,02%	0,03%	0,06%	k__Fungi;p__Ascomycota;c__Leotiomycetes;o__Helotiales;f__Myxotrichaceae	unidentified
0,00%	0,12%	0,00%	0,00%	0,00%	0,07%	0,01%	0,01%	k__Fungi;p__Ascomycota;c__Eurotiomycetes;o__Onygenales;Other;Other	
0,01%	0,01%	0,03%	0,01%	0,02%	0,07%	0,03%	0,02%	k__Fungi;p__Ascomycota;c__Sordariomycetes;o__Chaetosphaeriales;f__Chaetosphaeriaceae;Other	
0,00%	0,00%	0,00%	0,00%	0,07%	0,09%	0,02%	0,01%	k__Fungi;p__Basidiomycota;c__Agaricomycetes;o__Thelephorales;f__Thelephoraceae	Thelephora
0,01%	0,02%	0,00%	0,01%	0,04%	0,03%	0,07%	0,02%	k__Fungi;p__Ascomycota;c__Sordariomycetes;o__Hypocreales;f__Nectriaceae	Ilyonectria
0,01%	0,07%	0,01%	0,00%	0,02%	0,05%	0,01%	0,01%	k__Fungi;p__Ascomycota;c__Dothideomycetes;o__Botryosphaeriales;f__unidentified	unidentified
0,00%	0,00%	0,00%	0,00%	0,19%	0,00%	0,00%	0,00%	k__Fungi;p__Chytridiomycota;c__Spizellomycetes;o__Spizellomycetales;f__Spizellomycetaceae	Spizellomyces
0,00%	0,00%	0,00%	0,02%	0,03%	0,04%	0,03%	0,05%	k__Fungi;p__Ascomycota;c__Sordariomycetes;o__Calosphaeriales;f__Pleurostomataceae	Pleurostoma
0,02%	0,06%	0,03%	0,01%	0,02%	0,02%	0,02%	0,02%	k__Fungi;p__Ascomycota;c__Sordariomycetes;o__Hypocreales;f__Nectriaceae	unidentified
0,00%	0,00%	0,00%	0,01%	0,05%	0,05%	0,04%	0,03%	k__Fungi;p__Basidiomycota;c__Agaricomycetes;o__Boletales;f__Boletaceae	Neoboletus
0,00%	0,00%	0,00%	0,02%	0,04%	0,04%	0,04%	0,04%	k__Fungi;p__Ascomycota;c__Sordariomycetes;o__Coniochaetales;f__Coniochaetaceae	Lecythophora
0,01%	0,05%	0,01%	0,01%	0,04%	0,05%	0,02%	0,01%	k__Fungi;p__Ascomycota;c__Dothideomycetes;Other;Other;Other	
0,03%	0,00%	0,00%	0,01%	0,08%	0,02%	0,03%	0,02%	k__Fungi;p__Ascomycota;c__Dothideomycetes;o__Venturiales;f__Venturiaceae	Venturia
0,00%	0,06%	0,00%	0,00%	0,01%	0,07%	0,02%	0,01%	k__Fungi;p__Ascomycota;c__Dothideomycetes;o__unidentified;f__unidentified	unidentified
0,00%	0,00%	0,00%	0,00%	0,00%	0,00%	0,00%	0,18%	k__Fungi;p__Basidiomycota;c__Agaricomycetes;o__Agaricales;f__Agaricaceae	Gigasperma
0,03%	0,03%	0,02%	0,03%	0,01%	0,02%	0,01%	0,01%	k__Fungi;p__Basidiomycota;c__Agaricomycetes;o__Agaricales;f__Amanitaceae	Amanita
0,00%	0,00%	0,00%	0,00%	0,01%	0,01%	0,05%	0,11%	k__Fungi;p__Basidiomycota;c__Agaricomycetes;o__Agaricales;f__Agaricaceae	Lepiota
0,00%	0,00%	0,00%	0,02%	0,03%	0,04%	0,04%	0,03%	k__Fungi;p__Ascomycota;c__Leotiomycetes;o__Helotiales;f__Dermateaceae	Cryptosporiopsis
0,00%	0,01%	0,00%	0,01%	0,06%	0,05%	0,03%	0,01%	k__Fungi;p__Ascomycota;c__Sordariomycetes;o__Sordariales;f__Lasiosphaeriaceae;Other	
0,00%	0,04%	0,00%	0,01%	0,03%	0,03%	0,03%	0,03%	k__Fungi;p__Basidiomycota;c__Agaricomycetes;o__Sebacinales;f__Sebacinaceae	Helvellosebacina
0,01%	0,03%	0,01%	0,01%	0,01%	0,03%	0,05%	0,02%	k__Fungi;p__Ascomycota;c__Sordariomycetes;o__Hypocreales;f__Cordycipitaceae	Lecanicillium
0,00%	0,00%	0,00%	0,01%	0,03%	0,04%	0,05%	0,03%	k__Fungi;p__Ascomycota;c__Leotiomycetes;o__Helotiales;f__Helotiaceae	Helicodendron
0,05%	0,01%	0,01%	0,00%	0,05%	0,01%	0,03%	0,01%	k__Fungi;p__Olpidiomycota;c__GS17;o__unidentified;f__unidentified	unidentified
0,00%	0,00%	0,00%	0,02%	0,04%	0,00%	0,00%	0,07%	k__Fungi;p__Basidiomycota;c__Agaricomycetes;o__Boletales;f__Melanogastraceae	Melanogaster
0,02%	0,01%	0,02%	0,03%	0,02%	0,01%	0,02%	0,01%	k__Fungi;p__Ascomycota;c__Sordariomycetes;o__Chaetosphaeriales;f__Chaetosphaeriaceae	Chaetosphaeria
0,00%	0,01%	0,01%	0,00%	0,01%	0,02%	0,10%	0,00%	k__Fungi;p__Ascomycota;c__Leotiomycetes;o__Helotiales;f__Helotiaceae	Hymenoscyphus
0,00%	0,14%	0,01%	0,00%	0,00%	0,01%	0,00%	0,00%	k__Fungi;p__Ascomycota;c__Sordariomycetes;o__Hypocreales;f__Nectriaceae	Mariannaea
0,00%	0,00%	0,00%	0,01%	0,04%	0,02%	0,04%	0,04%	k__Fungi;p__Ascomycota;c__Saccharomycetes;o__Saccharomycetales;f__Pichiaceae	Pichia
0,04%	0,03%	0,03%	0,02%	0,00%	0,01%	0,00%	0,00%	k__Fungi;p__Basidiomycota;c__Agaricomycetes;o__Polyporales;f__unidentified	unidentified
0,01%	0,06%	0,06%	0,00%	0,00%	0,01%	0,01%	0,01%	k__Fungi;p__Rozellomycota;c__unidentified;o__unidentified;f__unidentified	unidentified
0,00%	0,00%	0,01%	0,01%	0,06%	0,02%	0,03%	0,02%	k__Fungi;p__Ascomycota;c__Sordariomycetes;o__Chaetosphaeriales;Other;Other	
0,01%	0,01%	0,10%	0,01%	0,00%	0,00%	0,01%	0,00%	k__Fungi;p__Basidiomycota;c__Tremellomycetes;o__Trichosporonales;f__Trichosporonaceae	Vanrija
0,00%	0,00%	0,00%	0,01%	0,03%	0,06%	0,03%	0,01%	k__Fungi;p__Ascomycota;c__Dothideomycetes;o__Pleosporales;f__Pleosporaceae	Alternaria
0,00%	0,00%	0,00%	0,02%	0,01%	0,01%	0,00%	0,08%	k__Fungi;p__Basidiomycota;c__Geminibasidiomycetes;o__Geminibasidiales;f__Geminibasidiaceae	Geminibasidium
0,00%	0,08%	0,00%	0,00%	0,01%	0,02%	0,01%	0,00%	k__Fungi;p__Ascomycota;c__Dothideomycetes;o__Pleosporales;f__Cucurbitariaceae	Pyrenochaeta
0,00%	0,02%	0,00%	0,01%	0,02%	0,02%	0,03%	0,02%	k__Fungi;p__Ascomycota;c__Sordariomycetes;o__Hypocreales;f__Nectriaceae	Volutella
0,00%	0,00%	0,00%	0,01%	0,02%	0,02%	0,05%	0,03%	k__Fungi;p__Ascomycota;c__Saccharomycetes;o__Saccharomycetales;f__Dipodascaceae	unidentified
0,00%	0,00%	0,00%	0,01%	0,02%	0,03%	0,03%	0,03%	k__Fungi;p__Ascomycota;c__Pezizomycetes;o__Pezizales;f__Pyronemataceae	Wilcoxina
0,00%	0,00%	0,00%	0,01%	0,03%	0,03%	0,02%	0,03%	k__Fungi;p__Ascomycota;c__Eurotiomycetes;o__Eurotiales;f__Aspergillaceae	Monascus
0,00%	0,00%	0,00%	0,01%	0,02%	0,03%	0,03%	0,02%	k__Fungi;p__Ascomycota;c__Leotiomycetes;o__Helotiales;f__Helotiales_fam_Incertae_sedis	Chalara
0,00%	0,00%	0,12%	0,00%	0,00%	0,00%	0,00%	0,00%	k__Fungi;p__Basidiomycota;c__Agaricomycetes;o__Agaricales;f__Pleurotaceae	Hohenbuehelia
0,00%	0,00%	0,00%	0,01%	0,02%	0,03%	0,03%	0,02%	k__Fungi;p__Ascomycota;c__Leotiomycetes;o__Helotiales;f__Dermateaceae;Other	
0,01%	0,03%	0,01%	0,01%	0,02%	0,01%	0,01%	0,03%	k__Fungi;p__Ascomycota;c__Eurotiomycetes;o__Eurotiales;f__Aspergillaceae;Other	
0,00%	0,01%	0,00%	0,03%	0,02%	0,01%	0,00%	0,01%	k__Fungi;p__Chytridiomycota;Other;Other;Other;Other	
0,00%	0,00%	0,00%	0,01%	0,03%	0,02%	0,03%	0,02%	k__Fungi;p__Basidiomycota;c__Agaricomycetes;o__Hysterangiales;Other;Other	
0,01%	0,02%	0,00%	0,01%	0,02%	0,03%	0,02%	0,01%	k__Fungi;p__Ascomycota;c__Eurotiomycetes;o__Chaetothyriales;Other;Other	
0,00%	0,00%	0,00%	0,00%	0,00%	0,11%	0,00%	0,00%	k__Fungi;p__Ascomycota;c__Pezizomycetes;o__Pezizales;f__Pyronemataceae	Pustularia
0,00%	0,00%	0,01%	0,01%	0,08%	0,00%	0,00%	0,01%	k__Fungi;p__Basidiomycota;c__Agaricomycetes;o__Hymenochaetales;f__Schizoporaceae	Xylodon
0,02%	0,05%	0,00%	0,01%	0,01%	0,01%	0,02%	0,01%	k__Fungi;p__Ascomycota;c__Sordariomycetes;o__Hypocreales;f__Bionectriaceae	Clonostachys
0,01%	0,04%	0,03%	0,01%	0,00%	0,00%	0,01%	0,01%	k__Fungi;p__Ascomycota;c__Eurotiomycetes;o__Eurotiales;f__Trichocomaceae	Rasamsonia
0,01%	0,05%	0,00%	0,01%	0,02%	0,01%	0,01%	0,02%	k__Fungi;p__Ascomycota;c__Eurotiomycetes;o__Eurotiales;f__unidentified	unidentified
0,02%	0,03%	0,01%	0,01%	0,01%	0,01%	0,01%	0,01%	k__Fungi;p__Basidiomycota;c__Agaricomycetes;o__Agaricales;f__Tricholomataceae	Macrocystidia
0,01%	0,02%	0,01%	0,00%	0,01%	0,03%	0,01%	0,01%	k__Fungi;p__Ascomycota;c__Sordariomycetes;o__Xylariales;f__Xylariaceae	Ascotricha
0,00%	0,00%	0,04%	0,01%	0,01%	0,01%	0,02%	0,01%	k__Fungi;p__Ascomycota;c__Leotiomycetes;o__Thelebolales;f__Pseudeurotiaceae	Pseudeurotium
0,00%	0,05%	0,00%	0,00%	0,02%	0,02%	0,02%	0,00%	k__Fungi;p__Ascomycota;c__Dothideomycetes;o__Pleosporales;f__Didymellaceae	Phoma
0,00%	0,00%	0,00%	0,00%	0,03%	0,06%	0,02%	0,00%	k__Fungi;p__Ascomycota;c__Sordariomycetes;o__Xylariales;f__Beltraniaceae	unidentified
0,00%	0,00%	0,00%	0,01%	0,02%	0,03%	0,03%	0,02%	k__Fungi;p__Basidiomycota;c__Agaricomycetes;o__Agaricales;f__Tricholomataceae	Pseudoclitocybe
0,00%	0,00%	0,00%	0,01%	0,02%	0,00%	0,01%	0,05%	k__Fungi;p__Ascomycota;c__Sordariomycetes;o__Hypocreales;f__Clavicipitaceae	Metapochonia

0,00%	0,00%	0,00%	0,01%	0,03%	0,02%	0,02%	0,02%	k__Fungi;p__Ascomycota;c__Saccharomycetes;o__Saccharomycetales;f__Saccharomycetaceae	Williopsis
0,02%	0,02%	0,02%	0,01%	0,02%	0,01%	0,01%	0,00%	k__Fungi;p__Ascomycota;c__Dothideomycetes;o__Venturiales;f__Sympoventuriaceae;Other	
0,00%	0,00%	0,04%	0,00%	0,01%	0,04%	0,01%	0,00%	k__Fungi;p__Basidiomycota;c__Agaricomycetes;o__Sebacinales;Other;Other	
0,00%	0,00%	0,00%	0,00%	0,00%	0,07%	0,01%	0,01%	k__Fungi;p__Ascomycota;c__Leotiomycetes;o__Helotiales;f__Myxotrichaceae	Myxotrichum
0,00%	0,05%	0,00%	0,00%	0,00%	0,04%	0,00%	0,01%	k__Fungi;p__Basidiomycota;c__Tremellomycetes;o__Holtermanniales;f__Holtermanniales_fam_Incertae _sedis	Holtermanniella
0,00%	0,05%	0,00%	0,00%	0,03%	0,01%	0,00%	0,00%	k__Fungi;p__Ascomycota;c__Sordariomycetes;o__Glomerellales;f__Reticulascaceae	Reticulascus
0,01%	0,00%	0,00%	0,00%	0,04%	0,03%	0,01%	0,01%	k__Fungi;p__Basidiomycota;c__Agaricomycetes;o__Cantharellales;f__Clavulinaceae	Clavulina
0,00%	0,00%	0,00%	0,01%	0,02%	0,02%	0,02%	0,02%	k__Fungi;p__Basidiomycota;c__Agaricomycetes;o__Auriculariales;f__Hyaloriaceae	Protodontia
0,01%	0,03%	0,00%	0,00%	0,01%	0,04%	0,00%	0,00%	k__Fungi;p__Ascomycota;c__Dothideomycetes;o__Pleosporales;f__Sporormiaceae	unidentified
0,00%	0,00%	0,00%	0,01%	0,02%	0,03%	0,01%	0,02%	k__Fungi;p__Ascomycota;c__Pezizomycetes;o__Pezizales;f__Sarcoscyphaceae	Desmazierella
0,00%	0,00%	0,00%	0,03%	0,01%	0,00%	0,01%	0,01%	k__Fungi;p__Ascomycota;c__Sordariomycetes;o__Xylariales;f__Amphisphaeriaceae	Polyscytalum
0,00%	0,04%	0,00%	0,01%	0,01%	0,03%	0,00%	0,00%	k__Fungi;p__Ascomycota;c__Dothideomycetes;o__Pleosporales;f__Lophiotremataceae	Lophiotrema
0,01%	0,00%	0,00%	0,01%	0,01%	0,02%	0,01%	0,03%	k__Fungi;p__Ascomycota;c__Eurotiomycetes;o__Onygenales;f__Onygenaceae	Auxarthron
0,00%	0,00%	0,00%	0,01%	0,02%	0,03%	0,02%	0,01%	k__Fungi;p__Ascomycota;c__Dothideomycetes;o__Pleosporales;f__Sporormiaceae	Westerdykella
0,00%	0,02%	0,00%	0,00%	0,01%	0,04%	0,01%	0,00%	k__Fungi;p__Ascomycota;c__Pezizomycetes;o__Pezizales;f__Pezizaceae	Peziza
0,00%	0,00%	0,00%	0,01%	0,00%	0,00%	0,05%	0,01%	k__Fungi;p__Ascomycota;c__Sordariomycetes;o__Sordariales;f__Cephalothecaceae	Phialemonium

(B)

AI1b	AI2b	AI3b	AI4b	AII1b	AII2b	AII3b	AII4b	taxonomy	
13,30%	3,40%	6,30%	16,90%	6,32%	2,45%	1,04%	10,74%	k__Fungi;p__Ascomycota;c__Eurotiomycetes;o__Eurotiales;f__Aspergillaceae	Penicillium
2,18%	0,56%	18,19%	1,11%	4,72%	15,16%	16,70%	2,49%	k__Fungi;p__Basidiomycota;c__Agaricomycetes;o__Russulales;f__Russulaceae	Russula
0,01%	0,00%	0,00%	0,07%	0,65%	0,11%	60,22%	0,15%	k__Fungi;p__Basidiomycota;c__Agaricomycetes;o__Agaricales;f__Agaricaceae	Agaricus
22,56%	22,53%	0,89%	0,11%	1,61%	4,64%	0,15%	0,13%	k__Fungi;p__Basidiomycota;c__Tremellomycetes;o__Filobasidiales;f__Piskurozymaceae	Solicoccozyma
9,72%	0,12%	0,24%	2,32%	10,06%	0,32%	0,78%	21,67%	k__Fungi;p__Basidiomycota;c__Agaricomycetes;o__Sebacinales;f__Sebacinaceae	Sebacina
0,12%	45,69%	0,09%	0,04%	0,00%	0,48%	0,00%	0,00%	k__Fungi;p__Basidiomycota;c__Agaricomycetes;o__Agaricales;f__Hygrophoraceae	Hygrophoraceae_unidentified
7,70%	0,44%	1,19%	9,37%	2,57%	0,32%	0,30%	8,76%	k__Fungi;p__Mortierellomycota;c__Mortierellomycetes;o__Mortierellales;f__Mortierellaceae	Mortierella
0,15%	0,03%	0,03%	0,16%	19,03%	13,10%	0,14%	3,44%	k__Fungi;p__Basidiomycota;c__Agaricomycetes;o__Agaricales;f__Inocybaceae	Inocybe
9,48%	7,70%	5,49%	2,74%	0,77%	0,71%	0,29%	0,46%	k__Fungi;p__Ascomycota;c__Sordariomycetes;o__Sordariales;f__Chaetomiaceae;Other	Chaetomiaceae_Other
0,71%	0,23%	0,59%	10,27%	0,83%	0,44%	0,45%	5,76%	k__Fungi;p__Ascomycota;c__Leotiomycetes;o__Helotiales;f__Myxotrichaceae	Oidiodendron
7,73%	5,70%	4,10%	2,19%	0,60%	0,52%	0,22%	0,32%	k__Fungi;p__Ascomycota;c__Sordariomycetes;o__Sordariales;f__Chaetomiaceae	Humicola
1,84%	0,20%	0,15%	0,26%	4,10%	2,79%	0,32%	13,02%	k__Fungi;p__Basidiomycota;c__Agaricomycetes;o__Agaricales;f__Cortinariaceae	Cortinarius
1,62%	2,44%	1,60%	4,24%	0,17%	7,32%	0,10%	0,62%	k__Fungi;p__Basidiomycota;c__Agaricomycetes;o__Trechisporales;f__Hydnodontaceae	Trechispora
1,16%	0,40%	5,09%	1,43%	10,54%	0,33%	0,11%	1,53%	k__Fungi;p__Ascomycota;c__Sordariomycetes;o__Hypocreales;f__Hypocreaceae	Trichoderma
2,47%	0,11%	0,50%	0,24%	1,85%	10,75%	4,11%	1,34%	k__Fungi;p__Basidiomycota;c__Agaricomycetes;o__Thelephorales;f__Thelephoraceae	Thelephoraceae_unidentified
0,11%	0,14%	0,14%	8,08%	0,14%	0,13%	2,58%	0,45%	k__Fungi;p__Basidiomycota;c__Agaricomycetes;o__Agaricales;f__Hymenogastraceae	Hebeloma
0,18%	0,16%	0,19%	0,18%	15,03%	3,53%	0,16%	0,17%	k__Fungi;p__Basidiomycota;c__Agaricomycetes;o__Agaricales;f__Tricholomataceae	Tricholoma
1,35%	0,79%	1,72%	4,88%	1,49%	1,17%	0,62%	1,95%	k__Fungi;p__unidentified;c__unidentified;o__unidentified;f__unidentified	Fungi_unidentified
0,13%	0,05%	15,24%	0,04%	0,38%	0,01%	0,10%	0,01%	k__Fungi;p__Basidiomycota;c__Agaricomycetes;o__Thelephorales;f__Thelephoraceae	Pseudotomentella
0,32%	0,13%	10,84%	1,18%	0,09%	0,06%	1,13%	0,27%	k__Fungi;p__Ascomycota;c__Leotiomycetes;o__Thelebolales;f__Pseudeurotiaceae;Other	Pseudeurotiaceae_Other
0,10%	0,04%	0,02%	0,04%	0,34%	11,27%	0,33%	0,18%	k__Fungi;p__Basidiomycota;c__Agaricomycetes;o__Thelephorales;f__Thelephoraceae	Tomentella
0,26%	0,13%	0,44%	2,82%	0,58%	0,28%	0,62%	1,79%	k__Fungi;p__Ascomycota;Other;Other;Other;Other	Ascomycota_Other
0,54%	0,08%	0,48%	2,74%	0,57%	0,27%	0,23%	1,65%	k__Fungi;p__Ascomycota;c__Eurotiomycetes;o__Chaetothyriales;f__Herpotrichiellaceae	Exophiala
0,00%	0,00%	0,00%	0,06%	0,10%	8,60%	0,06%	0,08%	k__Fungi;p__Basidiomycota;c__Agaricomycetes;o__Trechisporales;Other;Other	Trechisporales_Other
0,13%	0,08%	3,65%	1,39%	0,13%	0,10%	1,67%	0,40%	k__Fungi;p__Ascomycota;c__Leotiomycetes;o__Thelebolales;f__Pseudeurotiaceae	Geomyces
1,61%	0,46%	0,59%	1,09%	0,77%	0,30%	0,16%	1,47%	k__Fungi;p__Basidiomycota;c__Tremellomycetes;o__Tremellales;f__Trimorphomycetaceae	Saitozyma
0,19%	0,11%	1,11%	0,53%	0,24%	0,25%	0,25%	4,22%	k__Fungi;p__Ascomycota;c__Sordariomycetes;o__Hypocreales;f__Clavicipitaceae	Metarhizium
0,07%	0,04%	0,06%	2,17%	0,02%	0,02%	0,02%	1,71%	k__Fungi;p__Ascomycota;c__Saccharomycetes;o__Saccharomycetales;f__unidentified	unidentified
0,54%	0,07%	0,18%	1,05%	0,88%	0,22%	0,12%	0,64%	k__Fungi;p__Ascomycota;c__Sordariomycetes;o__Hypocreales;f__unidentified	unidentified
0,45%	0,44%	0,57%	1,28%	0,15%	0,17%	0,10%	0,08%	k__Fungi;p__Basidiomycota;c__Agaricomycetes;o__Russulales;f__Lachnocladiaceae	Vararia
1,24%	0,65%	0,77%	0,22%	0,10%	0,06%	0,04%	0,05%	k__Fungi;p__Ascomycota;c__Sordariomycetes;o__Sordariales;f__Chaetomiaceae	Chaetomium
0,60%	0,23%	0,59%	0,67%	0,18%	0,12%	0,07%	0,16%	k__Fungi;p__Ascomycota;c__Eurotiomycetes;o__Eurotiales;f__Aspergillaceae	Aspergillus
0,18%	0,81%	0,23%	0,18%	0,33%	0,88%	0,19%	0,31%	k__Fungi;p__Ascomycota;c__Dothideomycetes;o__Pleosporales;f__Sporormiaceae	Preussia
0,11%	0,02%	0,21%	0,59%	0,43%	0,25%	0,30%	0,72%	k__Fungi;p__Ascomycota;c__Leotiomycetes;o__Helotiales;f__unidentified	unidentified
0,22%	0,02%	0,01%	0,01%	2,47%	0,02%	0,00%	0,01%	k__Fungi;p__Basidiomycota;c__Agaricomycetes;o__Cantharellales;f__Clavulinaceae	Membranomyces
0,19%	0,08%	0,16%	0,49%	0,26%	0,16%	0,07%	0,54%	k__Fungi;p__Ascomycota;c__Eurotiomycetes;o__Eurotiales;f__Trichocomaceae	Sagenomella
0,25%	0,03%	0,50%	0,21%	0,36%	0,29%	0,22%	0,27%	k__Fungi;p__Ascomycota;c__Sordariomycetes;o__Coniochaetales;f__Coniochaetaceae	Coniochaeta
0,20%	0,04%	0,19%	0,57%	0,29%	0,03%	0,01%	0,38%	k__Fungi;p__Mucoromycota;c__Umbelopsidomycetes;o__Umbelopsidales;f__Umbelopsidaceae	Umbelopsis
0,59%	0,47%	0,41%	0,30%	0,03%	0,03%	0,02%	0,04%	k__Fungi;p__Ascomycota;c__Sordariomycetes;o__Sordariales;f__Chaetomiaceae	unidentified
0,07%	0,05%	1,26%	0,11%	0,05%	0,04%	0,37%	0,08%	k__Fungi;p__Basidiomycota;c__Tremellomycetes;o__Trichosporonales;f__Trichosporonaceae	Apiotrichum
0,00%	0,02%	1,84%	0,01%	0,00%	0,00%	0,00%	0,00%	k__Fungi;p__Basidiomycota;c__Tremellomycetes;o__Trichosporonales;f__Trichosporonaceae	Vanrija
0,30%	0,06%	0,15%	0,40%	0,16%	0,07%	0,05%	0,29%	k__Fungi;p__Ascomycota;c__Eurotiomycetes;o__Eurotiales;f__Trichocomaceae	Talaromyces
0,10%	0,01%	0,12%	0,24%	0,22%	0,12%	0,48%	0,23%	k__Fungi;p__Ascomycota;c__Leotiomycetes;o__Helotiales;Other;Other	
0,02%	0,00%	0,00%	0,02%	0,48%	1,14%	0,02%	0,03%	k__Fungi;p__Ascomycota;c__Pezizomycetes;o__Pezizales;f__Helvellaceae	Helvella
0,00%	0,00%	0,10%	0,66%	0,03%	0,02%	0,02%	0,21%	k__Fungi;p__Ascomycota;c__Eurotiomycetes;o__Onygenales;f__Ajellomycetaceae	Histoplasma
0,00%	0,00%	0,00%	0,28%	0,30%	0,29%	0,14%	0,39%	k__Fungi;p__Ascomycota;c__Eurotiomycetes;o__Eurotiales;f__Thermoascaceae	Byssochlamys
0,00%	0,00%	0,00%	0,77%	0,01%	0,01%	0,09%	0,01%	k__Fungi;p__Ascomycota;c__Dothideomycetes;o__Pleosporales;f__Pleosporaceae	Alternaria
0,19%	0,03%	0,14%	0,34%	0,14%	0,21%	0,02%	0,16%	k__Fungi;p__Ascomycota;c__Eurotiomycetes;o__Chaetothyriales;f__Herpotrichiellaceae;Other	
0,41%	0,07%	0,06%	0,03%	0,02%	0,80%	0,01%	0,07%	k__Fungi;p__Basidiomycota;c__Agaricomycetes;o__Agaricales;f__Amanitaceae	Amanita
0,33%	0,41%	0,38%	0,17%	0,03%	0,00%	0,00%	0,01%	k__Fungi;p__Ascomycota;c__Sordariomycetes;o__Diaporthales;f__Schizoparmeaceae	Coniella
0,02%	0,08%	0,21%	0,15%	0,15%	0,15%	0,09%	0,11%	k__Fungi;p__Ascomycota;c__Dothideomycetes;o__Capnodiales;f__Cladosporiaceae	Cladosporium
0,01%	0,00%	0,02%	0,08%	0,16%	0,10%	0,25%	0,38%	k__Fungi;p__Basidiomycota;c__Agaricomycetes;o__Agaricales;f__Tricholomataceae	unidentified
0,00%	0,00%	0,01%	0,07%	0,24%	0,23%	0,14%	0,25%	k__Fungi;p__Basidiomycota;c__Agaricomycetes;o__Agaricales;f__Tricholomataceae	Mycena
0,02%	0,01%	0,01%	0,08%	0,08%	0,15%	0,04%	0,50%	k__Fungi;p__Basidiomycota;c__Agaricomycetes;o__Agaricales;f__Tricholomataceae	Mycenella
0,02%	0,02%	0,83%	0,02%	0,04%	0,01%	0,01%	0,01%	k__Fungi;p__Ascomycota;c__Sordariomycetes;o__Chaetosphaeriales;f__Chaetosphaeriaceae	Chloridium
0,15%	0,00%	0,10%	0,05%	0,24%	0,09%	0,14%	0,14%	k__Fungi;p__Ascomycota;c__Leotiomycetes;o__Helotiales;f__Helotiales_fam_Incertae_sedis	Cadophora
0,15%	0,11%	0,36%	0,07%	0,02%	0,06%	0,04%	0,05%	k__Fungi;p__Basidiomycota;c__Tremellomycetes;o__Trichosporonales;f__Trichosporonaceae	Cutaneotrichosporon
0,11%	0,17%	0,12%	0,09%	0,08%	0,09%	0,07%	0,11%	k__Fungi;p__Ascomycota;c__Sordariomycetes;o__unidentified;f__unidentified	unidentified
0,02%	0,00%	0,01%	0,25%	0,05%	0,03%	0,03%	0,21%	k__Fungi;p__Ascomycota;c__Leotiomycetes;o__Helotiales;f__Myxotrichaceae	unidentified
0,05%	0,01%	0,06%	0,14%	0,12%	0,10%	0,03%	0,11%	k__Fungi;Other;Other;Other;Other;Other	
0,00%	0,01%	0,00%	0,35%	0,02%	0,01%	0,01%	0,01%	k__Fungi;p__Basidiomycota;c__Agaricomycetes;o__Geastrales;f__Sclerogastraceae	Sclerogaster
0,06%	0,06%	0,10%	0,07%	0,02%	0,03%	0,02%	0,28%	k__Fungi;p__Ascomycota;c__Sordariomycetes;o__Sordariales;f__unidentified	unidentified
0,01%	0,01%	0,02%	0,10%	0,09%	0,02%	0,14%	0,22%	k__Fungi;p__Ascomycota;c__Dothideomycetes;o__Mytilinidales;f__Gloniaceae	Cenococcum
0,01%	0,00%	0,00%	0,01%	0,09%	0,50%	0,00%	0,07%	k__Fungi;p__Ascomycota;c__Eurotiomycetes;o__Onygenales;f__Onygenaceae	unidentified
0,18%	0,01%	0,01%	0,12%	0,02%	0,01%	0,00%	0,23%	k__Fungi;p__Basidiomycota;c__Geminibasidiomycetes;o__Geminibasidiales;f__Geminibasidiaceae	Geminibasidium
0,01%	0,01%	0,10%	0,04%	0,03%	0,05%	0,18%	0,21%	k__Fungi;p__Ascomycota;c__Pezizomycetes;o__Pezizales;f__Tuberaceae	Tuber
0,00%	0,00%	0,00%	0,01%	0,02%	0,01%	0,18%	0,40%	k__Fungi;p__Ascomycota;c__Pezizomycetes;o__Pezizales;f__Pyronemataceae	Otidea
0,02%	0,11%	0,05%	0,03%	0,00%	0,37%	0,00%	0,02%	k__Fungi;p__Basidiomycota;c__Agaricomycetes;o__Polyporales;f__unidentified	unidentified
0,20%	0,02%	0,16%	0,05%	0,03%	0,02%	0,02%	0,01%	k__Fungi;p__Ascomycota;c__Sordariomycetes;o__Ophiostomatales;f__Ophiostomataceae	Sporothrix
0,14%	0,00%	0,00%	0,00%	0,23%	0,08%	0,01%	0,08%	k__Fungi;p__Basidiomycota;c__Agaricomycetes;o__Cantharellales;f__Clavulinaceae	Clavulina
0,20%	0,03%	0,03%	0,03%	0,14%	0,04%	0,01%	0,03%	k__Fungi;p__Basidiomycota;c__Tremellomycetes;o__unidentified;f__unidentified	unidentified
0,00%	0,13%	0,01%	0,02%	0,01%	0,27%	0,00%	0,03%	k__Fungi;p__Ascomycota;c__Eurotiomycetes;o__Onygenales;Other;Other	
0,07%	0,08%	0,09%	0,06%	0,05%	0,04%	0,03%	0,02%	k__Fungi;p__Basidiomycota;c__Agaricomycetes;o__Gomphales;f__Gomphaceae	Clavariadelphus
0,00%	0,00%	0,00%	0,06%	0,13%	0,08%	0,07%	0,10%	k__Fungi;p__Ascomycota;c__Sordariomycetes;o__Microascales;f__Microascaceae	Pseudallescheria
0,00%	0,00%	0,00%	0,05%	0,12%	0,15%	0,06%	0,07%	k__Fungi;p__Basidiomycota;c__Agaricomycetes;o__Geastrales;f__Geastraceae	Geastrum

0,01%	0,00%	0,03%	0,11%	0,05%	0,12%	0,01%	0,06%	k__Fungi;p__Ascomycota;c__Sordariomycetes;o__Hypocreales;Other;Other	
0,00%	0,00%	0,00%	0,04%	0,12%	0,09%	0,08%	0,10%	k__Fungi;p__Basidiomycota;c__Agaricomycetes;o__Agaricales;f__Psathyrellaceae	unidentified
0,02%	0,00%	0,04%	0,09%	0,09%	0,04%	0,02%	0,09%	k__Fungi;p__Ascomycota;c__Sordariomycetes;o__Hypocreales;f__Hypocreales_fam_Incertae_sedis	Acremonium
0,01%	0,01%	0,00%	0,10%	0,01%	0,18%	0,01%	0,04%	k__Fungi;p__Ascomycota;c__Eurotiomycetes;o__Onygenales;f__Onygenaceae	Auxarthron
0,11%	0,02%	0,04%	0,06%	0,04%	0,08%	0,02%	0,04%	k__Fungi;p__Ascomycota;c__Sordariomycetes;o__Microascales;f__Microascaceae	Cephalotrichum
0,31%	0,00%	0,00%	0,00%	0,11%	0,00%	0,00%	0,00%	k__Fungi;p__Ascomycota;c__Dothideomycetes;o__Capnodiales;f__Capnodiaceae	Readerielliopsis
0,00%	0,00%	0,01%	0,13%	0,01%	0,00%	0,00%	0,15%	k__Fungi;p__Basidiomycota;c__Microbotryomycetes;o__Leucosporidiales;f__unidentified	unidentified
0,01%	0,00%	0,24%	0,01%	0,01%	0,14%	0,01%	0,01%	k__Fungi;p__Ascomycota;c__Sordariomycetes;o__Chaetosphaeriales;f__Chaetosphaeriaceae;Other	
0,02%	0,13%	0,05%	0,03%	0,04%	0,04%	0,03%	0,04%	k__Fungi;p__Ascomycota;c__Dothideomycetes;o__Pleosporales;f__unidentified	unidentified
0,05%	0,01%	0,04%	0,05%	0,05%	0,07%	0,01%	0,08%	k__Fungi;p__Ascomycota;c__Eurotiomycetes;o__Chaetothyriales;f__Herpotrichiellaceae	Cladophialophora
0,04%	0,00%	0,06%	0,04%	0,08%	0,06%	0,03%	0,05%	k__Fungi;p__Ascomycota;c__Sordariomycetes;o__Hypocreales;f__Nectriaceae	Neonectria
0,07%	0,06%	0,17%	0,02%	0,02%	0,01%	0,01%	0,02%	k__Fungi;p__Ascomycota;c__Sordariomycetes;o__Hypocreales;f__Nectriaceae	unidentified
0,27%	0,05%	0,05%	0,01%	0,00%	0,00%	0,00%	0,00%	k__Fungi;p__Basidiomycota;c__Tremellomycetes;o__Tremellales;f__unidentified	unidentified
0,01%	0,03%	0,00%	0,14%	0,01%	0,01%	0,00%	0,04%	k__Fungi;p__Ascomycota;c__Eurotiomycetes;o__Onygenales;f__Onygenales_fam_Incertae_sedis	Spiromastix
0,04%	0,03%	0,01%	0,04%	0,08%	0,04%	0,03%	0,05%	k__Fungi;p__Basidiomycota;c__Agaricomycetes;o__Agaricales;f__Lycoperdaceae	Lycoperdon
0,01%	0,01%	0,07%	0,11%	0,01%	0,03%	0,01%	0,02%	k__Fungi;p__Basidiomycota;c__Agaricomycetes;o__Tremellodendropsidales;f__unidentified	unidentified
0,02%	0,00%	0,02%	0,03%	0,12%	0,05%	0,06%	0,06%	k__Fungi;p__Ascomycota;c__Sordariomycetes;o__Myrmecridiales;f__unidentified	unidentified
0,02%	0,01%	0,15%	0,02%	0,08%	0,02%	0,03%	0,02%	k__Fungi;p__Ascomycota;c__Sordariomycetes;Other;Other;Other	
0,01%	0,01%	0,08%	0,09%	0,02%	0,01%	0,04%	0,04%	k__Fungi;p__Ascomycota;c__Leotiomycetes;o__Thelebolales;f__Pseudeurotiaceae	Pseudogymnoascus
0,02%	0,00%	0,02%	0,05%	0,06%	0,06%	0,03%	0,06%	k__Fungi;p__Ascomycota;c__Eurotiomycetes;o__Chaetothyriales;f__unidentified	unidentified
0,00%	0,00%	0,01%	0,06%	0,06%	0,04%	0,08%	0,05%	k__Fungi;p__Basidiomycota;c__Agaricomycetes;o__Agaricales;f__Hymenogastraceae	Hymenogaster
0,06%	0,00%	0,01%	0,08%	0,07%	0,02%	0,02%	0,03%	k__Fungi;p__Ascomycota;c__Dothideomycetes;o__Capnodiales;f__unidentified	unidentified
0,03%	0,01%	0,01%	0,03%	0,09%	0,05%	0,06%	0,04%	k__Fungi;p__Ascomycota;c__unidentified;o__unidentified;f__unidentified	unidentified
0,04%	0,04%	0,01%	0,06%	0,03%	0,03%	0,01%	0,06%	k__Fungi;p__Ascomycota;c__Sordariomycetes;o__Xylariales;f__Xylariaceae	Ascotricha
0,00%	0,01%	0,24%	0,01%	0,02%	0,01%	0,01%	0,01%	k__Fungi;p__Ascomycota;c__Sordariomycetes;o__Hypocreales;f__Cordycipitaceae	Lecanicillium
0,10%	0,03%	0,01%	0,01%	0,06%	0,03%	0,00%	0,05%	k__Fungi;p__Ascomycota;c__Eurotiomycetes;o__Eurotiales;f__unidentified	unidentified
0,00%	0,00%	0,00%	0,03%	0,07%	0,06%	0,04%	0,07%	k__Fungi;p__Basidiomycota;c__Agaricomycetes;o__Boletales;f__Boletaceae	Boletus
0,03%	0,00%	0,00%	0,10%	0,05%	0,01%	0,00%	0,02%	k__Fungi;p__Basidiomycota;c__Agaricomycetes;o__Boletales;f__Melanogastraceae	Melanogaster
0,27%	0,01%	0,00%	0,00%	0,00%	0,00%	0,00%	0,00%	k__Fungi;p__Chytridiomycota;c__Rhizophydiomycetes;o__Rhizophydiales;f__unidentified	unidentified
0,02%	0,01%	0,03%	0,06%	0,06%	0,01%	0,00%	0,05%	k__Fungi;p__Ascomycota;c__Eurotiomycetes;o__Eurotiales;f__Aspergillaceae;Other	
0,00%	0,00%	0,00%	0,03%	0,05%	0,05%	0,03%	0,10%	k__Fungi;p__Ascomycota;c__Saccharomycetes;o__Saccharomycetales;f__Dipodascaceae	Geotrichum
0,06%	0,05%	0,10%	0,03%	0,00%	0,00%	0,00%	0,00%	k__Fungi;p__Ascomycota;c__Sordariomycetes;o__Hypocreales;f__Ophiocordycipitaceae	Ophiocordyceps
0,03%	0,00%	0,00%	0,04%	0,01%	0,00%	0,00%	0,15%	k__Fungi;p__Mucoromycota;c__Mucoromycotina_cls_Incertae_sedis;o__Mucoromycotina_ord_Incertae_sedis;f__Mucoromycotina_fam_Incertae_sedis	Bifiguratus
0,00%	0,00%	0,00%	0,01%	0,13%	0,05%	0,03%	0,03%	k__Fungi;p__Ascomycota;c__Dothideomycetes;o__Dothideales;f__Aureobasidiaceae	Aureobasidium
0,01%	0,00%	0,18%	0,02%	0,01%	0,01%	0,01%	0,01%	k__Fungi;p__Ascomycota;c__Sordariomycetes;o__Chaetosphaeriales;f__Chaetosphaeriaceae	Chaetosphaeria
0,01%	0,01%	0,01%	0,03%	0,04%	0,07%	0,01%	0,03%	k__Fungi;p__Ascomycota;c__Dothideomycetes;o__Pleosporales;f__Didymellaceae;Other	
0,03%	0,00%	0,00%	0,06%	0,02%	0,01%	0,00%	0,06%	k__Fungi;p__Ascomycota;c__Sordariomycetes;o__Hypocreales;f__Clavicipitaceae	Metapochonia
0,10%	0,00%	0,00%	0,00%	0,03%	0,09%	0,01%	0,01%	k__Fungi;p__Basidiomycota;c__Agaricomycetes;o__Thelephorales;f__Thelephoraceae;Other	
0,01%	0,00%	0,02%	0,02%	0,02%	0,10%	0,00%	0,05%	k__Fungi;p__Ascomycota;c__Sordariomycetes;o__Sordariales;f__Lasiosphaeriaceae	unidentified
0,01%	0,00%	0,01%	0,02%	0,06%	0,02%	0,06%	0,05%	k__Fungi;p__Ascomycota;c__Sordariomycetes;o__Hypocreales;f__Cordycipitaceae	Beauveria
0,00%	0,00%	0,02%	0,01%	0,01%	0,01%	0,16%	0,02%	k__Fungi;p__Ascomycota;c__Pezizomycetes;o__Pezizales;f__Pyronemataceae	Humaria
0,00%	0,00%	0,01%	0,09%	0,00%	0,00%	0,02%	0,01%	k__Fungi;p__Ascomycota;c__Sordariomycetes;o__Sordariales;f__Cephalothecaceae	Phialemonium
0,00%	0,02%	0,01%	0,03%	0,05%	0,05%	0,02%	0,04%	k__Fungi;p__Ascomycota;c__Sordariomycetes;o__Hypocreales;f__Nectriaceae	Fusarium
0,02%	0,02%	0,03%	0,02%	0,04%	0,03%	0,02%	0,03%	k__Fungi;p__Ascomycota;c__Sordariomycetes;o__Hypocreales;f__Nectriaceae	Ilyonectria
0,00%	0,00%	0,01%	0,03%	0,03%	0,07%	0,01%	0,05%	k__Fungi;p__Ascomycota;c__Leotiomycetes;o__Helotiales;f__Helotiaceae	Meliniomyces
0,02%	0,01%	0,04%	0,03%	0,04%	0,03%	0,01%	0,03%	k__Fungi;p__Ascomycota;c__Sordariomycetes;o__Hypocreales;f__Nectriaceae;Other	
0,06%	0,01%	0,03%	0,03%	0,04%	0,00%	0,01%	0,01%	k__Fungi;p__Ascomycota;c__Pezizomycotina_cls_Incertae_sedis;o__Pezizomycotina_ord_Incertae_sedis;f__Pezizomycotina_fam_Incertae_sedis	Ciliophora
0,01%	0,03%	0,01%	0,03%	0,01%	0,08%	0,01%	0,02%	k__Fungi;p__Ascomycota;c__Dothideomycetes;o__Pleosporales;Other;Other	
0,02%	0,01%	0,00%	0,06%	0,00%	0,00%	0,00%	0,07%	k__Fungi;p__Mucoromycota;Other;Other;Other;Other	
0,00%	0,00%	0,00%	0,02%	0,05%	0,04%	0,03%	0,06%	k__Fungi;p__Basidiomycota;c__Agaricomycetes;o__Boletales;f__Boletaceae	Neoboletus
0,03%	0,03%	0,04%	0,02%	0,02%	0,02%	0,01%	0,02%	k__Fungi;p__Ascomycota;c__Sordariomycetes;o__Sordariales;Other;Other	
0,01%	0,00%	0,00%	0,00%	0,10%	0,08%	0,00%	0,01%	k__Fungi;p__Basidiomycota;c__Agaricomycetes;o__Thelephorales;f__Thelephoraceae	Thelephora
0,00%	0,01%	0,19%	0,00%	0,00%	0,00%	0,00%	0,00%	k__Fungi;p__Basidiomycota;c__Tremellomycetes;o__Trichosporonales;f__Trichosporonaceae	Effuseotrichosporon
0,00%	0,00%	0,00%	0,03%	0,03%	0,04%	0,01%	0,07%	k__Fungi;p__Ascomycota;c__Saccharomycetes;o__Saccharomycetales;f__Pichiaceae	Pichia
0,00%	0,00%	0,00%	0,10%	0,00%	0,00%	0,00%	0,00%	k__Fungi;p__Basidiomycota;c__Agaricomycetes;o__Hymenochaetales;f__Hymenochaetaceae	Hymenochaete
0,00%	0,00%	0,00%	0,03%	0,04%	0,04%	0,02%	0,03%	k__Fungi;p__Ascomycota;c__Sordariomycetes;o__Coniochaetales;f__Coniochaetaceae	Lecythophora
0,04%	0,02%	0,07%	0,02%	0,00%	0,00%	0,00%	0,00%	k__Fungi;p__Ascomycota;c__Eurotiomycetes;o__Eurotiales;f__Trichocomaceae	Rasamsonia
0,04%	0,00%	0,03%	0,00%	0,10%	0,00%	0,00%	0,00%	k__Fungi;p__Basidiomycota;c__Agaricomycetes;o__Boletales;f__Sclerodermataceae	Scleroderma
0,00%	0,00%	0,00%	0,09%	0,00%	0,00%	0,00%	0,00%	k__Fungi;p__Ascomycota;c__Leotiomycetes;o__Helotiales;f__Helotiaceae	unidentified
0,03%	0,02%	0,02%	0,01%	0,00%	0,09%	0,00%	0,00%	k__Fungi;p__Basidiomycota;c__Agaricomycetes;o__Trechisporales;f__unidentified	unidentified
0,00%	0,00%	0,00%	0,03%	0,04%	0,03%	0,02%	0,03%	k__Fungi;p__Ascomycota;c__Sordariomycetes;o__Calosphaeriales;f__Pleurostomataceae	Pleurostoma
0,00%	0,00%	0,00%	0,07%	0,00%	0,01%	0,00%	0,02%	k__Fungi;p__Ascomycota;c__Eurotiomycetes;o__Onygenales;f__Onygenales_fam_Incertae_sedis;Other	
0,02%	0,03%	0,04%	0,02%	0,01%	0,01%	0,01%	0,01%	k__Fungi;p__Basidiomycota;c__Agaricomycetes;o__Agaricales;f__Tricholomataceae	Macrocystidia
0,04%	0,03%	0,05%	0,02%	0,00%	0,00%	0,00%	0,00%	k__Fungi;p__Ascomycota;c__Lecanoromycetes;o__Peltigerales;f__Collemataceae	Leptogium
0,01%	0,00%	0,14%	0,00%	0,00%	0,00%	0,00%	0,00%	k__Fungi;p__Rozellomycota;c__Rozellomycotina_cls_Incertae_sedis;o__GS11;f__unidentified	unidentified
0,00%	0,01%	0,00%	0,01%	0,04%	0,04%	0,01%	0,03%	k__Fungi;p__Basidiomycota;c__Agaricomycetes;o__Sebacinales;f__Sebacinaceae	Helvellosebacina
0,00%	0,00%	0,00%	0,04%	0,02%	0,02%	0,01%	0,03%	k__Fungi;p__Ascomycota;c__Eurotiomycetes;o__Eurotiales;f__Aspergillaceae	Monascus
0,11%	0,02%	0,01%	0,01%	0,00%	0,00%	0,00%	0,00%	k__Fungi;p__Chytridiomycota;c__Rhizophydiomycetes;o__Rhizophydiales;f__Rhizophydiales_fam_Incertae_sedis	Operculomyces
0,00%	0,00%	0,00%	0,01%	0,02%	0,01%	0,01%	0,09%	k__Fungi;p__Basidiomycota;c__Agaricomycetes;o__Agaricales;f__Omphalotaceae	Rhodocollybia
0,00%	0,00%	0,00%	0,03%	0,03%	0,02%	0,02%	0,02%	k__Fungi;p__Ascomycota;c__Leotiomycetes;o__Helotiales;f__Helotiaceae	Helicodendron
0,02%	0,03%	0,01%	0,01%	0,01%	0,05%	0,00%	0,01%	k__Fungi;p__Ascomycota;c__Sordariomycetes;o__Sordariales;f__Lasiosphaeriaceae	Apodus
0,00%	0,01%	0,00%	0,06%	0,00%	0,00%	0,00%	0,01%	k__Fungi;p__Basidiomycota;c__Agaricomycetes;o__Agaricales;f__Tricholomataceae	Ripartites
0,00%	0,00%	0,00%	0,02%	0,02%	0,03%	0,02%	0,04%	k__Fungi;p__Ascomycota;c__Saccharomycetes;o__Saccharomycetales;f__Saccharomycetaceae	Williopsis
0,01%	0,01%	0,00%	0,02%	0,03%	0,03%	0,02%	0,02%	k__Fungi;p__Ascomycota;c__Sordariomycetes;o__Sordariales;f__Sordariales_fam_Incertae_sedis	Ramophialophora
0,01%	0,02%	0,03%	0,01%	0,02%	0,01%	0,01%	0,01%	k__Fungi;p__Basidiomycota;c__Agaricomycetes;o__Agaricales;Other;Other	
0,00%	0,00%	0,00%	0,01%	0,03%	0,03%	0,02%	0,03%	k__Fungi;p__Basidiomycota;c__Agaricomycetes;o__Hysterangiales;Other;Other	
0,01%	0,01%	0,01%	0,02%	0,01%	0,03%	0,01%	0,04%	k__Fungi;p__Basidiomycota;Other;Other;Other;Other	
0,05%	0,00%	0,00%	0,01%	0,02%	0,01%	0,01%	0,02%	k__Fungi;p__Ascomycota;c__Dothideomycetes;o__Venturiales;f__Venturiaceae	Venturia
0,00%	0,00%	0,00%	0,02%	0,02%	0,02%	0,02%	0,03%	k__Fungi;p__Ascomycota;c__Leotiomycetes;o__Helotiales;f__Dermateaceae;Other	
0,02%	0,02%	0,00%	0,01%	0,02%	0,04%	0,01%	0,00%	k__Fungi;p__Ascomycota;c__Dothideomycetes;o__Dothideomycetes_ord_Incertae_sedis;f__Eremomycetaceae	Eremomyces
0,00%	0,00%	0,13%	0,00%	0,00%	0,00%	0,00%	0,00%	k__Fungi;p__Basidiomycota;c__Tremellomycetes;o__Trichosporonales;f__Trichosporonaceae;Other	
0,00%	0,00%	0,00%	0,01%	0,03%	0,02%	0,02%	0,04%	k__Fungi;p__Ascomycota;c__Saccharomycetes;o__Saccharomycetales;f__Dipodascaceae	unidentified
0,04%	0,01%	0,04%	0,02%	0,00%	0,00%	0,00%	0,01%	k__Fungi;p__Ascomycota;c__Eurotiomycetes;o__Eurotiales;Other;Other	
0,00%	0,00%	0,00%	0,03%	0,02%	0,00%	0,02%	0,04%	k__Fungi;p__Ascomycota;c__Leotiomycetes;o__Helotiales;f__Dermateaceae	Cryptosporiopsis
0,01%	0,00%	0,01%	0,02%	0,02%	0,01%	0,01%	0,03%	k__Fungi;p__Ascomycota;c__Leotiomycetes;o__Helotiales;f__Helotiales_fam_Incertae_sedis	Chalara
0,00%	0,00%	0,01%	0,04%	0,01%	0,00%	0,01%	0,03%	k__Fungi;p__Ascomycota;c__Leotiomycetes;o__Helotiales;f__Hyaloscyphaceae	unidentified
0,01%	0,01%	0,01%	0,02%	0,02%	0,01%	0,01%	0,03%	k__Fungi;p__Ascomycota;c__Dothideomycetes;o__Venturiales;f__Venturiaceae	unidentified
0,00%	0,00%	0,00%	0,01%	0,02%	0,03%	0,02%	0,03%	k__Fungi;p__Basidiomycota;c__Agaricomycetes;o__Auriculariales;f__Hyaloriaceae	Protodontia
0,01%	0,00%	0,04%	0,02%	0,01%	0,01%	0,01%	0,01%	k__Fungi;p__Ascomycota;c__Leotiomycetes;o__Thelebolales;f__Pseudeurotiaceae	Pseudeurotium
0,00%	0,00%	0,00%	0,05%	0,00%	0,00%	0,01%	0,00%	k__Fungi;p__Ascomycota;c__Leotiomycetes;o__Helotiales;f__Helotiaceae;Other	
0,03%	0,02%	0,01%	0,02%	0,01%	0,01%	0,00%	0,00%	k__Fungi;p__Ascomycota;c__Sordariomycetes;o__Hypocreales;f__Bionectriaceae	Clonostachys
0,00%	0,00%	0,01%	0,03%	0,01%	0,00%	0,02%	0,02%	k__Fungi;p__Basidiomycota;c__Cystobasidiomycetes;o__Erythrobasidiales;f__Erythrobasidiales_fam_Inc	Sakaguchia

								ertae_sedis	
0,01%	0,09%	0,00%	0,00%	0,00%	0,01%	0,00%	0,00%	k__Fungi;p__Basidiomycota;c__Agaricomycetes;o__Agaricales;f__Clavariaceae	Clavaria
0,00%	0,00%	0,00%	0,04%	0,00%	0,00%	0,00%	0,02%	k__Fungi;p__Ascomycota;c__Sordariomycetes;o__Sordariales;f__Sordariales_fam_Incertae_sedis	Cordana
0,00%	0,00%	0,00%	0,01%	0,03%	0,03%	0,02%	0,02%	k__Fungi;p__Basidiomycota;c__Agaricomycetes;o__Agaricales;f__Tricholomataceae	Pseudoclitocybe
0,00%	0,00%	0,05%	0,01%	0,02%	0,01%	0,02%	0,00%	k__Fungi;p__Basidiomycota;c__Agaricomycetes;o__Agaricales;f__Tricholomataceae	Paralepista
0,03%	0,01%	0,05%	0,01%	0,00%	0,00%	0,00%	0,01%	k__Fungi;p__Rozellomycota;c__unidentified;o__unidentified;f__unidentified	unidentified
0,02%	0,00%	0,03%	0,01%	0,02%	0,01%	0,01%	0,02%	k__Fungi;p__Ascomycota;c__Eurotiomycetes;o__Onygenales;f__Onygenales_fam_Incertae_sedis	Chrysosporium
0,00%	0,00%	0,00%	0,01%	0,01%	0,06%	0,01%	0,01%	k__Fungi;p__Ascomycota;c__Dothideomycetes;o__Pleosporales;f__Pleomassariaceae	Prosthemium
0,02%	0,00%	0,00%	0,01%	0,03%	0,02%	0,01%	0,01%	k__Fungi;p__Basidiomycota;c__Agaricomycetes;o__Thelephorales;f__Thelephoraceae	Odontia
0,03%	0,00%	0,02%	0,01%	0,00%	0,00%	0,01%	0,01%	k__Fungi;p__Ascomycota;c__Leotiomycetes;o__Helotiales;f__Myxotrichaceae;Other	
0,02%	0,00%	0,05%	0,01%	0,00%	0,00%	0,00%	0,00%	k__Fungi;p__Basidiomycota;c__Tremellomycetes;o__Tremellales;f__Carcinomycetaceae	Carcinomyces
0,04%	0,00%	0,02%	0,01%	0,01%	0,00%	0,00%	0,02%	k__Fungi;p__Mortierellomycota;c__Mortierellomycetes;o__Mortierellales;f__Mortierellaceae	unidentified
0,00%	0,00%	0,00%	0,00%	0,00%	0,09%	0,00%	0,00%	k__Fungi;p__Ascomycota;c__Sordariomycetes;o__Chaetosphaeriales;f__Chaetosphaeriaceae	Menispora
0,01%	0,00%	0,00%	0,01%	0,02%	0,01%	0,01%	0,02%	k__Fungi;p__Basidiomycota;c__Agaricomycetes;o__Agaricales;f__Pluteaceae	Pluteus
0,02%	0,01%	0,01%	0,01%	0,02%	0,00%	0,00%	0,01%	k__Fungi;p__Chytridiomycota;c__unidentified;o__unidentified;f__unidentified	unidentified
0,00%	0,00%	0,00%	0,02%	0,02%	0,01%	0,01%	0,02%	k__Fungi;p__Ascomycota;c__Sordariomycetes;o__Hypocreales;f__Nectriaceae	Volutella
0,00%	0,00%	0,00%	0,01%	0,02%	0,02%	0,01%	0,02%	k__Fungi;p__Ascomycota;c__Pezizomycetes;o__Pezizales;f__Sarcoscyphaceae	Desmazierella
0,00%	0,00%	0,00%	0,02%	0,03%	0,02%	0,01%	0,01%	k__Fungi;p__Ascomycota;c__Dothideomycetes;o__Pleosporales;f__Sporormiaceae	Westerdykella
0,01%	0,00%	0,01%	0,02%	0,00%	0,02%	0,00%	0,00%	k__Fungi;p__Ascomycota;c__Eurotiomycetes;o__Chaetothyriales;f__Herpotrichiellaceae	unidentified
0,00%	0,00%	0,08%	0,00%	0,00%	0,00%	0,00%	0,01%	k__Fungi;p__Ascomycota;c__Sordariomycetes;o__Glomerellales;f__Plectosphaerellaceae	Verticillium
0,00%	0,00%	0,00%	0,01%	0,00%	0,06%	0,00%	0,01%	k__Fungi;p__Ascomycota;c__Leotiomycetes;o__Helotiales;f__Myxotrichaceae	Myxotrichum
0,00%	0,02%	0,00%	0,01%	0,01%	0,02%	0,01%	0,01%	k__Fungi;p__Ascomycota;c__Leotiomycetes;o__Thelebolales;f__Thelebolaceae	Thelebolus
0,01%	0,01%	0,06%	0,00%	0,00%	0,01%	0,00%	0,01%	k__Fungi;p__Basidiomycota;c__Agaricomycetes;o__Sebacinales;Other;Other	
0,00%	0,00%	0,03%	0,00%	0,03%	0,01%	0,01%	0,01%	k__Fungi;p__Basidiomycota;c__Agaricomycetes;o__Agaricales;f__Hydnangiaceae	Laccaria
0,00%	0,01%	0,00%	0,01%	0,03%	0,01%	0,01%	0,03%	k__Fungi;p__Basidiomycota;c__Microbotryomycetes;o__Sporidiobolales;f__Sporidiobolaceae	Rhodotorula
0,00%	0,00%	0,00%	0,01%	0,01%	0,03%	0,01%	0,01%	k__Fungi;p__Ascomycota;c__Sordariomycetes;o__Chaetosphaeriales;Other;Other	
0,00%	0,02%	0,01%	0,01%	0,01%	0,01%	0,00%	0,01%	k__Fungi;p__Ascomycota;c__Dothideomycetes;Other;Other;Other	
0,00%	0,00%	0,00%	0,01%	0,02%	0,02%	0,01%	0,02%	k__Fungi;p__Ascomycota;c__Pezizomycetes;o__Pezizales;f__Pyronemataceae	Wilcoxina
0,01%	0,00%	0,00%	0,01%	0,03%	0,02%	0,01%	0,01%	k__Fungi;p__Ascomycota;c__Sordariomycetes;o__Sordariales;f__Lasiosphaeriaceae;Other	
0,00%	0,01%	0,00%	0,00%	0,01%	0,04%	0,01%	0,01%	k__Fungi;p__Basidiomycota;c__Tremellomycetes;o__Holtermanniales;f__Holtermanniales_fam_Incertae_sedis	Holtermanniella
0,00%	0,00%	0,00%	0,01%	0,00%	0,00%	0,00%	0,06%	k__Fungi;p__Basidiomycota;c__Agaricomycetes;o__Cantharellales;f__Cantharellaceae	Cantharellus
0,03%	0,00%	0,01%	0,00%	0,01%	0,01%	0,00%	0,00%	k__Fungi;p__Chytridiomycota;Other;Other;Other;Other	
0,01%	0,00%	0,01%	0,01%	0,01%	0,01%	0,01%	0,01%	k__Fungi;p__Ascomycota;c__Leotiomycetes;o__unidentified;f__unidentified	unidentified
0,00%	0,00%	0,00%	0,03%	0,00%	0,00%	0,00%	0,03%	k__Fungi;p__Ascomycota;c__Leotiomycetes;o__Thelebolales;f__Pseudeurotiaceae	Leuconeurospora

(C)

AI1c	AI2c	AI3c	AI4c	AII1c	AII2c	AII3c	AII4c	taxonomy	
10,34%	0,57%	66,47%	31,98%	4,30%	3,14%	28,74%	8,00%	k__Fungi;p__Basidiomycota;c__Agaricomycetes;o__Russulales;f__Russulaceae	Russula
1,52%	61,18%	2,35%	1,68%	0,12%	14,53%	0,15%	0,21%	k__Fungi;p__Basidiomycota;c__Agaricomycetes;o__Trechisporales;f__Hydnodontaceae	Trechispora
0,28%	5,25%	0,27%	0,16%	63,02%	24,77%	0,25%	0,18%	k__Fungi;p__Basidiomycota;c__Agaricomycetes;o__Agaricales;f__Tricholomataceae	Tricholoma
25,05%	0,74%	0,25%	0,22%	0,37%	1,06%	0,65%	43,49%	k__Fungi;p__Basidiomycota;c__Agaricomycetes;o__Agaricales;f__Cortinariaceae	Cortinarius
0,00%	22,20%	0,00%	0,00%	0,08%	16,78%	0,09%	0,09%	k__Fungi;p__Basidiomycota;c__Agaricomycetes;o__Trechisporales;Other;Other	Trechisporales_Other
0,06%	0,02%	0,10%	21,40%	0,01%	0,03%	0,03%	8,00%	k__Fungi;p__Ascomycota;c__Sordariomycetes;o__Sordariales;f__unidentified	Sordariales_unidentified
6,85%	0,61%	3,19%	2,09%	3,38%	3,10%	3,72%	4,13%	k__Fungi;p__Ascomycota;c__Eurotiomycetes;o__Eurotiales;f__Aspergillaceae	Penicillium
5,67%	0,18%	0,24%	0,59%	6,93%	0,24%	10,35%	1,90%	k__Fungi;p__Basidiomycota;c__Agaricomycetes;o__Sebacinales;f__Sebacinaceae	Sebacina
3,76%	0,15%	0,15%	0,11%	0,50%	14,96%	3,33%	0,35%	k__Fungi;p__Basidiomycota;c__Agaricomycetes;o__Thelephorales;f__Thelephoraceae	Thelephoraceae_unidentified
2,05%	0,34%	0,71%	6,67%	0,98%	2,59%	0,90%	7,62%	k__Fungi;p__Ascomycota;c__Leotiomycetes;o__Helotiales;f__Myxotrichaceae	Oidiodendron
5,10%	0,10%	0,49%	1,11%	2,08%	0,16%	0,65%	3,32%	k__Fungi;p__Mortierellomycota;c__Mortierellomycetes;o__Mortierellales;f__Mortierellaceae	Mortierella
0,01%	0,02%	0,00%	0,04%	0,17%	0,09%	12,03%	0,07%	k__Fungi;p__Basidiomycota;c__Agaricomycetes;o__Agaricales;f__Tricholomataceae	Tricholomataceae_unidentified
0,00%	0,05%	0,00%	0,00%	0,72%	0,12%	10,63%	0,16%	k__Fungi;p__Basidiomycota;c__Agaricomycetes;o__Agaricales;f__Agaricaceae	Agaricus
4,66%	0,65%	1,19%	0,18%	0,93%	1,91%	0,62%	0,08%	k__Fungi;p__Basidiomycota;c__Tremellomycetes;o__Filobasidiales;f__Piskurozymaceae	Solicoccozyma
0,42%	0,39%	0,73%	2,71%	0,57%	1,43%	1,98%	2,09%	k__Fungi;p__Ascomycota;Other;Other;Other;Other	Ascomycota_Other
0,77%	0,31%	0,79%	4,37%	0,67%	0,96%	0,74%	1,39%	k__Fungi;p__unidentified;c__unidentified;o__unidentified;f__unidentified	Fungi_unidentified
8,91%	0,11%	0,11%	0,05%	0,07%	0,19%	0,01%	0,32%	k__Fungi;p__Basidiomycota;c__Agaricomycetes;o__Agaricales;f__Amanitaceae	Amanita
0,19%	0,04%	0,31%	7,25%	0,22%	0,18%	0,53%	0,29%	k__Fungi;p__Ascomycota;c__Sordariomycetes;o__Hypocreales;f__Clavicipitaceae	Metarhizium
4,05%	0,32%	0,10%	1,31%	0,85%	0,15%	0,25%	0,41%	k__Fungi;p__Ascomycota;c__Sordariomycetes;o__Hypocreales;f__unidentified	unidentified
0,64%	0,12%	0,84%	2,69%	0,85%	0,41%	0,63%	1,30%	k__Fungi;p__Ascomycota;c__Sordariomycetes;o__Hypocreales;f__Hypocreaceae	Trichoderma
0,33%	0,07%	0,74%	1,37%	0,24%	0,08%	4,02%	0,42%	k__Fungi;p__Basidiomycota;c__Agaricomycetes;o__Agaricales;f__Hymenogastraceae	Hebeloma
2,77%	0,07%	0,46%	0,64%	0,80%	0,47%	0,74%	0,66%	k__Fungi;p__Basidiomycota;c__Tremellomycetes;o__Tremellales;f__Trimorphomycetaceae	Saitozyma
0,12%	0,11%	0,03%	1,42%	2,00%	1,60%	0,19%	0,58%	k__Fungi;p__Basidiomycota;c__Agaricomycetes;o__Agaricales;f__Inocybaceae	Inocybe
1,91%	0,17%	1,78%	0,39%	0,31%	0,29%	0,26%	0,14%	k__Fungi;p__Ascomycota;c__Sordariomycetes;o__Sordariales;f__Chaetomiaceae;Other	Chaetomiaceae_Other
0,08%	0,02%	0,09%	3,52%	0,02%	0,04%	0,04%	1,19%	k__Fungi;p__Ascomycota;c__Saccharomycetes;o__Saccharomycetales;f__unidentified	unidentified
0,24%	0,07%	3,48%	0,32%	0,10%	0,05%	0,59%	0,07%	k__Fungi;p__Ascomycota;c__Leotiomycetes;o__Thelebolales;f__Pseudeurotiaceae;Other	
0,06%	2,08%	0,09%	0,06%	0,00%	0,30%	0,00%	0,00%	k__Fungi;p__Basidiomycota;c__Agaricomycetes;o__Polyporales;f__unidentified	unidentified
0,91%	0,04%	0,58%	0,51%	0,41%	0,28%	0,84%	0,87%	k__Fungi;p__Ascomycota;c__Eurotiomycetes;o__Chaetothyriales;f__Herpotrichiellaceae	Exophiala
1,62%	0,12%	1,40%	0,30%	0,24%	0,20%	0,20%	0,10%	k__Fungi;p__Ascomycota;c__Sordariomycetes;o__Sordariales;f__Chaetomiaceae	Humicola
2,62%	0,01%	0,02%	0,02%	0,93%	0,01%	0,01%	0,01%	k__Fungi;p__Basidiomycota;c__Agaricomycetes;o__Cantharellales;f__Clavulinaceae	Membranomyces
0,15%	0,06%	1,61%	0,21%	0,14%	0,07%	0,90%	0,13%	k__Fungi;p__Ascomycota;c__Leotiomycetes;o__Thelebolales;f__Pseudeurotiaceae	Geomyces
0,07%	0,04%	0,01%	0,01%	0,12%	2,36%	0,28%	0,09%	k__Fungi;p__Basidiomycota;c__Agaricomycetes;o__Thelephorales;f__Thelephoraceae	Tomentella
0,20%	0,05%	0,24%	0,07%	0,26%	0,70%	0,69%	0,29%	k__Fungi;p__Ascomycota;c__Eurotiomycetes;o__Eurotiales;f__Trichocomaceae	Sagenomella
0,21%	0,01%	0,04%	0,78%	0,88%	0,06%	0,31%	0,27%	k__Fungi;p__Ascomycota;c__Dothideomycetes;o__Mytilinidales;f__Gloniaceae	Cenococcum
0,40%	0,19%	0,83%	0,52%	0,09%	0,12%	0,07%	0,08%	k__Fungi;p__Basidiomycota;c__Agaricomycetes;o__Russulales;f__Lachnocladiaceae	Vararia
0,30%	0,01%	0,01%	0,19%	0,04%	0,07%	0,07%	1,65%	k__Fungi;p__Ascomycota;c__Leotiomycetes;o__Helotiales;f__Myxotrichaceae	unidentified
0,00%	0,01%	0,01%	0,00%	0,01%	0,01%	1,96%	0,29%	k__Fungi;p__Ascomycota;c__Pezizomycetes;o__Pezizales;f__Pyronemataceae	Otidea
0,07%	0,04%	0,09%	0,06%	0,29%	0,22%	0,59%	0,41%	k__Fungi;p__Ascomycota;c__Leotiomycetes;o__Helotiales;f__unidentified	unidentified
0,04%	0,08%	0,36%	0,12%	0,02%	0,01%	1,02%	0,05%	k__Fungi;p__Ascomycota;c__Pezizomycetes;o__Pezizales;f__Tuberaceae	Tuber
0,15%	0,03%	0,16%	0,09%	0,08%	0,01%	1,14%	0,01%	k__Fungi;p__Basidiomycota;c__Agaricomycetes;o__Thelephorales;f__Thelephoraceae	Pseudotomentella
0,00%	0,00%	0,00%	0,00%	0,00%	0,00%	0,00%	1,68%	k__Fungi;p__Basidiomycota;c__Agaricomycetes;o__Cantharellales;f__Cantharellaceae	Cantharellus
0,27%	0,01%	0,04%	0,59%	0,26%	0,07%	0,04%	0,21%	k__Fungi;p__Mucoromycota;c__Umbelopsidomycetes;o__Umbelopsidales;f__Umbelopsidaceae	Umbelopsis
0,30%	0,08%	0,47%	0,43%	0,01%	0,00%	0,00%	0,01%	k__Fungi;p__Ascomycota;c__Sordariomycetes;o__Diaporthales;f__Schizoparmaceae	Coniella
0,02%	0,09%	0,22%	0,01%	0,20%	0,20%	0,26%	0,28%	k__Fungi;p__Ascomycota;c__Sordariomycetes;o__Coniochaetales;f__Coniochaetaceae	Coniochaeta
0,04%	0,04%	0,00%	0,00%	0,14%	0,16%	0,54%	0,14%	k__Fungi;p__Basidiomycota;c__Agaricomycetes;o__Agaricales;f__Tricholomataceae	Mycena
0,00%	0,06%	0,00%	0,00%	0,21%	0,19%	0,30%	0,26%	k__Fungi;p__Ascomycota;c__Eurotiomycetes;o__Eurotiales;f__Thermoascaceae	Byssochlamys
0,01%	0,47%	0,02%	0,01%	0,00%	0,08%	0,00%	0,00%	k__Fungi;p__Basidiomycota;c__Agaricomycetes;o__Trechisporales;f__unidentified	unidentified
0,31%	0,03%	0,32%	0,04%	0,07%	0,11%	0,10%	0,05%	k__Fungi;p__Ascomycota;c__Eurotiomycetes;o__Eurotiales;f__Aspergillaceae	Aspergillus
0,03%	0,05%	0,02%	0,01%	0,24%	0,29%	0,17%	0,11%	k__Fungi;p__Ascomycota;c__Dothideomycetes;o__Pleosporales;f__Sporormiaceae	Preussia
0,35%	0,03%	0,37%	0,07%	0,04%	0,04%	0,03%	0,02%	k__Fungi;p__Ascomycota;c__Sordariomycetes;o__Sordariales;f__Chaetomiaceae	Chaetomium
0,00%	0,01%	0,09%	0,00%	0,01%	0,00%	0,81%	0,01%	k__Fungi;p__Ascomycota;c__Pezizomycetes;o__Pezizales;f__Pyronemataceae	Humaria
0,08%	0,01%	0,14%	0,00%	0,10%	0,06%	0,23%	0,23%	k__Fungi;p__Ascomycota;c__Leotiomycetes;o__Helotiales;f__Helotiales_fam_Incertae_sedis	Cadophora
0,23%	0,01%	0,00%	0,01%	0,52%	0,07%	0,01%	0,01%	k__Fungi;p__Basidiomycota;c__Agaricomycetes;o__Cantharellales;f__Clavulinaceae	Clavulina
0,27%	0,02%	0,12%	0,07%	0,03%	0,10%	0,09%	0,08%	k__Fungi;p__Ascomycota;c__Eurotiomycetes;o__Chaetothyriales;f__Herpotrichiellaceae;Other	
0,03%	0,03%	0,04%	0,04%	0,11%	0,09%	0,22%	0,18%	k__Fungi;p__Ascomycota;c__Leotiomycetes;o__Helotiales;Other;Other	
0,01%	0,01%	0,11%	0,01%	0,05%	0,03%	0,47%	0,03%	k__Fungi;p__Basidiomycota;c__Agaricomycetes;o__Agaricales;f__Hymenogastraceae	Hymenogaster
0,09%	0,15%	0,16%	0,13%	0,00%	0,01%	0,00%	0,00%	k__Fungi;p__Basidiomycota;c__Agaricomycetes;o__Cantharellales;f__Hygrophoraceae	unidentified
0,00%	0,00%	0,00%	0,05%	0,00%	0,00%	0,00%	0,59%	k__Fungi;p__Basidiomycota;c__Agaricomycetes;o__Russulales;f__Russulaceae	Lactarius

0,20%	0,01%	0,18%	0,07%	0,02%	0,02%	0,01%	0,01%	k__Fungi;p__Ascomycota;c__Sordariomycetes;o__Sordariales;f__Chaetomiaceae	unidentified
0,08%	0,03%	0,15%	0,04%	0,02%	0,02%	0,14%	0,02%	k__Fungi;p__Basidiomycota;c__Tremellomycetes;o__Trichosporonales;f__Trichosporonaceae	Apiotrichum
0,00%	0,11%	0,00%	0,00%	0,06%	0,06%	0,07%	0,07%	k__Fungi;p__Basidiomycota;c__Agaricomycetes;o__Boletales;f__Boletaceae	Boletus
0,01%	0,00%	0,01%	0,02%	0,01%	0,20%	0,02%	0,20%	k__Fungi;p__Ascomycota;c__Eurotiomycetes;o__Onygenales;f__Onygenaceae	Auxarthron
0,05%	0,03%	0,02%	0,14%	0,04%	0,05%	0,03%	0,07%	k__Fungi;Other;Other;Other;Other;Other	
0,00%	0,03%	0,00%	0,00%	0,08%	0,07%	0,13%	0,11%	k__Fungi;p__Basidiomycota;c__Agaricomycetes;o__Agaricales;f__Psathyrellaceae	unidentified
0,06%	0,03%	0,11%	0,07%	0,04%	0,03%	0,04%	0,03%	k__Fungi;p__Basidiomycota;c__Agaricomycetes;o__Gomphales;f__Gomphaceae	Clavariadelphus
0,21%	0,02%	0,01%	0,00%	0,09%	0,02%	0,01%	0,05%	k__Fungi;p__Ascomycota;c__Eurotiomycetes;o__Eurotiales;f__unidentified	unidentified
0,03%	0,01%	0,02%	0,00%	0,07%	0,12%	0,08%	0,07%	k__Fungi;p__Ascomycota;c__Eurotiomycetes;o__Chaetothyriales;f__unidentified	unidentified
0,00%	0,03%	0,00%	0,00%	0,08%	0,09%	0,08%	0,09%	k__Fungi;p__Basidiomycota;c__Agaricomycetes;o__Geastrales;f__Geastraceae	Geastrum
0,12%	0,01%	0,04%	0,02%	0,05%	0,04%	0,08%	0,03%	k__Fungi;p__Ascomycota;c__Eurotiomycetes;o__Eurotiales;f__Trichocomaceae	Talaromyces
0,16%	0,00%	0,01%	0,04%	0,01%	0,02%	0,01%	0,11%	k__Fungi;p__Ascomycota;c__Leotiomycetes;o__Helotiales;f__Myxotrichaceae;Other	
0,01%	0,01%	0,07%	0,02%	0,05%	0,05%	0,10%	0,03%	k__Fungi;p__Ascomycota;c__Sordariomycetes;o__unidentified;f__unidentified	unidentified
0,09%	0,04%	0,09%	0,05%	0,01%	0,02%	0,02%	0,01%	k__Fungi;p__Basidiomycota;c__Tremellomycetes;o__Trichosporonales;f__Trichosporonaceae	Cutaneotrichosporon
0,00%	0,03%	0,00%	0,00%	0,05%	0,05%	0,09%	0,11%	k__Fungi;p__Ascomycota;c__Sordariomycetes;o__Microascales;f__Microascaceae	Pseudallescheria
0,04%	0,01%	0,02%	0,03%	0,02%	0,04%	0,05%	0,10%	k__Fungi;p__Ascomycota;c__Eurotiomycetes;o__Chaetothyriales;f__Herpotrichiellaceae	Cladophialophora
0,01%	0,01%	0,03%	0,00%	0,04%	0,04%	0,12%	0,04%	k__Fungi;p__Ascomycota;c__Dothideomycetes;o__Capnodiales;f__Cladosporiaceae	Cladosporium
0,01%	0,00%	0,20%	0,01%	0,01%	0,00%	0,08%	0,00%	k__Fungi;p__Basidiomycota;c__Agaricomycetes;o__Agaricales;f__Tricholomataceae	Paralepista
0,00%	0,06%	0,00%	0,00%	0,04%	0,04%	0,05%	0,06%	k__Fungi;p__Basidiomycota;c__Agaricomycetes;o__Boletales;f__Boletaceae	Neoboletus
0,02%	0,00%	0,00%	0,00%	0,06%	0,11%	0,08%	0,02%	k__Fungi;p__Ascomycota;c__Pezizomycetes;o__Pezizales;f__Helvellaceae	Helvella
0,14%	0,00%	0,05%	0,02%	0,01%	0,01%	0,05%	0,01%	k__Fungi;p__Ascomycota;c__Sordariomycetes;o__Ophiostomatales;f__Ophiostomataceae	Sporothrix
0,05%	0,00%	0,00%	0,00%	0,07%	0,14%	0,01%	0,01%	k__Fungi;p__Ascomycota;c__Eurotiomycetes;o__Onygenales;f__Onygenaceae	unidentified
0,07%	0,01%	0,01%	0,00%	0,05%	0,04%	0,05%	0,03%	k__Fungi;p__Ascomycota;c__unidentified;o__unidentified;f__unidentified	unidentified
0,01%	0,03%	0,00%	0,04%	0,02%	0,08%	0,01%	0,06%	k__Fungi;p__Ascomycota;c__Sordariomycetes;o__Hypocreales;Other;Other	
0,05%	0,01%	0,09%	0,08%	0,00%	0,00%	0,00%	0,00%	k__Fungi;p__Ascomycota;c__Sordariomycetes;o__Hypocreales;f__Ophiocordycipitaceae	Ophiocordyceps
0,03%	0,01%	0,04%	0,02%	0,06%	0,03%	0,03%	0,02%	k__Fungi;p__Basidiomycota;c__Agaricomycetes;o__Agaricales;f__Tricholomataceae	Mycenella
0,00%	0,00%	0,06%	0,02%	0,01%	0,01%	0,06%	0,06%	k__Fungi;p__Ascomycota;c__Eurotiomycetes;o__Onygenales;f__Ajellomycetaceae	Histoplasma
0,01%	0,00%	0,01%	0,05%	0,01%	0,00%	0,00%	0,14%	k__Fungi;p__Basidiomycota;c__Microbotryomycetes;o__Leucosporidiales;f__unidentified	unidentified
0,00%	0,00%	0,00%	0,00%	0,08%	0,01%	0,10%	0,01%	k__Fungi;p__Ascomycota;c__Dothideomycetes;o__Dothideales;f__Aureobasidiaceae	Aureobasidium
0,05%	0,01%	0,01%	0,01%	0,03%	0,03%	0,04%	0,02%	k__Fungi;p__Basidiomycota;c__Tremellomycetes;o__unidentified;f__unidentified	unidentified
0,01%	0,00%	0,20%	0,00%	0,00%	0,00%	0,00%	0,00%	k__Fungi;p__Mucoromycota;c__Mucoromycetes;o__Mucorales;f__Cunninghamellaceae	Cunninghamella
0,01%	0,01%	0,00%	0,00%	0,03%	0,04%	0,07%	0,03%	k__Fungi;p__Ascomycota;c__Sordariomycetes;o__Hypocreales;f__Nectriaceae	Neonectria
0,00%	0,01%	0,00%	0,00%	0,02%	0,03%	0,06%	0,06%	k__Fungi;p__Ascomycota;c__Saccharomycetes;o__Saccharomycetales;f__Dipodascaceae	Geotrichum
0,08%	0,00%	0,00%	0,01%	0,01%	0,00%	0,01%	0,08%	k__Fungi;p__Basidiomycota;c__Agaricomycetes;o__Boletales;f__Melanogastraceae	Melanogaster
0,00%	0,01%	0,01%	0,01%	0,04%	0,03%	0,05%	0,04%	k__Fungi;p__Ascomycota;c__Sordariomycetes;o__Hypocreales;f__Hypocreales_fam_Incertae_sedis	Acremonium
0,02%	0,02%	0,01%	0,00%	0,04%	0,03%	0,04%	0,02%	k__Fungi;p__Basidiomycota;c__Agaricomycetes;o__Agaricales;f__Lycoperdaceae	Lycoperdon
0,11%	0,00%	0,01%	0,00%	0,02%	0,02%	0,01%	0,00%	k__Fungi;p__Ascomycota;c__Leotiomycetes;o__Helotiales;f__Dermateaceae	Pezicula
0,01%	0,01%	0,03%	0,01%	0,02%	0,04%	0,03%	0,02%	k__Fungi;p__Ascomycota;c__Sordariomycetes;Other;Other;Other	
0,09%	0,00%	0,04%	0,01%	0,01%	0,01%	0,01%	0,01%	k__Fungi;p__Ascomycota;c__Sordariomycetes;o__Hypocreales;f__Nectriaceae	unidentified
0,01%	0,00%	0,02%	0,00%	0,11%	0,00%	0,01%	0,00%	k__Fungi;p__Ascomycota;c__Eurotiomycetes;o__Onygenales;f__Onygenales_fam_Incertae_sedis	Chrysosporium
0,00%	0,00%	0,00%	0,00%	0,02%	0,02%	0,05%	0,06%	k__Fungi;p__Ascomycota;c__Sordariomycetes;o__Myrmecridiales;f__unidentified	unidentified
0,03%	0,00%	0,02%	0,01%	0,08%	0,01%	0,01%	0,01%	k__Fungi;p__Ascomycota;c__Eurotiomycetes;o__Eurotiales;f__Aspergillaceae;Other	
0,04%	0,01%	0,06%	0,05%	0,00%	0,00%	0,00%	0,00%	k__Fungi;p__Ascomycota;c__Lecanoromycetes;o__Peltigerales;f__Collemataceae	Leptogium
0,03%	0,00%	0,00%	0,01%	0,01%	0,01%	0,01%	0,08%	k__Fungi;p__Basidiomycota;c__Geminibasidiomycetes;o__Geminibasidiales;f__Geminibasidiaceae	Geminibasidium
0,03%	0,01%	0,04%	0,03%	0,01%	0,00%	0,01%	0,00%	k__Fungi;p__Basidiomycota;c__Agaricomycetes;o__Agaricales;f__Tricholomataceae	Macrocystidia
0,00%	0,01%	0,00%	0,00%	0,03%	0,03%	0,03%	0,03%	k__Fungi;p__Ascomycota;c__Sordariomycetes;o__Hypocreales;f__Nectriaceae	Fusarium
0,00%	0,01%	0,00%	0,00%	0,01%	0,02%	0,04%	0,05%	k__Fungi;p__Ascomycota;c__Sordariomycetes;o__Calosphaeriales;f__Pleurostomataceae	Pleurostoma
0,01%	0,02%	0,01%	0,00%	0,01%	0,01%	0,03%	0,03%	k__Fungi;p__Ascomycota;c__Sordariomycetes;o__Hypocreales;f__Nectriaceae;Other	
0,01%	0,00%	0,04%	0,01%	0,02%	0,01%	0,05%	0,01%	k__Fungi;p__Ascomycota;c__Leotiomycetes;o__Thelebolales;f__Pseudeurotiaceae	Pseudogymnoascus
0,02%	0,01%	0,01%	0,01%	0,03%	0,01%	0,02%	0,03%	k__Fungi;p__Ascomycota;c__Dothideomycetes;o__Capnodiales;f__unidentified	unidentified
0,01%	0,00%	0,08%	0,01%	0,01%	0,00%	0,01%	0,00%	k__Fungi;p__Basidiomycota;c__Agaricomycetes;o__Agaricales;Other;Other	
0,00%	0,01%	0,00%	0,00%	0,03%	0,02%	0,03%	0,03%	k__Fungi;p__Ascomycota;c__Sordariomycetes;o__Coniochaetales;f__Coniochaetaceae	Lecythophora
0,01%	0,00%	0,00%	0,00%	0,04%	0,05%	0,02%	0,00%	k__Fungi;p__Basidiomycota;c__Agaricomycetes;o__Thelephorales;f__Thelephoraceae	Thelephora
0,01%	0,01%	0,03%	0,00%	0,01%	0,02%	0,02%	0,02%	k__Fungi;p__Ascomycota;c__Sordariomycetes;o__Microascales;f__Microascaceae	Cephalotrichum
0,00%	0,01%	0,00%	0,00%	0,02%	0,02%	0,04%	0,04%	k__Fungi;p__Ascomycota;c__Saccharomycetes;o__Saccharomycetales;f__Pichiaceae	Pichia
0,01%	0,00%	0,01%	0,00%	0,03%	0,02%	0,04%	0,02%	k__Fungi;p__Ascomycota;c__Dothideomycetes;o__Pleosporales;f__unidentified	unidentified
0,00%	0,01%	0,00%	0,00%	0,02%	0,02%	0,03%	0,03%	k__Fungi;p__Basidiomycota;c__Agaricomycetes;o__Sebacinales;f__Sebacinaceae	Helvellosebacina
0,04%	0,00%	0,00%	0,00%	0,01%	0,04%	0,02%	0,00%	k__Fungi;p__Basidiomycota;c__Agaricomycetes;o__Thelephorales;f__Thelephoraceae;Other	
0,00%	0,01%	0,01%	0,00%	0,01%	0,02%	0,05%	0,02%	k__Fungi;p__Ascomycota;c__Sordariomycetes;o__Hypocreales;f__Cordycipitaceae	Beauveria
0,01%	0,01%	0,01%	0,00%	0,02%	0,02%	0,04%	0,01%	k__Fungi;p__Ascomycota;c__Sordariomycetes;o__Hypocreales;f__Nectriaceae	Ilyonectria
0,00%	0,00%	0,00%	0,04%	0,00%	0,01%	0,01%	0,06%	k__Fungi;p__Ascomycota;c__Eurotiomycetes;o__Chaetothyriales;f__Herpotrichiellaceae	Capronia
0,11%	0,00%	0,00%	0,00%	0,00%	0,00%	0,00%	0,00%	k__Fungi;p__Basidiomycota;c__Agaricomycetes;o__Agaricales;f__Clavariaceae	unidentified
0,00%	0,01%	0,00%	0,00%	0,03%	0,01%	0,04%	0,02%	k__Fungi;p__Basidiomycota;c__Agaricomycetes;o__Hysterangiales;Other;Other	
0,04%	0,00%	0,00%	0,00%	0,06%	0,00%	0,00%	0,00%	k__Fungi;p__Ascomycota;c__Dothideomycetes;o__Capnodiales;f__Capnodiaceae	Readerielliopsis
0,00%	0,00%	0,00%	0,00%	0,02%	0,02%	0,04%	0,02%	k__Fungi;p__Ascomycota;c__Dothideomycetes;o__Pleosporales;f__Didymellaceae;Other	
0,05%	0,00%	0,06%	0,00%	0,00%	0,00%	0,00%	0,00%	k__Fungi;p__Rozellomycota;c__Rozellomycotina_cls_Incertae_sedis;o__GS11;f__unidentified	unidentified
0,00%	0,00%	0,00%	0,00%	0,02%	0,02%	0,03%	0,03%	k__Fungi;p__Ascomycota;c__Leotiomycetes;o__Helotiales;f__Helotiaceae	Helicodendron
0,00%	0,01%	0,00%	0,00%	0,01%	0,01%	0,03%	0,03%	k__Fungi;p__Ascomycota;c__Saccharomycetes;o__Saccharomycetales;f__Dipodascaceae	unidentified
0,00%	0,00%	0,00%	0,00%	0,03%	0,02%	0,03%	0,02%	k__Fungi;p__Basidiomycota;c__Agaricomycetes;o__Agaricales;f__Tricholomataceae	Pseudoclitocybe
0,00%	0,01%	0,00%	0,00%	0,01%	0,01%	0,03%	0,03%	k__Fungi;p__Ascomycota;c__Saccharomycetes;o__Saccharomycetales;f__Saccharomycetaceae	Williopsis
0,06%	0,00%	0,01%	0,02%	0,00%	0,00%	0,00%	0,00%	k__Fungi;p__Basidiomycota;c__Tremellomycetes;o__Tremellales;f__unidentified	unidentified
0,00%	0,00%	0,00%	0,04%	0,01%	0,01%	0,01%	0,03%	k__Fungi;p__Ascomycota;c__Sordariomycetes;o__Hypocreales;f__Clavicipitaceae	Metapochonia
0,04%	0,00%	0,03%	0,00%	0,01%	0,00%	0,00%	0,01%	k__Fungi;p__Ascomycota;c__Eurotiomycetes;o__Eurotiales;Other;Other	
0,00%	0,01%	0,00%	0,00%	0,02%	0,02%	0,03%	0,02%	k__Fungi;p__Ascomycota;c__Eurotiomycetes;o__Eurotiales;f__Aspergillaceae	Monascus
0,01%	0,00%	0,00%	0,00%	0,01%	0,01%	0,01%	0,03%	k__Fungi;p__Ascomycota;c__Eurotiomycetes;o__Chaetothyriales;Other;Other	
0,00%	0,01%	0,01%	0,01%	0,01%	0,01%	0,01%	0,01%	k__Fungi;p__Basidiomycota;Other;Other;Other;Other	
0,01%	0,00%	0,00%	0,00%	0,01%	0,04%	0,01%	0,01%	k__Fungi;p__Ascomycota;c__Eurotiomycetes;o__Onygenales;Other;Other	
0,00%	0,00%	0,00%	0,00%	0,02%	0,01%	0,03%	0,02%	k__Fungi;p__Ascomycota;c__Leotiomycetes;o__Helotiales;f__Dermateaceae	Cryptosporiopsis
0,01%	0,00%	0,02%	0,01%	0,00%	0,00%	0,03%	0,00%	k__Fungi;p__Ascomycota;c__Sordariomycetes;o__Chaetosphaeriales;f__Chaetosphaeriaceae	Chloridium
0,00%	0,00%	0,00%	0,00%	0,02%	0,02%	0,02%	0,02%	k__Fungi;p__Basidiomycota;c__Agaricomycetes;o__Auriculariales;f__Hyaloriaceae	Protodontia
0,02%	0,00%	0,01%	0,00%	0,01%	0,00%	0,00%	0,04%	k__Fungi;p__Mucoromycota;c__Mucoromycotina_cls_Incertae_sedis;o__Mucoromycotina_ord_Incertae_sedis;f__Mucoromycotina_fam_Incertae_sedis	Bifiguratus
0,02%	0,01%	0,00%	0,00%	0,02%	0,01%	0,01%	0,00%	k__Fungi;p__Basidiomycota;c__Agaricomycetes;o__Agaricales;f__Hydnangiaceae	Laccaria
0,00%	0,04%	0,00%	0,00%	0,00%	0,00%	0,00%	0,00%	k__Fungi;p__Basidiomycota;c__Agaricomycetes;o__Trechisporales;f__Trechisporales_fam_Incertae_sedis	Scytinopogon
0,00%	0,01%	0,00%	0,00%	0,01%	0,02%	0,02%	0,02%	k__Fungi;p__Ascomycota;c__Pezizomycetes;o__Pezizales;f__Pyronemataceae	Wilcoxina
0,00%	0,00%	0,00%	0,00%	0,01%	0,01%	0,04%	0,02%	k__Fungi;p__Basidiomycota;c__Agaricomycetes;o__Agaricales;f__Omphalotaceae	Rhodocollybia
0,00%	0,00%	0,00%	0,00%	0,02%	0,01%	0,03%	0,02%	k__Fungi;p__Ascomycota;c__Pezizomycetes;o__Pezizales;f__Sarcoscyphaceae	Desmazierella
0,03%	0,00%	0,02%	0,02%	0,00%	0,00%	0,00%	0,00%	k__Fungi;p__Basidiomycota;c__Tremellomycetes;o__Trichosporonales;f__Trichosporonaceae	Vanrija
0,02%	0,00%	0,00%	0,00%	0,01%	0,01%	0,03%	0,01%	k__Fungi;p__Ascomycota;c__Leotiomycetes;o__unidentified;f__unidentified	unidentified
0,00%	0,01%	0,00%	0,00%	0,01%	0,02%	0,01%	0,02%	k__Fungi;p__Ascomycota;c__Dothideomycetes;o__Pleosporales;Other;Other	
0,00%	0,00%	0,02%	0,00%	0,01%	0,00%	0,04%	0,01%	k__Fungi;p__Basidiomycota;c__Agaricomycetes;o__Agaricales;f__Tricholomataceae;Other	
0,01%	0,00%	0,02%	0,00%	0,01%	0,01%	0,01%	0,01%	k__Fungi;p__Ascomycota;c__Sordariomycetes;o__Sordariales;Other;Other	
0,00%	0,00%	0,00%	0,02%	0,00%	0,00%	0,01%	0,03%	k__Fungi;p__Ascomycota;c__Leotiomycetes;o__Helotiales;f__Hyaloscyphaceae	unidentified
0,01%	0,00%	0,01%	0,01%	0,01%	0,02%	0,01%	0,01%	k__Fungi;p__Ascomycota;c__Pezizomycotina_cls_Incertae_sedis;o__Pezizomycotina_ord_Incertae_sedis;f__Pezizomycotina_fam_Incertae_sedis	Ciliophora

0,00%	0,00%	0,00%	0,00%	0,02%	0,01%	0,02%	0,02%	k__Fungi;p__Ascomycota;c__Leotiomycetes;o__Helotiales;f__Dermateaceae;Other	
0,00%	0,01%	0,00%	0,00%	0,01%	0,01%	0,01%	0,03%	k__Fungi;p__Basidiomycota;c__Microbotryomycetes;o__Sporidiobolales;f__Sporidiobolaceae	Rhodotorula
0,00%	0,00%	0,00%	0,00%	0,01%	0,01%	0,03%	0,01%	k__Fungi;p__Ascomycota;c__Pezizomycetes;o__Pezizales;f__Pyronemataceae	Trichophaea
0,03%	0,00%	0,01%	0,01%	0,00%	0,01%	0,01%	0,00%	k__Fungi;p__Ascomycota;c__Sordariomycetes;o__Hypocreales;f__Bionectriaceae	Clonostachys
0,01%	0,00%	0,00%	0,00%	0,01%	0,02%	0,00%	0,01%	k__Fungi;p__Ascomycota;c__Sordariomycetes;o__Xylariales;f__Xylariaceae	Ascotricha
0,00%	0,00%	0,00%	0,01%	0,01%	0,01%	0,02%	0,01%	k__Fungi;p__Basidiomycota;c__Agaricomycetes;o__Tremellodendropsidales;f__unidentified	unidentified
0,00%	0,01%	0,00%	0,00%	0,02%	0,01%	0,02%	0,01%	k__Fungi;p__Basidiomycota;c__Agaricomycetes;o__Thelephorales;f__Thelephoraceae	Odontia
0,00%	0,00%	0,00%	0,00%	0,01%	0,01%	0,02%	0,03%	k__Fungi;p__Ascomycota;c__Leotiomycetes;o__Helotiales;f__Helotiales_fam_Incertae_sedis	Chalara
0,00%	0,00%	0,00%	0,00%	0,01%	0,01%	0,02%	0,02%	k__Fungi;p__Ascomycota;c__Pezizomycetes;o__Pezizales;f__Pyronemataceae	Geopora
0,02%	0,02%	0,00%	0,00%	0,00%	0,00%	0,00%	0,01%	k__Fungi;p__Basidiomycota;c__Agaricomycetes;o__Agaricales;f__Cortinariaceae	unidentified
0,00%	0,02%	0,00%	0,00%	0,01%	0,01%	0,01%	0,01%	k__Fungi;p__Basidiomycota;c__Agaricomycetes;Other;Other;Other	
0,02%	0,00%	0,00%	0,00%	0,00%	0,01%	0,02%	0,01%	k__Fungi;p__Ascomycota;c__Leotiomycetes;o__Thelebolales;f__Pseudeurotiaceae	Pseudeurotium
0,00%	0,00%	0,00%	0,00%	0,01%	0,01%	0,02%	0,02%	k__Fungi;p__Ascomycota;c__Sordariomycetes;o__Hypocreales;f__Nectriaceae	Volutella
0,00%	0,00%	0,00%	0,00%	0,02%	0,01%	0,01%	0,02%	k__Fungi;p__Ascomycota;c__Sordariomycetes;o__Sordariales;f__Sordariales_fam_Incertae_sedis	Ramophialophora
0,03%	0,00%	0,00%	0,00%	0,00%	0,01%	0,00%	0,01%	k__Fungi;p__Ascomycota;c__Sordariomycetes;o__Hypocreales;f__Cordycipitaceae	Lecanicillium
0,00%	0,00%	0,00%	0,01%	0,01%	0,01%	0,01%	0,01%	k__Fungi;p__Basidiomycota;c__Agaricomycetes;o__Geastrales;f__Sclerogastraceae	Sclerogaster
0,00%	0,02%	0,00%	0,00%	0,00%	0,00%	0,00%	0,00%	k__Fungi;p__Ascomycota;c__Eurotiomycetes;o__Onygenales;f__Onygenaceae	Myriodontium
0,01%	0,00%	0,00%	0,00%	0,01%	0,00%	0,02%	0,02%	k__Fungi;p__Ascomycota;c__Leotiomycetes;o__Helotiales;f__Helotiaceae	Meliniomyces
0,00%	0,00%	0,00%	0,00%	0,00%	0,00%	0,04%	0,00%	k__Fungi;p__Ascomycota;c__Pezizomycetes;o__Pezizales;f__Pezizaceae	Peziza
0,00%	0,00%	0,00%	0,00%	0,01%	0,01%	0,02%	0,01%	k__Fungi;p__Ascomycota;c__Dothideomycetes;o__Pleosporales;f__Sporormiaceae	Westerdykella
0,00%	0,00%	0,00%	0,00%	0,01%	0,01%	0,02%	0,01%	k__Fungi;p__Basidiomycota;c__Agaricomycetes;o__unidentified;f__unidentified	unidentified
0,00%	0,00%	0,00%	0,00%	0,01%	0,02%	0,01%	0,01%	k__Fungi;p__Ascomycota;c__Dothideomycetes;o__Pleosporales;f__Pleosporaceae	Alternaria
0,01%	0,00%	0,00%	0,00%	0,01%	0,01%	0,01%	0,01%	k__Fungi;p__Ascomycota;c__Sordariomycetes;o__Sordariales;f__Lasiosphaeriaceae	unidentified
0,00%	0,00%	0,00%	0,02%	0,00%	0,00%	0,00%	0,03%	k__Fungi;p__Basidiomycota;c__Agaricomycetes;o__Sebacinales;f__unidentified	unidentified
0,00%	0,00%	0,00%	0,00%	0,03%	0,01%	0,01%	0,00%	k__Fungi;p__Ascomycota;c__Sordariomycetes;o__Sordariales;f__Lasiosphaeriaceae	Apodus
0,01%	0,00%	0,03%	0,00%	0,00%	0,00%	0,00%	0,00%	k__Fungi;p__Basidiomycota;c__Agaricomycetes;o__Agaricales;f__Entolomataceae	Entoloma
0,00%	0,00%	0,00%	0,00%	0,01%	0,01%	0,01%	0,02%	k__Fungi;p__Ascomycota;c__Saccharomycetes;o__Saccharomycetales;Other;Other	
0,00%	0,00%	0,01%	0,00%	0,00%	0,00%	0,03%	0,01%	k__Fungi;p__Basidiomycota;c__Cystobasidiomycetes;o__Erythrobasidiales;f__Erythrobasidiales_fam_Incertae_sedis	Sakaguchia
0,00%	0,00%	0,00%	0,00%	0,01%	0,01%	0,01%	0,01%	k__Fungi;p__Basidiomycota;c__Agaricomycetes;o__Agaricales;f__Agaricaceae	Lepiota
0,01%	0,00%	0,00%	0,00%	0,01%	0,01%	0,01%	0,01%	k__Fungi;p__Ascomycota;c__Dothideomycetes;Other;Other;Other	
0,01%	0,00%	0,00%	0,00%	0,03%	0,00%	0,00%	0,00%	k__Fungi;p__Basidiomycota;c__Agaricomycetes;o__Boletales;f__Sclerodermataceae	Scleroderma
0,00%	0,01%	0,00%	0,00%	0,02%	0,01%	0,01%	0,00%	k__Fungi;p__Basidiomycota;c__Agaricomycetes;o__Agaricales;f__Lyophyllaceae	Tephrocybe
0,00%	0,00%	0,00%	0,00%	0,01%	0,01%	0,01%	0,01%	k__Fungi;p__Basidiomycota;c__Agaricomycetes;o__Agaricales;f__Bolbitiaceae	Conocybe
0,00%	0,00%	0,00%	0,00%	0,00%	0,00%	0,04%	0,01%	k__Fungi;p__Ascomycota;c__Sordariomycetes;o__Chaetosphaeriales;Other;Other	
0,00%	0,00%	0,00%	0,00%	0,01%	0,01%	0,02%	0,01%	k__Fungi;p__Ascomycota;c__Pezizomycetes;o__Pezizales;f__Pyronemataceae	unidentified
0,01%	0,00%	0,00%	0,01%	0,00%	0,00%	0,00%	0,02%	k__Fungi;p__Mucoromycota;Other;Other;Other;Other	
0,00%	0,00%	0,00%	0,00%	0,00%	0,04%	0,00%	0,00%	k__Fungi;p__Ascomycota;c__Pezizomycetes;o__Pezizales;f__Pyronemataceae	Pustularia
0,00%	0,00%	0,05%	0,00%	0,00%	0,00%	0,00%	0,00%	k__Fungi;p__Glomeromycota;c__Glomeromycetes;o__Diversisporales;f__Diversisporaceae	Diversispora
0,00%	0,00%	0,00%	0,00%	0,01%	0,01%	0,01%	0,01%	k__Fungi;p__Ascomycota;c__Dothideomycetes;o__Venturiales;f__Venturiaceae	unidentified
0,00%	0,00%	0,00%	0,00%	0,00%	0,02%	0,00%	0,02%	k__Fungi;p__Basidiomycota;c__Agaricomycetes;o__Agaricales;f__Entolomataceae	Clitopilus
0,00%	0,00%	0,00%	0,01%	0,01%	0,01%	0,01%	0,01%	k__Fungi;p__Ascomycota;c__Dothideomycetes;o__unidentified;f__unidentified	unidentified
0,00%	0,00%	0,00%	0,00%	0,00%	0,00%	0,04%	0,00%	k__Fungi;p__Basidiomycota;c__Agaricomycetes;o__Boletales;f__Boletaceae	unidentified
0,00%	0,00%	0,00%	0,00%	0,01%	0,01%	0,01%	0,01%	k__Fungi;p__Ascomycota;c__Dothideomycetes;o__Venturiales;f__Venturiaceae	Venturia
0,01%	0,00%	0,00%	0,00%	0,01%	0,01%	0,01%	0,00%	k__Fungi;p__Ascomycota;c__Sordariomycetes;o__Hypocreales;f__Ophiocordycipitaceae	Purpureocillium
0,00%	0,00%	0,00%	0,00%	0,01%	0,01%	0,01%	0,01%	k__Fungi;p__Ascomycota;c__Leotiomycetes;o__Thelebolales;f__Thelebolaceae	Thelebolus
0,04%	0,00%	0,00%	0,00%	0,00%	0,00%	0,00%	0,00%	k__Fungi;p__Basidiomycota;c__Agaricomycetes;o__Russulales;f__Russulaceae	Gymnomyces
0,01%	0,00%	0,01%	0,01%	0,00%	0,00%	0,00%	0,01%	k__Fungi;p__Basidiomycota;c__Microbotryomycetes;o__Microbotryomycetes_ord_Incertae_sedis;f__Chrysozymaceae	Udeniozyma
0,00%	0,00%	0,01%	0,02%	0,00%	0,00%	0,00%	0,01%	k__Fungi;p__Ascomycota;c__Sordariomycetes;o__Hypocreales;f__Clavicipitaceae	unidentified
0,00%	0,00%	0,01%	0,00%	0,00%	0,00%	0,02%	0,01%	k__Fungi;p__Ascomycota;c__Sordariomycetes;o__Chaetosphaeriales;f__Chaetosphaeriaceae	Chaetosphaeria

(D)

PI1a	PI2a	PI3a	PI4a	PII1a	PII2a	PII3a	PII4a	taxonomy	
1,26%	33,89%	3,59%	2,04%	0,32%	62,13%	0,69%	0,16%	k__Fungi;p__Basidiomycota;c__Agaricomycetes;o__Agaricales;f__Tricholomataceae	Mycena
0,46%	6,04%	27,50%	7,27%	11,10%	11,20%	9,20%	3,59%	k__Fungi;p__Basidiomycota;c__Agaricomycetes;o__Sebacinales;f__Sebacinaceae	Sebacina
0,16%	0,20%	24,26%	20,80%	0,13%	0,11%	0,17%	0,13%	k__Fungi;p__Basidiomycota;c__Agaricomycetes;o__Trechisporales;f__Hydnodontaceae	Trechispora
8,56%	3,53%	2,94%	3,54%	2,34%	1,23%	13,65%	6,48%	k__Fungi;p__unidentified;c__unidentified;o__unidentified;f__unidentified	unidentified
11,18%	0,92%	1,38%	4,64%	0,58%	0,67%	6,22%	6,33%	k__Fungi;p__Mortierellomycota;c__Mortierellomycetes;o__Mortierellales;f__Mortierellaceae	Mortierella
0,29%	0,34%	0,17%	0,31%	29,16%	0,18%	0,26%	0,20%	k__Fungi;p__Basidiomycota;c__Agaricomycetes;o__Agaricales;f__Tricholomataceae	Tricholoma
3,25%	1,59%	2,28%	2,03%	1,71%	2,98%	3,02%	10,08%	k__Fungi;p__Ascomycota;c__Leotiomycetes;o__Helotiales;f__unidentified	unidentified
1,07%	0,41%	1,17%	2,51%	7,27%	2,39%	8,50%	0,17%	k__Fungi;p__Basidiomycota;c__Agaricomycetes;o__Thelephorales;f__Thelephoraceae	unidentified
0,73%	4,51%	0,25%	13,79%	0,15%	0,57%	0,20%	0,08%	k__Fungi;p__Basidiomycota;c__Agaricomycetes;o__Agaricales;f__Tricholomataceae	unidentified
3,97%	1,26%	1,36%	3,21%	0,81%	0,91%	2,49%	2,81%	k__Fungi;p__Ascomycota;c__Leotiomycetes;o__Helotiales;Other;Other	Helotiales_Other
0,82%	0,60%	1,06%	1,19%	0,44%	3,57%	1,87%	4,81%	k__Fungi;p__Ascomycota;c__Eurotiomycetes;o__Eurotiales;f__Aspergillaceae	Penicillium
0,02%	13,96%	0,02%	0,02%	0,02%	0,02%	0,07%	0,02%	k__Fungi;p__Basidiomycota;c__Agaricomycetes;o__Auriculariales;f__Hyaloriaceae	Protodontia
3,75%	3,69%	1,47%	1,68%	0,22%	0,13%	1,00%	1,24%	k__Fungi;p__Ascomycota;c__Dothideomycetes;o__Pleosporales;f__Sporormiaceae	Preussia
0,04%	0,04%	0,02%	0,05%	11,61%	0,01%	0,05%	0,04%	k__Fungi;p__Ascomycota;c__Pezizomycetes;o__Pezizales;f__Sarcoscyphaceae	Desmazierella
1,79%	0,29%	0,46%	0,58%	1,82%	0,24%	0,81%	5,58%	k__Fungi;p__Ascomycota;c__Eurotiomycetes;o__Chaetothyriales;f__Herpotrichiellaceae	Exophiala
0,80%	0,55%	0,29%	0,33%	0,23%	0,59%	2,20%	6,30%	k__Fungi;p__Ascomycota;c__Dothideomycetes;o__Capnodiales;f__Cladosporiaceae	Cladosporium
4,17%	0,48%	1,21%	1,74%	0,12%	0,27%	1,61%	1,29%	k__Fungi;p__Ascomycota;c__Sordariomycetes;o__Sordariales;f__Chaetomiaceae;Other	Chaetomiaceae_Other
0,88%	0,45%	0,42%	0,53%	0,34%	0,27%	0,58%	6,82%	k__Fungi;p__Ascomycota;c__Sordariomycetes;o__Coniochaetales;f__Coniochaetaceae	Coniochaeta
3,29%	0,27%	0,27%	2,04%	0,17%	0,17%	0,81%	3,25%	k__Fungi;p__Basidiomycota;c__Tremellomycetes;o__Filobasidiales;f__Piskurozymaceae	Solicoccozyma
5,45%	0,82%	0,47%	0,53%	0,47%	0,94%	0,54%	0,53%	k__Fungi;p__Basidiomycota;c__Agaricomycetes;o__Russulales;f__Russulaceae	Russula
3,33%	0,39%	0,86%	1,37%	0,09%	0,21%	1,35%	1,06%	k__Fungi;p__Ascomycota;c__Sordariomycetes;o__Sordariales;f__Chaetomiaceae	Humicola
0,60%	0,18%	0,26%	0,16%	0,06%	0,03%	0,12%	6,53%	k__Fungi;p__Ascomycota;c__Sordariomycetes;o__Coniochaetales;f__Coniochaetaceae	Lecythophora
0,21%	0,02%	0,01%	0,03%	6,57%	0,01%	0,03%	0,01%	k__Fungi;p__Basidiomycota;c__Agaricomycetes;o__Thelephorales;f__Thelephoraceae	Odontia
0,06%	5,24%	0,03%	0,06%	0,03%	0,01%	0,04%	0,02%	k__Fungi;p__Basidiomycota;c__Agaricomycetes;o__Agaricales;f__Tricholomataceae	Pseudoclitocybe
0,23%	0,56%	2,02%	0,68%	0,40%	0,67%	0,39%	0,16%	k__Fungi;p__Basidiomycota;c__Agaricomycetes;o__Agaricales;f__Inocybaceae	Inocybe
0,44%	0,09%	0,34%	0,16%	2,54%	0,39%	0,71%	0,06%	k__Fungi;p__Basidiomycota;c__Agaricomycetes;o__Thelephorales;f__Thelephoraceae	Tomentella
0,27%	0,71%	0,32%	0,56%	0,04%	0,27%	1,40%	0,99%	k__Fungi;p__Ascomycota;c__Dothideomycetes;o__Pleosporales;f__Didymellaceae;Other	
0,47%	0,11%	0,11%	0,24%	1,79%	0,06%	0,47%	1,10%	k__Fungi;p__Ascomycota;c__Eurotiomycetes;o__Chaetothyriales;f__Herpotrichiellaceae;Other	
0,70%	0,28%	0,52%	0,56%	0,17%	0,15%	1,15%	0,62%	k__Fungi;p__Ascomycota;Other;Other;Other;Other	
0,45%	0,52%	0,84%	0,53%	0,40%	0,41%	0,40%	0,32%	k__Fungi;p__Basidiomycota;c__Agaricomycetes;o__Agaricales;f__Cortinariaceae	Cortinarius
0,22%	0,02%	3,08%	0,14%	0,03%	0,06%	0,15%	0,07%	k__Fungi;p__Ascomycota;c__Pezizomycetes;o__Pezizales;f__Tuberaceae	Tuber
0,35%	0,11%	0,33%	0,42%	0,03%	0,12%	1,04%	1,23%	k__Fungi;p__Ascomycota;c__Sordariomycetes;o__Hypocreales;f__Nectriaceae	Fusarium
0,33%	0,21%	0,70%	0,43%	0,14%	0,15%	1,19%	0,45%	k__Fungi;p__Ascomycota;c__Leotiomycetes;o__Helotiales;f__Myxotrichaceae	Oidiodendron
0,62%	0,22%	0,24%	0,80%	0,27%	0,16%	0,64%	0,58%	k__Fungi;p__Ascomycota;c__Leotiomycetes;o__Helotiales;f__Dermateaceae	Cryptosporiopsis
2,00%	0,04%	0,04%	0,04%	0,08%	0,03%	1,15%	0,03%	k__Fungi;p__Basidiomycota;c__Agaricomycetes;o__Sebacinales;f__Sebacinaceae	Helvellosebacina
0,11%	0,19%	0,15%	0,14%	0,12%	0,08%	2,55%	0,07%	k__Fungi;p__Basidiomycota;c__Agaricomycetes;o__Agaricales;f__Hymenogastraceae	Hebeloma
0,77%	0,16%	0,25%	0,85%	0,05%	0,10%	0,38%	0,49%	k__Fungi;p__Ascomycota;c__Sordariomycetes;o__Hypocreales;f__Nectriaceae	Neonectria
1,12%	0,25%	0,21%	0,47%	0,06%	0,09%	0,19%	0,47%	k__Fungi;p__Ascomycota;c__Leotiomycetes;o__Helotiales;f__Helotiaceae	Helicodendron
0,02%	0,11%	1,81%	0,76%	0,01%	0,02%	0,11%	0,01%	k__Fungi;p__Basidiomycota;c__Agaricomycetes;Other;Other;Other	
0,52%	0,22%	0,34%	0,48%	0,10%	0,20%	0,39%	0,47%	k__Fungi;p__Ascomycota;c__Leotiomycetes;o__Helotiales;f__Dermateaceae;Other	
0,41%	0,13%	0,26%	0,17%	0,81%	0,05%	0,30%	0,58%	k__Fungi;p__Ascomycota;c__Eurotiomycetes;o__Chaetothyriales;f__Herpotrichiellaceae	Cladophialophora
0,90%	0,17%	0,03%	0,16%	0,21%	0,05%	0,50%	0,52%	k__Fungi;p__Ascomycota;c__Dothideomycetes;o__Venturiales;f__Venturiaceae	Venturia

0,34%	0,30%	0,12%	0,19%	0,18%	0,17%	0,45%	0,74%	k__Fungi;p__Ascomycota;c__Sordariomycetes;o__Hypocreales;f__Hypocreaceae	Trichoderma
0,27%	0,09%	0,33%	0,27%	0,08%	0,06%	1,18%	0,19%	k__Fungi;p__Ascomycota;c__Leotiomycetes;o__Helotiales;f__Helotiales_fam_Incertae_sedis	Cadophora
0,01%	0,03%	0,01%	0,02%	0,01%	0,00%	2,39%	0,01%	k__Fungi;p__Basidiomycota;c__Agaricomycetes;o__Auriculariales;f__Auriculariales_fam_Incertae_sedis	Heterochaetella
0,45%	0,15%	0,24%	0,40%	0,13%	0,25%	0,35%	0,46%	k__Fungi;p__Ascomycota;c__Sordariomycetes;o__Hypocreales;f__unidentified	unidentified
0,04%	0,16%	0,15%	0,46%	0,08%	0,01%	1,35%	0,07%	k__Fungi;p__Basidiomycota;c__Agaricomycetes;o__unidentified;f__unidentified	unidentified
0,53%	0,08%	0,15%	0,48%	0,06%	0,08%	0,38%	0,54%	k__Fungi;p__Ascomycota;c__Dothideomycetes;o__Capnodiales;f__unidentified	unidentified
0,69%	0,23%	0,03%	0,22%	0,05%	0,05%	0,44%	0,61%	k__Fungi;p__Ascomycota;c__Dothideomycetes;o__Venturiales;f__Venturiaceae;Other	
0,37%	0,17%	0,11%	0,31%	0,10%	0,07%	0,47%	0,61%	k__Fungi;p__Ascomycota;c__Leotiomycetes;o__Helotiales;f__Helotiales_fam_Incertae_sedis	Chalara
0,11%	0,85%	0,29%	0,13%	0,04%	0,10%	0,29%	0,39%	k__Fungi;p__Ascomycota;c__Pezizomycetes;o__Pezizales;f__Pyronemataceae	Geopora
1,26%	0,04%	0,04%	0,24%	0,52%	0,01%	0,05%	0,05%	k__Fungi;p__Ascomycota;c__Pezizomycetes;o__Pezizales;f__Pyronemataceae	Wilcoxina
0,05%	0,00%	0,00%	0,02%	0,06%	0,01%	2,04%	0,00%	k__Fungi;p__Basidiomycota;c__Agaricomycetes;o__Thelephorales;f__Thelephoraceae;Other	
0,06%	0,09%	0,73%	0,21%	0,03%	0,07%	0,72%	0,12%	k__Fungi;p__Ascomycota;c__Eurotiomycetes;o__Eurotiales;f__Trichocomaceae	Sagenomella
0,05%	1,58%	0,01%	0,26%	0,03%	0,01%	0,07%	0,03%	k__Fungi;p__Ascomycota;c__Pezizomycetes;o__Pezizales;f__Pyronemataceae	Trichophaea
0,35%	0,14%	0,15%	0,32%	0,19%	0,08%	0,22%	0,56%	k__Fungi;Other;Other;Other;Other;Other	
1,89%	0,01%	0,01%	0,02%	0,01%	0,00%	0,02%	0,02%	k__Fungi;p__Basidiomycota;c__Agaricomycetes;o__Agaricales;f__Agaricaceae	Lepiota
0,31%	0,11%	0,07%	0,11%	0,04%	0,04%	0,33%	0,90%	k__Fungi;p__Ascomycota;c__Dothideomycetes;o__Pleosporales;f__unidentified	unidentified
0,30%	0,33%	0,09%	0,27%	0,06%	0,03%	0,57%	0,24%	k__Fungi;p__Ascomycota;c__Dothideomycetes;o__Venturiales;f__Venturiaceae	unidentified
0,70%	0,37%	0,12%	0,25%	0,04%	0,03%	0,20%	0,16%	k__Fungi;p__Ascomycota;c__Sordariomycetes;o__unidentified;f__unidentified	unidentified
0,23%	0,18%	0,18%	0,22%	0,15%	0,22%	0,28%	0,35%	k__Fungi;p__Ascomycota;c__Eurotiomycetes;o__Eurotiales;f__Thermoascaceae	Byssochlamys
0,21%	0,07%	0,18%	0,20%	0,10%	0,06%	0,64%	0,19%	k__Fungi;p__Ascomycota;c__unidentified;o__unidentified;f__unidentified	unidentified
0,37%	0,18%	0,01%	0,14%	0,01%	0,08%	0,07%	0,73%	k__Fungi;p__Ascomycota;c__Sordariomycetes;o__Sordariales;f__Sordariales_fam_Incertae_sedis	Ramophialophora
0,39%	0,05%	0,07%	0,19%	0,12%	0,05%	0,18%	0,43%	k__Fungi;p__Ascomycota;c__Eurotiomycetes;o__Chaetothyriales;f__unidentified	unidentified
0,16%	0,11%	0,09%	0,15%	0,24%	0,11%	0,19%	0,32%	k__Fungi;p__Ascomycota;c__Leotiomycetes;o__Thelebolales;f__Pseudeurotiaceae	Geomyces
0,06%	0,02%	0,03%	0,04%	0,02%	1,03%	0,06%	0,10%	k__Fungi;p__Ascomycota;c__Sordariomycetes;Other;Other;Other	
0,02%	0,13%	0,17%	0,07%	0,04%	0,01%	0,83%	0,04%	k__Fungi;p__Basidiomycota;Other;Other;Other;Other	
0,67%	0,03%	0,09%	0,09%	0,02%	0,00%	0,34%	0,02%	k__Fungi;p__Chytridiomycota;Other;Other;Other;Other	
0,22%	0,13%	0,08%	0,14%	0,18%	0,07%	0,13%	0,29%	k__Fungi;p__Ascomycota;c__Sordariomycetes;o__Hypocreales;f__Clavicipitaceae	Metarhizium
0,03%	0,99%	0,02%	0,06%	0,03%	0,03%	0,07%	0,02%	k__Fungi;p__Basidiomycota;c__Agaricomycetes;o__Agaricales;f__Hymenogastraceae	Hymenogaster
0,14%	0,04%	0,06%	0,12%	0,01%	0,07%	0,08%	0,70%	k__Fungi;p__Ascomycota;c__Sordariomycetes;o__Hypocreales;f__Nectriaceae	Volutella
0,33%	0,19%	0,10%	0,14%	0,14%	0,10%	0,09%	0,14%	k__Fungi;p__Basidiomycota;c__Agaricomycetes;o__Tremellodendropsidales;f__unidentified	unidentified
0,18%	0,43%	0,01%	0,13%	0,01%	0,07%	0,08%	0,25%	k__Fungi;p__Ascomycota;c__Dothideomycetes;o__Botryosphaeriales;f__unidentified	unidentified
0,01%	0,17%	0,01%	0,01%	0,01%	0,00%	0,90%	0,01%	k__Fungi;p__Basidiomycota;c__Agaricomycetes;o__Agaricales;f__Tricholomataceae	Macrocystidia
0,07%	0,03%	0,02%	0,05%	0,04%	0,03%	0,53%	0,36%	k__Fungi;p__Ascomycota;c__Dothideomycetes;o__Dothideales;f__Aureobasidiaceae	Aureobasidium
0,15%	0,13%	0,09%	0,27%	0,11%	0,05%	0,14%	0,12%	k__Fungi;p__Basidiomycota;c__Agaricomycetes;o__Geastrales;f__Geastraceae	Geastrum
0,17%	0,10%	0,07%	0,10%	0,09%	0,13%	0,19%	0,16%	k__Fungi;p__Basidiomycota;c__Agaricomycetes;o__Russulales;f__Lachnocladiaceae	Vararia
0,17%	0,09%	0,04%	0,18%	0,05%	0,04%	0,23%	0,16%	k__Fungi;p__Ascomycota;c__Leotiomycetes;o__unidentified;f__unidentified	unidentified
0,14%	0,10%	0,04%	0,10%	0,02%	0,02%	0,22%	0,32%	k__Fungi;p__Basidiomycota;c__Tremellomycetes;o__Holtermanniales;f__Holtermanniales_fam_Incertae_sedis	Holtermanniella
0,08%	0,06%	0,02%	0,69%	0,01%	0,03%	0,02%	0,01%	k__Fungi;p__Basidiomycota;c__Agaricomycetes;o__Agaricales;f__Bolbitiaceae	Conocybe
0,25%	0,09%	0,09%	0,40%	0,01%	0,01%	0,02%	0,02%	k__Fungi;p__Ascomycota;c__Leotiomycetes;o__Rhytismatales;f__Rhytismataceae	Lophodermium
0,11%	0,14%	0,06%	0,16%	0,10%	0,09%	0,11%	0,11%	k__Fungi;p__Basidiomycota;c__Agaricomycetes;o__Agaricales;f__Agaricaceae	Agaricus
0,10%	0,07%	0,07%	0,09%	0,14%	0,08%	0,13%	0,19%	k__Fungi;p__Ascomycota;c__Leotiomycetes;o__Thelebolales;f__Pseudeurotiaceae;Other	
0,31%	0,05%	0,04%	0,06%	0,06%	0,01%	0,14%	0,21%	k__Fungi;p__Ascomycota;c__Eurotiomycetes;o__Chaetothyriales;f__Herpotrichiellaceae	Capronia
0,09%	0,09%	0,18%	0,17%	0,08%	0,07%	0,10%	0,07%	k__Fungi;p__Basidiomycota;c__Agaricomycetes;o__Trechisporales;Other;Other	
0,26%	0,07%	0,05%	0,14%	0,02%	0,03%	0,10%	0,16%	k__Fungi;p__Ascomycota;c__Sordariomycetes;o__Sordariales;f__Chaetomiaceae	Chaetomium
0,05%	0,14%	0,10%	0,06%	0,01%	0,06%	0,21%	0,18%	k__Fungi;p__Ascomycota;c__Dothideomycetes;o__Pleosporales;f__Pleosporaceae	Alternaria
0,01%	0,01%	0,01%	0,74%	0,00%	0,00%	0,02%	0,00%	k__Fungi;p__Basidiomycota;c__Agaricomycetes;o__Thelephorales;f__Thelephoraceae	Tomentellopsis
0,11%	0,08%	0,06%	0,10%	0,11%	0,07%	0,09%	0,14%	k__Fungi;p__Ascomycota;c__Sordariomycetes;o__Microascales;f__Microascaceae	Pseudallescheria
0,20%	0,16%	0,03%	0,20%	0,01%	0,03%	0,03%	0,10%	k__Fungi;p__Ascomycota;c__Leotiomycetes;o__Thelebolales;f__Thelebolaceae	Thelebolus
0,34%	0,04%	0,06%	0,10%	0,01%	0,02%	0,09%	0,10%	k__Fungi;p__Ascomycota;c__Sordariomycetes;o__Sordariales;f__Chaetomiaceae	unidentified
0,16%	0,05%	0,03%	0,12%	0,03%	0,03%	0,13%	0,21%	k__Fungi;p__Ascomycota;c__Dothideomycetes;Other;Other;Other	
0,14%	0,09%	0,01%	0,04%	0,35%	0,01%	0,03%	0,05%	k__Fungi;p__Ascomycota;c__Sordariomycetes;o__Myrmecridiales;f__unidentified	unidentified
0,09%	0,12%	0,06%	0,12%	0,09%	0,09%	0,10%	0,06%	k__Fungi;p__Basidiomycota;c__Agaricomycetes;o__Agaricales;f__Psathyrellaceae	unidentified
0,16%	0,03%	0,04%	0,13%	0,02%	0,05%	0,11%	0,19%	k__Fungi;p__Ascomycota;c__Sordariomycetes;o__Hypocreales;f__Nectriaceae;Other	
0,01%	0,01%	0,01%	0,02%	0,57%	0,01%	0,00%	0,06%	k__Fungi;p__Ascomycota;c__Sordariomycetes;o__Microascales;f__Microascaceae	Cephalotrichum
0,14%	0,04%	0,02%	0,10%	0,03%	0,03%	0,13%	0,18%	k__Fungi;p__Ascomycota;c__Dothideomycetes;o__unidentified;f__unidentified	unidentified
0,01%	0,01%	0,00%	0,01%	0,04%	0,01%	0,01%	0,57%	k__Fungi;p__Basidiomycota;c__Agaricomycetes;o__Agaricales;f__Tricholomataceae	Melanoleuca
0,03%	0,02%	0,01%	0,02%	0,00%	0,01%	0,53%	0,03%	k__Fungi;p__Ascomycota;c__Dothideomycetes;o__Capnodiales;f__Mycosphaerellaceae	Mycocentrospora
0,12%	0,05%	0,06%	0,07%	0,02%	0,03%	0,10%	0,16%	k__Fungi;p__Basidiomycota;c__Tremellomycetes;o__Cystofilobasidiales;f__Mrakiaceae	Tausonia
0,05%	0,03%	0,07%	0,05%	0,17%	0,05%	0,05%	0,15%	k__Fungi;p__Ascomycota;c__Leotiomycetes;o__Thelebolales;f__Pseudeurotiaceae	Pseudogymnoascus
0,04%	0,05%	0,05%	0,07%	0,23%	0,05%	0,07%	0,05%	k__Fungi;p__Basidiomycota;c__Agaricomycetes;o__Boletales;f__Boletaceae	Boletus
0,09%	0,05%	0,03%	0,07%	0,02%	0,02%	0,09%	0,24%	k__Fungi;p__Ascomycota;c__Dothideomycetes;o__Pleosporales;Other;Other	
0,08%	0,07%	0,06%	0,06%	0,02%	0,01%	0,13%	0,16%	k__Fungi;p__Ascomycota;c__Dothideomycetes;o__Dothideales;f__Dothioraceae	Hormonema
0,17%	0,01%	0,00%	0,02%	0,37%	0,00%	0,01%	0,00%	k__Fungi;p__Basidiomycota;c__Agaricomycetes;o__Agaricales;f__Lyophyllaceae	Tephrocybe
0,14%	0,02%	0,03%	0,07%	0,02%	0,02%	0,10%	0,18%	k__Fungi;p__Ascomycota;c__Leotiomycetes;o__Helotiales;f__Hyaloscyphaceae	unidentified
0,07%	0,05%	0,01%	0,08%	0,03%	0,01%	0,13%	0,19%	k__Fungi;p__Ascomycota;c__Dothideomycetes;o__Venturiales;Other;Other	
0,10%	0,03%	0,04%	0,09%	0,03%	0,03%	0,09%	0,14%	k__Fungi;p__Ascomycota;c__Sordariomycetes;o__Hypocreales;f__Hypocreales_fam_Incertae_sedis	Acremonium
0,12%	0,02%	0,18%	0,08%	0,01%	0,01%	0,06%	0,07%	k__Fungi;p__Ascomycota;c__Sordariomycetes;o__Sordariales;f__Lasiosphaeriaceae;Other	
0,33%	0,00%	0,01%	0,01%	0,09%	0,01%	0,06%	0,00%	k__Fungi;p__Basidiomycota;c__Agaricomycetes;o__Thelephorales;f__Thelephoraceae	Thelephora
0,03%	0,01%	0,00%	0,02%	0,15%	0,00%	0,30%	0,00%	k__Fungi;p__Chytridiomycota;c__Spizellomycetes;o__Spizellomycetales;f__Spizellomycetaceae	Kochiomyces
0,09%	0,01%	0,19%	0,01%	0,03%	0,01%	0,16%	0,02%	k__Fungi;p__Ascomycota;c__Pezizomycetes;o__Pezizales;f__Pyronemataceae	Humaria
0,08%	0,03%	0,08%	0,08%	0,02%	0,03%	0,06%	0,13%	k__Fungi;p__Ascomycota;c__Pezizomycotina_cls_Incertae_sedis;o__Pezizomycotina_ord_Incertae_sedis;f__Pezizomycotina_fam_Incertae_sedis	Ciliophora
0,04%	0,03%	0,20%	0,04%	0,01%	0,02%	0,10%	0,04%	k__Fungi;p__Ascomycota;c__Leotiomycetes;o__Helotiales;f__Myxotrichaceae	unidentified
0,06%	0,03%	0,05%	0,05%	0,01%	0,02%	0,22%	0,05%	k__Fungi;p__Ascomycota;c__Leotiomycetes;o__Helotiales;f__Helotiaceae	Tetracladium
0,02%	0,02%	0,13%	0,11%	0,02%	0,01%	0,12%	0,06%	k__Fungi;p__Basidiomycota;c__Agaricomycetes;o__Thelephorales;f__Thelephoraceae	Pseudotomentella
0,01%	0,01%	0,01%	0,41%	0,01%	0,01%	0,02%	0,01%	k__Fungi;p__Basidiomycota;c__Agaricomycetes;o__Agaricales;f__Amanitaceae	Amanita
0,10%	0,03%	0,02%	0,04%	0,09%	0,01%	0,07%	0,12%	k__Fungi;p__Ascomycota;c__Eurotiomycetes;o__Chaetothyriales;Other;Other	
0,06%	0,04%	0,08%	0,08%	0,03%	0,02%	0,11%	0,06%	k__Fungi;p__Ascomycota;c__Sordariomycetes;o__Sordariales;f__unidentified	unidentified
0,10%	0,02%	0,10%	0,08%	0,01%	0,01%	0,04%	0,10%	k__Fungi;p__Ascomycota;c__Sordariomycetes;o__Sordariales;Other;Other	
0,03%	0,03%	0,04%	0,05%	0,17%	0,03%	0,05%	0,03%	k__Fungi;p__Basidiomycota;c__Agaricomycetes;o__Boletales;f__Boletaceae	Neoboletus
0,08%	0,09%	0,00%	0,08%	0,01%	0,01%	0,07%	0,10%	k__Fungi;p__Ascomycota;c__Dothideomycetes;o__Pleosporales;f__Melanommataceae	unidentified
0,10%	0,02%	0,01%	0,17%	0,01%	0,01%	0,05%	0,08%	k__Fungi;p__Ascomycota;c__Dothideomycetes;o__Capnodiales;Other;Other	
0,20%	0,02%	0,01%	0,01%	0,00%	0,01%	0,01%	0,17%	k__Fungi;p__Ascomycota;c__Sordariomycetes;o__Coniochaetales;f__unidentified	unidentified
0,06%	0,04%	0,02%	0,06%	0,02%	0,03%	0,04%	0,18%	k__Fungi;p__Basidiomycota;c__Tremellomycetes;o__Tremellales;f__Trimorphomycetaceae	Saitozyma
0,13%	0,01%	0,00%	0,08%	0,01%	0,01%	0,01%	0,16%	k__Fungi;p__Ascomycota;c__Lecanoromycetes;o__GS36;f__unidentified	unidentified
0,09%	0,02%	0,07%	0,08%	0,01%	0,01%	0,09%	0,05%	k__Fungi;p__Ascomycota;c__Sordariomycetes;o__Hypocreales;f__Nectriaceae	Ilyonectria
0,21%	0,02%	0,02%	0,02%	0,03%	0,01%	0,06%	0,04%	k__Fungi;p__Ascomycota;c__Dothideomycetes;o__Pleosporales;f__Didymellaceae	Phoma
0,05%	0,02%	0,01%	0,05%	0,01%	0,04%	0,07%	0,13%	k__Fungi;p__Ascomycota;c__Sordariomycetes;o__Hypocreales;f__Nectriaceae	Gibberella
0,04%	0,01%	0,06%	0,02%	0,02%	0,11%	0,03%	0,07%	k__Fungi;p__Ascomycota;c__Sordariomycetes;o__Hypocreales;f__Cordycipitaceae	Beauveria
0,05%	0,09%	0,02%	0,05%	0,02%	0,02%	0,05%	0,06%	k__Fungi;p__Ascomycota;c__Dothideomycetes;o__Mytilinidales;f__Gloniaceae	Cenococcum
0,04%	0,05%	0,04%	0,05%	0,04%	0,03%	0,08%	0,04%	k__Fungi;p__Basidiomycota;c__Agaricomycetes;o__Gomphales;f__Gomphaceae	Clavariadelphus
0,01%	0,01%	0,01%	0,32%	0,00%	0,00%	0,01%	0,00%	k__Fungi;p__Basidiomycota;c__Agaricomycetes;o__Hymenochaetales;f__Tubulicrinaceae	Tubulicrinis
0,09%	0,03%	0,02%	0,06%	0,01%	0,01%	0,03%	0,10%	k__Fungi;p__Ascomycota;c__Sordariomycetes;o__Sordariales;f__Lasiosphaeriaceae	unidentified
0,04%	0,03%	0,05%	0,02%	0,01%	0,01%	0,17%	0,01%	k__Fungi;p__Basidiomycota;c__Geminibasidiomycetes;o__Geminibasidiales;f__Geminibasidiaceae	Geminibasidium
0,00%	0,00%	0,00%	0,31%	0,00%	0,00%	0,01%	0,00%	k__Fungi;p__Basidiomycota;c__Agaricomycetes;o__Polyporales;f__Hyphodermataceae	Hyphoderma

0,11%	0,03%	0,01%	0,06%	0,01%	0,02%	0,03%	0,04%	k__Fungi;p__Ascomycota;c__Leotiomycetes;o__Helotiales;f__Sclerotiniaceae;Other	
0,04%	0,04%	0,02%	0,04%	0,04%	0,03%	0,05%	0,05%	k__Fungi;p__Ascomycota;c__Saccharomycetes;o__Saccharomycetales;f__Dipodascaceae	Geotrichum
0,14%	0,02%	0,02%	0,03%	0,01%	0,01%	0,04%	0,04%	k__Fungi;p__Ascomycota;c__Pezizomycetes;o__Pezizales;f__Helvellaceae	Helvella
0,07%	0,04%	0,02%	0,07%	0,01%	0,01%	0,01%	0,08%	k__Fungi;p__Ascomycota;c__Leotiomycetes;o__Helotiales;f__Helotiales_fam_Incertae_sedis	Xenochalara
0,06%	0,02%	0,03%	0,03%	0,02%	0,02%	0,05%	0,07%	k__Fungi;p__Ascomycota;c__Sordariomycetes;o__Calosphaeriales;f__Pleurostomataceae	Pleurostoma
0,03%	0,04%	0,03%	0,04%	0,03%	0,02%	0,04%	0,05%	k__Fungi;p__Ascomycota;c__Saccharomycetes;o__Saccharomycetales;f__Pichiaceae	Pichia
0,01%	0,00%	0,00%	0,01%	0,01%	0,00%	0,02%	0,24%	k__Fungi;p__Olpidiomycota;c__GS17;o__unidentified;f__unidentified	unidentified
0,03%	0,03%	0,03%	0,05%	0,03%	0,02%	0,06%	0,03%	k__Fungi;p__Basidiomycota;c__Agaricomycetes;o__Agaricales;f__Lycoperdaceae	Lycoperdon
0,02%	0,02%	0,04%	0,03%	0,01%	0,08%	0,03%	0,05%	k__Fungi;p__Ascomycota;c__Eurotiomycetes;o__Eurotiales;f__Aspergillaceae	Aspergillus
0,06%	0,01%	0,01%	0,04%	0,01%	0,02%	0,05%	0,05%	k__Fungi;p__Ascomycota;c__Sordariomycetes;o__Hypocreales;f__Nectriaceae	unidentified
0,05%	0,01%	0,02%	0,05%	0,01%	0,06%	0,04%	0,02%	k__Fungi;p__Ascomycota;c__Dothideomycetes;o__Capnodiales;f__Teratosphaeriaceae	Capnobotryella
0,06%	0,03%	0,02%	0,03%	0,01%	0,01%	0,04%	0,05%	k__Fungi;p__Ascomycota;c__Dothideomycetes;o__Pleosporales;f__Sporormiaceae	Westerdykella
0,01%	0,02%	0,01%	0,15%	0,01%	0,00%	0,06%	0,00%	k__Fungi;p__Basidiomycota;c__Agaricomycetes;o__Agaricales;f__Tricholomataceae	Hemimycena
0,01%	0,01%	0,19%	0,01%	0,00%	0,01%	0,01%	0,01%	k__Fungi;p__Basidiomycota;c__Agaricomycetes;o__Agaricales;f__Pluteaceae	Pluteus
0,02%	0,01%	0,03%	0,02%	0,10%	0,00%	0,04%	0,04%	k__Fungi;p__Ascomycota;c__Eurotiomycetes;o__Chaetothyriales;f__Herpotrichiellaceae	Phialophora
0,02%	0,02%	0,02%	0,01%	0,00%	0,00%	0,01%	0,15%	k__Fungi;p__Ascomycota;c__Sordariomycetes;o__Coniochaetales;f__Coniochaetaceae;Other	
0,17%	0,01%	0,03%	0,01%	0,00%	0,00%	0,01%	0,01%	k__Fungi;p__Ascomycota;c__Eurotiomycetes;o__Verrucariales;f__Verrucariaceae	Verrucaria
0,04%	0,01%	0,01%	0,05%	0,02%	0,02%	0,03%	0,05%	k__Fungi;p__Ascomycota;c__Sordariomycetes;o__Hypocreales;Other;Other	
0,03%	0,03%	0,02%	0,02%	0,03%	0,03%	0,05%	0,02%	k__Fungi;p__Basidiomycota;c__Agaricomycetes;o__Hysterangiales;Other;Other	
0,05%	0,01%	0,01%	0,04%	0,03%	0,00%	0,03%	0,06%	k__Fungi;p__Ascomycota;c__Pezizomycetes;o__Pezizales;f__Pyronemataceae	unidentified
0,05%	0,02%	0,01%	0,03%	0,01%	0,02%	0,05%	0,03%	k__Fungi;p__Ascomycota;c__Leotiomycetes;o__Helotiales;f__Helotiaceae	Claussenomyces
0,05%	0,02%	0,02%	0,04%	0,01%	0,01%	0,03%	0,04%	k__Fungi;p__Ascomycota;c__Dothideomycetes;o__Mytilinidiales;f__Mytilinidiaceae	Lophium
0,03%	0,02%	0,01%	0,03%	0,01%	0,02%	0,04%	0,04%	k__Fungi;p__Ascomycota;c__Leotiomycetes;o__Thelebolales;f__Pseudeurotiaceae	Pseudeurotium
0,01%	0,12%	0,01%	0,06%	0,00%	0,01%	0,01%	0,00%	k__Fungi;p__Basidiomycota;c__Agaricomycetes;o__Agaricales;f__Strophariaceae	Galerina
0,04%	0,02%	0,02%	0,03%	0,02%	0,01%	0,04%	0,03%	k__Fungi;p__Ascomycota;c__Saccharomycetes;o__Saccharomycetales;f__Saccharomycetaceae	Williopsis
0,16%	0,00%	0,00%	0,00%	0,01%	0,01%	0,00%	0,01%	k__Fungi;p__Rozellomycota;c__unidentified;o__unidentified;f__unidentified	unidentified
0,00%	0,00%	0,16%	0,01%	0,00%	0,00%	0,00%	0,00%	k__Fungi;p__Ascomycota;c__Lecanoromycetes;o__Teloschistales;f__Teloschistaceae	Xanthocarpia
0,04%	0,01%	0,01%	0,02%	0,02%	0,02%	0,03%	0,04%	k__Fungi;p__Ascomycota;c__Pezizomycetes;o__Pezizales;f__Pyronemataceae	Otidea
0,03%	0,02%	0,01%	0,03%	0,01%	0,00%	0,03%	0,06%	k__Fungi;p__Ascomycota;c__Dothideomycetes;o__Dothideales;f__Dothideaceae	Rhizosphaera
0,03%	0,02%	0,01%	0,02%	0,01%	0,03%	0,04%	0,03%	k__Fungi;p__Ascomycota;c__Leotiomycetes;Other;Other;Other	
0,03%	0,03%	0,01%	0,02%	0,00%	0,01%	0,04%	0,04%	k__Fungi;p__Ascomycota;c__Dothideomycetes;o__Pleosporales;f__Didymellaceae	unidentified
0,01%	0,01%	0,01%	0,01%	0,00%	0,00%	0,13%	0,01%	k__Fungi;p__Ascomycota;c__Leotiomycetes;o__Helotiales;f__Dermateaceae	Calloria
0,03%	0,02%	0,01%	0,03%	0,01%	0,01%	0,04%	0,04%	k__Fungi;p__Ascomycota;c__Dothideomycetes;o__Pleosporales;f__Pleomassariaceae	Prosthemium
0,03%	0,02%	0,02%	0,03%	0,01%	0,01%	0,02%	0,04%	k__Fungi;p__Ascomycota;c__Leotiomycetes;o__Helotiales;f__Helotiaceae	Meliniomyces
0,08%	0,01%	0,03%	0,01%	0,00%	0,04%	0,01%	0,00%	k__Fungi;p__Rozellomycota;c__Rozellomycotina_cls_Incertae_sedis;o__GS11;f__unidentified	unidentified
0,03%	0,02%	0,01%	0,03%	0,01%	0,02%	0,03%	0,03%	k__Fungi;p__Ascomycota;c__Saccharomycetes;o__Saccharomycetales;f__unidentified	unidentified
0,03%	0,03%	0,01%	0,02%	0,00%	0,01%	0,04%	0,04%	k__Fungi;p__Ascomycota;c__Dothideomycetes;o__Pleosporales;f__Melanommataceae;Other	
0,02%	0,01%	0,01%	0,02%	0,00%	0,01%	0,04%	0,06%	k__Fungi;p__Ascomycota;c__Dothideomycetes;o__Pleosporales;f__Leptosphaeriaceae	Plenodomus
0,03%	0,01%	0,03%	0,01%	0,00%	0,01%	0,07%	0,01%	k__Fungi;p__Ascomycota;c__Dothideomycetes;o__Capnodiales;f__Capnodiaceae	Antennariella
0,04%	0,01%	0,01%	0,03%	0,00%	0,00%	0,02%	0,06%	k__Fungi;p__Ascomycota;c__Sordariomycetes;o__Sordariales;f__Sordariaceae	unidentified
0,07%	0,01%	0,01%	0,04%	0,00%	0,00%	0,02%	0,02%	k__Fungi;p__Ascomycota;c__Leotiomycetes;o__Helotiales;f__Helotiales_fam_Incertae_sedis	Xenopolyscytalum
0,01%	0,02%	0,01%	0,08%	0,01%	0,01%	0,04%	0,00%	k__Fungi;p__Basidiomycota;c__Agaricomycetes;o__Agaricales;Other;Other	
0,00%	0,01%	0,15%	0,00%	0,00%	0,00%	0,01%	0,00%	k__Fungi;p__Basidiomycota;c__Agaricomycetes;o__Hymenochaetales;f__Schizoporaceae	Hyphodontia
0,04%	0,01%	0,03%	0,04%	0,00%	0,00%	0,03%	0,01%	k__Fungi;p__Ascomycota;c__Lecanoromycetes;o__Caliciales;f__Physciaceae	Physcia
0,01%	0,00%	0,05%	0,09%	0,00%	0,00%	0,01%	0,01%	k__Fungi;p__Ascomycota;c__Pezizomycetes;o__Pezizales;f__Pezizaceae	Peziza
0,06%	0,00%	0,01%	0,02%	0,01%	0,00%	0,02%	0,04%	k__Fungi;p__Basidiomycota;c__Agaricomycetes;o__Boletales;f__Melanogastraceae	Melanogaster
0,02%	0,03%	0,01%	0,03%	0,01%	0,02%	0,02%	0,01%	k__Fungi;p__Basidiomycota;c__Agaricomycetes;o__Agaricales;f__Omphalotaceae	Rhodocollybia
0,05%	0,01%	0,01%	0,02%	0,02%	0,01%	0,03%	0,02%	k__Fungi;p__Ascomycota;c__Pezizomycetes;o__Pezizales;f__Pyronemataceae	Scutellinia
0,02%	0,01%	0,02%	0,02%	0,01%	0,02%	0,03%	0,04%	k__Fungi;p__Ascomycota;c__Eurotiomycetes;o__Eurotiales;f__Trichocomaceae	Talaromyces
0,02%	0,01%	0,02%	0,08%	0,01%	0,00%	0,02%	0,01%	k__Fungi;p__Ascomycota;c__Sordariomycetes;o__Chaetosphaeriales;f__Chaetosphaeriaceae	Chaetosphaeria
0,04%	0,00%	0,02%	0,04%	0,02%	0,01%	0,01%	0,02%	k__Fungi;p__Ascomycota;c__Leotiomycetes;o__Helotiales;f__Hyaloscyphaceae	Hyaloscypha
0,01%	0,02%	0,05%	0,05%	0,02%	0,01%	0,01%	0,00%	k__Fungi;p__Basidiomycota;c__Agaricomycetes;o__Agaricales;f__Tricholomataceae;Other	
0,01%	0,04%	0,01%	0,06%	0,00%	0,00%	0,01%	0,01%	k__Fungi;p__Ascomycota;c__Pezizomycetes;o__Pezizales;f__Helvellaceae	Barssia
0,05%	0,01%	0,01%	0,03%	0,00%	0,00%	0,02%	0,02%	k__Fungi;p__Basidiomycota;c__Tremellomycetes;o__unidentified;f__unidentified	unidentified
0,03%	0,01%	0,01%	0,02%	0,01%	0,02%	0,02%	0,02%	k__Fungi;p__Ascomycota;c__Saccharomycetes;o__Saccharomycetales;f__Dipodascaceae	unidentified
0,01%	0,00%	0,01%	0,01%	0,01%	0,02%	0,02%	0,08%	k__Fungi;p__Ascomycota;c__Sordariomycetes;o__Hypocreales;f__Cordycipitaceae	Lecanicillium
0,02%	0,01%	0,01%	0,01%	0,04%	0,00%	0,01%	0,05%	k__Fungi;p__Ascomycota;c__Eurotiomycetes;o__Chaetothyriales;f__Herpotrichiellaceae	unidentified
0,05%	0,01%	0,01%	0,03%	0,00%	0,00%	0,02%	0,02%	k__Fungi;p__Ascomycota;c__Sordariomycetes;o__Hypocreales;f__Nectriaceae	Cosmospora
0,01%	0,01%	0,02%	0,02%	0,01%	0,03%	0,02%	0,03%	k__Fungi;p__Ascomycota;c__Eurotiomycetes;o__Eurotiales;f__Aspergillaceae	Monascus
0,00%	0,00%	0,00%	0,01%	0,10%	0,00%	0,00%	0,02%	k__Fungi;p__Ascomycota;c__Sordariomycetes;o__Microascales;f__Microascaceae	Lophotrichus
0,09%	0,00%	0,01%	0,01%	0,01%	0,00%	0,02%	0,01%	k__Fungi;p__Chytridiomycota;c__unidentified;o__unidentified;f__unidentified	unidentified
0,01%	0,01%	0,01%	0,01%	0,00%	0,00%	0,03%	0,07%	k__Fungi;p__Ascomycota;c__Dothideomycetes;o__Pleosporales;f__Cucurbitariaceae	Pyrenochaeta
0,02%	0,06%	0,00%	0,01%	0,02%	0,02%	0,01%	0,00%	k__Fungi;p__Basidiomycota;c__Agaricomycetes;o__Agaricales;f__Tricholomataceae	Paralepista
0,04%	0,01%	0,01%	0,03%	0,00%	0,01%	0,01%	0,03%	k__Fungi;p__Ascomycota;c__Sordariomycetes;o__Sordariales;f__Lasiosphaeriaceae	Apodus

(E)

PI1b	PI2b	PI3b	PI4b	PII1b	PII2b	PII3b	PII4b	taxonomy	
67,57%	9,43%	0,99%	6,39%	0,52%	73,07%	0,57%	29,96%	k__Fungi;p__Basidiomycota;c__Agaricomycetes;o__Russulales;f__Russulaceae	Russula
0,38%	18,81%	47,50%	9,15%	0,33%	4,25%	29,67%	6,27%	k__Fungi;p__Basidiomycota;c__Agaricomycetes;o__Sebacinales;f__Sebacinaceae	Sebacina
0,17%	0,12%	0,09%	57,66%	0,04%	0,07%	0,01%	0,12%	k__Fungi;p__Basidiomycota;c__Agaricomycetes;o__Geastrales;f__Geastraceae	Geastrum
0,04%	0,13%	0,06%	0,04%	0,03%	0,03%	42,07%	0,04%	k__Fungi;p__Basidiomycota;c__Agaricomycetes;o__Gomphales;f__Gomphaceae	Clavariadelphus
0,06%	0,06%	0,06%	0,07%	32,09%	0,05%	0,00%	0,05%	k__Fungi;p__Basidiomycota;c__Agaricomycetes;o__Boletales;f__Boletaceae	Boletus
0,04%	0,04%	0,04%	0,05%	24,19%	0,04%	0,00%	0,04%	k__Fungi;p__Basidiomycota;c__Agaricomycetes;o__Boletales;f__Boletaceae	Neoboletus
0,18%	0,21%	0,27%	0,17%	22,27%	0,13%	0,05%	0,81%	k__Fungi;p__Ascomycota;c__Sordariomycetes;o__Hypocreales;f__unidentified	Hypocreales_unidentified
0,19%	18,00%	1,98%	0,47%	0,16%	1,26%	0,90%	0,18%	k__Fungi;p__Basidiomycota;c__Agaricomycetes;o__Agaricales;f__Inocybaceae	Inocybe
0,20%	0,68%	13,72%	0,58%	0,23%	5,66%	0,58%	0,37%	k__Fungi;p__Basidiomycota;c__Agaricomycetes;o__Agaricales;f__Cortinariaceae	Cortinarius
0,48%	1,64%	2,02%	0,79%	10,48%	0,69%	0,78%	3,54%	k__Fungi;p__Ascomycota;c__Eurotiomycetes;o__Eurotiales;f__Aspergillaceae	Penicillium
4,37%	10,99%	1,27%	0,66%	0,20%	0,09%	0,04%	2,20%	k__Fungi;p__Ascomycota;c__Dothideomycetes;o__Pleosporales;f__Sporormiaceae	Preussia
3,66%	3,00%	1,41%	0,68%	0,17%	0,36%	0,43%	5,70%	k__Fungi;p__Mortierellomycota;c__Mortierellomycetes;o__Mortierellales;f__Mortierellaceae	Mortierella
1,24%	1,95%	3,82%	1,28%	0,43%	1,72%	0,36%	3,65%	k__Fungi;p__unidentified;c__unidentified;o__unidentified;f__unidentified	Fungi_unidentified
0,67%	2,03%	2,08%	0,82%	1,13%	0,79%	0,10%	3,37%	k__Fungi;p__Ascomycota;c__Leotiomycetes;o__Helotiales;f__unidentified	unidentified
0,00%	0,00%	0,01%	0,01%	0,00%	0,00%	10,75%	0,01%	k__Fungi;p__Basidiomycota;c__Agaricomycetes;o__Agaricales;f__Tricholomataceae	Macrocystidia
0,72%	0,57%	0,57%	3,13%	0,31%	2,01%	2,83%	0,18%	k__Fungi;p__Basidiomycota;c__Agaricomycetes;o__Thelephorales;f__Thelephoraceae	unidentified
0,01%	8,31%	0,12%	0,03%	0,02%	0,13%	0,01%	0,03%	k__Fungi;p__Basidiomycota;c__Agaricomycetes;o__Agaricales;f__Hymenogastraceae	Hymenogaster
0,91%	0,86%	0,64%	0,73%	0,12%	0,29%	0,26%	3,10%	k__Fungi;p__Ascomycota;c__Sordariomycetes;o__Sordariales;f__Chaetomiaceae;Other	Chaetomiaceae_Other
0,72%	0,68%	0,48%	0,60%	0,09%	0,22%	0,22%	2,63%	k__Fungi;p__Ascomycota;c__Sordariomycetes;o__Sordariales;f__Chaetomiaceae	Humicola
0,61%	0,45%	0,84%	0,61%	0,18%	0,12%	0,23%	2,02%	k__Fungi;p__Basidiomycota;c__Tremellomycetes;o__Filobasidiales;f__Piskurozymaceae	Solicoccozyma
1,55%	0,55%	0,57%	0,41%	0,12%	0,19%	0,05%	1,11%	k__Fungi;p__Ascomycota;c__Eurotiomycetes;o__Chaetothyriales;f__Herpotrichiellaceae	Exophiala
0,31%	0,77%	0,58%	0,60%	0,13%	0,42%	0,12%	1,51%	k__Fungi;p__Ascomycota;c__Leotiomycetes;o__Helotiales;Other;Other	Helotiales_Other
0,13%	0,29%	0,26%	0,55%	0,33%	0,16%	0,26%	2,41%	k__Fungi;p__Basidiomycota;c__Agaricomycetes;o__Agaricales;f__Tricholomataceae	Tricholoma
0,28%	0,43%	0,35%	0,25%	0,18%	0,20%	0,02%	2,48%	k__Fungi;p__Ascomycota;c__Sordariomycetes;o__Coniochaetales;f__Coniochaetaceae	Coniochaeta
0,03%	0,44%	2,90%	0,24%	0,19%	0,04%	0,05%	0,25%	k__Fungi;p__Ascomycota;c__Eurotiomycetes;o__Eurotiales;f__Trichocomaceae	Sagenomella
0,00%	0,01%	0,00%	0,01%	0,01%	0,01%	0,00%	3,78%	k__Fungi;p__Basidiomycota;c__Agaricomycetes;o__Cantharellales;f__Clavulinaceae	Clavulina
0,11%	0,63%	1,56%	0,29%	0,16%	0,14%	0,20%	0,52%	k__Fungi;p__Ascomycota;c__Leotiomycetes;o__Helotiales;f__Myxotrichaceae	Oidiodendron
1,23%	0,08%	0,92%	0,29%	0,02%	0,05%	0,01%	0,52%	k__Fungi;p__Ascomycota;c__Pezizomycetes;o__Pezizales;f__Tuberaceae	Tuber
1,93%	0,05%	0,03%	0,24%	0,46%	0,02%	0,00%	0,03%	k__Fungi;p__Ascomycota;c__Pezizomycetes;o__Pezizales;f__Pyronemataceae	Wilcoxina

0,11%	0,15%	0,19%	0,10%	0,11%	0,12%	1,80%	0,14%	k__Fungi;p__Basidiomycota;c__Agaricomycetes;o__Trechisporales;f__Hydnodontaceae	Trechispora
0,37%	0,22%	0,20%	0,39%	0,13%	0,99%	0,02%	0,26%	k__Fungi;p__Basidiomycota;c__Agaricomycetes;o__Agaricales;f__Tricholomataceae	Mycena
0,11%	0,40%	0,14%	0,12%	0,41%	0,18%	0,10%	1,01%	k__Fungi;p__Ascomycota;c__Sordariomycetes;o__Hypocreales;f__Hypocreaceae	Trichoderma
0,16%	0,35%	0,73%	0,27%	0,16%	0,12%	0,08%	0,53%	k__Fungi;p__Ascomycota;Other;Other;Other;Other	
0,15%	0,20%	0,21%	0,05%	0,03%	0,03%	0,00%	1,68%	k__Fungi;p__Ascomycota;c__Sordariomycetes;o__Coniochaetales;f__Coniochaetaceae	Lecythophora
0,23%	0,56%	0,23%	0,18%	0,05%	0,07%	0,89%	0,07%	k__Fungi;p__Basidiomycota;c__Agaricomycetes;o__Agaricales;f__Hymenogastraceae	Hebeloma
0,09%	0,95%	0,09%	0,20%	0,01%	0,22%	0,07%	0,50%	k__Fungi;p__Ascomycota;c__Pezizomycetes;o__Pezizales;f__Pyronemataceae	Geopora
0,02%	0,23%	1,07%	0,42%	0,01%	0,20%	0,05%	0,02%	k__Fungi;p__Basidiomycota;Other;Other;Other;Other	
0,08%	0,50%	0,44%	0,60%	0,06%	0,07%	0,08%	0,16%	k__Fungi;p__Ascomycota;c__Leotiomycetes;o__Helotiales;f__Helotiales_fam_Incertae_sedis	Cadophora
1,10%	0,11%	0,11%	0,14%	0,05%	0,29%	0,08%	0,09%	k__Fungi;p__Basidiomycota;c__Agaricomycetes;o__Thelephorales;f__Thelephoraceae	Tomentella
1,43%	0,06%	0,07%	0,06%	0,02%	0,03%	0,13%	0,03%	k__Fungi;p__Basidiomycota;c__Agaricomycetes;o__Sebacinales;f__Sebacinaceae	Helvellosebacina
0,20%	0,24%	0,26%	0,20%	0,02%	0,09%	0,01%	0,77%	k__Fungi;p__Ascomycota;c__Sordariomycetes;o__Hypocreales;f__Nectriaceae	Neonectria
0,26%	0,20%	0,29%	0,10%	0,12%	0,06%	0,03%	0,59%	k__Fungi;Other;Other;Other;Other;Other	
0,10%	0,49%	0,33%	0,15%	0,12%	0,09%	0,01%	0,35%	k__Fungi;p__Basidiomycota;c__Agaricomycetes;o__Agaricales;f__Tricholomataceae	unidentified
0,16%	0,22%	0,28%	0,19%	0,27%	0,14%	0,00%	0,26%	k__Fungi;p__Ascomycota;c__Eurotiomycetes;o__Eurotiales;f__Thermoascaceae	Byssochlamys
0,01%	0,02%	0,01%	1,31%	0,00%	0,01%	0,07%	0,01%	k__Fungi;p__Basidiomycota;c__Agaricomycetes;o__Agaricales;f__Amanitaceae	Amanita
0,07%	0,34%	0,28%	0,18%	0,01%	0,10%	0,02%	0,42%	k__Fungi;p__Ascomycota;c__Dothideomycetes;o__Pleosporales;f__Didymellaceae;Other	
0,04%	1,16%	0,02%	0,10%	0,02%	0,01%	0,02%	0,02%	k__Fungi;p__Ascomycota;c__Pezizomycetes;o__Pezizales;f__Pyronemataceae	Trichophaea
0,06%	0,10%	0,17%	0,09%	0,09%	0,12%	0,07%	0,59%	k__Fungi;p__Ascomycota;c__Leotiomycetes;o__Thelebolales;f__Pseudeurotiaceae	Geomyces
0,07%	0,17%	0,19%	0,21%	0,02%	0,08%	0,07%	0,29%	k__Fungi;p__Ascomycota;c__Leotiomycetes;o__Helotiales;f__Dermateaceae;Other	
0,10%	0,06%	0,09%	0,05%	0,05%	0,09%	0,45%	0,13%	k__Fungi;p__Basidiomycota;c__Agaricomycetes;o__Russulales;f__Lachnocladiaceae	Vararia
0,01%	0,60%	0,03%	0,15%	0,01%	0,01%	0,12%	0,02%	k__Fungi;p__Ascomycota;c__Dothideomycetes;o__Mytilinidales;f__Gloniaceae	Cenococcum
0,10%	0,12%	0,09%	0,09%	0,10%	0,07%	0,03%	0,32%	k__Fungi;p__Ascomycota;c__Sordariomycetes;o__Hypocreales;f__Clavicipitaceae	Metarhizium
0,01%	0,07%	0,66%	0,02%	0,01%	0,01%	0,01%	0,05%	k__Fungi;p__Ascomycota;c__Leotiomycetes;o__Helotiales;f__Myxotrichaceae	unidentified
0,00%	0,47%	0,01%	0,35%	0,00%	0,01%	0,00%	0,01%	k__Fungi;p__Ascomycota;c__Pezizomycetes;o__Pezizales;f__Helvellaceae	Barssia
0,20%	0,10%	0,07%	0,12%	0,07%	0,04%	0,03%	0,23%	k__Fungi;p__Ascomycota;c__Eurotiomycetes;o__Chaetothyriales;f__Herpotrichiellaceae;Other	
0,05%	0,13%	0,16%	0,16%	0,02%	0,10%	0,01%	0,18%	k__Fungi;p__Ascomycota;c__Leotiomycetes;o__Helotiales;f__Dermateaceae	Cryptosporiopsis
0,13%	0,07%	0,11%	0,15%	0,05%	0,04%	0,01%	0,25%	k__Fungi;p__Ascomycota;c__Dothideomycetes;o__Capnodiales;f__unidentified	unidentified
0,07%	0,12%	0,07%	0,06%	0,06%	0,09%	0,07%	0,27%	k__Fungi;p__Ascomycota;c__Dothideomycetes;o__Capnodiales;f__Cladosporiaceae	Cladosporium
0,04%	0,06%	0,11%	0,05%	0,06%	0,09%	0,13%	0,25%	k__Fungi;p__Ascomycota;c__Leotiomycetes;o__Thelebolales;f__Pseudeurotiaceae;Other	
0,02%	0,03%	0,04%	0,54%	0,01%	0,01%	0,07%	0,05%	k__Fungi;p__Ascomycota;c__Sordariomycetes;o__Sordariales;f__unidentified	unidentified
0,07%	0,32%	0,02%	0,06%	0,01%	0,03%	0,00%	0,24%	k__Fungi;p__Ascomycota;c__Sordariomycetes;o__Sordariales;f__Sordariales_fam_Incertae_sedis	Ramophialophora
0,04%	0,07%	0,41%	0,07%	0,04%	0,04%	0,01%	0,06%	k__Fungi;p__Ascomycota;c__unidentified;o__unidentified;f__unidentified	unidentified
0,10%	0,12%	0,14%	0,07%	0,02%	0,04%	0,01%	0,22%	k__Fungi;p__Ascomycota;c__Sordariomycetes;o__Hypocreales;f__Nectriaceae	Fusarium
0,05%	0,23%	0,09%	0,11%	0,04%	0,05%	0,01%	0,14%	k__Fungi;p__Ascomycota;c__Leotiomycetes;o__Helotiales;f__Helotiaceae	Helicodendron
0,07%	0,11%	0,09%	0,11%	0,07%	0,10%	0,00%	0,12%	k__Fungi;p__Basidiomycota;c__Agaricomycetes;o__Agaricales;f__Agaricaceae	Agaricus
0,13%	0,05%	0,07%	0,08%	0,02%	0,01%	0,00%	0,29%	k__Fungi;p__Ascomycota;c__Eurotiomycetes;o__Chaetothyriales;f__unidentified	unidentified
0,24%	0,07%	0,05%	0,08%	0,05%	0,03%	0,01%	0,10%	k__Fungi;p__Ascomycota;c__Eurotiomycetes;o__Chaetothyriales;f__Herpotrichiellaceae	Cladophialophora
0,02%	0,03%	0,02%	0,49%	0,01%	0,03%	0,00%	0,03%	k__Fungi;p__Ascomycota;c__Leotiomycetes;o__unidentified;f__unidentified	unidentified
0,04%	0,05%	0,12%	0,09%	0,06%	0,13%	0,01%	0,10%	k__Fungi;p__Ascomycota;c__Leotiomycetes;o__Thelebolales;f__Pseudeurotiaceae	Pseudogymnoascus
0,15%	0,08%	0,04%	0,10%	0,03%	0,03%	0,01%	0,14%	k__Fungi;p__Ascomycota;c__Sordariomycetes;o__unidentified;f__unidentified	unidentified
0,09%	0,10%	0,07%	0,07%	0,08%	0,07%	0,00%	0,09%	k__Fungi;p__Basidiomycota;c__Agaricomycetes;o__Agaricales;f__Psathyrellaceae	unidentified
0,06%	0,09%	0,12%	0,07%	0,07%	0,07%	0,00%	0,07%	k__Fungi;p__Basidiomycota;c__Agaricomycetes;o__Trechisporales;Other;Other	
0,01%	0,05%	0,15%	0,04%	0,01%	0,02%	0,20%	0,05%	k__Fungi;p__Basidiomycota;c__Agaricomycetes;o__Thelephorales;f__Thelephoraceae	Pseudotomentella
0,06%	0,09%	0,07%	0,08%	0,05%	0,05%	0,00%	0,14%	k__Fungi;p__Ascomycota;c__Sordariomycetes;o__Microascales;f__Microascaceae	Pseudallescheria
0,27%	0,01%	0,01%	0,00%	0,16%	0,00%	0,06%	0,01%	k__Fungi;p__Basidiomycota;c__Agaricomycetes;o__Thelephorales;f__Thelephoraceae	Thelephora
0,05%	0,08%	0,06%	0,08%	0,01%	0,03%	0,03%	0,19%	k__Fungi;p__Ascomycota;c__Sordariomycetes;o__Sordariales;f__Chaetomiaceae	Chaetomium
0,04%	0,05%	0,01%	0,00%	0,02%	0,02%	0,34%	0,00%	k__Fungi;p__Rozellomycota;c__Rozellomycotina_cls_Incertae_sedis;o__GS11;f__unidentified	unidentified
0,04%	0,10%	0,05%	0,11%	0,03%	0,08%	0,00%	0,07%	k__Fungi;p__Ascomycota;c__Leotiomycetes;o__Helotiales;f__Helotiales_fam_Incertae_sedis	Chalara
0,01%	0,07%	0,12%	0,03%	0,05%	0,03%	0,02%	0,10%	k__Fungi;p__Ascomycota;c__Eurotiomycetes;o__Eurotiales;f__Aspergillaceae	Aspergillus
0,03%	0,06%	0,03%	0,04%	0,01%	0,03%	0,00%	0,23%	k__Fungi;p__Ascomycota;c__Sordariomycetes;o__Hypocreales;f__Nectriaceae	Volutella
0,07%	0,16%	0,05%	0,01%	0,02%	0,01%	0,02%	0,07%	k__Fungi;p__Ascomycota;c__Pezizomycetes;o__Pezizales;f__Pyronemataceae	Humaria
0,02%	0,09%	0,01%	0,03%	0,01%	0,03%	0,00%	0,21%	k__Fungi;p__Basidiomycota;c__Tremellomycetes;o__Cystofilobasidiales;f__Mrakiaceae	Tausonia
0,00%	0,01%	0,00%	0,00%	0,00%	0,00%	0,00%	0,35%	k__Fungi;p__Ascomycota;c__Dothideomycetes;o__Dothideomycetes_ord_Incertae_sedis;f__Eremomycetaceae	Eremomyces
0,00%	0,00%	0,00%	0,02%	0,00%	0,00%	0,00%	0,35%	k__Fungi;p__Ascomycota;c__Sordariomycetes;o__Microascales;f__Microascaceae	Lophotrichus
0,00%	0,00%	0,00%	0,01%	0,00%	0,00%	0,35%	0,00%	k__Fungi;p__Ascomycota;c__Sordariomycetes;o__Diaporthales;f__Schizoparmaceae	Coniella
0,04%	0,06%	0,03%	0,03%	0,01%	0,02%	0,05%	0,12%	k__Fungi;p__Ascomycota;c__Sordariomycetes;o__Sordariales;f__Chaetomiaceae	unidentified
0,04%	0,02%	0,01%	0,02%	0,02%	0,16%	0,00%	0,07%	k__Fungi;p__Ascomycota;c__Dothideomycetes;o__Venturiales;f__Venturiaceae;Other	
0,01%	0,06%	0,03%	0,02%	0,01%	0,01%	0,19%	0,01%	k__Fungi;p__Basidiomycota;c__Agaricomycetes;o__unidentified;f__unidentified	unidentified
0,05%	0,03%	0,01%	0,02%	0,02%	0,14%	0,00%	0,05%	k__Fungi;p__Ascomycota;c__Dothideomycetes;o__Venturiales;f__Venturiaceae	Venturia
0,06%	0,06%	0,05%	0,03%	0,02%	0,03%	0,00%	0,10%	k__Fungi;p__Ascomycota;c__Dothideomycetes;o__Pleosporales;f__unidentified	unidentified
0,04%	0,07%	0,02%	0,01%	0,16%	0,02%	0,00%	0,02%	k__Fungi;p__Basidiomycota;c__Agaricomycetes;o__Thelephorales;f__Thelephoraceae	Odontia
0,01%	0,06%	0,00%	0,02%	0,01%	0,13%	0,01%	0,08%	k__Fungi;p__Ascomycota;c__Dothideomycetes;o__Botryosphaeriales;f__unidentified	unidentified
0,03%	0,07%	0,04%	0,02%	0,00%	0,01%	0,00%	0,15%	k__Fungi;p__Ascomycota;c__Sordariomycetes;o__Hypocreales;f__Nectriaceae	Ilyonectria
0,03%	0,02%	0,17%	0,03%	0,01%	0,00%	0,00%	0,05%	k__Fungi;p__Ascomycota;c__Sordariomycetes;o__Sordariales;f__Lasiosphaeriaceae;Other	
0,01%	0,24%	0,00%	0,00%	0,00%	0,03%	0,01%	0,01%	k__Fungi;p__Basidiomycota;c__Agaricomycetes;o__Agaricales;f__Tricholomataceae	Paralepista
0,04%	0,05%	0,04%	0,04%	0,01%	0,02%	0,00%	0,10%	k__Fungi;p__Ascomycota;c__Sordariomycetes;o__Hypocreales;f__Nectriaceae;Other	
0,26%	0,03%	0,00%	0,01%	0,00%	0,00%	0,00%	0,01%	k__Fungi;p__Basidiomycota;c__Agaricomycetes;o__Agaricales;f__Stephanosporaceae	Lindtneria
0,00%	0,17%	0,04%	0,02%	0,02%	0,03%	0,00%	0,02%	k__Fungi;p__Basidiomycota;c__Agaricomycetes;o__Auriculariales;f__Hyaloriaceae	Protodontia
0,00%	0,03%	0,08%	0,09%	0,01%	0,01%	0,00%	0,09%	k__Fungi;p__Ascomycota;c__Eurotiomycetes;o__Onygenales;f__Onygenales_fam_Incertae_sedis	Chrysosporium
0,06%	0,05%	0,04%	0,01%	0,01%	0,01%	0,00%	0,12%	k__Fungi;p__Ascomycota;c__Leotiomycetes;o__Thelebolales;f__Thelebolaceae	Thelebolus
0,03%	0,06%	0,03%	0,02%	0,01%	0,01%	0,00%	0,13%	k__Fungi;p__Basidiomycota;c__Tremellomycetes;o__Holtermanniales;f__Holtermanniales_fam_Incertae_sedis	Holtermanniella
0,02%	0,06%	0,04%	0,02%	0,01%	0,02%	0,08%	0,04%	k__Fungi;p__Basidiomycota;c__Tremellomycetes;o__Tremellales;f__Trimorphomycetaceae	Saitozyma
0,01%	0,03%	0,01%	0,02%	0,01%	0,14%	0,00%	0,04%	k__Fungi;p__Ascomycota;c__Dothideomycetes;o__Venturiales;f__Venturiaceae	unidentified
0,03%	0,12%	0,06%	0,02%	0,01%	0,02%	0,01%	0,00%	k__Fungi;p__Basidiomycota;c__Geminibasidiomycetes;o__Geminibasidiales;f__Geminibasidiaceae	Geminibasidium
0,02%	0,05%	0,04%	0,03%	0,01%	0,02%	0,00%	0,09%	k__Fungi;p__Ascomycota;c__Sordariomycetes;o__Hypocreales;f__Hypocreales_fam_Incertae_sedis	Acremonium
0,03%	0,04%	0,02%	0,01%	0,12%	0,01%	0,00%	0,03%	k__Fungi;p__Ascomycota;c__Sordariomycetes;o__Myrmecridiales;f__unidentified	unidentified
0,01%	0,06%	0,05%	0,02%	0,00%	0,01%	0,01%	0,09%	k__Fungi;p__Ascomycota;c__Pezizomycotina_cls_Incertae_sedis;o__Pezizomycotina_ord_Incertae_sedis;f__Pezizomycotina_fam_Incertae_sedis	Ciliophora
0,02%	0,02%	0,11%	0,02%	0,00%	0,01%	0,00%	0,07%	k__Fungi;p__Ascomycota;c__Sordariomycetes;o__Sordariales;Other;Other	
0,08%	0,04%	0,02%	0,02%	0,01%	0,01%	0,00%	0,07%	k__Fungi;p__Ascomycota;c__Dothideomycetes;o__Pleosporales;Other;Other	
0,03%	0,02%	0,01%	0,06%	0,01%	0,00%	0,00%	0,10%	k__Fungi;p__Ascomycota;c__Pezizomycetes;o__Pezizales;f__Pyronemataceae	unidentified
0,00%	0,00%	0,00%	0,01%	0,00%	0,00%	0,00%	0,22%	k__Fungi;p__Ascomycota;c__Sordariomycetes;o__Microascales;f__Microascaceae	Kernia
0,02%	0,02%	0,04%	0,03%	0,01%	0,04%	0,01%	0,08%	k__Fungi;p__Ascomycota;c__Sordariomycetes;Other;Other;Other	
0,03%	0,05%	0,04%	0,04%	0,03%	0,02%	0,00%	0,03%	k__Fungi;p__Ascomycota;c__Saccharomycetes;o__Saccharomycetales;f__Dipodascaceae	Geotrichum
0,01%	0,02%	0,02%	0,03%	0,10%	0,03%	0,00%	0,03%	k__Fungi;p__Ascomycota;c__Leotiomycetes;o__Thelebolales;f__Pseudeurotiaceae	Pseudeurotium
0,00%	0,01%	0,00%	0,14%	0,00%	0,00%	0,00%	0,06%	k__Fungi;p__Basidiomycota;c__Cystobasidiomycetes;o__Erythrobasidiales;f__Erythrobasidiales_fam_Incertae_sedis	Sakaguchia
0,00%	0,01%	0,01%	0,01%	0,00%	0,00%	0,00%	0,18%	k__Fungi;p__Basidiomycota;c__Agaricomycetes;o__Agaricales;f__Tricholomataceae	Melanoleuca
0,01%	0,03%	0,04%	0,03%	0,01%	0,01%	0,02%	0,05%	k__Fungi;p__Basidiomycota;c__Agaricomycetes;o__Agaricales;f__Lycoperdaceae	Lycoperdon
0,07%	0,03%	0,02%	0,02%	0,01%	0,02%	0,00%	0,03%	k__Fungi;p__Ascomycota;c__Pezizomycetes;o__Pezizales;f__Sarcoscyphaceae	Desmazierella
0,02%	0,05%	0,02%	0,08%	0,01%	0,01%	0,00%	0,01%	k__Fungi;p__Ascomycota;c__Leotiomycetes;o__Helotiales;f__Hyaloscyphaceae	unidentified
0,01%	0,07%	0,04%	0,01%	0,01%	0,02%	0,00%	0,03%	k__Fungi;p__Ascomycota;c__Dothideomycetes;o__Pleosporales;f__Pleosporaceae	Alternaria
0,02%	0,04%	0,03%	0,03%	0,02%	0,03%	0,00%	0,02%	k__Fungi;p__Basidiomycota;c__Agaricomycetes;o__Hysterangiales;Other;Other	

0,01%	0,02%	0,02%	0,01%	0,01%	0,01%	0,00%	0,12%	k__Fungi;p__Ascomycota;c__Pezizomycetes;o__Pezizales;f__Pyronemataceae	Otidea
0,03%	0,01%	0,04%	0,04%	0,01%	0,00%	0,00%	0,05%	k__Fungi;p__Basidiomycota;c__Agaricomycetes;o__Boletales;f__Melanogastraceae	Melanogaster
0,01%	0,02%	0,10%	0,02%	0,01%	0,01%	0,01%	0,01%	k__Fungi;p__Ascomycota;c__Leotiomycetes;o__Helotiales;f__Helotiaceae	Tetracladium
0,02%	0,02%	0,03%	0,03%	0,04%	0,02%	0,00%	0,03%	k__Fungi;p__Ascomycota;c__Saccharomycetes;o__Saccharomycetales;f__Pichiaceae	Pichia
0,01%	0,01%	0,01%	0,00%	0,14%	0,00%	0,00%	0,01%	k__Fungi;p__Basidiomycota;c__Agaricomycetes;o__Agaricales;f__Lyophyllaceae	Tephrocybe
0,01%	0,01%	0,05%	0,01%	0,01%	0,06%	0,02%	0,02%	k__Fungi;p__Ascomycota;c__Sordariomycetes;o__Hypocreales;f__Cordycipitaceae	Beauveria
0,17%	0,00%	0,00%	0,00%	0,00%	0,00%	0,00%	0,01%	k__Fungi;p__Basidiomycota;c__Agaricomycetes;o__Russulales;f__Russulaceae	unidentified
0,00%	0,00%	0,00%	0,00%	0,00%	0,00%	0,00%	0,17%	k__Fungi;p__Ascomycota;c__Leotiomycetes;o__Phacidiales;f__Phacidiaceae;Other	
0,03%	0,02%	0,01%	0,02%	0,01%	0,01%	0,01%	0,08%	k__Fungi;p__Ascomycota;c__Eurotiomycetes;o__Chaetothyriales;f__Herpotrichiellaceae	Capronia
0,03%	0,02%	0,02%	0,02%	0,02%	0,01%	0,01%	0,03%	k__Fungi;p__Basidiomycota;c__Agaricomycetes;o__Tremellodendropsidales;f__unidentified	unidentified
0,01%	0,05%	0,01%	0,01%	0,00%	0,01%	0,00%	0,08%	k__Fungi;p__Basidiomycota;c__Agaricomycetes;o__Agaricales;f__Tricholomataceae	Mycenella
0,01%	0,03%	0,01%	0,02%	0,02%	0,01%	0,01%	0,07%	k__Fungi;p__Ascomycota;c__Sordariomycetes;o__Microascales;f__Microascaceae	Cephalotrichum
0,08%	0,02%	0,02%	0,01%	0,01%	0,01%	0,00%	0,02%	k__Fungi;p__Ascomycota;c__Pezizomycetes;o__Pezizales;f__Helvellaceae	Helvella
0,04%	0,02%	0,01%	0,04%	0,01%	0,01%	0,00%	0,05%	k__Fungi;p__Ascomycota;c__Eurotiomycetes;o__Chaetothyriales;Other;Other	
0,02%	0,05%	0,03%	0,02%	0,00%	0,01%	0,00%	0,02%	k__Fungi;p__Ascomycota;c__Dothideomycetes;o__Pleosporales;f__Sporormiaceae	Westerdykella
0,04%	0,02%	0,03%	0,03%	0,00%	0,01%	0,01%	0,02%	k__Fungi;p__Chytridiomycota;Other;Other;Other;Other	
0,01%	0,04%	0,02%	0,04%	0,01%	0,01%	0,00%	0,02%	k__Fungi;p__Ascomycota;c__Leotiomycetes;o__Helotiales;f__Helotiaceae	Meliniomyces
0,02%	0,05%	0,02%	0,04%	0,01%	0,00%	0,00%	0,01%	k__Fungi;p__Ascomycota;c__Leotiomycetes;o__Rhytismatales;f__Rhytismataceae	Lophodermium
0,02%	0,02%	0,02%	0,02%	0,01%	0,01%	0,00%	0,05%	k__Fungi;p__Ascomycota;c__Sordariomycetes;o__Calosphaeriales;f__Pleurostomataceae	Pleurostoma
0,01%	0,03%	0,02%	0,02%	0,00%	0,01%	0,01%	0,04%	k__Fungi;p__Ascomycota;c__Dothideomycetes;Other;Other;Other	
0,01%	0,03%	0,02%	0,02%	0,01%	0,01%	0,00%	0,04%	k__Fungi;p__Basidiomycota;c__Agaricomycetes;o__Agaricales;f__Tricholomataceae	Pseudoclitocybe
0,00%	0,02%	0,05%	0,01%	0,02%	0,01%	0,01%	0,01%	k__Fungi;p__Ascomycota;c__Eurotiomycetes;o__Eurotiales;f__Trichocomaceae	Talaromyces
0,03%	0,04%	0,02%	0,01%	0,00%	0,01%	0,00%	0,03%	k__Fungi;p__Basidiomycota;c__Tremellomycetes;o__unidentified;f__unidentified	unidentified
0,03%	0,05%	0,02%	0,02%	0,00%	0,01%	0,00%	0,01%	k__Fungi;p__Basidiomycota;c__Agaricomycetes;o__Agaricales;f__Bolbitiaceae	Conocybe
0,02%	0,02%	0,02%	0,02%	0,01%	0,01%	0,00%	0,04%	k__Fungi;p__Ascomycota;c__Dothideomycetes;o__Dothideales;f__Aureobasidiaceae	Aureobasidium
0,02%	0,02%	0,01%	0,01%	0,01%	0,01%	0,00%	0,05%	k__Fungi;p__Ascomycota;c__Dothideomycetes;o__Pleosporales;f__Melanommataceae	unidentified
0,01%	0,02%	0,01%	0,01%	0,00%	0,01%	0,05%	0,01%	k__Fungi;p__Ascomycota;c__Saccharomycetes;o__Saccharomycetales;f__unidentified	unidentified
0,00%	0,02%	0,05%	0,01%	0,00%	0,03%	0,01%	0,01%	k__Fungi;p__Basidiomycota;c__Agaricomycetes;Other;Other;Other	
0,01%	0,05%	0,00%	0,01%	0,04%	0,01%	0,00%	0,01%	k__Fungi;p__Ascomycota;c__Leotiomycetes;o__Helotiales;f__Helotiales_fam_Incertae_sedis	Xenochalara
0,01%	0,01%	0,03%	0,03%	0,01%	0,00%	0,00%	0,03%	k__Fungi;p__Ascomycota;c__Sordariomycetes;o__Sordariales;f__Lasiosphaeriaceae	unidentified
0,00%	0,01%	0,01%	0,01%	0,00%	0,00%	0,08%	0,00%	k__Fungi;p__Basidiomycota;c__Tremellomycetes;o__Trichosporonales;f__Trichosporonaceae	Cutaneotrichosporon
0,01%	0,02%	0,01%	0,01%	0,00%	0,01%	0,01%	0,03%	k__Fungi;p__Ascomycota;c__Dothideomycetes;o__unidentified;f__unidentified	unidentified
0,00%	0,00%	0,01%	0,03%	0,00%	0,02%	0,00%	0,06%	k__Fungi;p__Ascomycota;c__Lecanoromycetes;o__GS36;f__unidentified	unidentified
0,01%	0,01%	0,03%	0,02%	0,02%	0,01%	0,00%	0,02%	k__Fungi;p__Ascomycota;c__Eurotiomycetes;o__Eurotiales;f__Aspergillaceae	Monascus
0,00%	0,02%	0,01%	0,01%	0,01%	0,01%	0,04%	0,01%	k__Fungi;p__Basidiomycota;c__Tremellomycetes;o__Trichosporonales;f__Trichosporonaceae	Apiotrichum
0,03%	0,03%	0,01%	0,00%	0,00%	0,00%	0,01%	0,03%	k__Fungi;p__Ascomycota;c__Sordariomycetes;o__Coniochaetales;f__unidentified	unidentified
0,03%	0,01%	0,01%	0,01%	0,02%	0,01%	0,03%	0,01%	k__Fungi;p__Basidiomycota;c__Agaricomycetes;o__Thelephorales;f__Thelephoraceae;Other	
0,01%	0,01%	0,01%	0,01%	0,00%	0,01%	0,00%	0,04%	k__Fungi;p__Ascomycota;c__Sordariomycetes;o__Hypocreales;f__Nectriaceae	unidentified
0,00%	0,01%	0,01%	0,01%	0,00%	0,01%	0,06%	0,01%	k__Fungi;p__Mucoromycota;c__Umbelopsidomycetes;o__Umbelopsidales;f__Umbelopsidaceae	Umbelopsis
0,01%	0,01%	0,00%	0,02%	0,00%	0,00%	0,00%	0,07%	k__Fungi;p__Basidiomycota;c__Agaricomycetes;o__Agaricales;f__Hydnangiaceae	Laccaria
0,02%	0,02%	0,02%	0,01%	0,02%	0,01%	0,00%	0,02%	k__Fungi;p__Ascomycota;c__Saccharomycetes;o__Saccharomycetales;f__Saccharomycetaceae	Williopsis
0,02%	0,01%	0,01%	0,02%	0,02%	0,01%	0,00%	0,01%	k__Fungi;p__Ascomycota;c__Saccharomycetes;o__Saccharomycetales;f__Dipodascaceae	unidentified
0,00%	0,01%	0,01%	0,01%	0,00%	0,03%	0,00%	0,05%	k__Fungi;p__Ascomycota;c__Dothideomycetes;o__Mytilinidiales;f__Mytilinidiales_fam_Incertae_sedis	Halokirschsteiniothelia
0,00%	0,00%	0,00%	0,00%	0,00%	0,00%	0,09%	0,00%	k__Fungi;p__Basidiomycota;c__Agaricomycetes;o__Agaricales;f__Hygrophoraceae	unidentified
0,00%	0,01%	0,00%	0,09%	0,00%	0,00%	0,00%	0,00%	k__Fungi;p__Ascomycota;c__Pezizomycetes;o__Pezizales;f__Pyronemataceae	Pustularia
0,03%	0,01%	0,01%	0,02%	0,01%	0,01%	0,00%	0,02%	k__Fungi;p__Ascomycota;c__Pezizomycetes;o__Pezizales;f__Pyronemataceae	Scutellinia
0,01%	0,03%	0,01%	0,01%	0,00%	0,04%	0,00%	0,01%	k__Fungi;p__Ascomycota;c__Leotiomycetes;Other;Other;Other	
0,00%	0,01%	0,01%	0,01%	0,00%	0,06%	0,00%	0,01%	k__Fungi;p__Ascomycota;c__Leotiomycetes;o__Helotiales;f__Myxotrichaceae	Myxotrichum
0,01%	0,03%	0,03%	0,01%	0,01%	0,01%	0,00%	0,01%	k__Fungi;p__Ascomycota;c__Eurotiomycetes;o__Onygenales;f__Onygenaceae	Auxarthron
0,01%	0,02%	0,00%	0,01%	0,00%	0,00%	0,00%	0,05%	k__Fungi;p__Basidiomycota;c__Agaricomycetes;o__Agaricales;f__unidentified	unidentified
0,00%	0,02%	0,01%	0,00%	0,00%	0,00%	0,00%	0,05%	k__Fungi;p__Ascomycota;c__Sordariomycetes;o__Coniochaetales;f__Coniochaetaceae;Other	
0,00%	0,01%	0,00%	0,00%	0,00%	0,00%	0,00%	0,08%	k__Fungi;p__Ascomycota;c__Sordariomycetes;o__Hypocreales;f__Bionectriaceae	Nectriopsis
0,01%	0,01%	0,02%	0,01%	0,01%	0,01%	0,00%	0,03%	k__Fungi;p__Ascomycota;c__Dothideomycetes;o__Dothideales;f__Dothioraceae	Hormonema
0,02%	0,01%	0,02%	0,01%	0,00%	0,01%	0,00%	0,02%	k__Fungi;p__Ascomycota;c__Dothideomycetes;o__Capnodiales;f__Teratosphaeriaceae	Capnobotryella
0,01%	0,01%	0,01%	0,01%	0,00%	0,01%	0,00%	0,05%	k__Fungi;p__Ascomycota;c__Dothideomycetes;o__Pleosporales;f__Melanommataceae;Other	
0,03%	0,01%	0,02%	0,01%	0,01%	0,00%	0,00%	0,01%	k__Fungi;p__Ascomycota;c__Dothideomycetes;o__Pleosporales;f__Didymellaceae	Phoma
0,01%	0,02%	0,02%	0,01%	0,00%	0,01%	0,00%	0,01%	k__Fungi;p__Basidiomycota;c__Microbotryomycetes;o__Sporidiobolales;f__Sporidiobolaceae	Rhodotorula
0,01%	0,02%	0,01%	0,01%	0,00%	0,00%	0,00%	0,03%	k__Fungi;p__Chytridiomycota;c__unidentified;o__unidentified;f__unidentified	unidentified
0,01%	0,01%	0,01%	0,01%	0,00%	0,01%	0,00%	0,03%	k__Fungi;p__Ascomycota;c__Sordariomycetes;o__Hypocreales;Other;Other	
0,00%	0,02%	0,00%	0,00%	0,00%	0,00%	0,00%	0,04%	k__Fungi;p__Ascomycota;c__Leotiomycetes;o__Thelebolales;f__Pseudeurotiaceae	Gymnostellatospora
0,00%	0,00%	0,06%	0,00%	0,00%	0,00%	0,00%	0,01%	k__Fungi;p__Ascomycota;c__Pezizomycetes;o__Pezizales;f__Pezizaceae	Peziza
0,01%	0,02%	0,02%	0,01%	0,01%	0,01%	0,00%	0,00%	k__Fungi;p__Basidiomycota;c__Agaricomycetes;o__Agaricales;f__Omphalotaceae	Rhodocollybia
0,01%	0,01%	0,01%	0,01%	0,00%	0,00%	0,01%	0,02%	k__Fungi;p__Ascomycota;c__Sordariomycetes;o__Hypocreales;f__Nectriaceae	Gibberella
0,00%	0,00%	0,01%	0,00%	0,01%	0,01%	0,01%	0,05%	k__Fungi;p__Basidiomycota;c__Agaricomycetes;o__Cantharellales;f__Clavulinaceae	Membranomyces
0,00%	0,01%	0,02%	0,01%	0,00%	0,01%	0,00%	0,03%	k__Fungi;p__Ascomycota;c__Sordariomycetes;o__Sordariales;f__Lasiosphaeriaceae	Apodus
0,01%	0,02%	0,01%	0,01%	0,00%	0,00%	0,00%	0,02%	k__Fungi;p__Ascomycota;c__Dothideomycetes;o__Pleosporales;f__Pleomassariaceae	Prosthemium
0,04%	0,02%	0,01%	0,01%	0,00%	0,00%	0,00%	0,00%	k__Fungi;p__Ascomycota;c__Leotiomycetes;o__Helotiales;f__Helotiales_fam_Incertae_sedis	Leohumicola
0,00%	0,00%	0,00%	0,06%	0,00%	0,00%	0,00%	0,00%	k__Fungi;p__Ascomycota;c__Archaeorhizomycetes;o__Archaeorhizomycetales;f__Archaeorhizomycetaceae	Archaeorhizomyces
0,01%	0,01%	0,01%	0,02%	0,01%	0,01%	0,00%	0,01%	k__Fungi;p__Basidiomycota;c__Agaricomycetes;o__Geastrales;f__Sclerogastraceae	Sclerogaster
0,01%	0,01%	0,01%	0,02%	0,01%	0,00%	0,00%	0,01%	k__Fungi;p__Ascomycota;c__Dothideomycetes;o__Capnodiales;Other;Other	
0,01%	0,01%	0,01%	0,01%	0,00%	0,01%	0,00%	0,03%	k__Fungi;p__Ascomycota;c__Sordariomycetes;o__Hypocreales;f__Clavicipitaceae	Metapochonia
0,00%	0,02%	0,02%	0,00%	0,01%	0,01%	0,00%	0,01%	k__Fungi;p__Ascomycota;c__Eurotiomycetes;o__Onygenales;f__Ajellomycetaceae	Histoplasma
0,01%	0,03%	0,01%	0,00%	0,00%	0,00%	0,00%	0,01%	k__Fungi;p__Mortierellomycota;c__Mortierellomycetes;o__Mortierellales;f__Mortierellaceae	unidentified
0,00%	0,00%	0,00%	0,00%	0,00%	0,00%	0,01%	0,04%	k__Fungi;p__Rozellomycota;c__unidentified;o__unidentified;f__unidentified	unidentified
0,03%	0,00%	0,00%	0,00%	0,01%	0,00%	0,02%	0,01%	k__Fungi;p__Basidiomycota;c__Agaricomycetes;o__Agaricales;f__Lyophyllaceae	Lyophyllum
0,02%	0,01%	0,01%	0,01%	0,00%	0,00%	0,00%	0,01%	k__Fungi;p__Ascomycota;c__Sordariomycetes;o__Hypocreales;f__Nectriaceae	Cosmospora
0,01%	0,01%	0,01%	0,01%	0,00%	0,01%	0,00%	0,02%	k__Fungi;p__Ascomycota;c__Dothideomycetes;o__Venturiales;Other;Other	
0,00%	0,00%	0,00%	0,00%	0,00%	0,00%	0,00%	0,06%	k__Fungi;p__Basidiomycota;c__Agaricomycetes;o__Agaricales;f__Agaricaceae	Cystolepiota
0,01%	0,01%	0,01%	0,00%	0,01%	0,01%	0,00%	0,02%	k__Fungi;p__Basidiomycota;c__Agaricomycetes;o__Agaricales;f__Agaricaceae	Lepiota

(F)

PI1c	PI2c	PI3c	PI4c	PII1c	PII2c	PII3c	PII4c	taxonomy	
91,79%	49,41%	0,56%	1,44%	2,08%	67,13%	3,84%	58,78%	k__Fungi;p__Basidiomycota;c__Agaricomycetes;o__Russulales;f__Russulaceae	Russula
0,13%	1,08%	11,48%	73,71%	0,94%	1,30%	1,62%	11,88%	k__Fungi;p__Basidiomycota;c__Agaricomycetes;o__Agaricales;f__Cortinariaceae	Cortinarius
0,24%	27,04%	17,41%	3,71%	0,24%	6,36%	2,18%	13,71%	k__Fungi;p__Basidiomycota;c__Agaricomycetes;o__Sebacinales;f__Sebacinaceae	Sebacina
0,03%	0,17%	0,04%	0,04%	0,03%	0,04%	61,52%	0,04%	k__Fungi;p__Basidiomycota;c__Agaricomycetes;o__Gomphales;f__Gomphaceae	Clavariadelphus
0,08%	0,10%	0,08%	0,07%	31,04%	0,08%	0,09%	0,16%	k__Fungi;p__Ascomycota;c__Sordariomycetes;o__Hypocreales;f__unidentified	Hypocreales_unidentified
0,05%	0,06%	28,40%	0,08%	0,07%	0,06%	0,06%	0,11%	k__Fungi;p__Basidiomycota;c__Agaricomycetes;o__Trechisporales;Other;Other	Trechisporales_Other
0,08%	0,11%	24,85%	0,13%	0,11%	0,10%	0,12%	0,15%	k__Fungi;p__Basidiomycota;c__Agaricomycetes;o__Trechisporales;f__Hydnodontaceae	Trechispora
0,04%	0,06%	0,06%	0,05%	23,77%	0,05%	0,05%	0,08%	k__Fungi;p__Basidiomycota;c__Agaricomycetes;o__Boletales;f__Boletaceae	Boletus
0,03%	0,14%	0,25%	0,09%	0,05%	0,06%	20,48%	0,12%	k__Fungi;p__Basidiomycota;c__Agaricomycetes;o__Agaricales;f__Hymenogastraceae	Hebeloma
0,02%	0,03%	0,02%	0,02%	0,02%	20,16%	0,05%	0,03%	k__Fungi;p__Basidiomycota;c__Agaricomycetes;o__Hysterangiales;Other;Other	Hysterangiales_Other
0,03%	0,04%	0,05%	0,04%	18,04%	0,03%	0,04%	0,05%	k__Fungi;p__Basidiomycota;c__Agaricomycetes;o__Boletales;f__Boletaceae	Neoboletus
0,22%	0,50%	0,31%	0,15%	16,26%	0,15%	0,33%	0,34%	k__Fungi;p__Ascomycota;c__Eurotiomycetes;o__Eurotiales;f__Aspergillaceae	Penicillium

0,05%	0,06%	0,11%	13,81%	0,06%	0,10%	0,15%	0,07%	k__Fungi;p__Basidiomycota;c__Agaricomycetes;o__Geastrales;f__Geastraceae	Geastrum
0,13%	4,97%	7,92%	0,20%	0,18%	0,22%	0,47%	0,15%	k__Fungi;p__Basidiomycota;c__Agaricomycetes;o__Agaricales;f__Inocybaceae	Inocybe
0,10%	0,21%	0,14%	0,15%	0,15%	0,11%	0,49%	3,16%	k__Fungi;p__Ascomycota;c__Sordariomycetes;o__Coniochaetales;f__Coniochaetaceae	Coniochaeta
0,43%	0,90%	0,48%	0,21%	0,31%	0,10%	0,29%	1,38%	k__Fungi;p__unidentified;c__unidentified;o__unidentified;f__unidentified	Fungi_unidentified
0,10%	0,31%	0,51%	0,73%	0,38%	0,32%	1,03%	0,51%	k__Fungi;p__Basidiomycota;c__Agaricomycetes;o__Agaricales;f__Tricholomataceae	Tricholoma
0,04%	0,23%	0,07%	0,47%	0,10%	0,02%	1,80%	0,03%	k__Fungi;p__Ascomycota;c__Leotiomycetes;o__Helotiales;f__Helotiales_fam_Incertae_sedis	Cadophora
0,01%	0,44%	0,96%	0,03%	0,02%	0,65%	0,11%	0,22%	k__Fungi;p__Basidiomycota;c__Agaricomycetes;o__Agaricales;f__Hymenogastraceae	Hymenogaster
0,10%	0,65%	0,30%	0,17%	0,19%	0,16%	0,31%	0,37%	k__Fungi;p__Ascomycota;c__Leotiomycetes;o__Helotiales;f__unidentified	Helotiales_unidentified
0,03%	0,43%	0,34%	0,18%	0,26%	0,04%	0,47%	0,26%	k__Fungi;p__Ascomycota;c__Leotiomycetes;o__Helotiales;f__Myxotrichaceae	Oidiodendron
0,14%	1,00%	0,11%	0,11%	0,06%	0,08%	0,13%	0,36%	k__Fungi;p__Mortierellomycota;c__Mortierellomycetes;o__Mortierellales;f__Mortierellaceae	Mortierella
0,01%	0,31%	0,18%	0,13%	0,89%	0,03%	0,18%	0,06%	k__Fungi;p__Ascomycota;c__Eurotiomycetes;o__Eurotiales;f__Trichocomaceae	Sagenomella
0,03%	0,12%	0,03%	0,03%	1,15%	0,03%	0,04%	0,18%	k__Fungi;p__Ascomycota;c__Sordariomycetes;o__Hypocreales;f__Hypocreaceae	Trichoderma
0,29%	0,33%	0,16%	0,21%	0,11%	0,14%	0,20%	0,13%	k__Fungi;p__Basidiomycota;c__Agaricomycetes;o__Thelephorales;f__Thelephoraceae	unidentified
0,09%	1,36%	0,01%	0,05%	0,01%	0,01%	0,01%	0,01%	k__Fungi;p__Ascomycota;c__Pezizomycetes;o__Pezizales;f__Pyronemataceae	Trichophaea
0,08%	0,86%	0,04%	0,15%	0,02%	0,08%	0,09%	0,18%	k__Fungi;p__Ascomycota;c__Pezizomycetes;o__Pezizales;f__Pyronemataceae	Geopora
0,08%	0,37%	0,46%	0,06%	0,05%	0,04%	0,06%	0,32%	k__Fungi;p__Ascomycota;c__Dothideomycetes;o__Pleosporales;f__Sporormiaceae	Preussia
0,09%	0,17%	0,14%	0,10%	0,26%	0,13%	0,14%	0,14%	k__Fungi;p__Ascomycota;c__Eurotiomycetes;o__Eurotiales;f__Thermoascaceae	Byssochlamys
0,01%	0,01%	0,01%	0,01%	0,01%	0,01%	0,01%	1,06%	k__Fungi;p__Ascomycota;c__Pezizomycetes;o__Pezizales;f__Pyronemataceae	Otidea
0,00%	0,00%	0,01%	0,00%	0,00%	0,00%	0,01%	0,93%	k__Fungi;p__Basidiomycota;c__Agaricomycetes;o__Agaricales;f__Hydnangiaceae	Laccaria
0,31%	0,06%	0,19%	0,02%	0,01%	0,01%	0,02%	0,32%	k__Fungi;p__Ascomycota;c__Pezizomycetes;o__Pezizales;f__Tuberaceae	Tuber
0,16%	0,34%	0,07%	0,06%	0,04%	0,04%	0,07%	0,12%	k__Fungi;p__Ascomycota;c__Eurotiomycetes;o__Chaetothyriales;f__Herpotrichiellaceae	Exophiala
0,03%	0,16%	0,13%	0,08%	0,08%	0,03%	0,17%	0,18%	k__Fungi;p__Ascomycota;Other;Other;Other;Other	
0,06%	0,30%	0,07%	0,09%	0,05%	0,03%	0,05%	0,20%	k__Fungi;p__Ascomycota;c__Sordariomycetes;o__Sordariales;f__Chaetomiaceae;Other	
0,07%	0,14%	0,15%	0,10%	0,08%	0,08%	0,12%	0,09%	k__Fungi;p__Basidiomycota;c__Agaricomycetes;o__Agaricales;f__Tricholomataceae	Mycena
0,63%	0,01%	0,01%	0,03%	0,10%	0,01%	0,02%	0,02%	k__Fungi;p__Ascomycota;c__Pezizomycetes;o__Pezizales;f__Pyronemataceae	Wilcoxina
0,60%	0,14%	0,01%	0,00%	0,01%	0,01%	0,01%	0,02%	k__Fungi;p__Ascomycota;c__Pezizomycetes;o__Pezizales;f__Pyronemataceae	Humaria
0,01%	0,10%	0,18%	0,20%	0,02%	0,02%	0,18%	0,07%	k__Fungi;p__Ascomycota;c__Dothideomycetes;o__Mytilinidales;f__Gloniaceae	Cenococcum
0,00%	0,59%	0,02%	0,04%	0,00%	0,03%	0,06%	0,00%	k__Fungi;p__Ascomycota;c__Pezizomycetes;o__Pezizales;f__Helvellaceae	Barssia
0,05%	0,08%	0,13%	0,12%	0,09%	0,07%	0,12%	0,09%	k__Fungi;p__Basidiomycota;c__Agaricomycetes;o__Agaricales;f__Agaricaceae	Agaricus
0,06%	0,17%	0,07%	0,07%	0,06%	0,10%	0,11%	0,09%	k__Fungi;p__Ascomycota;c__Leotiomycetes;o__Helotiales;Other;Other	
0,55%	0,07%	0,01%	0,01%	0,03%	0,01%	0,01%	0,01%	k__Fungi;p__Ascomycota;c__Eurotiomycetes;o__Chaetothyriales;f__Herpotrichiellaceae	Cladophialophora
0,05%	0,07%	0,11%	0,10%	0,07%	0,08%	0,11%	0,06%	k__Fungi;p__Basidiomycota;c__Agaricomycetes;o__Agaricales;f__Psathyrellaceae	unidentified
0,46%	0,04%	0,04%	0,02%	0,01%	0,02%	0,02%	0,02%	k__Fungi;p__Basidiomycota;c__Agaricomycetes;o__Sebacinales;f__Sebacinaceae	Helvellosebacina
0,00%	0,01%	0,01%	0,01%	0,01%	0,01%	0,05%	0,54%	k__Fungi;p__Ascomycota;c__Sordariomycetes;Other;Other;Other	
0,04%	0,23%	0,05%	0,08%	0,03%	0,02%	0,04%	0,15%	k__Fungi;p__Ascomycota;c__Sordariomycetes;o__Sordariales;f__Chaetomiaceae	Humicola
0,07%	0,08%	0,06%	0,07%	0,05%	0,07%	0,07%	0,08%	k__Fungi;p__Basidiomycota;c__Agaricomycetes;o__Russulales;f__Lachnocladiaceae	Vararia
0,03%	0,12%	0,08%	0,13%	0,03%	0,03%	0,05%	0,07%	k__Fungi;p__Basidiomycota;c__Tremellomycetes;o__Filobasidiales;f__Piskurozymaceae	Solicoccozyma
0,12%	0,11%	0,04%	0,04%	0,04%	0,05%	0,05%	0,07%	k__Fungi;p__Basidiomycota;c__Agaricomycetes;o__Thelephorales;f__Thelephoraceae	Tomentella
0,07%	0,03%	0,07%	0,05%	0,04%	0,03%	0,03%	0,07%	k__Fungi;p__Basidiomycota;c__Agaricomycetes;o__Agaricales;f__Tricholomataceae	unidentified
0,00%	0,06%	0,04%	0,04%	0,01%	0,01%	0,16%	0,06%	k__Fungi;p__Ascomycota;c__Leotiomycetes;o__Helotiales;f__Myxotrichaceae	unidentified
0,03%	0,06%	0,05%	0,04%	0,05%	0,04%	0,05%	0,05%	k__Fungi;p__Ascomycota;c__Sordariomycetes;o__Microascales;f__Microascaceae	Pseudallescheria
0,00%	0,00%	0,00%	0,00%	0,33%	0,00%	0,00%	0,02%	k__Fungi;p__Basidiomycota;c__Agaricomycetes;o__Atheliales;f__Atheliaceae	Piloderma
0,03%	0,06%	0,03%	0,03%	0,06%	0,03%	0,05%	0,05%	k__Fungi;p__Ascomycota;c__Sordariomycetes;o__Hypocreales;f__Clavicipitaceae	Metarhizium
0,00%	0,03%	0,01%	0,21%	0,01%	0,01%	0,02%	0,01%	k__Fungi;p__Basidiomycota;c__Agaricomycetes;o__Agaricales;f__Amanitaceae	Amanita
0,06%	0,16%	0,01%	0,01%	0,01%	0,01%	0,01%	0,02%	k__Fungi;p__Ascomycota;c__Eurotiomycetes;o__Chaetothyriales;f__Herpotrichiellaceae;Other	
0,00%	0,01%	0,00%	0,03%	0,21%	0,00%	0,00%	0,01%	k__Fungi;p__Ascomycota;c__Leotiomycetes;o__Thelebolales;f__Pseudeurotiaceae	Pseudeurotium
0,01%	0,05%	0,08%	0,03%	0,04%	0,01%	0,02%	0,02%	k__Fungi;Other;Other;Other;Other;Other	
0,00%	0,11%	0,02%	0,03%	0,00%	0,06%	0,02%	0,01%	k__Fungi;p__Basidiomycota;Other;Other;Other;Other	
0,00%	0,00%	0,00%	0,25%	0,00%	0,00%	0,00%	0,00%	k__Fungi;p__Basidiomycota;c__Agaricomycetes;o__Gomphales;f__Gomphaceae	Ramaria
0,01%	0,07%	0,02%	0,02%	0,02%	0,01%	0,02%	0,06%	k__Fungi;p__Ascomycota;c__Sordariomycetes;o__Coniochaetales;f__Coniochaetaceae	Lecythophora
0,01%	0,08%	0,02%	0,01%	0,02%	0,01%	0,02%	0,07%	k__Fungi;p__Ascomycota;c__Sordariomycetes;o__Hypocreales;f__Nectriaceae	Neonectria
0,01%	0,05%	0,02%	0,03%	0,04%	0,02%	0,03%	0,04%	k__Fungi;p__Ascomycota;c__Leotiomycetes;o__Thelebolales;f__Pseudeurotiaceae	Geomyces
0,02%	0,05%	0,02%	0,02%	0,03%	0,02%	0,02%	0,03%	k__Fungi;p__Ascomycota;c__Dothideomycetes;o__Capnodiales;f__Cladosporiaceae	Cladosporium
0,02%	0,04%	0,04%	0,03%	0,02%	0,02%	0,02%	0,03%	k__Fungi;p__Ascomycota;c__Saccharomycetes;o__Saccharomycetales;f__Dipodascaceae	Geotrichum
0,02%	0,06%	0,03%	0,03%	0,01%	0,02%	0,02%	0,01%	k__Fungi;p__Basidiomycota;c__Agaricomycetes;o__Auriculariales;f__Hyaloriaceae	Protodontia
0,03%	0,11%	0,01%	0,01%	0,00%	0,00%	0,01%	0,00%	k__Fungi;p__Basidiomycota;c__Geminibasidiomycetes;o__Geminibasidiales;f__Geminibasidiaceae	Geminibasidium
0,06%	0,02%	0,01%	0,01%	0,01%	0,00%	0,01%	0,06%	k__Fungi;p__Ascomycota;c__Eurotiomycetes;o__Chaetothyriales;f__unidentified	unidentified
0,16%	0,00%	0,00%	0,00%	0,00%	0,00%	0,00%	0,01%	k__Fungi;p__Basidiomycota;c__Agaricomycetes;o__Russulales;f__Russulaceae	unidentified
0,00%	0,07%	0,06%	0,01%	0,01%	0,00%	0,01%	0,01%	k__Fungi;p__Basidiomycota;c__Agaricomycetes;o__Thelephorales;f__Thelephoraceae	Pseudotomentella
0,00%	0,01%	0,01%	0,02%	0,00%	0,00%	0,01%	0,13%	k__Fungi;p__Ascomycota;c__Leotiomycetes;o__unidentified;f__unidentified	unidentified
0,01%	0,06%	0,01%	0,02%	0,01%	0,01%	0,02%	0,03%	k__Fungi;p__Ascomycota;c__Dothideomycetes;o__Pleosporales;f__Didymellaceae;Other	
0,00%	0,10%	0,01%	0,01%	0,00%	0,00%	0,01%	0,02%	k__Fungi;p__Ascomycota;c__Sordariomycetes;o__Sordariales;f__Sordariales_fam_Incertae_sedis	Ramophialophora
0,01%	0,03%	0,02%	0,02%	0,02%	0,01%	0,02%	0,02%	k__Fungi;p__Ascomycota;c__Leotiomycetes;o__Thelebolales;f__Pseudeurotiaceae;Other	
0,01%	0,02%	0,01%	0,02%	0,03%	0,02%	0,02%	0,02%	k__Fungi;p__Ascomycota;c__Saccharomycetes;o__Saccharomycetales;f__Pichiaceae	Pichia
0,00%	0,02%	0,01%	0,01%	0,08%	0,00%	0,01%	0,02%	k__Fungi;p__Ascomycota;c__Eurotiomycetes;o__Eurotiales;f__Aspergillaceae	Aspergillus
0,05%	0,01%	0,02%	0,01%	0,01%	0,01%	0,01%	0,02%	k__Fungi;p__Ascomycota;c__Sordariomycetes;o__unidentified;f__unidentified	unidentified
0,01%	0,03%	0,02%	0,02%	0,01%	0,01%	0,02%	0,02%	k__Fungi;p__Ascomycota;c__Pezizomycetes;o__Pezizales;f__Sarcoscyphaceae	Desmazierella
0,01%	0,02%	0,02%	0,02%	0,02%	0,01%	0,01%	0,02%	k__Fungi;p__Basidiomycota;c__Tremellomycetes;o__Tremellales;f__Trimorphomycetaceae	Saitozyma
0,00%	0,02%	0,02%	0,03%	0,02%	0,01%	0,02%	0,02%	k__Fungi;p__Ascomycota;c__Sordariomycetes;o__Sordariales;f__unidentified	unidentified
0,01%	0,03%	0,02%	0,01%	0,01%	0,01%	0,02%	0,02%	k__Fungi;p__Ascomycota;c__unidentified;o__unidentified;f__unidentified	unidentified
0,01%	0,03%	0,01%	0,01%	0,01%	0,04%	0,01%	0,02%	k__Fungi;p__Ascomycota;c__Leotiomycetes;o__Helotiales;f__Helotiales_fam_Incertae_sedis	Chalara
0,01%	0,04%	0,01%	0,01%	0,01%	0,01%	0,02%	0,02%	k__Fungi;p__Ascomycota;c__Leotiomycetes;o__Helotiales;f__Helotiaceae	Helicodendron
0,00%	0,00%	0,00%	0,00%	0,00%	0,13%	0,00%	0,00%	k__Fungi;p__Basidiomycota;c__Agaricomycetes;o__Hysterangiales;f__Hysterangiaceae	Hysterangium
0,12%	0,00%	0,00%	0,00%	0,00%	0,00%	0,00%	0,00%	k__Fungi;p__Ascomycota;c__Saccharomycetes;o__Saccharomycetales;f__Saccharomycodaceae	Hanseniaspora
0,01%	0,03%	0,01%	0,02%	0,02%	0,01%	0,01%	0,01%	k__Fungi;p__Ascomycota;c__Leotiomycetes;o__Helotiales;f__Dermateaceae	Cryptosporiopsis
0,01%	0,01%	0,03%	0,02%	0,01%	0,01%	0,02%	0,01%	k__Fungi;p__Basidiomycota;c__Agaricomycetes;o__Agaricales;f__Tricholomataceae	Pseudoclitocybe
0,00%	0,01%	0,00%	0,02%	0,01%	0,00%	0,06%	0,00%	k__Fungi;p__Ascomycota;c__Leotiomycetes;o__Helotiales;f__Helotiaceae	Meliniomyces
0,01%	0,03%	0,01%	0,01%	0,01%	0,01%	0,01%	0,02%	k__Fungi;p__Ascomycota;c__Leotiomycetes;o__Helotiales;f__Dermateaceae;Other	
0,01%	0,01%	0,01%	0,02%	0,02%	0,02%	0,02%	0,01%	k__Fungi;p__Ascomycota;c__Saccharomycetes;o__Saccharomycetales;f__Saccharomycetaceae	Williopsis
0,00%	0,01%	0,02%	0,02%	0,01%	0,01%	0,02%	0,02%	k__Fungi;p__Basidiomycota;c__Agaricomycetes;o__Agaricales;f__Lycoperdaceae	Lycoperdon
0,02%	0,04%	0,01%	0,01%	0,01%	0,01%	0,01%	0,01%	k__Fungi;p__Basidiomycota;c__Agaricomycetes;o__Thelephorales;f__Thelephoraceae	Odontia
0,02%	0,01%	0,01%	0,01%	0,01%	0,01%	0,01%	0,02%	k__Fungi;p__Ascomycota;c__Dothideomycetes;o__Capnodiales;f__unidentified	unidentified
0,00%	0,00%	0,01%	0,00%	0,01%	0,00%	0,01%	0,06%	k__Fungi;p__Basidiomycota;c__Agaricomycetes;o__Cantharellales;f__Clavulinaceae	Clavulina
0,00%	0,01%	0,07%	0,00%	0,00%	0,00%	0,00%	0,01%	k__Fungi;p__Ascomycota;c__Sordariomycetes;o__Sordariales;f__Lasiosphaeriaceae;Other	
0,04%	0,01%	0,00%	0,00%	0,00%	0,02%	0,00%	0,01%	k__Fungi;p__Ascomycota;c__Leotiomycetes;o__Helotiales;f__Helotiales_fam_Incertae_sedis	Leohumicola
0,01%	0,02%	0,01%	0,01%	0,01%	0,01%	0,01%	0,02%	k__Fungi;p__Ascomycota;c__Sordariomycetes;o__Hypocreales;f__Nectriaceae	Fusarium
0,00%	0,03%	0,01%	0,02%	0,01%	0,00%	0,01%	0,02%	k__Fungi;p__Ascomycota;c__Leotiomycetes;o__Thelebolales;f__Pseudeurotiaceae	Pseudogymnoascus
0,01%	0,03%	0,01%	0,01%	0,01%	0,00%	0,01%	0,02%	k__Fungi;p__Ascomycota;c__Sordariomycetes;o__Hypocreales;f__Hypocreales_fam_Incertae_sedis	Acremonium
0,01%	0,02%	0,01%	0,01%	0,01%	0,01%	0,00%	0,01%	k__Fungi;p__Ascomycota;c__Saccharomycetes;o__Saccharomycetales;f__Dipodascaceae	unidentified
0,01%	0,02%	0,01%	0,01%	0,01%	0,00%	0,01%	0,01%	k__Fungi;p__Ascomycota;c__Dothideomycetes;o__Pleosporales;f__unidentified	unidentified
0,00%	0,01%	0,02%	0,02%	0,00%	0,01%	0,02%	0,01%	k__Fungi;p__Basidiomycota;c__Agaricomycetes;o__Agaricales;f__Bolbitiaceae	Conocybe
0,00%	0,01%	0,04%	0,00%	0,00%	0,00%	0,00%	0,01%	k__Fungi;p__Ascomycota;c__Sordariomycetes;o__Sordariales;Other;Other	
0,00%	0,01%	0,01%	0,00%	0,03%	0,01%	0,01%	0,00%	k__Fungi;p__Ascomycota;c__Eurotiomycetes;o__Eurotiales;f__Aspergillaceae	Monascus
0,00%	0,01%	0,01%	0,00%	0,01%	0,00%	0,04%	0,01%	k__Fungi;p__Ascomycota;c__Sordariomycetes;o__Hypocreales;f__Cordycipitaceae	Beauveria
0,00%	0,01%	0,01%	0,02%	0,00%	0,01%	0,02%	0,01%	k__Fungi;p__Basidiomycota;c__Agaricomycetes;o__Geastrales;f__Sclerogastraceae	Sclerogaster
0,01%	0,02%	0,00%	0,00%	0,01%	0,00%	0,00%	0,03%	k__Fungi;p__Ascomycota;c__Sordariomycetes;o__Hypocreales;f__Nectriaceae	Ilyonectria

0,01%	0,01%	0,01%	0,01%	0,01%	0,00%	0,00%	0,02%	k__Fungi;p__Ascomycota;c__Pezizomycetes;o__Pezizales;f__Helvellaceae	Helvella
0,01%	0,01%	0,01%	0,00%	0,01%	0,01%	0,01%	0,01%	k__Fungi;p__Ascomycota;c__Sordariomycetes;o__Calosphaeriales;f__Pleurostomataceae	Pleurostoma
0,05%	0,00%	0,00%	0,00%	0,00%	0,00%	0,00%	0,01%	k__Fungi;p__Basidiomycota;c__Agaricomycetes;o__Agaricales;f__Lyophyllaceae	Lyophyllum
0,00%	0,02%	0,01%	0,01%	0,00%	0,00%	0,00%	0,01%	k__Fungi;p__Ascomycota;c__Sordariomycetes;o__Sordariales;f__Chaetomiaceae	Chaetomium
0,00%	0,01%	0,01%	0,01%	0,01%	0,01%	0,01%	0,01%	k__Fungi;p__Basidiomycota;c__Agaricomycetes;o__Agaricales;f__Omphalotaceae	Rhodocollybia
0,00%	0,01%	0,02%	0,01%	0,00%	0,00%	0,01%	0,00%	k__Fungi;p__Basidiomycota;c__Tremellomycetes;o__Trichosporonales;f__Trichosporonaceae	Apiotrichum
0,00%	0,01%	0,01%	0,01%	0,02%	0,00%	0,01%	0,01%	k__Fungi;p__Ascomycota;c__Eurotiomycetes;o__Eurotiales;f__Trichocomaceae	Talaromyces
0,00%	0,01%	0,01%	0,01%	0,01%	0,00%	0,01%	0,01%	k__Fungi;p__Ascomycota;c__Saccharomycetes;o__Saccharomycetales;f__unidentified	unidentified
0,00%	0,03%	0,00%	0,01%	0,00%	0,00%	0,00%	0,02%	k__Fungi;p__Basidiomycota;c__Tremellomycetes;o__Cystofilobasidiales;f__Mrakiaceae	Tausonia
0,01%	0,01%	0,01%	0,01%	0,01%	0,01%	0,00%	0,01%	k__Fungi;p__Basidiomycota;c__Microbotryomycetes;o__Sporidiobolales;f__Sporidiobolaceae	Rhodotorula
0,00%	0,01%	0,02%	0,01%	0,00%	0,00%	0,01%	0,01%	k__Fungi;p__Basidiomycota;c__Agaricomycetes;o__Tremellodendropsidales;f__unidentified	unidentified
0,00%	0,00%	0,05%	0,00%	0,00%	0,00%	0,00%	0,00%	k__Fungi;p__Basidiomycota;c__Agaricomycetes;o__Agaricales;f__Hebelomataceae	unidentified
0,00%	0,01%	0,01%	0,00%	0,00%	0,00%	0,01%	0,03%	k__Fungi;p__Basidiomycota;c__Agaricomycetes;o__Agaricales;f__Tricholomataceae	Melanoleuca
0,00%	0,01%	0,01%	0,02%	0,01%	0,00%	0,00%	0,00%	k__Fungi;p__Basidiomycota;c__Agaricomycetes;o__Boletales;f__Melanogastraceae	Melanogaster
0,00%	0,01%	0,00%	0,01%	0,00%	0,01%	0,01%	0,01%	k__Fungi;p__Basidiomycota;c__Agaricomycetes;o__Cantharellales;f__Clavulinaceae	Membranomyces
0,01%	0,01%	0,01%	0,00%	0,01%	0,00%	0,01%	0,01%	k__Fungi;p__Ascomycota;c__Sordariomycetes;o__Hypocreales;f__Nectriaceae;Other	
0,00%	0,01%	0,01%	0,00%	0,01%	0,00%	0,01%	0,02%	k__Fungi;p__Ascomycota;c__Sordariomycetes;o__Hypocreales;f__Nectriaceae	Volutella
0,00%	0,01%	0,01%	0,01%	0,01%	0,00%	0,01%	0,01%	k__Fungi;p__Ascomycota;c__Dothideomycetes;o__Dothideales;f__Aureobasidiaceae	Aureobasidium
0,00%	0,01%	0,00%	0,01%	0,00%	0,00%	0,02%	0,00%	k__Fungi;p__Ascomycota;c__Leotiomycetes;o__Helotiales;f__Hyaloscyphaceae	unidentified
0,00%	0,01%	0,01%	0,01%	0,00%	0,01%	0,01%	0,01%	k__Fungi;p__Basidiomycota;c__Agaricomycetes;o__unidentified;f__unidentified	unidentified
0,01%	0,01%	0,00%	0,01%	0,01%	0,00%	0,01%	0,01%	k__Fungi;p__Ascomycota;c__Sordariomycetes;o__Myrmecridiales;f__unidentified	unidentified
0,00%	0,01%	0,01%	0,01%	0,00%	0,00%	0,01%	0,01%	k__Fungi;p__Basidiomycota;c__Agaricomycetes;o__Agaricales;f__Agaricaceae	Lepiota
0,01%	0,01%	0,01%	0,00%	0,00%	0,00%	0,01%	0,01%	k__Fungi;p__Ascomycota;c__Dothideomycetes;o__Pleosporales;Other;Other	
0,01%	0,01%	0,01%	0,00%	0,01%	0,00%	0,00%	0,01%	k__Fungi;p__Ascomycota;c__Pezizomycetes;o__Pezizales;f__Pyronemataceae	Scutellinia
0,00%	0,02%	0,01%	0,00%	0,00%	0,00%	0,01%	0,00%	k__Fungi;p__Ascomycota;c__Dothideomycetes;o__Pleosporales;f__Pleosporaceae	Alternaria
0,00%	0,00%	0,03%	0,00%	0,00%	0,00%	0,00%	0,00%	k__Fungi;p__Basidiomycota;c__Agaricomycetes;o__Polyporales;f__unidentified	unidentified
0,00%	0,00%	0,05%	0,00%	0,00%	0,00%	0,00%	0,00%	k__Fungi;p__Basidiomycota;c__Agaricomycetes;o__Trechisporales;f__unidentified	unidentified
0,00%	0,00%	0,00%	0,00%	0,04%	0,00%	0,00%	0,00%	k__Fungi;p__Ascomycota;c__Eurotiomycetes;o__Eurotiales;f__Aspergillaceae;Other	
0,00%	0,01%	0,01%	0,00%	0,01%	0,00%	0,01%	0,01%	k__Fungi;p__Ascomycota;c__Dothideomycetes;o__Pleosporales;f__Sporormiaceae	Westerdykella
0,00%	0,01%	0,00%	0,01%	0,00%	0,00%	0,01%	0,00%	k__Fungi;p__Basidiomycota;c__Agaricomycetes;o__Thelephorales;f__Thelephoraceae;Other	
0,00%	0,01%	0,01%	0,01%	0,01%	0,00%	0,00%	0,00%	k__Fungi;p__Ascomycota;c__Pezizomycetes;o__Pezizales;f__Pyronemataceae	unidentified
0,00%	0,01%	0,01%	0,01%	0,00%	0,00%	0,01%	0,00%	k__Fungi;p__Basidiomycota;c__Agaricomycetes;Other;Other;Other	
0,00%	0,02%	0,00%	0,01%	0,00%	0,00%	0,00%	0,01%	k__Fungi;p__Ascomycota;c__Sordariomycetes;o__Sordariales;f__Chaetomiaceae	unidentified
0,00%	0,01%	0,00%	0,00%	0,01%	0,01%	0,01%	0,00%	k__Fungi;p__Ascomycota;c__Saccharomycetes;o__Saccharomycetales;Other;Other	
0,00%	0,01%	0,00%	0,00%	0,00%	0,00%	0,00%	0,01%	k__Fungi;p__Basidiomycota;c__Tremellomycetes;o__Holtermanniales;f__Holtermanniales_fam_Incertae_sedis	Holtermanniella
0,00%	0,01%	0,01%	0,01%	0,00%	0,00%	0,01%	0,01%	k__Fungi;p__Basidiomycota;c__Agaricomycetes;o__Agaricales;f__Tricholomataceae	Mycenella
0,00%	0,00%	0,01%	0,00%	0,01%	0,00%	0,01%	0,00%	k__Fungi;p__Ascomycota;c__Eurotiomycetes;o__Onygenales;f__Ajellomycetaceae	Histoplasma
0,00%	0,00%	0,00%	0,00%	0,00%	0,00%	0,00%	0,03%	k__Fungi;p__Basidiomycota;c__Agaricomycetes;o__Agaricales;f__Agaricaceae	Cystolepiota
0,00%	0,01%	0,00%	0,02%	0,01%	0,00%	0,01%	0,00%	k__Fungi;p__Ascomycota;c__Eurotiomycetes;o__Onygenales;f__Onygenales_fam_Incertae_sedis	Chrysosporium
0,00%	0,02%	0,01%	0,00%	0,01%	0,00%	0,00%	0,00%	k__Fungi;p__Ascomycota;c__Leotiomycetes;o__Rhytismatales;f__Rhytismataceae	Lophodermium
0,00%	0,01%	0,01%	0,00%	0,00%	0,00%	0,01%	0,01%	k__Fungi;p__Ascomycota;c__Saccharomycetes;o__Saccharomycetales;f__Debaryomycetaceae	Schwanniomyces
0,00%	0,01%	0,01%	0,01%	0,00%	0,00%	0,01%	0,01%	k__Fungi;p__Basidiomycota;c__Agaricomycetes;o__Agaricales;f__Lyophyllaceae	Tephrocybe
0,00%	0,01%	0,01%	0,00%	0,00%	0,00%	0,00%	0,00%	k__Fungi;p__Basidiomycota;c__Agaricomycetes;o__Agaricales;f__Tricholomataceae	Paralepista
0,00%	0,01%	0,00%	0,00%	0,01%	0,00%	0,00%	0,01%	k__Fungi;p__Ascomycota;c__Dothideomycetes;o__Venturiales;f__Venturiaceae	Venturia
0,00%	0,01%	0,00%	0,00%	0,00%	0,00%	0,00%	0,01%	k__Fungi;p__Ascomycota;c__Dothideomycetes;o__Venturiales;f__Venturiaceae;Other	
0,00%	0,01%	0,00%	0,00%	0,00%	0,00%	0,00%	0,01%	k__Fungi;p__Ascomycota;c__Sordariomycetes;o__Hypocreales;f__Nectriaceae	unidentified
0,00%	0,01%	0,01%	0,00%	0,00%	0,00%	0,00%	0,01%	k__Fungi;p__Ascomycota;c__Dothideomycetes;o__Venturiales;f__Venturiaceae	unidentified
0,00%	0,00%	0,01%	0,00%	0,00%	0,00%	0,00%	0,01%	k__Fungi;p__Chytridiomycota;Other;Other;Other;Other	
0,01%	0,01%	0,00%	0,00%	0,00%	0,00%	0,00%	0,01%	k__Fungi;p__Ascomycota;c__Eurotiomycetes;o__Chaetothyriales;Other;Other	
0,00%	0,00%	0,01%	0,00%	0,00%	0,00%	0,01%	0,00%	k__Fungi;p__Basidiomycota;c__Agaricomycetes;o__Agaricales;f__Tricholomataceae	Macrocystidia
0,00%	0,01%	0,00%	0,01%	0,00%	0,00%	0,00%	0,01%	k__Fungi;p__Basidiomycota;c__Agaricomycetes;o__Auriculariales;f__Auriculariales_fam_Incertae_sedis	Heterochaetella
0,00%	0,00%	0,01%	0,00%	0,00%	0,00%	0,01%	0,00%	k__Fungi;p__Ascomycota;c__Saccharomycetes;o__Saccharomycetales;f__Metschnikowiaceae	Clavispora
0,00%	0,01%	0,01%	0,00%	0,00%	0,00%	0,01%	0,00%	k__Fungi;p__Mucoromycota;c__Umbelopsidomycetes;o__Umbelopsidales;f__Umbelopsidaceae	Umbelopsis
0,00%	0,00%	0,00%	0,01%	0,00%	0,00%	0,00%	0,02%	k__Fungi;p__Basidiomycota;c__Cystobasidiomycetes;o__Erythrobasidiales;f__Erythrobasidiales_fam_Incertae_sedis	Sakaguchia
0,00%	0,01%	0,01%	0,00%	0,00%	0,00%	0,00%	0,00%	k__Fungi;p__Ascomycota;c__Sordariomycetes;o__Sordariales;f__Lasiosphaeriaceae	unidentified
0,01%	0,00%	0,00%	0,00%	0,00%	0,00%	0,00%	0,01%	k__Fungi;p__Ascomycota;c__Sordariomycetes;o__Sordariales;f__Sordariaceae	unidentified
0,00%	0,00%	0,01%	0,00%	0,00%	0,00%	0,02%	0,00%	k__Fungi;p__Basidiomycota;c__Agaricomycetes;o__Agaricales;f__Bolbitiaceae	Agrocybe
0,00%	0,01%	0,01%	0,00%	0,00%	0,00%	0,01%	0,01%	k__Fungi;p__Ascomycota;c__Sordariomycetes;o__Hypocreales;Other;Other	
0,00%	0,00%	0,01%	0,00%	0,00%	0,00%	0,00%	0,00%	k__Fungi;p__Basidiomycota;c__Agaricomycetes;o__Thelephorales;f__Thelephoraceae	Thelephora
0,00%	0,00%	0,01%	0,00%	0,00%	0,00%	0,00%	0,01%	k__Fungi;p__Ascomycota;c__Leotiomycetes;o__Thelebolales;f__Thelebolaceae	Thelebolus
0,00%	0,00%	0,00%	0,00%	0,02%	0,00%	0,00%	0,00%	k__Fungi;p__Ascomycota;c__Eurotiomycetes;o__Eurotiales;f__unidentified	unidentified
0,00%	0,01%	0,00%	0,01%	0,00%	0,00%	0,00%	0,00%	k__Fungi;p__Ascomycota;c__Dothideomycetes;Other;Other;Other	
0,00%	0,01%	0,01%	0,00%	0,00%	0,00%	0,01%	0,00%	k__Fungi;p__Basidiomycota;c__Agaricomycetes;o__Agaricales;f__Pluteaceae	Pluteus
0,00%	0,01%	0,00%	0,00%	0,00%	0,00%	0,00%	0,00%	k__Fungi;p__Ascomycota;c__Leotiomycetes;o__Helotiales;f__Helotiaceae	Tetracladium
0,00%	0,01%	0,01%	0,00%	0,00%	0,00%	0,00%	0,00%	k__Fungi;p__Ascomycota;c__Dothideomycetes;o__Pleosporales;f__Pleomassariaceae	Prosthemium
0,00%	0,02%	0,00%	0,00%	0,00%	0,00%	0,00%	0,00%	k__Fungi;p__Ascomycota;c__Pezizomycetes;o__Pezizales;f__Pyronemataceae	Rhodoscypha
0,00%	0,01%	0,00%	0,00%	0,00%	0,00%	0,00%	0,00%	k__Fungi;p__Ascomycota;c__Sordariomycetes;o__Microascales;f__Microascaceae	Cephalotrichum
0,00%	0,01%	0,00%	0,00%	0,01%	0,00%	0,00%	0,00%	k__Fungi;p__Ascomycota;c__Eurotiomycetes;o__Onygenales;f__Onygenaceae	Auxarthron
0,00%	0,02%	0,00%	0,00%	0,00%	0,00%	0,00%	0,00%	k__Fungi;p__Basidiomycota;c__Agaricomycetes;o__Hymenochaetales;f__Schizoporaceae	Hyphodontia
0,01%	0,01%	0,00%	0,00%	0,00%	0,00%	0,00%	0,00%	k__Fungi;p__Ascomycota;c__Leotiomycetes;o__Helotiales;f__Vibrisseaceae	Phialocephala
0,00%	0,01%	0,00%	0,00%	0,00%	0,00%	0,00%	0,01%	k__Fungi;p__Ascomycota;c__Eurotiomycetes;o__Chaetothyriales;f__Herpotrichiellaceae	Capronia
0,01%	0,00%	0,00%	0,00%	0,00%	0,00%	0,01%	0,00%	k__Fungi;p__Rozellomycota;c__Rozellomycotina_cls_Incertae_sedis;o__GS11;f__unidentified	unidentified
0,00%	0,00%	0,00%	0,00%	0,00%	0,00%	0,01%	0,01%	k__Fungi;p__Ascomycota;c__Sordariomycetes;o__Coniochaetales;f__Coniochaetaceae;Other	
0,02%	0,00%	0,00%	0,00%	0,00%	0,00%	0,00%	0,00%	k__Fungi;p__Ascomycota;c__Eurotiomycetes;o__Chaetothyriales;f__Herpotrichiellaceae	unidentified
0,01%	0,01%	0,00%	0,00%	0,00%	0,00%	0,00%	0,00%	k__Fungi;p__Basidiomycota;c__Tremellomycetes;o__unidentified;f__unidentified	unidentified
0,00%	0,01%	0,00%	0,00%	0,00%	0,00%	0,00%	0,00%	k__Fungi;p__Basidiomycota;c__Agaricomycetes;o__Agaricales;f__Stephanosporaceae	Lindtneria
0,00%	0,00%	0,01%	0,02%	0,00%	0,00%	0,00%	0,00%	k__Fungi;p__Ascomycota;c__Pezizomycetes;o__Pezizales;f__Pyronemataceae	Tricharina
0,00%	0,01%	0,00%	0,00%	0,00%	0,00%	0,00%	0,00%	k__Fungi;p__Basidiomycota;c__Microbotryomycetes;o__Leucosporidiales;f__unidentified	unidentified
0,00%	0,01%	0,00%	0,00%	0,00%	0,00%	0,00%	0,01%	k__Fungi;p__Ascomycota;c__Pezizomycotina_cls_Incertae_sedis;o__Pezizomycotina_ord_Incertae_sedis; f__Pezizomycotina_fam_Incertae_sedis	Ciliophora
0,01%	0,01%	0,00%	0,00%	0,00%	0,00%	0,00%	0,00%	k__Fungi;p__Basidiomycota;c__Agaricomycetes;o__Agaricales;Other;Other	
0,00%	0,00%	0,00%	0,00%	0,01%	0,00%	0,00%	0,00%	k__Fungi;p__Ascomycota;c__Dothideomycetes;o__Pleosporales;f__Didymellaceae	Phoma
0,00%	0,00%	0,01%	0,00%	0,00%	0,00%	0,00%	0,00%	k__Fungi;p__Basidiomycota;c__Agaricomycetes;o__Agaricales;f__Agaricaceae	Coprinus
0,00%	0,00%	0,00%	0,00%	0,00%	0,00%	0,00%	0,01%	k__Fungi;p__Ascomycota;c__Dothideomycetes;o__Pleosporales;f__Melanommataceae	unidentified
0,00%	0,00%	0,01%	0,00%	0,00%	0,00%	0,00%	0,00%	k__Fungi;p__Basidiomycota;c__Agaricomycetes;o__Polyporales;f__Hyphodermataceae	Hyphoderma
0,00%	0,00%	0,00%	0,00%	0,00%	0,00%	0,00%	0,02%	k__Fungi;p__Basidiomycota;c__Agaricomycetes;o__Sebacinales;f__Sebacinaceae	unidentified
0,00%	0,00%	0,00%	0,00%	0,00%	0,00%	0,00%	0,00%	k__Fungi;p__Basidiomycota;c__Agaricomycetes;o__Russulales;f__Russulaceae	Lactarius
0,00%	0,01%	0,00%	0,00%	0,00%	0,00%	0,00%	0,01%	k__Fungi;p__Ascomycota;c__Sordariomycetes;o__Coniochaetales;f__unidentified	unidentified
0,00%	0,01%	0,01%	0,00%	0,00%	0,00%	0,00%	0,00%	k__Fungi;p__Basidiomycota;c__Agaricomycetes;o__Polyporales;f__Ganodermataceae	Ganoderma
0,00%	0,00%	0,01%	0,00%	0,00%	0,00%	0,00%	0,00%	k__Fungi;p__Basidiomycota;c__Agaricomycetes;o__Agaricales;f__unidentified	unidentified
0,00%	0,00%	0,01%	0,00%	0,00%	0,00%	0,01%	0,00%	k__Fungi;p__Basidiomycota;c__Agaricomycetes;o__Agaricales;f__Strophariaceae	Psilocybe
0,00%	0,01%	0,00%	0,00%	0,00%	0,00%	0,00%	0,00%	k__Fungi;p__Basidiomycota;c__Agaricomycetes;o__Hymenochaetales;f__Schizoporaceae	Xylodon
0,00%	0,00%	0,00%	0,00%	0,00%	0,00%	0,00%	0,00%	k__Fungi;p__Ascomycota;c__Eurotiomycetes;o__Onygenales;f__Onygenaceae	unidentified
